# Supplementary figures and images for: Increased genetic contribution to wellbeing during the COVID-19 pandemic
Source: PLoS Genet. 2022 May 19;18(5):e1010135. doi: 10.1371/journal.pgen.1010135 (PMC9119461; doi:10.1371/journal.pgen.1010135)

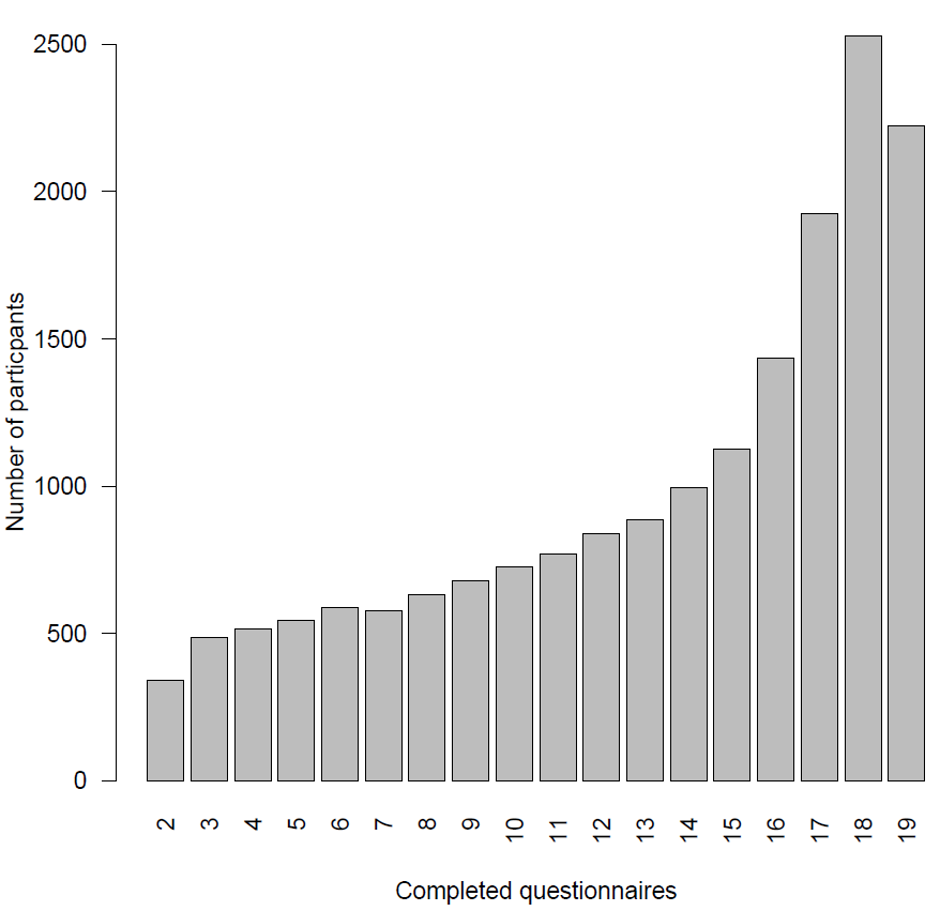

Supplement: S1 Fig — The 17,831 selected participants completed on average 13 questionnaires ranging from 2 to 19. (PNG) [file pgen.1010135.s014.png]

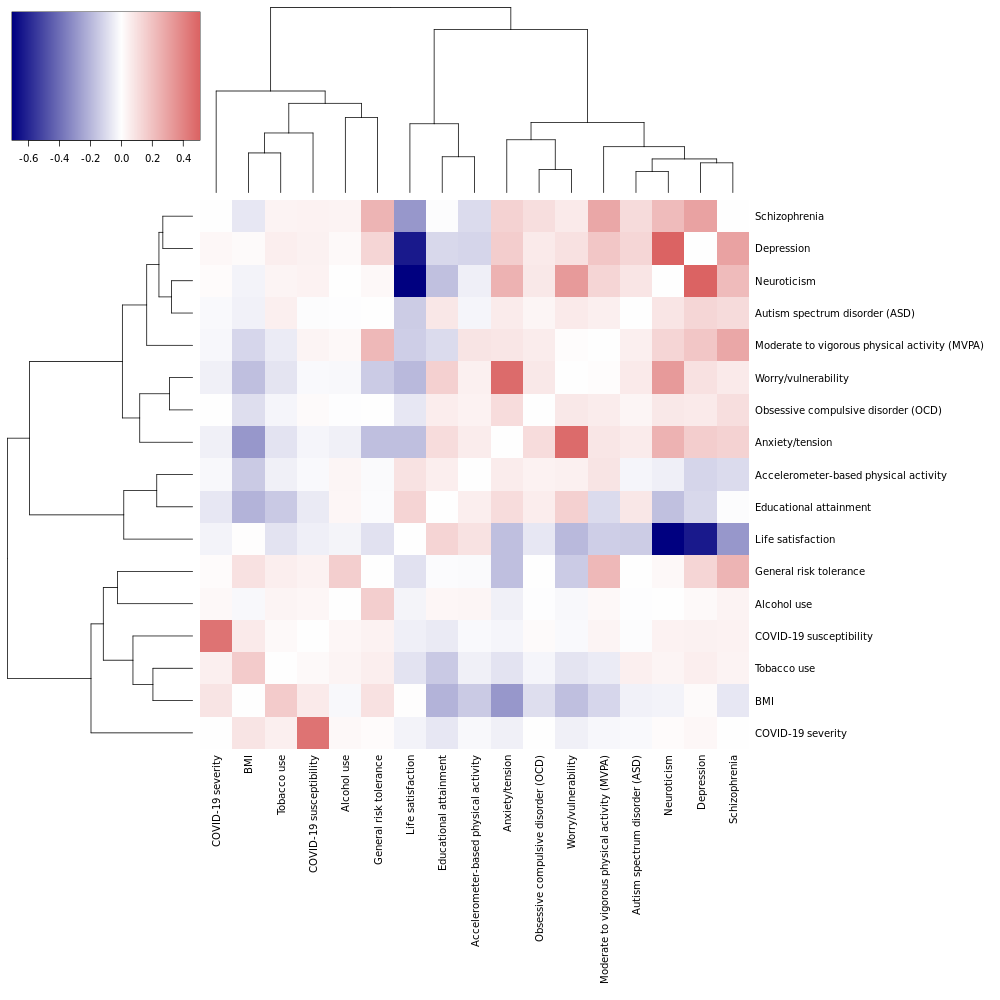

Supplement: S2 Fig — Pearson r correlations between the calculated PGSs of all 27.537 participants. (PNG) [file pgen.1010135.s015.png]

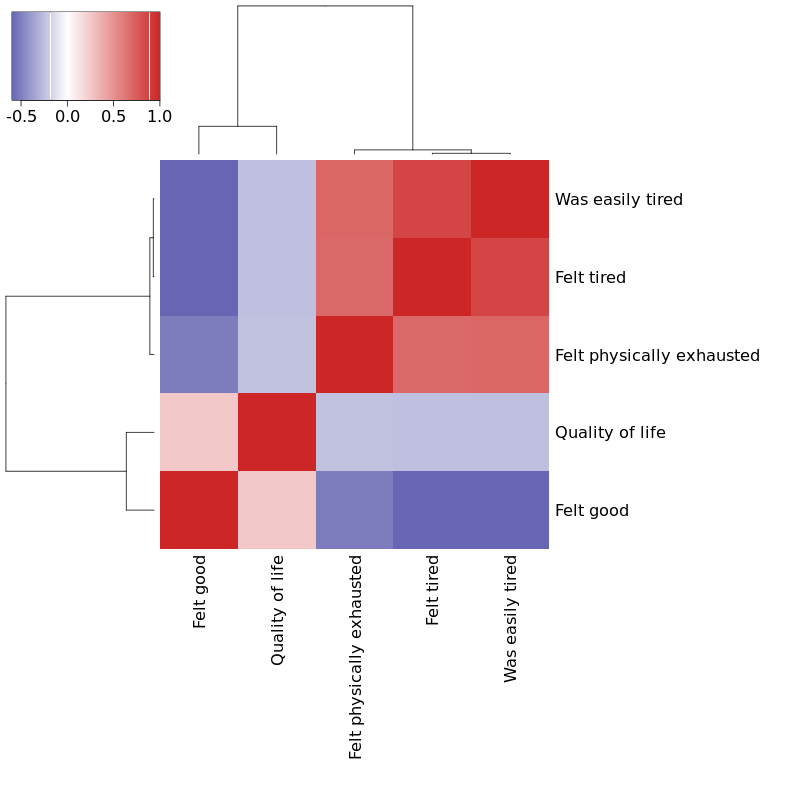

Supplement: S5 Fig — The spearman correlation estimates calculated for the wellbeing and fatigue items for which the impact of genetics has significantly increased during the pandemic. The heatmap shows that the items ‘Was easily tired’, ‘Felt tired’, and ‘Felt physically exhausted’ are highly correlated (Spearman’s rho = 0.69–0.85). The item ‘Felt good is also correlated to these items (Spearman’s rho for ‘Was easily tired’ = -0.60). Quality of life is correlated to the other items as well (Spearman’s rho values for ‘Was easily tired’ and ‘Felt fine’ are equal to -0.25 and 0.25 respectively). The baseline instance of every question was used. From all 27,537 participants the pairwise complete observations were used to handle missing values. (PNG) [file pgen.1010135.s018.png]

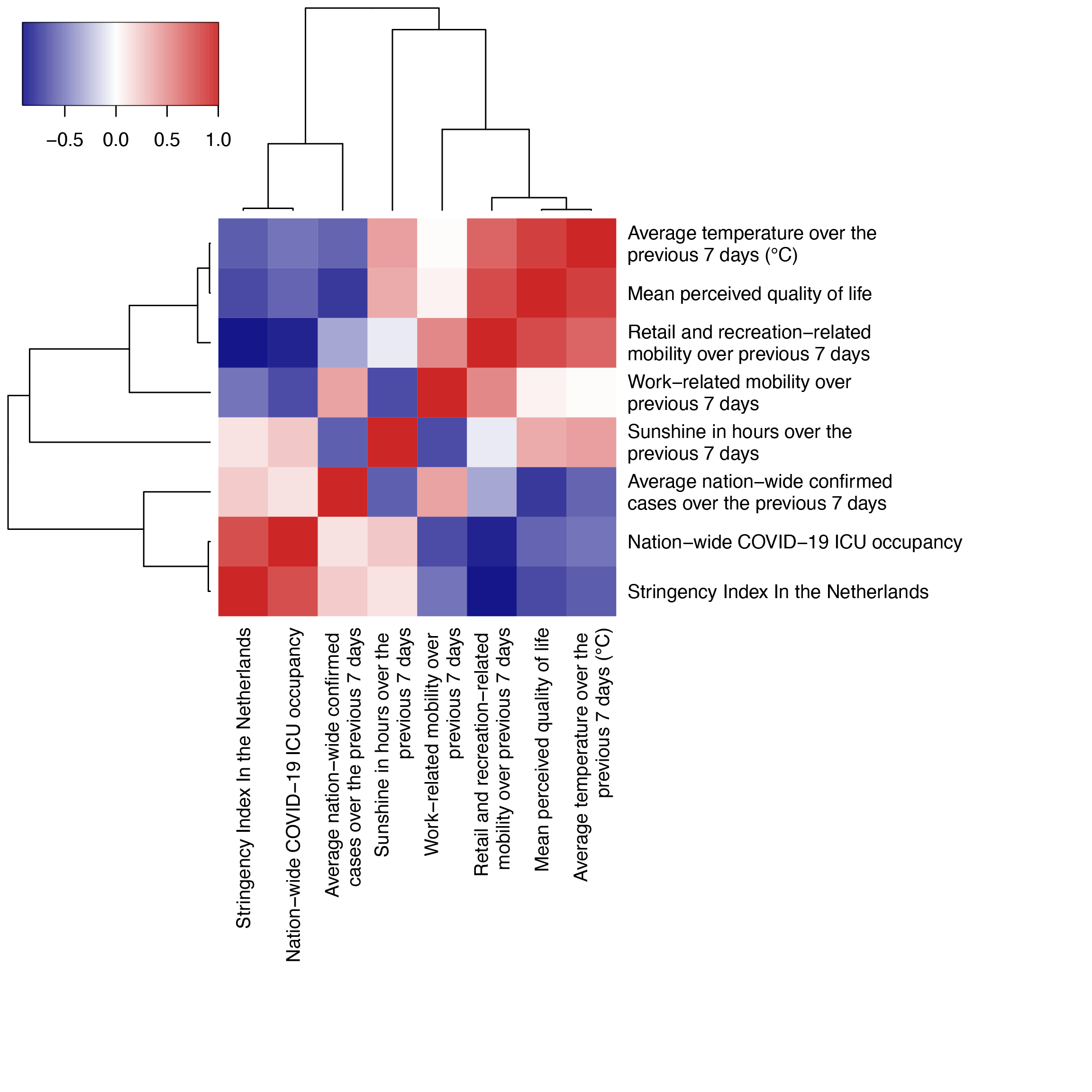

Supplement: S7 Fig — The Pearson r correlation estimates calculated within 7 publicly available variables (averaged over 7 days if indicated) and the mean perceived quality of life. For each of the variables, we extracted the values that coincided with the average response dates for the questionnaires. (PNG) [file pgen.1010135.s020.png]
