# Supplementary material for: Increased genetic contribution to wellbeing during the COVID-19 pandemic
Source: PLoS Genet. 2022 May 19;18(5):e1010135. doi: 10.1371/journal.pgen.1010135 (PMC9119461; doi:10.1371/journal.pgen.1010135)
Supplement: S3 Fig — The baseline associations between PGS and the different outcomes. The baseline association are obtained by a meta-analysis over samples run on Global Screening Array and the HumanCytoSNP-12. (PDF) [file pgen.1010135.s016.pdf]

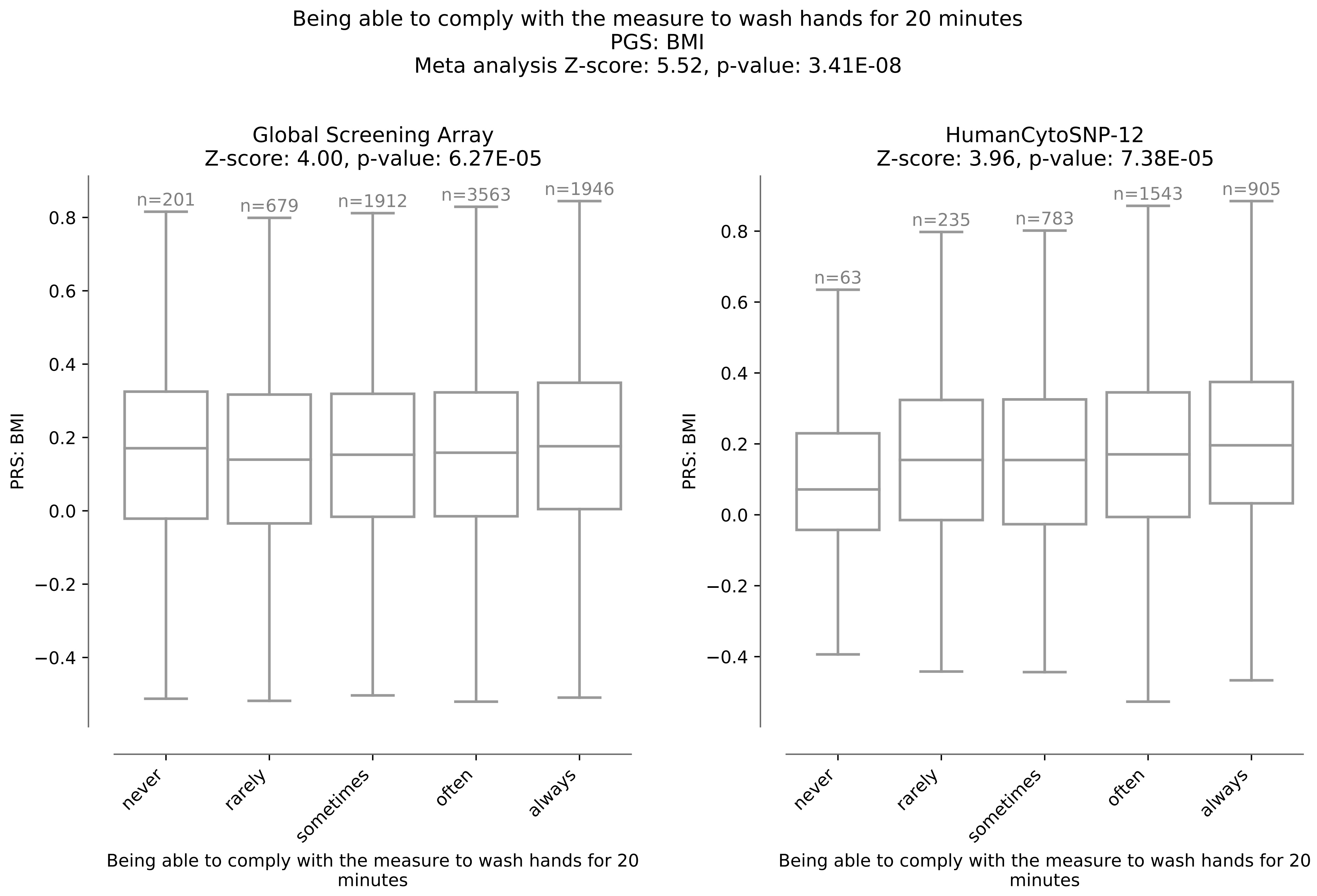

Being able to comply with the measure to wash hands for 20 minutes
PGS: BMI
Meta analysis Z-score: 5.52, p-value: 3.41E-08
Global Screening Array
Z-score: 4.00, p-value: 6.27E-05
n=201
n=679
n=1912
n=3563
n=1946
PRS: BMI
never
rarely
sometimes
often
always
Being able to comply with the measure to wash hands for 20 minutes
HumanCytoSNP-12
Z-score: 3.96, p-value: 7.38E-05
n=63
n=235
n=783
n=1543
n=905
PRS: BMI
never
rarely
sometimes
often
always
Being able to comply with the measure to wash hands for 20 minutes

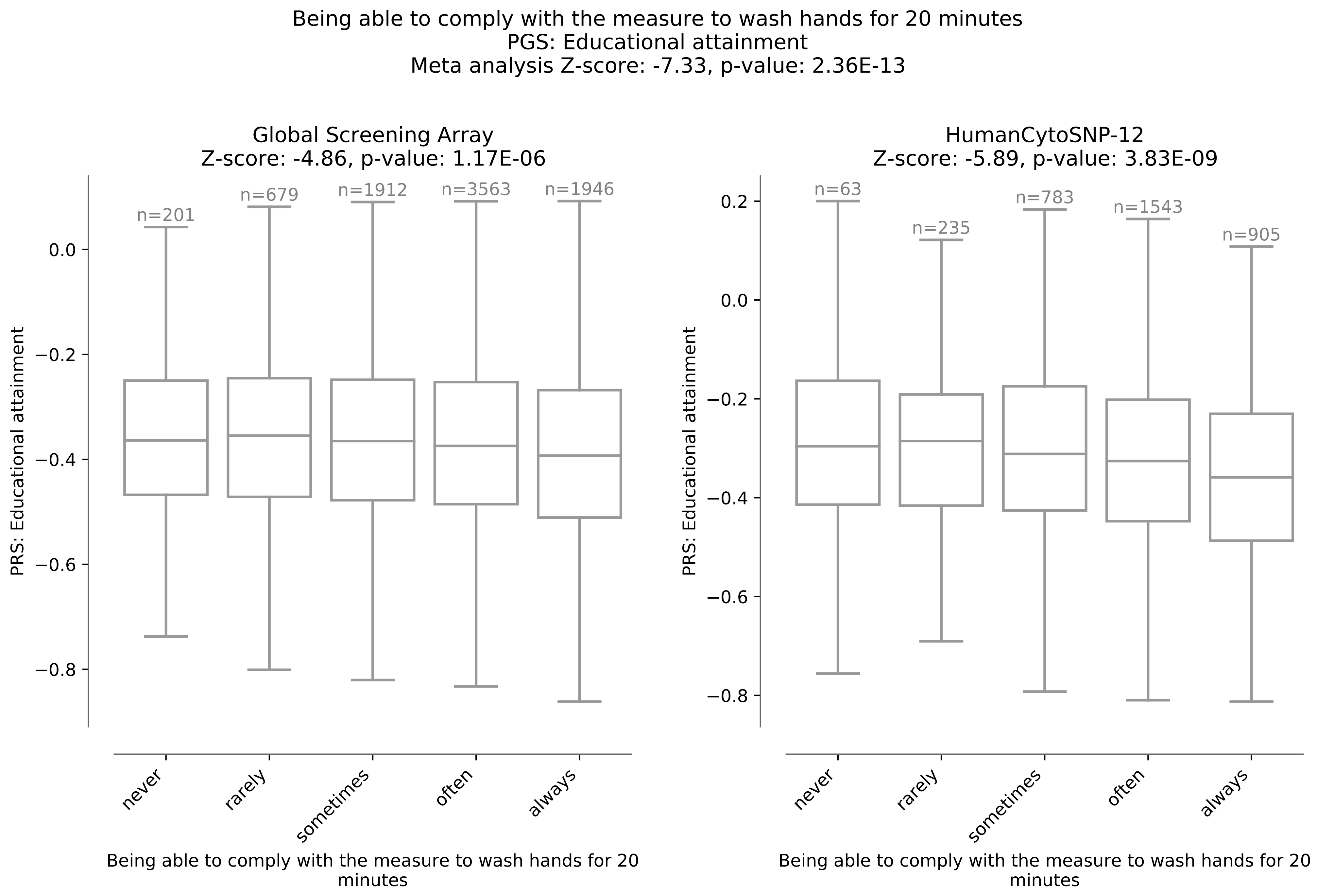
Being able to comply with the measure to wash hands for 20 minutes
PGS: Educational attainment
Meta analysis Z-score: -7.33, p-value: 2.36E-13
Global Screening Array
Z-score: -4.86, p-value: 1.17E-06
n=201
n=679
n=1912
n=3563
n=1946
PRS: Educational attainment
0.0
−0.2
−0.4
−0.6
−0.8
never
rarely
sometimes
often
always
Being able to comply with the measure to wash hands for 20 minutes
HumanCytoSNP-12
Z-score: -5.89, p-value: 3.83E-09
n=63
n=235
n=783
n=1543
n=905
PRS: Educational attainment
0.2
0.0
−0.2
−0.4
−0.6
−0.8
never
rarely
sometimes
often
always
Being able to comply with the measure to wash hands for 20 minutes

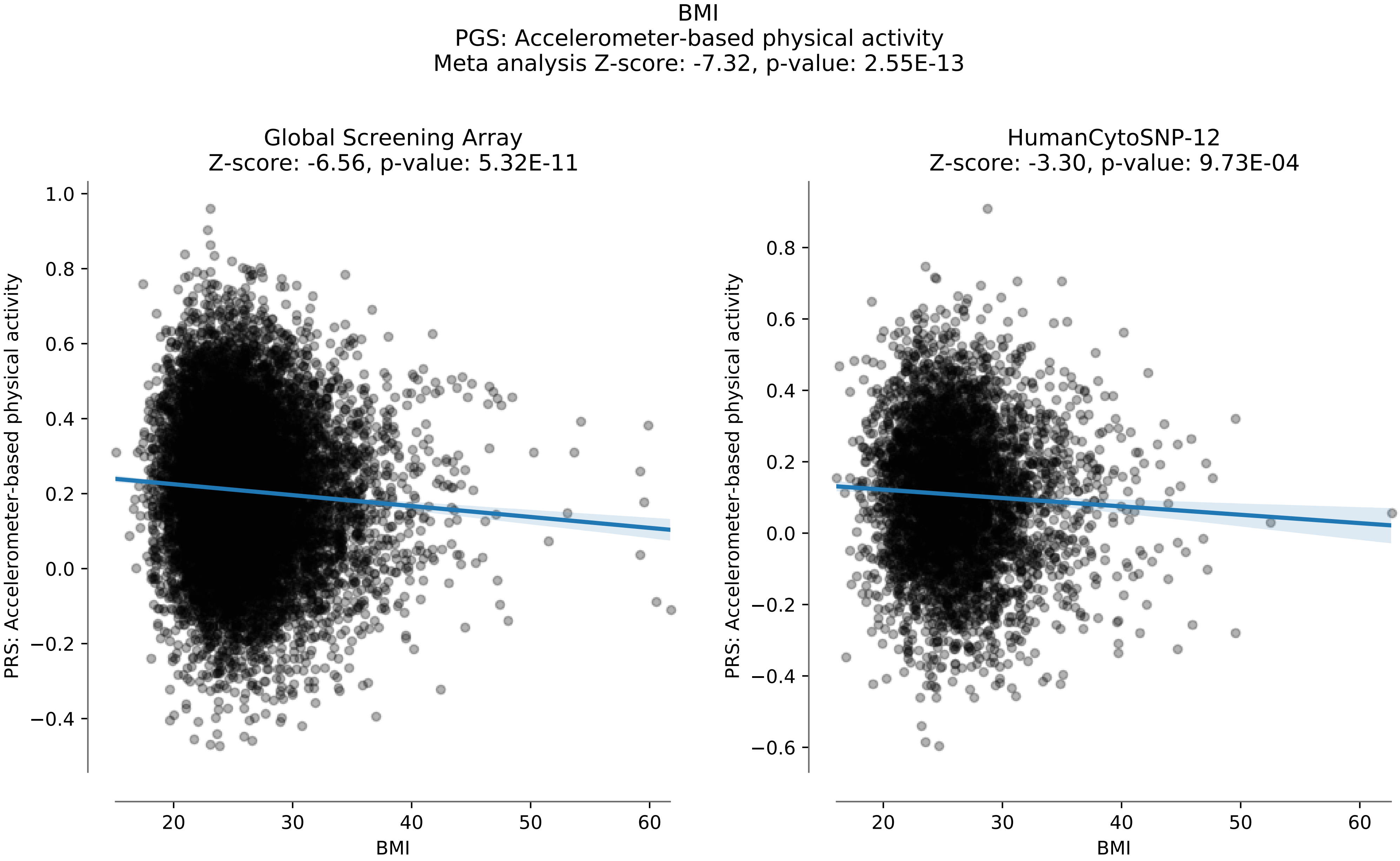
BMI
PGS: Accelerometer-based physical activity
Meta analysis Z-score: -7.32, p-value: 2.55E-13
Global Screening Array
Z-score: -6.56, p-value: 5.32E-11
HumanCytoSNP-12
Z-score: -3.30, p-value: 9.73E-04
PRS: Accelerometer-based physical activity
BMI

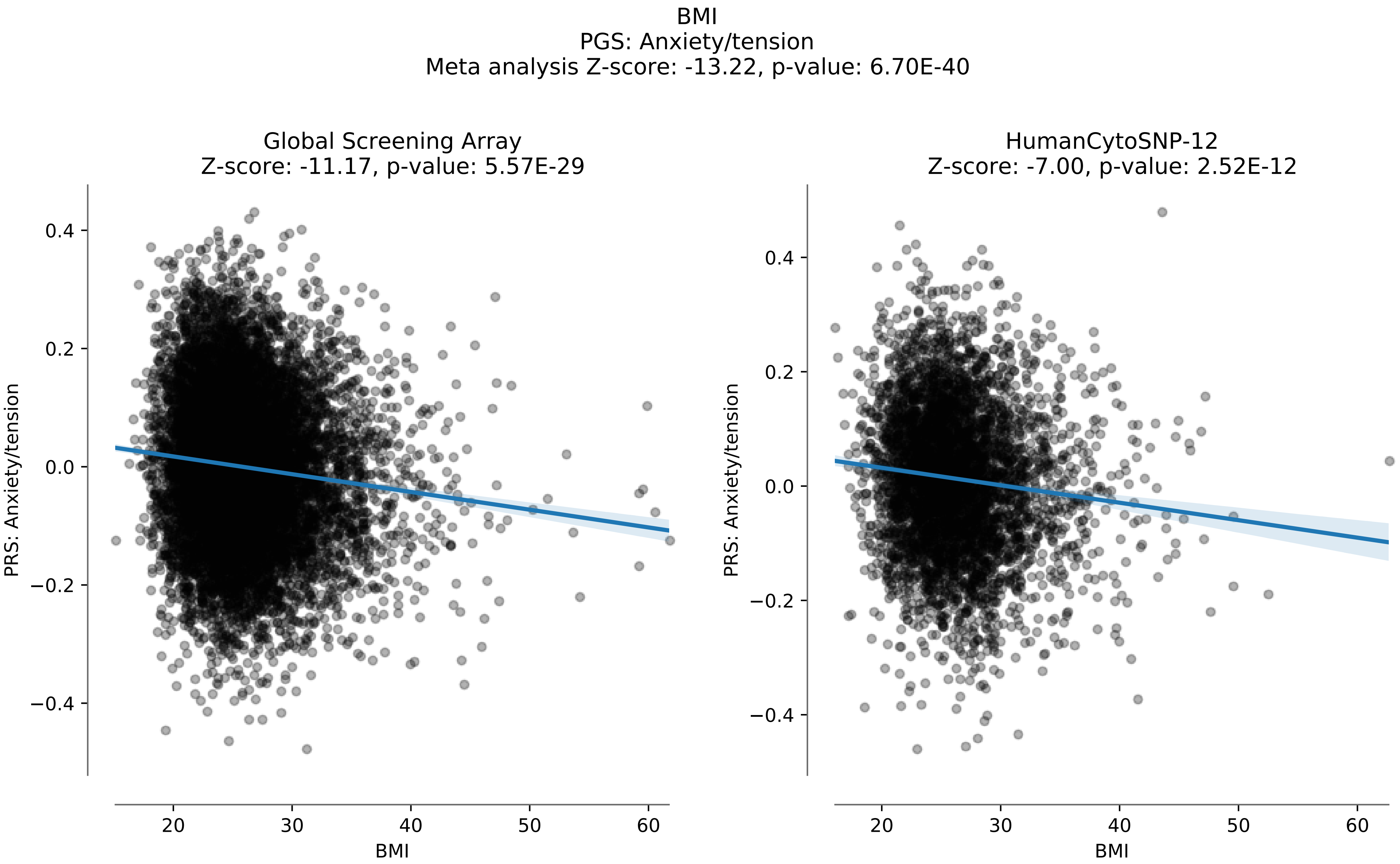

BMI
PGS: Anxiety/tension
Meta analysis Z-score: -13.22, p-value: 6.70E-40
Global Screening Array
Z-score: -11.17, p-value: 5.57E-29
HumanCytoSNP-12
Z-score: -7.00, p-value: 2.52E-12
PRS: Anxiety/tension
BMI
0.4
0.2
0.0
−0.2
−0.4
20
30
40
50
60

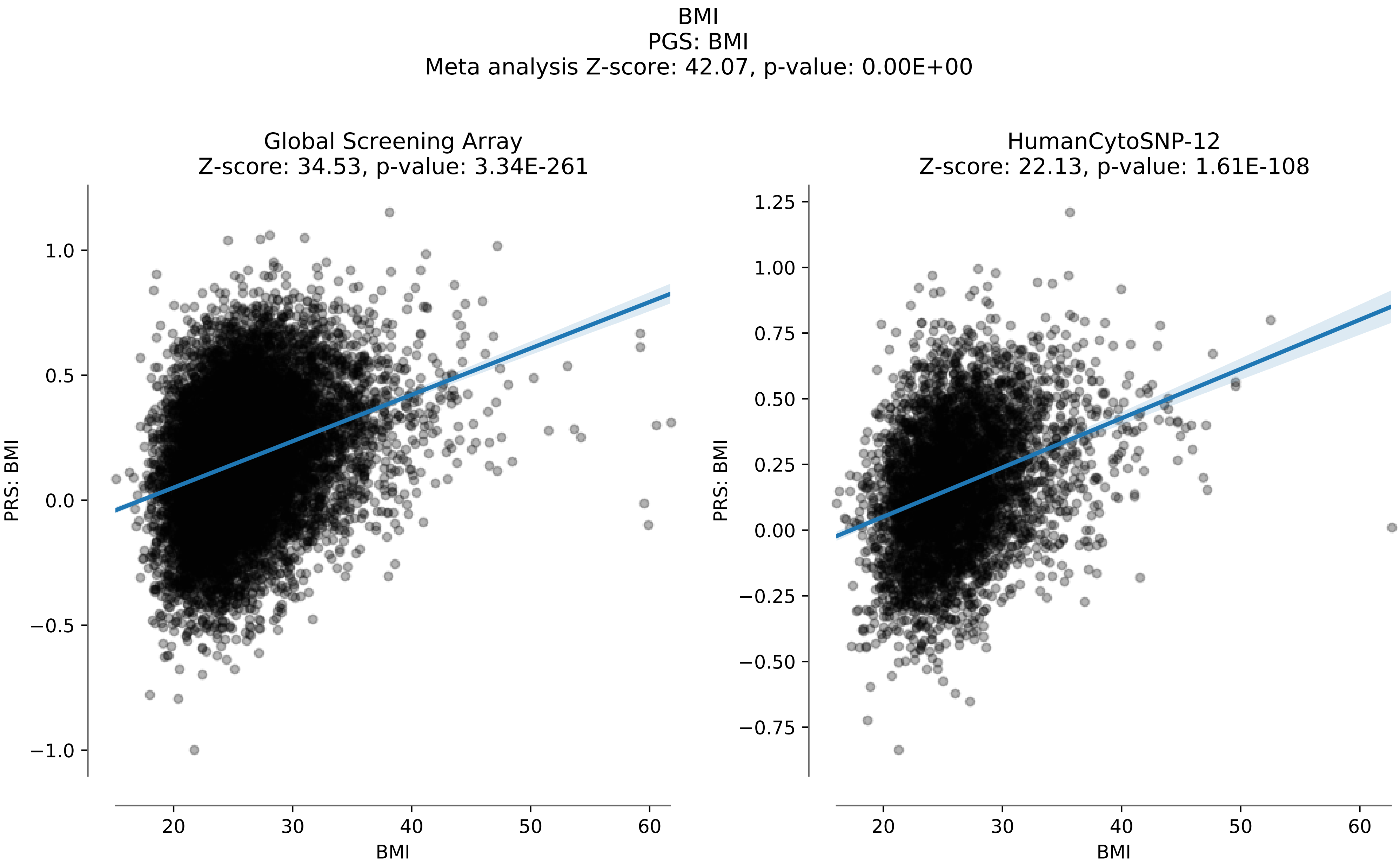
BMI
PGS: BMI
Meta analysis Z-score: 42.07, p-value: 0.00E+00
Global Screening Array
Z-score: 34.53, p-value: 3.34E-261
HumanCytoSNP-12
Z-score: 22.13, p-value: 1.61E-108
PRS: BMI
BMI
PRS: BMI
BMI
1.0
0.5
0.0
−0.5
−1.0
20
30
40
50
60
1.25
1.00
0.75
0.50
0.25
0.00
−0.25
−0.50
−0.75
20
30
40
50
60

BMI
PGS: Tabacco use
Meta analysis Z-score: 10.03, p-value: 1.15E-23

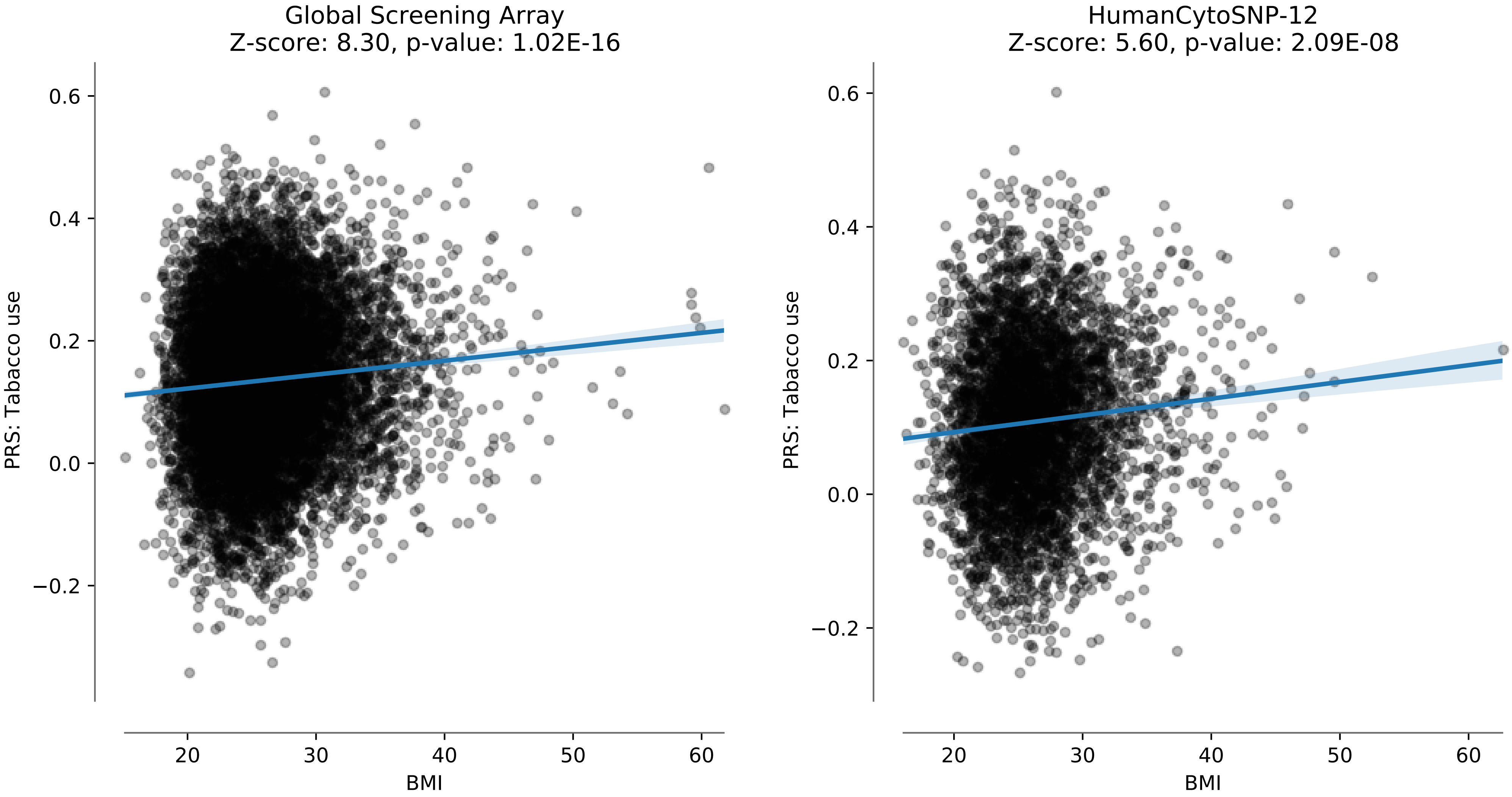

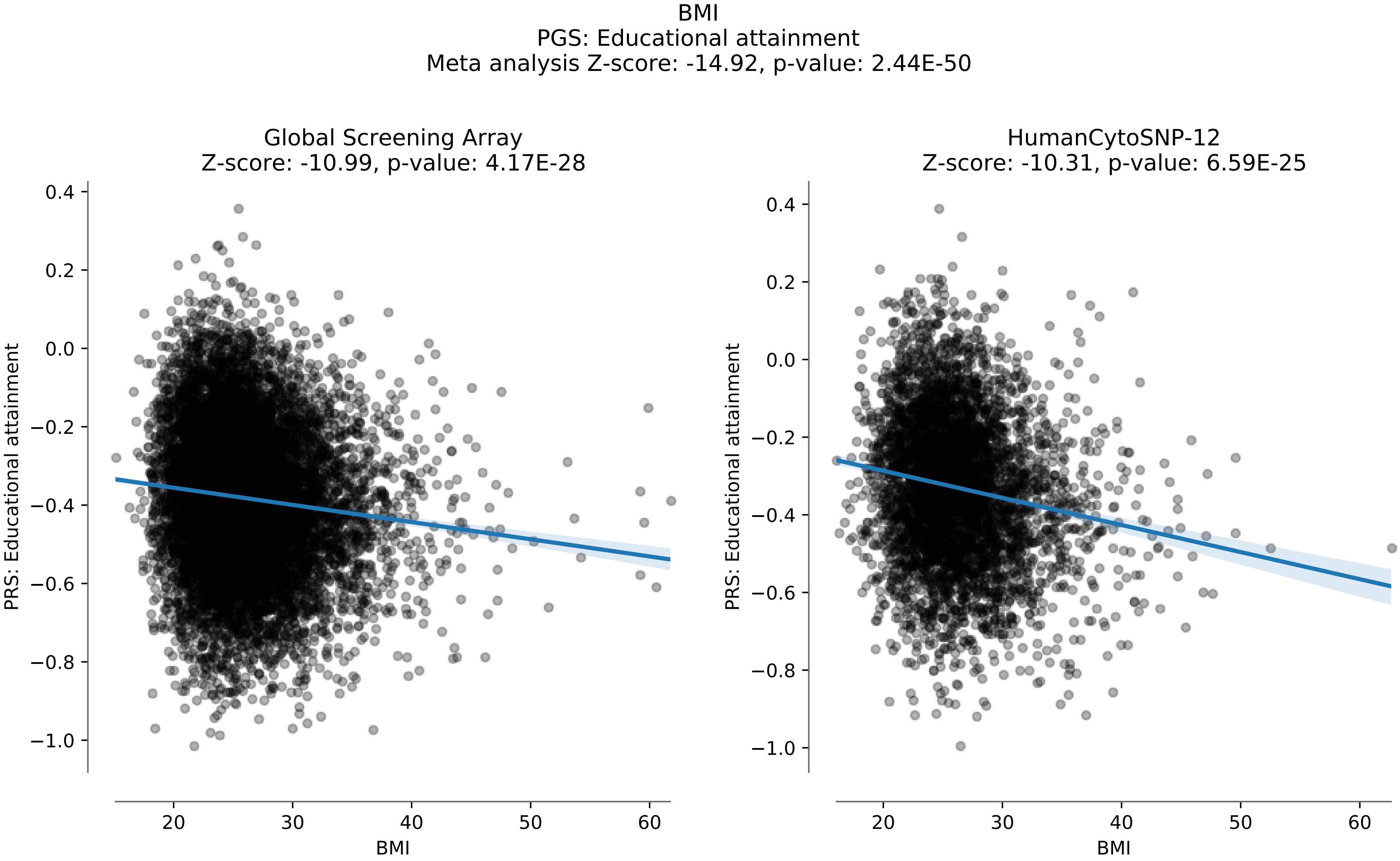
BMI
PGS: Educational attainment
Meta analysis Z-score: -14.92, p-value: 2.44E-50
Global Screening Array
Z-score: -10.99, p-value: 4.17E-28
HumanCytoSNP-12
Z-score: -10.31, p-value: 6.59E-25
PRS: Educational attainment
BMI
0.4
0.2
0.0
−0.2
−0.4
−0.6
−0.8
−1.0
20
30
40
50
60

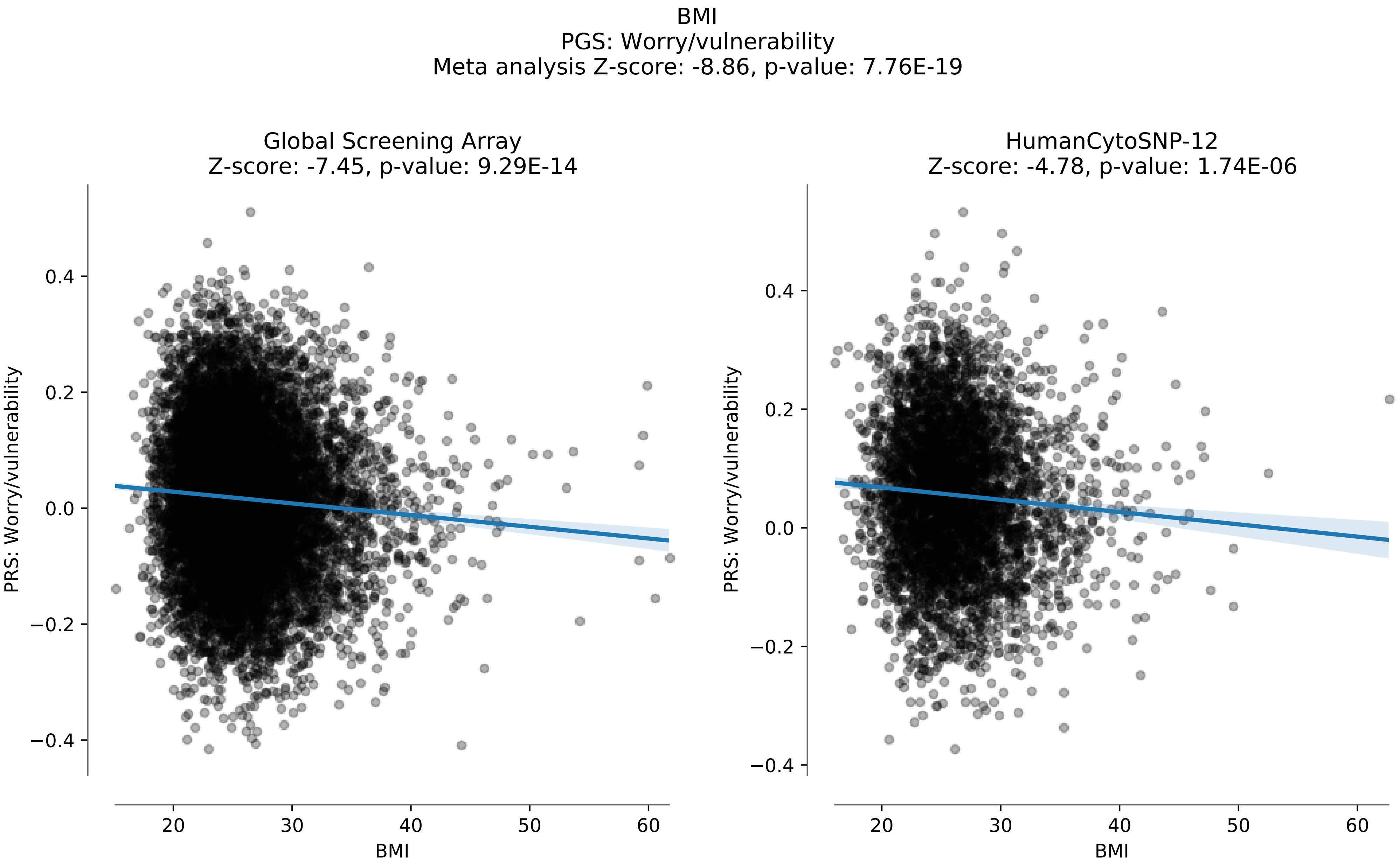

BMI
PGS: Worry/vulnerability
Meta analysis Z-score: -8.86, p-value: 7.76E-19
Global Screening Array
Z-score: -7.45, p-value: 9.29E-14
HumanCytoSNP-12
Z-score: -4.78, p-value: 1.74E-06
PRS: Worry/vulnerability
BMI
0.4
0.2
0.0
−0.2
−0.4
20
30
40
50
60

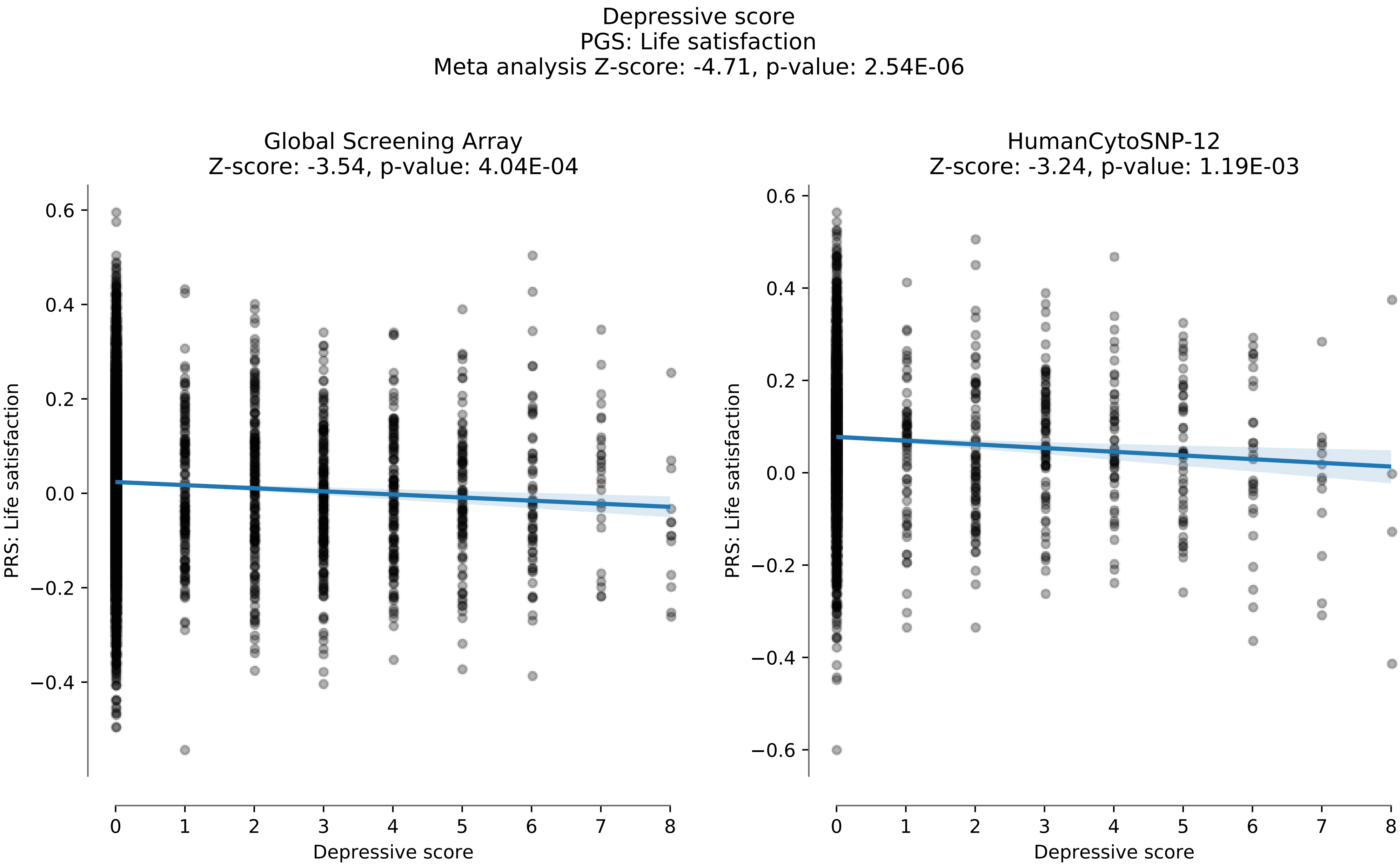
Depressive score
PGS: Life satisfaction
Meta analysis Z-score: -4.71, p-value: 2.54E-06
Global Screening Array
Z-score: -3.54, p-value: 4.04E-04
HumanCytoSNP-12
Z-score: -3.24, p-value: 1.19E-03
PRS: Life satisfaction
Depressive score
0.6
0.4
0.2
0.0
−0.2
−0.4
−0.6
0
1
2
3
4
5
6
7
8

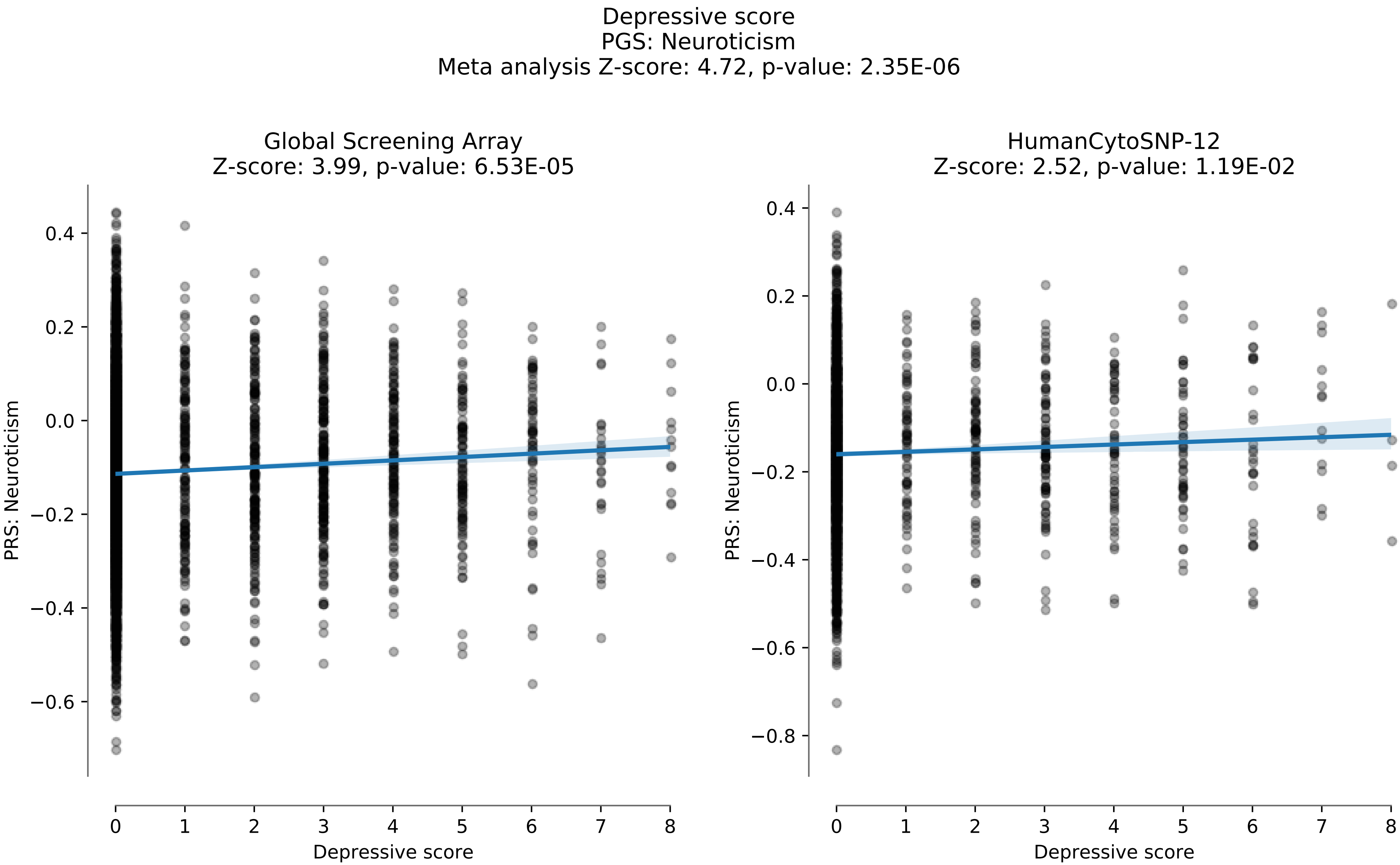
Depressive score
PGS: Neuroticism
Meta analysis Z-score: 4.72, p-value: 2.35E-06
Global Screening Array
Z-score: 3.99, p-value: 6.53E-05
HumanCytoSNP-12
Z-score: 2.52, p-value: 1.19E-02
PRS: Neuroticism
Depressive score
0.4
0.2
0.0
−0.2
−0.4
−0.6
−0.8
0
1
2
3
4
5
6
7
8

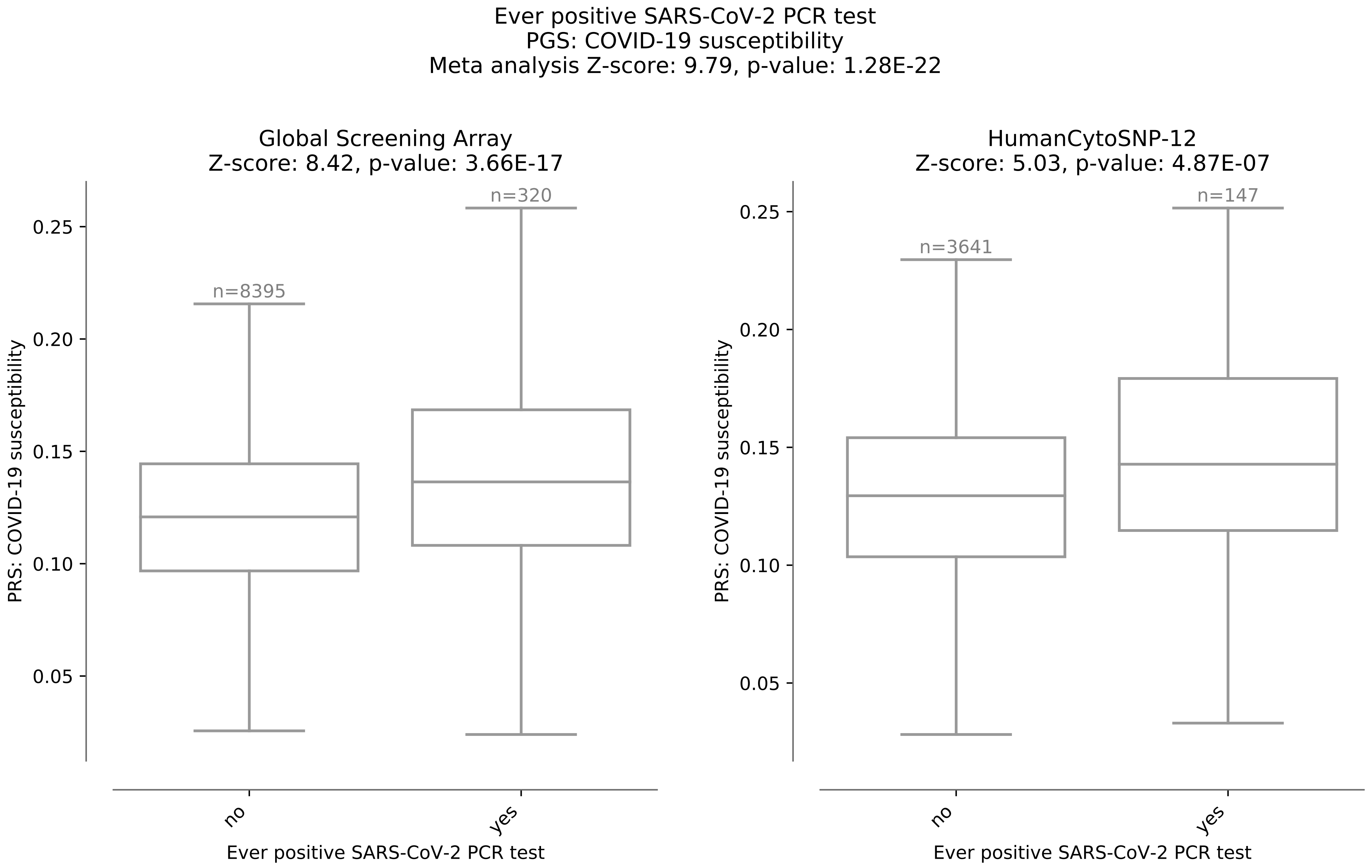

Ever positive SARS-CoV-2 PCR test
PGS: COVID-19 susceptibility
Meta analysis Z-score: 9.79, p-value: 1.28E-22
Global Screening Array
Z-score: 8.42, p-value: 3.66E-17
HumanCytoSNP-12
Z-score: 5.03, p-value: 4.87E-07
n=8395
n=320
n=3641
n=147
PRS: COVID-19 susceptibility
0.25
0.20
0.15
0.10
0.05
no
yes
Ever positive SARS-CoV-2 PCR test

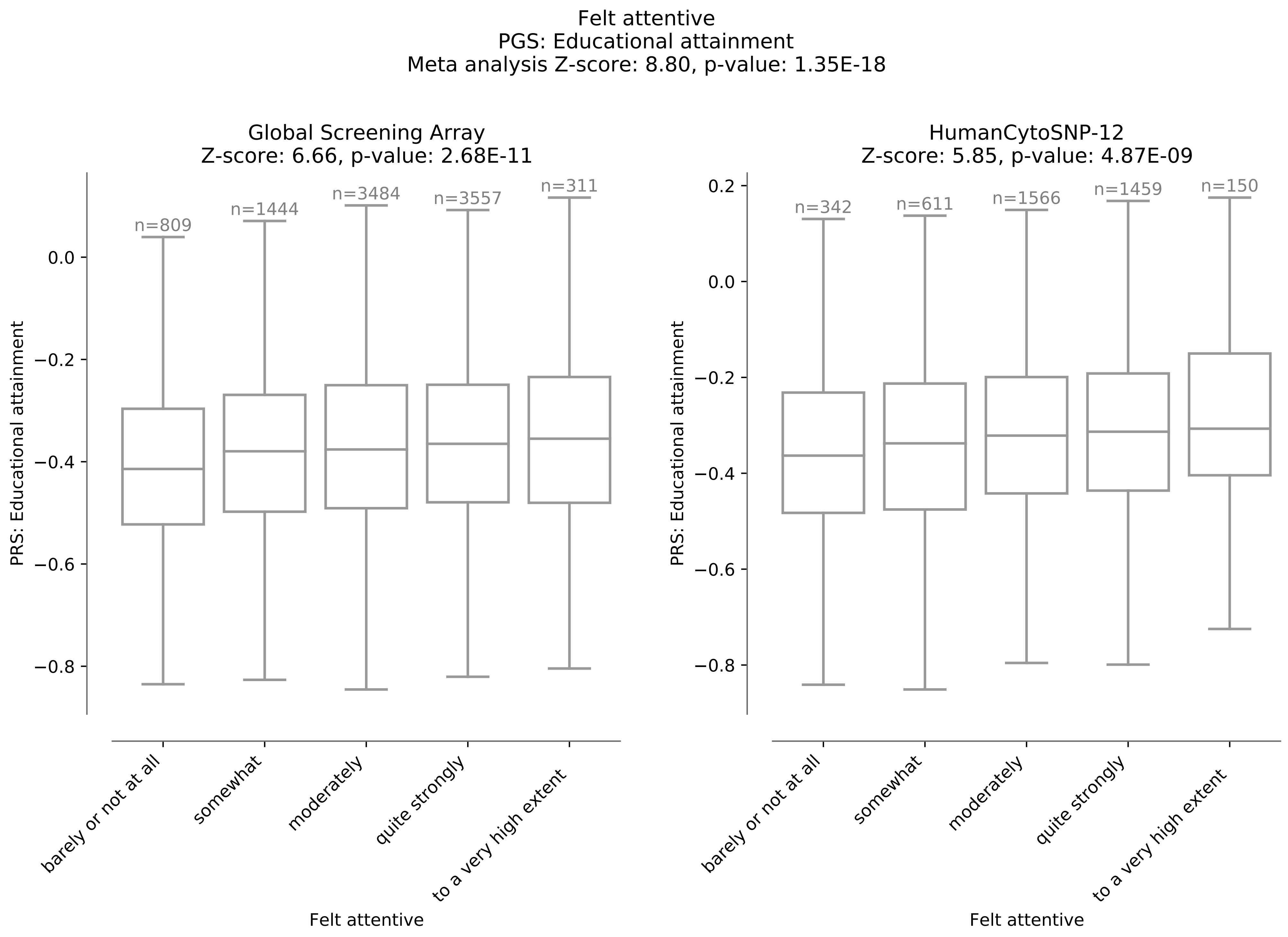

Felt attentive
PGS: Educational attainment
Meta analysis Z-score: 8.80, p-value: 1.35E-18
Global Screening Array
Z-score: 6.66, p-value: 2.68E-11
HumanCytoSNP-12
Z-score: 5.85, p-value: 4.87E-09
PRS: Educational attainment
n=809
n=1444
n=3484
n=3557
n=311
n=342
n=611
n=1566
n=1459
n=150
0.2
0.0
−0.2
−0.4
−0.6
−0.8
barely or not at all
somewhat
moderately
quite strongly
to a very high extent
Felt attentive

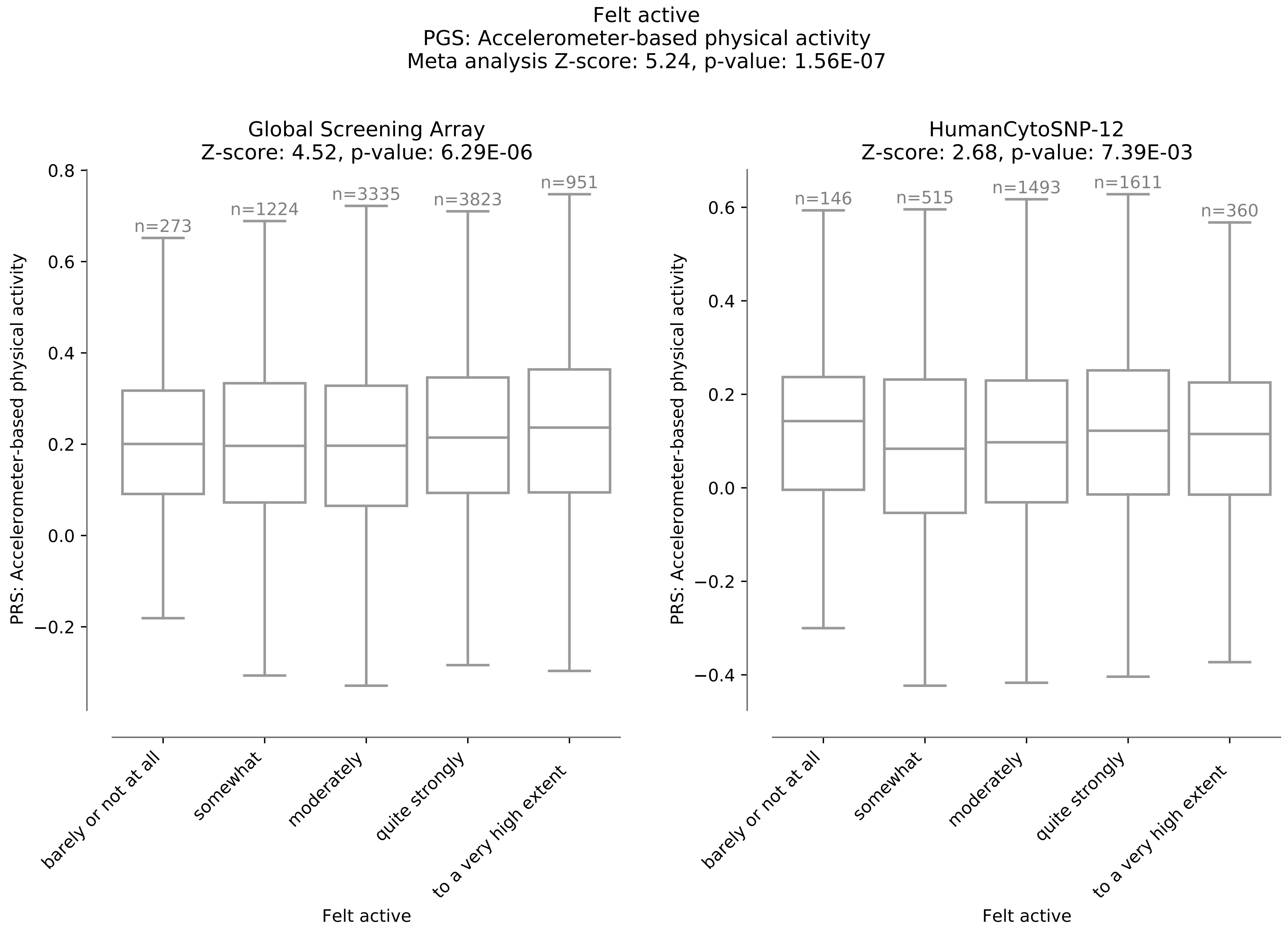

Felt active
PGS: Accelerometer-based physical activity
Meta analysis Z-score: 5.24, p-value: 1.56E-07
Global Screening Array
Z-score: 4.52, p-value: 6.29E-06
HumanCytoSNP-12
Z-score: 2.68, p-value: 7.39E-03
PRS: Accelerometer-based physical activity
n=273
n=1224
n=3335
n=3823
n=951
n=146
n=515
n=1493
n=1611
n=360
barely or not at all
somewhat
moderately
quite strongly
to a very high extent
Felt active

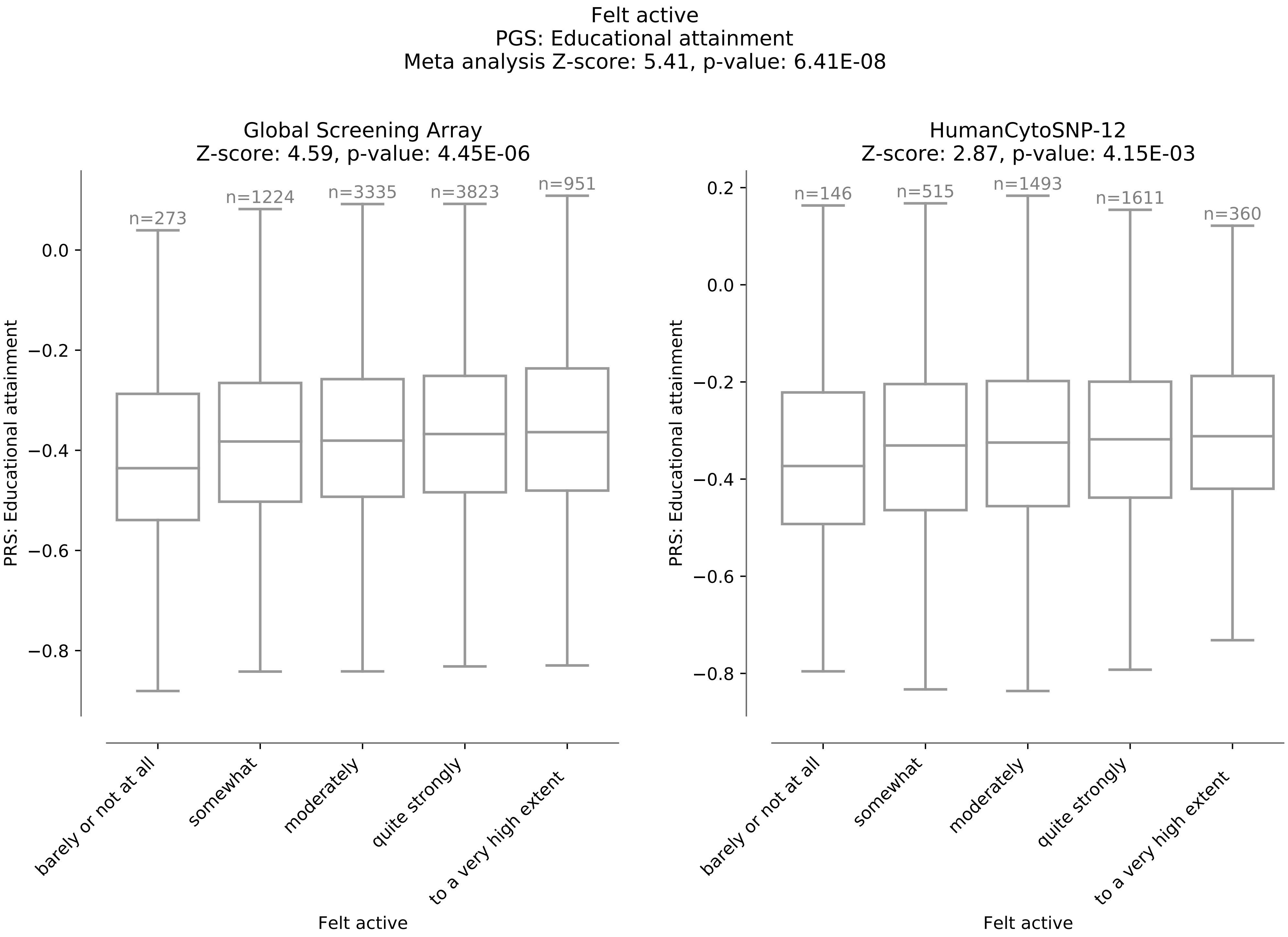
Felt active
PGS: Educational attainment
Meta analysis Z-score: 5.41, p-value: 6.41E-08
Global Screening Array
Z-score: 4.59, p-value: 4.45E-06
HumanCytoSNP-12
Z-score: 2.87, p-value: 4.15E-03
PRS: Educational attainment
n=273
n=1224
n=3335
n=3823
n=951
n=146
n=515
n=1493
n=1611
n=360
0.2
0.0
−0.2
−0.4
−0.6
−0.8
barely or not at all
somewhat
moderately
quite strongly
to a very high extent
Felt active

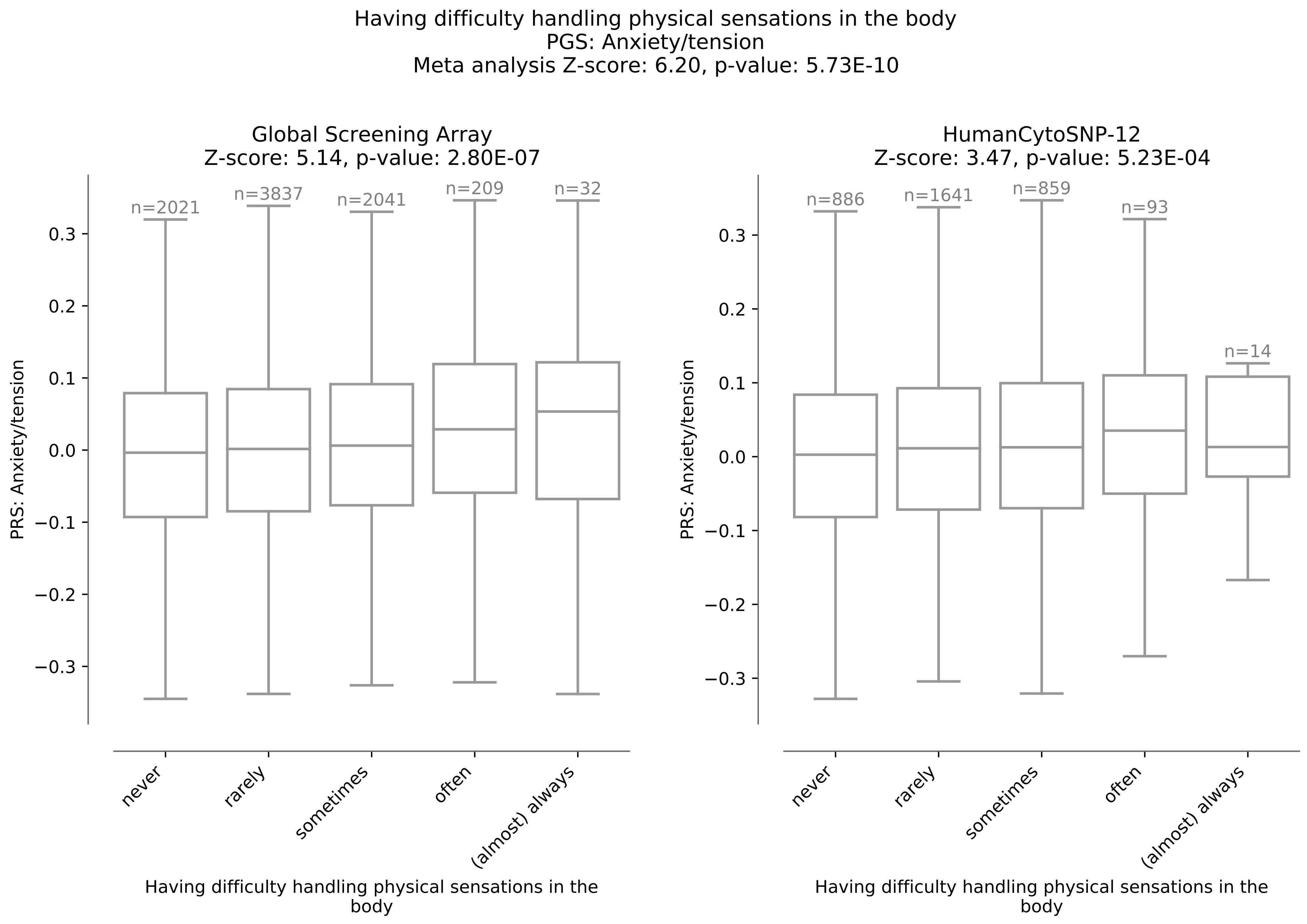
Having difficulty handling physical sensations in the body
PGS: Anxiety/tension
Meta analysis Z-score: 6.20, p-value: 5.73E-10
Global Screening Array
Z-score: 5.14, p-value: 2.80E-07
n=2021
n=3837
n=2041
n=209
n=32
PRS: Anxiety/tension
0.3
0.2
0.1
0.0
−0.1
−0.2
−0.3
never
rarely
sometimes
often
(almost) always
Having difficulty handling physical sensations in the body
HumanCytoSNP-12
Z-score: 3.47, p-value: 5.23E-04
n=886
n=1641
n=859
n=93
n=14
PRS: Anxiety/tension
0.3
0.2
0.1
0.0
−0.1
−0.2
−0.3
never
rarely
sometimes
often
(almost) always
Having difficulty handling physical sensations in the body

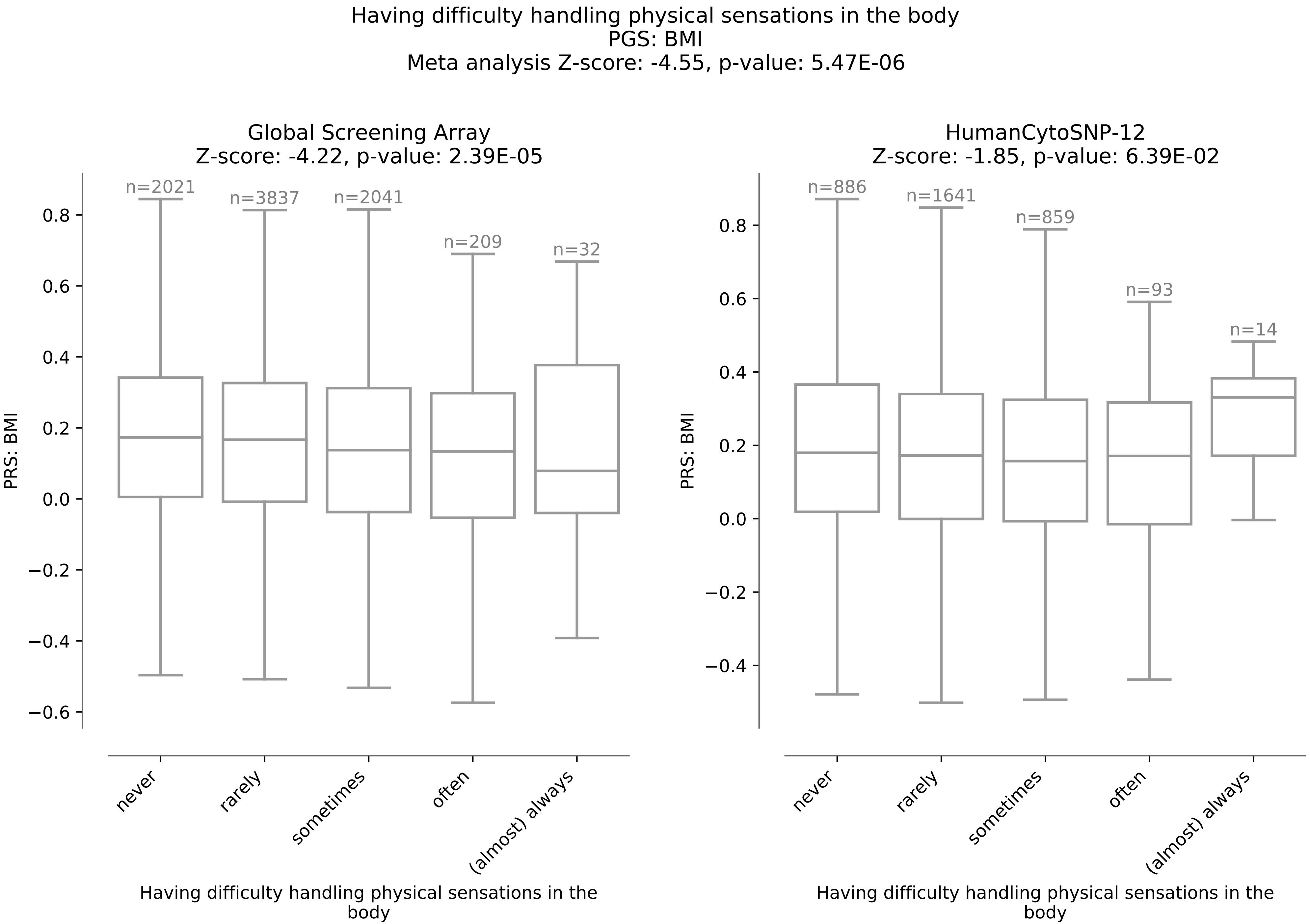
Having difficulty handling physical sensations in the body
PGS: BMI
Meta analysis Z-score: -4.55, p-value: 5.47E-06
Global Screening Array
Z-score: -4.22, p-value: 2.39E-05
n=2021
n=3837
n=2041
n=209
n=32
PRS: BMI
0.8
0.6
0.4
0.2
0.0
−0.2
−0.4
−0.6
never
rarely
sometimes
often
(almost) always
Having difficulty handling physical sensations in the body
HumanCytoSNP-12
Z-score: -1.85, p-value: 6.39E-02
n=886
n=1641
n=859
n=93
n=14
PRS: BMI
0.8
0.6
0.4
0.2
0.0
−0.2
−0.4
never
rarely
sometimes
often
(almost) always
Having difficulty handling physical sensations in the body

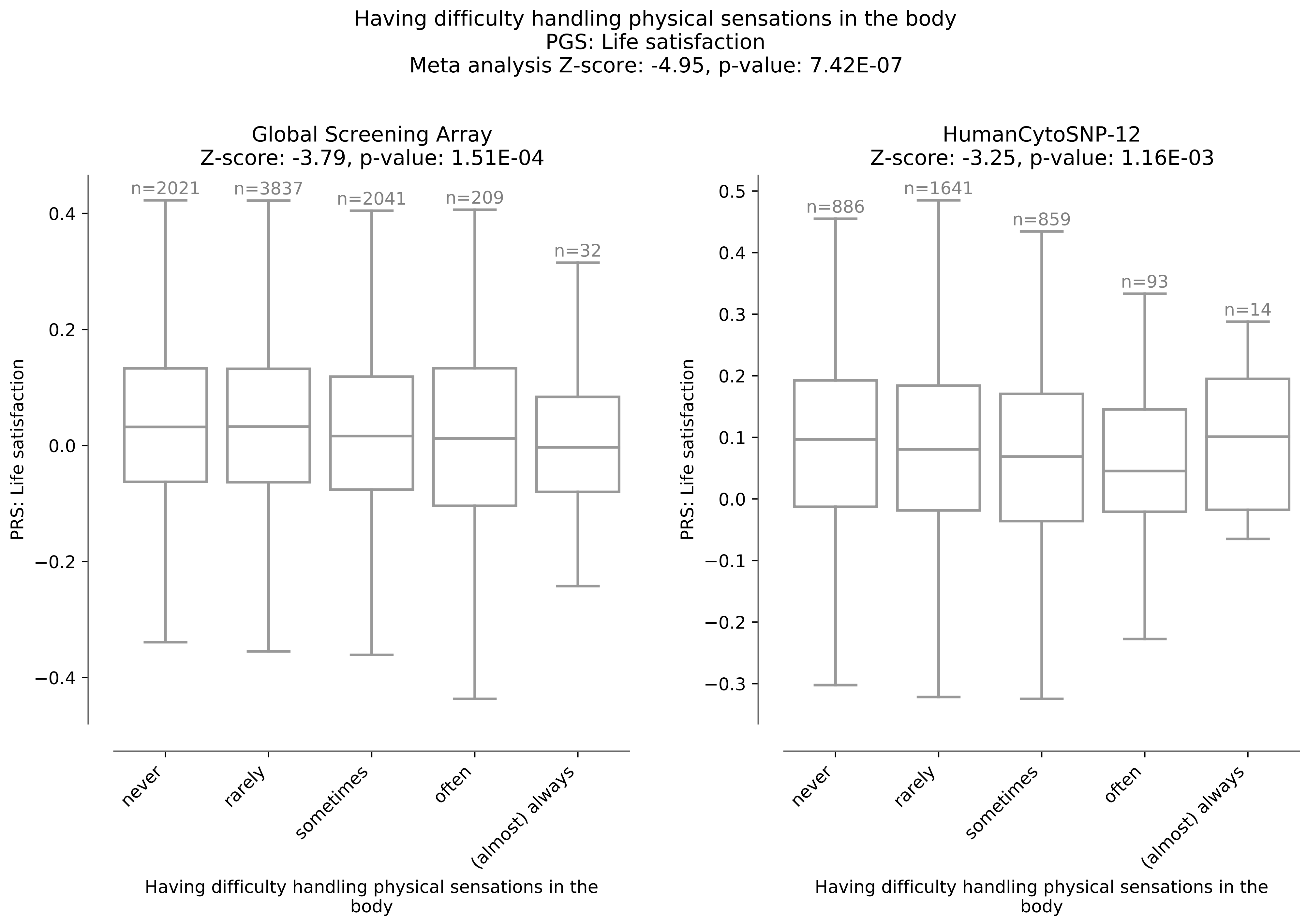
Having difficulty handling physical sensations in the body
PGS: Life satisfaction
Meta analysis Z-score: -4.95, p-value: 7.42E-07
Global Screening Array
Z-score: -3.79, p-value: 1.51E-04
n=2021
n=3837
n=2041
n=209
n=32
PRS: Life satisfaction
0.4
0.2
0.0
−0.2
−0.4
never
rarely
sometimes
often
(almost) always
Having difficulty handling physical sensations in the body
HumanCytoSNP-12
Z-score: -3.25, p-value: 1.16E-03
n=886
n=1641
n=859
n=93
n=14
PRS: Life satisfaction
0.5
0.4
0.3
0.2
0.1
0.0
−0.1
−0.2
−0.3
never
rarely
sometimes
often
(almost) always
Having difficulty handling physical sensations in the body

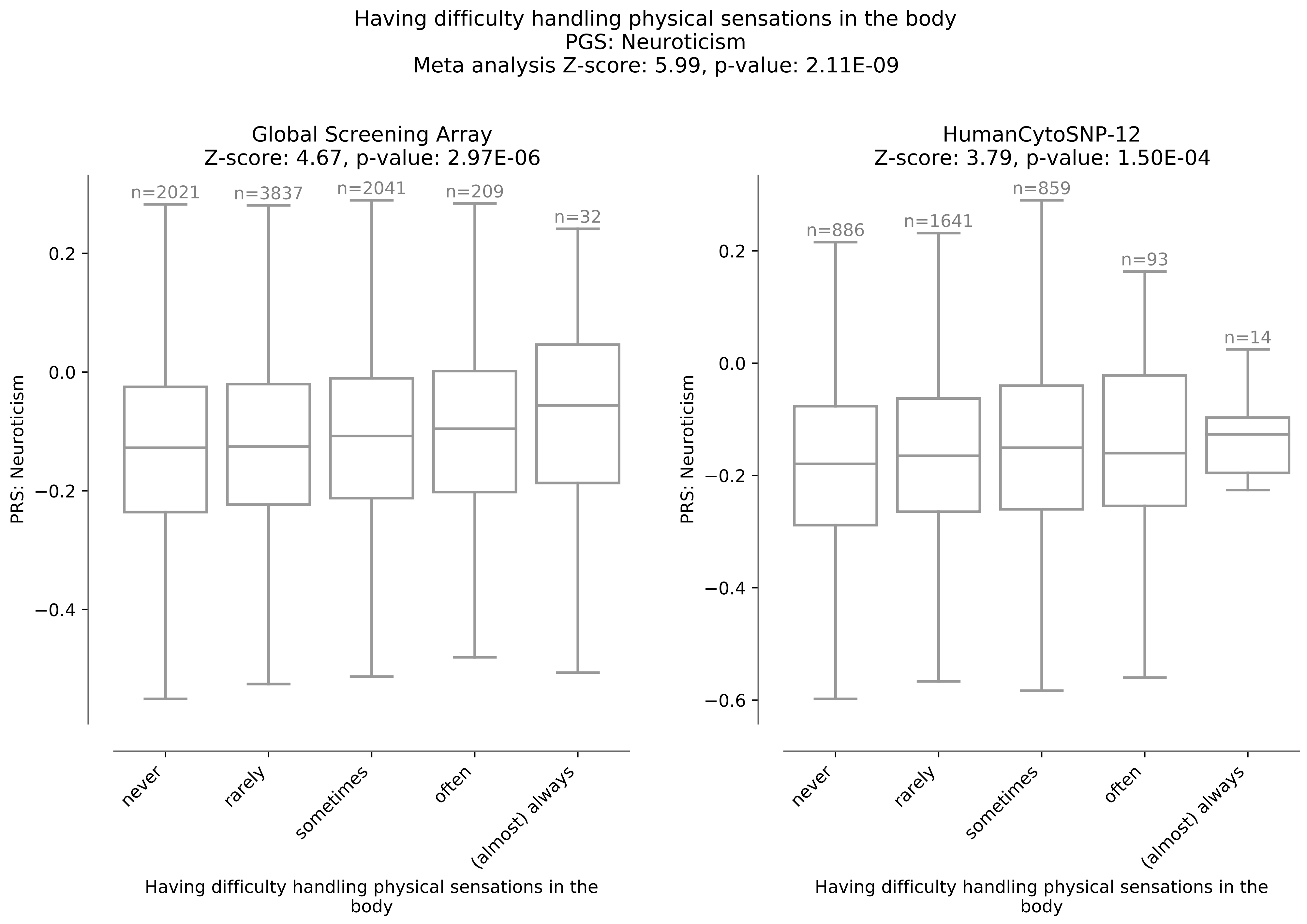

Having difficulty handling physical sensations in the body
PGS: Neuroticism
Meta analysis Z-score: 5.99, p-value: 2.11E-09
Global Screening Array
Z-score: 4.67, p-value: 2.97E-06
n=2021
n=3837
n=2041
n=209
n=32
PRS: Neuroticism
0.2
0.0
−0.2
−0.4
never
rarely
sometimes
often
(almost) always
Having difficulty handling physical sensations in the body
HumanCytoSNP-12
Z-score: 3.79, p-value: 1.50E-04
n=886
n=1641
n=859
n=93
n=14
PRS: Neuroticism
0.2
0.0
−0.2
−0.4
−0.6
never
rarely
sometimes
often
(almost) always
Having difficulty handling physical sensations in the body

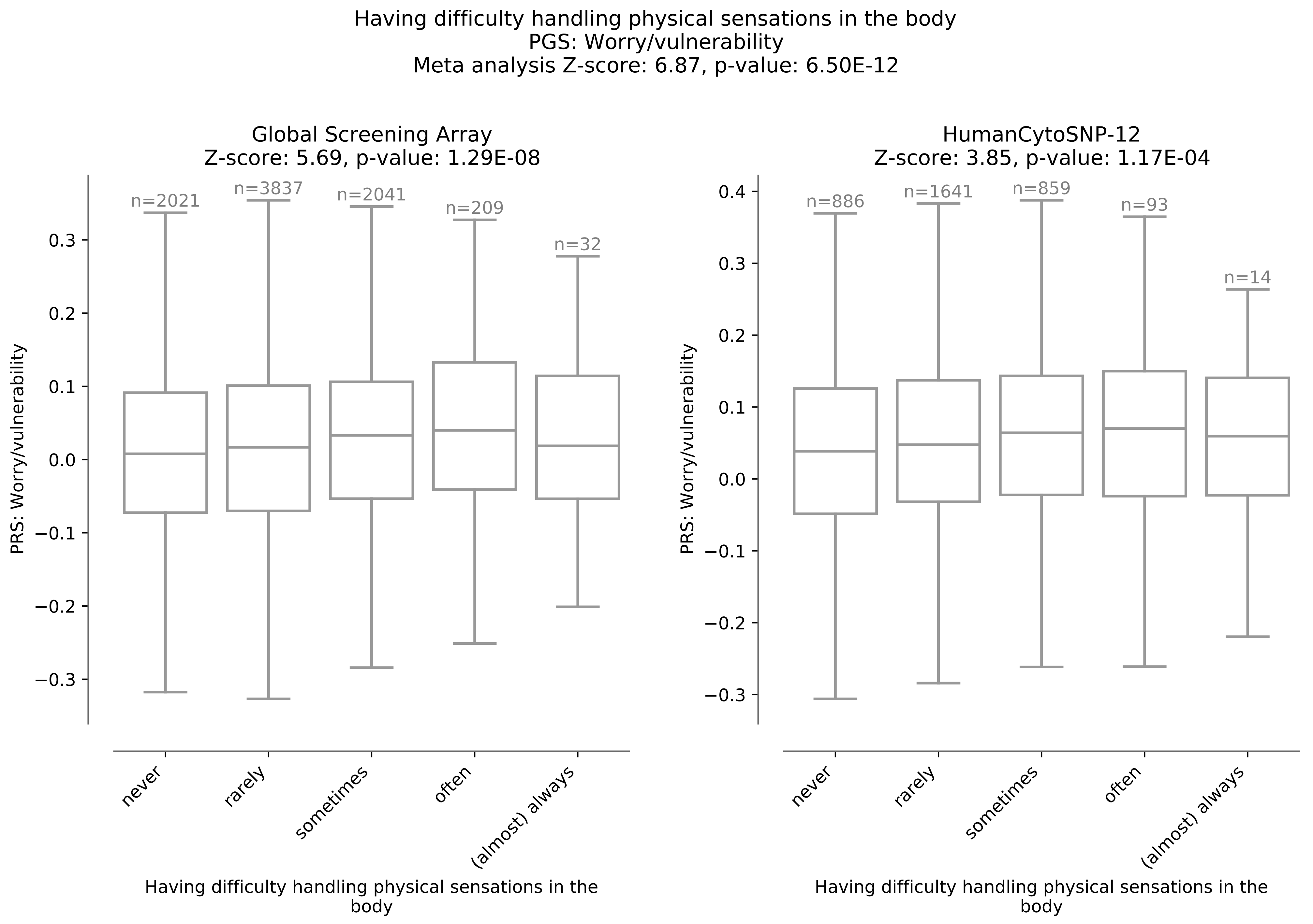
Having difficulty handling physical sensations in the body
PGS: Worry/vulnerability
Meta analysis Z-score: 6.87, p-value: 6.50E-12
Global Screening Array
Z-score: 5.69, p-value: 1.29E-08
n=2021
n=3837
n=2041
n=209
n=32
PRS: Worry/vulnerability
never
rarely
sometimes
often
(almost) always
Having difficulty handling physical sensations in the body
HumanCytoSNP-12
Z-score: 3.85, p-value: 1.17E-04
n=886
n=1641
n=859
n=93
n=14
PRS: Worry/vulnerability
never
rarely
sometimes
often
(almost) always
Having difficulty handling physical sensations in the body

Worry when feeling some sensation in the body
PGS: Anxiety/tension
Meta analysis Z-score: 6.04, p-value: 1.58E-09

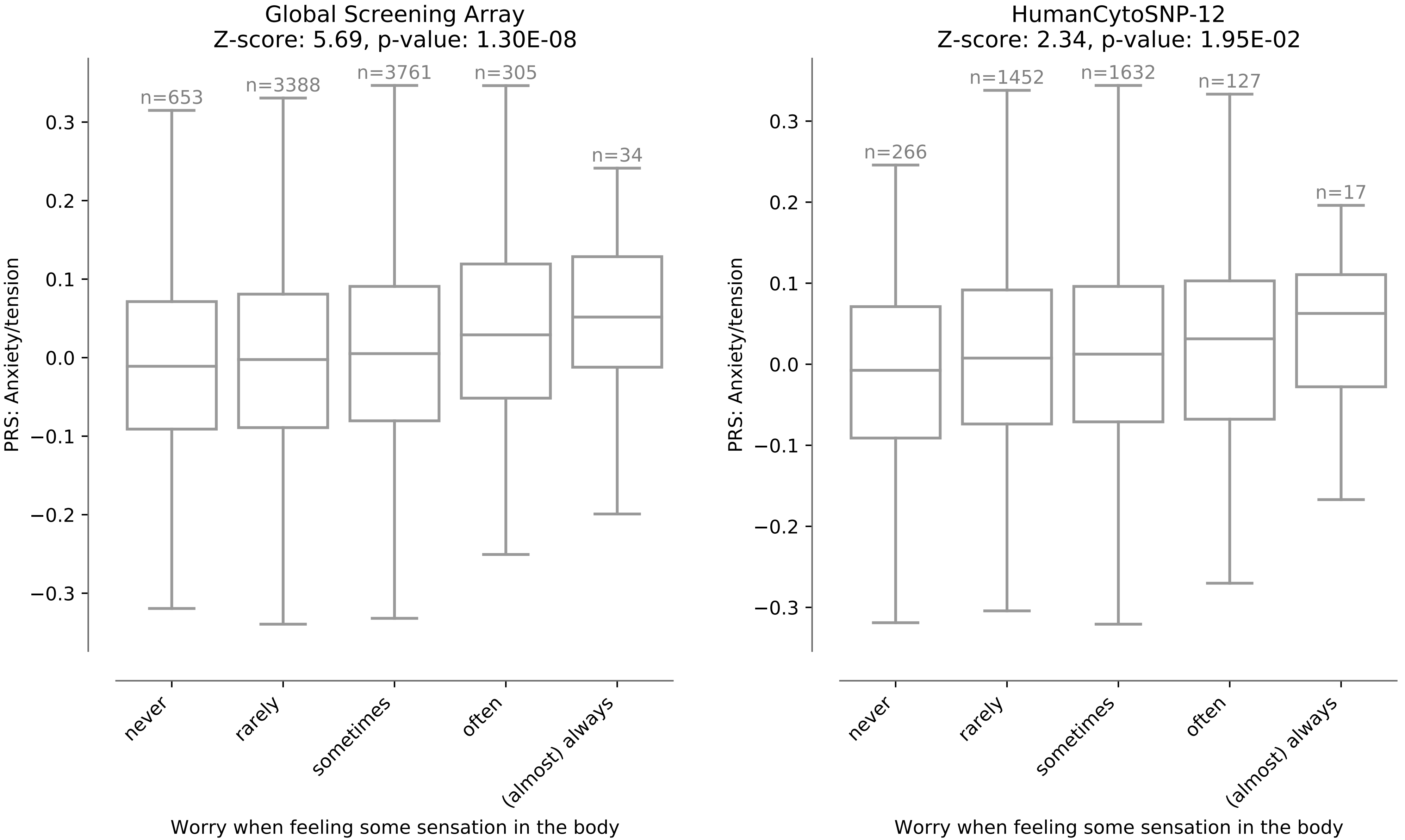

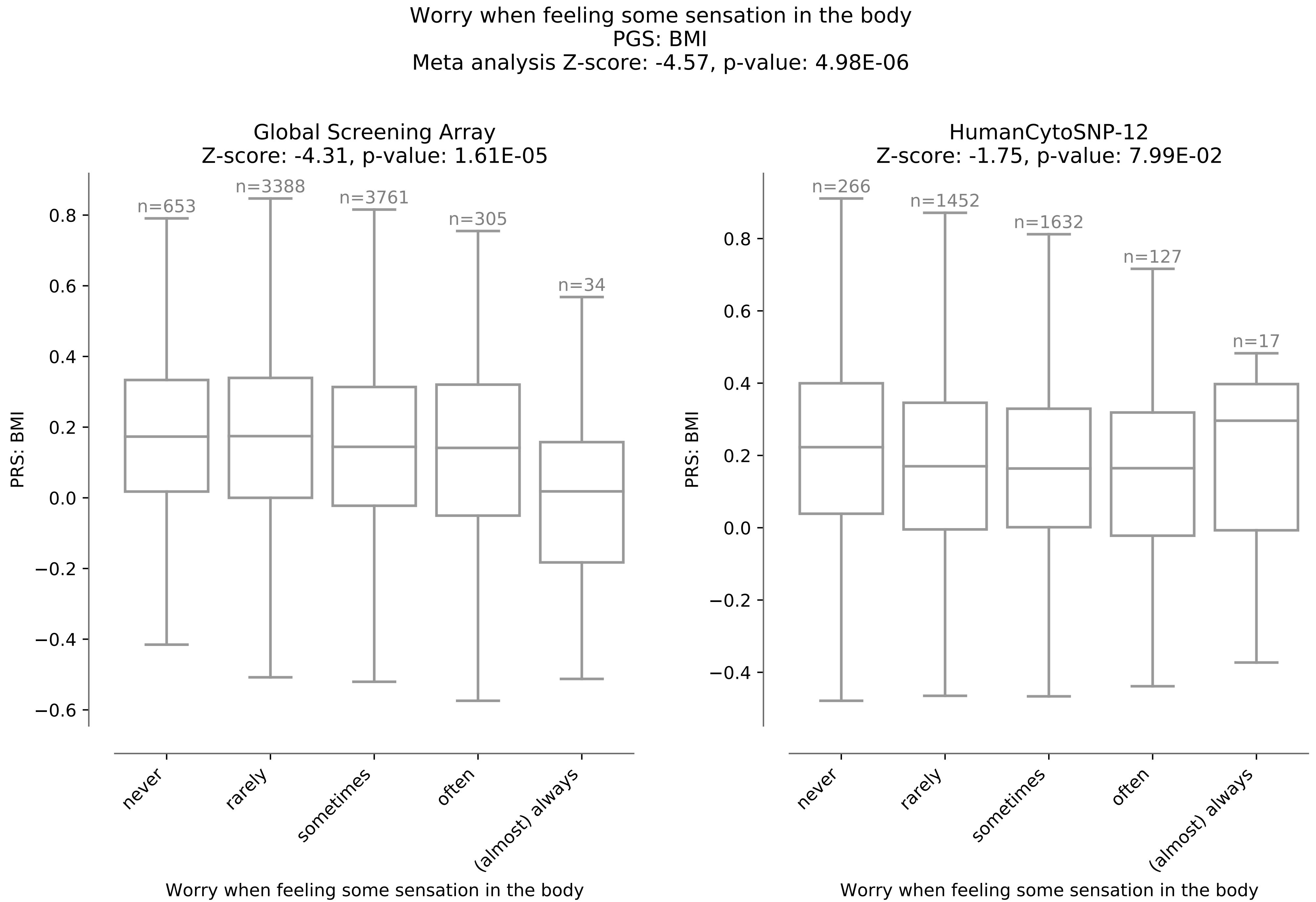
Worry when feeling some sensation in the body
PGS: BMI
Meta analysis Z-score: -4.57, p-value: 4.98E-06
Global Screening Array
Z-score: -4.31, p-value: 1.61E-05
HumanCytoSNP-12
Z-score: -1.75, p-value: 7.99E-02
PRS: BMI
n=653
n=3388
n=3761
n=305
n=34
n=266
n=1452
n=1632
n=127
n=17
never
rarely
sometimes
often
(almost) always
Worry when feeling some sensation in the body

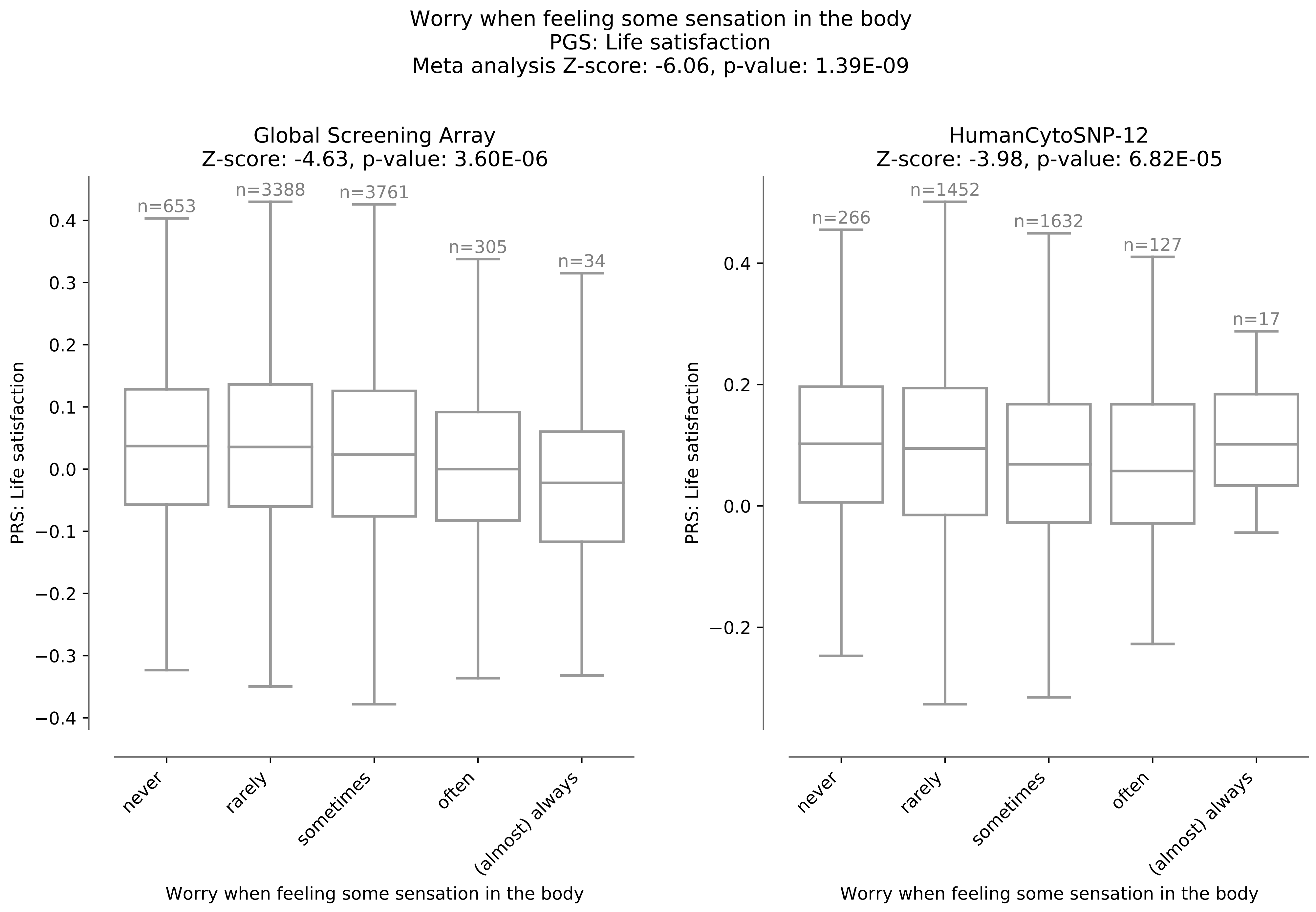

Worry when feeling some sensation in the body
PGS: Life satisfaction
Meta analysis Z-score: -6.06, p-value: 1.39E-09
Global Screening Array
Z-score: -4.63, p-value: 3.60E-06
HumanCytoSNP-12
Z-score: -3.98, p-value: 6.82E-05
PRS: Life satisfaction
n=653
n=3388
n=3761
n=305
n=34
n=266
n=1452
n=1632
n=127
n=17
never
rarely
sometimes
often
(almost) always
Worry when feeling some sensation in the body

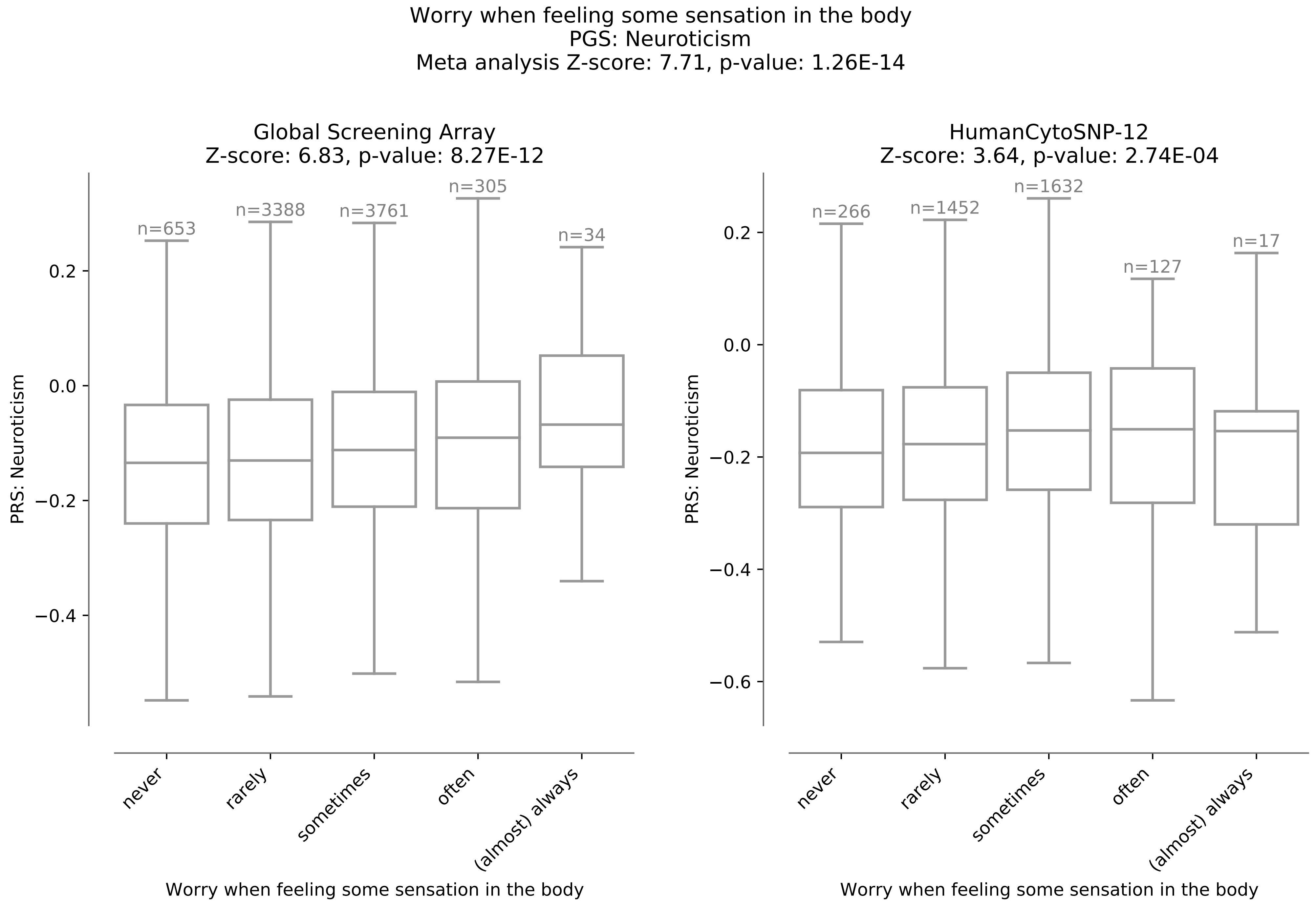
Worry when feeling some sensation in the body
PGS: Neuroticism
Meta analysis Z-score: 7.71, p-value: 1.26E-14
Global Screening Array
Z-score: 6.83, p-value: 8.27E-12
HumanCytoSNP-12
Z-score: 3.64, p-value: 2.74E-04
PRS: Neuroticism
n=653
n=3388
n=3761
n=305
n=34
n=266
n=1452
n=1632
n=127
n=17
0.2
0.0
−0.2
−0.4
−0.6
never
rarely
sometimes
often
(almost) always
Worry when feeling some sensation in the body

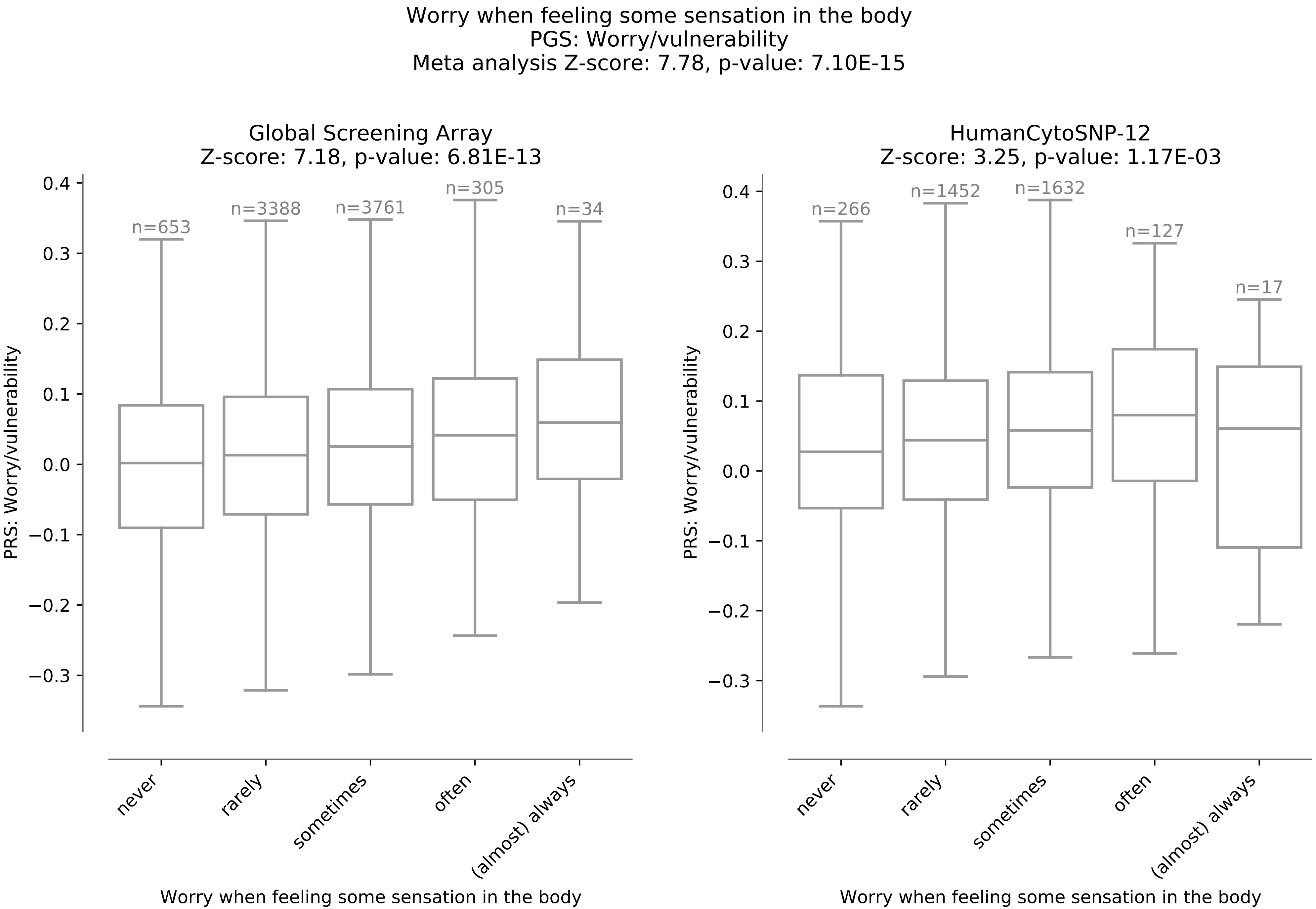
Worry when feeling some sensation in the body
PGS: Worry/vulnerability
Meta analysis Z-score: 7.78, p-value: 7.10E-15
Global Screening Array
Z-score: 7.18, p-value: 6.81E-13
HumanCytoSNP-12
Z-score: 3.25, p-value: 1.17E-03
PRS: Worry/vulnerability
n=653
n=3388
n=3761
n=305
n=34
n=266
n=1452
n=1632
n=127
n=17
never
rarely
sometimes
often
(almost) always
Worry when feeling some sensation in the body

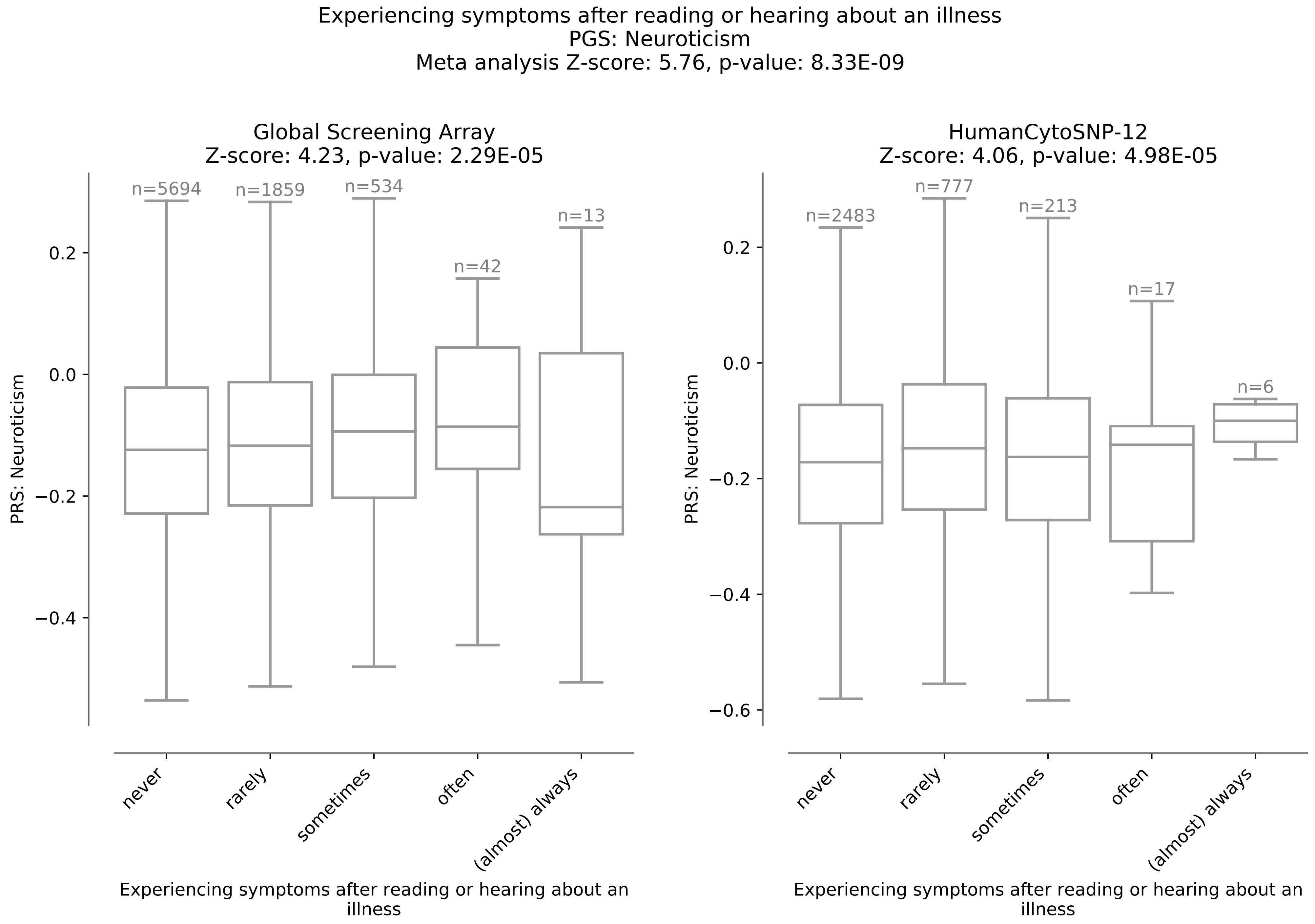
Experiencing symptoms after reading or hearing about an illness
PGS: Neuroticism
Meta analysis Z-score: 5.76, p-value: 8.33E-09
Global Screening Array
Z-score: 4.23, p-value: 2.29E-05
HumanCytoSNP-12
Z-score: 4.06, p-value: 4.98E-05
PRS: Neuroticism
0.2
0.0
−0.2
−0.4
−0.6
n=5694
n=1859
n=534
n=42
n=13
n=2483
n=777
n=213
n=17
n=6
never
rarely
sometimes
often
(almost) always
Experiencing symptoms after reading or hearing about an illness

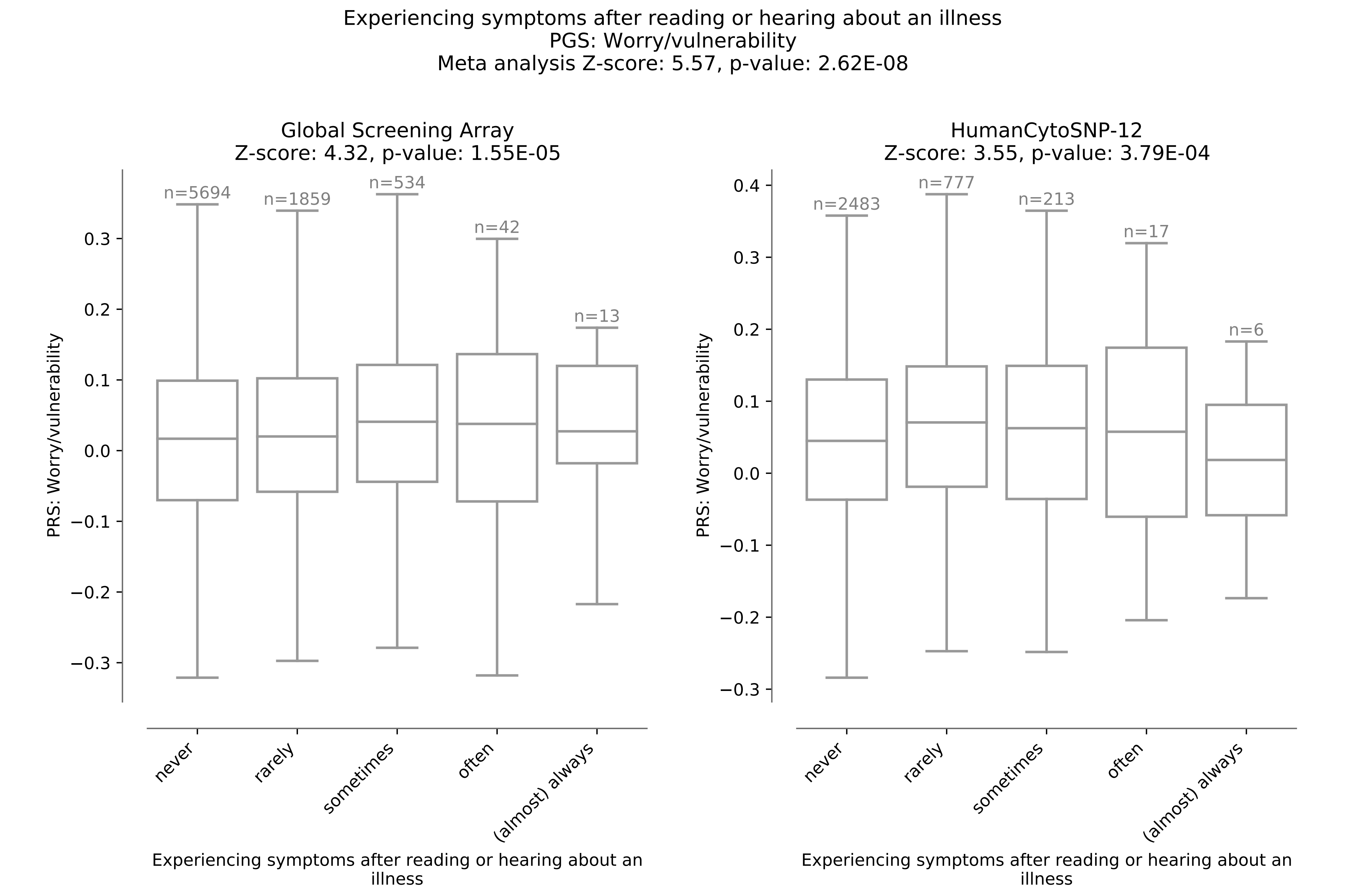

Experiencing symptoms after reading or hearing about an illness
PGS: Worry/vulnerability
Meta analysis Z-score: 5.57, p-value: 2.62E-08
Global Screening Array
Z-score: 4.32, p-value: 1.55E-05
HumanCytoSNP-12
Z-score: 3.55, p-value: 3.79E-04
PRS: Worry/vulnerability
n=5694
n=1859
n=534
n=42
n=13
n=2483
n=777
n=213
n=17
n=6
never
rarely
sometimes
often
(almost) always
Experiencing symptoms after reading or hearing about an illness

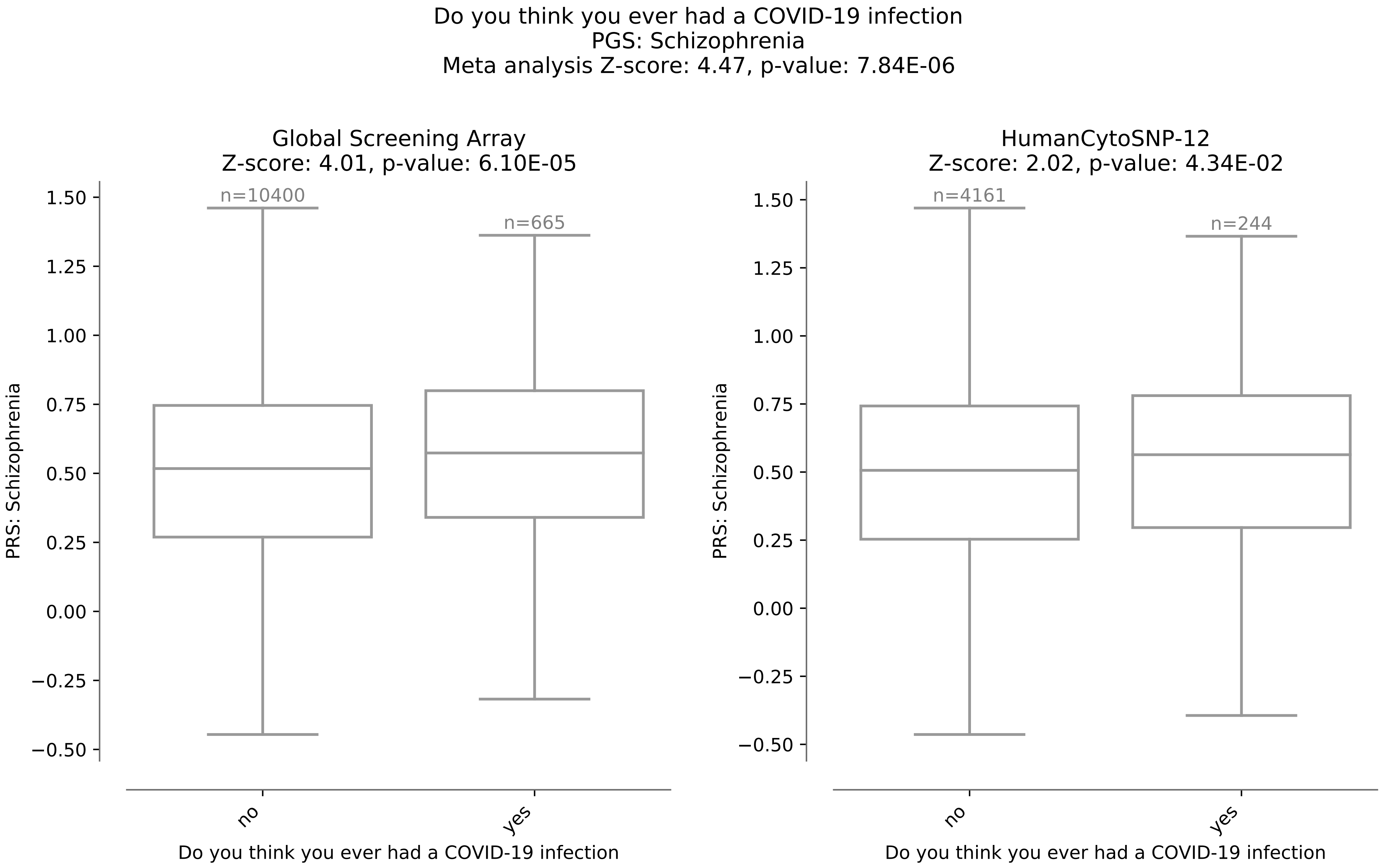
Do you think you ever had a COVID-19 infection
PGS: Schizophrenia
Meta analysis Z-score: 4.47, p-value: 7.84E-06
Global Screening Array
Z-score: 4.01, p-value: 6.10E-05
HumanCytoSNP-12
Z-score: 2.02, p-value: 4.34E-02
n=10400
n=665
n=4161
n=244
PRS: Schizophrenia
1.50
1.25
1.00
0.75
0.50
0.25
0.00
−0.25
−0.50
no
yes
Do you think you ever had a COVID-19 infection

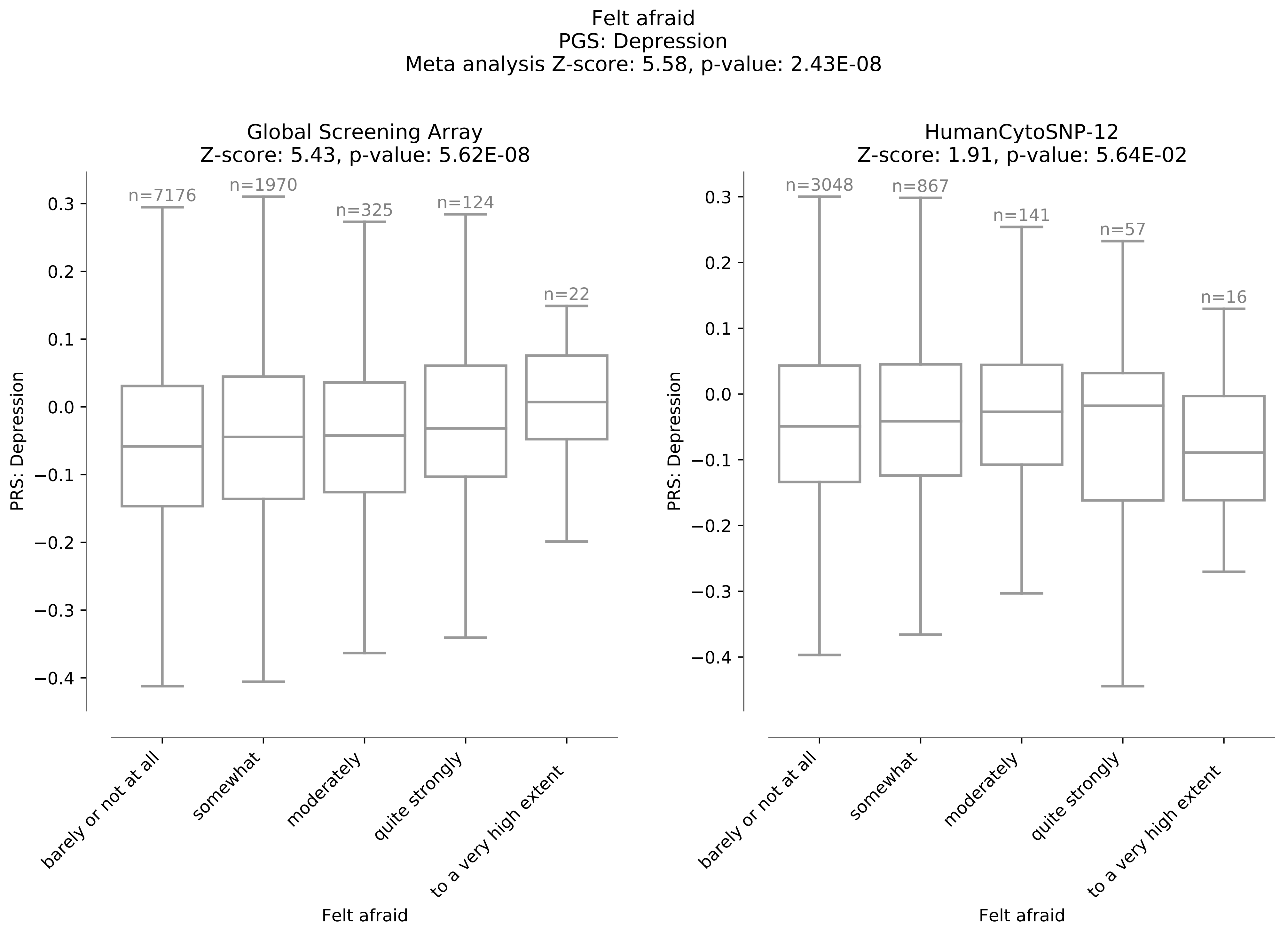

Felt afraid
PGS: Depression
Meta analysis Z-score: 5.58, p-value: 2.43E-08
Global Screening Array
Z-score: 5.43, p-value: 5.62E-08
n=7176
n=1970
n=325
n=124
n=22
PRS: Depression
0.3
0.2
0.1
0.0
−0.1
−0.2
−0.3
−0.4
barely or not at all
somewhat
moderately
quite strongly
to a very high extent
Felt afraid
HumanCytoSNP-12
Z-score: 1.91, p-value: 5.64E-02
n=3048
n=867
n=141
n=57
n=16
PRS: Depression
0.3
0.2
0.1
0.0
−0.1
−0.2
−0.3
−0.4
barely or not at all
somewhat
moderately
quite strongly
to a very high extent
Felt afraid

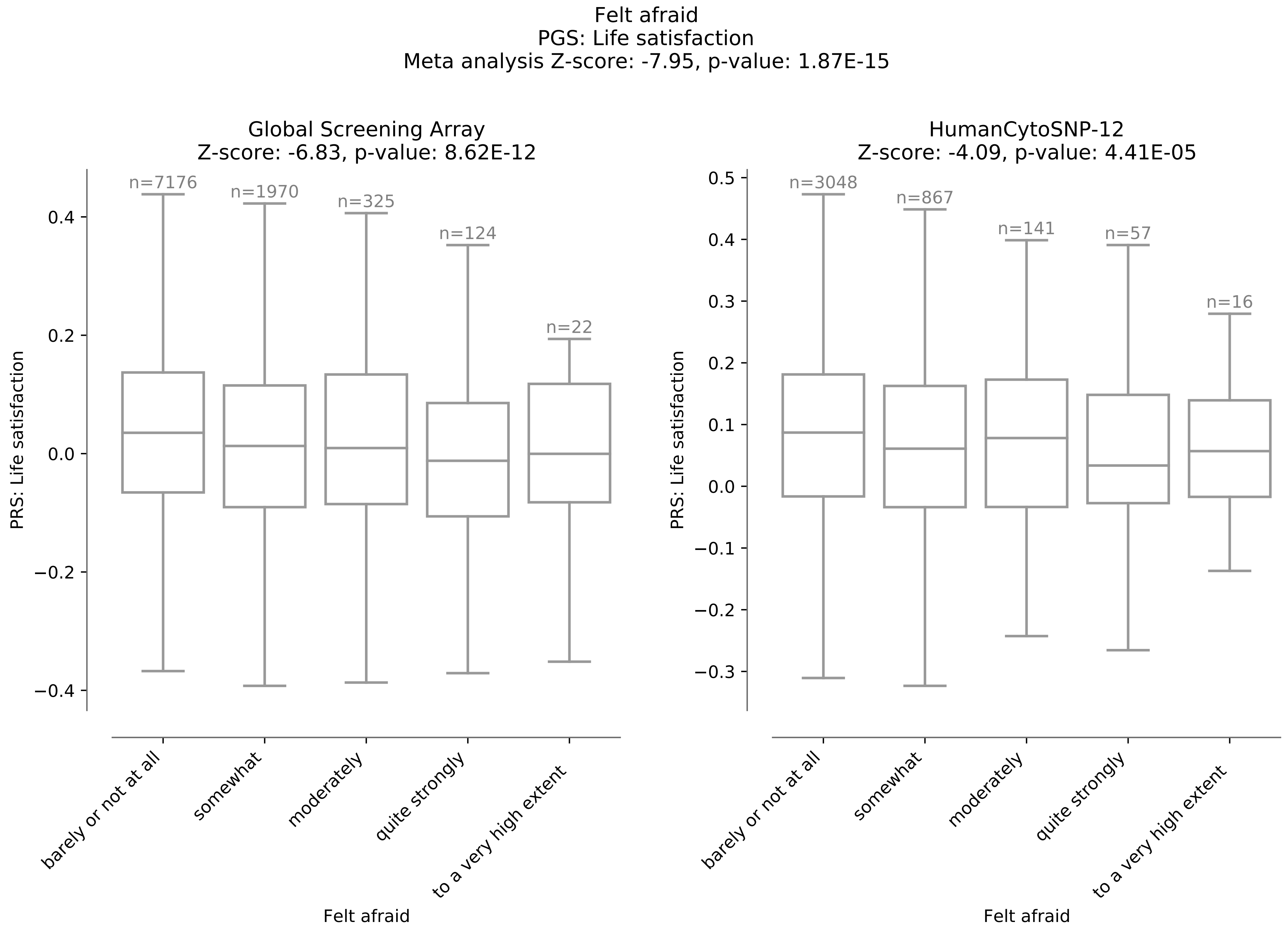
Felt afraid
PGS: Life satisfaction
Meta analysis Z-score: -7.95, p-value: 1.87E-15
Global Screening Array
Z-score: -6.83, p-value: 8.62E-12
HumanCytoSNP-12
Z-score: -4.09, p-value: 4.41E-05
n=7176
n=1970
n=325
n=124
n=22
n=3048
n=867
n=141
n=57
n=16
PRS: Life satisfaction
barely or not at all
somewhat
moderately
quite strongly
to a very high extent
Felt afraid

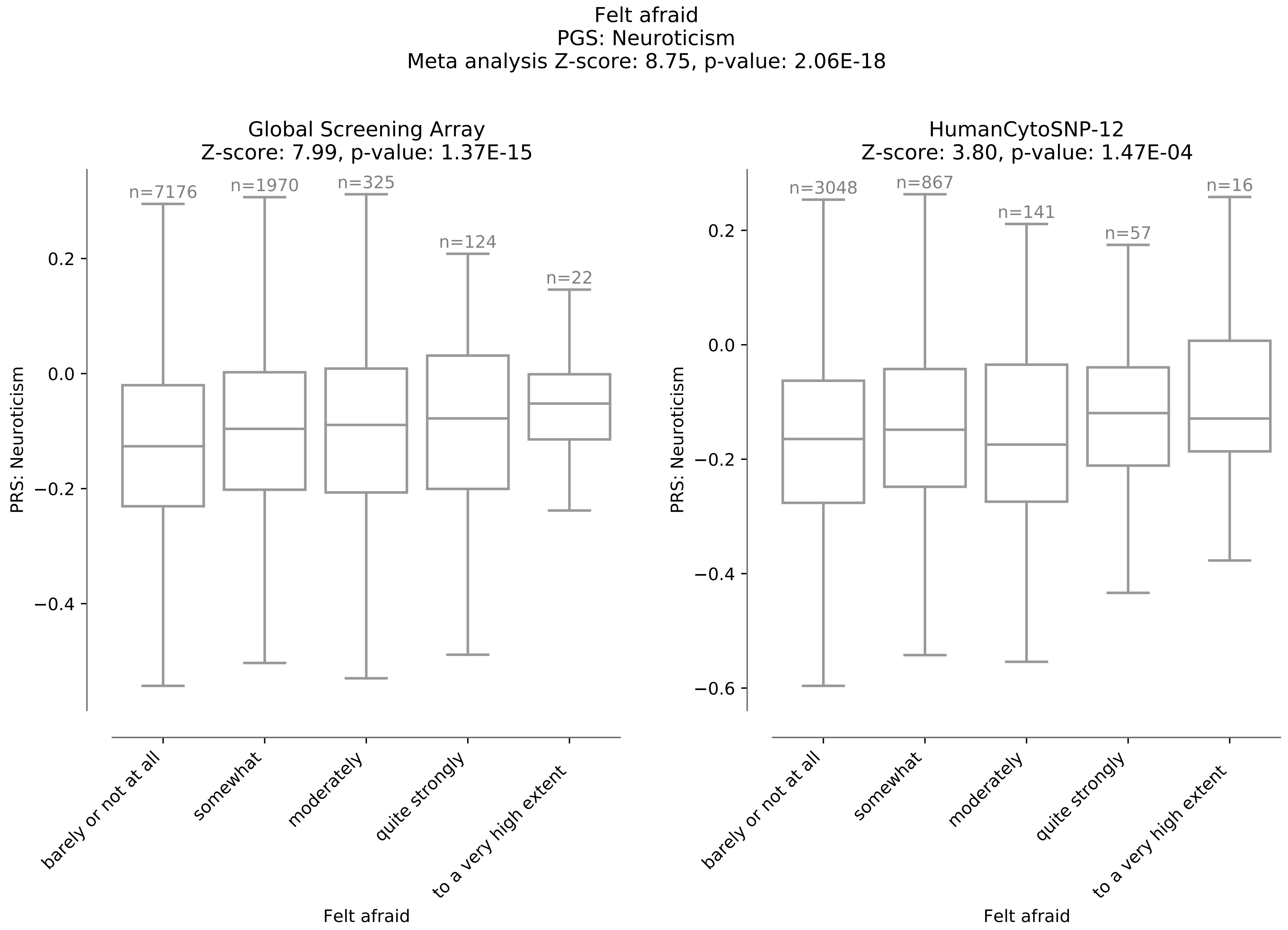
Felt afraid
PGS: Neuroticism
Meta analysis Z-score: 8.75, p-value: 2.06E-18
Global Screening Array
Z-score: 7.99, p-value: 1.37E-15
n=7176
n=1970
n=325
n=124
n=22
0.2
0.0
−0.2
−0.4
PRS: Neuroticism
barely or not at all
somewhat
moderately
quite strongly
to a very high extent
Felt afraid
HumanCytoSNP-12
Z-score: 3.80, p-value: 1.47E-04
n=3048
n=867
n=141
n=57
n=16
0.2
0.0
−0.2
−0.4
−0.6
PRS: Neuroticism
barely or not at all
somewhat
moderately
quite strongly
to a very high extent
Felt afraid

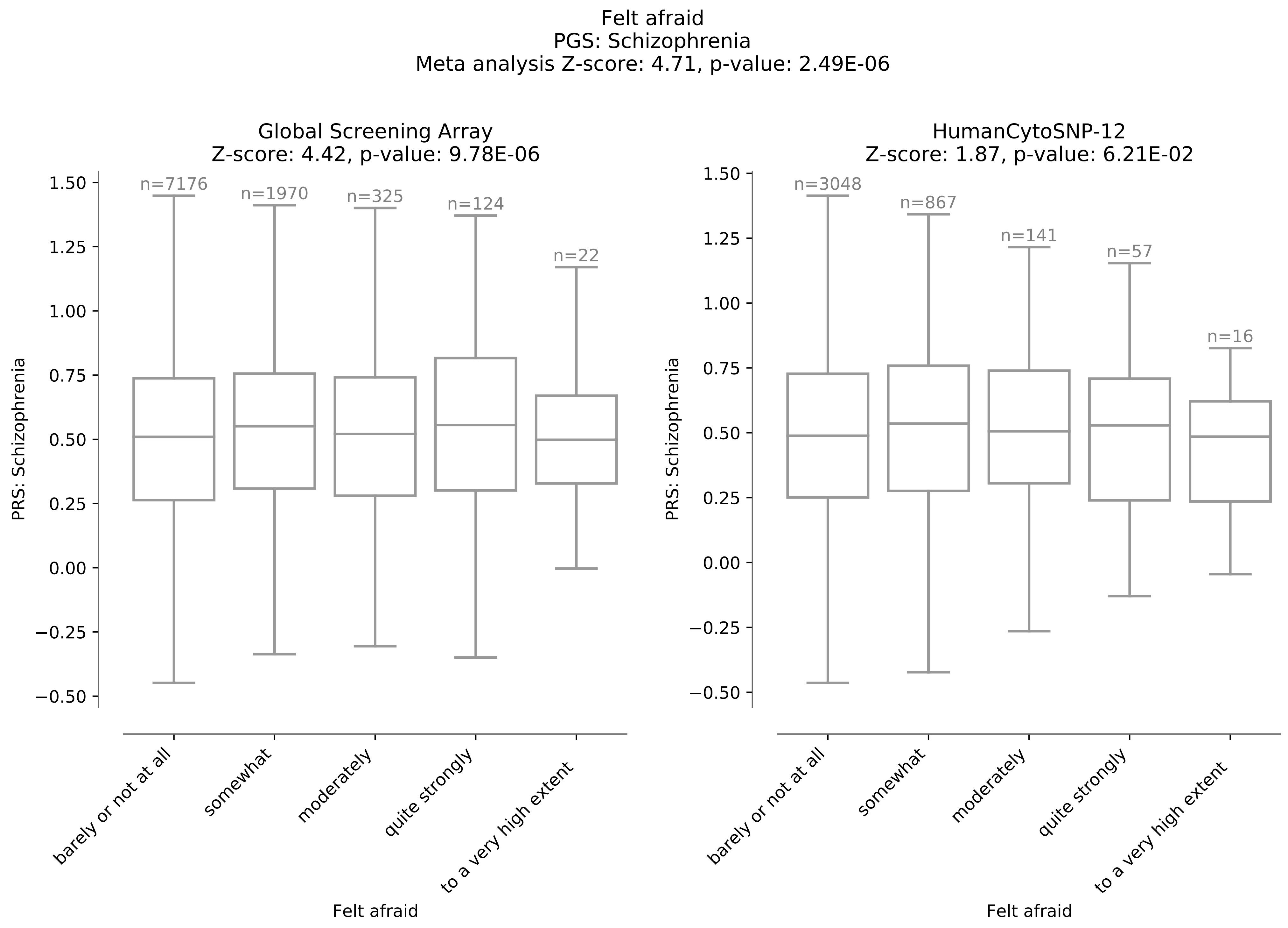
Felt afraid
PGS: Schizophrenia
Meta analysis Z-score: 4.71, p-value: 2.49E-06
Global Screening Array
Z-score: 4.42, p-value: 9.78E-06
HumanCytoSNP-12
Z-score: 1.87, p-value: 6.21E-02
PRS: Schizophrenia
n=7176
n=1970
n=325
n=124
n=22
n=3048
n=867
n=141
n=57
n=16
barely or not at all
somewhat
moderately
quite strongly
to a very high extent
Felt afraid

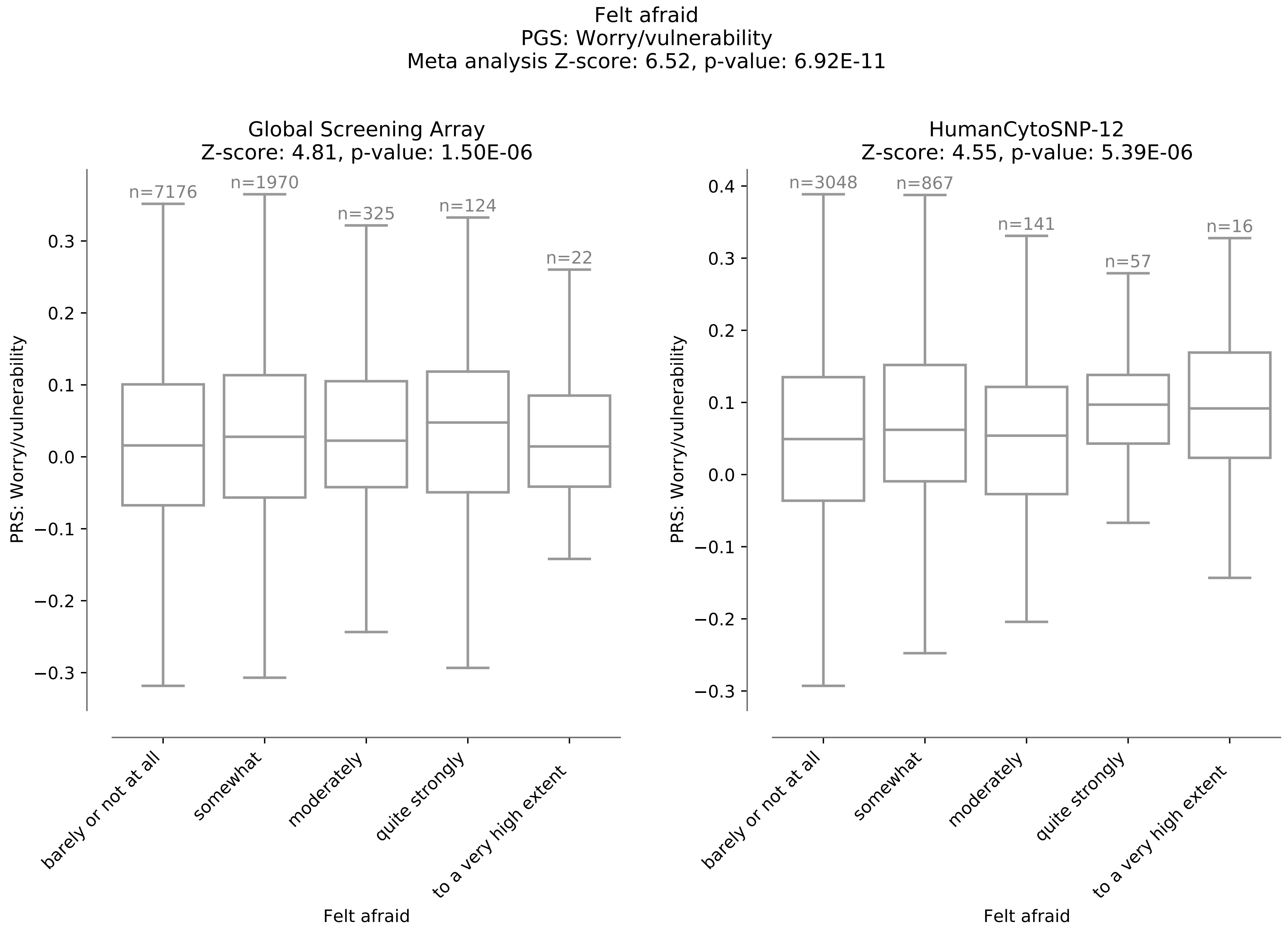
Felt afraid
PGS: Worry/vulnerability
Meta analysis Z-score: 6.52, p-value: 6.92E-11
Global Screening Array
Z-score: 4.81, p-value: 1.50E-06
HumanCytoSNP-12
Z-score: 4.55, p-value: 5.39E-06
PRS: Worry/vulnerability
n=7176
n=1970
n=325
n=124
n=22
n=3048
n=867
n=141
n=57
n=16
barely or not at all
somewhat
moderately
quite strongly
to a very high extent
Felt afraid

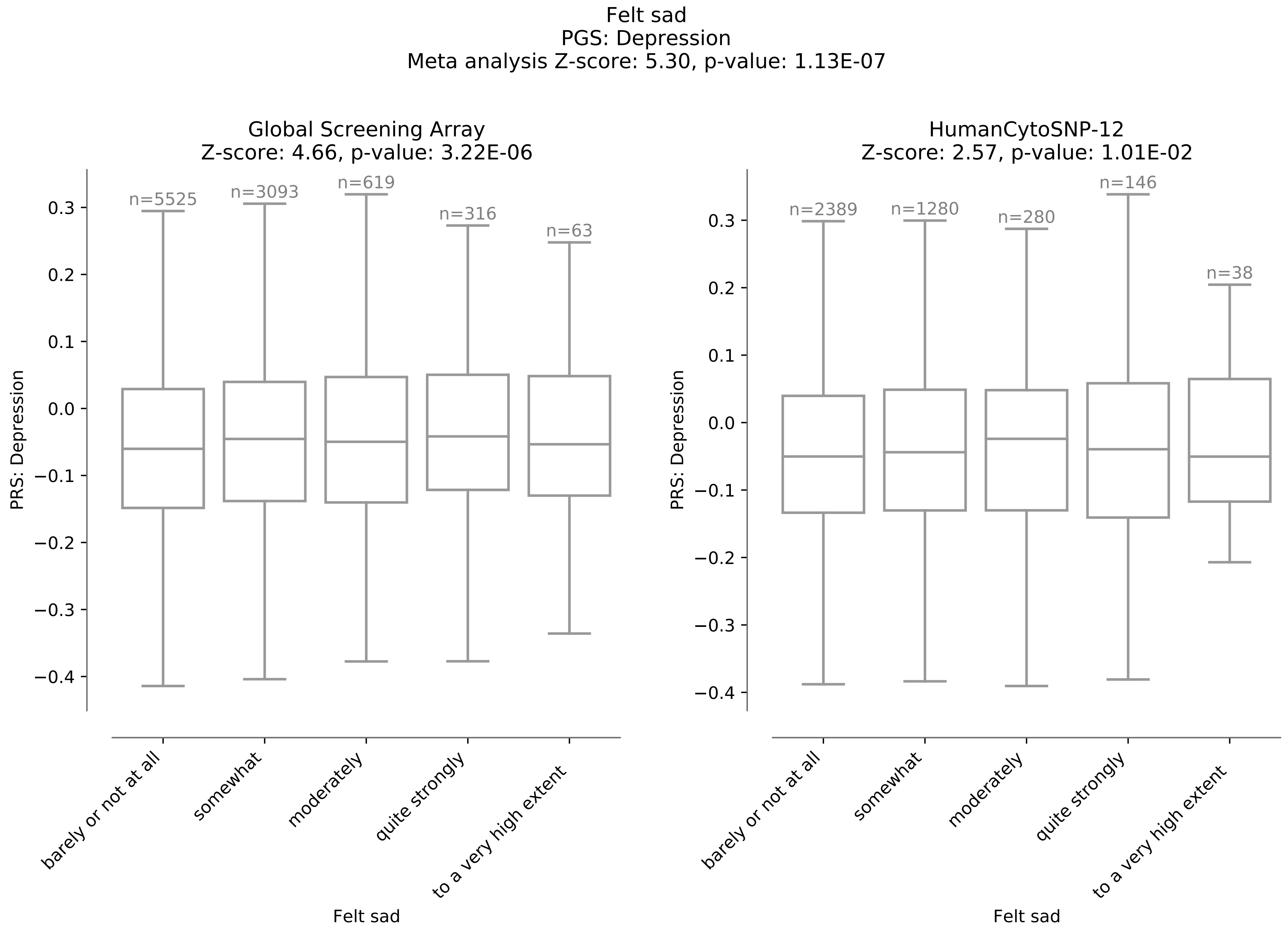
Felt sad
PGS: Depression
Meta analysis Z-score: 5.30, p-value: 1.13E-07
Global Screening Array
Z-score: 4.66, p-value: 3.22E-06
HumanCytoSNP-12
Z-score: 2.57, p-value: 1.01E-02
PRS: Depression
n=5525
n=3093
n=619
n=316
n=63
n=2389
n=1280
n=280
n=146
n=38
0.3
0.2
0.1
0.0
−0.1
−0.2
−0.3
−0.4
barely or not at all
somewhat
moderately
quite strongly
to a very high extent
Felt sad

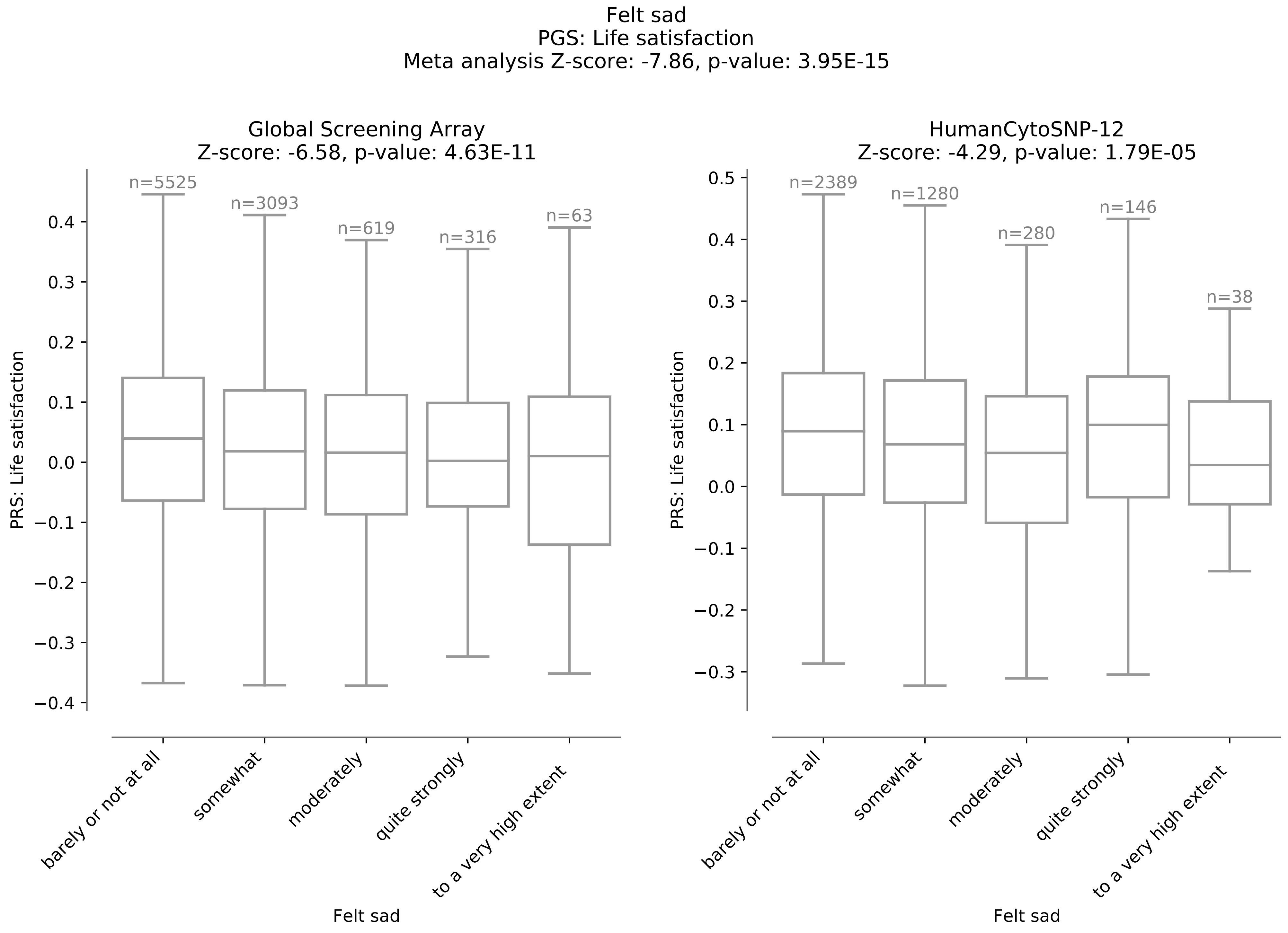

Felt sad
PGS: Life satisfaction
Meta analysis Z-score: -7.86, p-value: 3.95E-15
Global Screening Array
Z-score: -6.58, p-value: 4.63E-11
n=5525
n=3093
n=619
n=316
n=63
PRS: Life satisfaction
barely or not at all
somewhat
moderately
quite strongly
to a very high extent
Felt sad
HumanCytoSNP-12
Z-score: -4.29, p-value: 1.79E-05
n=2389
n=1280
n=280
n=146
n=38
PRS: Life satisfaction
barely or not at all
somewhat
moderately
quite strongly
to a very high extent
Felt sad

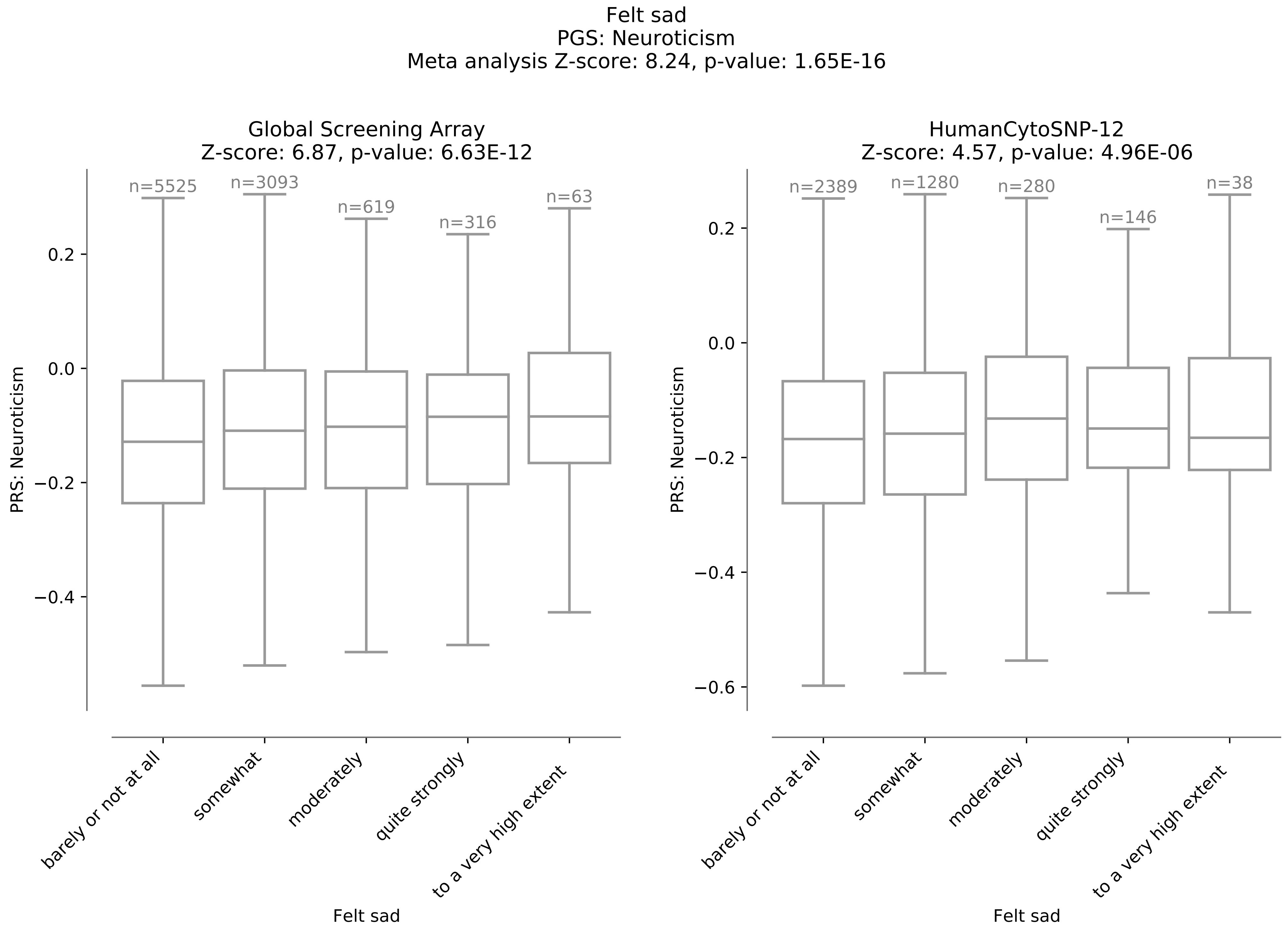
Felt sad
PGS: Neuroticism
Meta analysis Z-score: 8.24, p-value: 1.65E-16
Global Screening Array
Z-score: 6.87, p-value: 6.63E-12
n=5525
n=3093
n=619
n=316
n=63
PRS: Neuroticism
0.2
0.0
−0.2
−0.4
barely or not at all
somewhat
moderately
quite strongly
to a very high extent
Felt sad
HumanCytoSNP-12
Z-score: 4.57, p-value: 4.96E-06
n=2389
n=1280
n=280
n=146
n=38
PRS: Neuroticism
0.2
0.0
−0.2
−0.4
−0.6
barely or not at all
somewhat
moderately
quite strongly
to a very high extent
Felt sad

Having quarantined or gone into (self-)isolation during second wave
PGS: Educational attainment
Meta analysis Z-score: 5.09, p-value: 3.63E-07

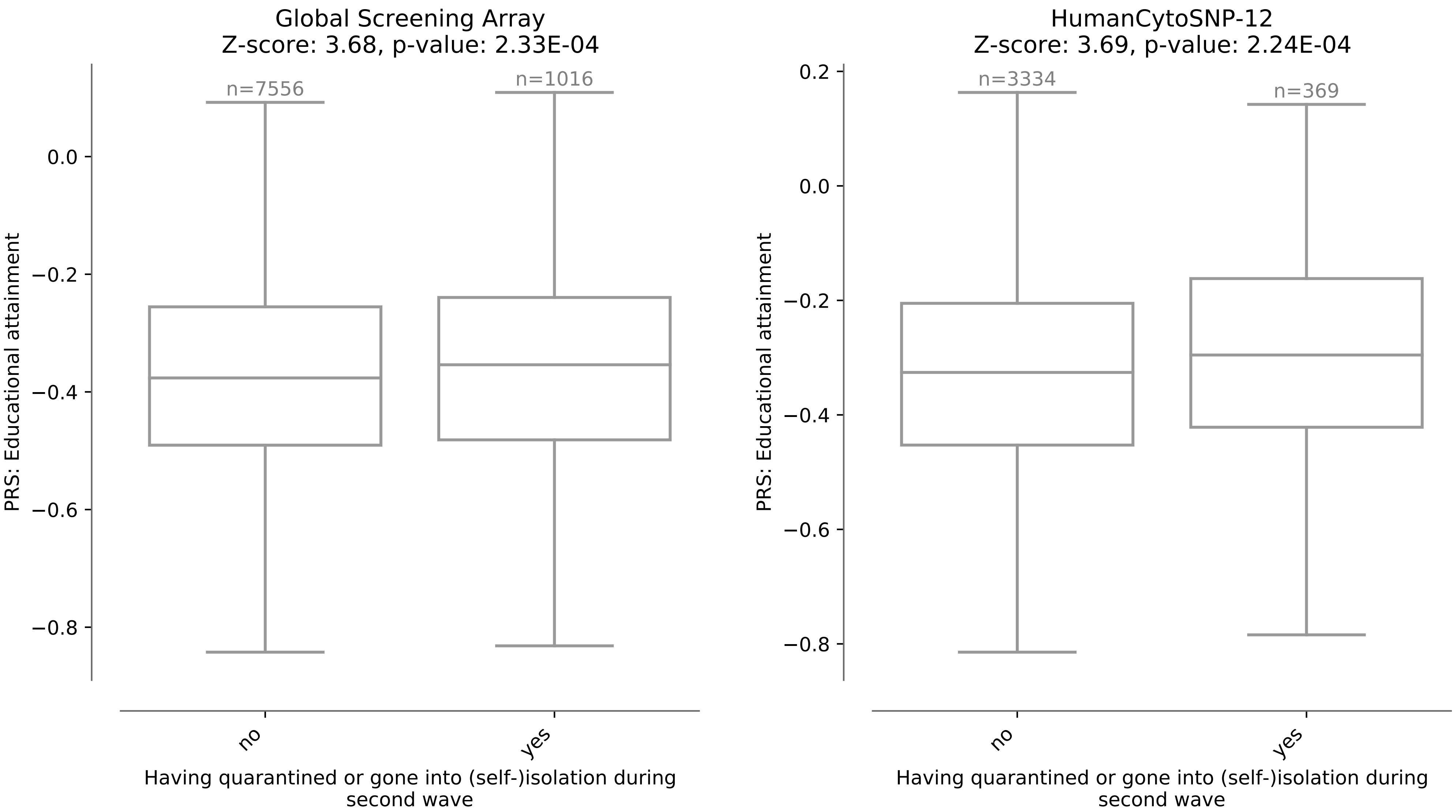

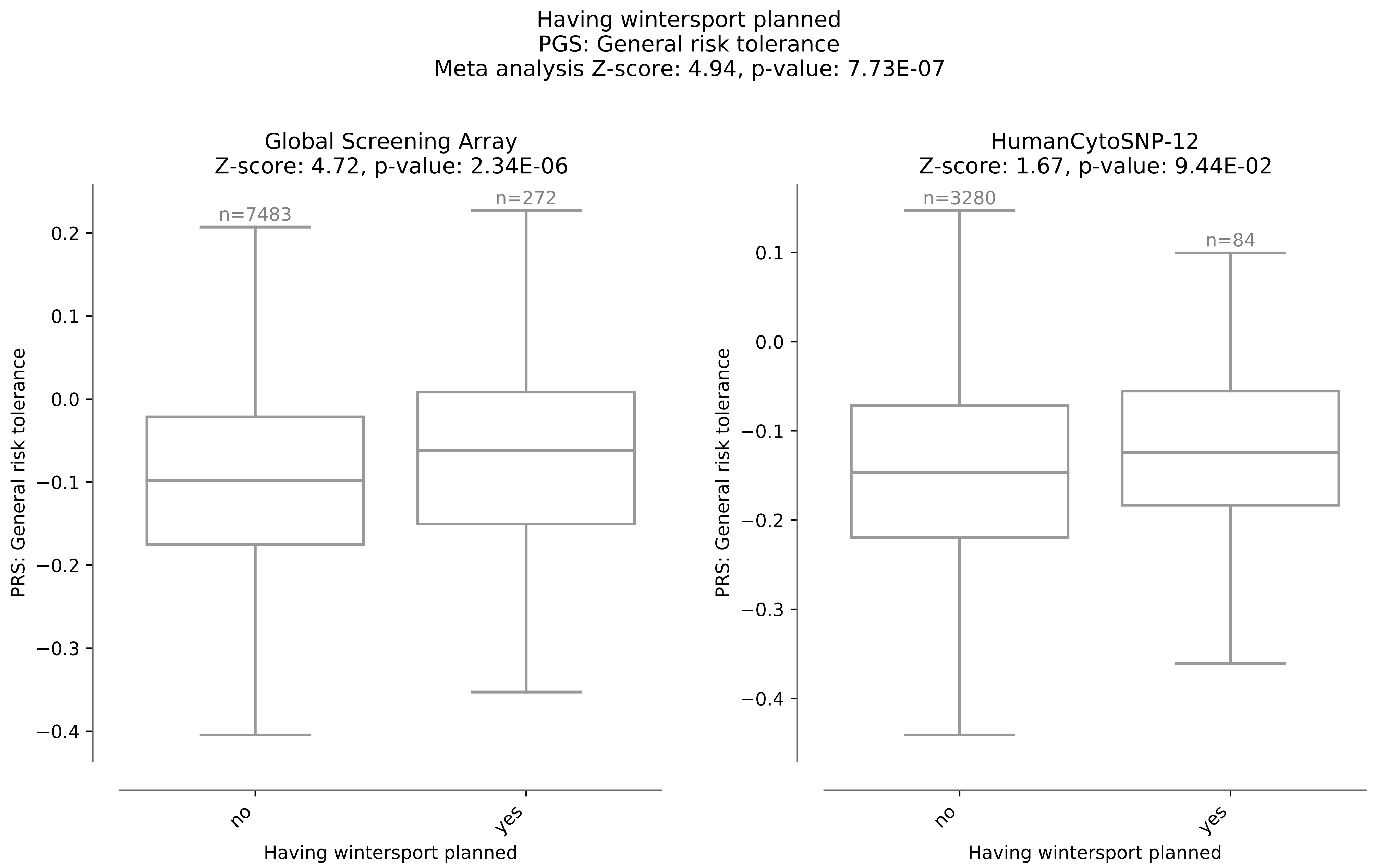
Having wintersport planned
PGS: General risk tolerance
Meta analysis Z-score: 4.94, p-value: 7.73E-07
Global Screening Array
Z-score: 4.72, p-value: 2.34E-06
n=7483
n=272
PRS: General risk tolerance
0.2
0.1
0.0
−0.1
−0.2
−0.3
−0.4
no
yes
Having wintersport planned
HumanCytoSNP-12
Z-score: 1.67, p-value: 9.44E-02
n=3280
n=84
PRS: General risk tolerance
0.1
0.0
−0.1
−0.2
−0.3
−0.4
no
yes
Having wintersport planned

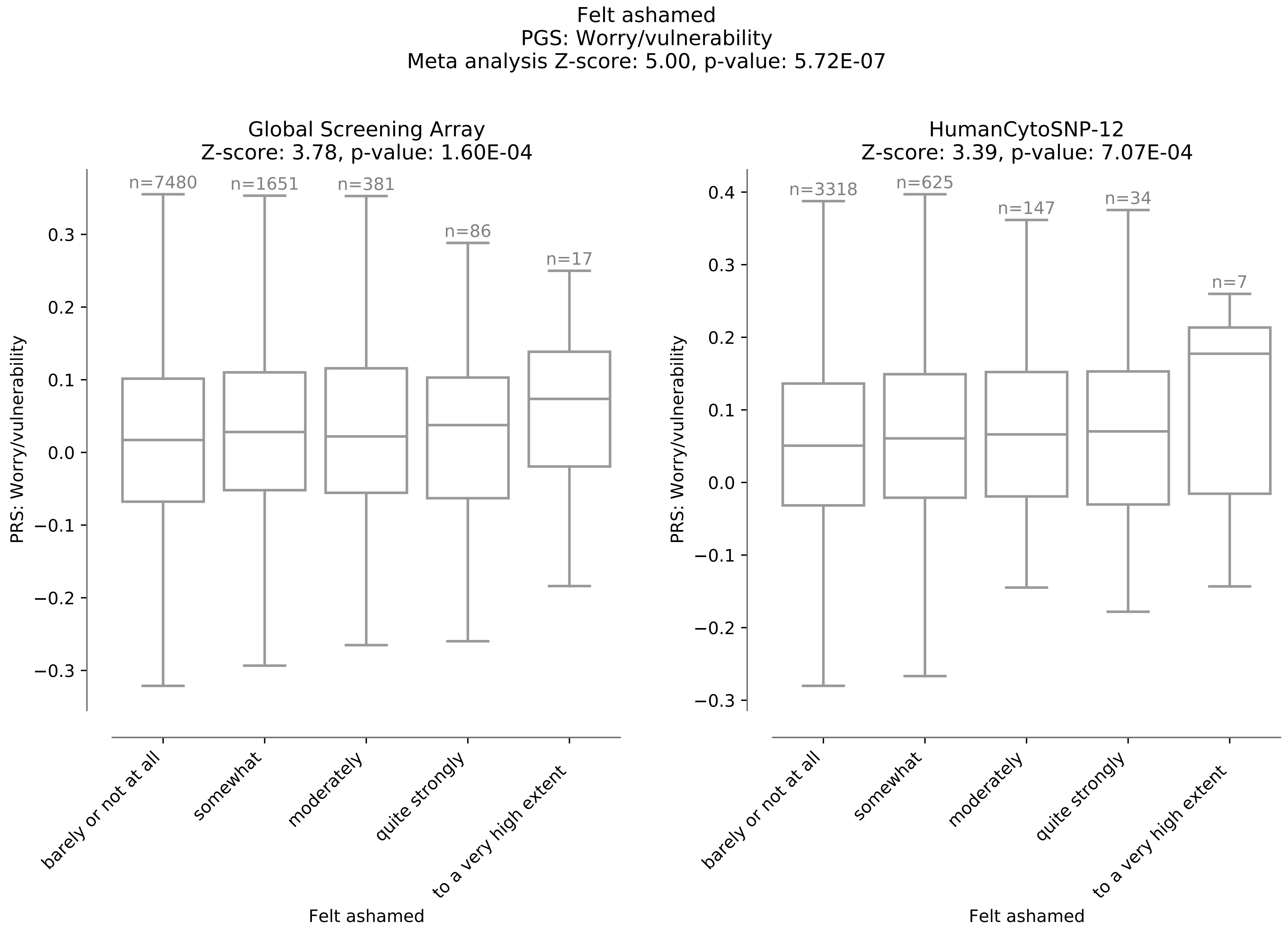
Felt ashamed
PGS: Worry/vulnerability
Meta analysis Z-score: 5.00, p-value: 5.72E-07
Global Screening Array
Z-score: 3.78, p-value: 1.60E-04
HumanCytoSNP-12
Z-score: 3.39, p-value: 7.07E-04
n=7480
n=1651
n=381
n=86
n=17
n=3318
n=625
n=147
n=34
n=7
PRS: Worry/vulnerability
barely or not at all
somewhat
moderately
quite strongly
to a very high extent
Felt ashamed

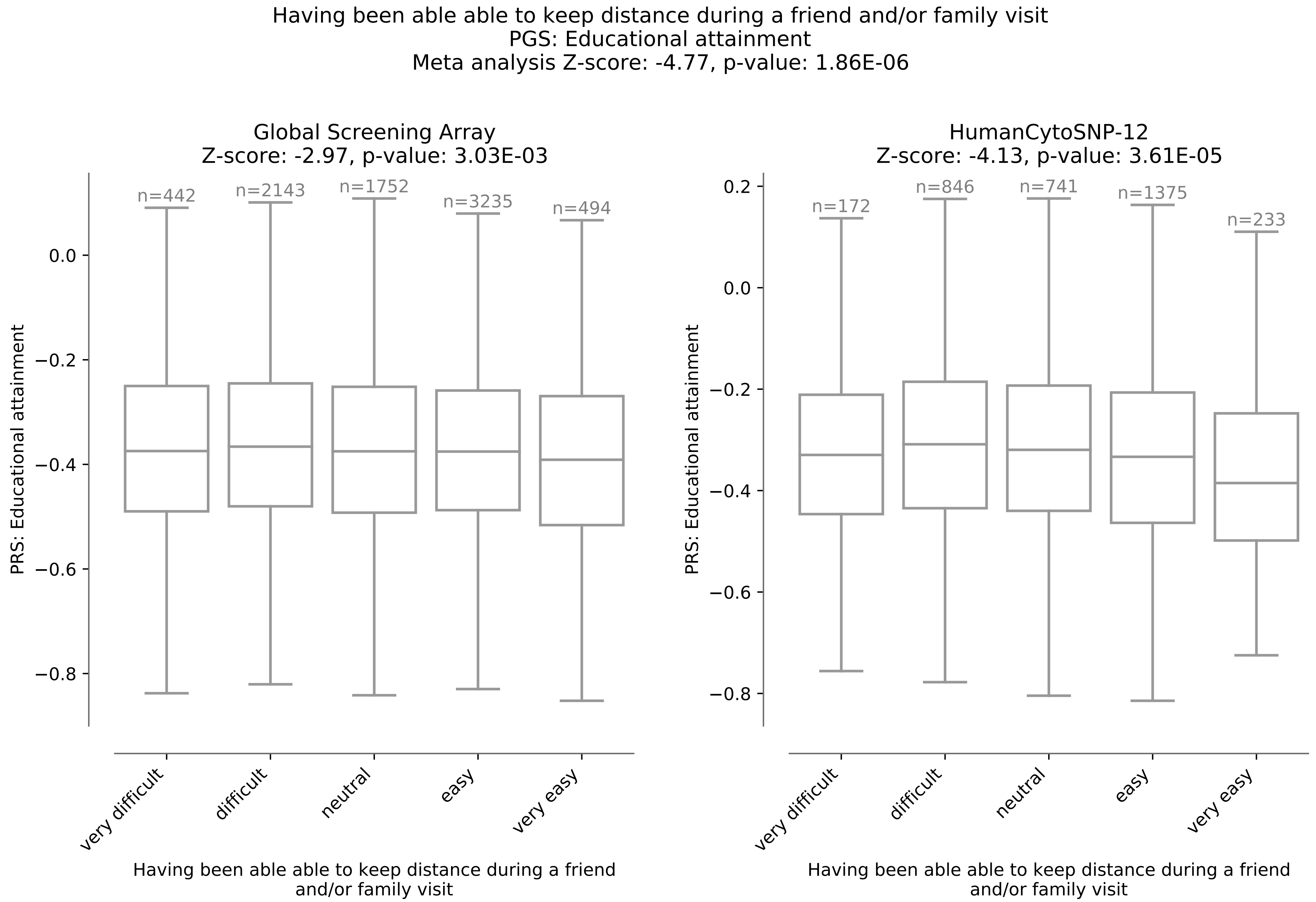
Having been able able to keep distance during a friend and/or family visit
PGS: Educational attainment
Meta analysis Z-score: -4.77, p-value: 1.86E-06
Global Screening Array
Z-score: -2.97, p-value: 3.03E-03
n=442
n=2143
n=1752
n=3235
n=494
PRS: Educational attainment
0.0
−0.2
−0.4
−0.6
−0.8
very difficult
difficult
neutral
easy
very easy
Having been able able to keep distance during a friend and/or family visit
HumanCytoSNP-12
Z-score: -4.13, p-value: 3.61E-05
n=172
n=846
n=741
n=1375
n=233
PRS: Educational attainment
0.2
0.0
−0.2
−0.4
−0.6
−0.8
very difficult
difficult
neutral
easy
very easy
Having been able able to keep distance during a friend and/or family visit

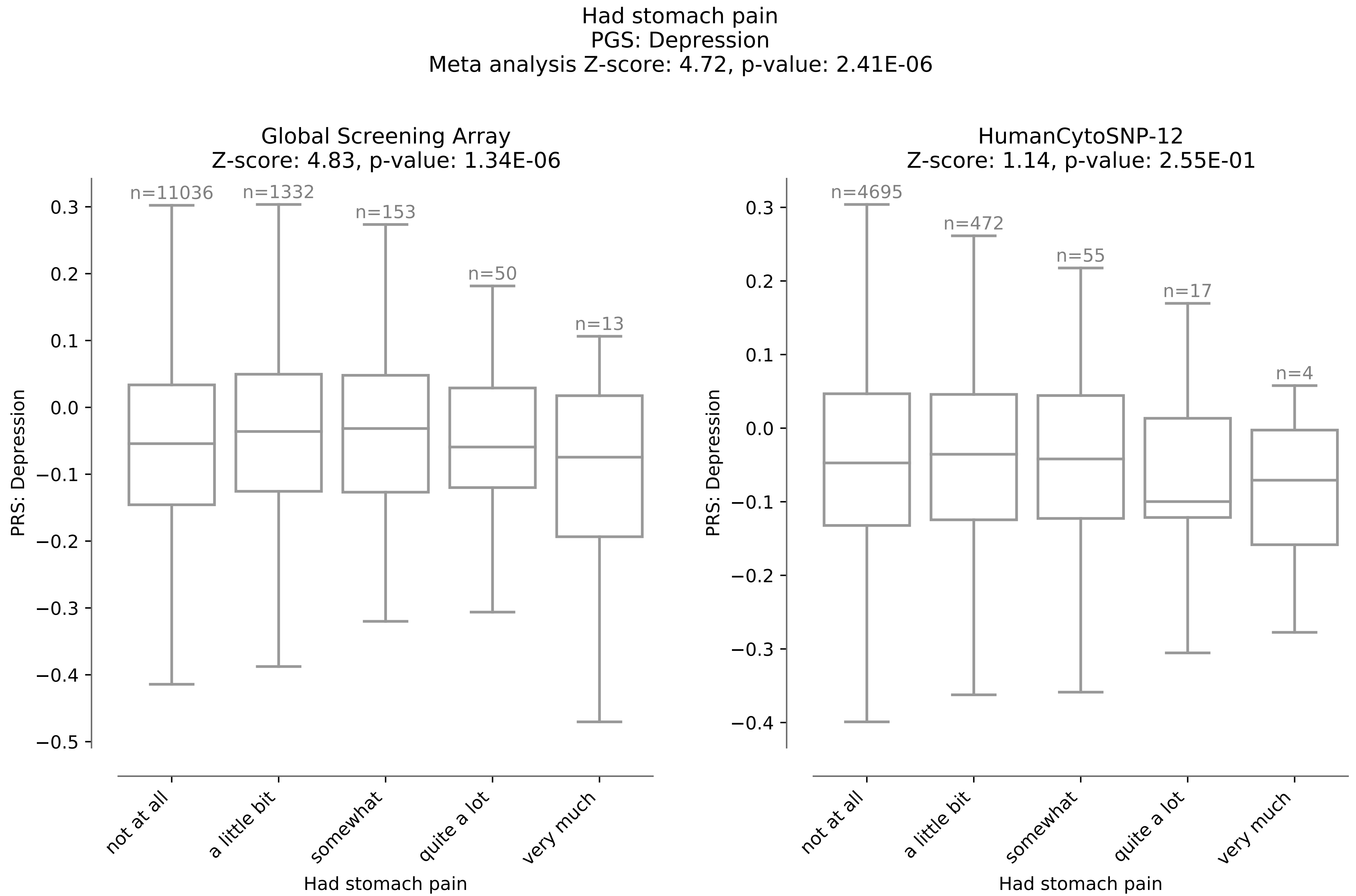
Had stomach pain
PGS: Depression
Meta analysis Z-score: 4.72, p-value: 2.41E-06
Global Screening Array
Z-score: 4.83, p-value: 1.34E-06
n=11036
n=1332
n=153
n=50
n=13
PRS: Depression
not at all
a little bit
somewhat
quite a lot
very much
Had stomach pain
HumanCytoSNP-12
Z-score: 1.14, p-value: 2.55E-01
n=4695
n=472
n=55
n=17
n=4
PRS: Depression
not at all
a little bit
somewhat
quite a lot
very much
Had stomach pain

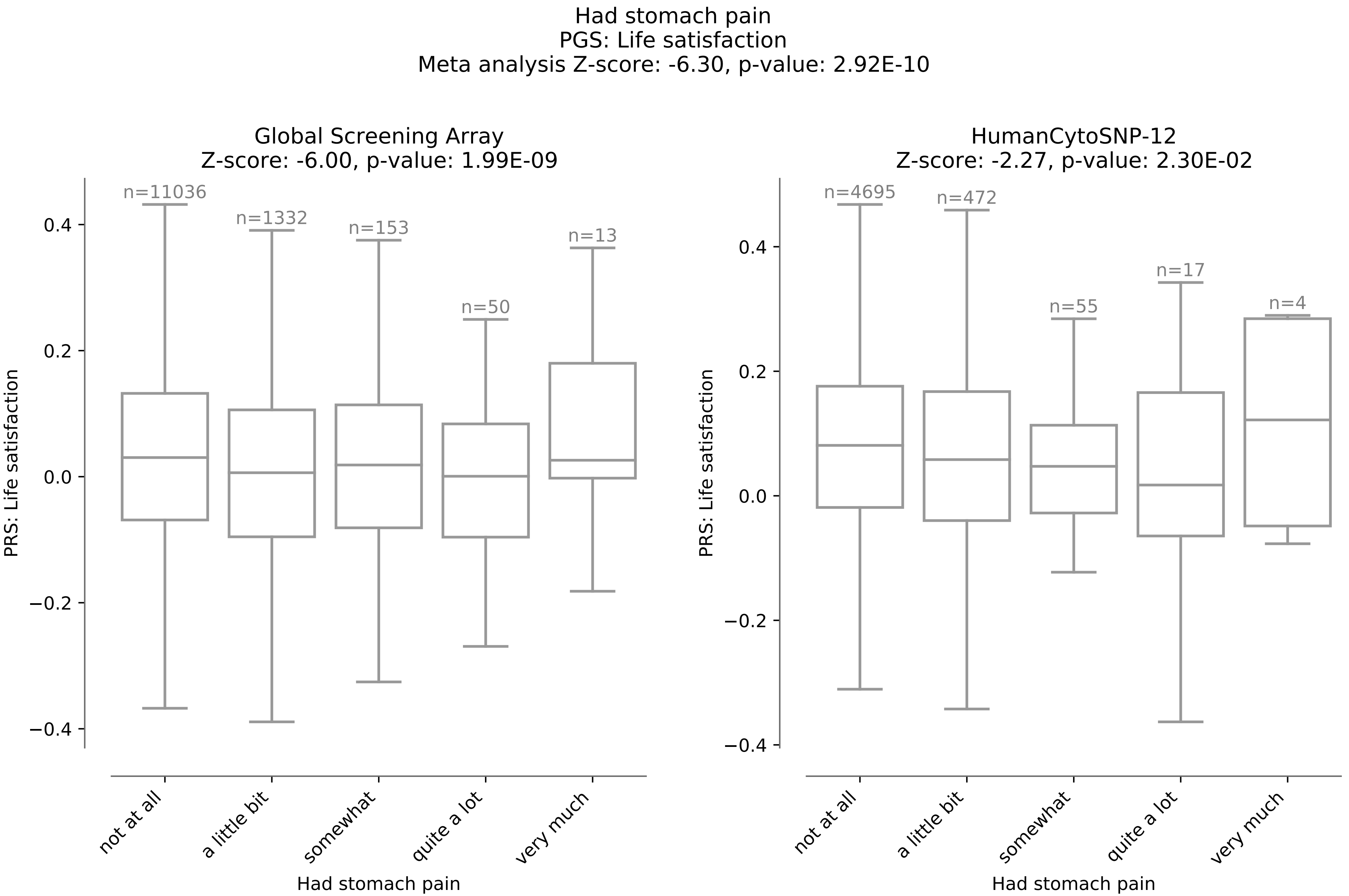
Had stomach pain
PGS: Life satisfaction
Meta analysis Z-score: -6.30, p-value: 2.92E-10
Global Screening Array
Z-score: -6.00, p-value: 1.99E-09
HumanCytoSNP-12
Z-score: -2.27, p-value: 2.30E-02
PRS: Life satisfaction
n=11036
n=1332
n=153
n=50
n=13
n=4695
n=472
n=55
n=17
n=4
not at all
a little bit
somewhat
quite a lot
very much
Had stomach pain

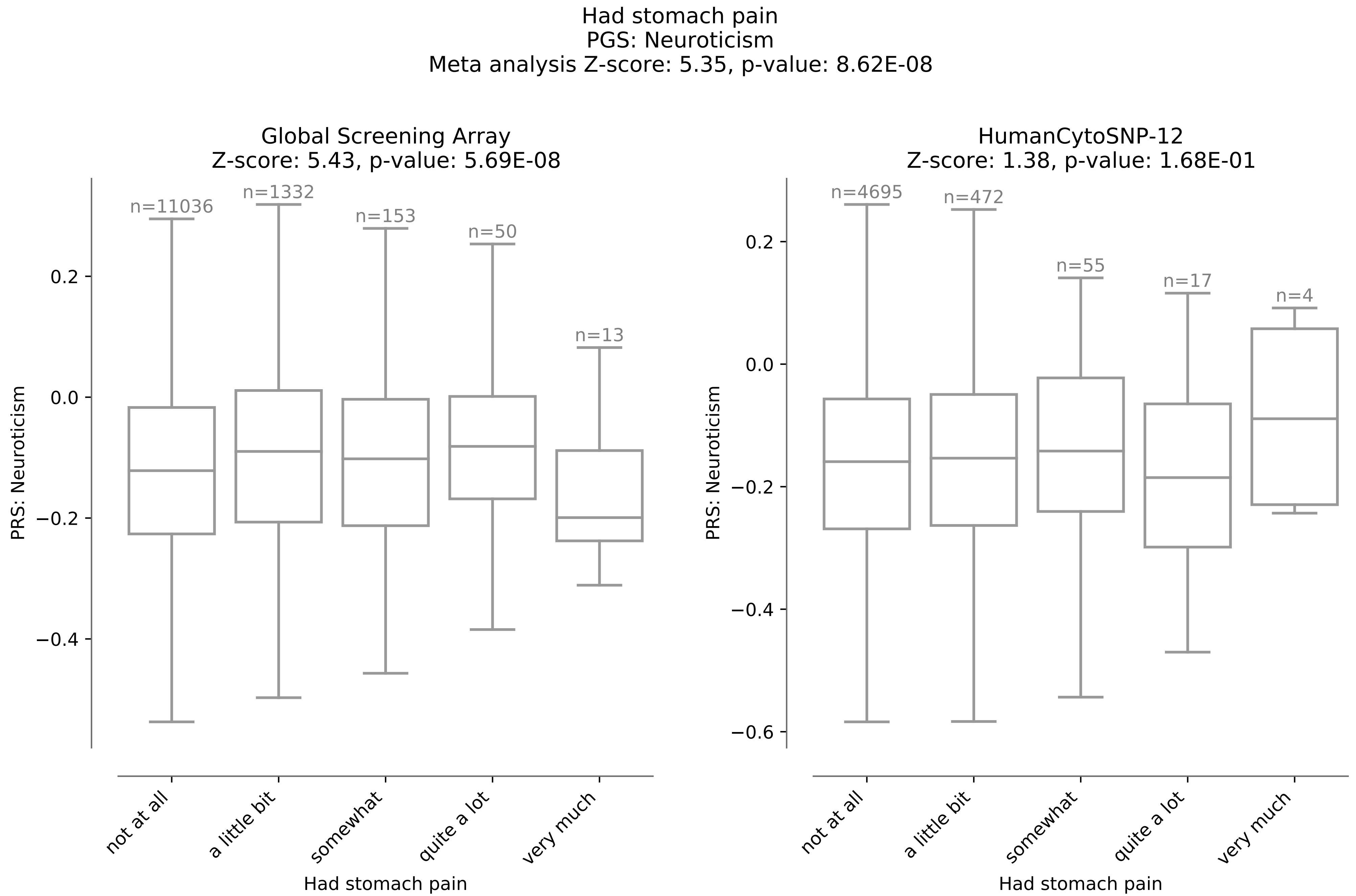
Had stomach pain
PGS: Neuroticism
Meta analysis Z-score: 5.35, p-value: 8.62E-08
Global Screening Array
Z-score: 5.43, p-value: 5.69E-08
n=11036
n=1332
n=153
n=50
n=13
PRS: Neuroticism
0.2
0.0
−0.2
−0.4
not at all
a little bit
somewhat
quite a lot
very much
Had stomach pain
HumanCytoSNP-12
Z-score: 1.38, p-value: 1.68E-01
n=4695
n=472
n=55
n=17
n=4
PRS: Neuroticism
0.2
0.0
−0.2
−0.4
−0.6
not at all
a little bit
somewhat
quite a lot
very much
Had stomach pain

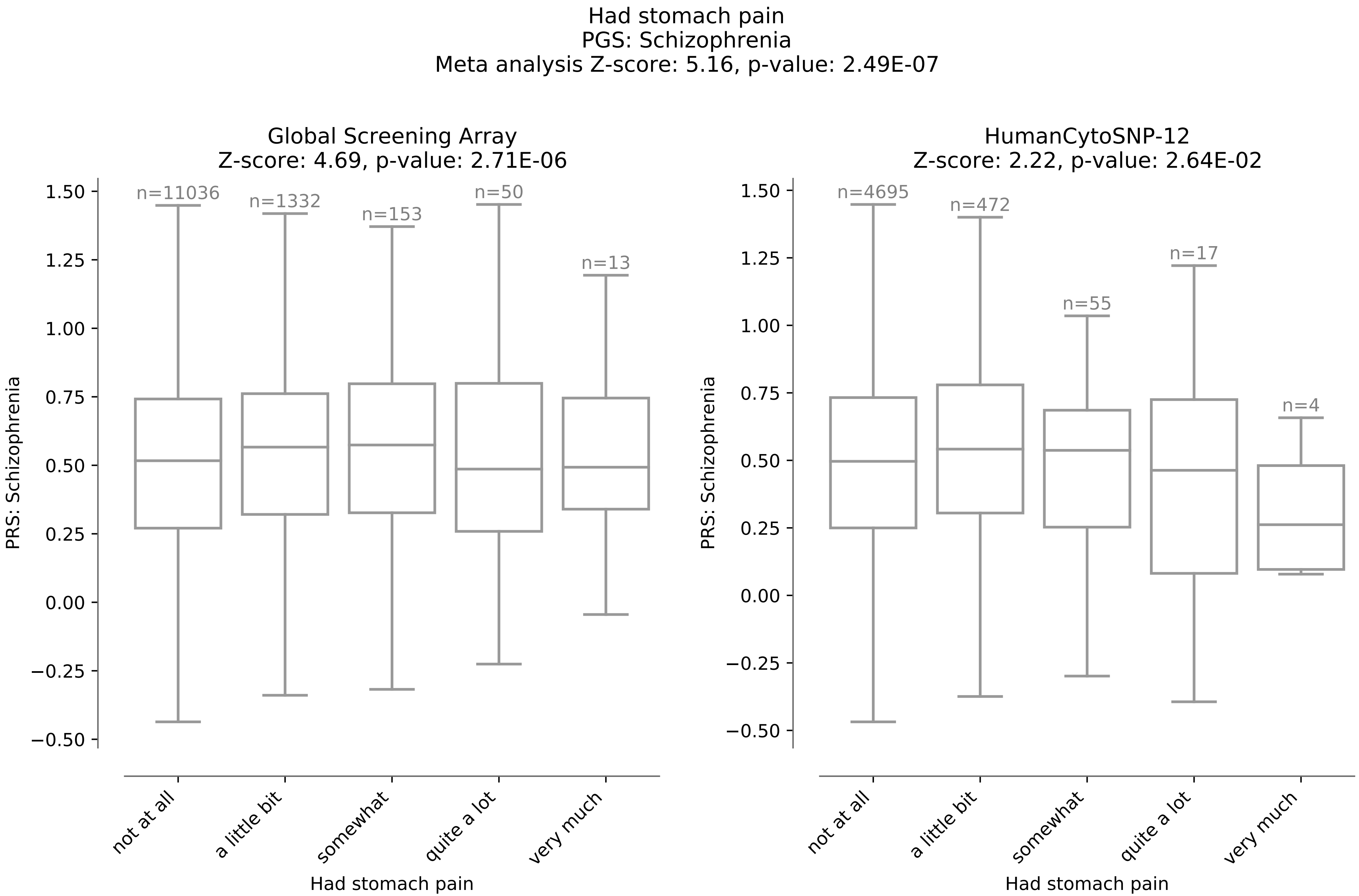
Had stomach pain
PGS: Schizophrenia
Meta analysis Z-score: 5.16, p-value: 2.49E-07
Global Screening Array
Z-score: 4.69, p-value: 2.71E-06
HumanCytoSNP-12
Z-score: 2.22, p-value: 2.64E-02
PRS: Schizophrenia
n=11036
n=1332
n=153
n=50
n=13
n=4695
n=472
n=55
n=17
n=4
1.50
1.25
1.00
0.75
0.50
0.25
0.00
−0.25
−0.50
not at all
a little bit
somewhat
quite a lot
very much
Had stomach pain

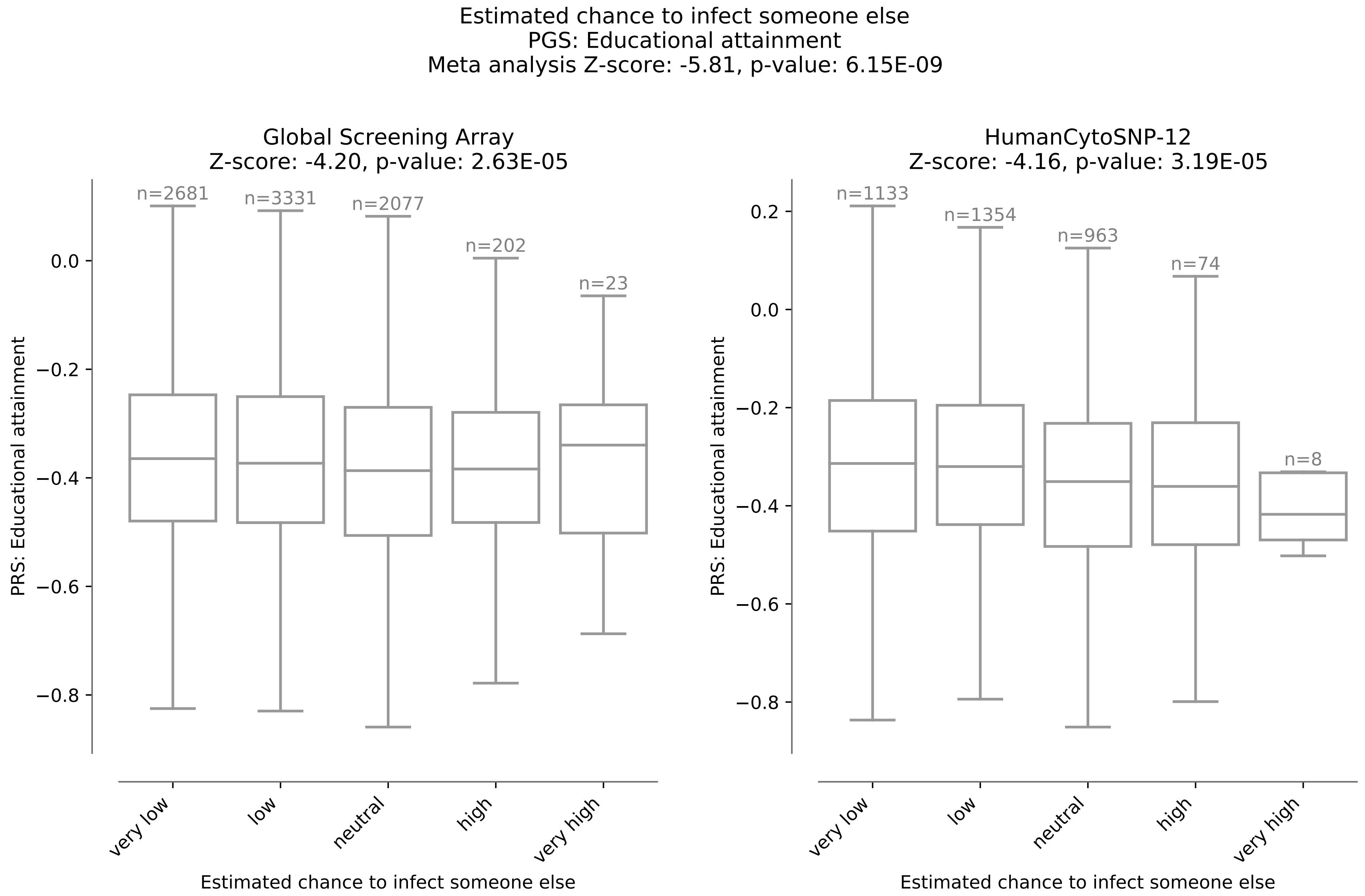

Estimated chance to infect someone else
PGS: Educational attainment
Meta analysis Z-score: -5.81, p-value: 6.15E-09
Global Screening Array
Z-score: -4.20, p-value: 2.63E-05
HumanCytoSNP-12
Z-score: -4.16, p-value: 3.19E-05
PRS: Educational attainment
n=2681
n=3331
n=2077
n=202
n=23
n=1133
n=1354
n=963
n=74
n=8
0.2
0.0
−0.2
−0.4
−0.6
−0.8
very low
low
neutral
high
very high
Estimated chance to infect someone else

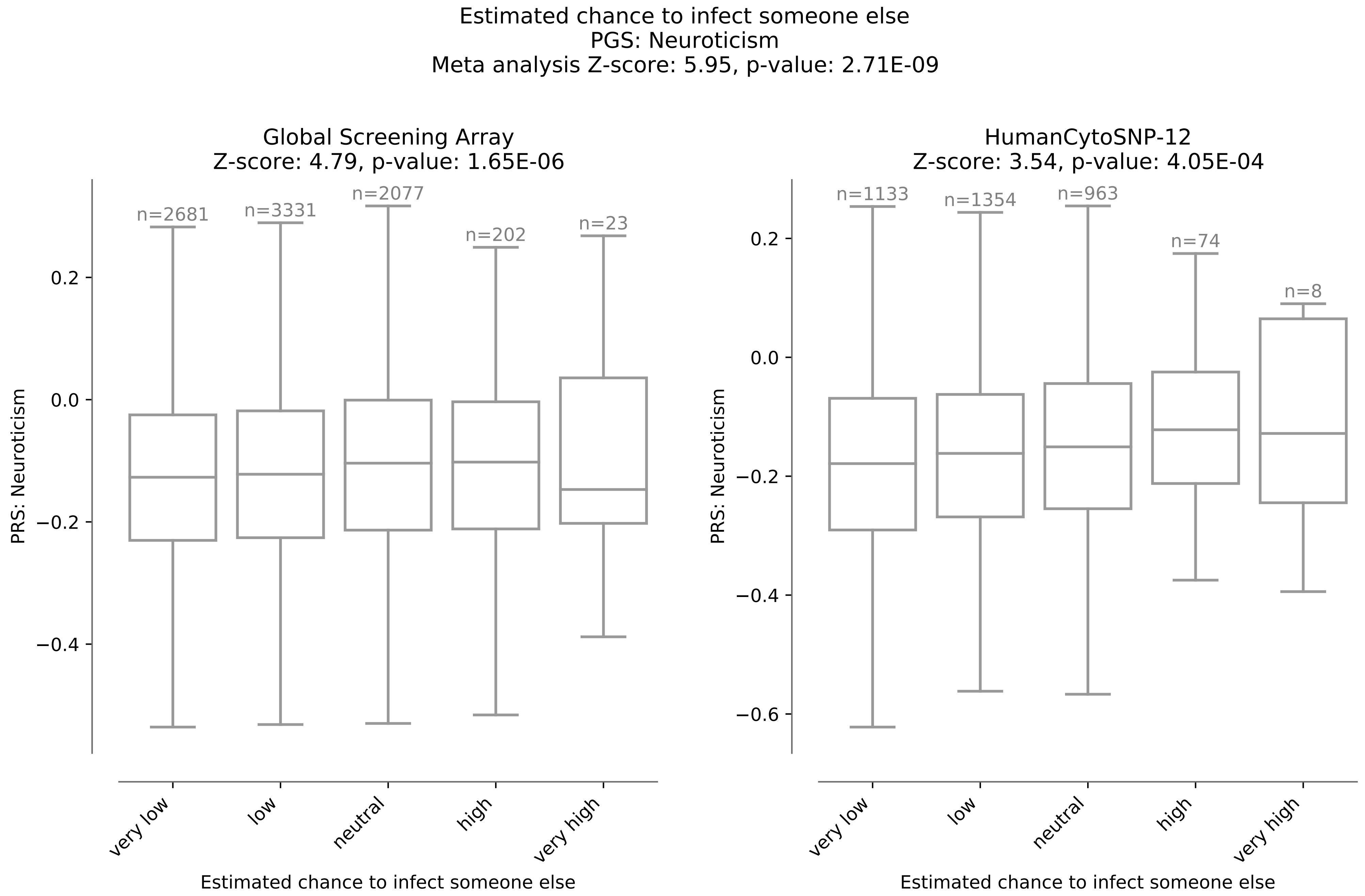

Estimated chance to infect someone else
PGS: Neuroticism
Meta analysis Z-score: 5.95, p-value: 2.71E-09
Global Screening Array
Z-score: 4.79, p-value: 1.65E-06
n=2681
n=3331
n=2077
n=202
n=23
0.2
0.0
−0.2
−0.4
PRS: Neuroticism
very low
low
neutral
high
very high
Estimated chance to infect someone else
HumanCytoSNP-12
Z-score: 3.54, p-value: 4.05E-04
n=1133
n=1354
n=963
n=74
n=8
0.2
0.0
−0.2
−0.4
−0.6
PRS: Neuroticism
very low
low
neutral
high
very high
Estimated chance to infect someone else

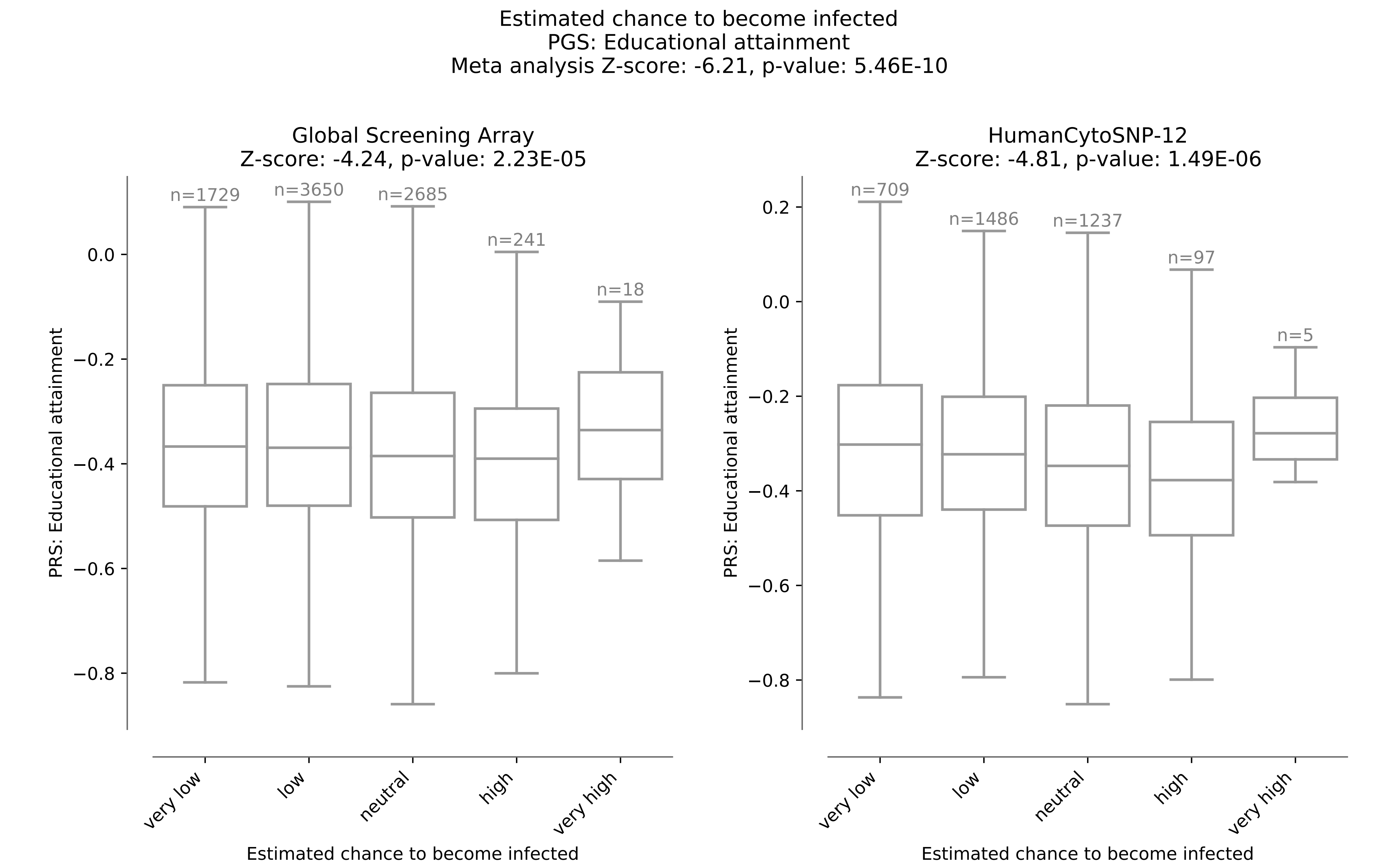

Estimated chance to become infected
PGS: Educational attainment
Meta analysis Z-score: -6.21, p-value: 5.46E-10
Global Screening Array
Z-score: -4.24, p-value: 2.23E-05
n=1729
n=3650
n=2685
n=241
n=18
PRS: Educational attainment
0.0
−0.2
−0.4
−0.6
−0.8
very low
low
neutral
high
very high
Estimated chance to become infected
HumanCytoSNP-12
Z-score: -4.81, p-value: 1.49E-06
n=709
n=1486
n=1237
n=97
n=5
PRS: Educational attainment
0.2
0.0
−0.2
−0.4
−0.6
−0.8
very low
low
neutral
high
very high
Estimated chance to become infected

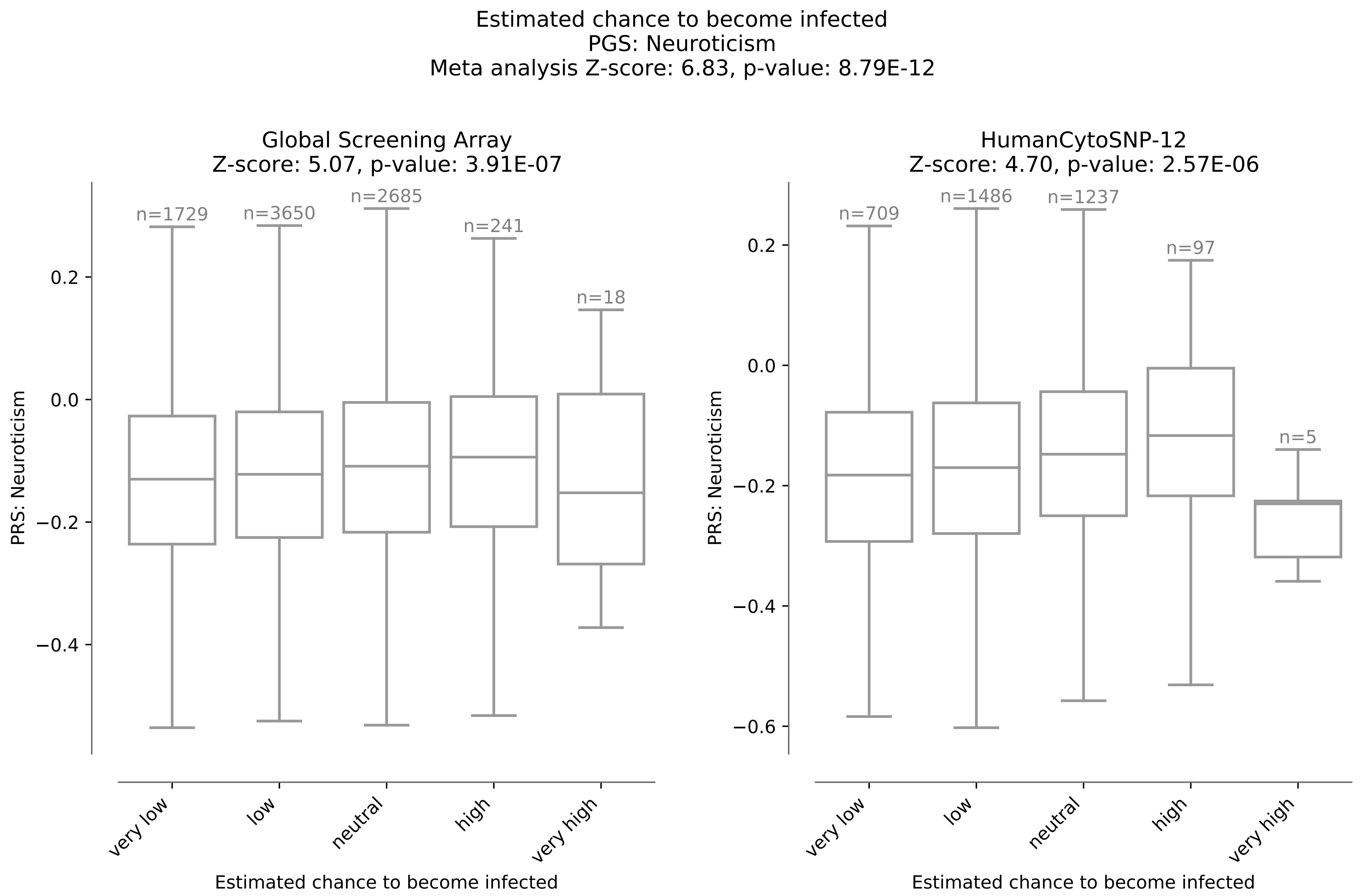

Estimated chance to become infected
PGS: Neuroticism
Meta analysis Z-score: 6.83, p-value: 8.79E-12
Global Screening Array
Z-score: 5.07, p-value: 3.91E-07
HumanCytoSNP-12
Z-score: 4.70, p-value: 2.57E-06
PRS: Neuroticism
n=1729
n=3650
n=2685
n=241
n=18
n=709
n=1486
n=1237
n=97
n=5
0.2
0.0
−0.2
−0.4
−0.6
very low
low
neutral
high
very high
Estimated chance to become infected

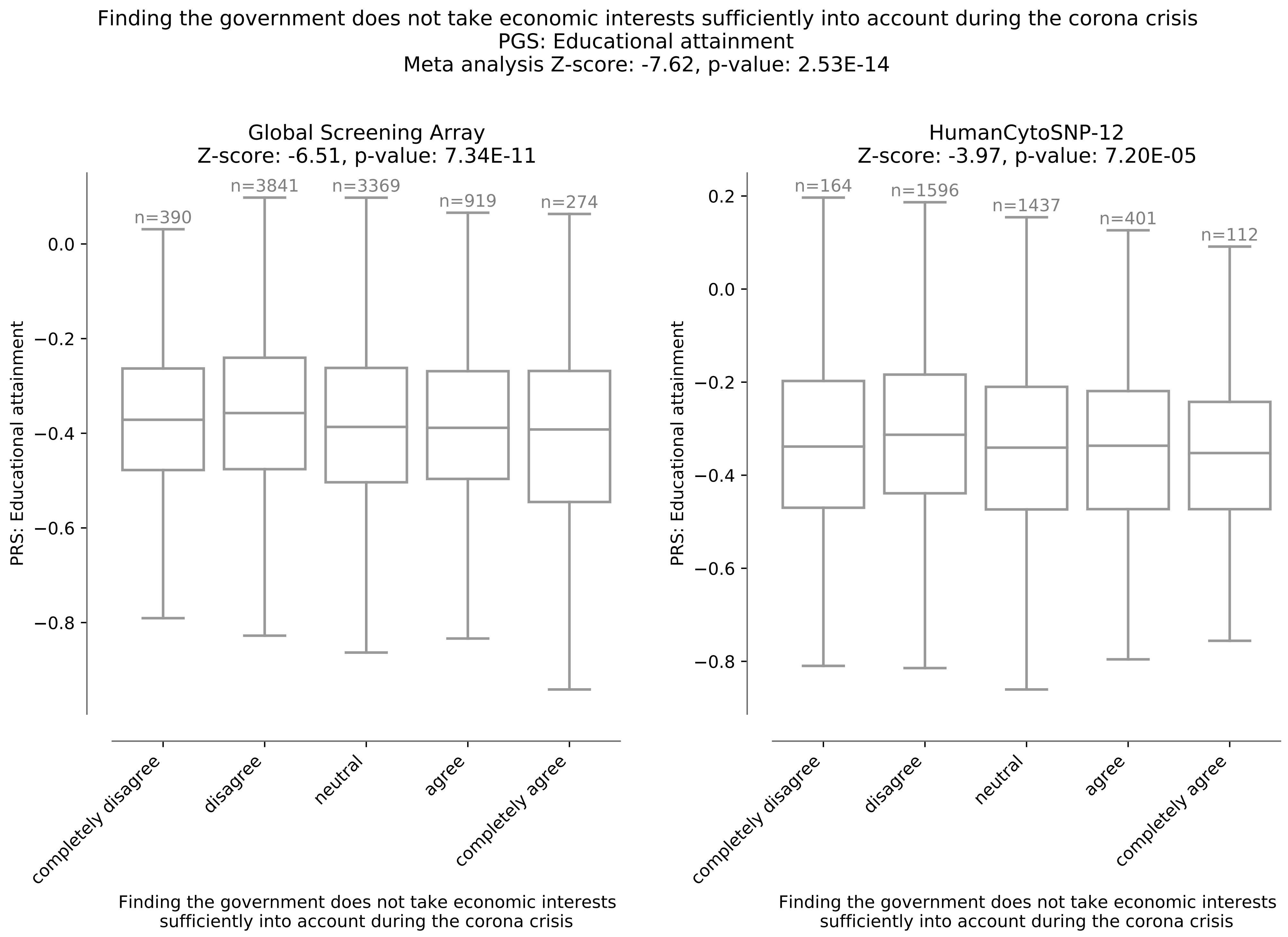

Finding the government does not take economic interests sufficiently into account during the corona crisis
PGS: Educational attainment
Meta analysis Z-score: -7.62, p-value: 2.53E-14
Global Screening Array
Z-score: -6.51, p-value: 7.34E-11
HumanCytoSNP-12
Z-score: -3.97, p-value: 7.20E-05
n=390
n=3841
n=3369
n=919
n=274
n=164
n=1596
n=1437
n=401
n=112
PRS: Educational attainment
0.2
0.0
−0.2
−0.4
−0.6
−0.8
completely disagree
disagree
neutral
agree
completely agree
Finding the government does not take economic interests sufficiently into account during the corona crisis

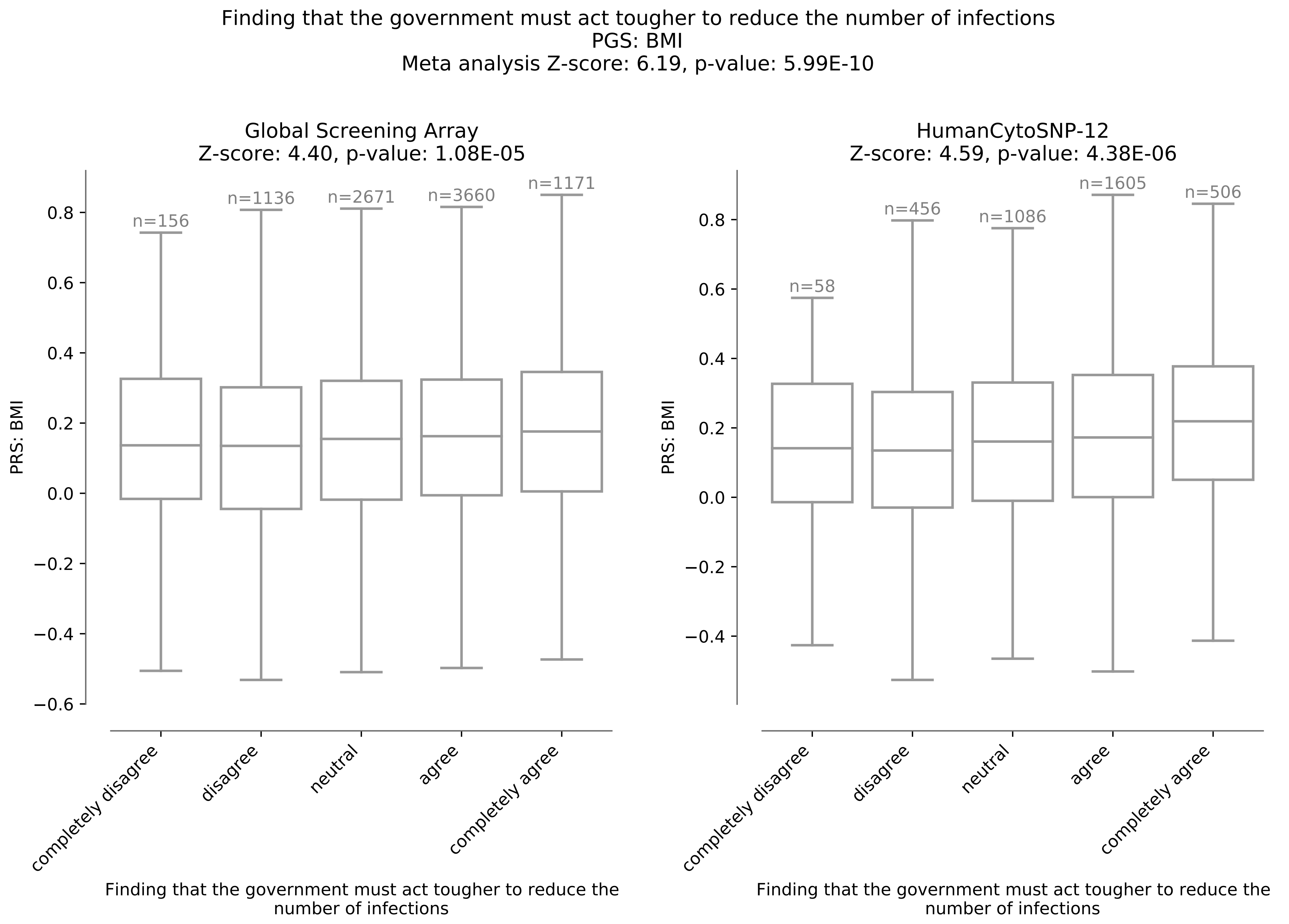
Finding that the government must act tougher to reduce the number of infections
PGS: BMI
Meta analysis Z-score: 6.19, p-value: 5.99E-10
Global Screening Array
Z-score: 4.40, p-value: 1.08E-05
HumanCytoSNP-12
Z-score: 4.59, p-value: 4.38E-06
PRS: BMI
n=156
n=1136
n=2671
n=3660
n=1171
n=58
n=456
n=1086
n=1605
n=506
0.8
0.6
0.4
0.2
0.0
−0.2
−0.4
−0.6
completely disagree
disagree
neutral
agree
completely agree
Finding that the government must act tougher to reduce the number of infections

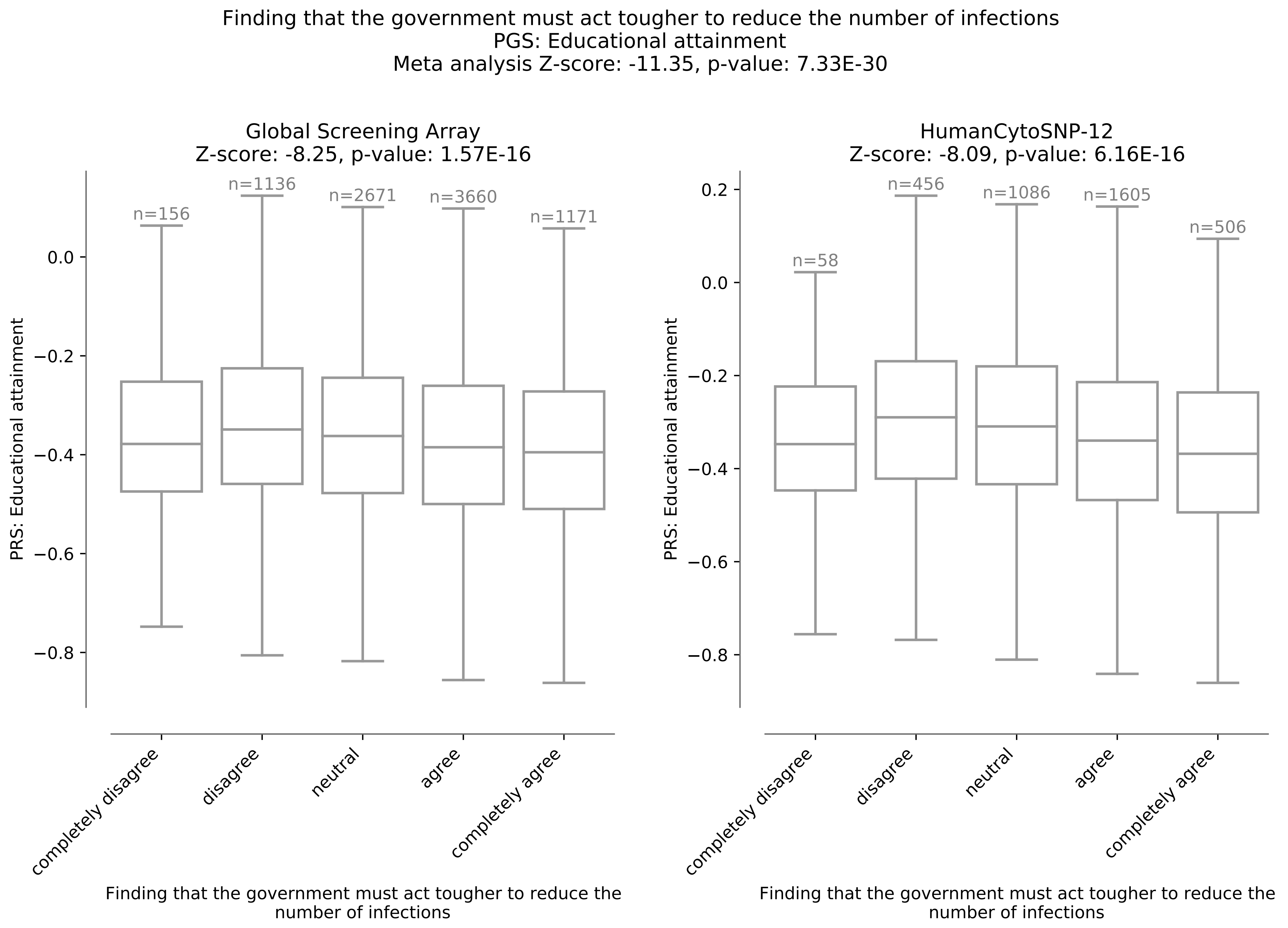
Finding that the government must act tougher to reduce the number of infections
PGS: Educational attainment
Meta analysis Z-score: -11.35, p-value: 7.33E-30
Global Screening Array
Z-score: -8.25, p-value: 1.57E-16
n=156
n=1136
n=2671
n=3660
n=1171
PRS: Educational attainment
0.0
−0.2
−0.4
−0.6
−0.8
completely disagree
disagree
neutral
agree
completely agree
Finding that the government must act tougher to reduce the number of infections
HumanCytoSNP-12
Z-score: -8.09, p-value: 6.16E-16
n=58
n=456
n=1086
n=1605
n=506
PRS: Educational attainment
0.2
0.0
−0.2
−0.4
−0.6
−0.8
completely disagree
disagree
neutral
agree
completely agree
Finding that the government must act tougher to reduce the number of infections

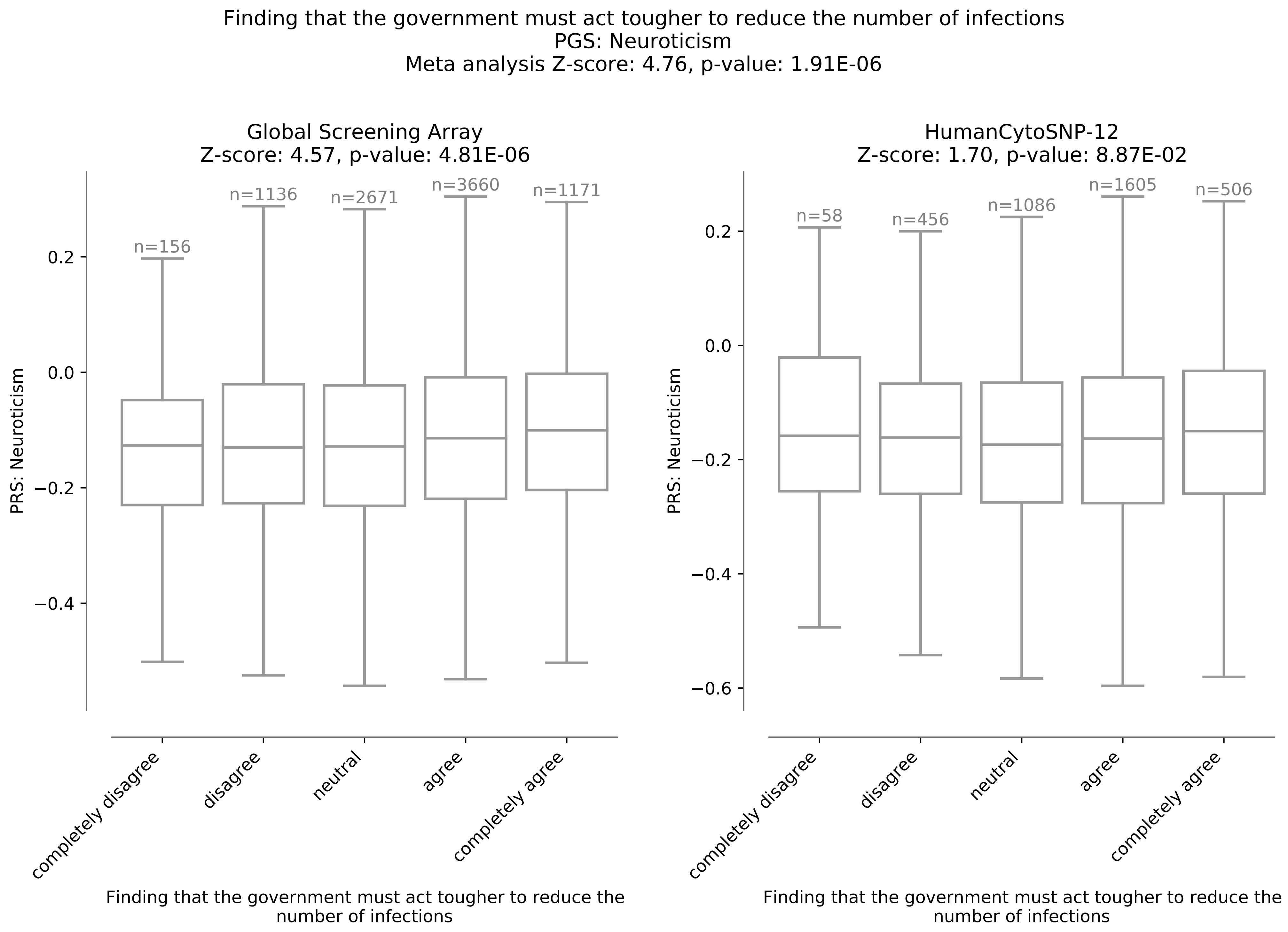

Finding that the government must act tougher to reduce the number of infections
PGS: Neuroticism
Meta analysis Z-score: 4.76, p-value: 1.91E-06
Global Screening Array
Z-score: 4.57, p-value: 4.81E-06
HumanCytoSNP-12
Z-score: 1.70, p-value: 8.87E-02
PRS: Neuroticism
n=156
n=1136
n=2671
n=3660
n=1171
n=58
n=456
n=1086
n=1605
n=506
0.2
0.0
−0.2
−0.4
−0.6
completely disagree
disagree
neutral
agree
completely agree
Finding that the government must act tougher to reduce the number of infections

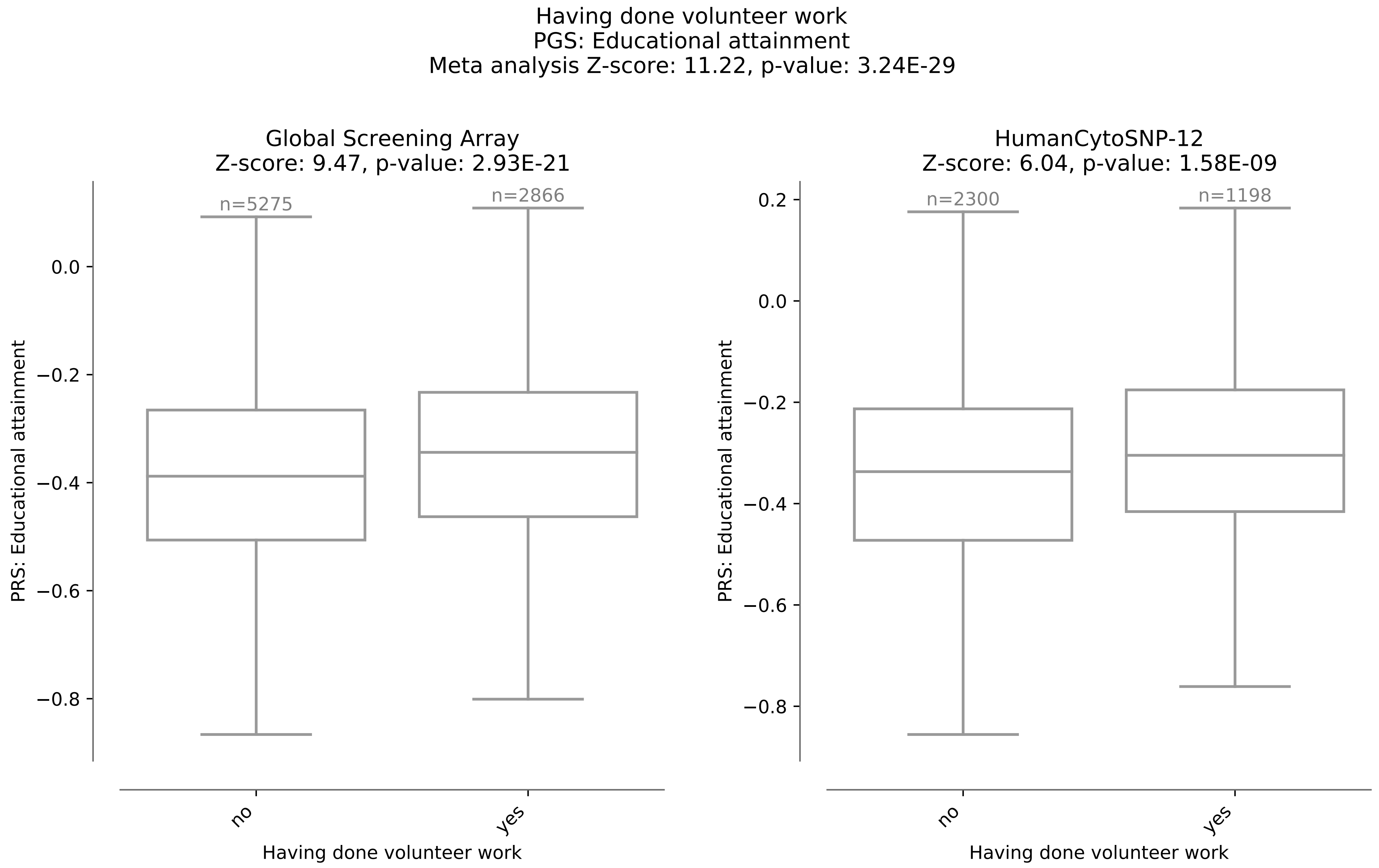
Having done volunteer work
PGS: Educational attainment
Meta analysis Z-score: 11.22, p-value: 3.24E-29
Global Screening Array
Z-score: 9.47, p-value: 2.93E-21
HumanCytoSNP-12
Z-score: 6.04, p-value: 1.58E-09
n=5275
n=2866
n=2300
n=1198
PRS: Educational attainment
0.2
0.0
−0.2
−0.4
−0.6
−0.8
no
yes
Having done volunteer work

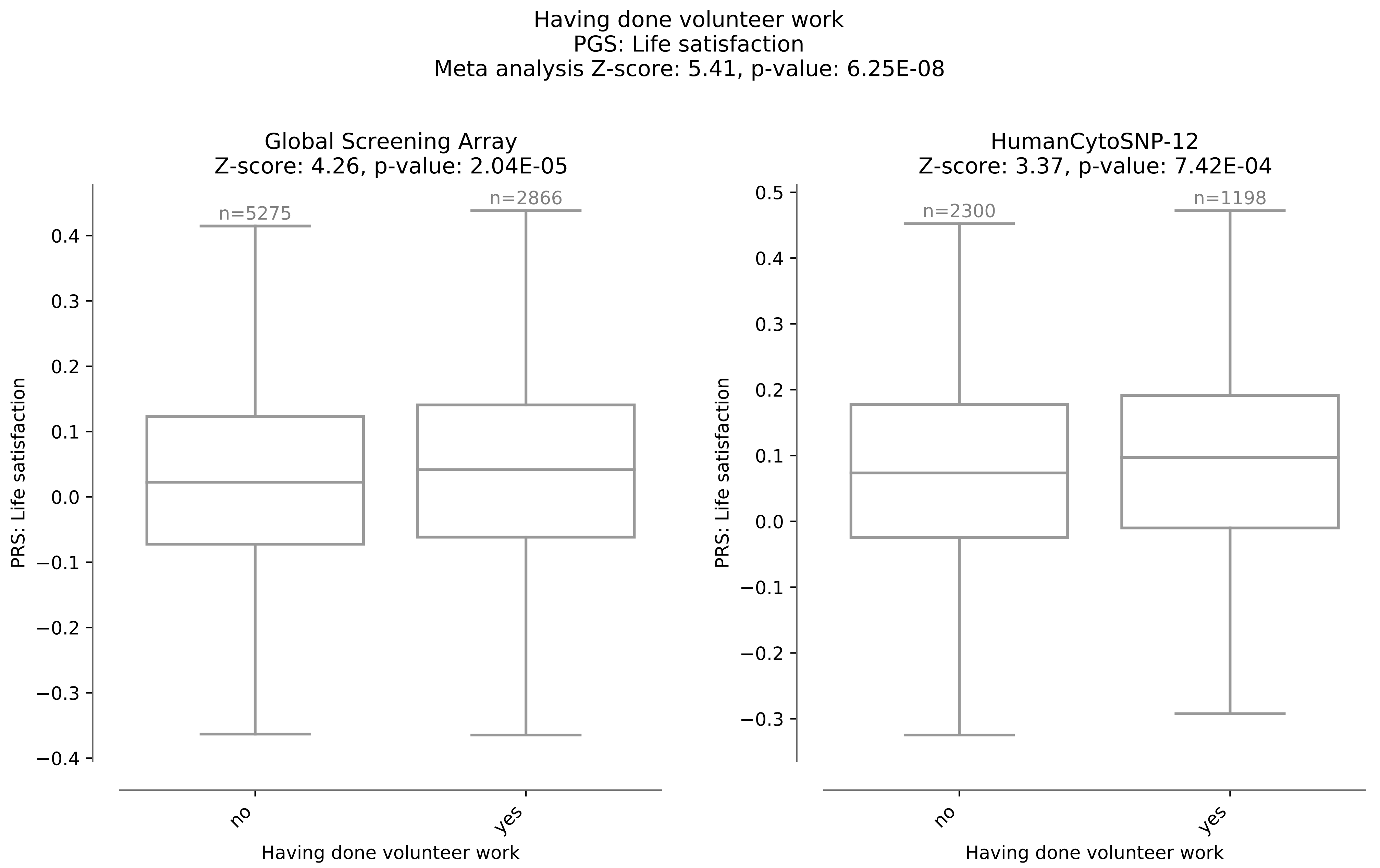

Having done volunteer work
PGS: Life satisfaction
Meta analysis Z-score: 5.41, p-value: 6.25E-08
Global Screening Array
Z-score: 4.26, p-value: 2.04E-05
HumanCytoSNP-12
Z-score: 3.37, p-value: 7.42E-04
n=5275
n=2866
n=2300
n=1198
PRS: Life satisfaction
0.5
0.4
0.3
0.2
0.1
0.0
−0.1
−0.2
−0.3
−0.4
no
yes
Having done volunteer work

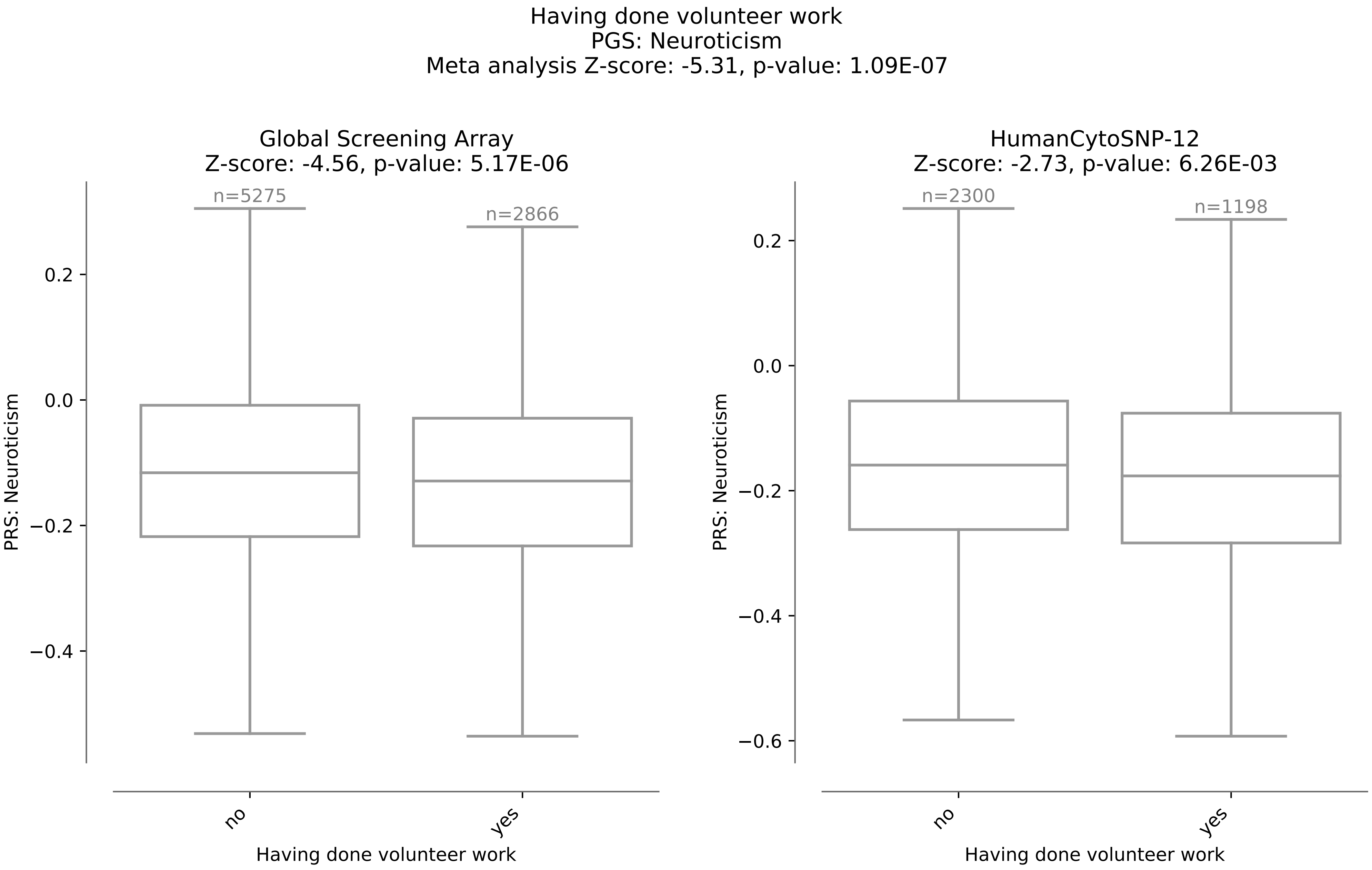

Having done volunteer work
PGS: Neuroticism
Meta analysis Z-score: -5.31, p-value: 1.09E-07
Global Screening Array
Z-score: -4.56, p-value: 5.17E-06
n=5275
n=2866
0.2
0.0
−0.2
−0.4
PRS: Neuroticism
no
yes
Having done volunteer work
HumanCytoSNP-12
Z-score: -2.73, p-value: 6.26E-03
n=2300
n=1198
0.2
0.0
−0.2
−0.4
−0.6
PRS: Neuroticism
no
yes
Having done volunteer work

Had dizziness
PGS: Depression
Meta analysis Z-score: 4.69, p-value: 2.72E-06

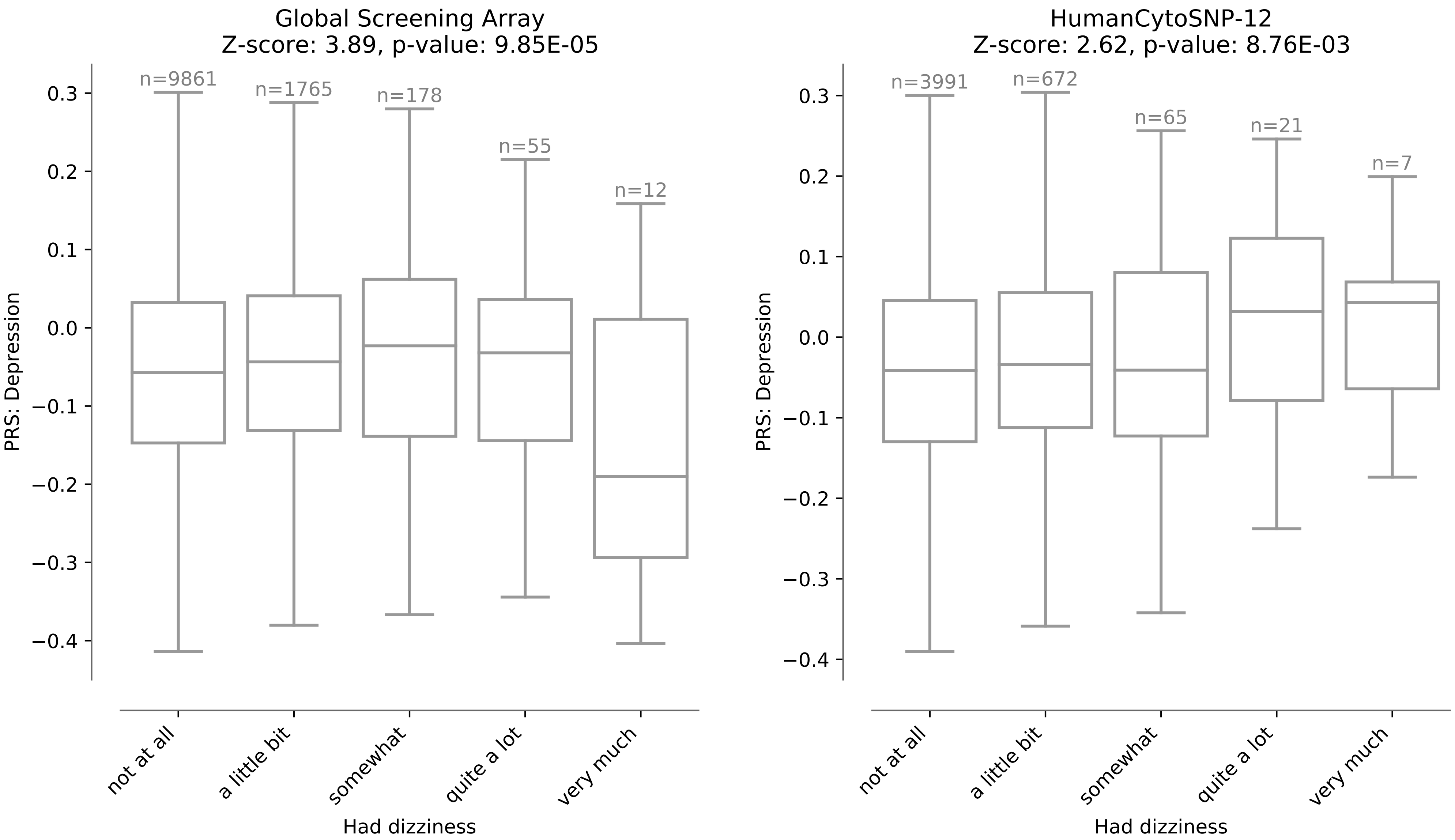

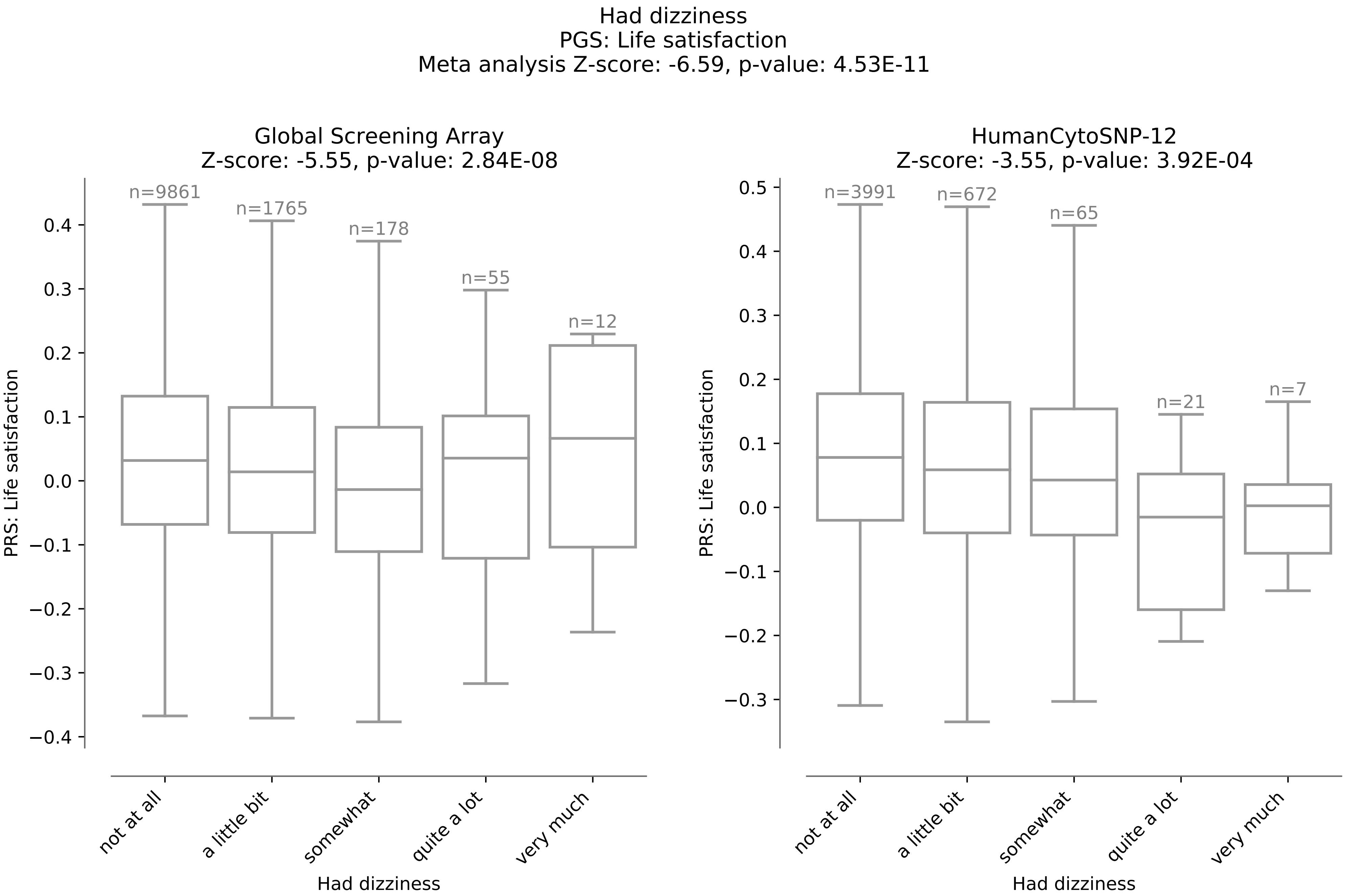
Had dizziness
PGS: Life satisfaction
Meta analysis Z-score: -6.59, p-value: 4.53E-11
Global Screening Array
Z-score: -5.55, p-value: 2.84E-08
n=9861
n=1765
n=178
n=55
n=12
PRS: Life satisfaction
Had dizziness
not at all
a little bit
somewhat
quite a lot
very much
HumanCytoSNP-12
Z-score: -3.55, p-value: 3.92E-04
n=3991
n=672
n=65
n=21
n=7

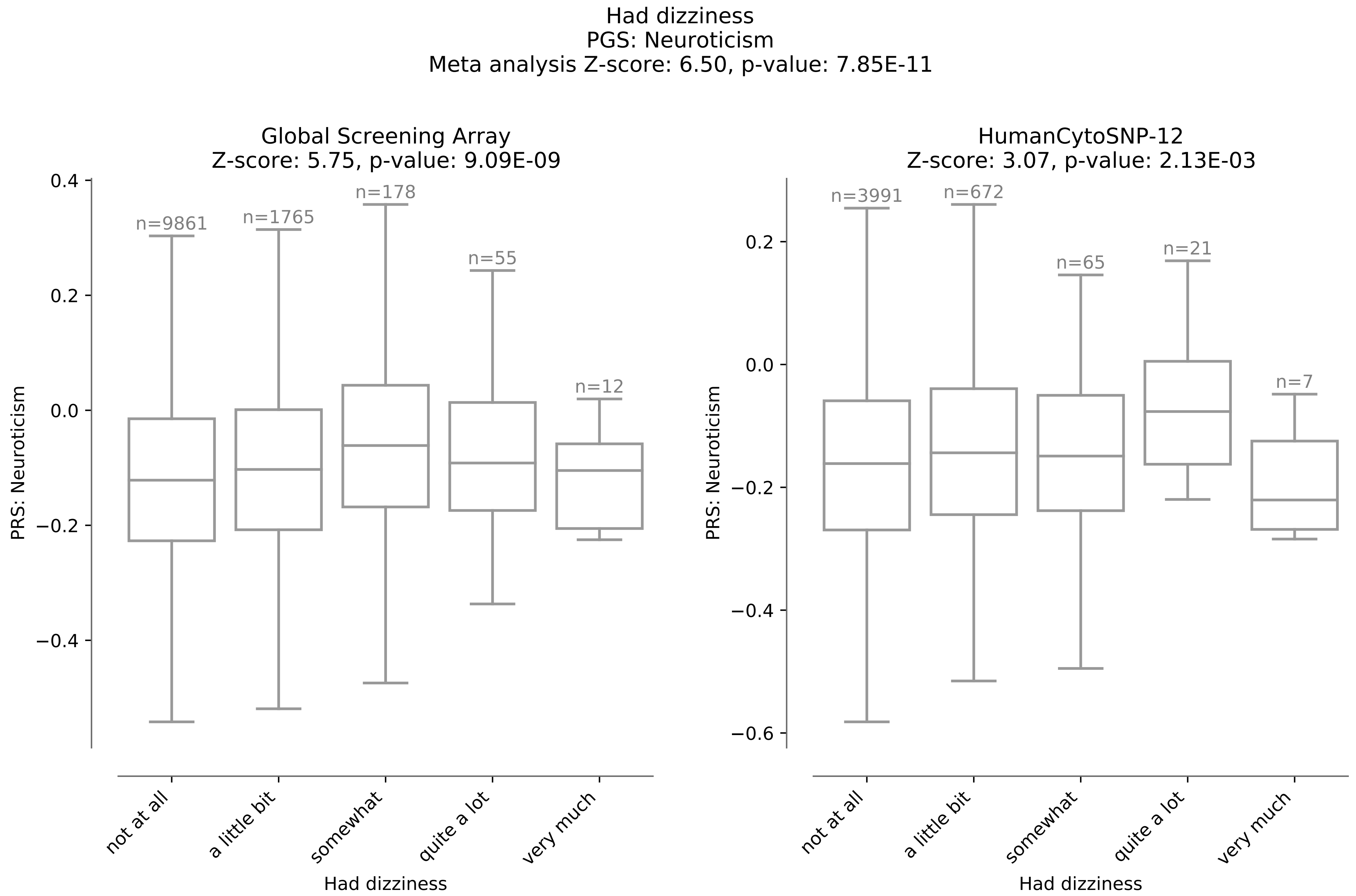

Had dizziness
PGS: Neuroticism
Meta analysis Z-score: 6.50, p-value: 7.85E-11
Global Screening Array
Z-score: 5.75, p-value: 9.09E-09
HumanCytoSNP-12
Z-score: 3.07, p-value: 2.13E-03
PRS: Neuroticism
n=9861
n=1765
n=178
n=55
n=12
n=3991
n=672
n=65
n=21
n=7
0.4
0.2
0.0
−0.2
−0.4
−0.6
not at all
a little bit
somewhat
quite a lot
very much
Had dizziness

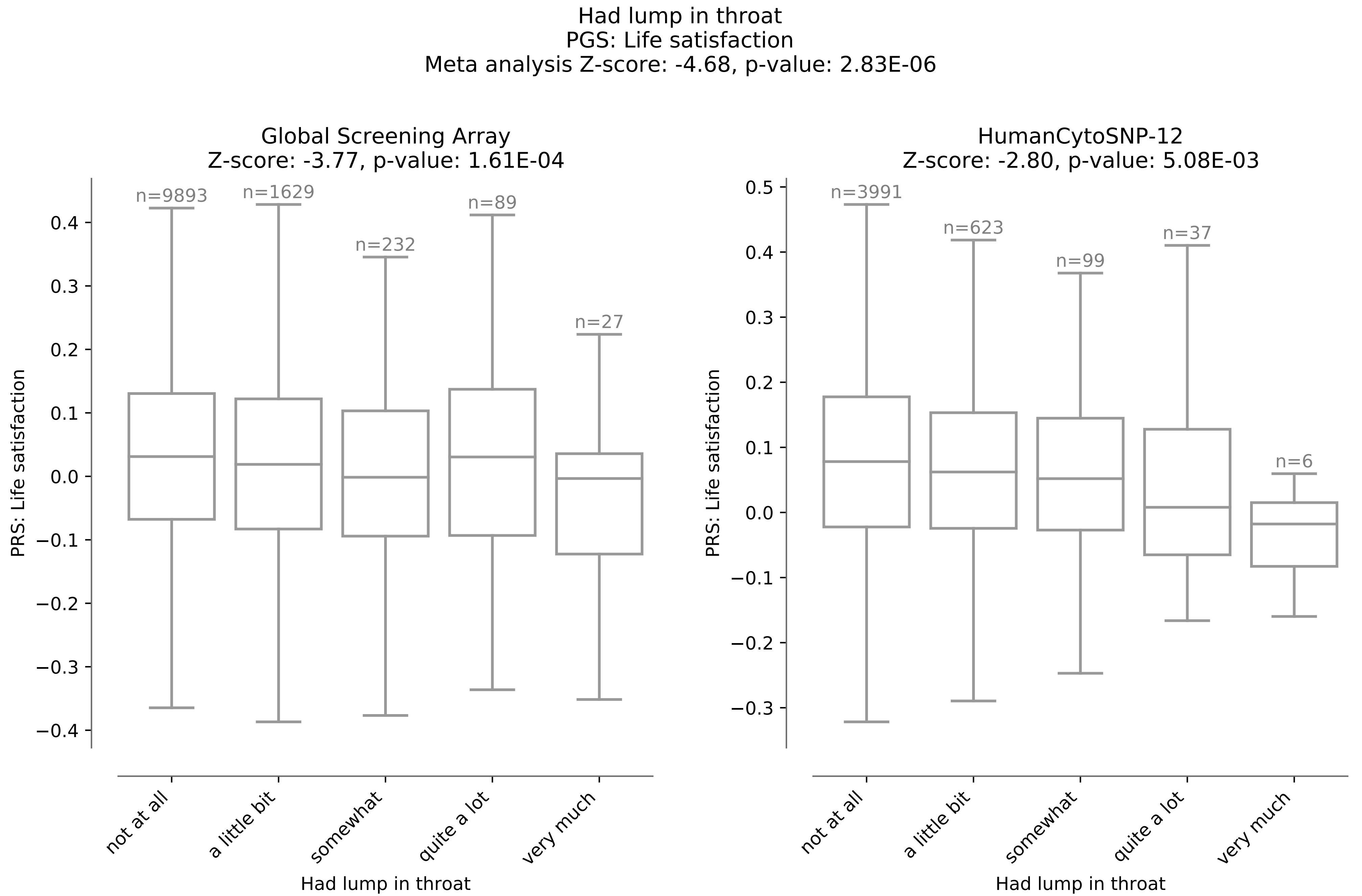
Had lump in throat
PGS: Life satisfaction
Meta analysis Z-score: -4.68, p-value: 2.83E-06
Global Screening Array
Z-score: -3.77, p-value: 1.61E-04
HumanCytoSNP-12
Z-score: -2.80, p-value: 5.08E-03
PRS: Life satisfaction
n=9893
n=1629
n=232
n=89
n=27
n=3991
n=623
n=99
n=37
n=6
not at all
a little bit
somewhat
quite a lot
very much
Had lump in throat

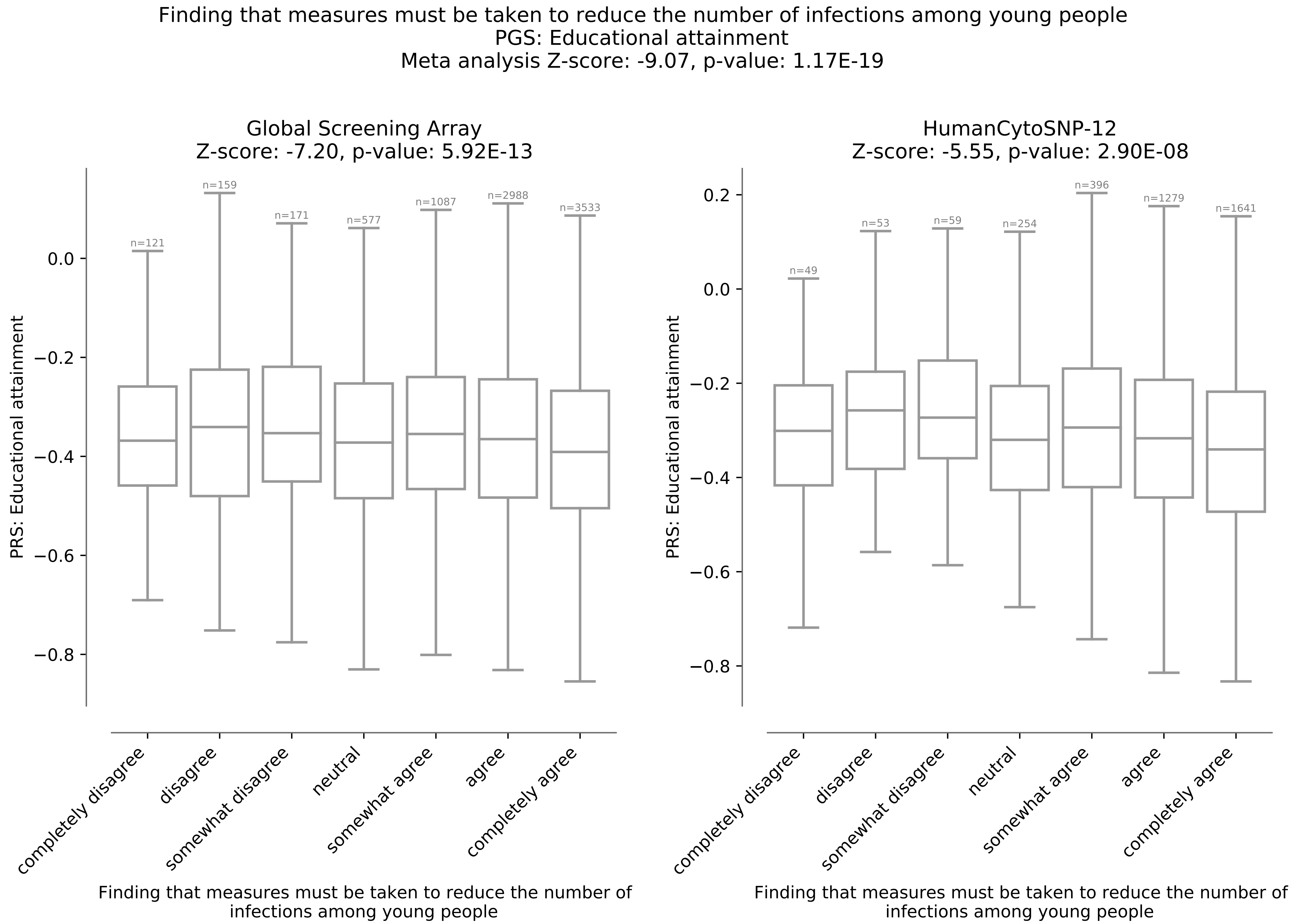

Finding that measures must be taken to reduce the number of infections among young people
PGS: Educational attainment
Meta analysis Z-score: -9.07, p-value: 1.17E-19
Global Screening Array
Z-score: -7.20, p-value: 5.92E-13
HumanCytoSNP-12
Z-score: -5.55, p-value: 2.90E-08
PRS: Educational attainment
n=121
n=159
n=171
n=577
n=1087
n=2988
n=3533
n=49
n=53
n=59
n=254
n=396
n=1279
n=1641
0.0
−0.2
−0.4
−0.6
−0.8
0.2
completely disagree
disagree
somewhat disagree
neutral
somewhat agree
agree
completely agree
Finding that measures must be taken to reduce the number of infections among young people

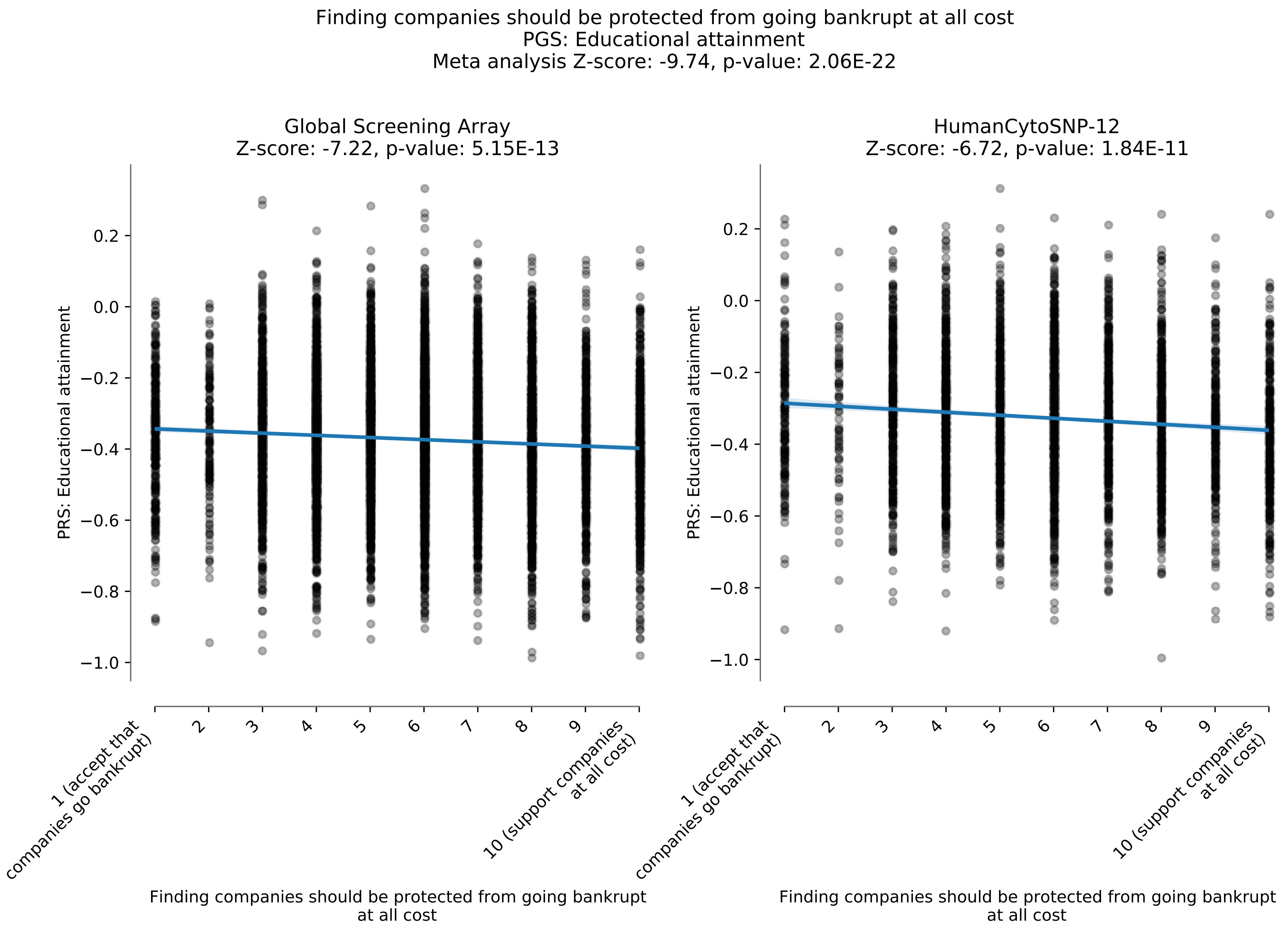
Finding companies should be protected from going bankrupt at all cost
PGS: Educational attainment
Meta analysis Z-score: -9.74, p-value: 2.06E-22
Global Screening Array
Z-score: -7.22, p-value: 5.15E-13
HumanCytoSNP-12
Z-score: -6.72, p-value: 1.84E-11
PRS: Educational attainment
0.2
0.0
−0.2
−0.4
−0.6
−0.8
−1.0
1 (accept that companies go bankrupt)
2
3
4
5
6
7
8
9
10 (support companies at all cost)
Finding companies should be protected from going bankrupt at all cost

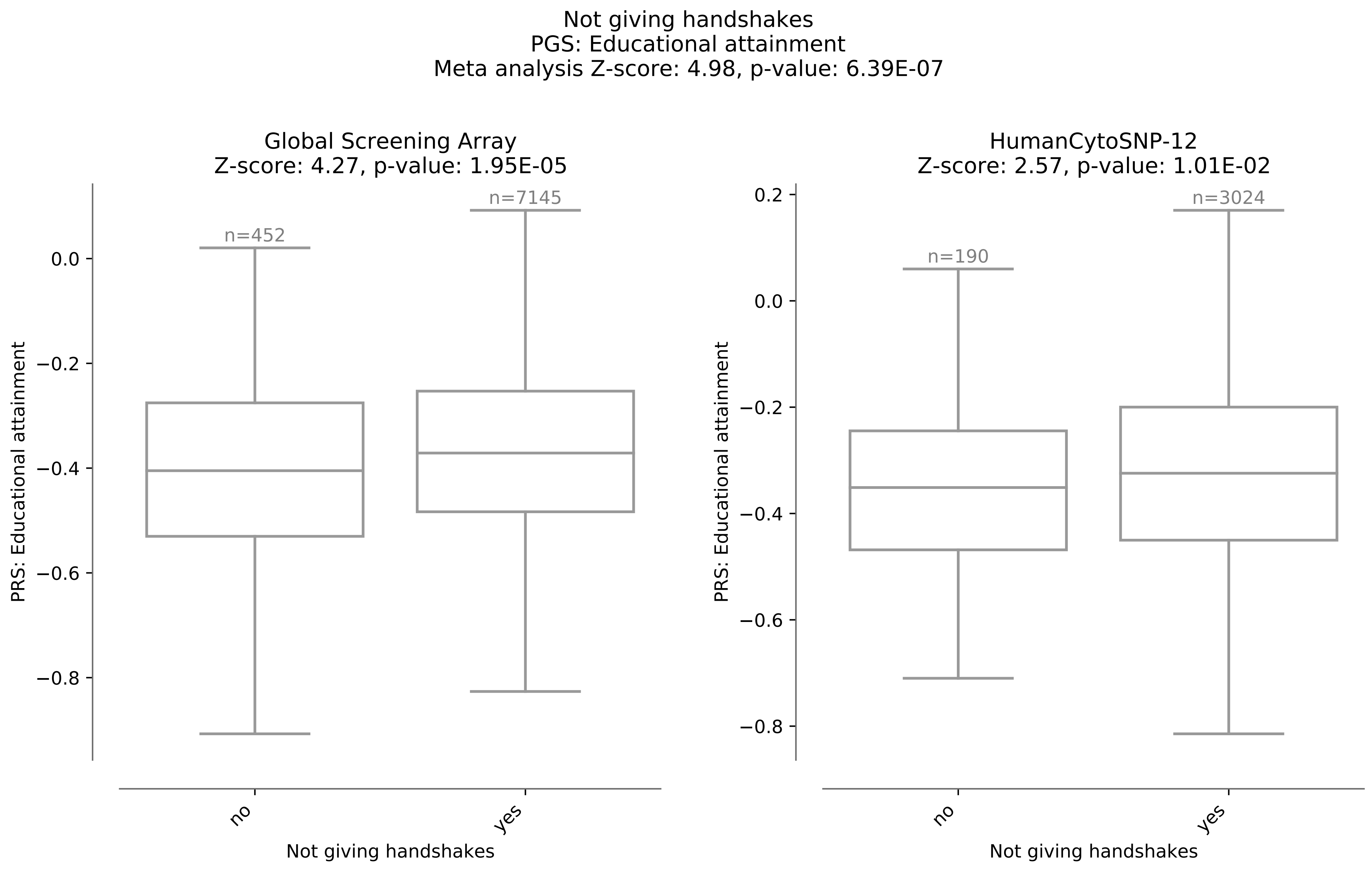
Not giving handshakes
PGS: Educational attainment
Meta analysis Z-score: 4.98, p-value: 6.39E-07
Global Screening Array
Z-score: 4.27, p-value: 1.95E-05
HumanCytoSNP-12
Z-score: 2.57, p-value: 1.01E-02
n=452
n=7145
n=190
n=3024
PRS: Educational attainment
0.2
0.0
−0.2
−0.4
−0.6
−0.8
no
yes
Not giving handshakes

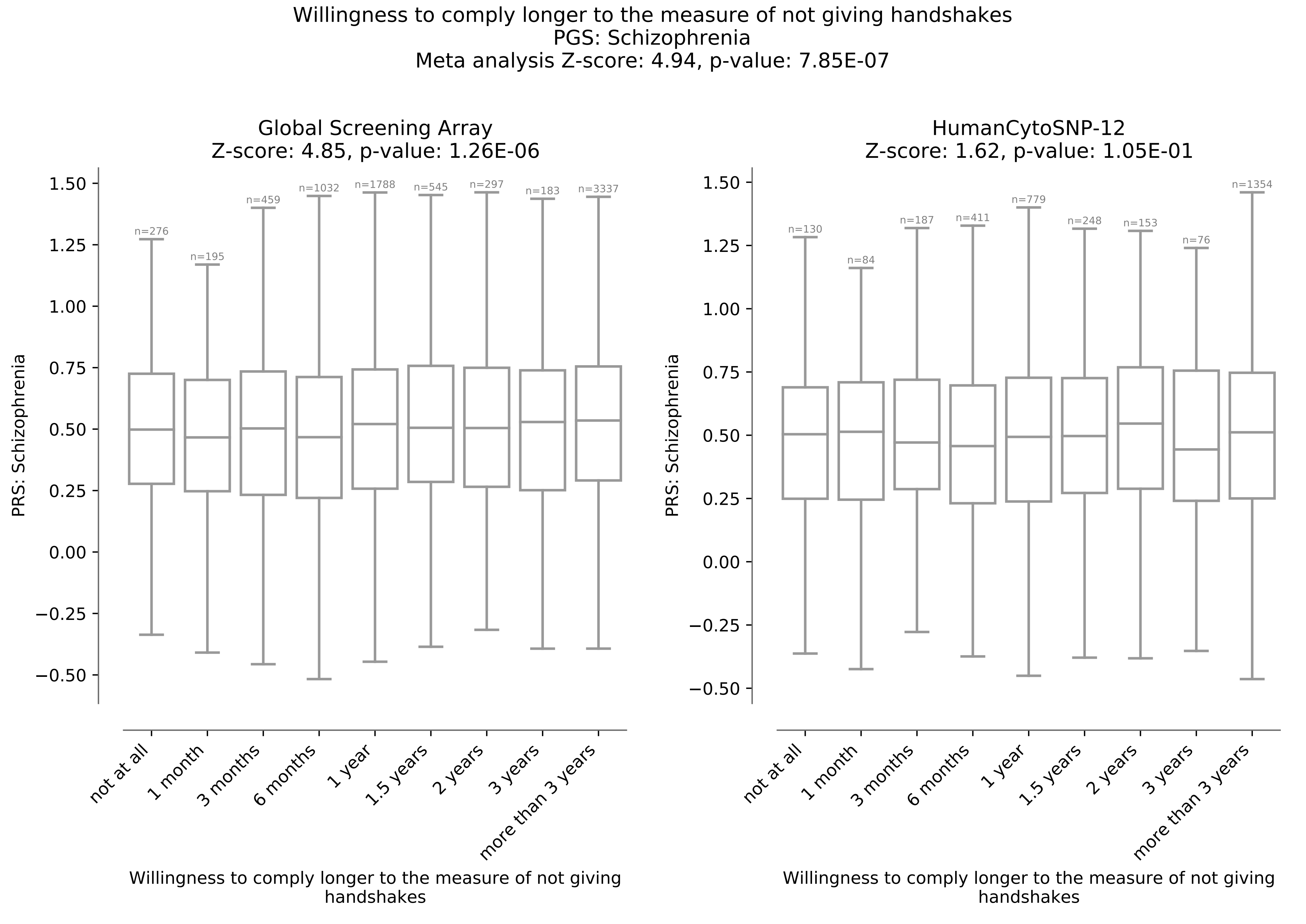
Willingness to comply longer to the measure of not giving handshakes
PGS: Schizophrenia
Meta analysis Z-score: 4.94, p-value: 7.85E-07
Global Screening Array
Z-score: 4.85, p-value: 1.26E-06
HumanCytoSNP-12
Z-score: 1.62, p-value: 1.05E-01
PRS: Schizophrenia
n=276
n=195
n=459
n=1032
n=1788
n=545
n=297
n=183
n=3337
n=130
n=84
n=187
n=411
n=779
n=248
n=153
n=76
n=1354
1.50
1.25
1.00
0.75
0.50
0.25
0.00
−0.25
−0.50
not at all
1 month
3 months
6 months
1 year
1.5 years
2 years
3 years
more than 3 years
Willingness to comply longer to the measure of not giving handshakes

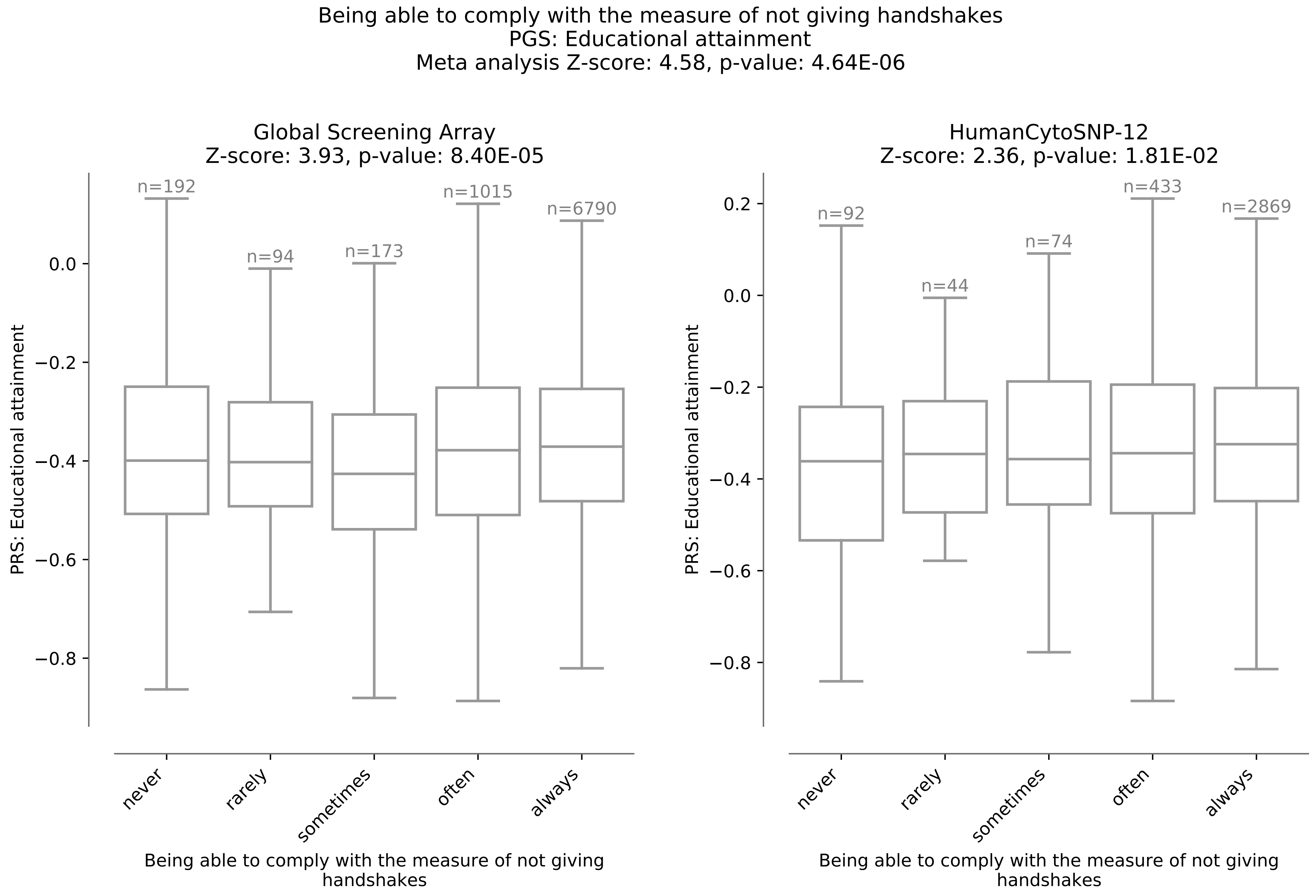

Being able to comply with the measure of not giving handshakes
PGS: Educational attainment
Meta analysis Z-score: 4.58, p-value: 4.64E-06
Global Screening Array
Z-score: 3.93, p-value: 8.40E-05
HumanCytoSNP-12
Z-score: 2.36, p-value: 1.81E-02
PRS: Educational attainment
n=192
n=94
n=173
n=1015
n=6790
n=92
n=44
n=74
n=433
n=2869
0.2
0.0
−0.2
−0.4
−0.6
−0.8
never
rarely
sometimes
often
always
Being able to comply with the measure of not giving handshakes

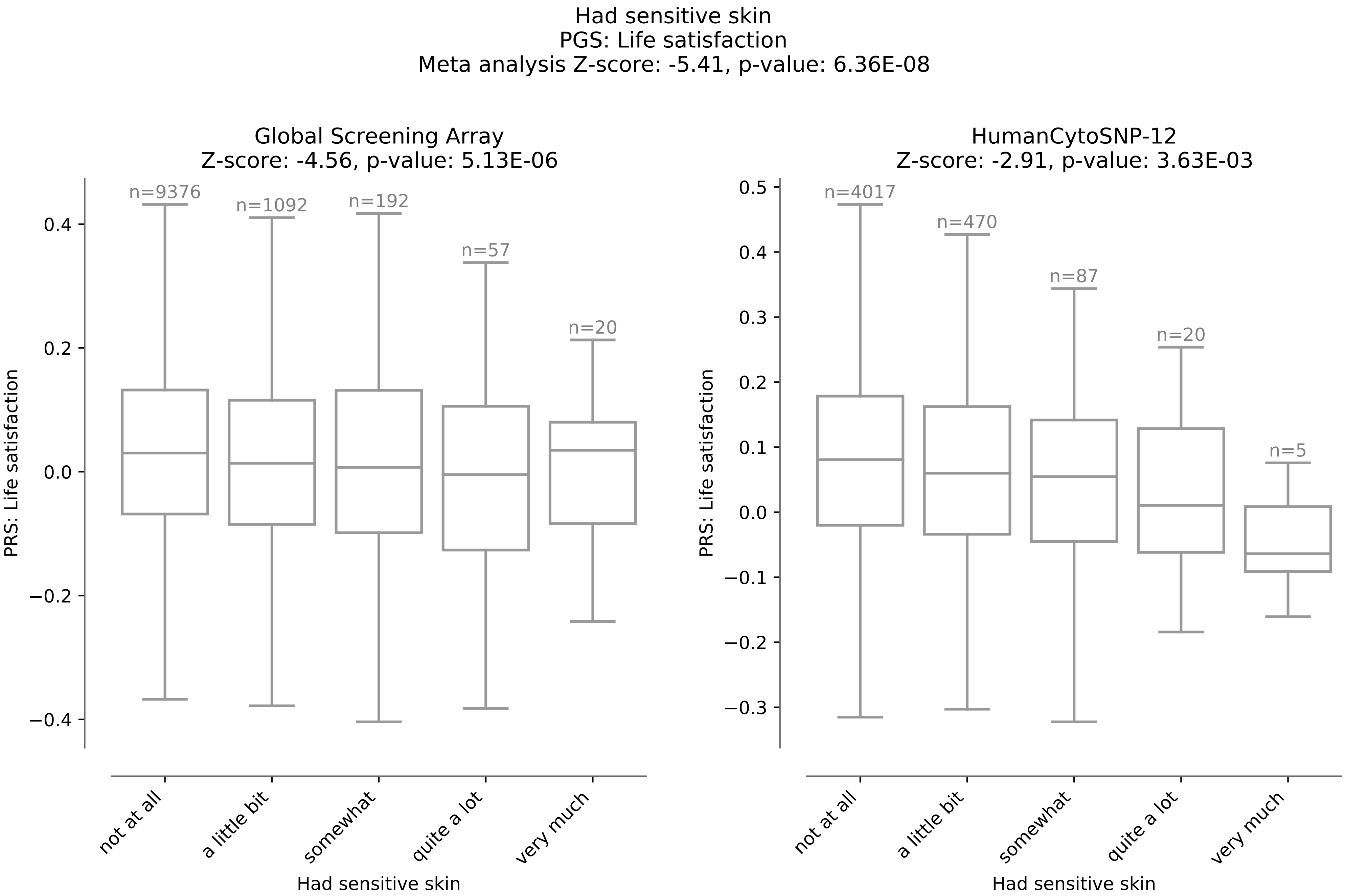

Had sensitive skin
PGS: Life satisfaction
Meta analysis Z-score: -5.41, p-value: 6.36E-08
Global Screening Array
Z-score: -4.56, p-value: 5.13E-06
n=9376
n=1092
n=192
n=57
n=20
0.4
0.2
0.0
−0.2
−0.4
PRS: Life satisfaction
not at all
a little bit
somewhat
quite a lot
very much
Had sensitive skin
HumanCytoSNP-12
Z-score: -2.91, p-value: 3.63E-03
n=4017
n=470
n=87
n=20
n=5
0.5
0.4
0.3
0.2
0.1
0.0
−0.1
−0.2
−0.3
PRS: Life satisfaction
not at all
a little bit
somewhat
quite a lot
very much
Had sensitive skin

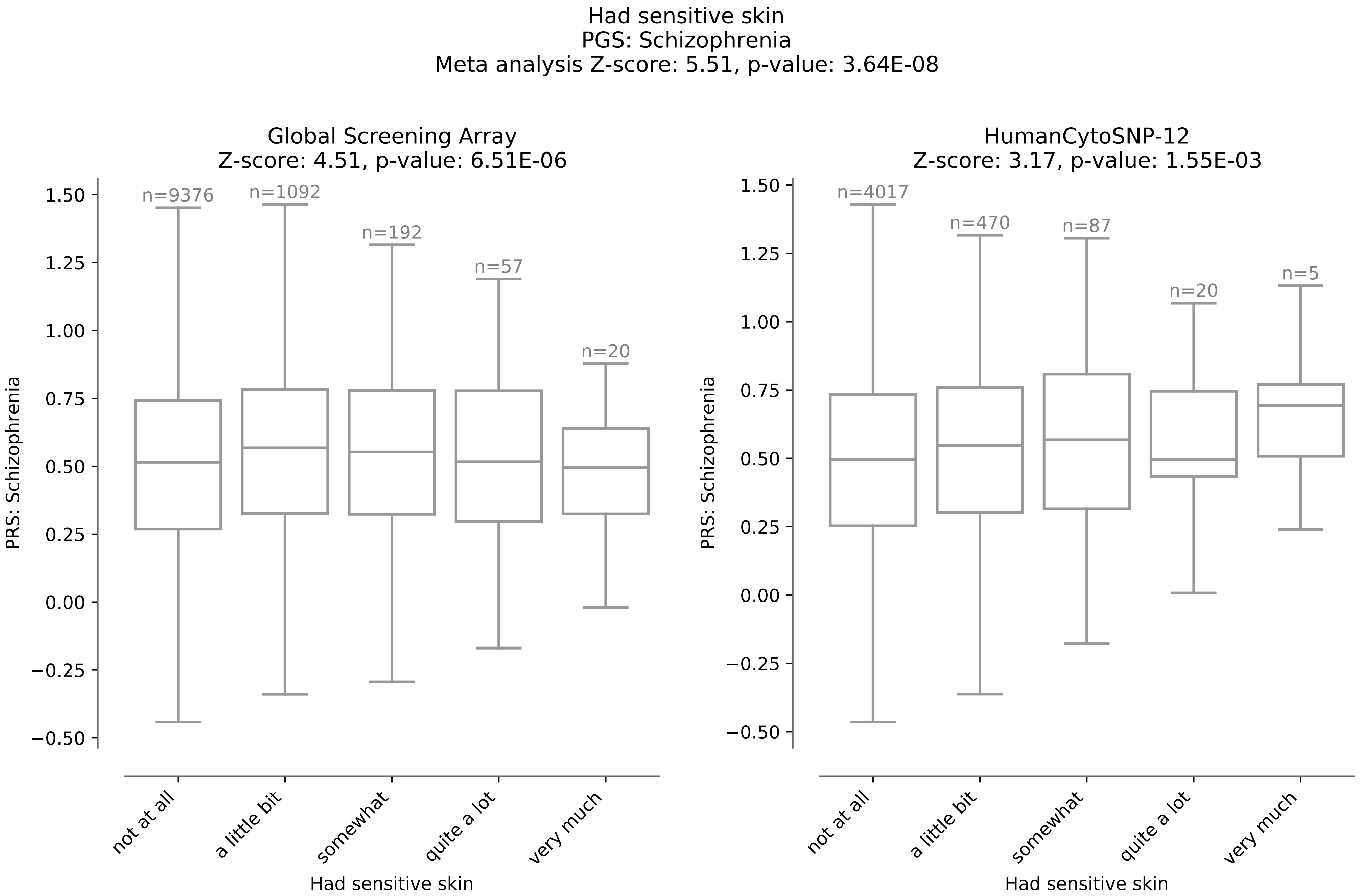
Had sensitive skin
PGS: Schizophrenia
Meta analysis Z-score: 5.51, p-value: 3.64E-08
Global Screening Array
Z-score: 4.51, p-value: 6.51E-06
HumanCytoSNP-12
Z-score: 3.17, p-value: 1.55E-03
PRS: Schizophrenia
Had sensitive skin
not at all
a little bit
somewhat
quite a lot
very much
n=9376
n=1092
n=192
n=57
n=20
n=4017
n=470
n=87
n=20
n=5

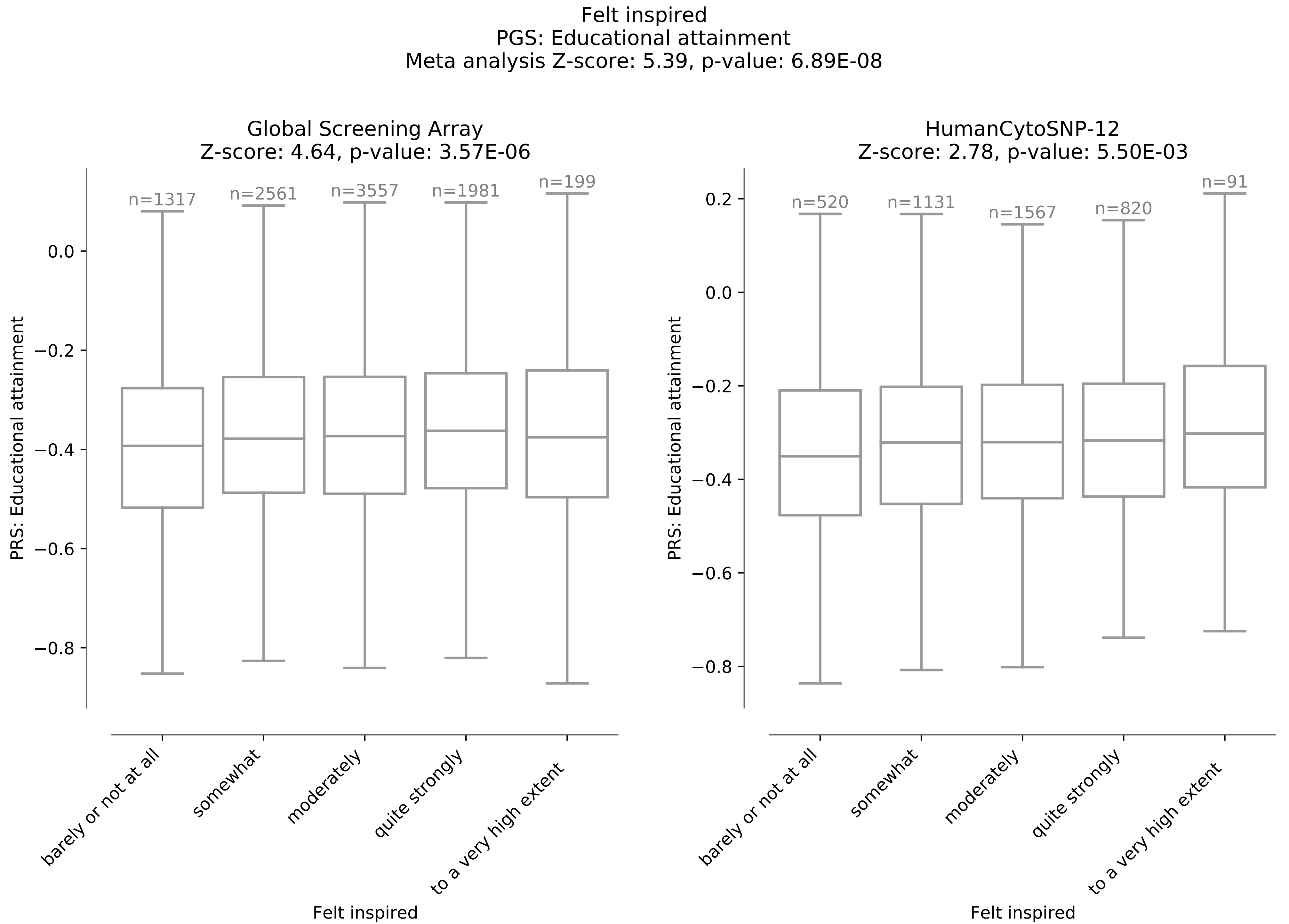
Felt inspired
PGS: Educational attainment
Meta analysis Z-score: 5.39, p-value: 6.89E-08
Global Screening Array
Z-score: 4.64, p-value: 3.57E-06
n=1317
n=2561
n=3557
n=1981
n=199
PRS: Educational attainment
0.0
−0.2
−0.4
−0.6
−0.8
barely or not at all
somewhat
moderately
quite strongly
to a very high extent
Felt inspired
HumanCytoSNP-12
Z-score: 2.78, p-value: 5.50E-03
n=520
n=1131
n=1567
n=820
n=91
PRS: Educational attainment
0.2
0.0
−0.2
−0.4
−0.6
−0.8
barely or not at all
somewhat
moderately
quite strongly
to a very high extent
Felt inspired

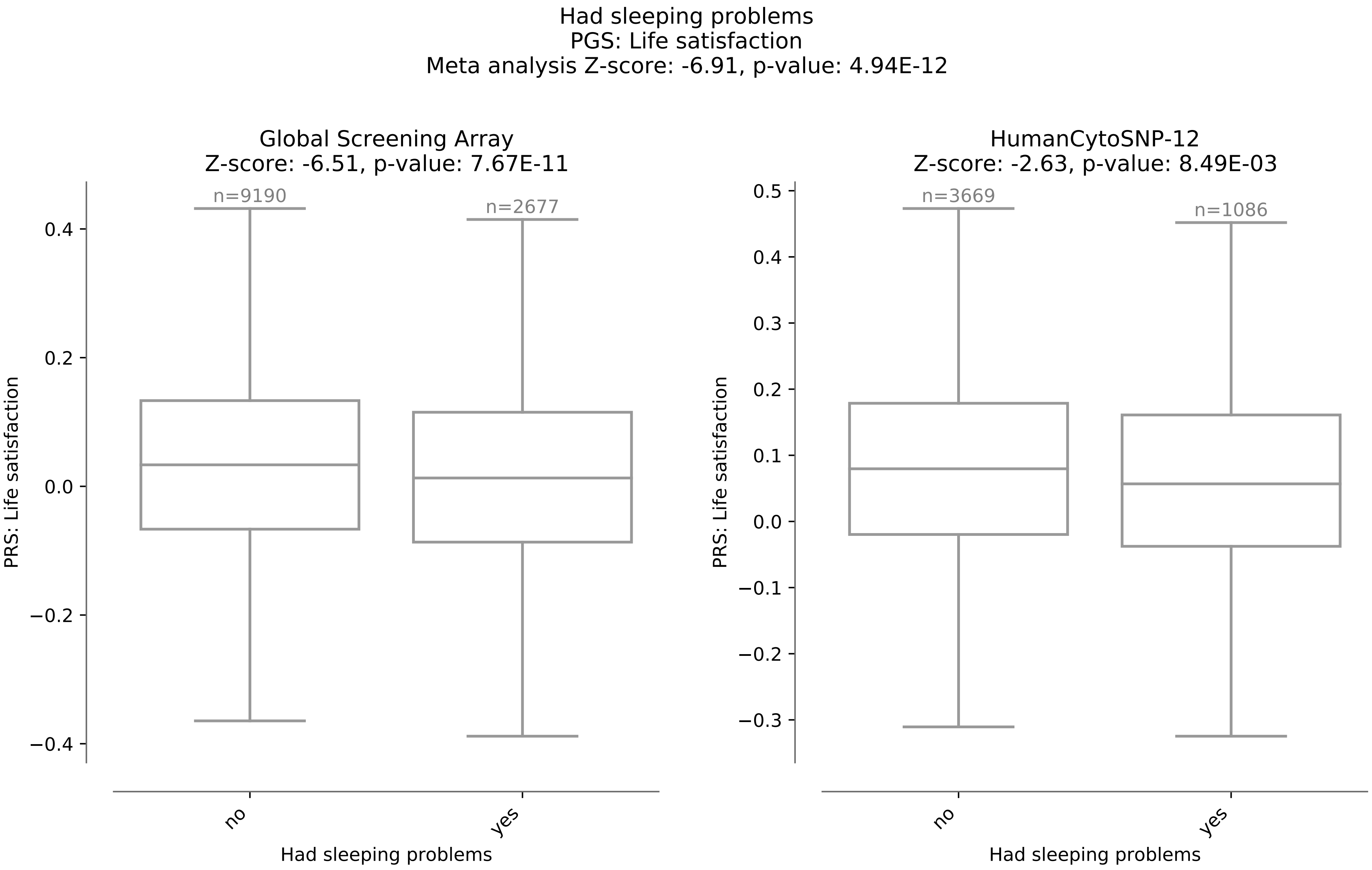
Had sleeping problems
PGS: Life satisfaction
Meta analysis Z-score: -6.91, p-value: 4.94E-12
Global Screening Array
Z-score: -6.51, p-value: 7.67E-11
n=9190
n=2677
PRS: Life satisfaction
0.4
0.2
0.0
−0.2
−0.4
no
yes
Had sleeping problems
HumanCytoSNP-12
Z-score: -2.63, p-value: 8.49E-03
n=3669
n=1086
PRS: Life satisfaction
0.5
0.4
0.3
0.2
0.1
0.0
−0.1
−0.2
−0.3
no
yes
Had sleeping problems

Had sleeping problems
PGS: Neuroticism
Meta analysis Z-score: 5.25, p-value: 1.55E-07

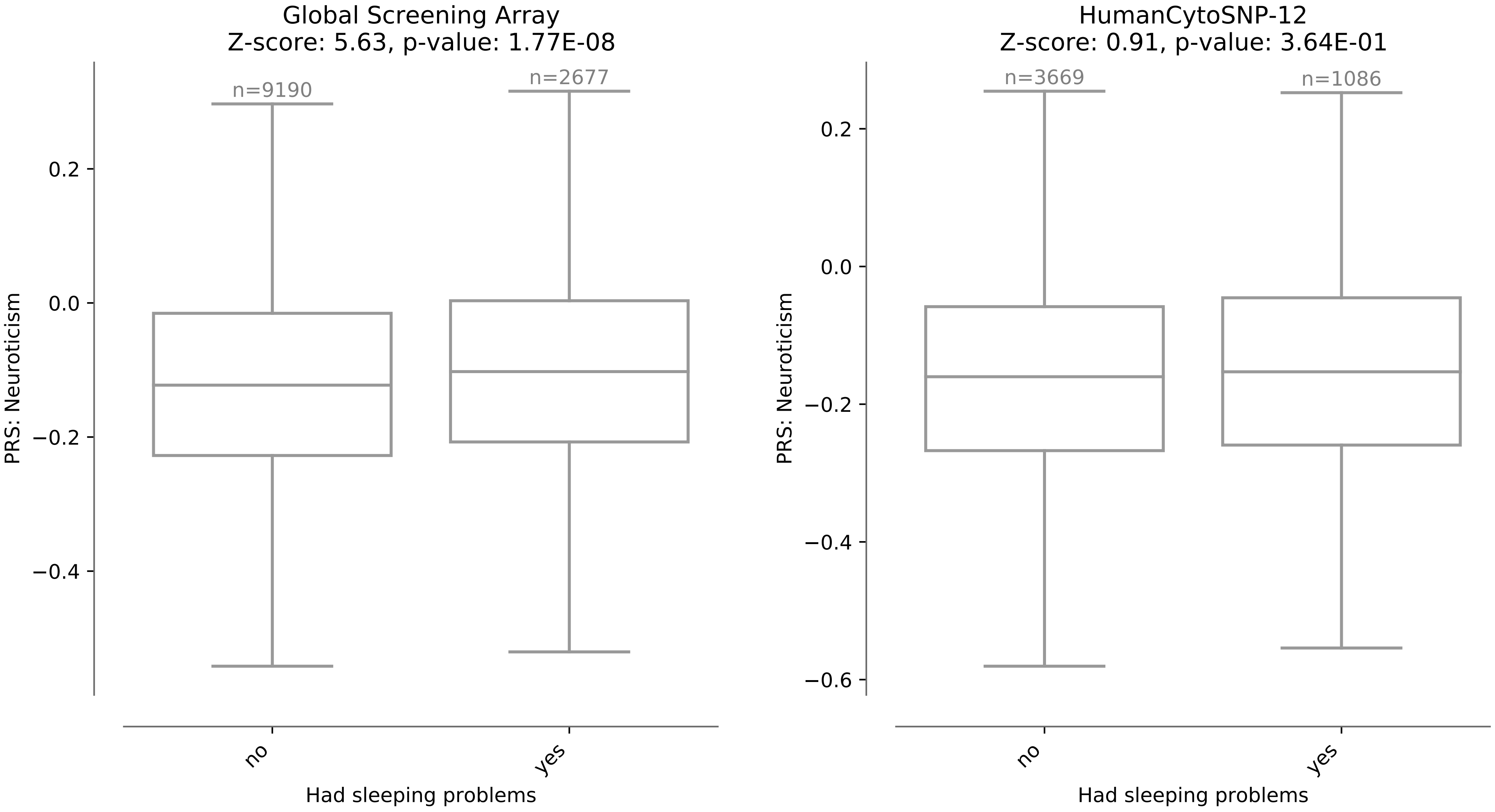

Smoked
PGS: Anxiety/tension
Meta analysis Z-score: -5.85, p-value: 4.87E-09

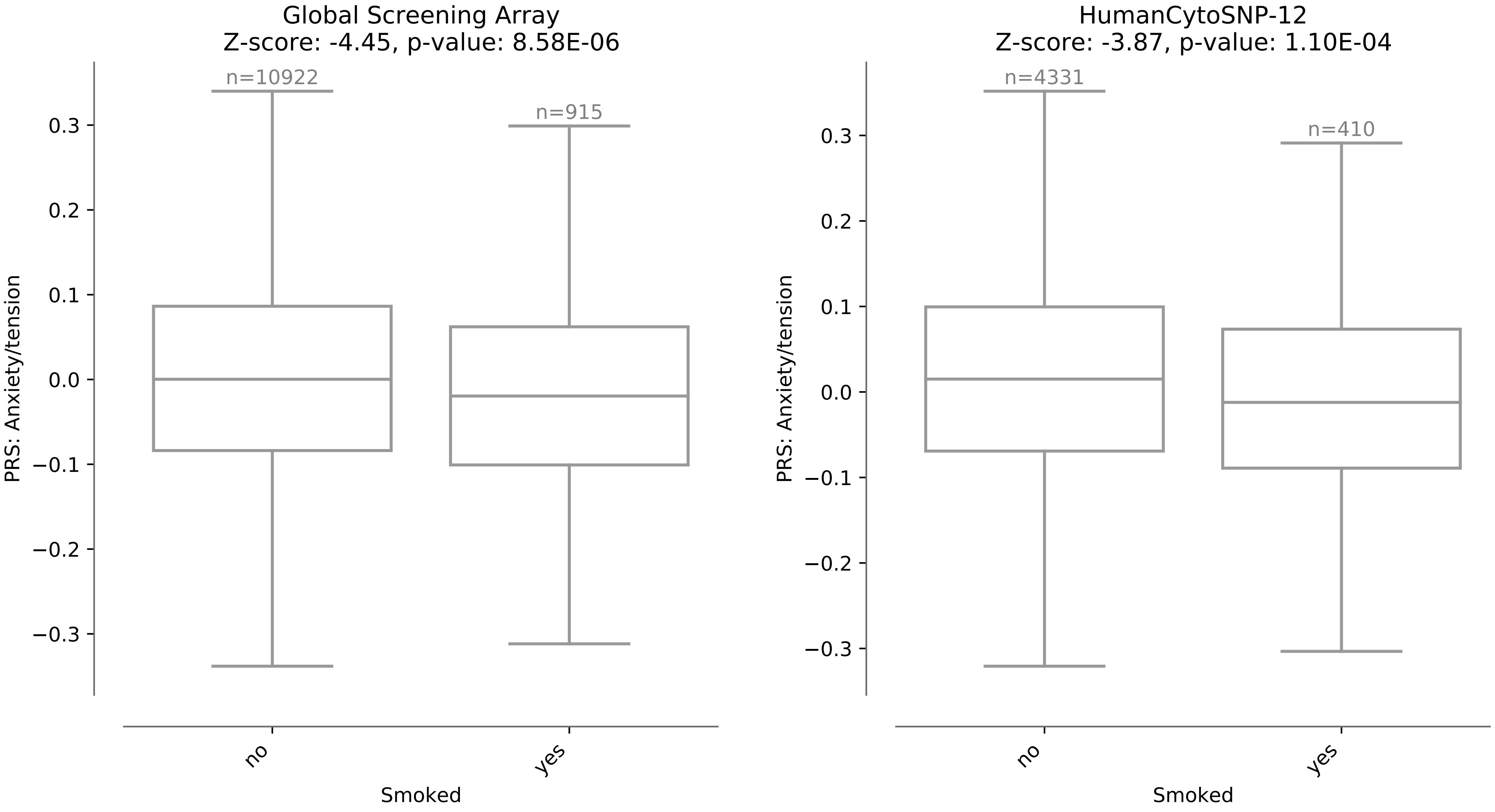

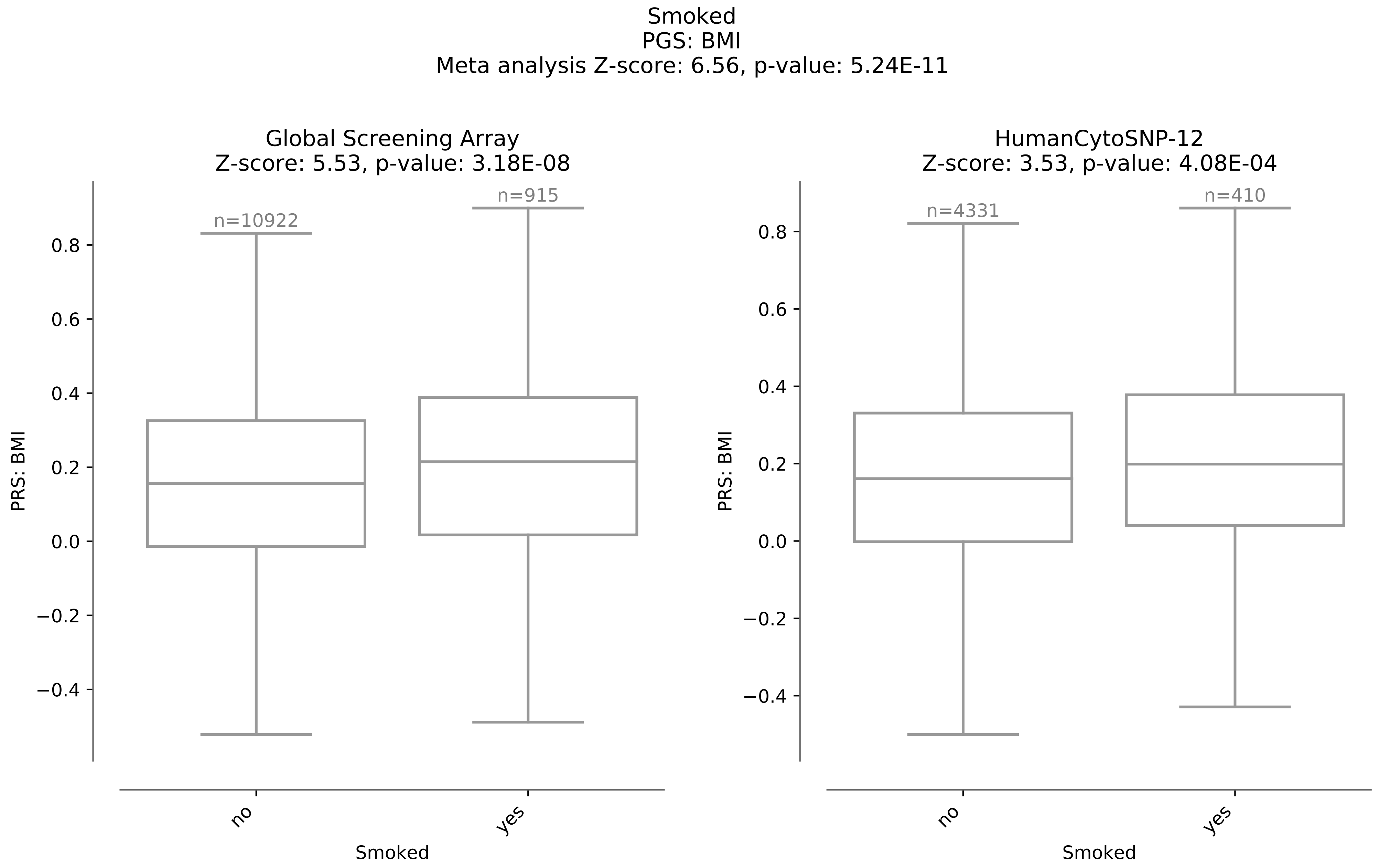

Smoked
PGS: BMI
Meta analysis Z-score: 6.56, p-value: 5.24E-11
Global Screening Array
Z-score: 5.53, p-value: 3.18E-08
HumanCytoSNP-12
Z-score: 3.53, p-value: 4.08E-04
n=10922
n=915
n=4331
n=410
PRS: BMI
0.8
0.6
0.4
0.2
0.0
−0.2
−0.4
no
yes
Smoked

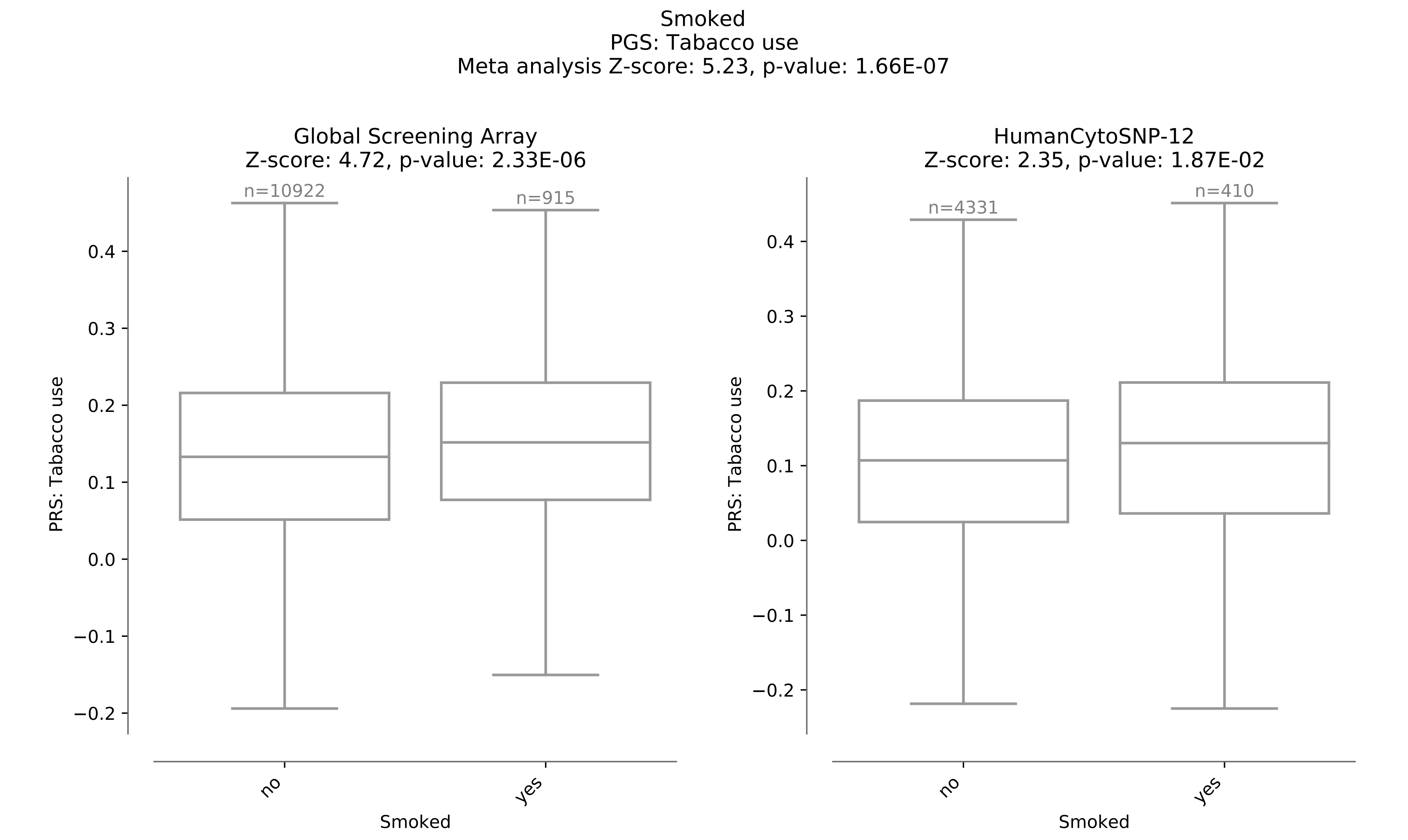
Smoked
PGS: Tabacco use
Meta analysis Z-score: 5.23, p-value: 1.66E-07
Global Screening Array
Z-score: 4.72, p-value: 2.33E-06
n=10922
n=915
PRS: Tabacco use
Smoked
no
yes
HumanCytoSNP-12
Z-score: 2.35, p-value: 1.87E-02
n=4331
n=410
PRS: Tabacco use
Smoked
no
yes

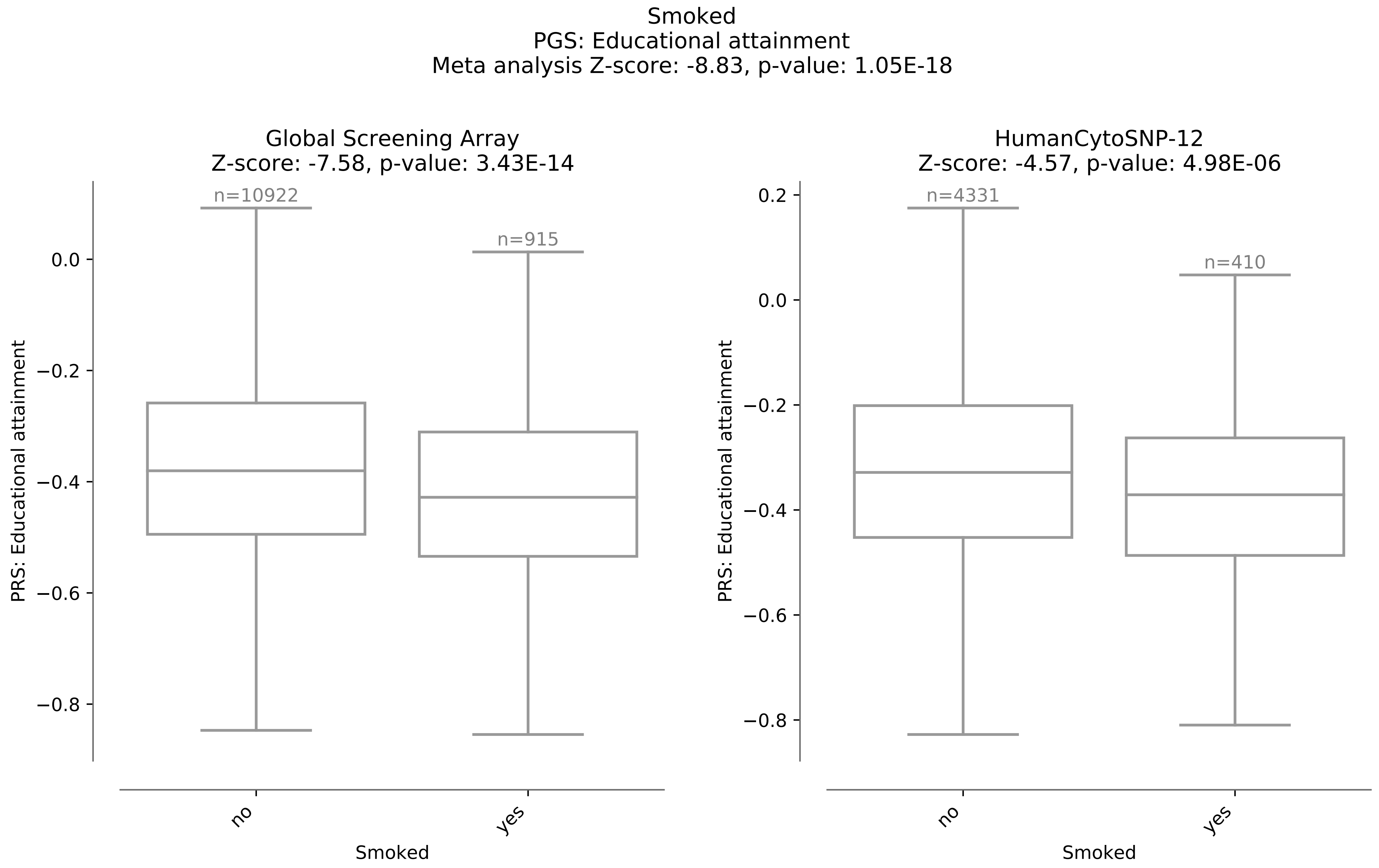
Smoked
PGS: Educational attainment
Meta analysis Z-score: -8.83, p-value: 1.05E-18
Global Screening Array
Z-score: -7.58, p-value: 3.43E-14
n=10922
n=915
PRS: Educational attainment
0.0
−0.2
−0.4
−0.6
−0.8
no
yes
Smoked
HumanCytoSNP-12
Z-score: -4.57, p-value: 4.98E-06
n=4331
n=410
PRS: Educational attainment
0.2
0.0
−0.2
−0.4
−0.6
−0.8
no
yes
Smoked

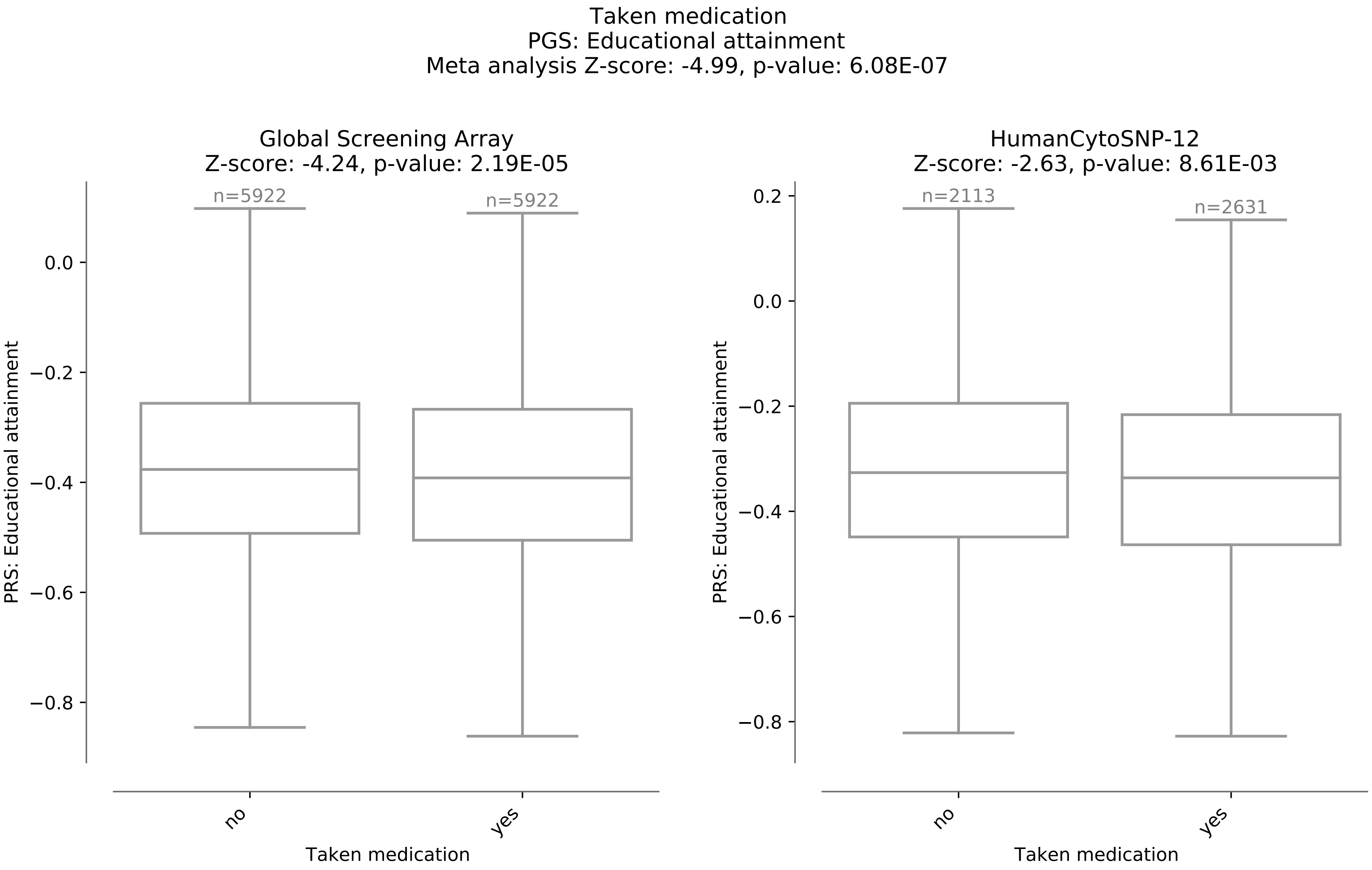

Taken medication
PGS: Educational attainment
Meta analysis Z-score: -4.99, p-value: 6.08E-07
Global Screening Array
Z-score: -4.24, p-value: 2.19E-05
HumanCytoSNP-12
Z-score: -2.63, p-value: 8.61E-03
PRS: Educational attainment
n=5922
n=5922
n=2113
n=2631
0.2
0.0
−0.2
−0.4
−0.6
−0.8
no
yes
Taken medication

Taken medication
PGS: Life satisfaction
Meta analysis Z-score: -4.54, p-value: 5.70E-06

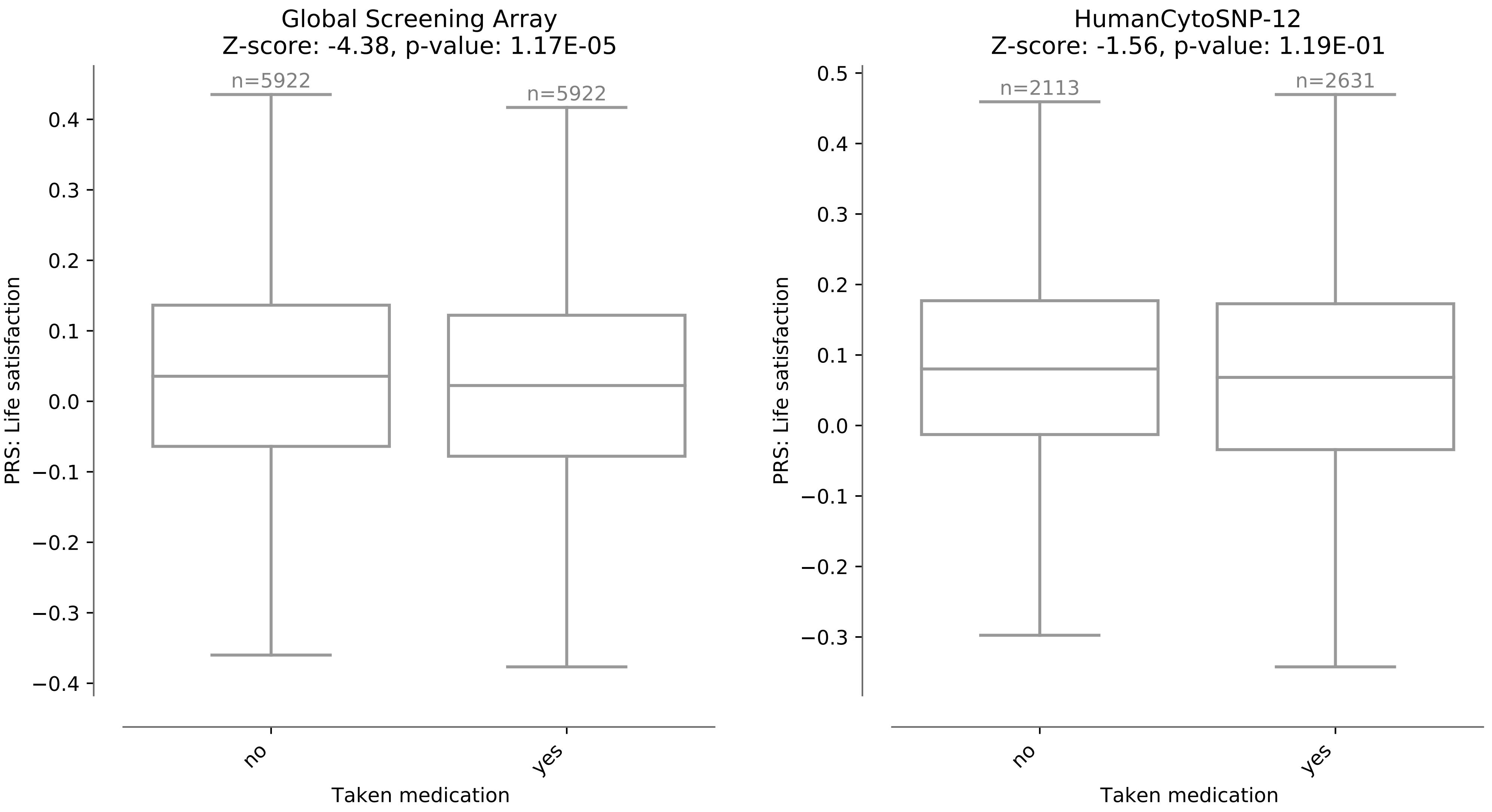

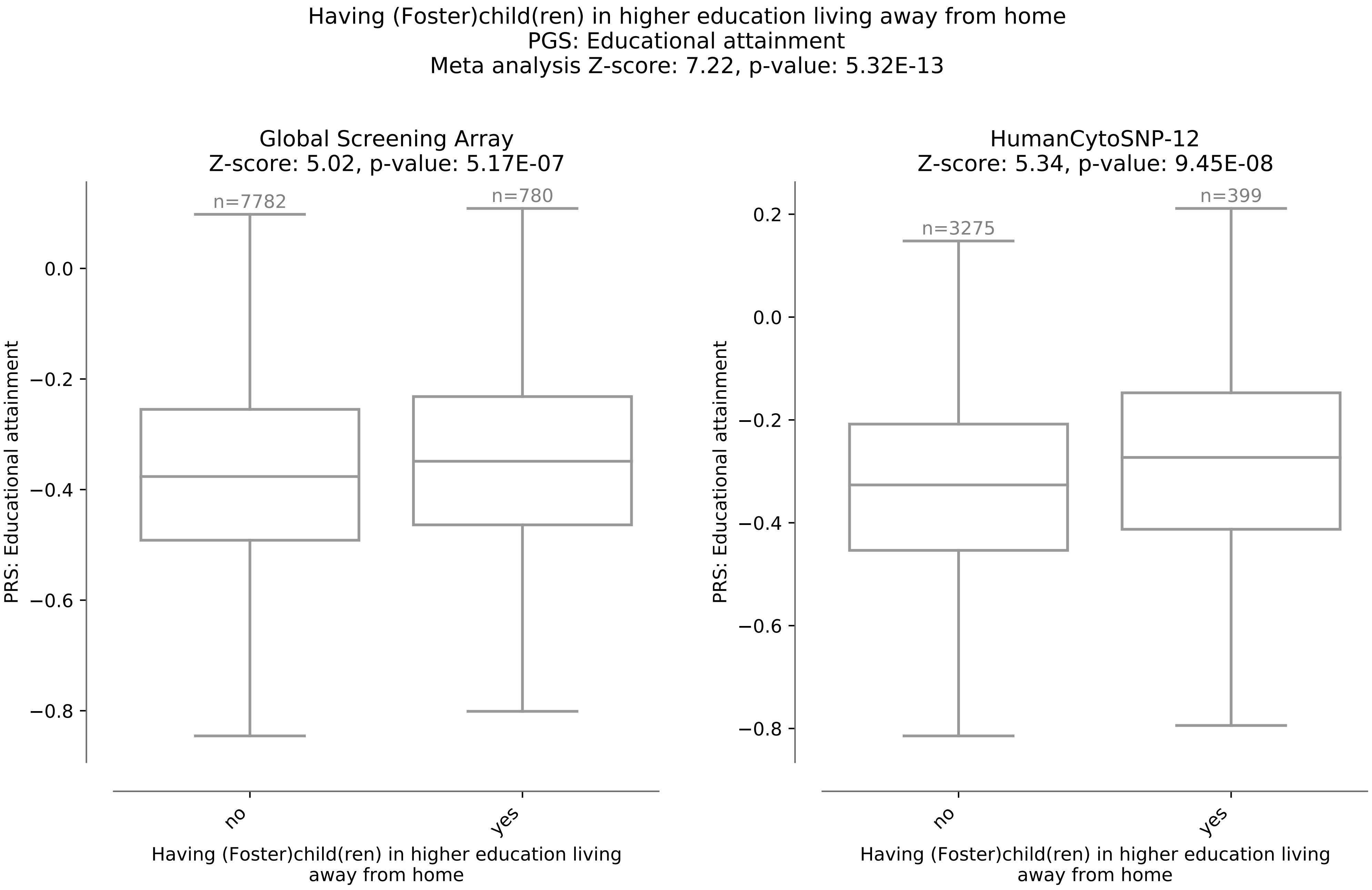

Having (Foster)child(ren) in higher education living away from home
PGS: Educational attainment
Meta analysis Z-score: 7.22, p-value: 5.32E-13
Global Screening Array
Z-score: 5.02, p-value: 5.17E-07
HumanCytoSNP-12
Z-score: 5.34, p-value: 9.45E-08
n=7782
n=780
n=3275
n=399
PRS: Educational attainment
0.2
0.0
−0.2
−0.4
−0.6
−0.8
no
yes
Having (Foster)child(ren) in higher education living away from home

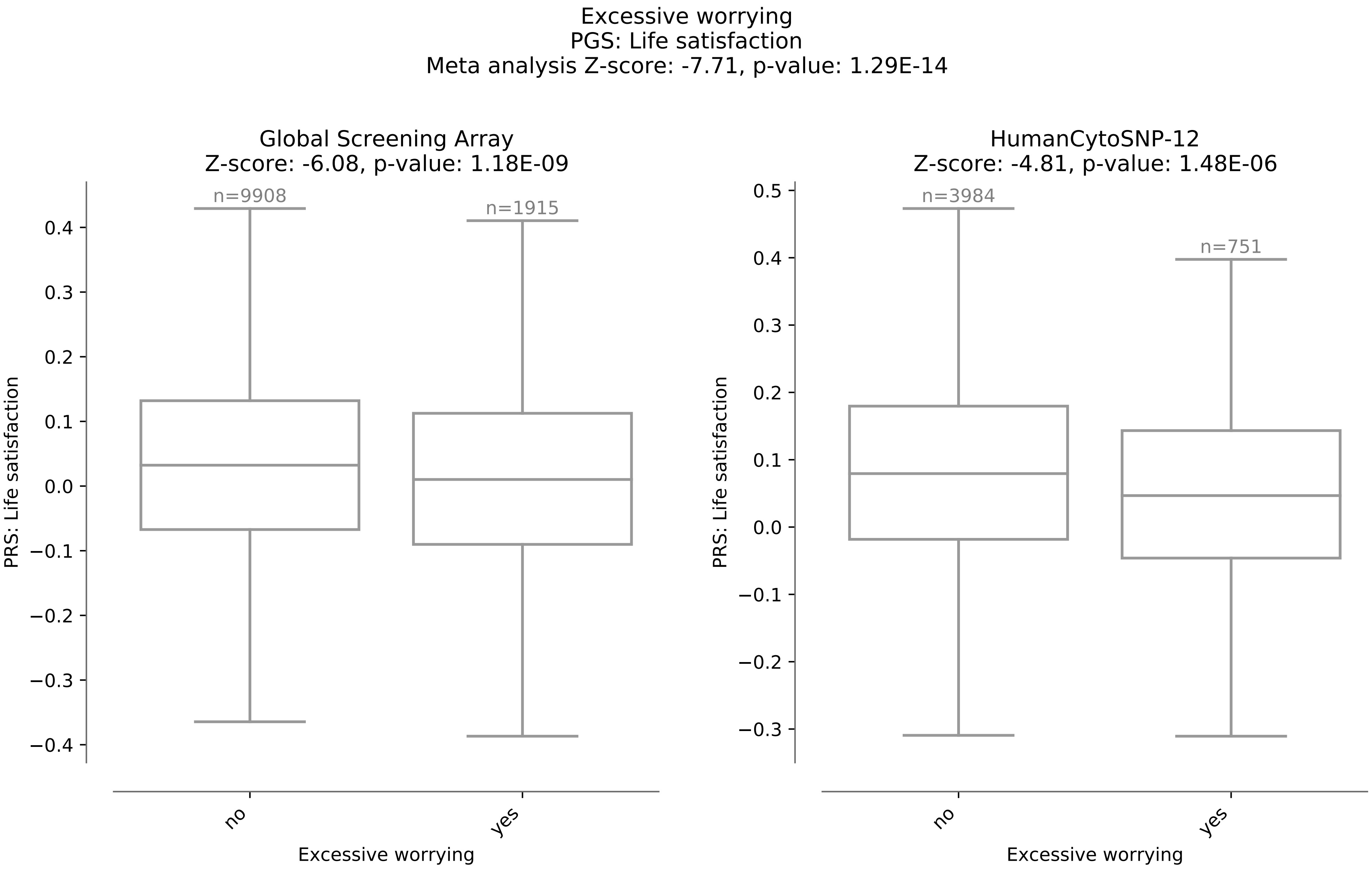
Excessive worrying
PGS: Life satisfaction
Meta analysis Z-score: -7.71, p-value: 1.29E-14
Global Screening Array
Z-score: -6.08, p-value: 1.18E-09
n=9908
n=1915
0.4
0.3
0.2
0.1
0.0
−0.1
−0.2
−0.3
−0.4
PRS: Life satisfaction
no
yes
Excessive worrying
HumanCytoSNP-12
Z-score: -4.81, p-value: 1.48E-06
n=3984
n=751
0.5
0.4
0.3
0.2
0.1
0.0
−0.1
−0.2
−0.3
PRS: Life satisfaction
no
yes
Excessive worrying

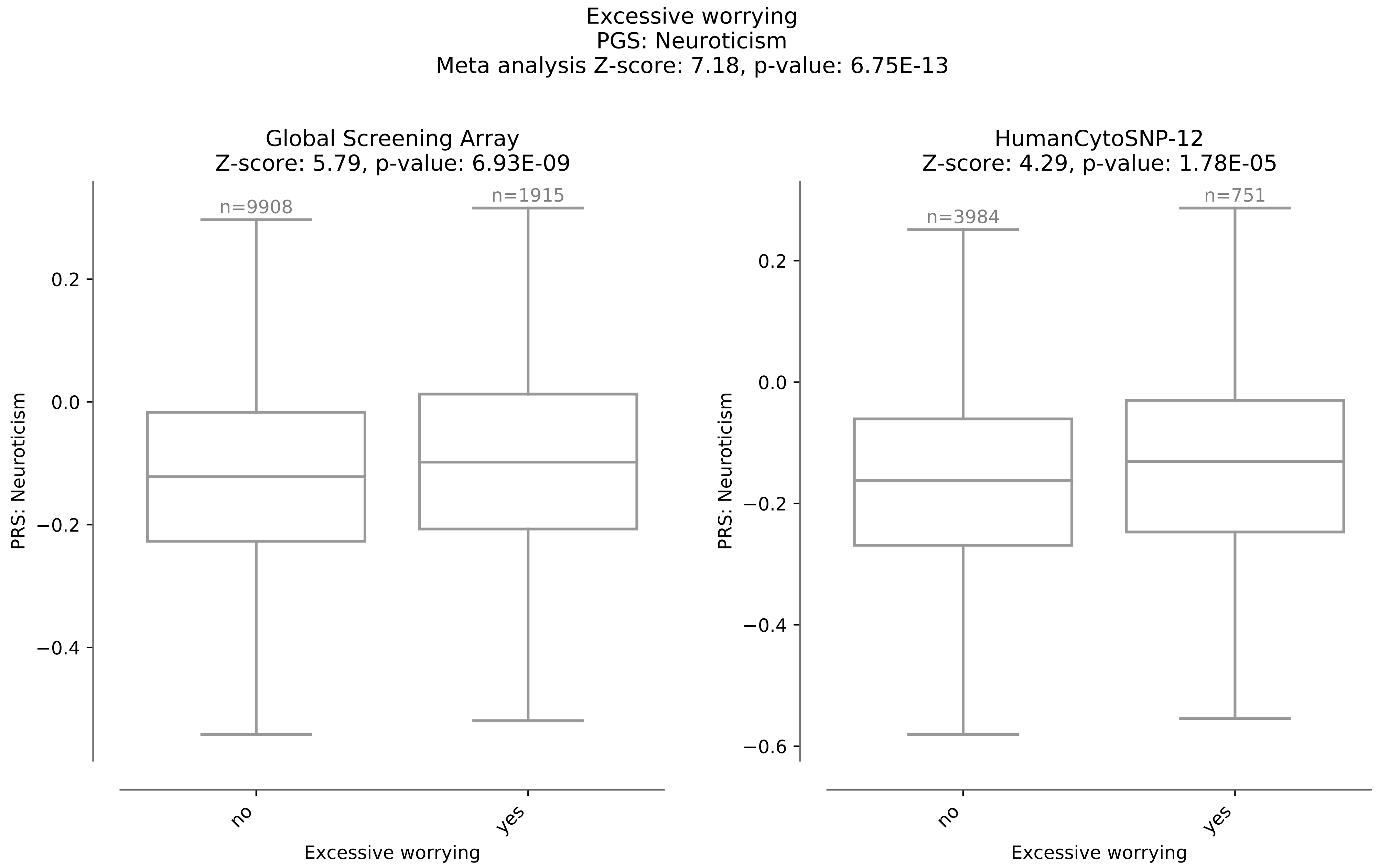
Excessive worrying
PGS: Neuroticism
Meta analysis Z-score: 7.18, p-value: 6.75E-13
Global Screening Array
Z-score: 5.79, p-value: 6.93E-09
n=9908
n=1915
0.2
0.0
−0.2
−0.4
PRS: Neuroticism
no
yes
Excessive worrying
HumanCytoSNP-12
Z-score: 4.29, p-value: 1.78E-05
n=3984
n=751
0.2
0.0
−0.2
−0.4
−0.6
PRS: Neuroticism
no
yes
Excessive worrying

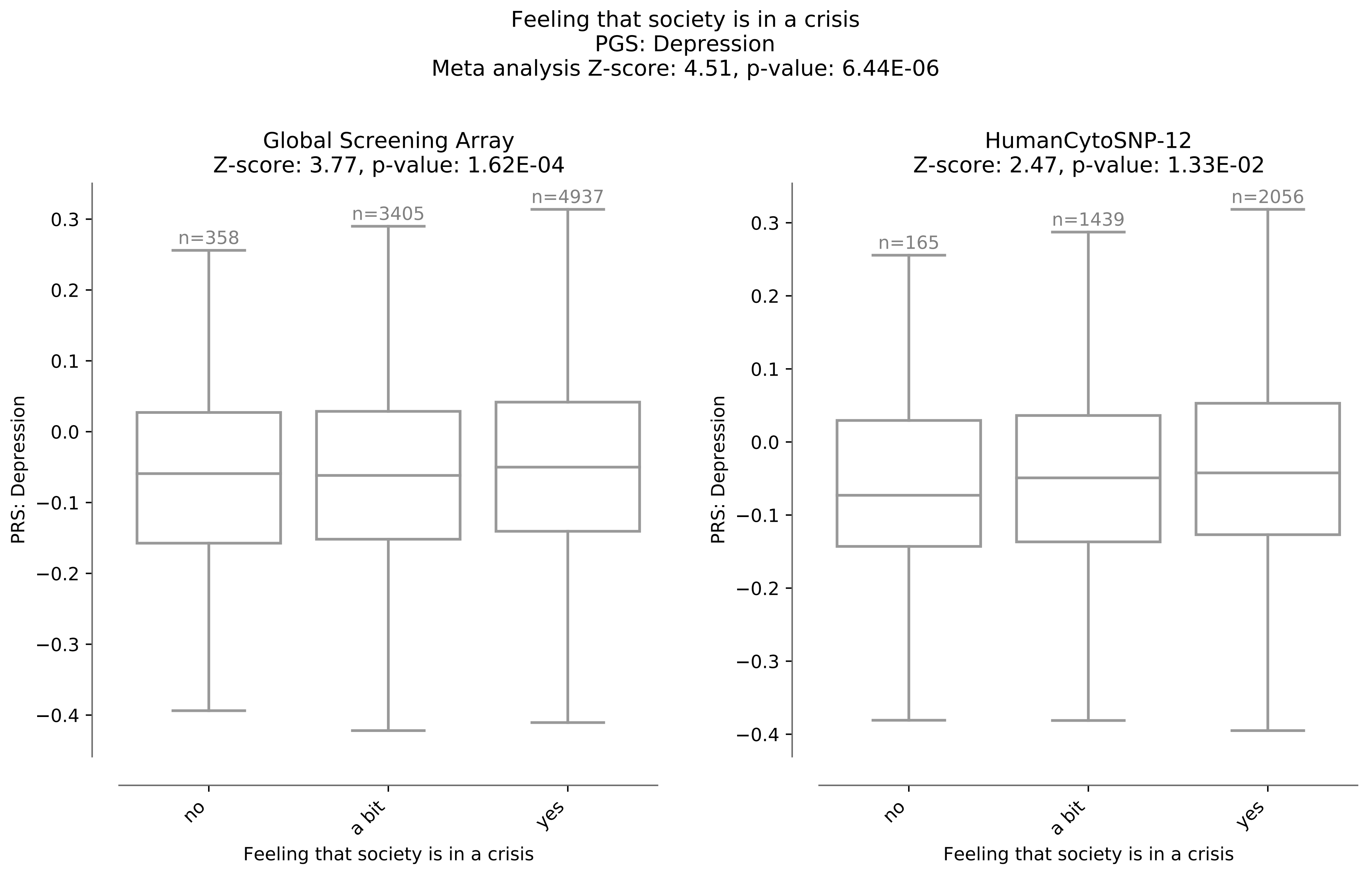

Feeling that society is in a crisis
PGS: Depression
Meta analysis Z-score: 4.51, p-value: 6.44E-06
Global Screening Array
Z-score: 3.77, p-value: 1.62E-04
n=358
n=3405
n=4937
PRS: Depression
0.3
0.2
0.1
0.0
−0.1
−0.2
−0.3
−0.4
no
a bit
yes
Feeling that society is in a crisis
HumanCytoSNP-12
Z-score: 2.47, p-value: 1.33E-02
n=165
n=1439
n=2056
PRS: Depression
0.3
0.2
0.1
0.0
−0.1
−0.2
−0.3
−0.4
no
a bit
yes
Feeling that society is in a crisis

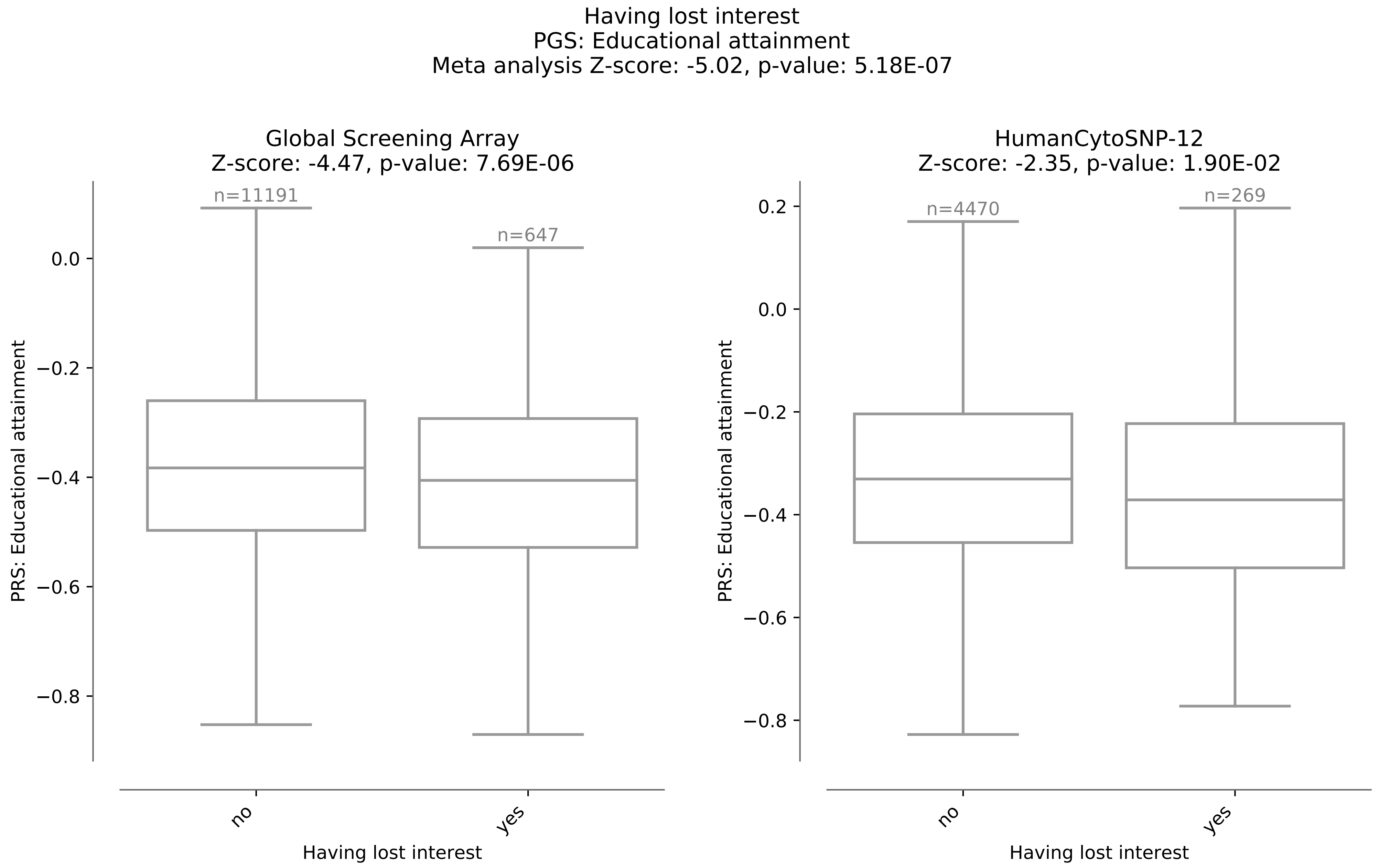
Having lost interest
PGS: Educational attainment
Meta analysis Z-score: -5.02, p-value: 5.18E-07
Global Screening Array
Z-score: -4.47, p-value: 7.69E-06
n=11191
n=647
PRS: Educational attainment
0.0
−0.2
−0.4
−0.6
−0.8
no
yes
Having lost interest
HumanCytoSNP-12
Z-score: -2.35, p-value: 1.90E-02
n=4470
n=269
0.2
0.0
−0.2
−0.4
−0.6
−0.8
PRS: Educational attainment
no
yes
Having lost interest

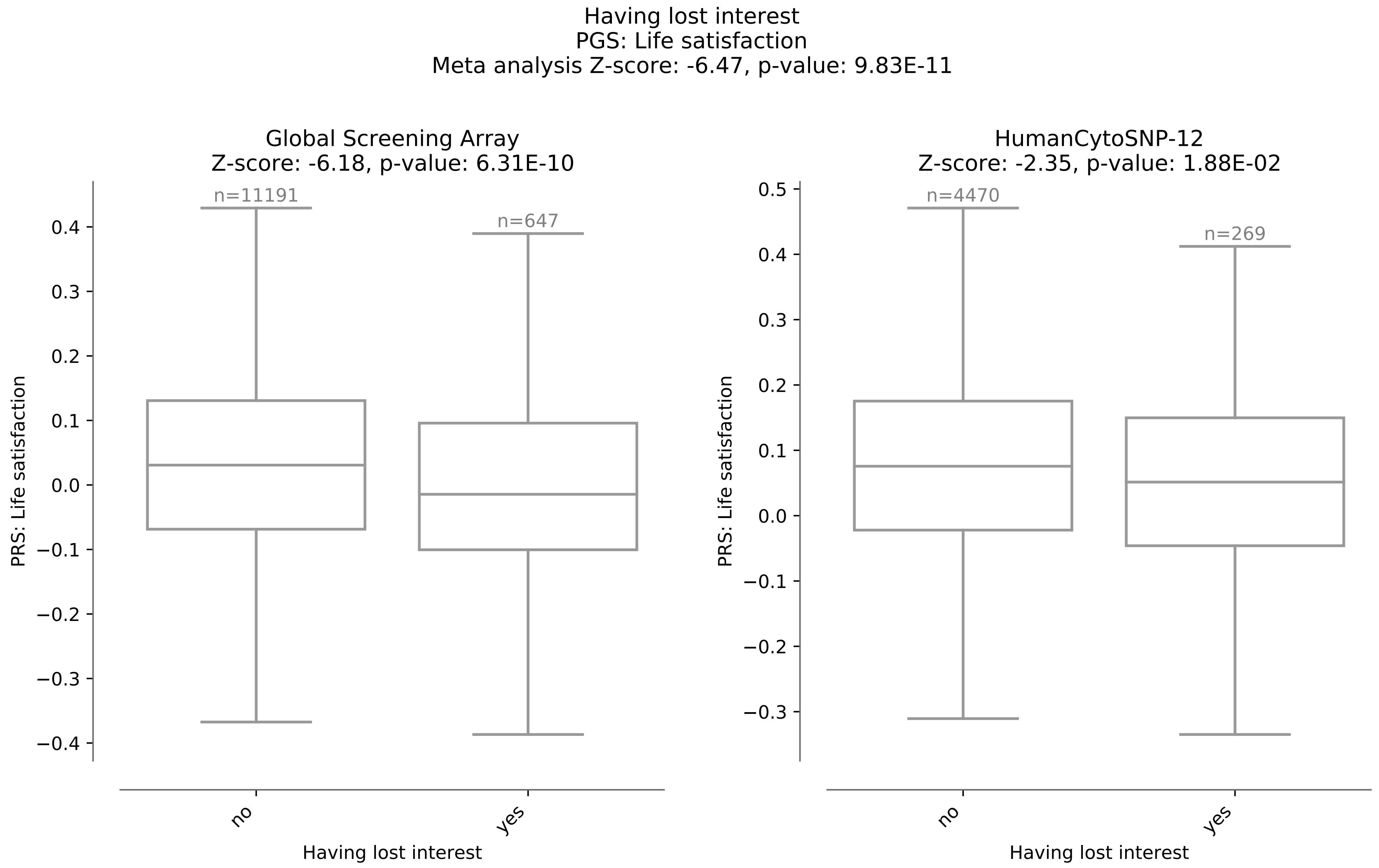
Having lost interest
PGS: Life satisfaction
Meta analysis Z-score: -6.47, p-value: 9.83E-11
Global Screening Array
Z-score: -6.18, p-value: 6.31E-10
n=11191
n=647
PRS: Life satisfaction
0.4
0.3
0.2
0.1
0.0
−0.1
−0.2
−0.3
−0.4
no
yes
Having lost interest
HumanCytoSNP-12
Z-score: -2.35, p-value: 1.88E-02
n=4470
n=269
PRS: Life satisfaction
0.5
0.4
0.3
0.2
0.1
0.0
−0.1
−0.2
−0.3
no
yes
Having lost interest

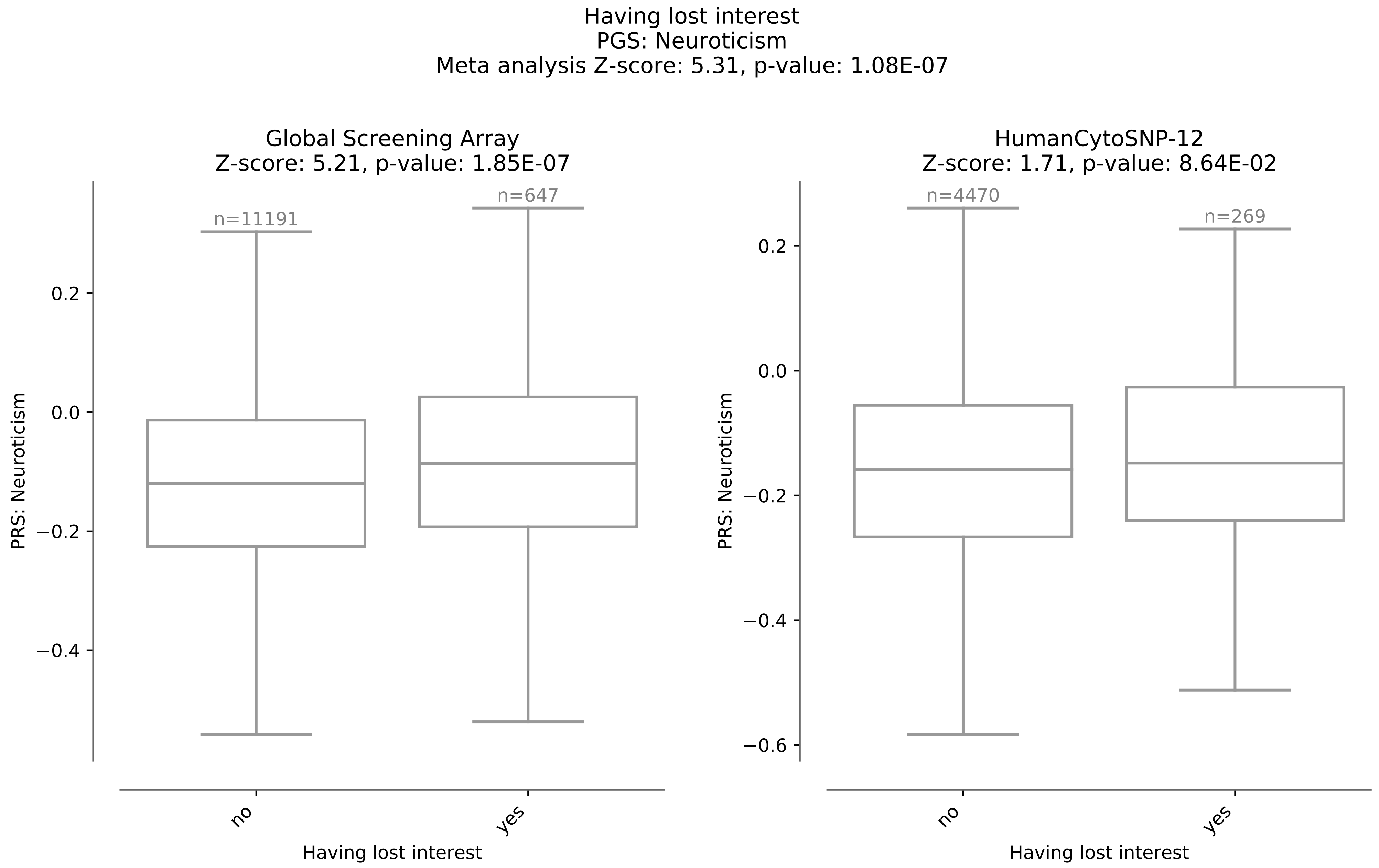
Having lost interest
PGS: Neuroticism
Meta analysis Z-score: 5.31, p-value: 1.08E-07
Global Screening Array
Z-score: 5.21, p-value: 1.85E-07
n=11191
n=647
0.2
0.0
−0.2
−0.4
PRS: Neuroticism
no
yes
Having lost interest
HumanCytoSNP-12
Z-score: 1.71, p-value: 8.64E-02
n=4470
n=269
0.2
0.0
−0.2
−0.4
−0.6
PRS: Neuroticism
no
yes
Having lost interest

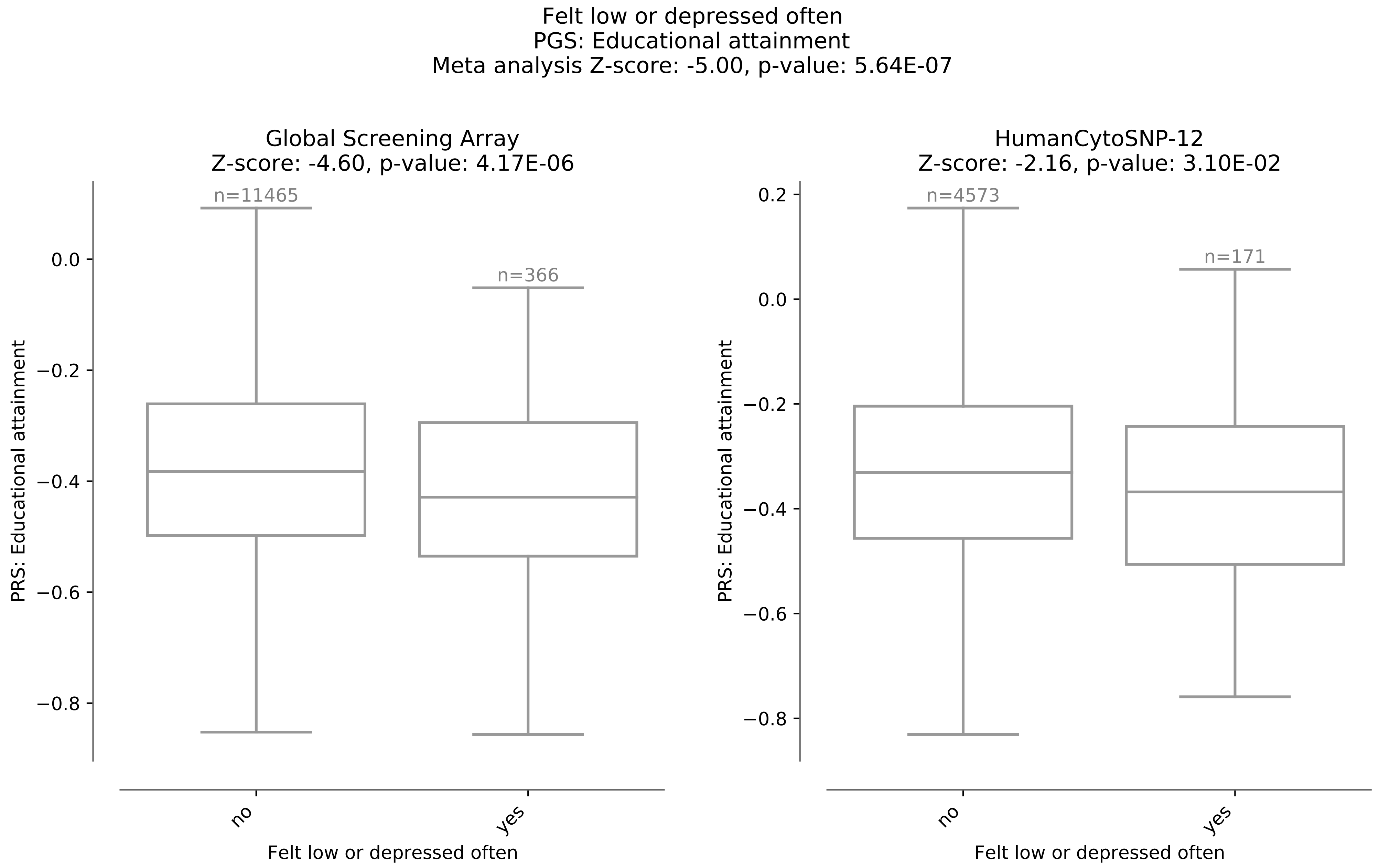
Felt low or depressed often
PGS: Educational attainment
Meta analysis Z-score: -5.00, p-value: 5.64E-07
Global Screening Array
Z-score: -4.60, p-value: 4.17E-06
n=11465
n=366
PRS: Educational attainment
0.0
−0.2
−0.4
−0.6
−0.8
no
yes
Felt low or depressed often
HumanCytoSNP-12
Z-score: -2.16, p-value: 3.10E-02
n=4573
n=171
PRS: Educational attainment
0.2
0.0
−0.2
−0.4
−0.6
−0.8
no
yes
Felt low or depressed often

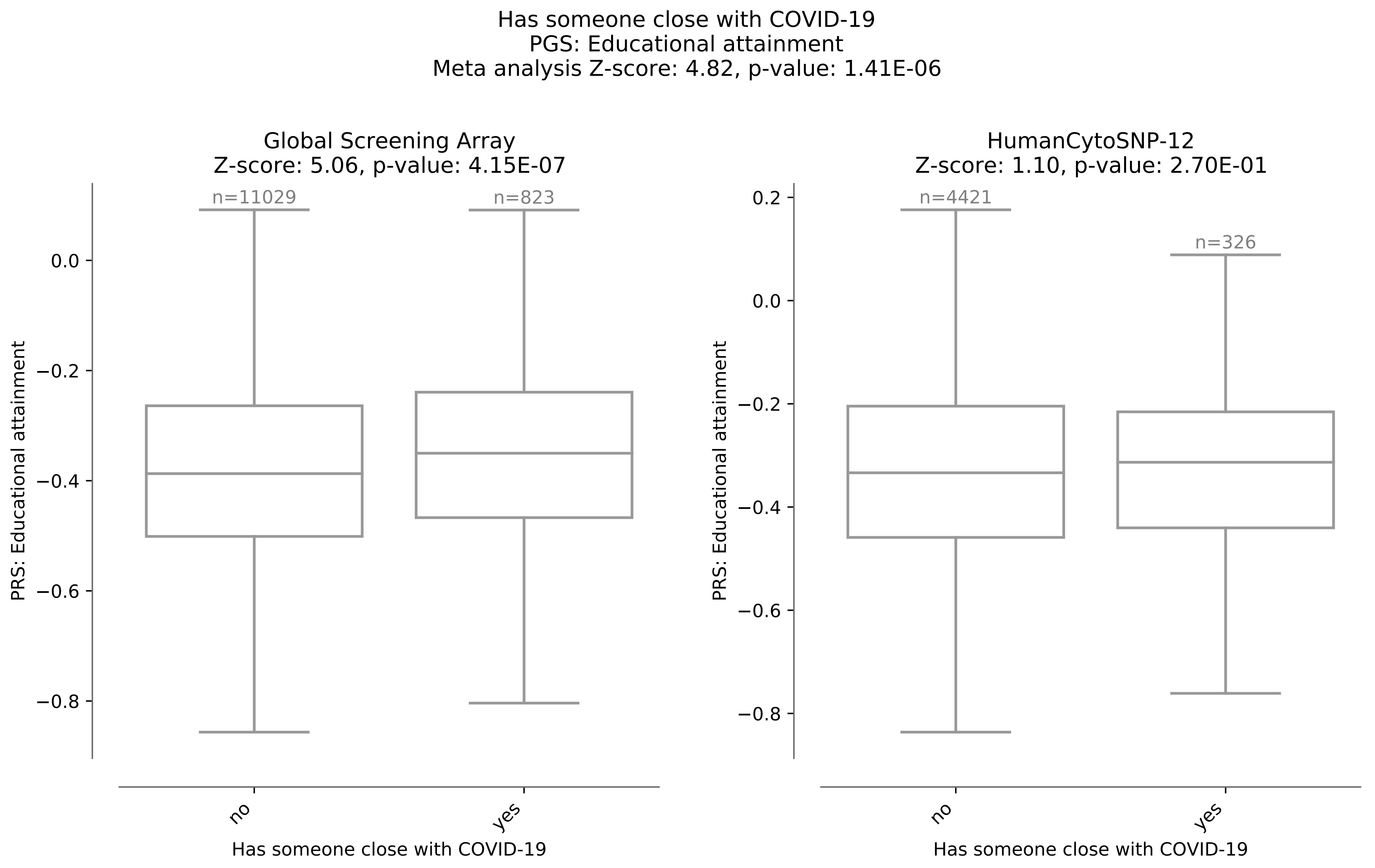
Has someone close with COVID-19
PGS: Educational attainment
Meta analysis Z-score: 4.82, p-value: 1.41E-06
Global Screening Array
Z-score: 5.06, p-value: 4.15E-07
n=11029
n=823
PRS: Educational attainment
0.0
−0.2
−0.4
−0.6
−0.8
no
yes
Has someone close with COVID-19
HumanCytoSNP-12
Z-score: 1.10, p-value: 2.70E-01
n=4421
n=326
PRS: Educational attainment
0.2
0.0
−0.2
−0.4
−0.6
−0.8
no
yes
Has someone close with COVID-19

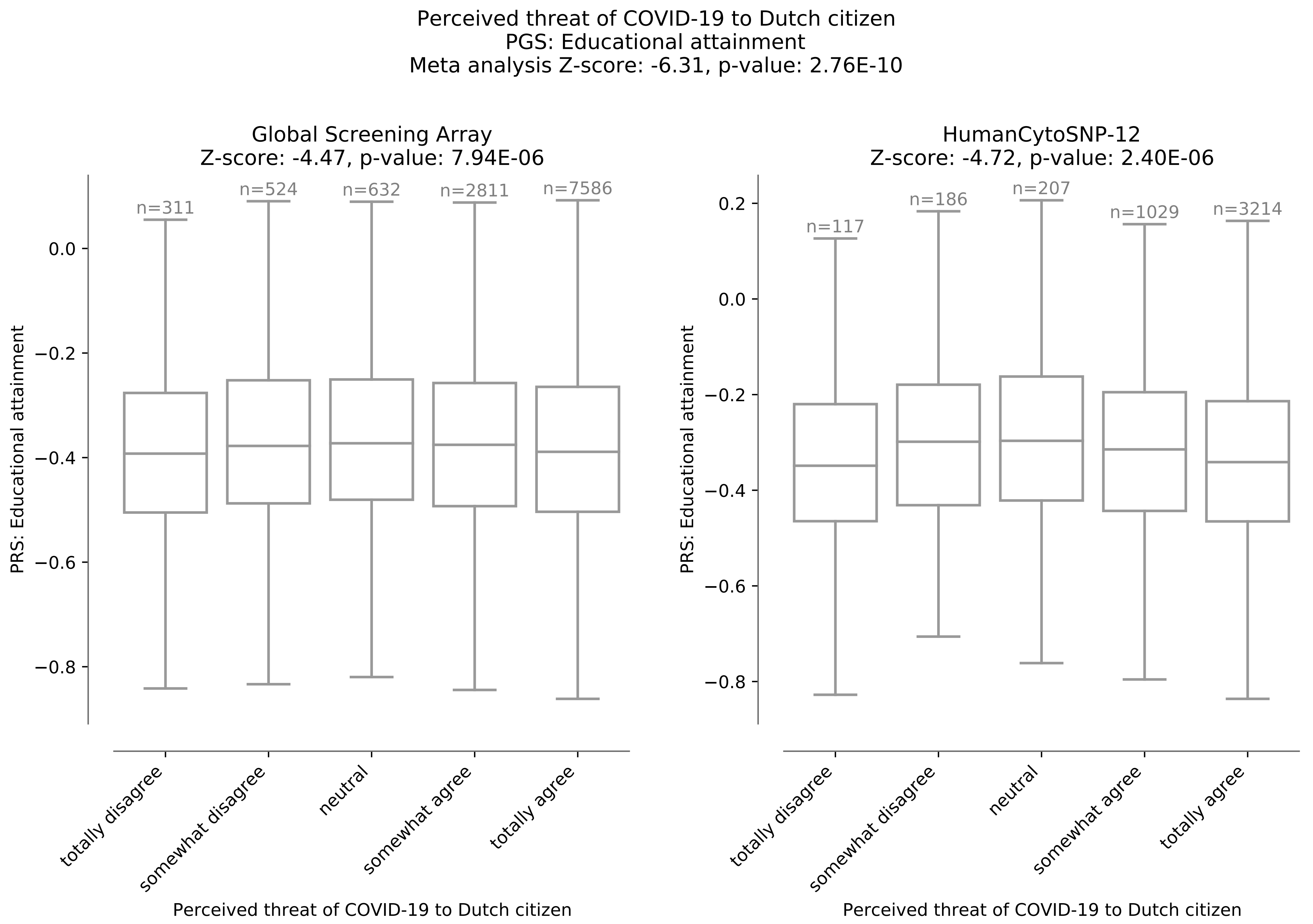
Perceived threat of COVID-19 to Dutch citizen
PGS: Educational attainment
Meta analysis Z-score: -6.31, p-value: 2.76E-10
Global Screening Array
Z-score: -4.47, p-value: 7.94E-06
HumanCytoSNP-12
Z-score: -4.72, p-value: 2.40E-06
n=311
n=524
n=632
n=2811
n=7586
n=117
n=186
n=207
n=1029
n=3214
PRS: Educational attainment
0.2
0.0
−0.2
−0.4
−0.6
−0.8
totally disagree
somewhat disagree
neutral
somewhat agree
totally agree
Perceived threat of COVID-19 to Dutch citizen

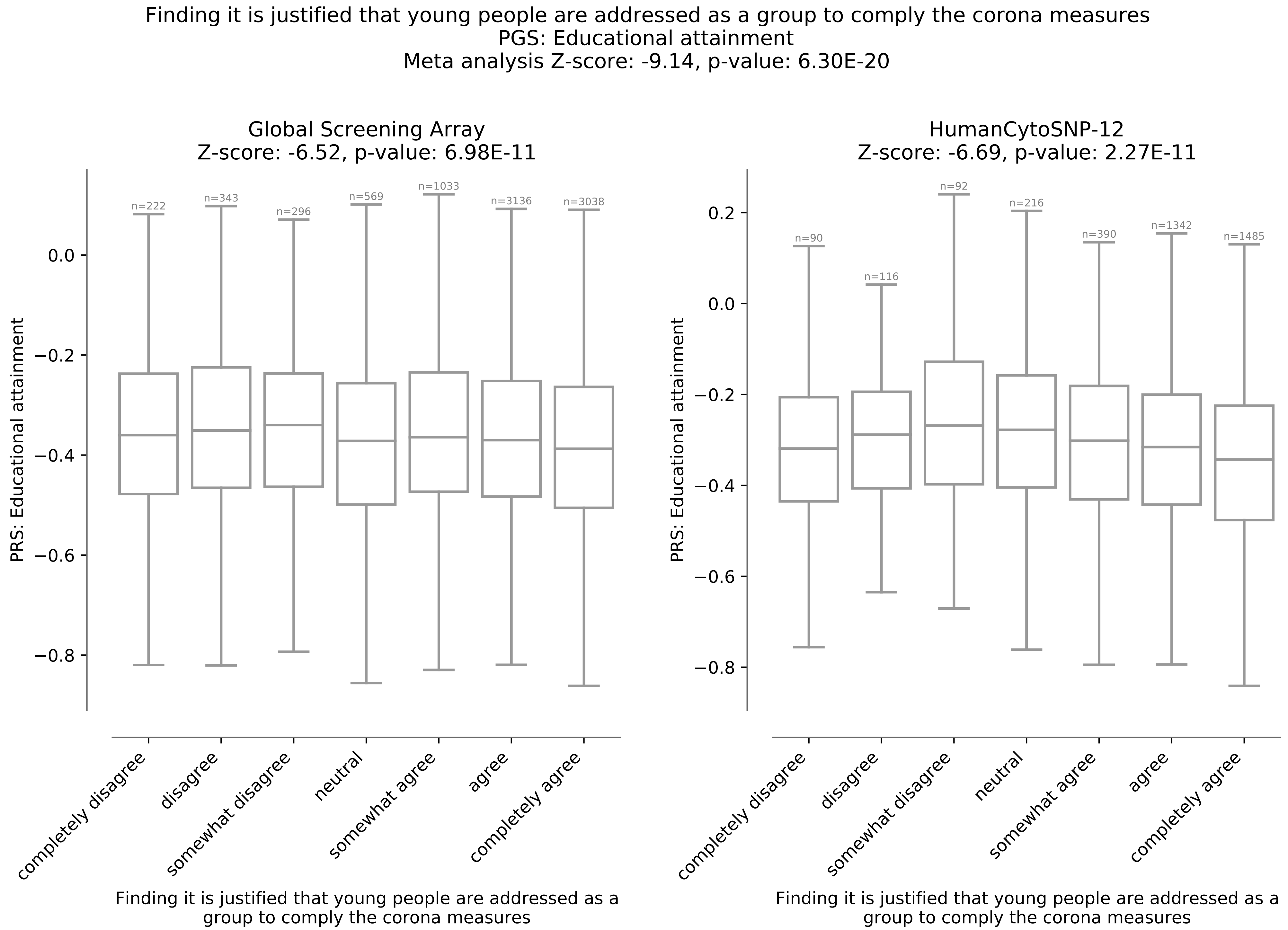
Finding it is justified that young people are addressed as a group to comply the corona measures
PGS: Educational attainment
Meta analysis Z-score: -9.14, p-value: 6.30E-20
Global Screening Array
Z-score: -6.52, p-value: 6.98E-11
HumanCytoSNP-12
Z-score: -6.69, p-value: 2.27E-11
PRS: Educational attainment
n=222
n=343
n=296
n=569
n=1033
n=3136
n=3038
n=90
n=116
n=92
n=216
n=390
n=1342
n=1485
completely disagree
disagree
somewhat disagree
neutral
somewhat agree
agree
completely agree
Finding it is justified that young people are addressed as a group to comply the corona measures

Finding vaccines contribute to the return to the normal living situation
PGS: Educational attainment
Meta analysis Z-score: 6.00, p-value: 1.92E-09

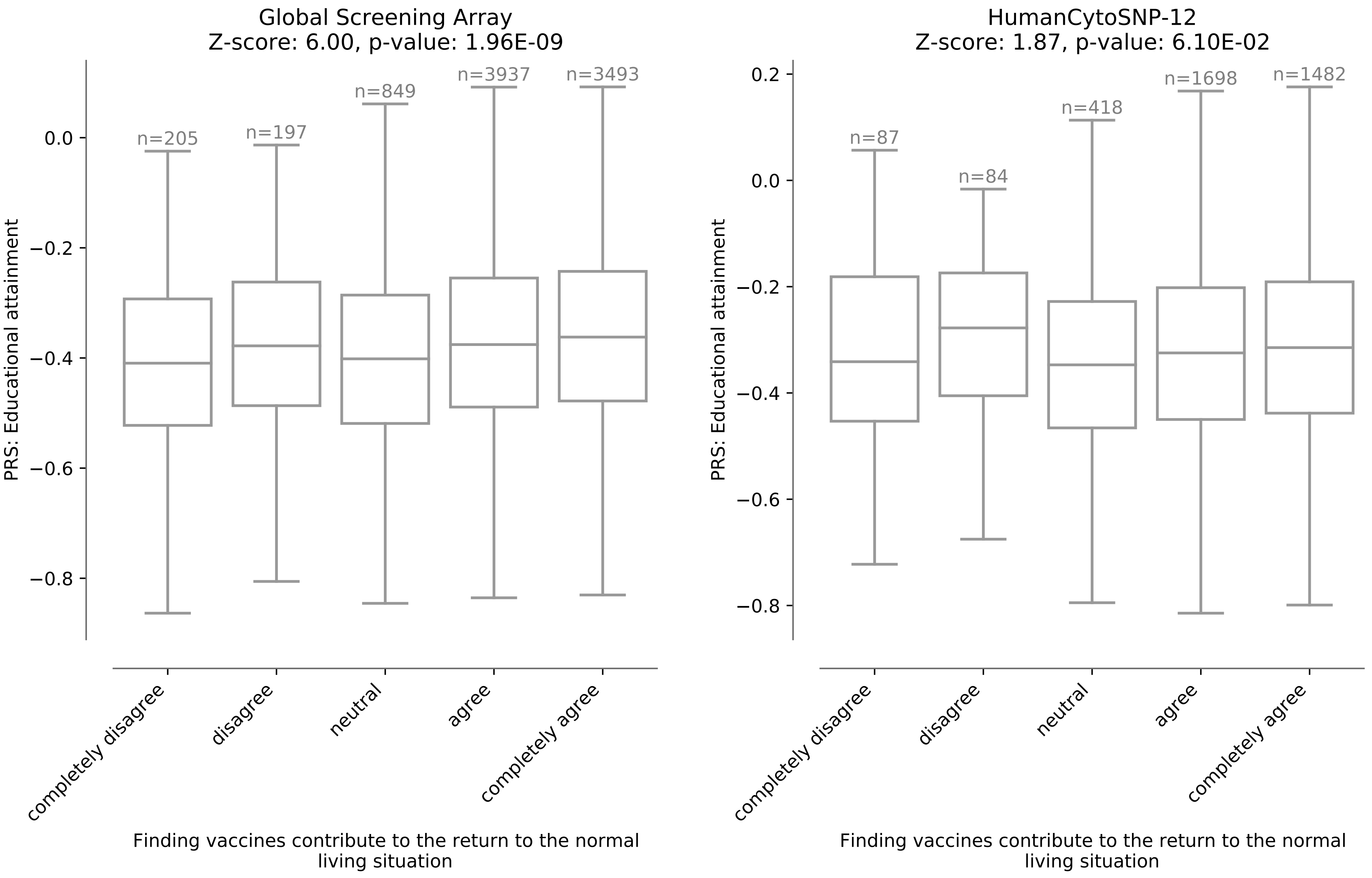

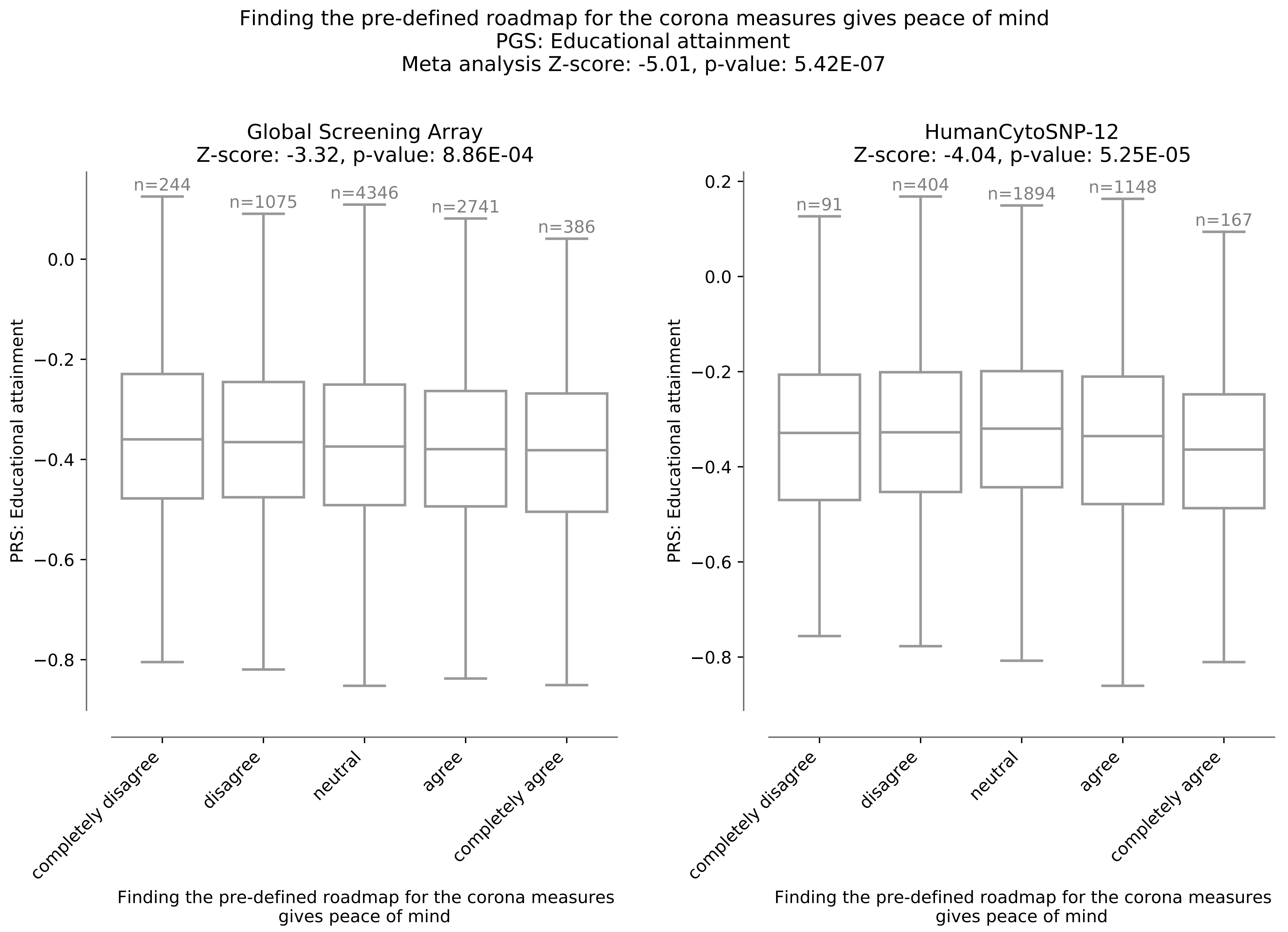
Finding the pre-defined roadmap for the corona measures gives peace of mind
PGS: Educational attainment
Meta analysis Z-score: -5.01, p-value: 5.42E-07
Global Screening Array
Z-score: -3.32, p-value: 8.86E-04
n=244
n=1075
n=4346
n=2741
n=386
0.0
−0.2
−0.4
−0.6
−0.8
PRS: Educational attainment
completely disagree
disagree
neutral
agree
completely agree
Finding the pre-defined roadmap for the corona measures gives peace of mind
HumanCytoSNP-12
Z-score: -4.04, p-value: 5.25E-05
n=91
n=404
n=1894
n=1148
n=167
0.2
0.0
−0.2
−0.4
−0.6
−0.8
PRS: Educational attainment
completely disagree
disagree
neutral
agree
completely agree
Finding the pre-defined roadmap for the corona measures gives peace of mind

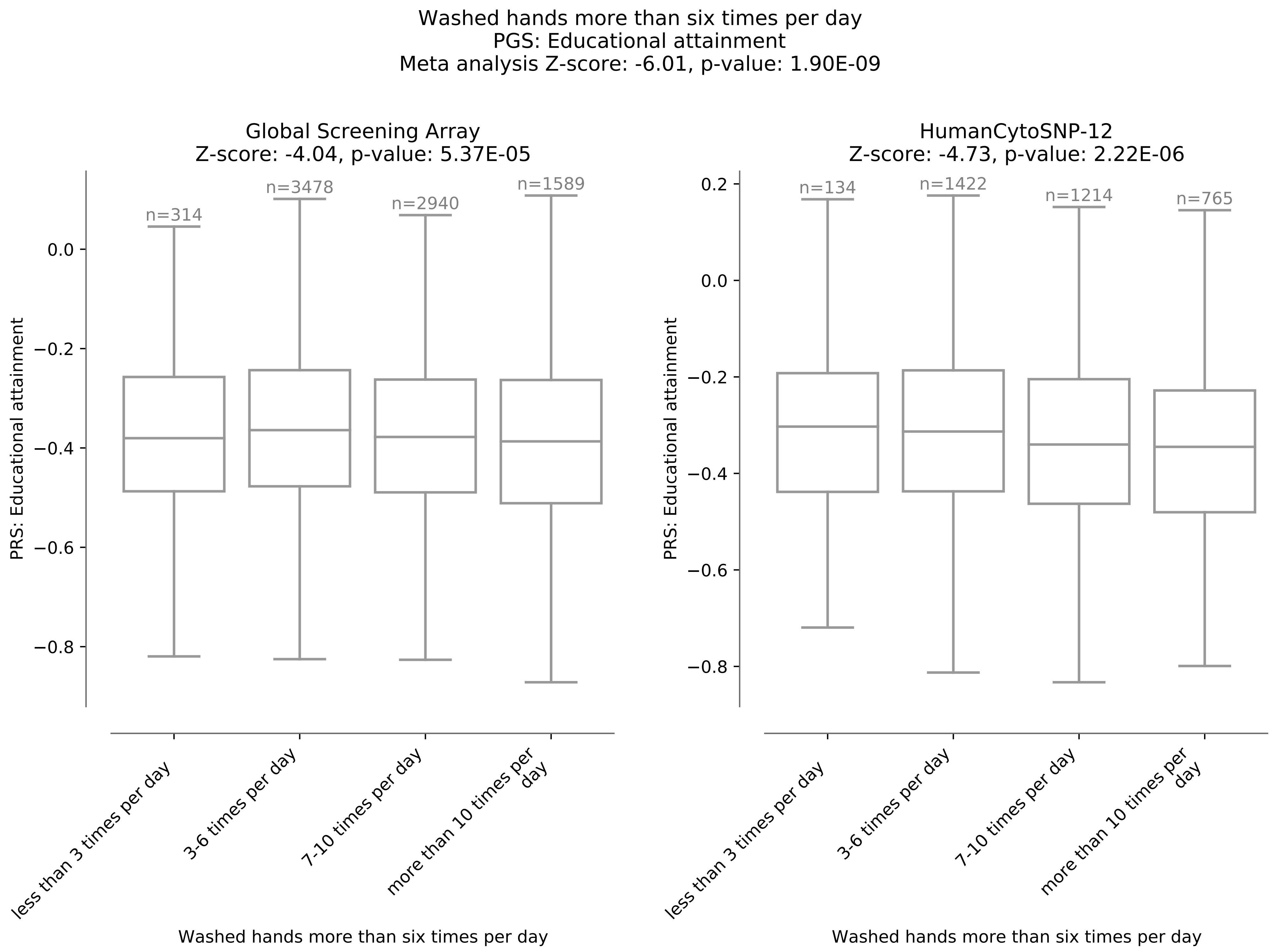

Washed hands more than six times per day
PGS: Educational attainment
Meta analysis Z-score: -6.01, p-value: 1.90E-09
Global Screening Array
Z-score: -4.04, p-value: 5.37E-05
HumanCytoSNP-12
Z-score: -4.73, p-value: 2.22E-06
PRS: Educational attainment
n=314
n=3478
n=2940
n=1589
n=134
n=1422
n=1214
n=765
0.2
0.0
−0.2
−0.4
−0.6
−0.8
less than 3 times per day
3-6 times per day
7-10 times per day
more than 10 times per day
Washed hands more than six times per day

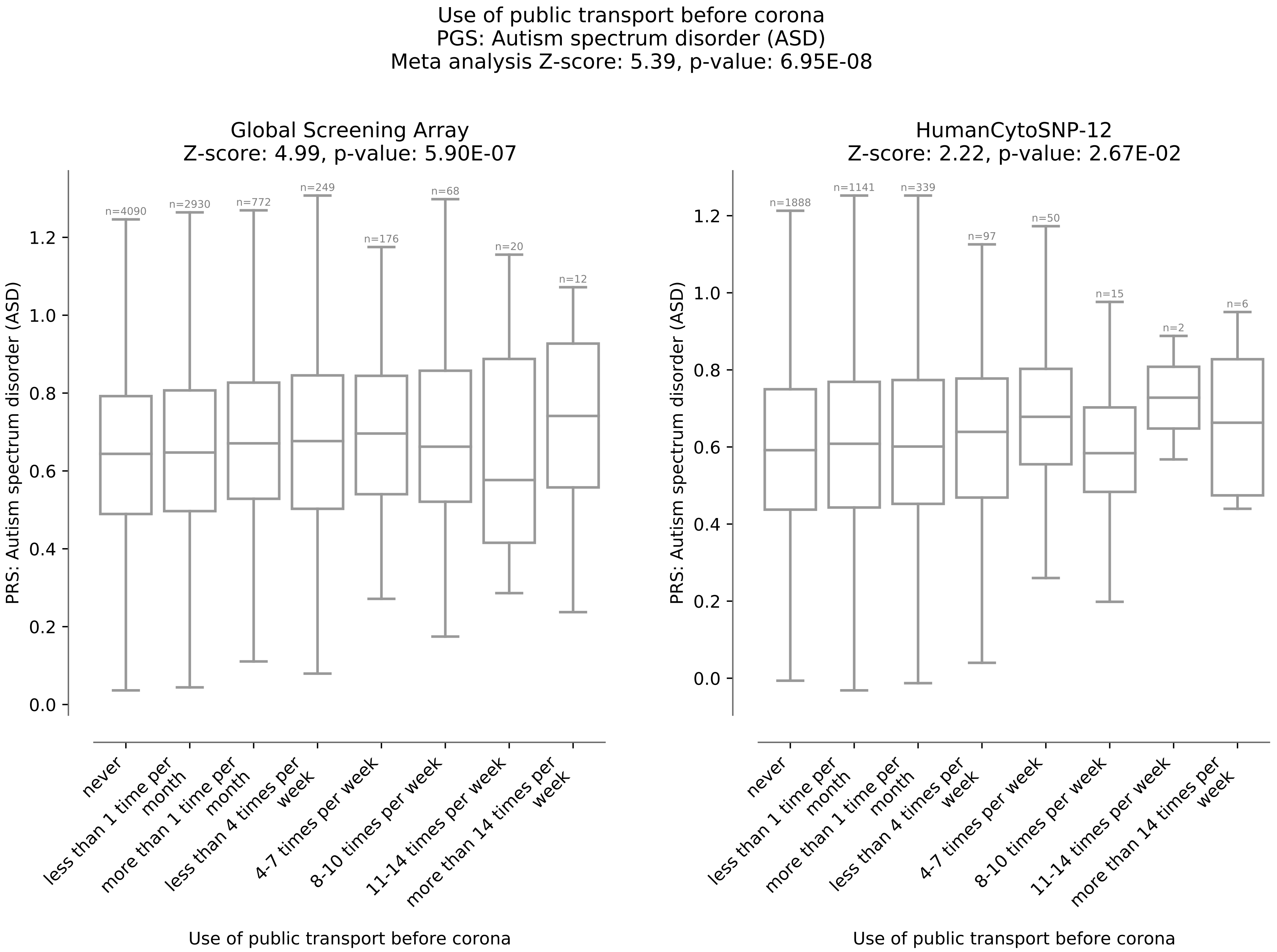

Use of public transport before corona
PGS: Autism spectrum disorder (ASD)
Meta analysis Z-score: 5.39, p-value: 6.95E-08
Global Screening Array
Z-score: 4.99, p-value: 5.90E-07
HumanCytoSNP-12
Z-score: 2.22, p-value: 2.67E-02
PRS: Autism spectrum disorder (ASD)
n=4090
n=2930
n=772
n=249
n=176
n=68
n=20
n=12
n=1888
n=1141
n=339
n=97
n=50
n=15
n=2
n=6
0.0
0.2
0.4
0.6
0.8
1.0
1.2
never
less than 1 time per month
more than 1 time per month
less than 4 times per week
4-7 times per week
8-10 times per week
11-14 times per week
more than 14 times per week
Use of public transport before corona

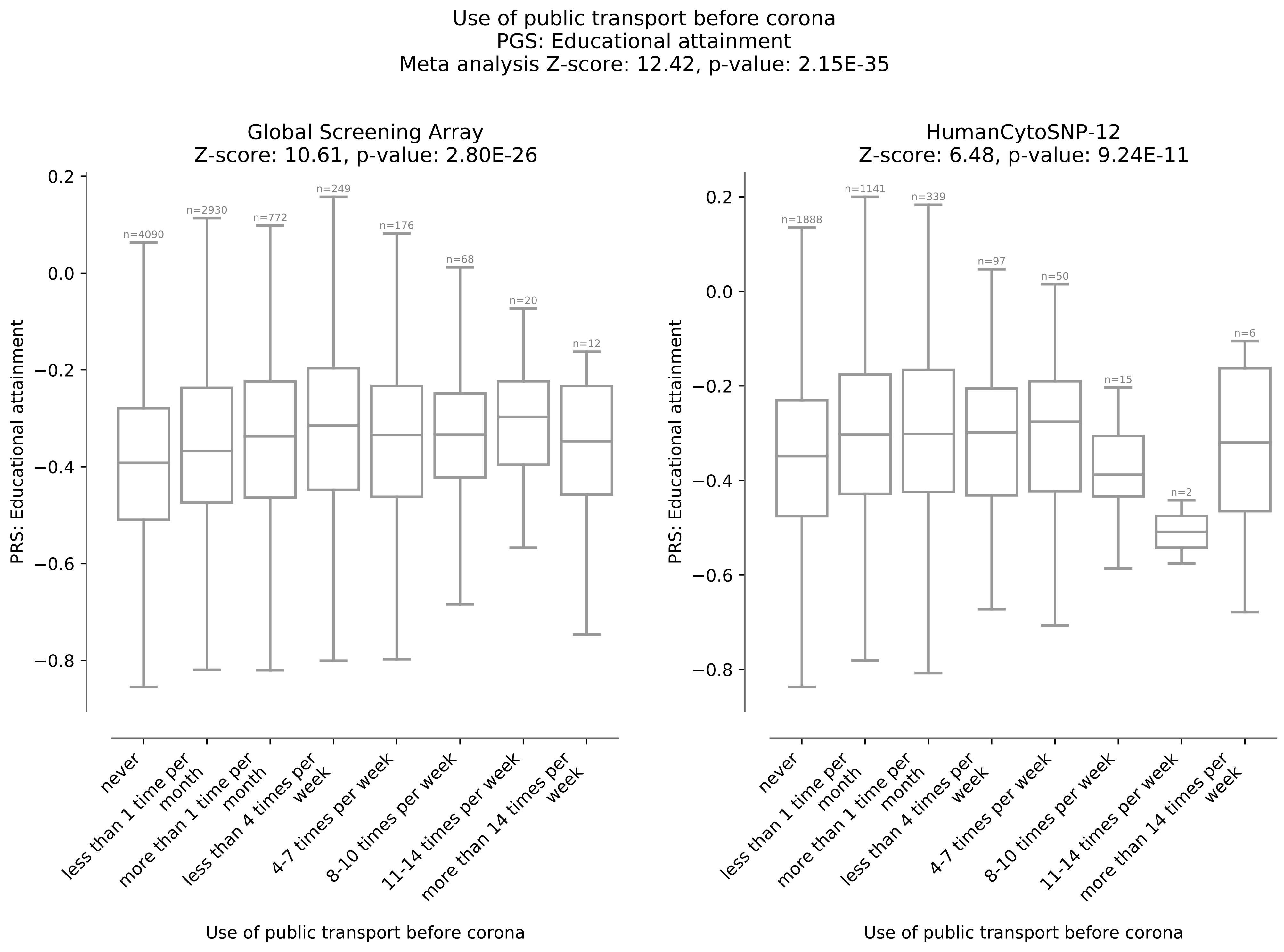
Use of public transport before corona
PGS: Educational attainment
Meta analysis Z-score: 12.42, p-value: 2.15E-35
Global Screening Array
Z-score: 10.61, p-value: 2.80E-26
HumanCytoSNP-12
Z-score: 6.48, p-value: 9.24E-11
PRS: Educational attainment
Use of public transport before corona
never
less than 1 time per month
more than 1 time per month
less than 4 times per week
4-7 times per week
8-10 times per week
11-14 times per week
more than 14 times per week
n=4090
n=2930
n=772
n=249
n=176
n=68
n=20
n=12
n=1888
n=1141
n=339
n=97
n=50
n=15
n=2
n=6

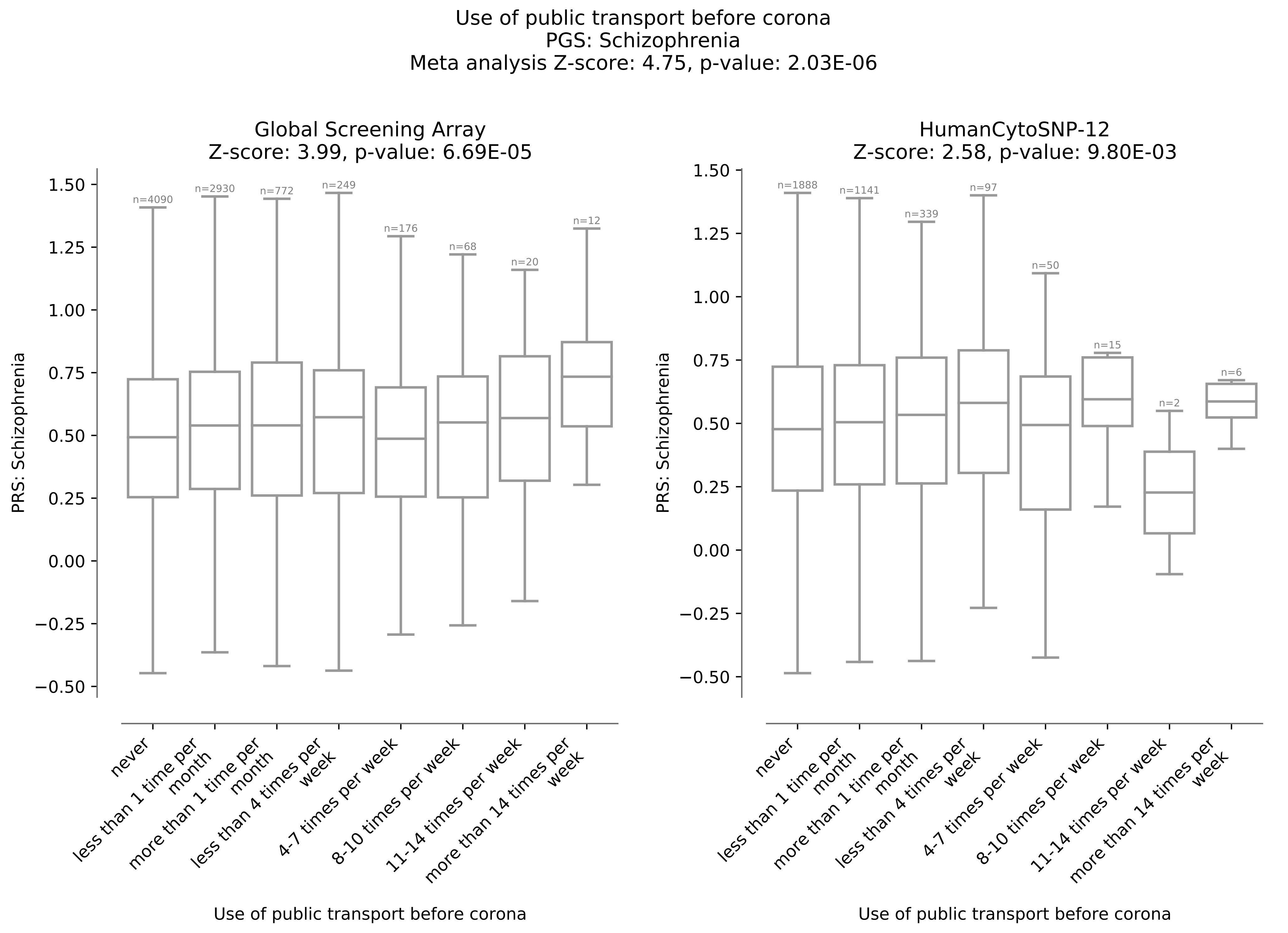
Use of public transport before corona
PGS: Schizophrenia
Meta analysis Z-score: 4.75, p-value: 2.03E-06
Global Screening Array
Z-score: 3.99, p-value: 6.69E-05
HumanCytoSNP-12
Z-score: 2.58, p-value: 9.80E-03
PRS: Schizophrenia
n=4090
n=2930
n=772
n=249
n=176
n=68
n=20
n=12
n=1888
n=1141
n=339
n=97
n=50
n=15
n=2
n=6
never
less than 1 time per month
more than 1 time per month
less than 4 times per week
4-7 times per week
8-10 times per week
11-14 times per week
more than 14 times per week
Use of public transport before corona

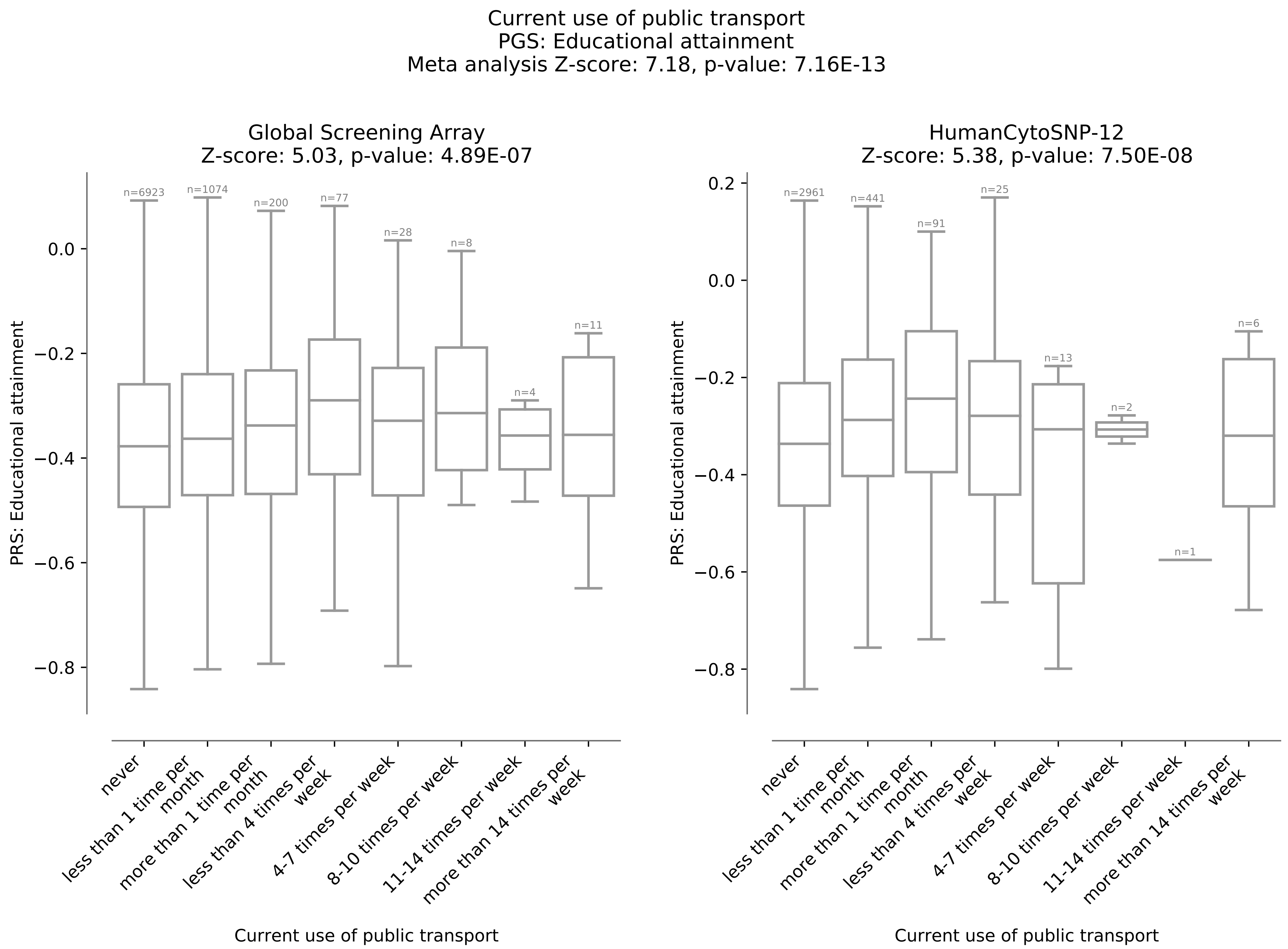
Current use of public transport
PGS: Educational attainment
Meta analysis Z-score: 7.18, p-value: 7.16E-13
Global Screening Array
Z-score: 5.03, p-value: 4.89E-07
HumanCytoSNP-12
Z-score: 5.38, p-value: 7.50E-08
PRS: Educational attainment
0.2
0.0
−0.2
−0.4
−0.6
−0.8
n=6923
n=1074
n=200
n=77
n=28
n=8
n=4
n=11
n=2961
n=441
n=91
n=25
n=13
n=2
n=1
n=6
never
less than 1 time per month
more than 1 time per month
less than 4 times per week
4-7 times per week
8-10 times per week
11-14 times per week
more than 14 times per week
Current use of public transport

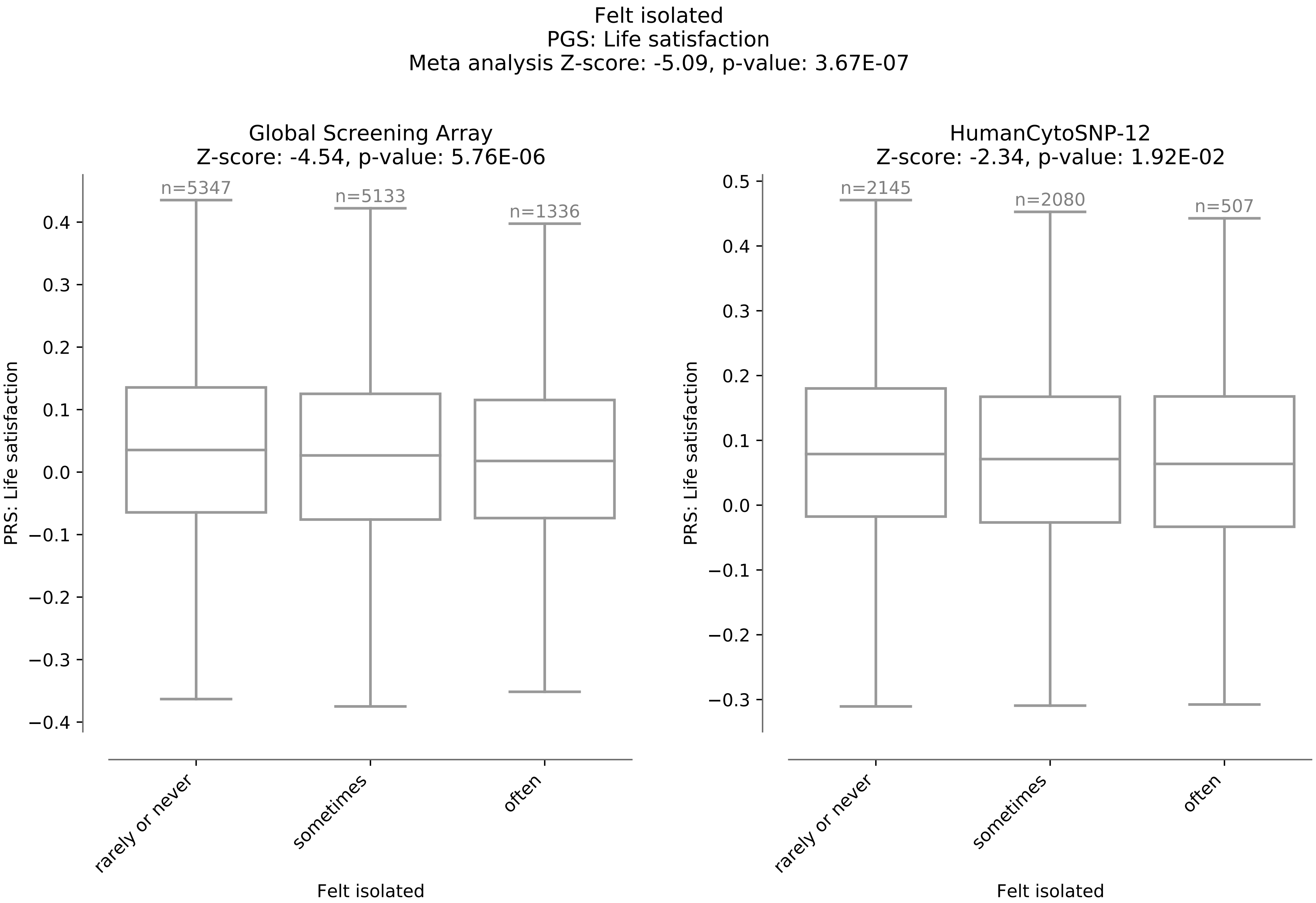
Felt isolated
PGS: Life satisfaction
Meta analysis Z-score: -5.09, p-value: 3.67E-07
Global Screening Array
Z-score: -4.54, p-value: 5.76E-06
HumanCytoSNP-12
Z-score: -2.34, p-value: 1.92E-02
PRS: Life satisfaction
n=5347
n=5133
n=1336
n=2145
n=2080
n=507
rarely or never
sometimes
often
Felt isolated

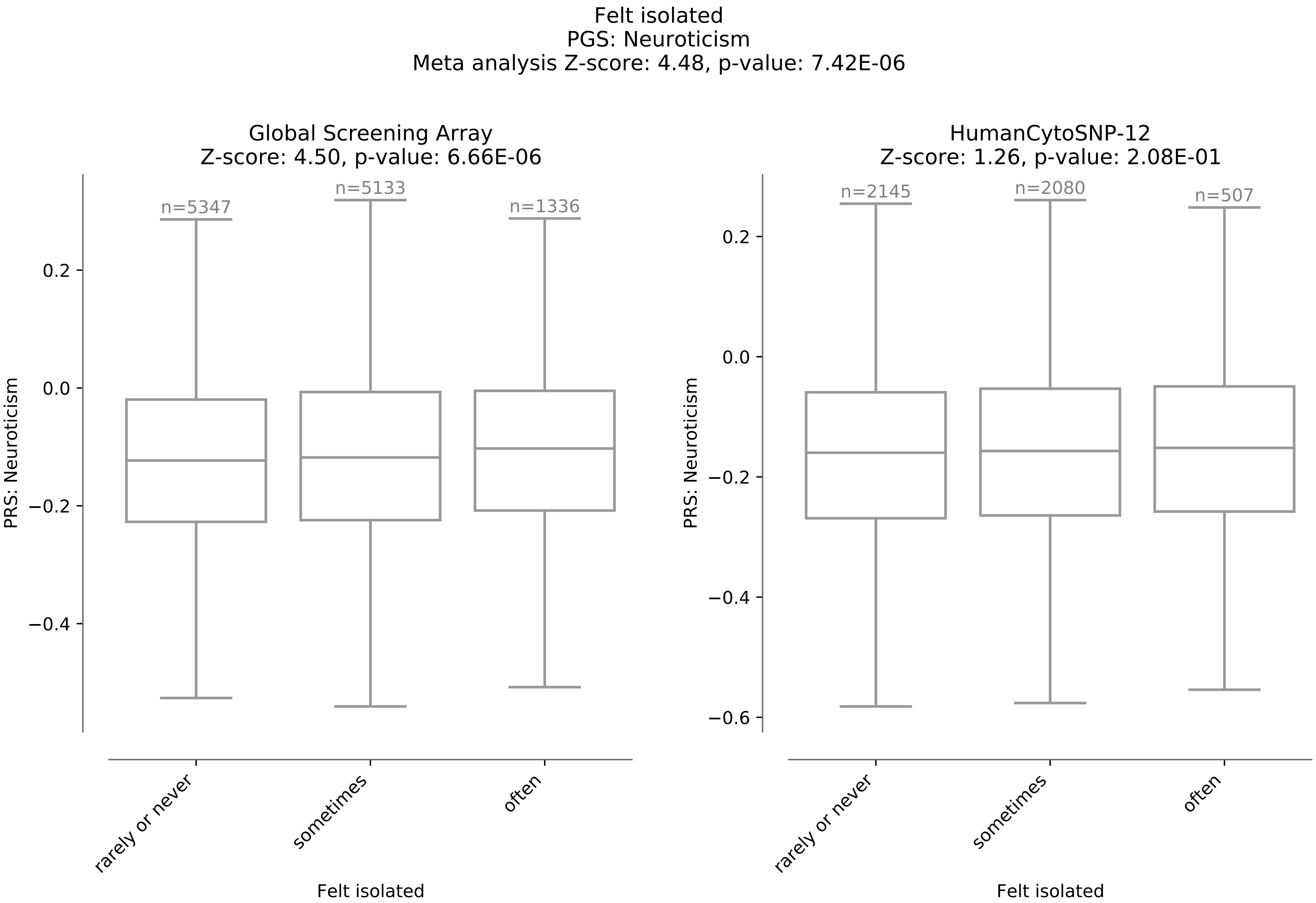

Felt isolated
PGS: Neuroticism
Meta analysis Z-score: 4.48, p-value: 7.42E-06
Global Screening Array
Z-score: 4.50, p-value: 6.66E-06
n=5347
n=5133
n=1336
0.2
0.0
−0.2
−0.4
PRS: Neuroticism
rarely or never
sometimes
often
Felt isolated
HumanCytoSNP-12
Z-score: 1.26, p-value: 2.08E-01
n=2145
n=2080
n=507
0.2
0.0
−0.2
−0.4
−0.6
PRS: Neuroticism
rarely or never
sometimes
often
Felt isolated

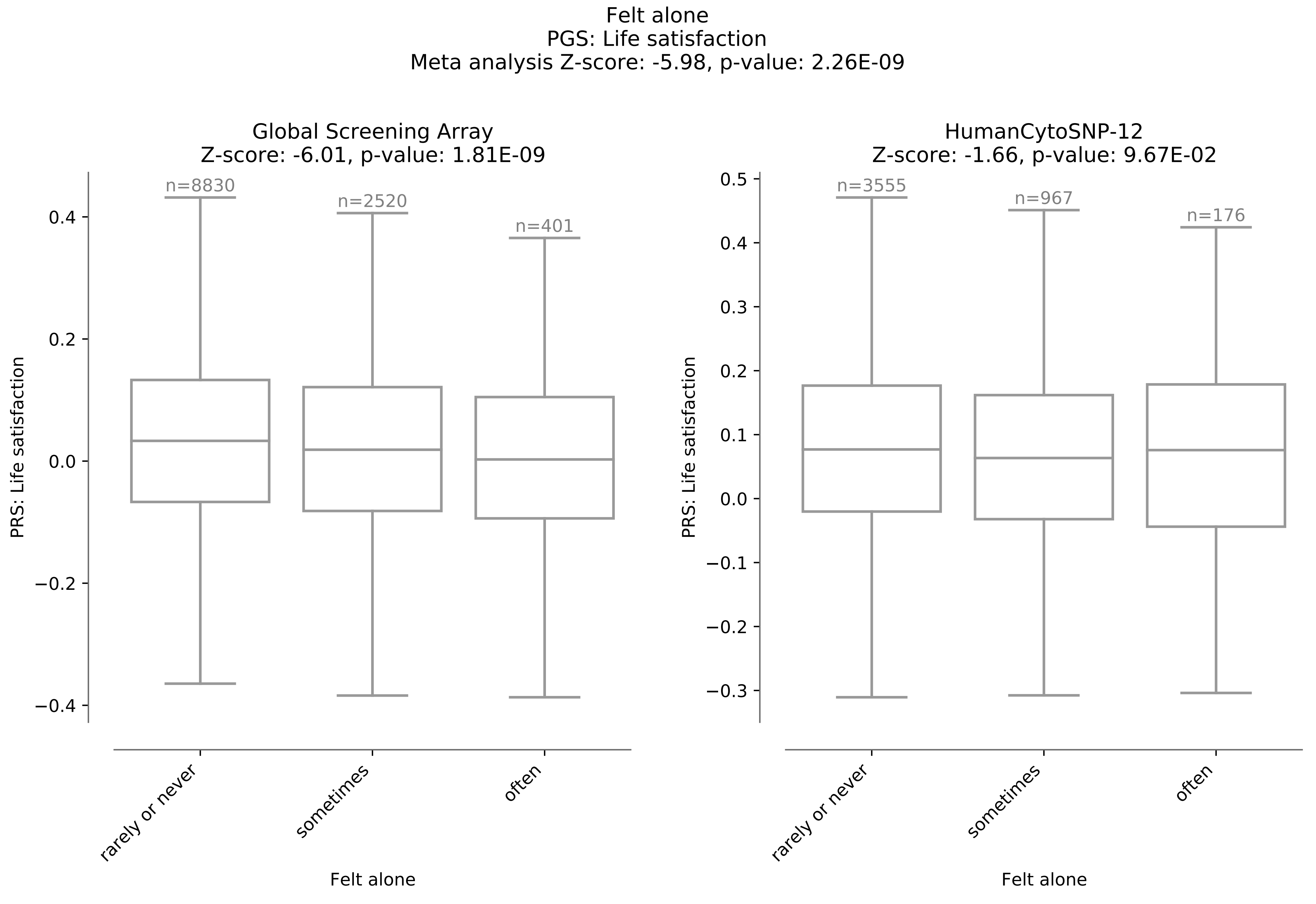

Felt alone
PGS: Life satisfaction
Meta analysis Z-score: -5.98, p-value: 2.26E-09
Global Screening Array
Z-score: -6.01, p-value: 1.81E-09
HumanCytoSNP-12
Z-score: -1.66, p-value: 9.67E-02
PRS: Life satisfaction
n=8830
n=2520
n=401
n=3555
n=967
n=176
0.4
0.2
0.0
−0.2
−0.4
0.5
0.3
0.1
−0.1
−0.3
rarely or never
sometimes
often
Felt alone

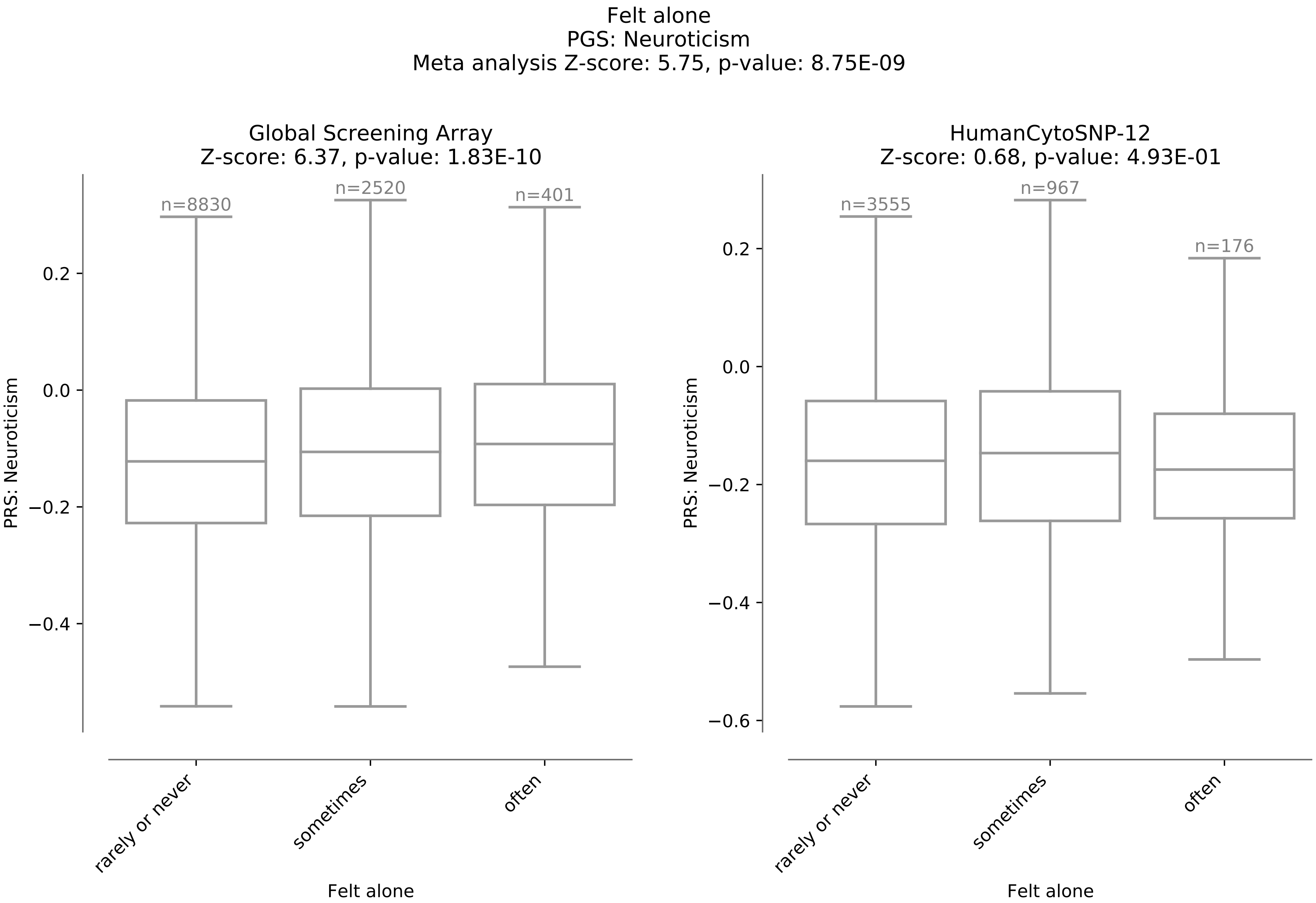
Felt alone
PGS: Neuroticism
Meta analysis Z-score: 5.75, p-value: 8.75E-09
Global Screening Array
Z-score: 6.37, p-value: 1.83E-10
HumanCytoSNP-12
Z-score: 0.68, p-value: 4.93E-01
n=8830
n=2520
n=401
n=3555
n=967
n=176
PRS: Neuroticism
0.2
0.0
−0.2
−0.4
−0.6
rarely or never
sometimes
often
Felt alone

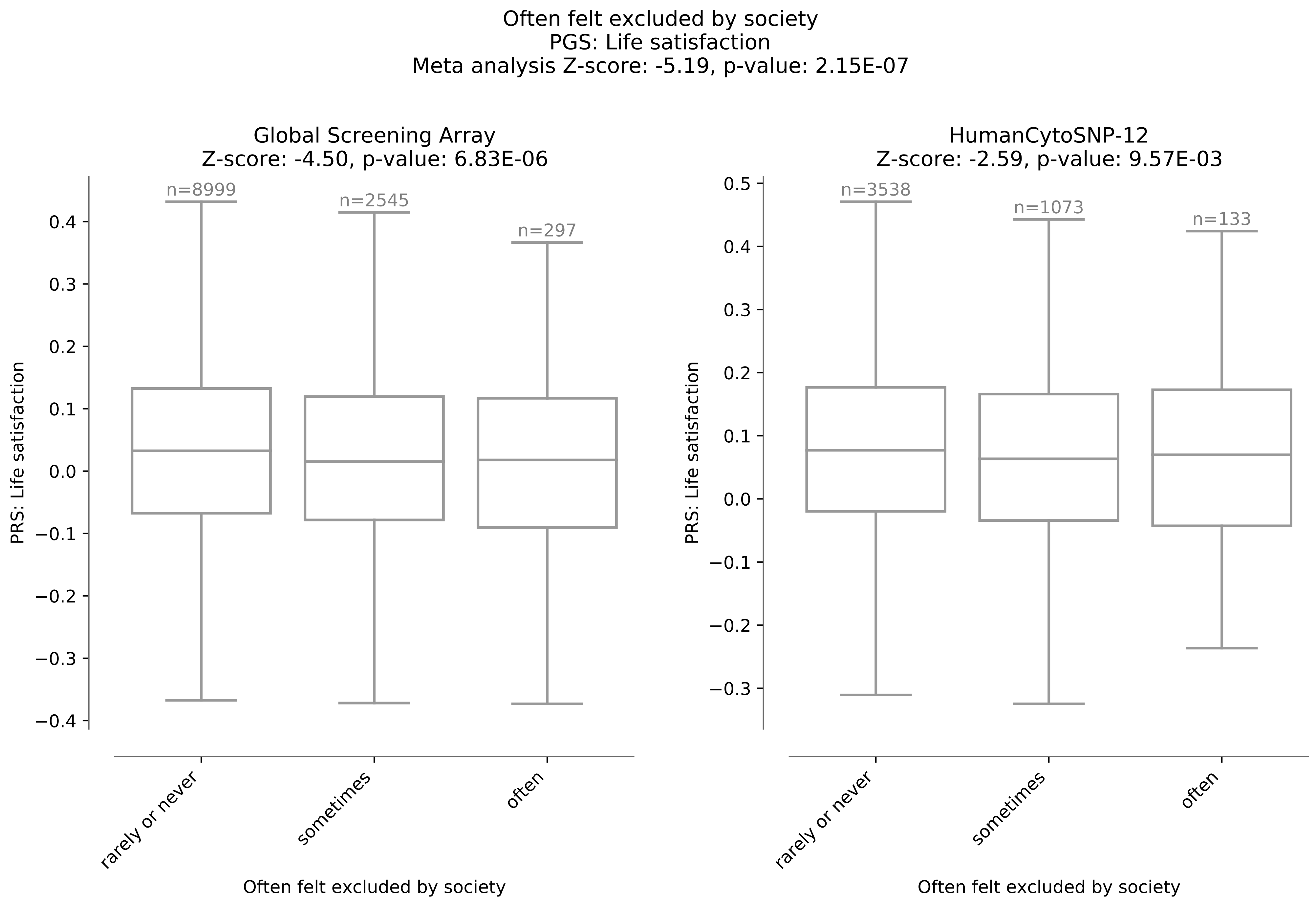
Often felt excluded by society
PGS: Life satisfaction
Meta analysis Z-score: -5.19, p-value: 2.15E-07
Global Screening Array
Z-score: -4.50, p-value: 6.83E-06
n=8999
n=2545
n=297
PRS: Life satisfaction
rarely or never
sometimes
often
Often felt excluded by society
HumanCytoSNP-12
Z-score: -2.59, p-value: 9.57E-03
n=3538
n=1073
n=133
PRS: Life satisfaction
rarely or never
sometimes
often
Often felt excluded by society

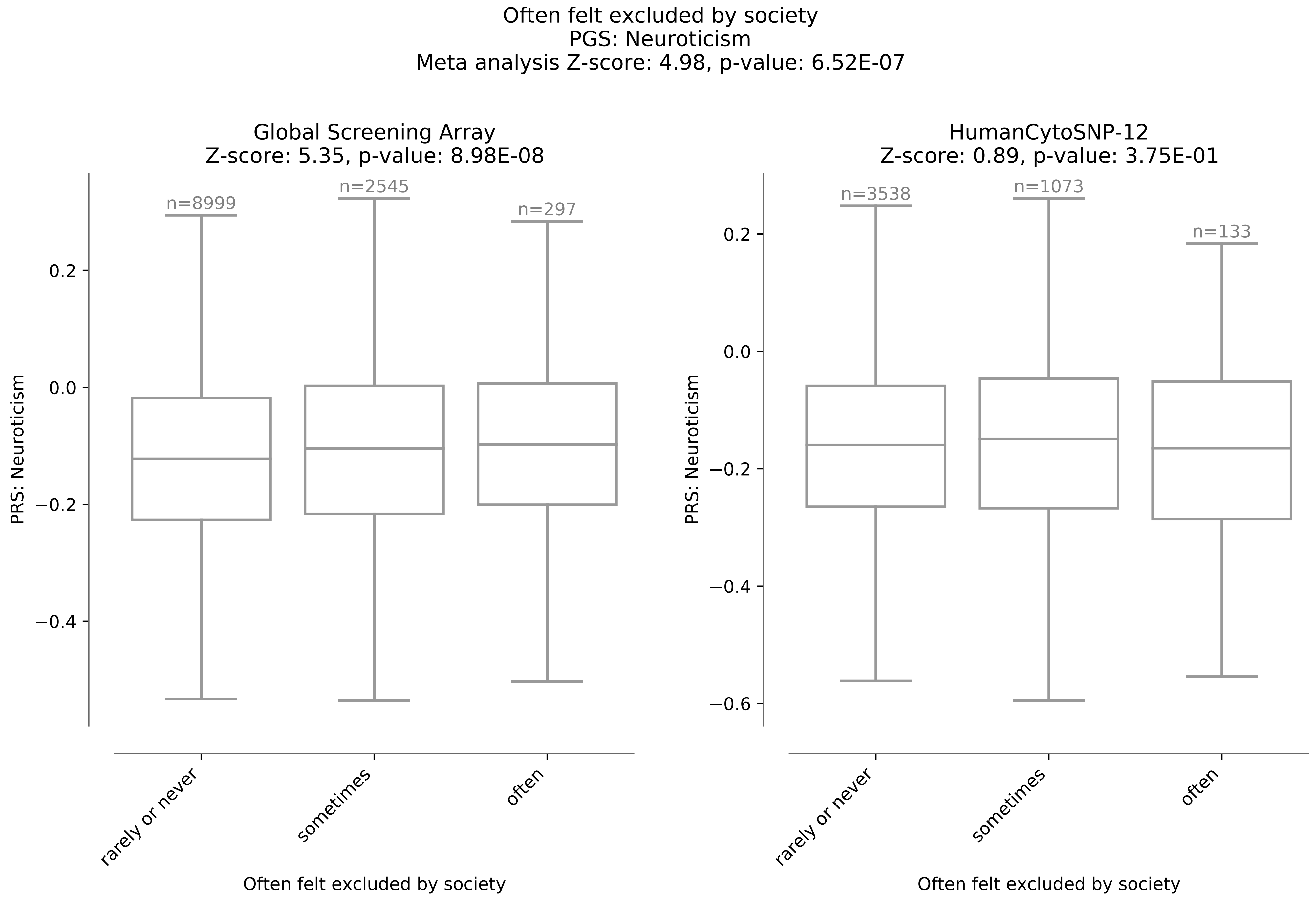

Often felt excluded by society
PGS: Neuroticism
Meta analysis Z-score: 4.98, p-value: 6.52E-07
Global Screening Array
Z-score: 5.35, p-value: 8.98E-08
n=8999
n=2545
n=297
0.2
0.0
−0.2
−0.4
PRS: Neuroticism
rarely or never
sometimes
often
Often felt excluded by society
HumanCytoSNP-12
Z-score: 0.89, p-value: 3.75E-01
n=3538
n=1073
n=133
0.2
0.0
−0.2
−0.4
−0.6
PRS: Neuroticism
rarely or never
sometimes
often
Often felt excluded by society

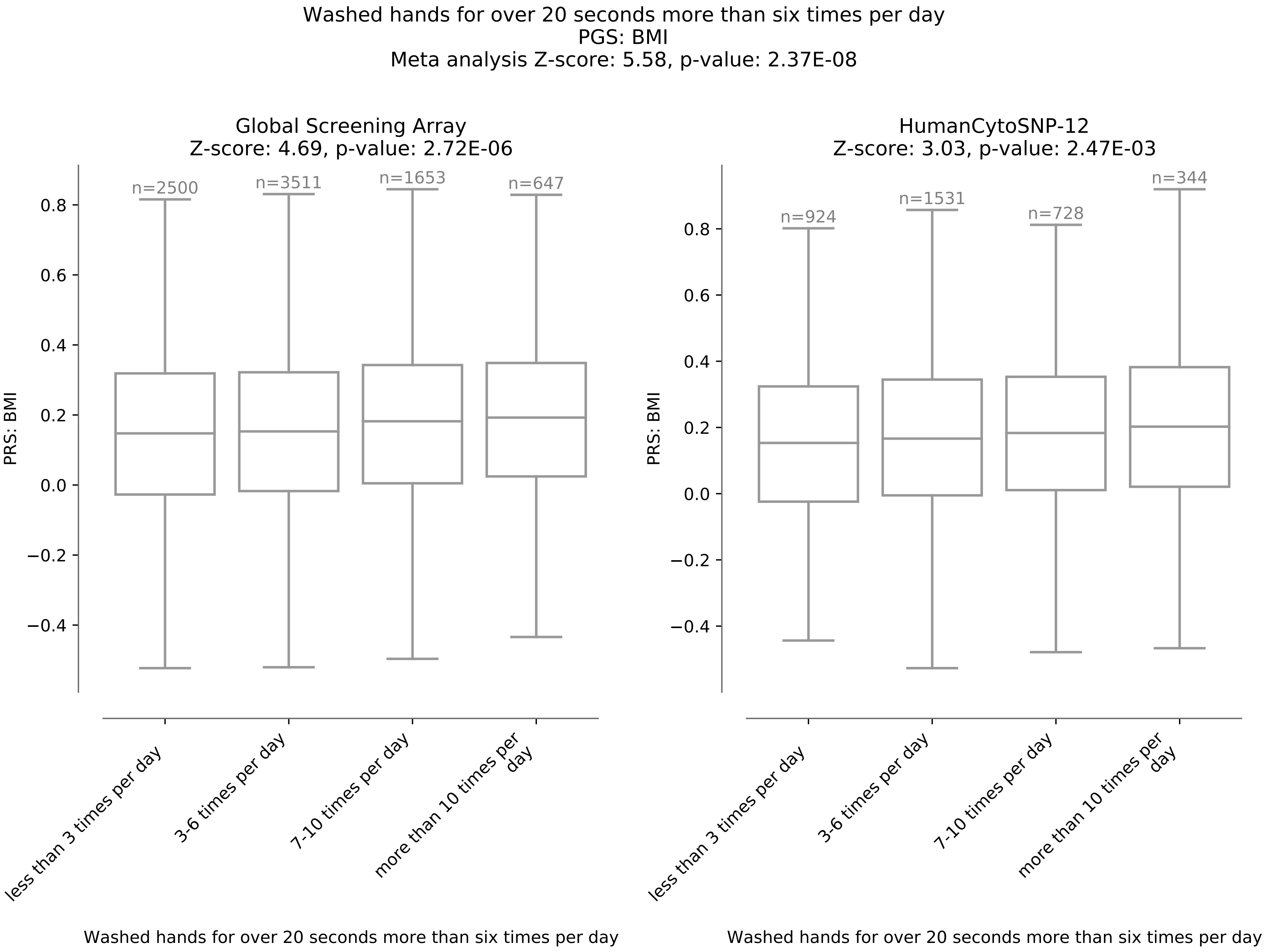
Washed hands for over 20 seconds more than six times per day
PGS: BMI
Meta analysis Z-score: 5.58, p-value: 2.37E-08
Global Screening Array
Z-score: 4.69, p-value: 2.72E-06
HumanCytoSNP-12
Z-score: 3.03, p-value: 2.47E-03
PRS: BMI
n=2500
n=3511
n=1653
n=647
n=924
n=1531
n=728
n=344
0.8
0.6
0.4
0.2
0.0
−0.2
−0.4
less than 3 times per day
3-6 times per day
7-10 times per day
more than 10 times per day
Washed hands for over 20 seconds more than six times per day

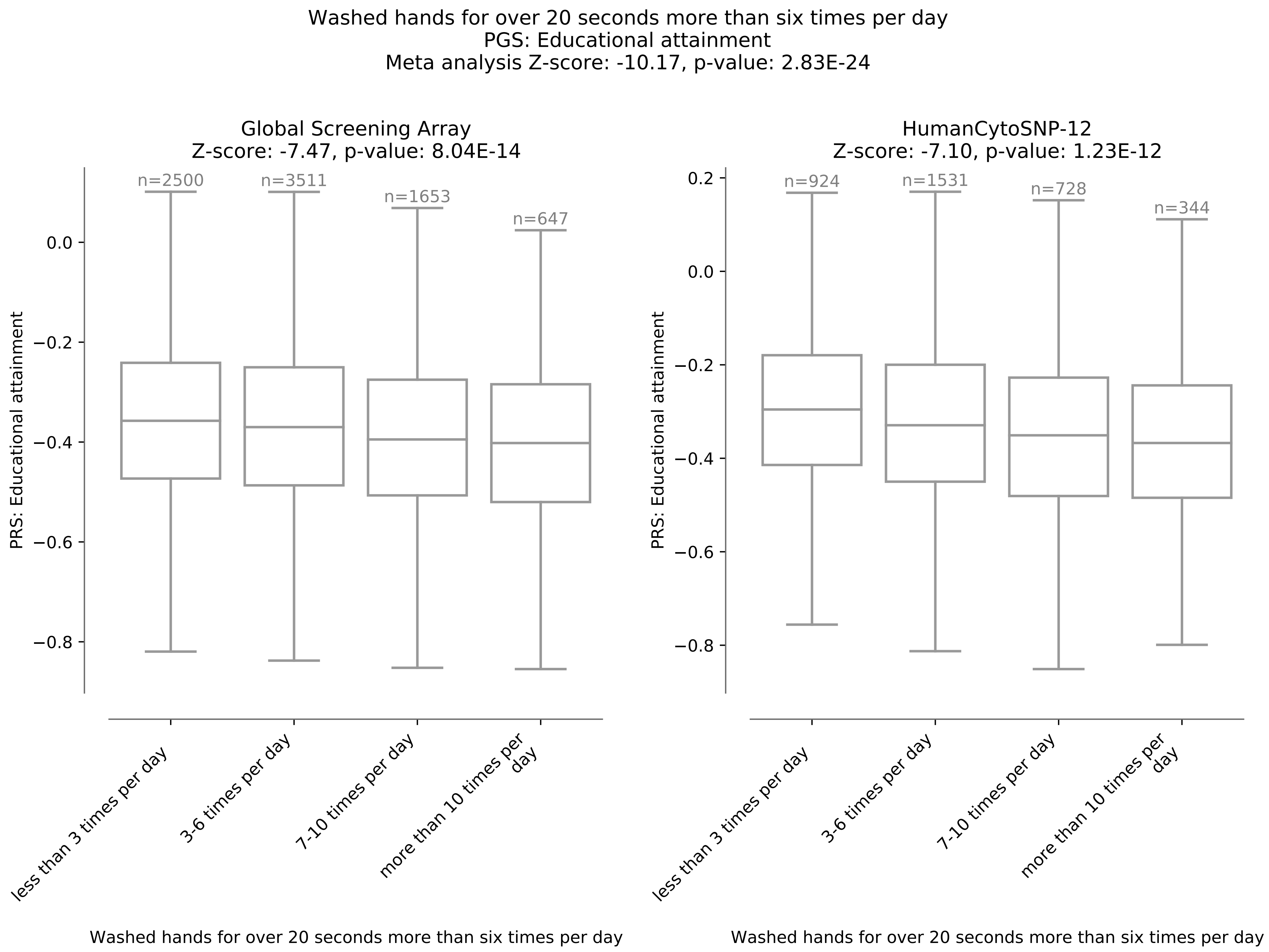

Washed hands for over 20 seconds more than six times per day
PGS: Educational attainment
Meta analysis Z-score: -10.17, p-value: 2.83E-24
Global Screening Array
Z-score: -7.47, p-value: 8.04E-14
n=2500
n=3511
n=1653
n=647
PRS: Educational attainment
0.0
−0.2
−0.4
−0.6
−0.8
less than 3 times per day
3-6 times per day
7-10 times per day
more than 10 times per day
Washed hands for over 20 seconds more than six times per day
HumanCytoSNP-12
Z-score: -7.10, p-value: 1.23E-12
n=924
n=1531
n=728
n=344
PRS: Educational attainment
0.2
0.0
−0.2
−0.4
−0.6
−0.8
less than 3 times per day
3-6 times per day
7-10 times per day
more than 10 times per day
Washed hands for over 20 seconds more than six times per day

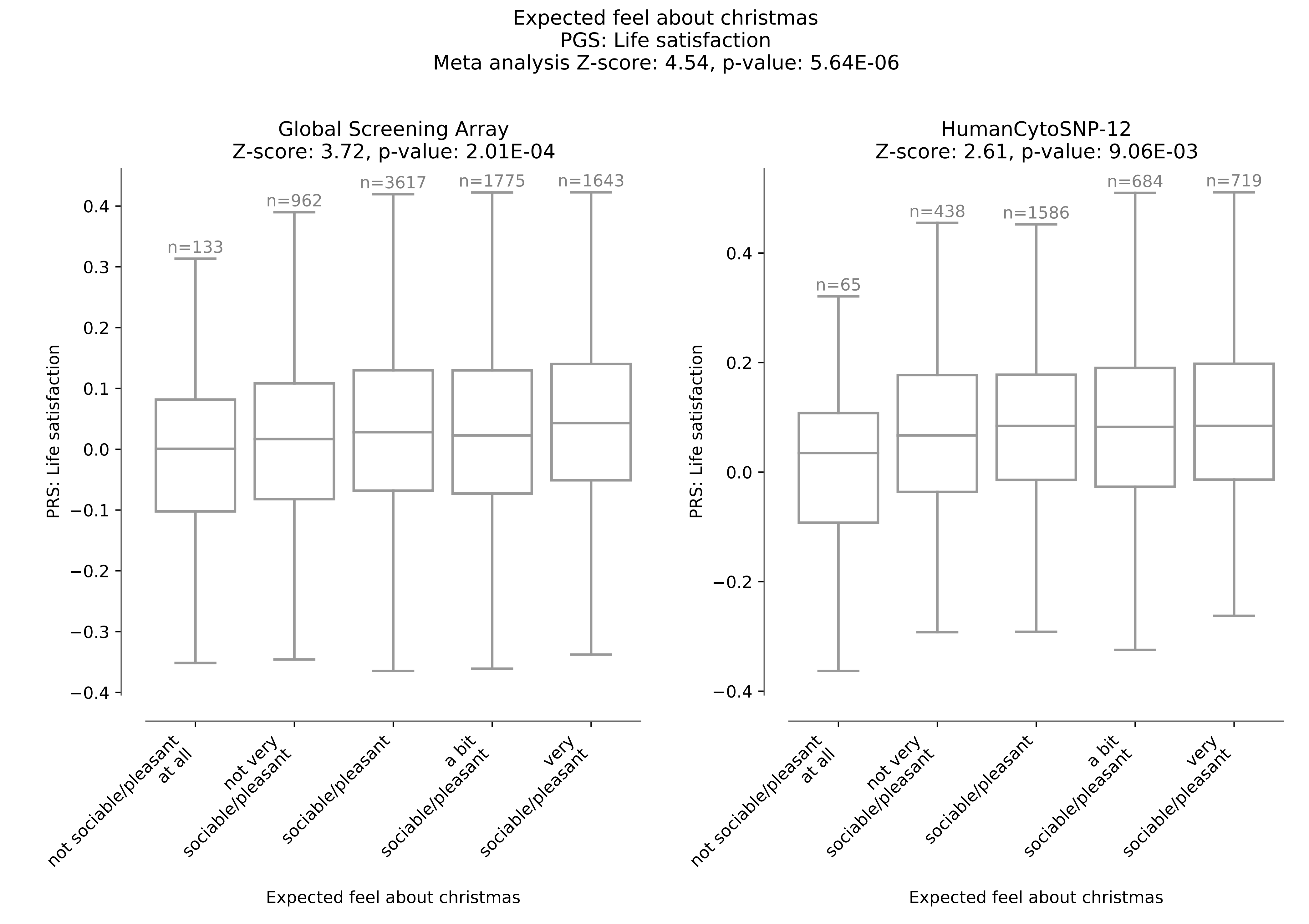

Expected feel about christmas
PGS: Life satisfaction
Meta analysis Z-score: 4.54, p-value: 5.64E-06
Global Screening Array
Z-score: 3.72, p-value: 2.01E-04
HumanCytoSNP-12
Z-score: 2.61, p-value: 9.06E-03
PRS: Life satisfaction
n=133
n=962
n=3617
n=1775
n=1643
n=65
n=438
n=1586
n=684
n=719
not sociable/pleasant at all
not very sociable/pleasant
sociable/pleasant
a bit sociable/pleasant
very sociable/pleasant
Expected feel about christmas

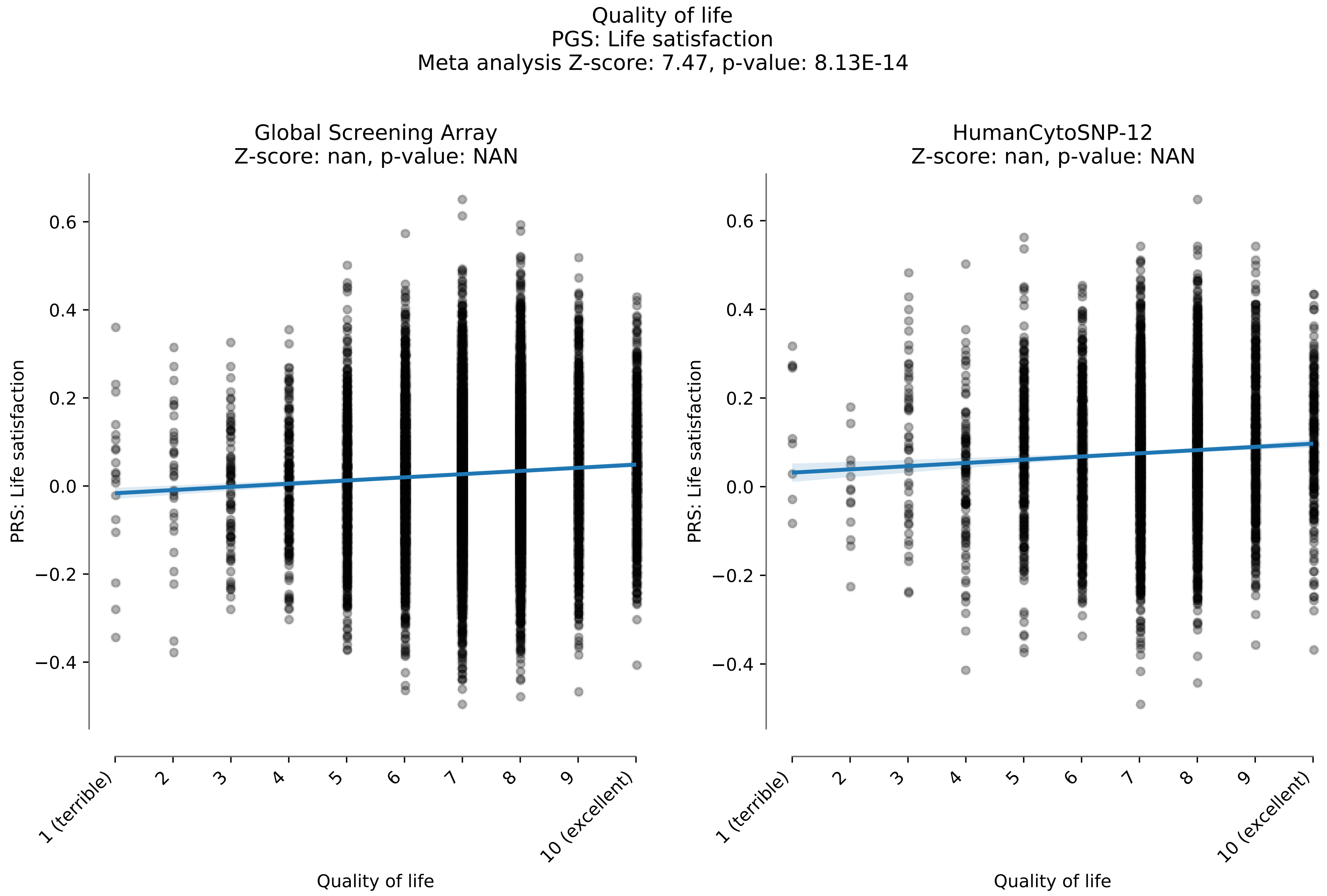

Quality of life
PGS: Life satisfaction
Meta analysis Z-score: 7.47, p-value: 8.13E-14
Global Screening Array
Z-score: nan, p-value: NAN
HumanCytoSNP-12
Z-score: nan, p-value: NAN
PRS: Life satisfaction
0.6
0.4
0.2
0.0
−0.2
−0.4
1 (terrible)
2
3
4
5
6
7
8
9
10 (excellent)
Quality of life

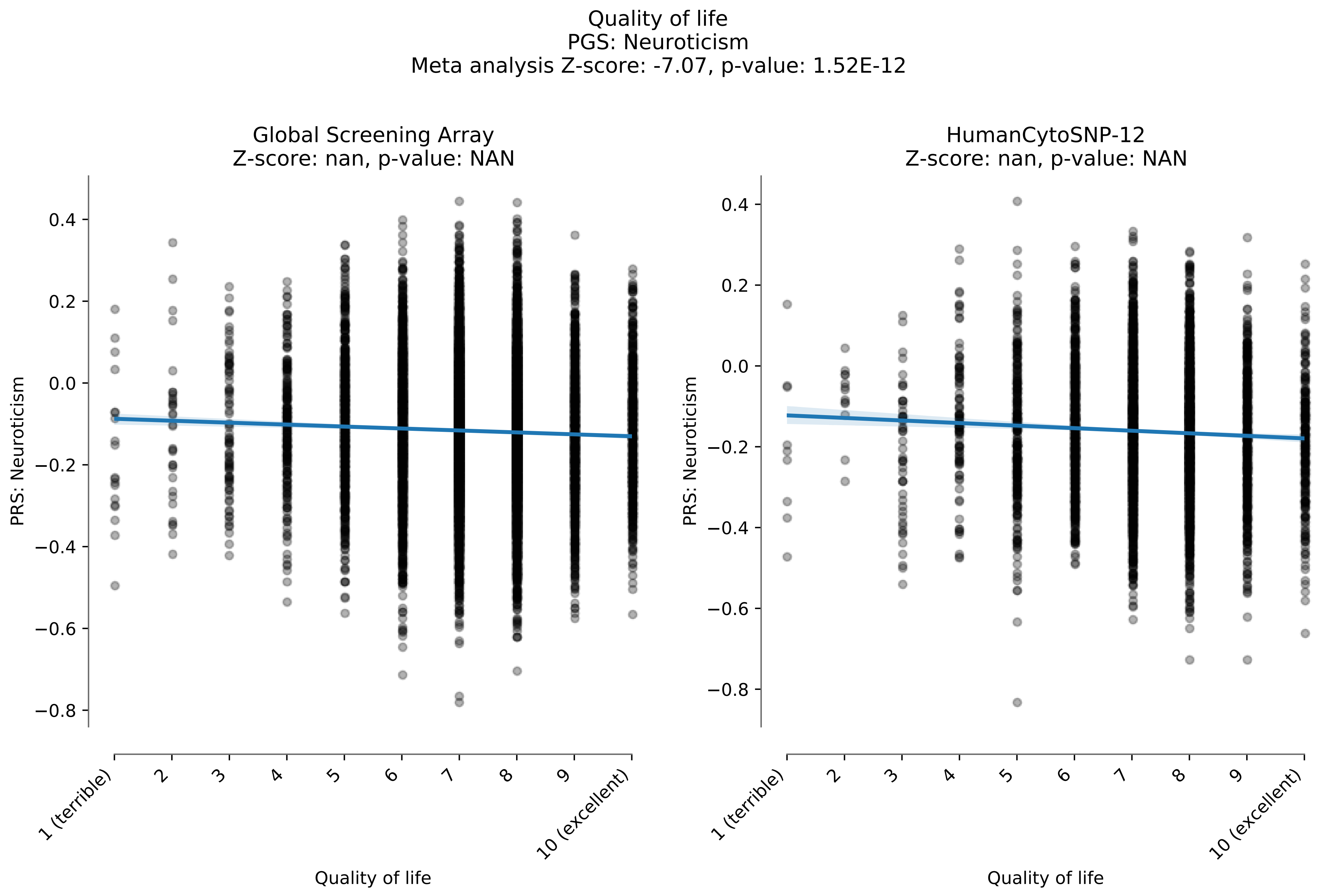
Quality of life
PGS: Neuroticism
Meta analysis Z-score: -7.07, p-value: 1.52E-12
Global Screening Array
Z-score: nan, p-value: NAN
PRS: Neuroticism
0.4
0.2
0.0
−0.2
−0.4
−0.6
−0.8
1 (terrible)
2
3
4
5
6
7
8
9
10 (excellent)
Quality of life
HumanCytoSNP-12
Z-score: nan, p-value: NAN
PRS: Neuroticism
0.4
0.2
0.0
−0.2
−0.4
−0.6
−0.8
1 (terrible)
2
3
4
5
6
7
8
9
10 (excellent)
Quality of life

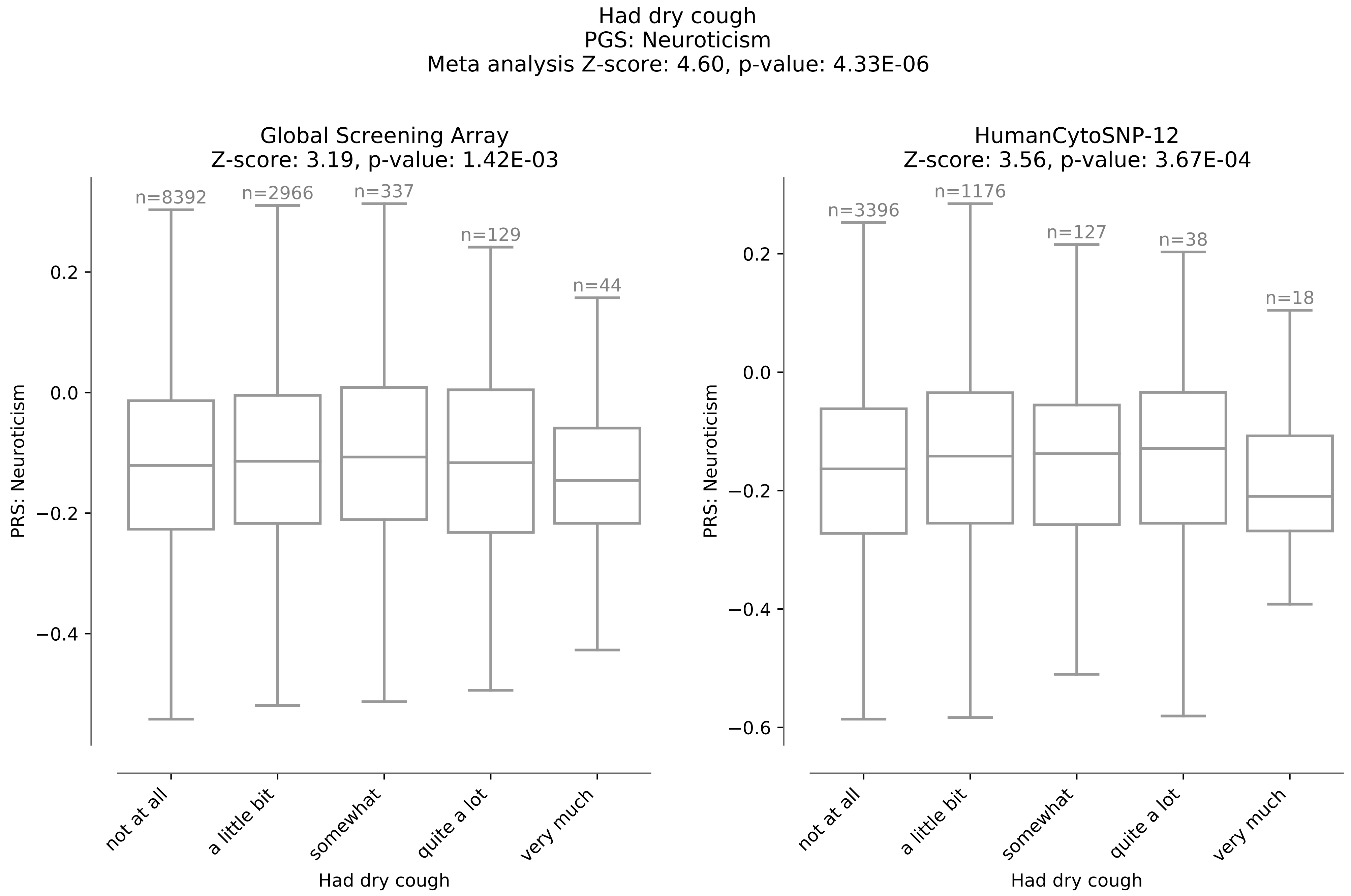
Had dry cough
PGS: Neuroticism
Meta analysis Z-score: 4.60, p-value: 4.33E-06
Global Screening Array
Z-score: 3.19, p-value: 1.42E-03
n=8392
n=2966
n=337
n=129
n=44
0.2
0.0
−0.2
−0.4
PRS: Neuroticism
not at all
a little bit
somewhat
quite a lot
very much
Had dry cough
HumanCytoSNP-12
Z-score: 3.56, p-value: 3.67E-04
n=3396
n=1176
n=127
n=38
n=18
0.2
0.0
−0.2
−0.4
−0.6
PRS: Neuroticism
not at all
a little bit
somewhat
quite a lot
very much
Had dry cough

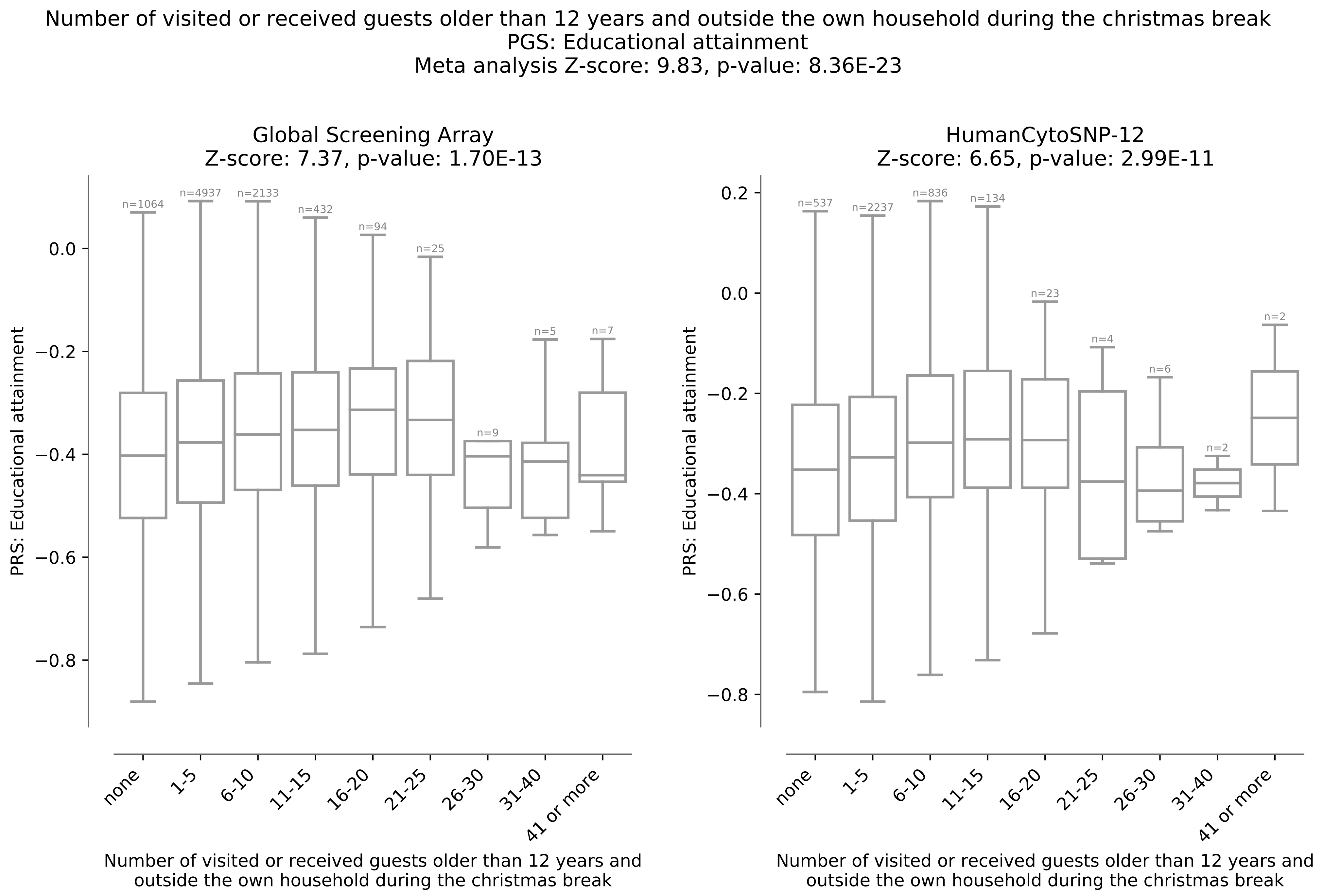

Number of visited or received guests older than 12 years and outside the own household during the christmas break
PGS: Educational attainment
Meta analysis Z-score: 9.83, p-value: 8.36E-23
Global Screening Array
Z-score: 7.37, p-value: 1.70E-13
HumanCytoSNP-12
Z-score: 6.65, p-value: 2.99E-11
PRS: Educational attainment
n=1064
n=4937
n=2133
n=432
n=94
n=25
n=9
n=5
n=7
n=537
n=2237
n=836
n=134
n=23
n=4
n=6
n=2
n=2
0.2
0.0
−0.2
−0.4
−0.6
−0.8
none
1-5
6-10
11-15
16-20
21-25
26-30
31-40
41 or more
Number of visited or received guests older than 12 years and outside the own household during the christmas break

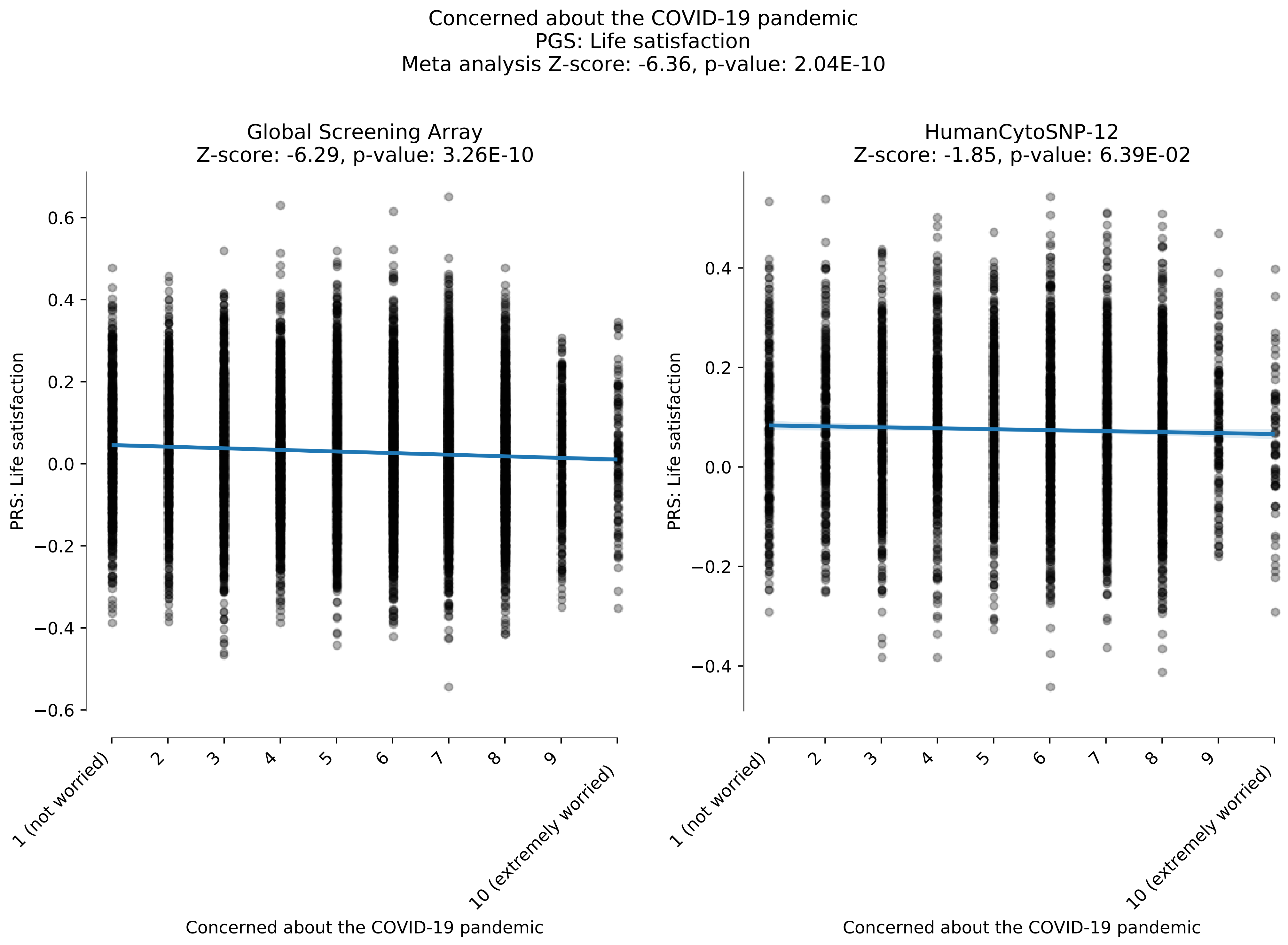
Concerned about the COVID-19 pandemic
PGS: Life satisfaction
Meta analysis Z-score: -6.36, p-value: 2.04E-10
Global Screening Array
Z-score: -6.29, p-value: 3.26E-10
HumanCytoSNP-12
Z-score: -1.85, p-value: 6.39E-02
PRS: Life satisfaction
0.6
0.4
0.2
0.0
−0.2
−0.4
−0.6
1 (not worried)
2
3
4
5
6
7
8
9
10 (extremely worried)
Concerned about the COVID-19 pandemic

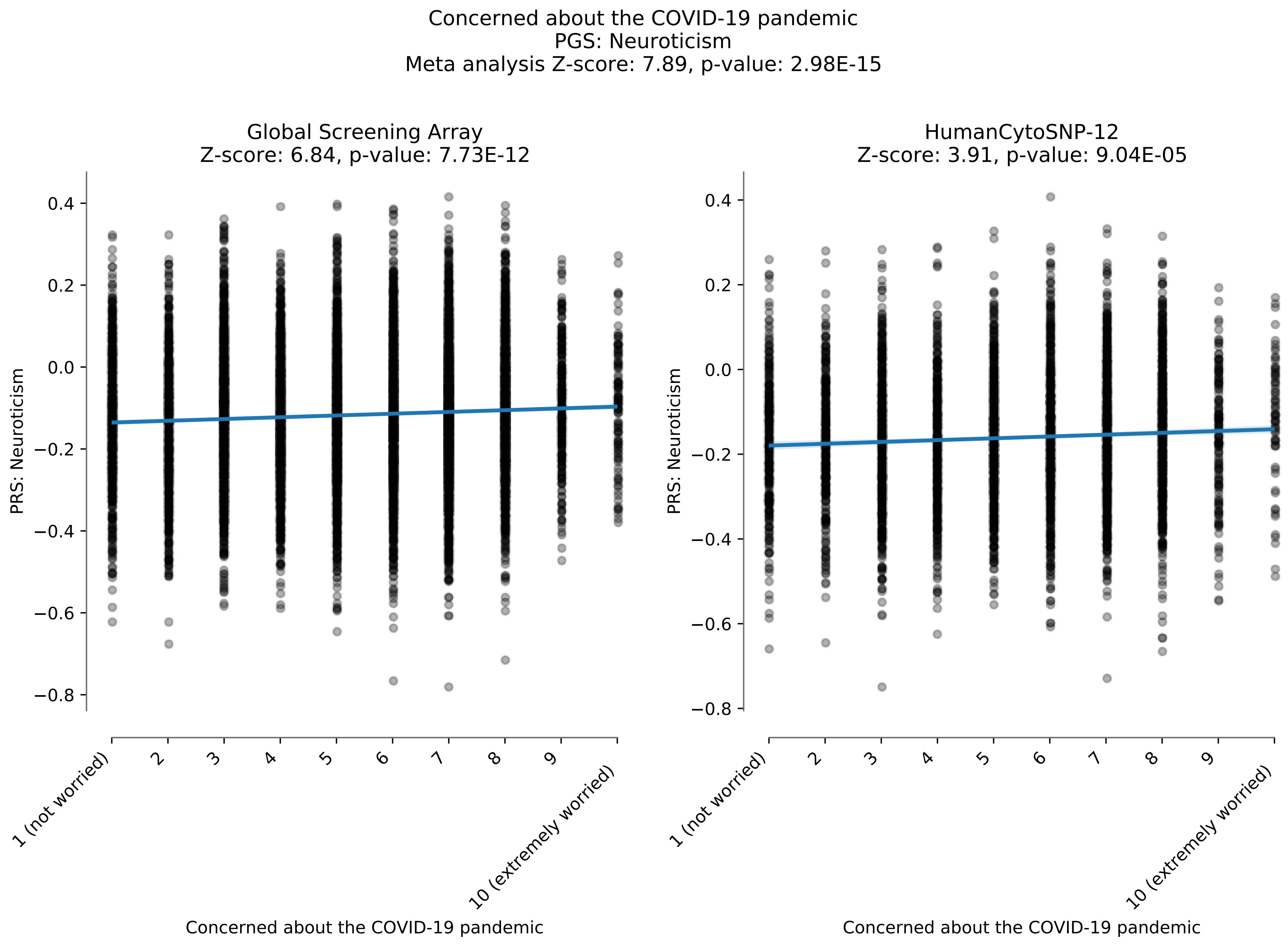
Concerned about the COVID-19 pandemic
PGS: Neuroticism
Meta analysis Z-score: 7.89, p-value: 2.98E-15
Global Screening Array
Z-score: 6.84, p-value: 7.73E-12
HumanCytoSNP-12
Z-score: 3.91, p-value: 9.04E-05
PRS: Neuroticism
0.4
0.2
0.0
−0.2
−0.4
−0.6
−0.8
1 (not worried)
2
3
4
5
6
7
8
9
10 (extremely worried)
Concerned about the COVID-19 pandemic

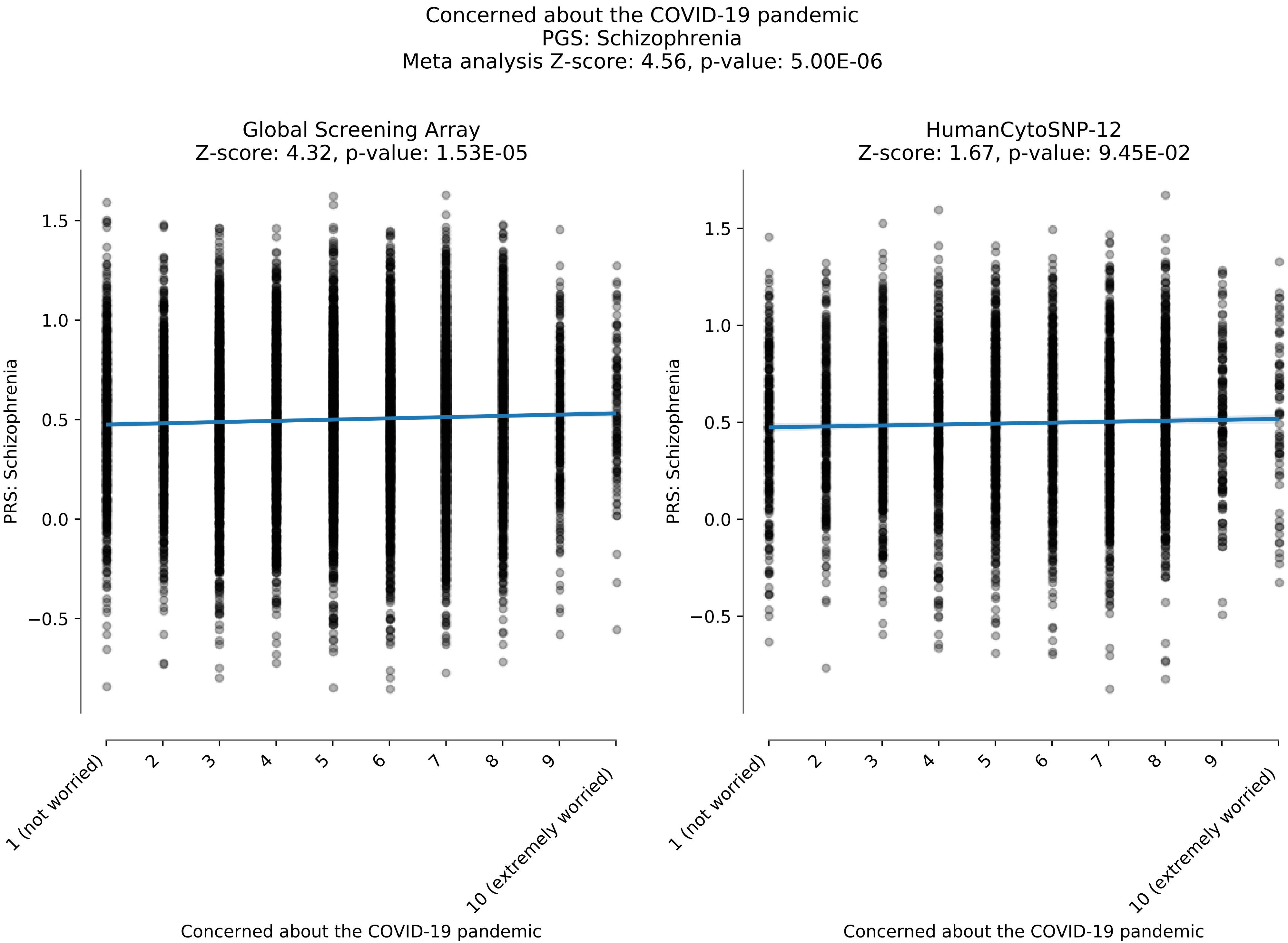
Concerned about the COVID-19 pandemic
PGS: Schizophrenia
Meta analysis Z-score: 4.56, p-value: 5.00E-06
Global Screening Array
Z-score: 4.32, p-value: 1.53E-05
HumanCytoSNP-12
Z-score: 1.67, p-value: 9.45E-02
PRS: Schizophrenia
1.5
1.0
0.5
0.0
−0.5
1 (not worried)
2
3
4
5
6
7
8
9
10 (extremely worried)
Concerned about the COVID-19 pandemic

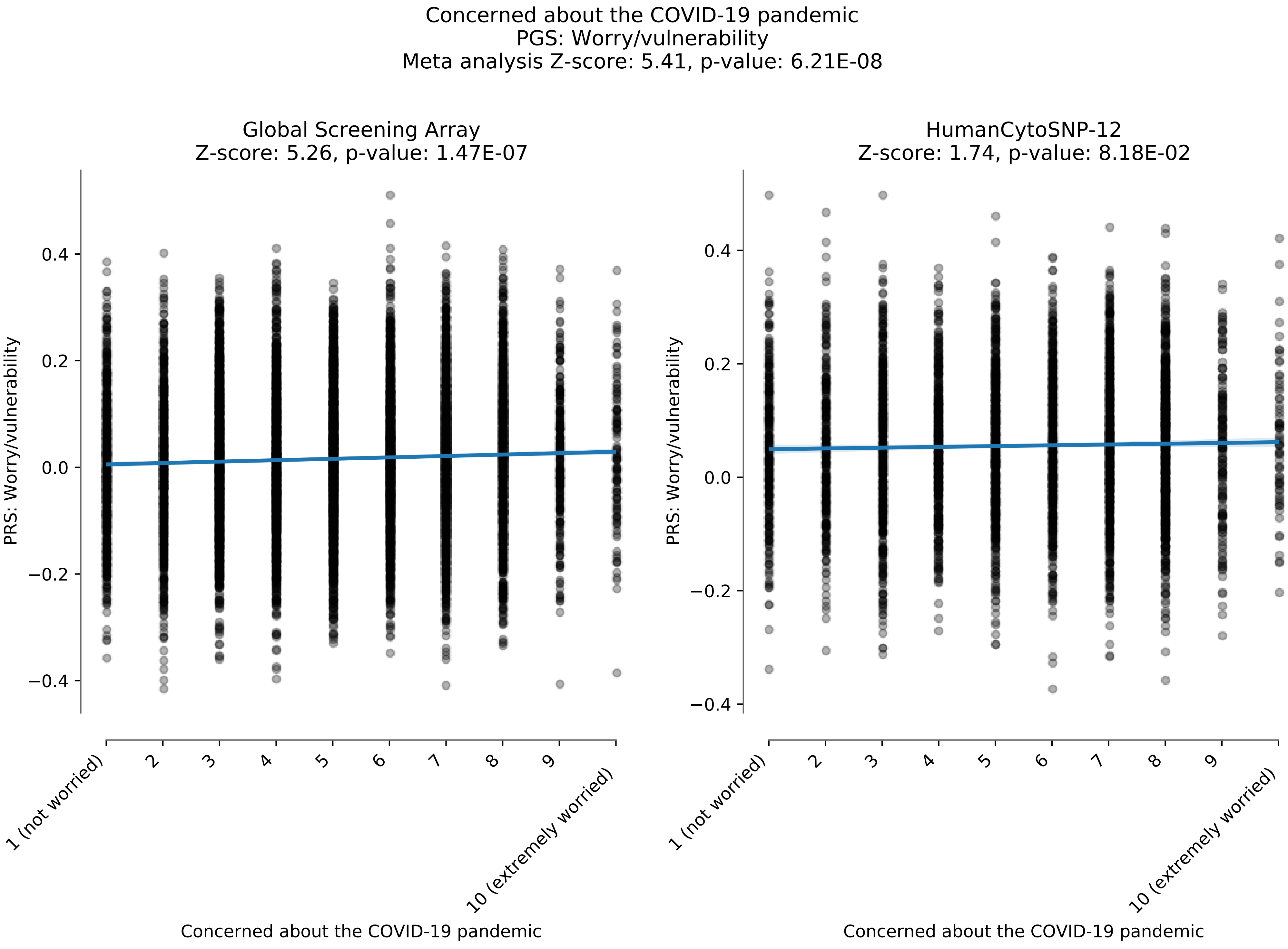
Concerned about the COVID-19 pandemic
PGS: Worry/vulnerability
Meta analysis Z-score: 5.41, p-value: 6.21E-08
Global Screening Array
Z-score: 5.26, p-value: 1.47E-07
HumanCytoSNP-12
Z-score: 1.74, p-value: 8.18E-02
PRS: Worry/vulnerability
0.4
0.2
0.0
−0.2
−0.4
1 (not worried)
2
3
4
5
6
7
8
9
10 (extremely worried)
Concerned about the COVID-19 pandemic

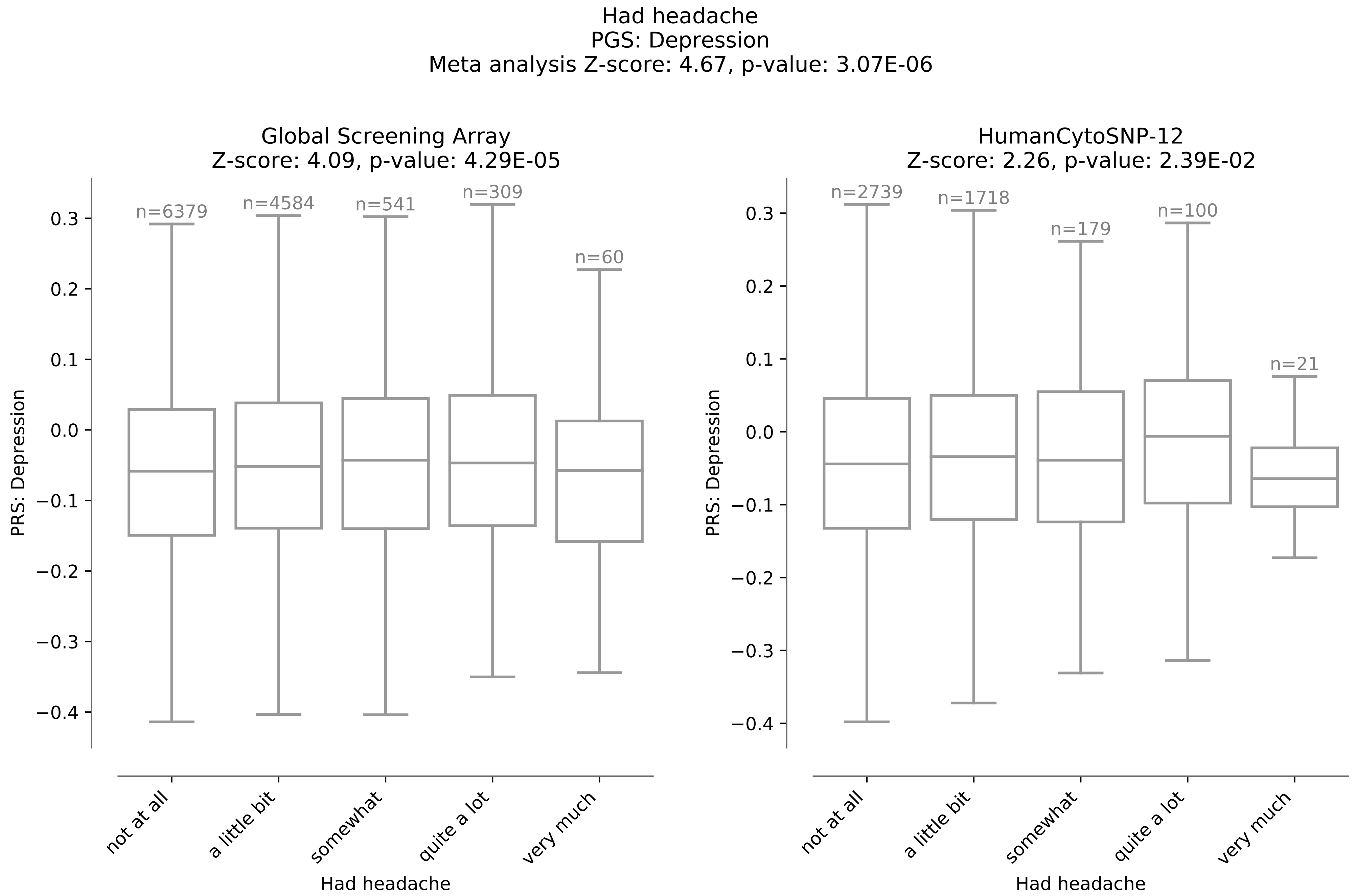

Had headache
PGS: Depression
Meta analysis Z-score: 4.67, p-value: 3.07E-06
Global Screening Array
Z-score: 4.09, p-value: 4.29E-05
HumanCytoSNP-12
Z-score: 2.26, p-value: 2.39E-02
PRS: Depression
Had headache
n=6379
n=4584
n=541
n=309
n=60
n=2739
n=1718
n=179
n=100
n=21
not at all
a little bit
somewhat
quite a lot
very much
0.3
0.2
0.1
0.0
−0.1
−0.2
−0.3
−0.4

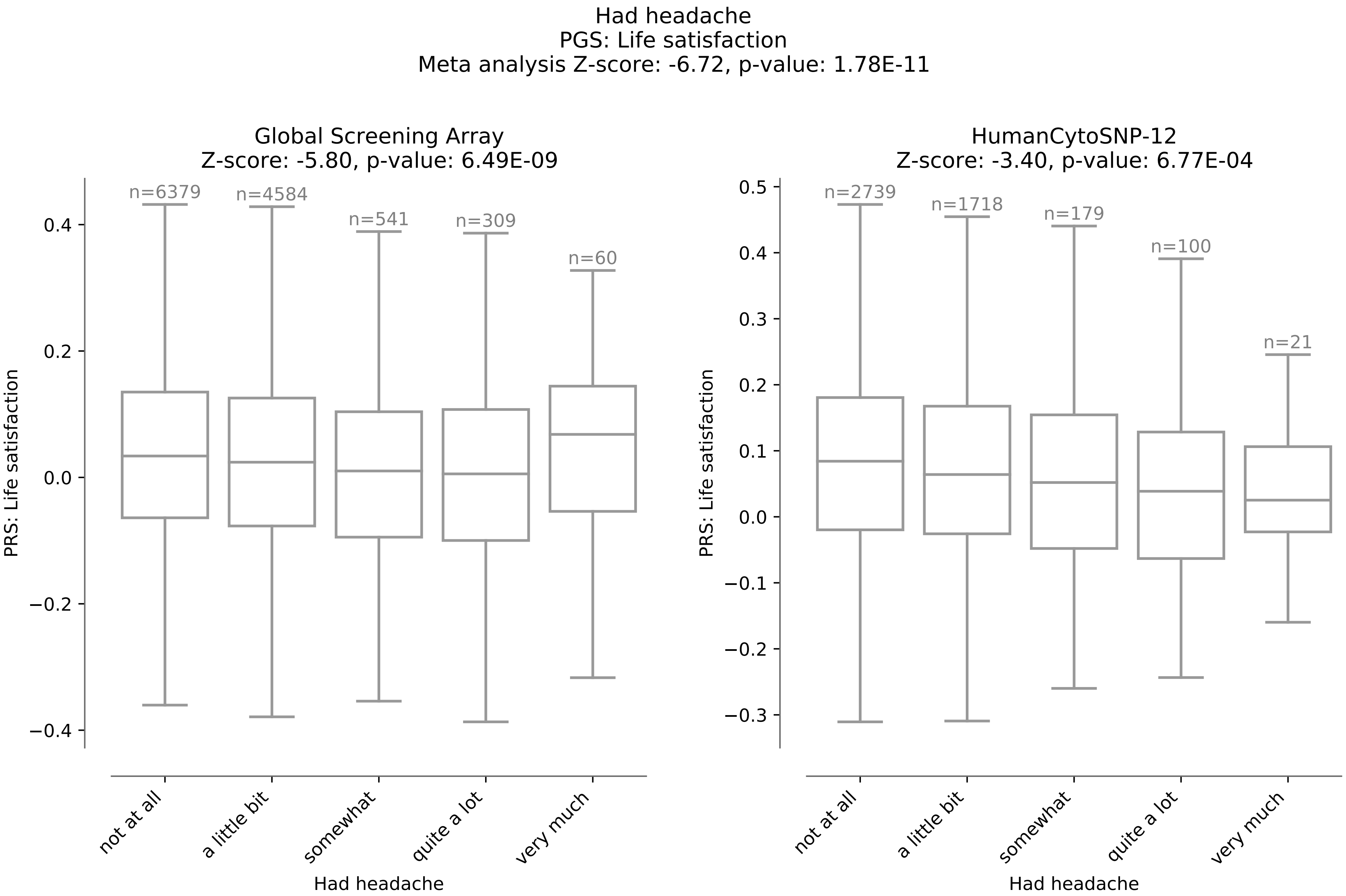
Had headache
PGS: Life satisfaction
Meta analysis Z-score: -6.72, p-value: 1.78E-11
Global Screening Array
Z-score: -5.80, p-value: 6.49E-09
n=6379
n=4584
n=541
n=309
n=60
PRS: Life satisfaction
0.4
0.2
0.0
−0.2
−0.4
not at all
a little bit
somewhat
quite a lot
very much
Had headache
HumanCytoSNP-12
Z-score: -3.40, p-value: 6.77E-04
n=2739
n=1718
n=179
n=100
n=21
PRS: Life satisfaction
0.5
0.4
0.3
0.2
0.1
0.0
−0.1
−0.2
−0.3
not at all
a little bit
somewhat
quite a lot
very much
Had headache

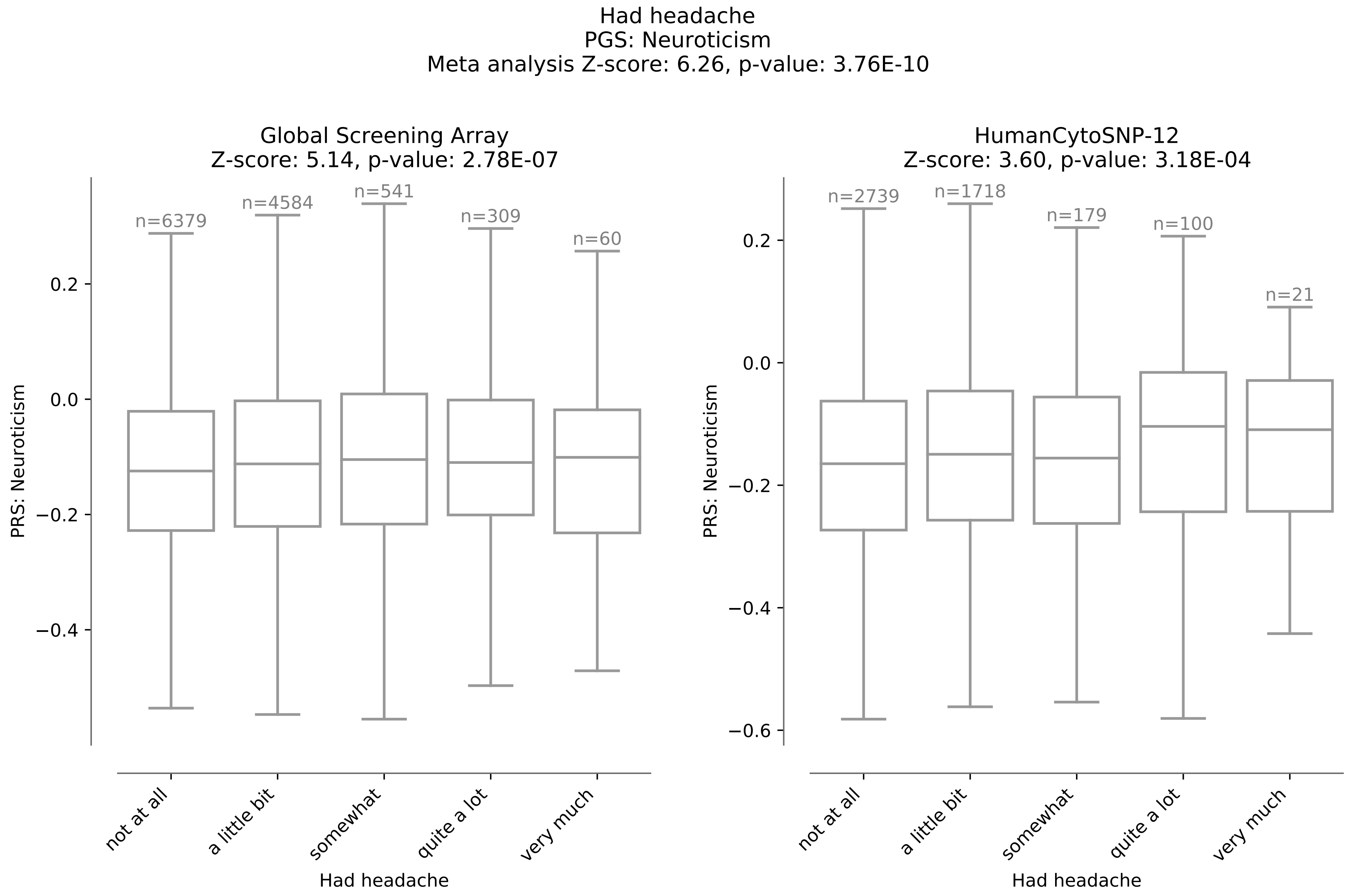
Had headache
PGS: Neuroticism
Meta analysis Z-score: 6.26, p-value: 3.76E-10
Global Screening Array
Z-score: 5.14, p-value: 2.78E-07
n=6379
n=4584
n=541
n=309
n=60
0.2
0.0
−0.2
−0.4
PRS: Neuroticism
not at all
a little bit
somewhat
quite a lot
very much
Had headache
HumanCytoSNP-12
Z-score: 3.60, p-value: 3.18E-04
n=2739
n=1718
n=179
n=100
n=21
0.2
0.0
−0.2
−0.4
−0.6
PRS: Neuroticism
not at all
a little bit
somewhat
quite a lot
very much
Had headache

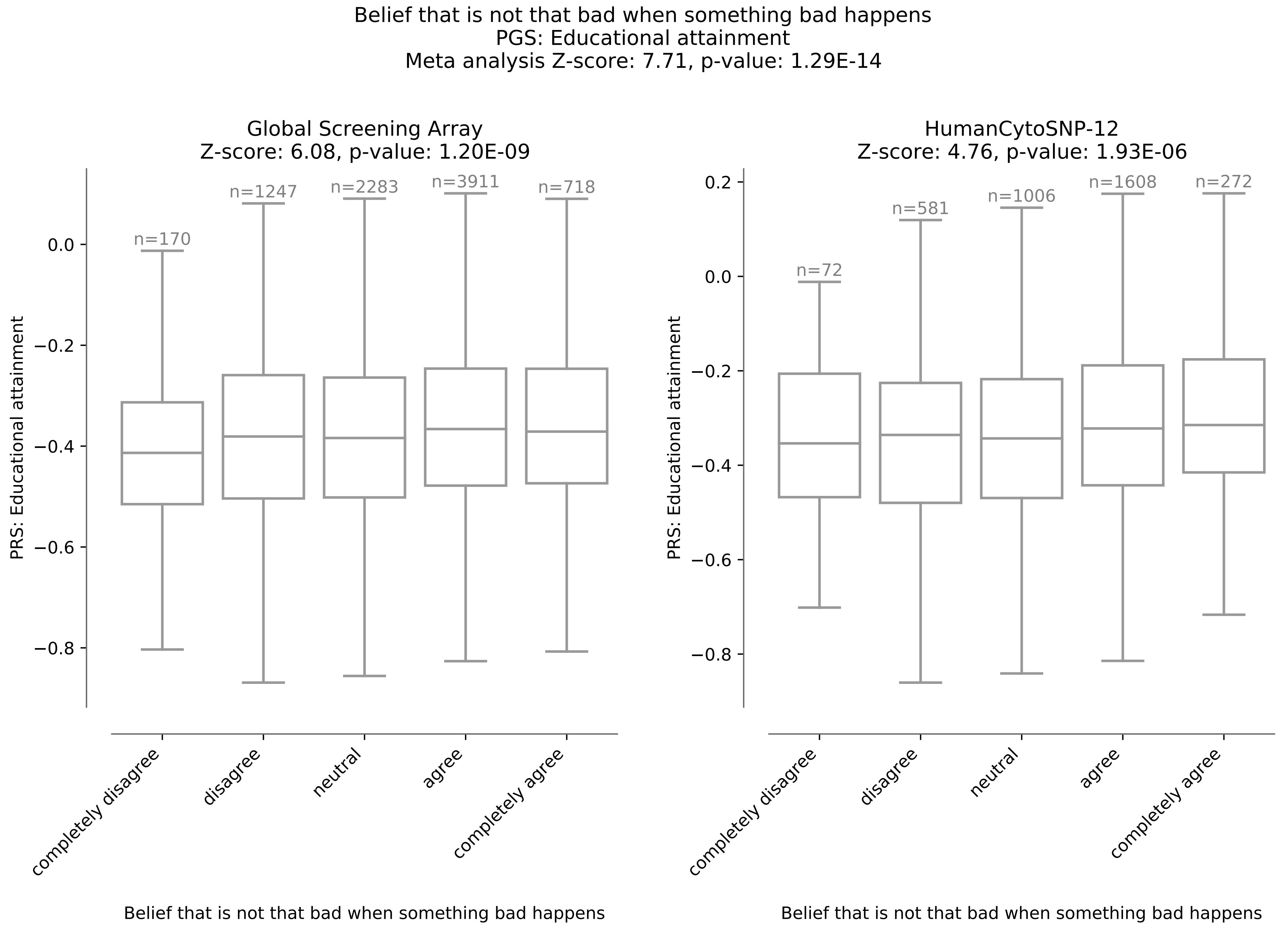

Belief that is not that bad when something bad happens
PGS: Educational attainment
Meta analysis Z-score: 7.71, p-value: 1.29E-14
Global Screening Array
Z-score: 6.08, p-value: 1.20E-09
PRS: Educational attainment
n=170
n=1247
n=2283
n=3911
n=718
0.0
−0.2
−0.4
−0.6
−0.8
completely disagree
disagree
neutral
agree
completely agree
Belief that is not that bad when something bad happens
HumanCytoSNP-12
Z-score: 4.76, p-value: 1.93E-06
PRS: Educational attainment
n=72
n=581
n=1006
n=1608
n=272
0.2
0.0
−0.2
−0.4
−0.6
−0.8
completely disagree
disagree
neutral
agree
completely agree
Belief that is not that bad when something bad happens

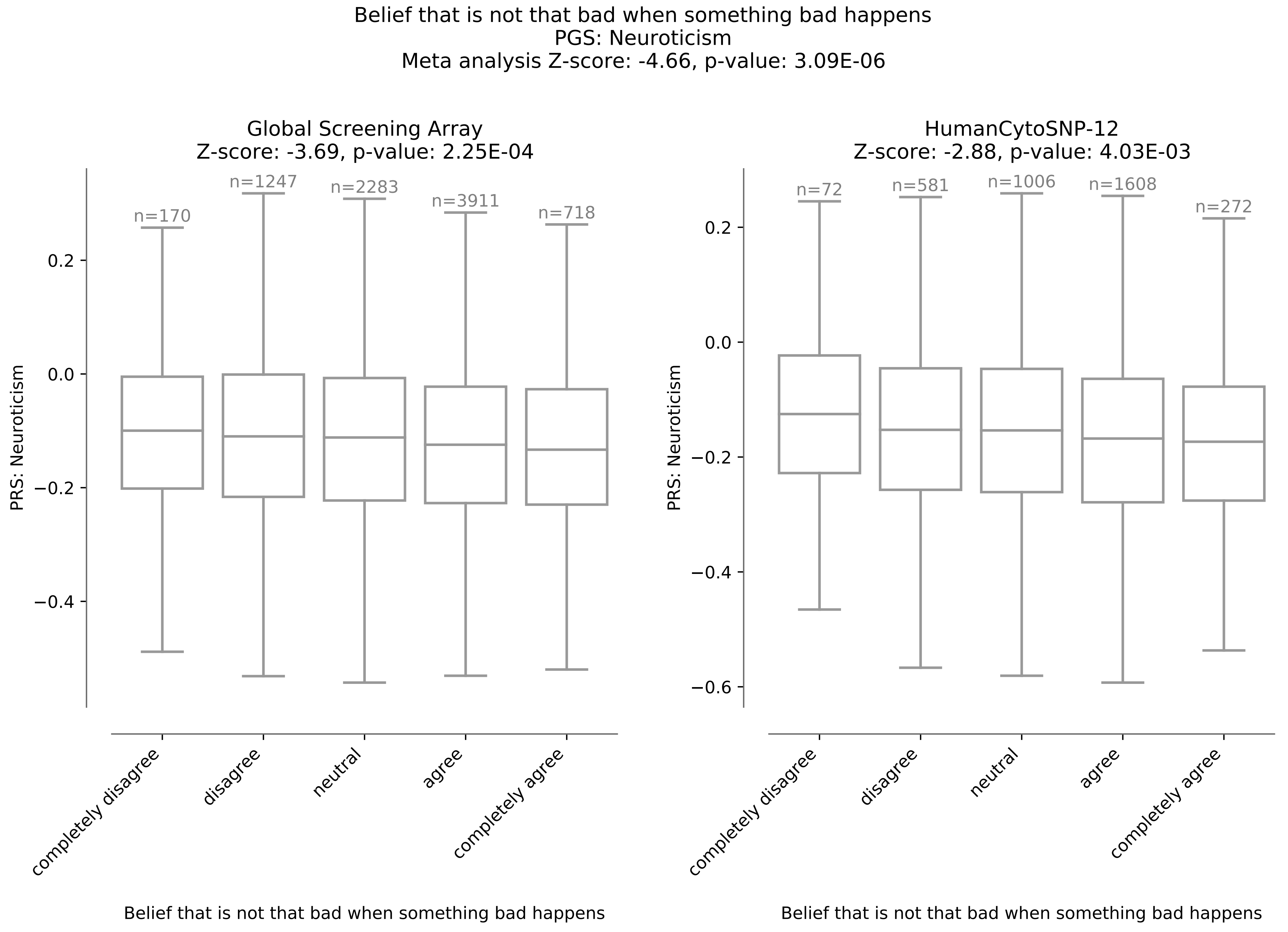
Belief that is not that bad when something bad happens
PGS: Neuroticism
Meta analysis Z-score: -4.66, p-value: 3.09E-06
Global Screening Array
Z-score: -3.69, p-value: 2.25E-04
n=170
n=1247
n=2283
n=3911
n=718
PRS: Neuroticism
0.2
0.0
−0.2
−0.4
completely disagree
disagree
neutral
agree
completely agree
Belief that is not that bad when something bad happens
HumanCytoSNP-12
Z-score: -2.88, p-value: 4.03E-03
n=72
n=581
n=1006
n=1608
n=272
PRS: Neuroticism
0.2
0.0
−0.2
−0.4
−0.6
completely disagree
disagree
neutral
agree
completely agree
Belief that is not that bad when something bad happens

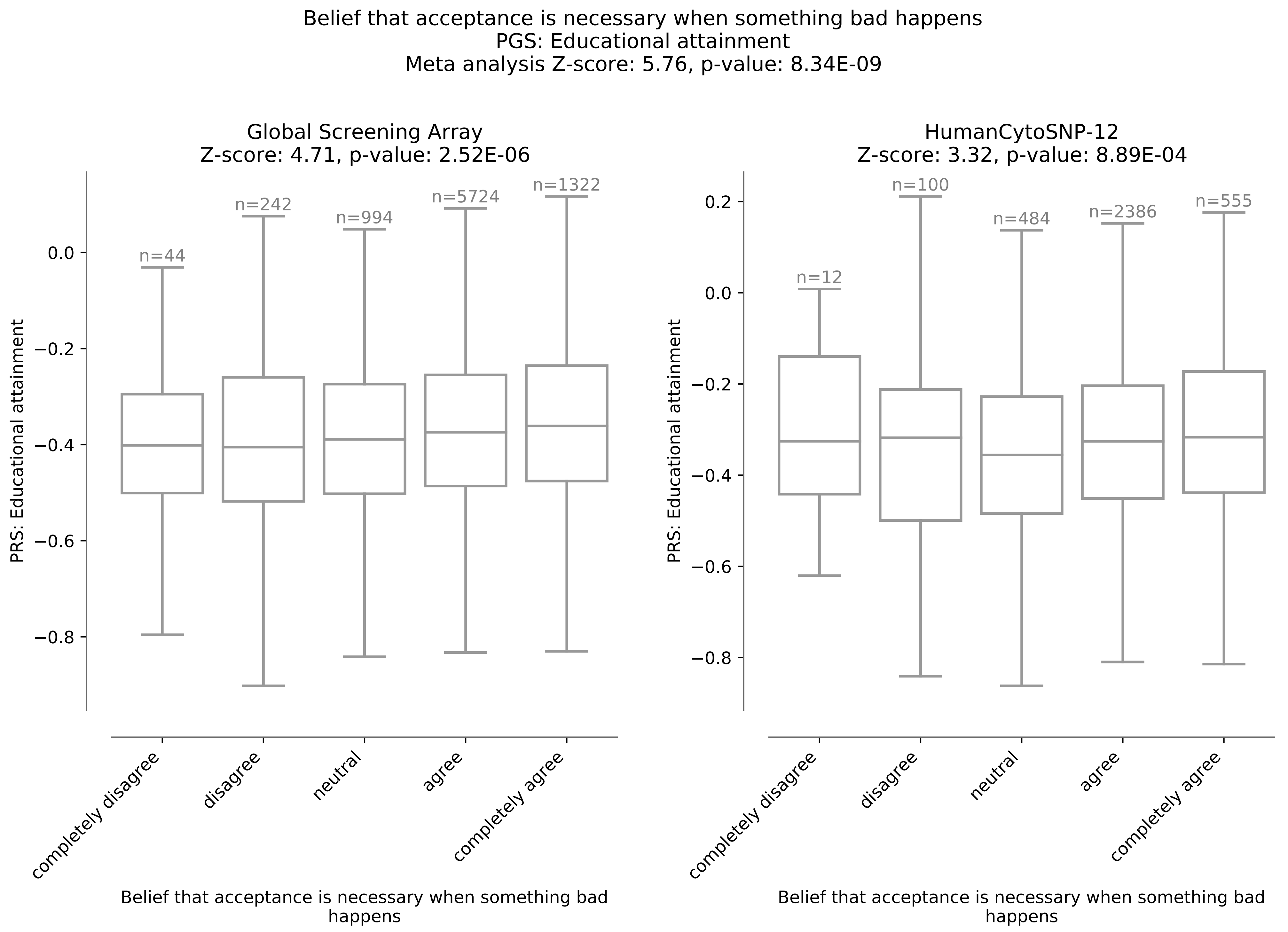
Belief that acceptance is necessary when something bad happens
PGS: Educational attainment
Meta analysis Z-score: 5.76, p-value: 8.34E-09
Global Screening Array
Z-score: 4.71, p-value: 2.52E-06
HumanCytoSNP-12
Z-score: 3.32, p-value: 8.89E-04
PRS: Educational attainment
n=44
n=242
n=994
n=5724
n=1322
n=12
n=100
n=484
n=2386
n=555
0.2
0.0
−0.2
−0.4
−0.6
−0.8
completely disagree
disagree
neutral
agree
completely agree
Belief that acceptance is necessary when something bad happens

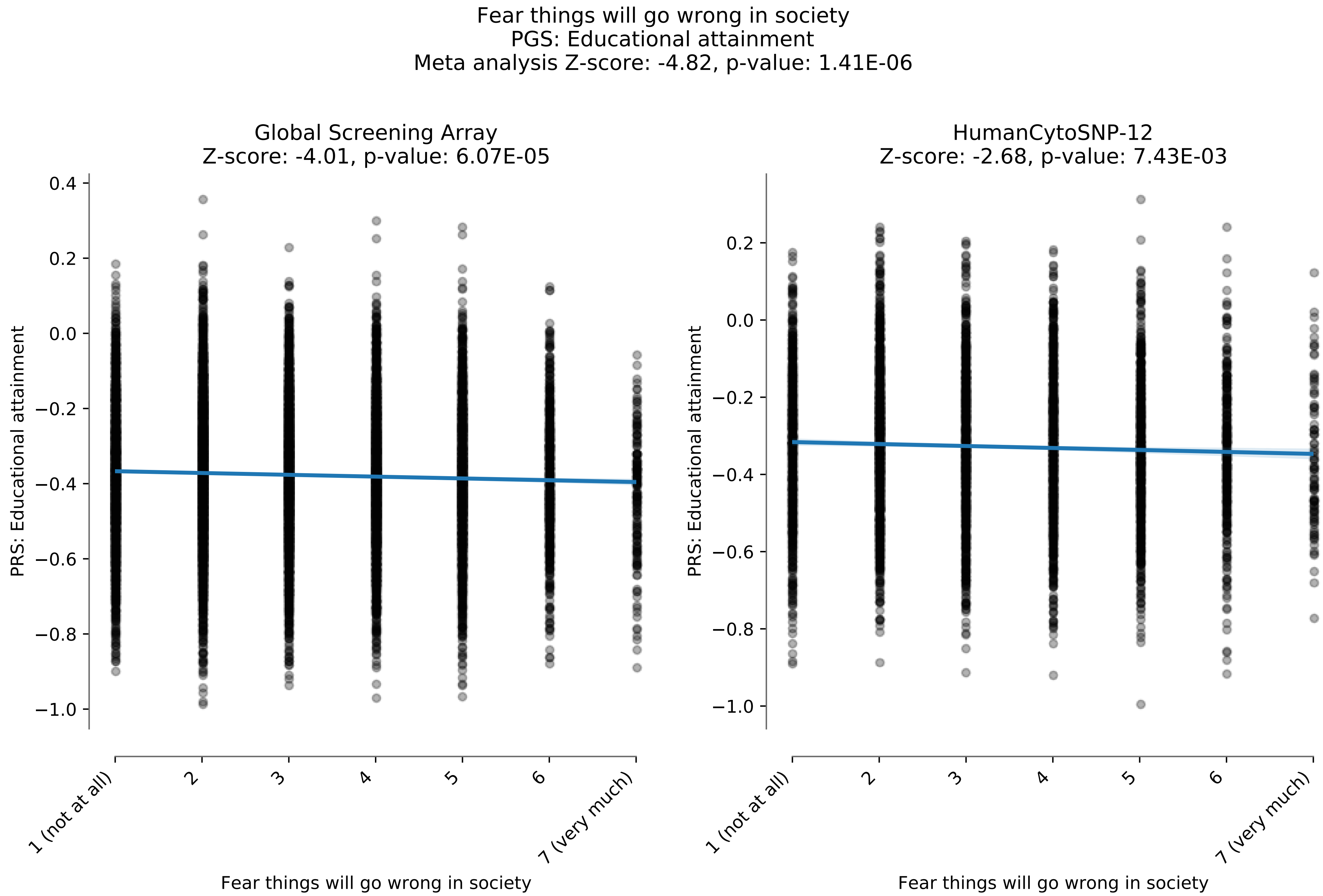
Fear things will go wrong in society
PGS: Educational attainment
Meta analysis Z-score: -4.82, p-value: 1.41E-06
Global Screening Array
Z-score: -4.01, p-value: 6.07E-05
PRS: Educational attainment
0.4
0.2
0.0
−0.2
−0.4
−0.6
−0.8
−1.0
1 (not at all)
2
3
4
5
6
7 (very much)
Fear things will go wrong in society
HumanCytoSNP-12
Z-score: -2.68, p-value: 7.43E-03
PRS: Educational attainment
0.2
0.0
−0.2
−0.4
−0.6
−0.8
−1.0
1 (not at all)
2
3
4
5
6
7 (very much)
Fear things will go wrong in society

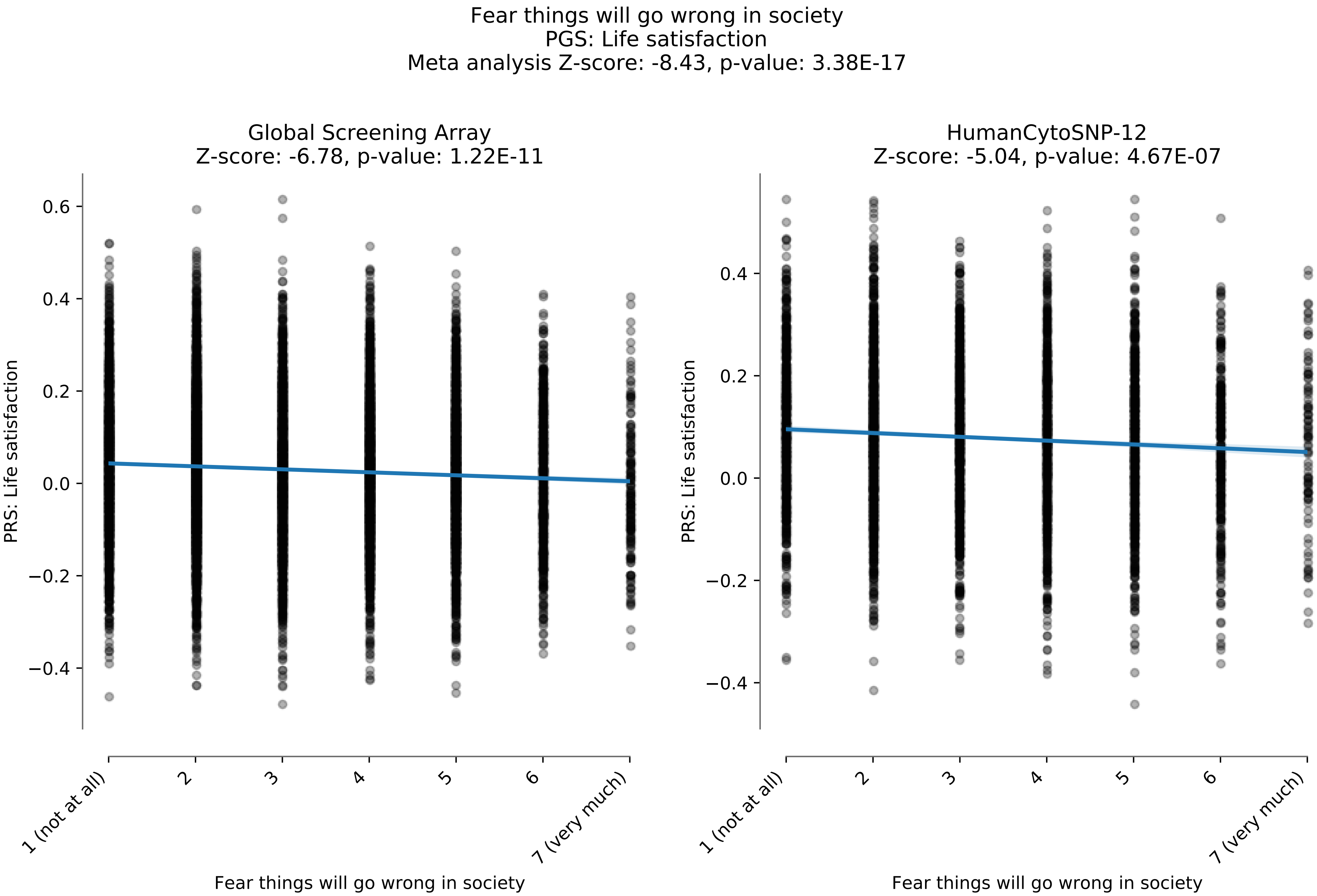
Fear things will go wrong in society
PGS: Life satisfaction
Meta analysis Z-score: -8.43, p-value: 3.38E-17
Global Screening Array
Z-score: -6.78, p-value: 1.22E-11
HumanCytoSNP-12
Z-score: -5.04, p-value: 4.67E-07
PRS: Life satisfaction
0.6
0.4
0.2
0.0
−0.2
−0.4
1 (not at all)
2
3
4
5
6
7 (very much)
Fear things will go wrong in society

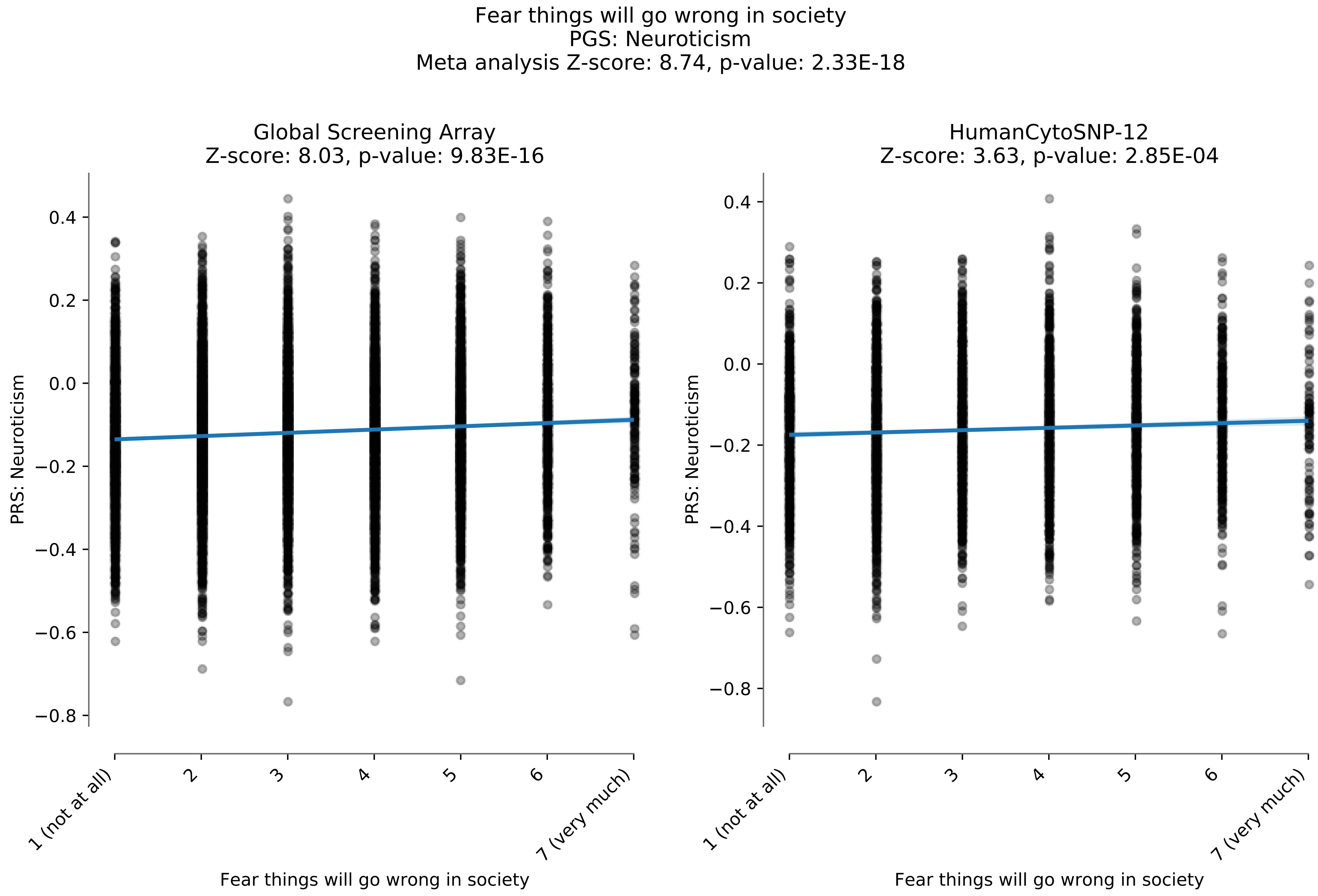

Fear things will go wrong in society
PGS: Neuroticism
Meta analysis Z-score: 8.74, p-value: 2.33E-18
Global Screening Array
Z-score: 8.03, p-value: 9.83E-16
HumanCytoSNP-12
Z-score: 3.63, p-value: 2.85E-04
PRS: Neuroticism
0.4
0.2
0.0
−0.2
−0.4
−0.6
−0.8
1 (not at all)
2
3
4
5
6
7 (very much)
Fear things will go wrong in society

Being afraid of the important ground rights more easily swept aside in the future
PGS: BMI
Meta analysis Z-score: 4.88, p-value: 1.08E-06

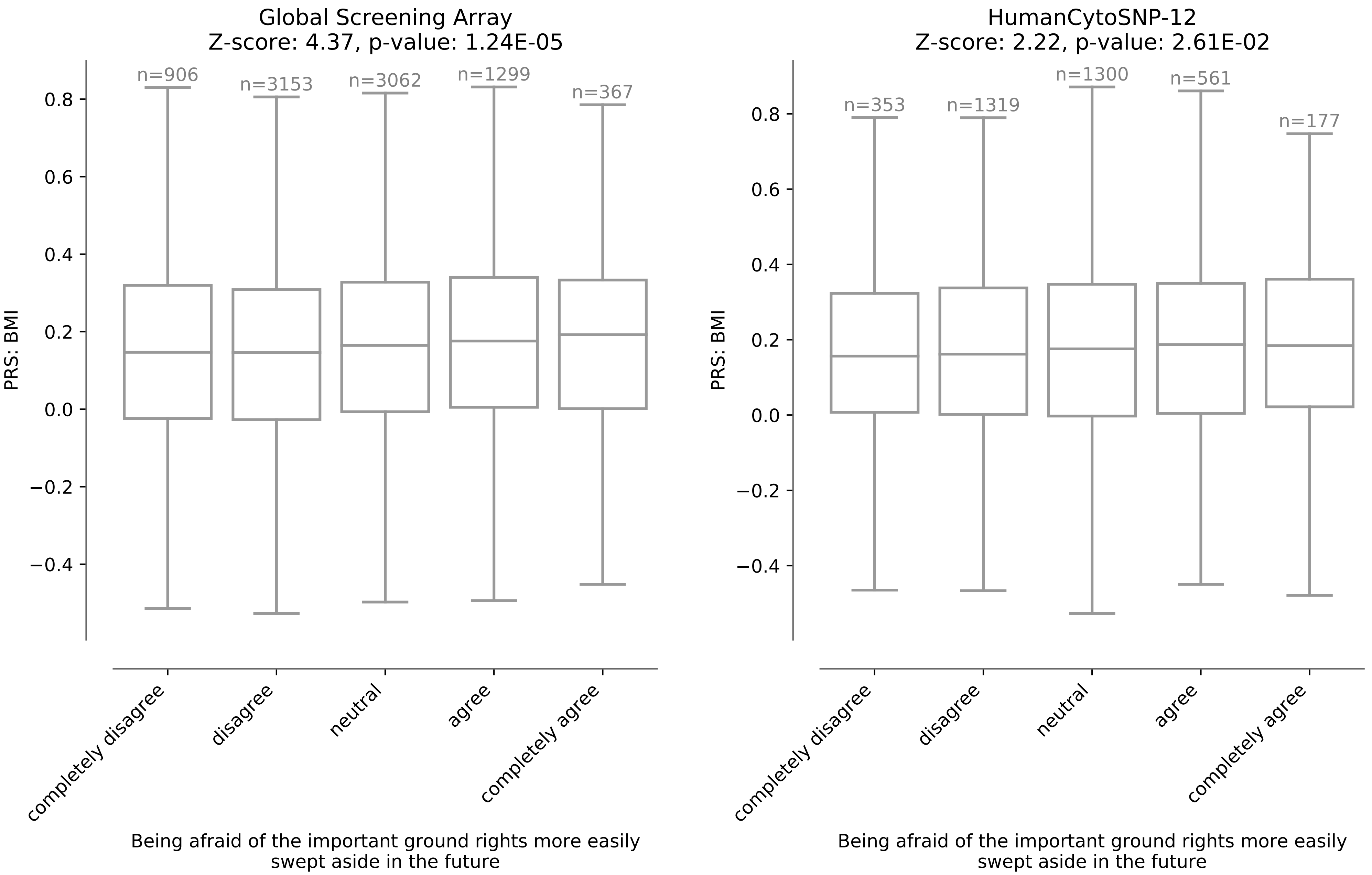

Being afraid of the important ground rights more easily swept aside in the future
PGS: Educational attainment
Meta analysis Z-score: -12.15, p-value: 5.81E-34

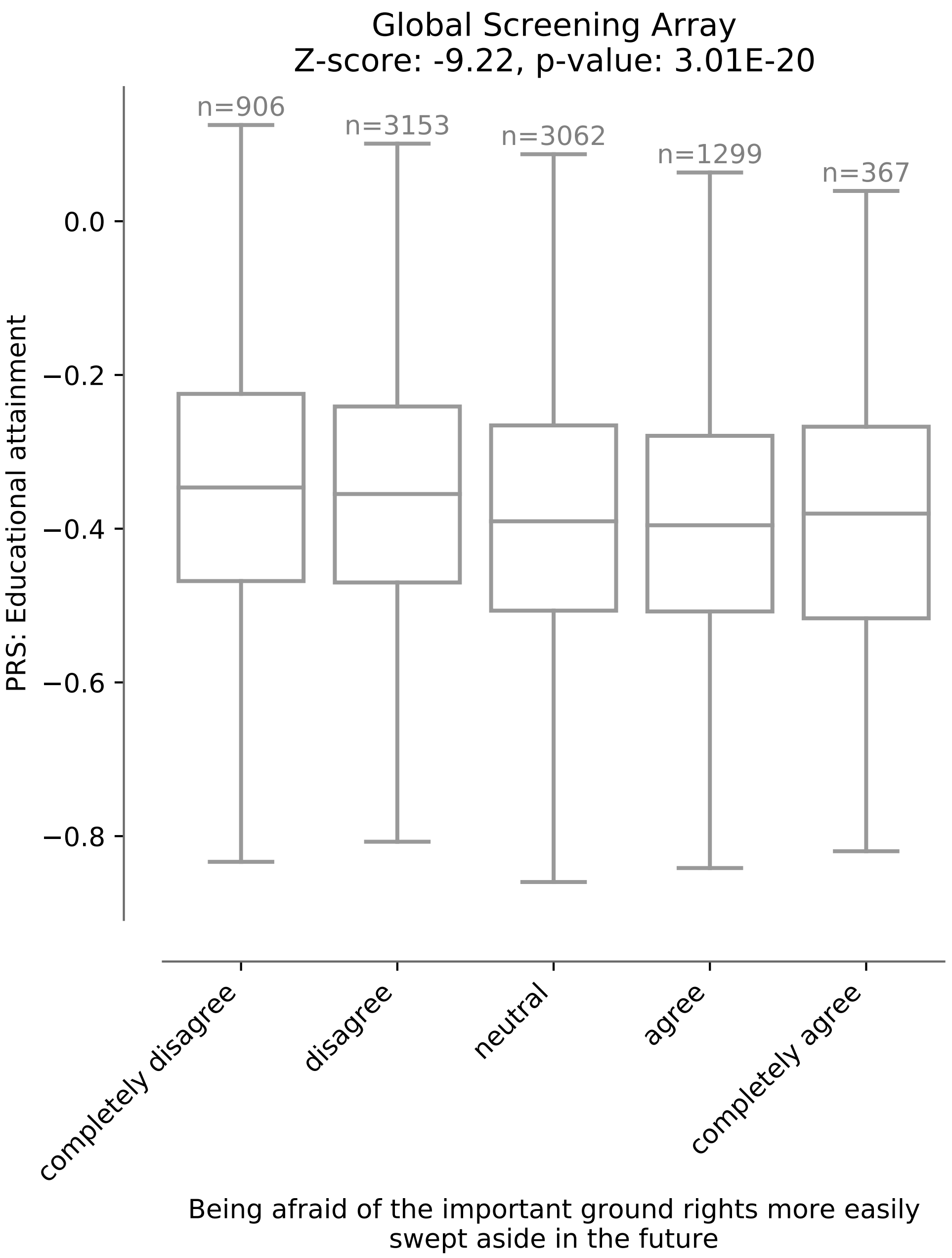

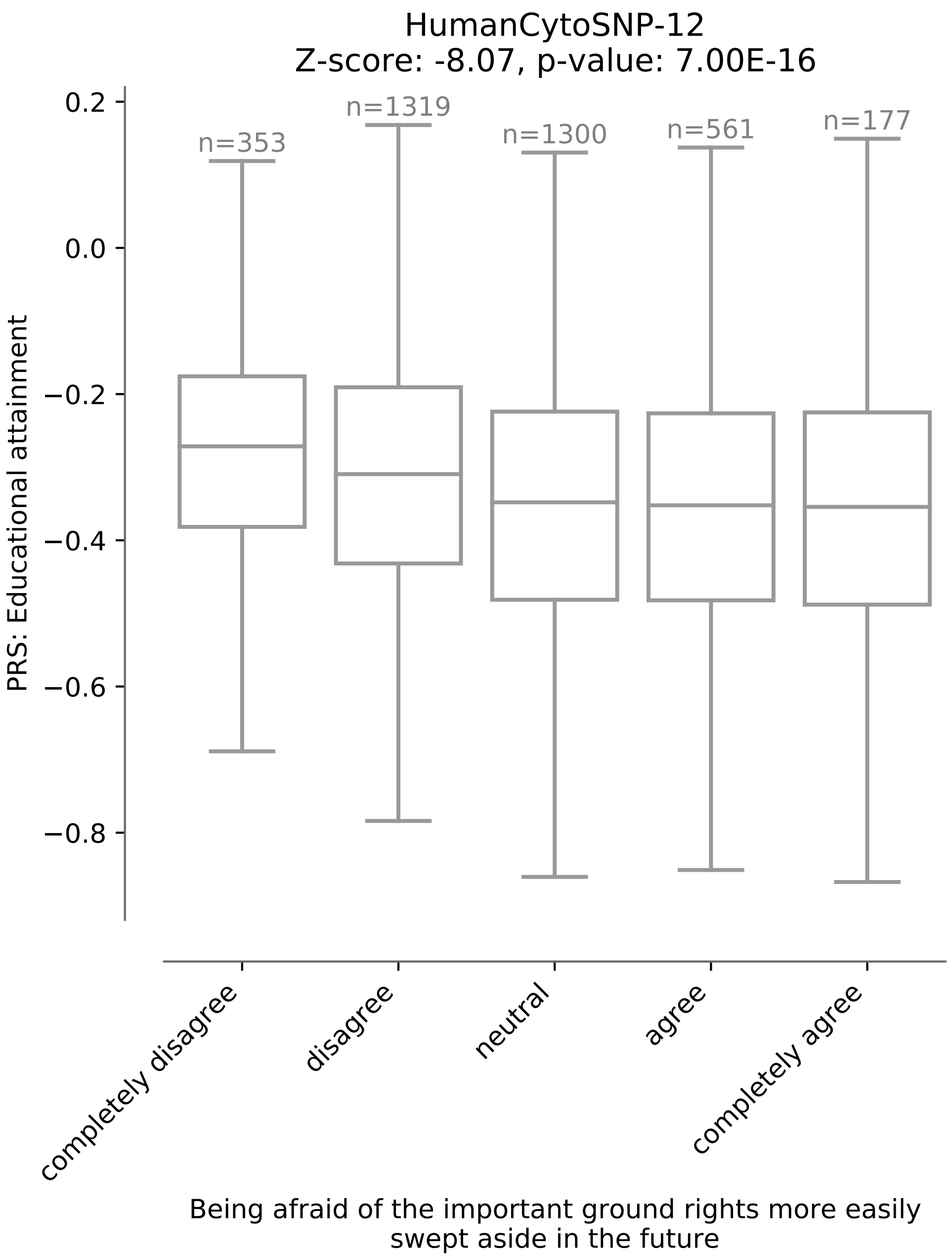

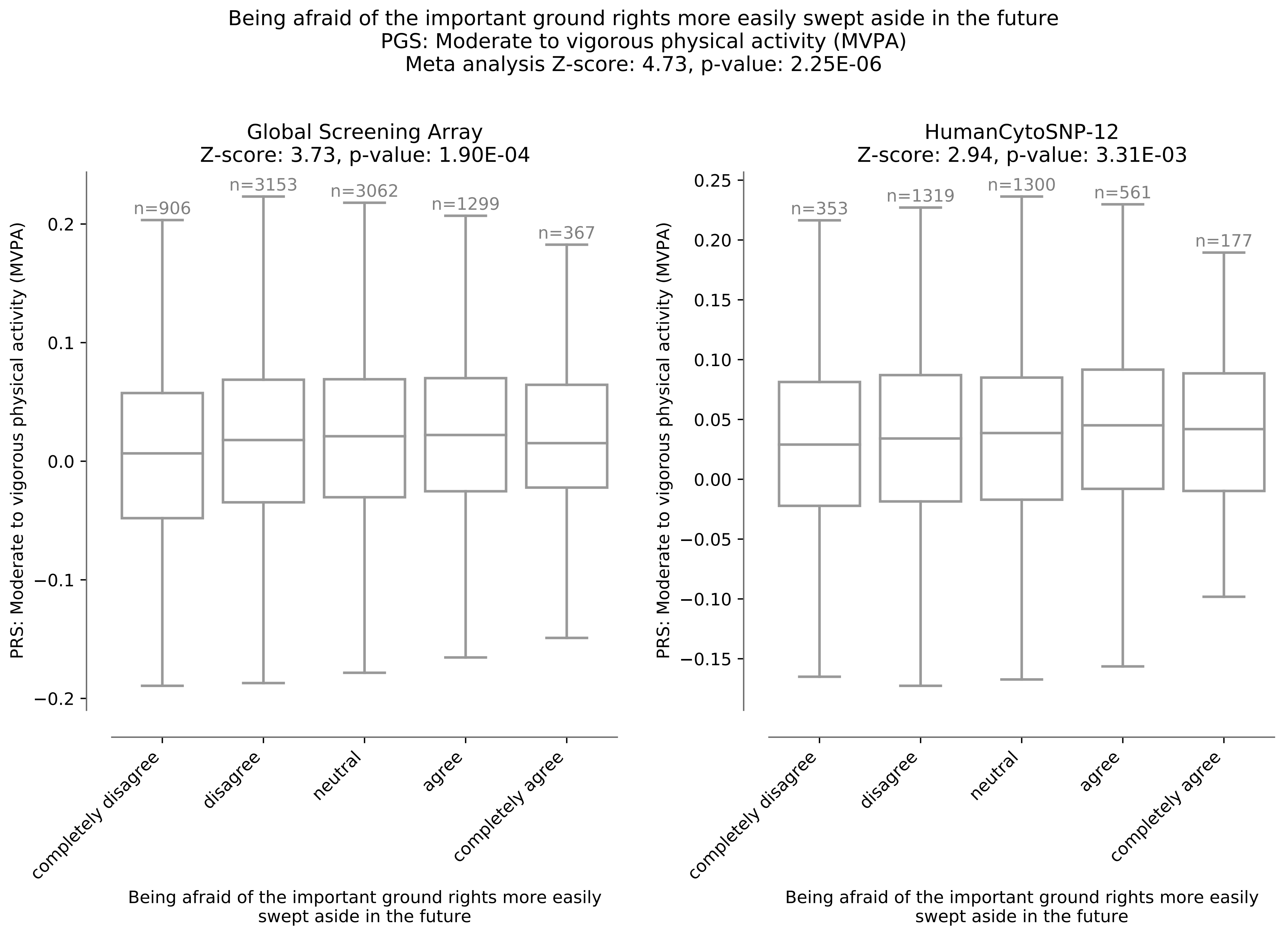
Being afraid of the important ground rights more easily swept aside in the future
PGS: Moderate to vigorous physical activity (MVPA)
Meta analysis Z-score: 4.73, p-value: 2.25E-06
Global Screening Array
Z-score: 3.73, p-value: 1.90E-04
HumanCytoSNP-12
Z-score: 2.94, p-value: 3.31E-03
PRS: Moderate to vigorous physical activity (MVPA)
n=906
n=3153
n=3062
n=1299
n=367
n=353
n=1319
n=1300
n=561
n=177
completely disagree
disagree
neutral
agree
completely agree
Being afraid of the important ground rights more easily swept aside in the future

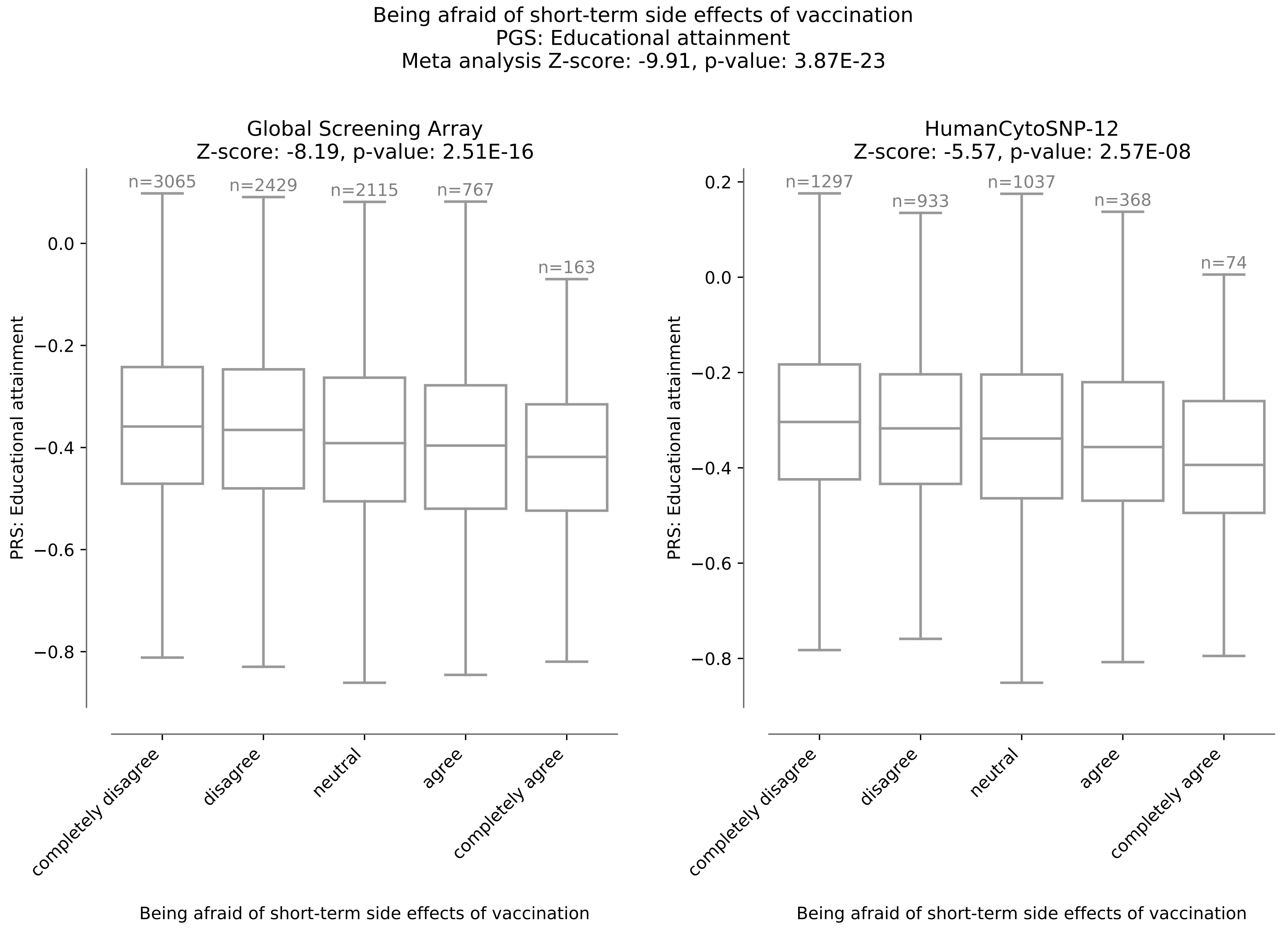
Being afraid of short-term side effects of vaccination
PGS: Educational attainment
Meta analysis Z-score: -9.91, p-value: 3.87E-23
Global Screening Array
Z-score: -8.19, p-value: 2.51E-16
HumanCytoSNP-12
Z-score: -5.57, p-value: 2.57E-08
n=3065
n=2429
n=2115
n=767
n=163
n=1297
n=933
n=1037
n=368
n=74
PRS: Educational attainment
0.2
0.0
−0.2
−0.4
−0.6
−0.8
completely disagree
disagree
neutral
agree
completely agree
Being afraid of short-term side effects of vaccination

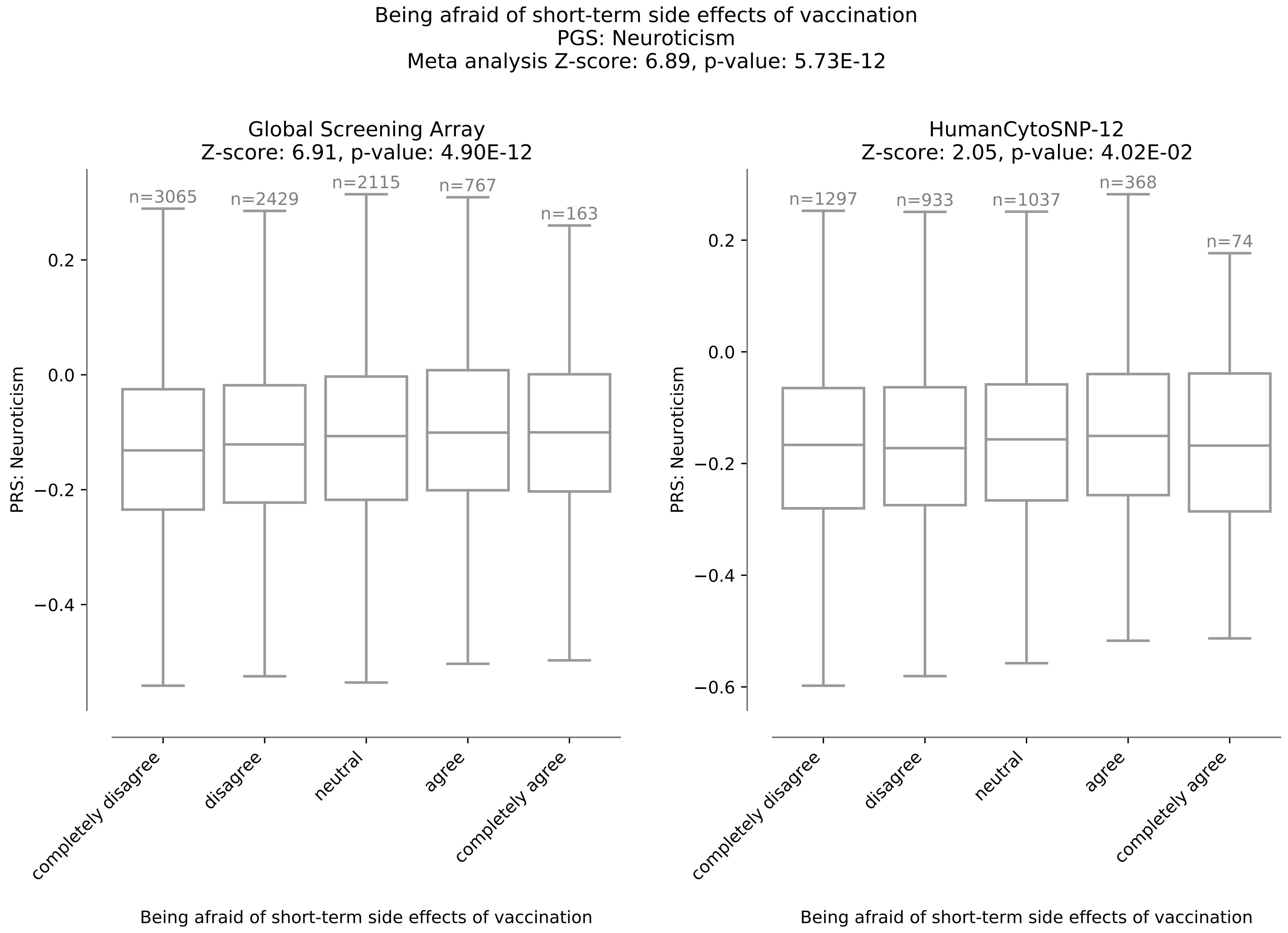
Being afraid of short-term side effects of vaccination
PGS: Neuroticism
Meta analysis Z-score: 6.89, p-value: 5.73E-12
Global Screening Array
Z-score: 6.91, p-value: 4.90E-12
n=3065
n=2429
n=2115
n=767
n=163
PRS: Neuroticism
0.2
0.0
−0.2
−0.4
completely disagree
disagree
neutral
agree
completely agree
Being afraid of short-term side effects of vaccination
HumanCytoSNP-12
Z-score: 2.05, p-value: 4.02E-02
n=1297
n=933
n=1037
n=368
n=74
PRS: Neuroticism
0.2
0.0
−0.2
−0.4
−0.6
completely disagree
disagree
neutral
agree
completely agree
Being afraid of short-term side effects of vaccination

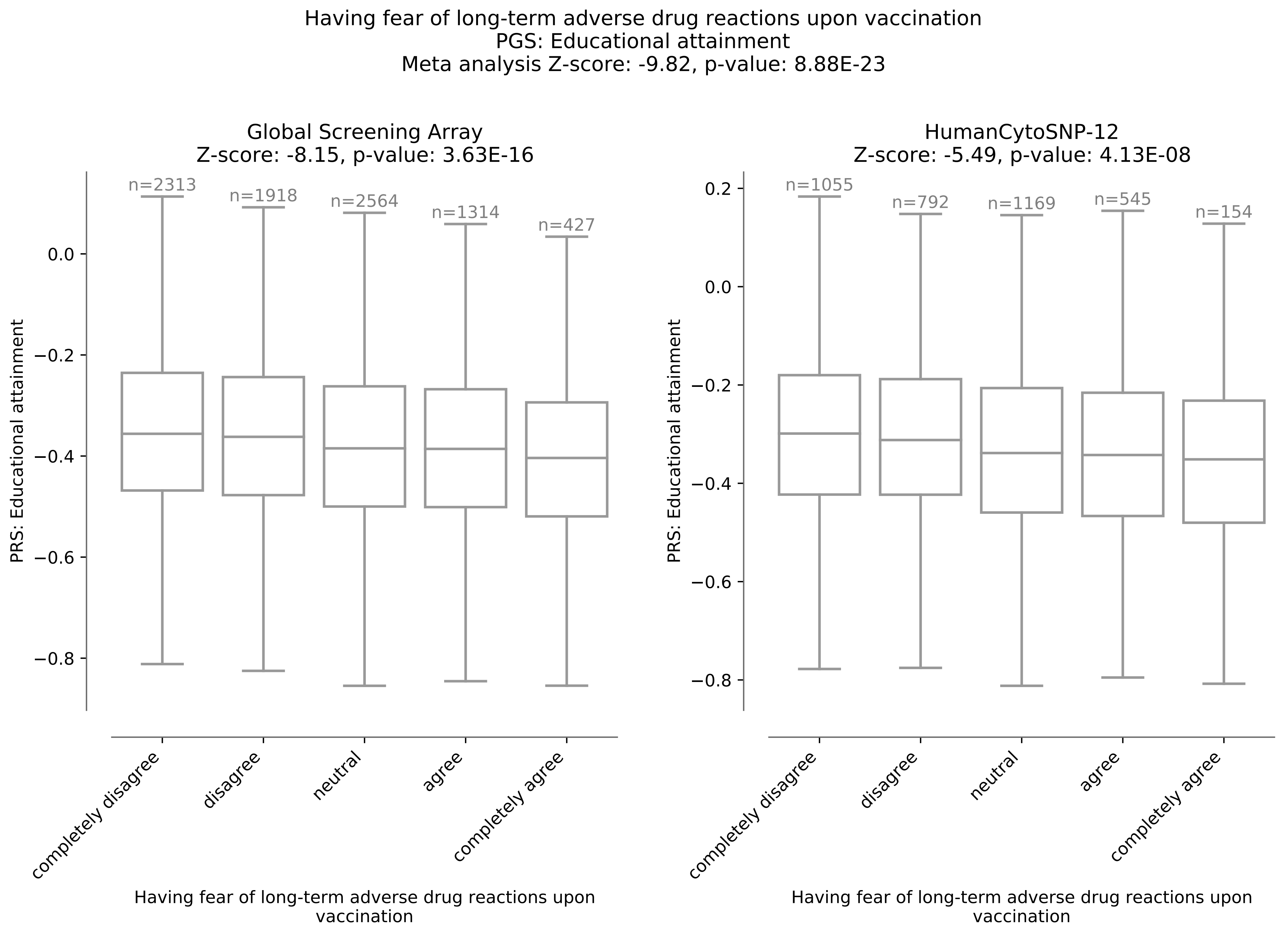
Having fear of long-term adverse drug reactions upon vaccination
PGS: Educational attainment
Meta analysis Z-score: -9.82, p-value: 8.88E-23
Global Screening Array
Z-score: -8.15, p-value: 3.63E-16
HumanCytoSNP-12
Z-score: -5.49, p-value: 4.13E-08
PRS: Educational attainment
n=2313
n=1918
n=2564
n=1314
n=427
n=1055
n=792
n=1169
n=545
n=154
0.2
0.0
−0.2
−0.4
−0.6
−0.8
completely disagree
disagree
neutral
agree
completely agree
Having fear of long-term adverse drug reactions upon vaccination

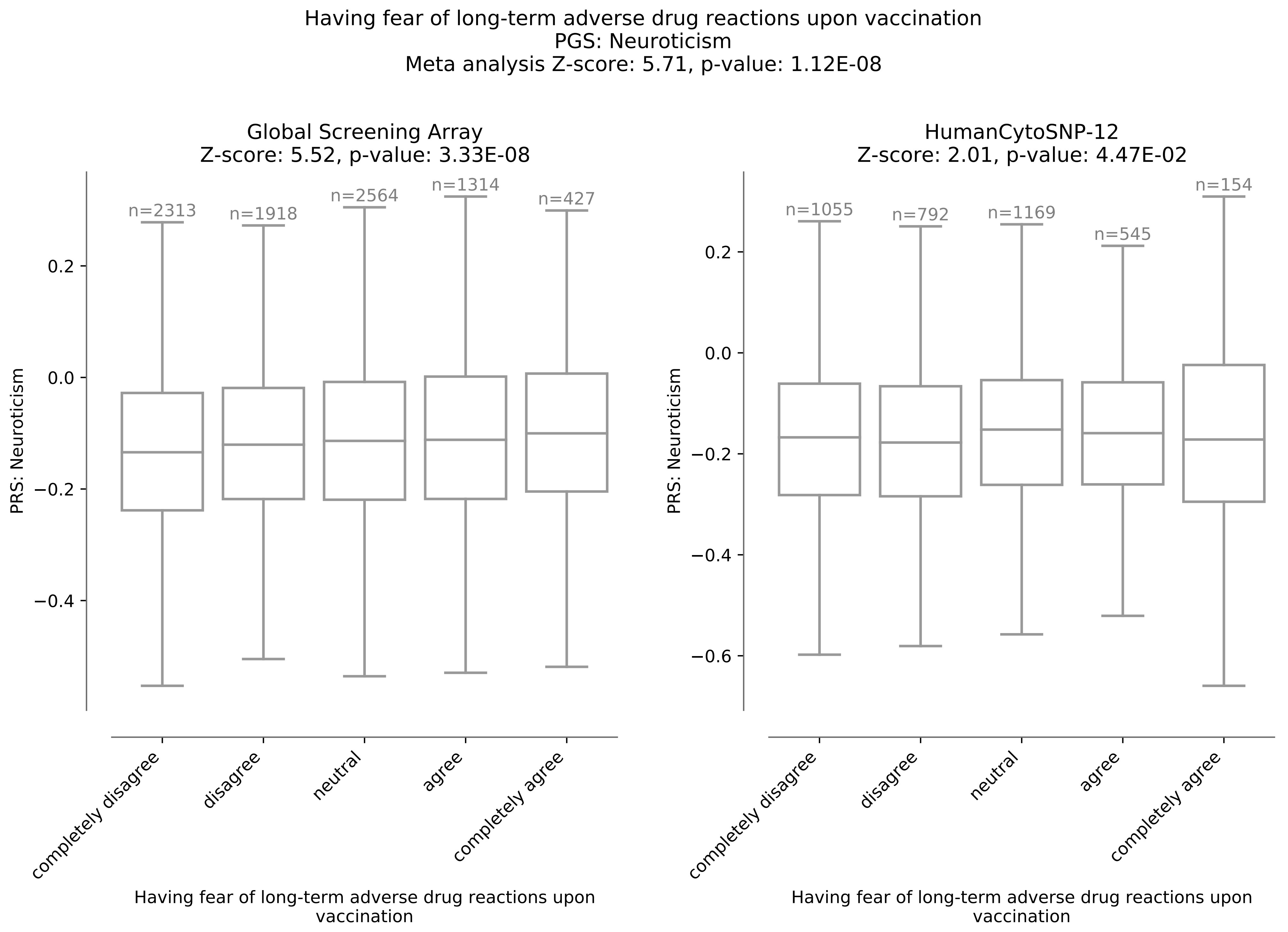

Having fear of long-term adverse drug reactions upon vaccination
PGS: Neuroticism
Meta analysis Z-score: 5.71, p-value: 1.12E-08
Global Screening Array
Z-score: 5.52, p-value: 3.33E-08
HumanCytoSNP-12
Z-score: 2.01, p-value: 4.47E-02
PRS: Neuroticism
n=2313
n=1918
n=2564
n=1314
n=427
n=1055
n=792
n=1169
n=545
n=154
0.2
0.0
−0.2
−0.4
−0.6
completely disagree
disagree
neutral
agree
completely agree
Having fear of long-term adverse drug reactions upon vaccination

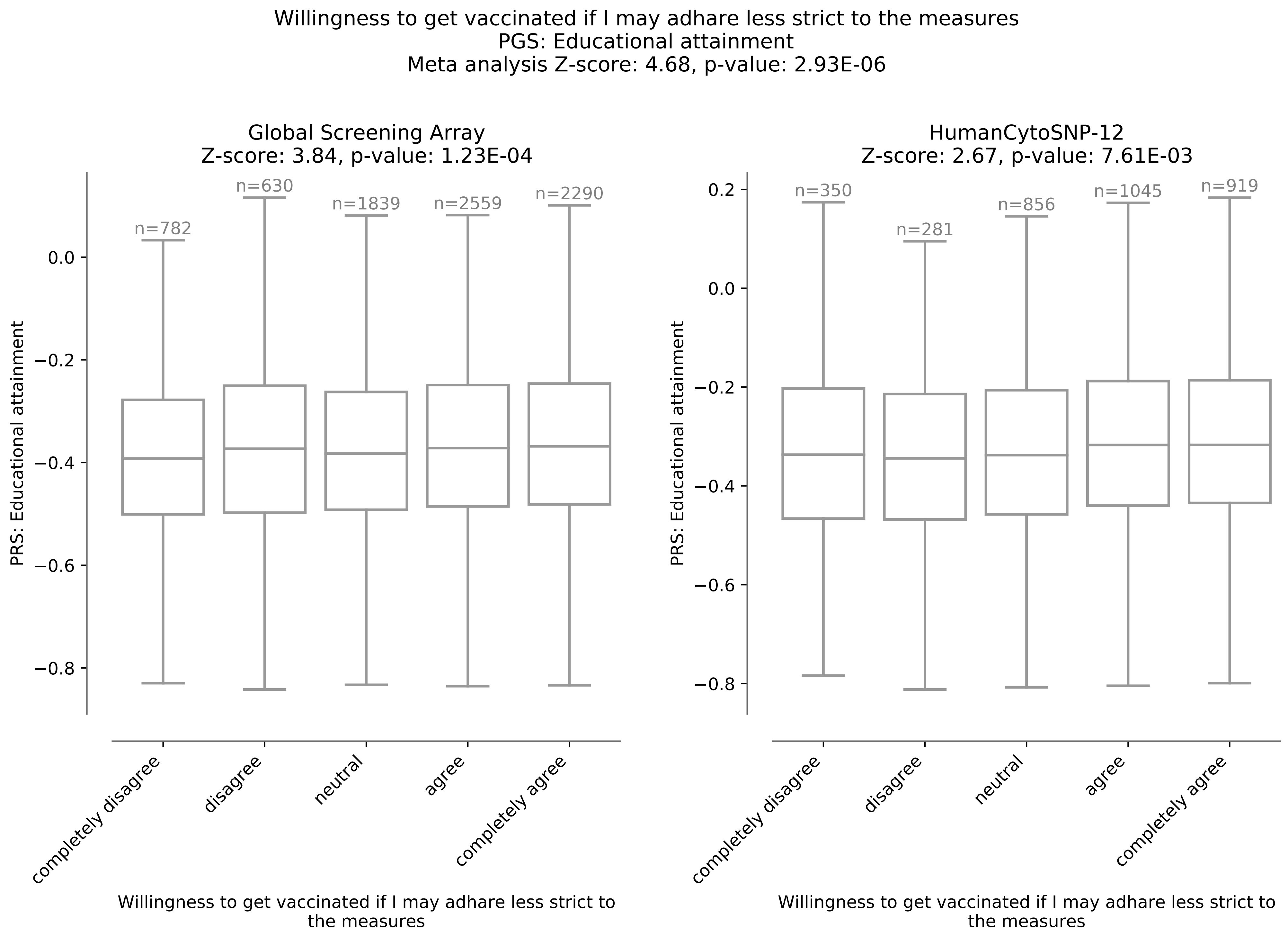
Willingness to get vaccinated if I may adhare less strict to the measures
PGS: Educational attainment
Meta analysis Z-score: 4.68, p-value: 2.93E-06
Global Screening Array
Z-score: 3.84, p-value: 1.23E-04
HumanCytoSNP-12
Z-score: 2.67, p-value: 7.61E-03
PRS: Educational attainment
n=782
n=630
n=1839
n=2559
n=2290
n=350
n=281
n=856
n=1045
n=919
0.2
0.0
−0.2
−0.4
−0.6
−0.8
completely disagree
disagree
neutral
agree
completely agree
Willingness to get vaccinated if I may adhare less strict to the measures

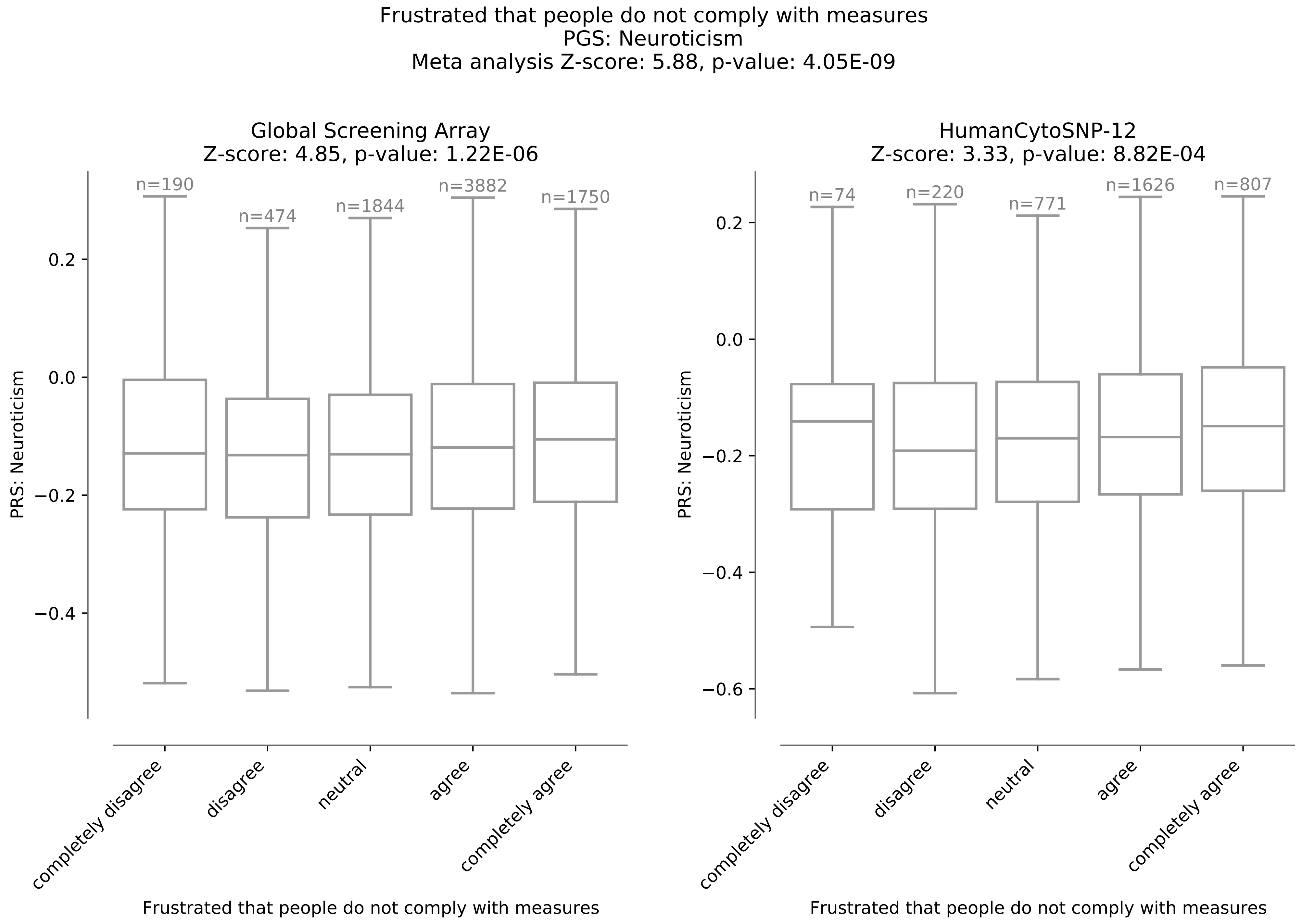
Frustrated that people do not comply with measures
PGS: Neuroticism
Meta analysis Z-score: 5.88, p-value: 4.05E-09
Global Screening Array
Z-score: 4.85, p-value: 1.22E-06
n=190
n=474
n=1844
n=3882
n=1750
PRS: Neuroticism
0.2
0.0
−0.2
−0.4
completely disagree
disagree
neutral
agree
completely agree
Frustrated that people do not comply with measures
HumanCytoSNP-12
Z-score: 3.33, p-value: 8.82E-04
n=74
n=220
n=771
n=1626
n=807
PRS: Neuroticism
0.2
0.0
−0.2
−0.4
−0.6
completely disagree
disagree
neutral
agree
completely agree
Frustrated that people do not comply with measures

Frustrated with how things go in society
PGS: Educational attainment
Meta analysis Z-score: -5.19, p-value: 2.07E-07

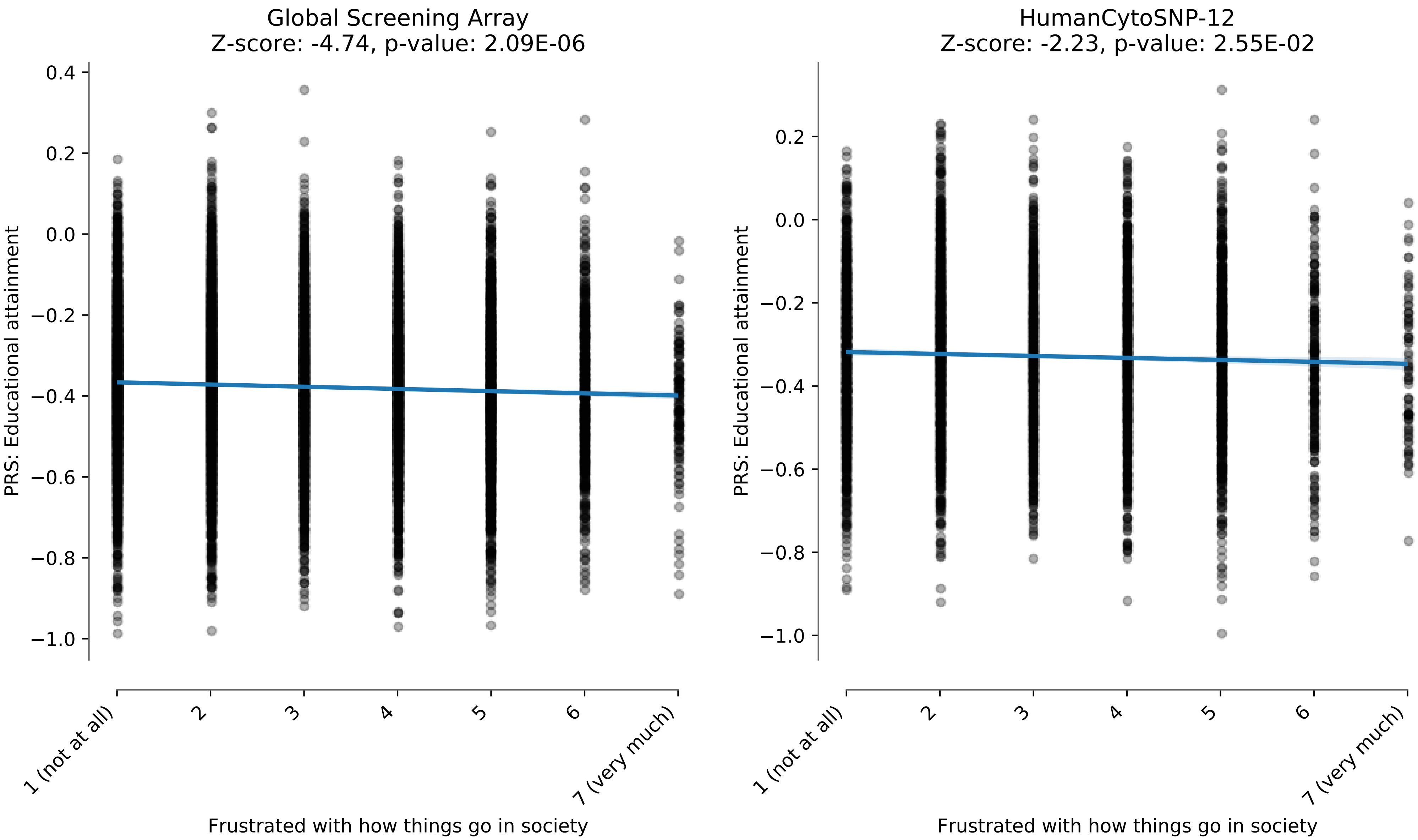

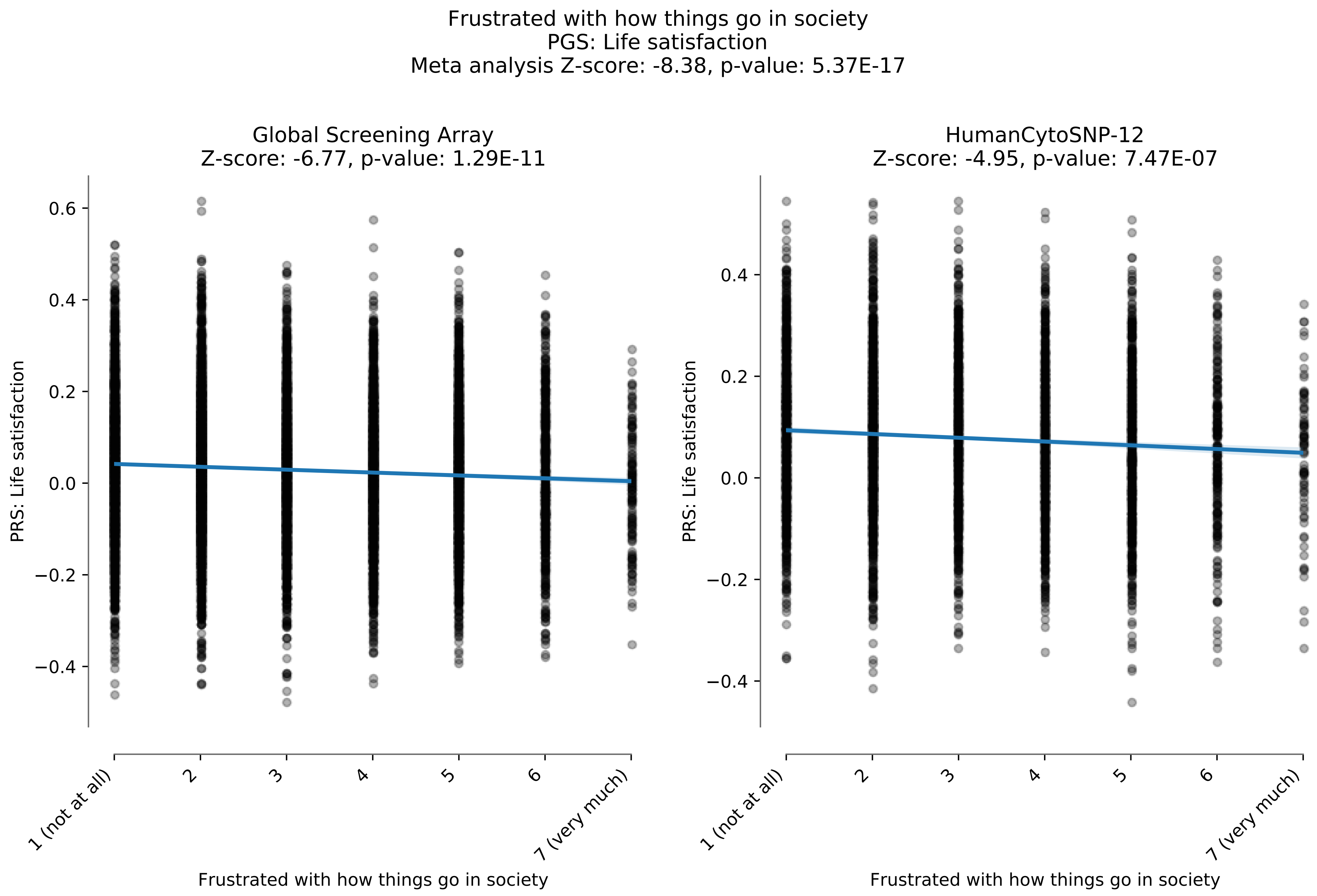
Frustrated with how things go in society
PGS: Life satisfaction
Meta analysis Z-score: -8.38, p-value: 5.37E-17
Global Screening Array
Z-score: -6.77, p-value: 1.29E-11
HumanCytoSNP-12
Z-score: -4.95, p-value: 7.47E-07
PRS: Life satisfaction
0.6
0.4
0.2
0.0
−0.2
−0.4
1 (not at all)
2
3
4
5
6
7 (very much)
Frustrated with how things go in society

Frustrated with how things go in society
PGS: Neuroticism
Meta analysis Z-score: 8.51, p-value: 1.74E-17

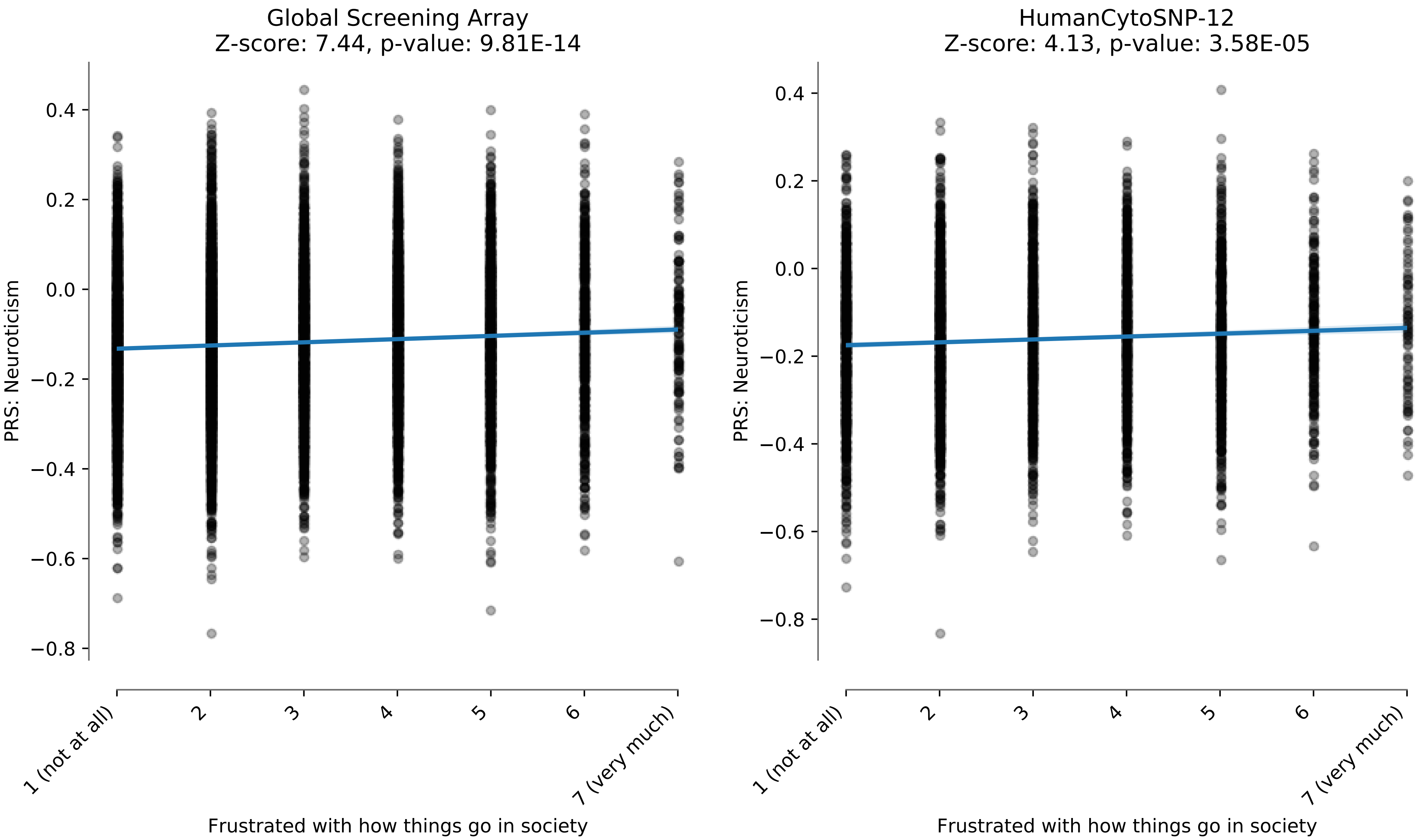

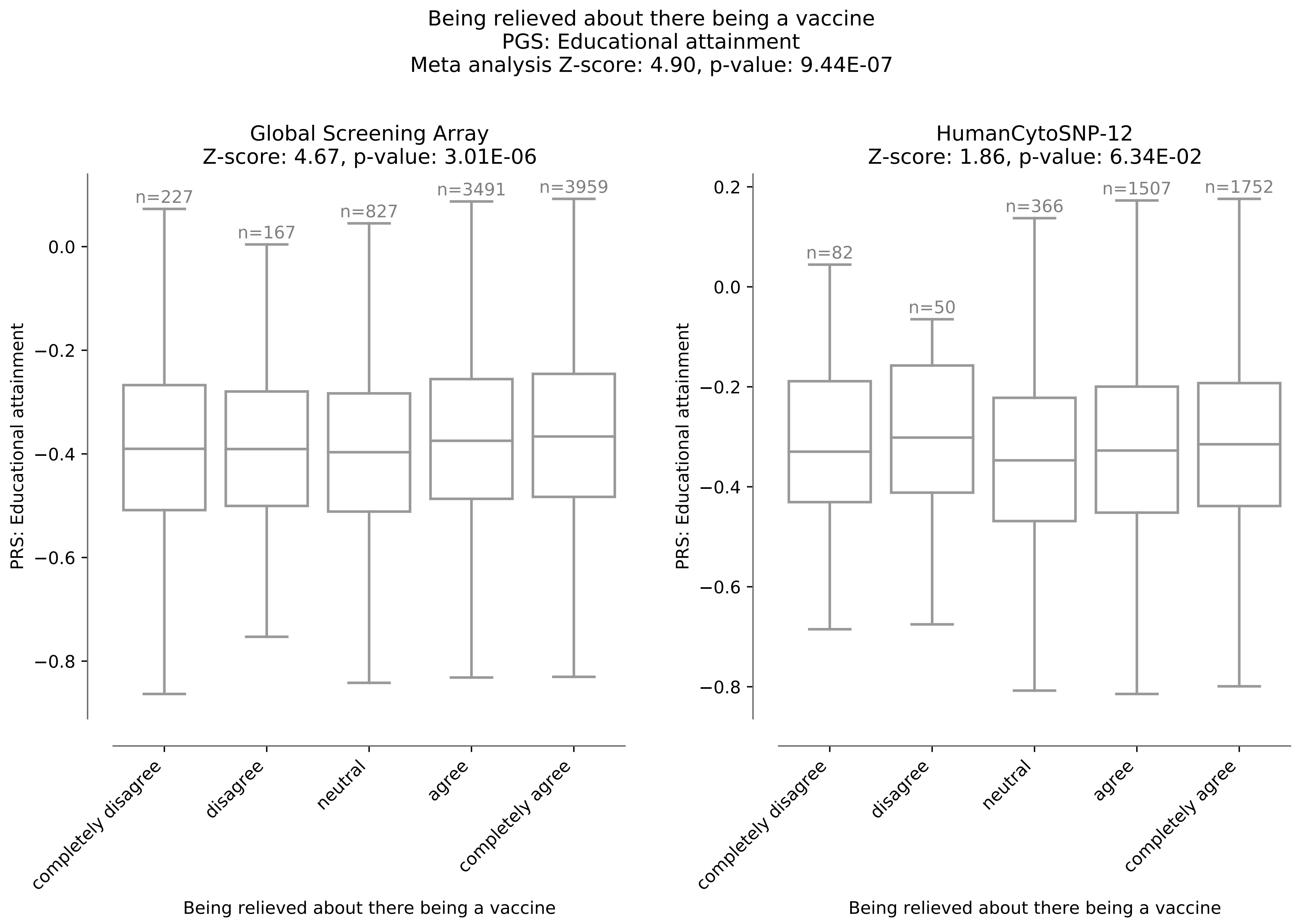
Being relieved about there being a vaccine
PGS: Educational attainment
Meta analysis Z-score: 4.90, p-value: 9.44E-07
Global Screening Array
Z-score: 4.67, p-value: 3.01E-06
HumanCytoSNP-12
Z-score: 1.86, p-value: 6.34E-02
PRS: Educational attainment
n=227
n=167
n=827
n=3491
n=3959
n=82
n=50
n=366
n=1507
n=1752
0.2
0.0
−0.2
−0.4
−0.6
−0.8
completely disagree
disagree
neutral
agree
completely agree
Being relieved about there being a vaccine

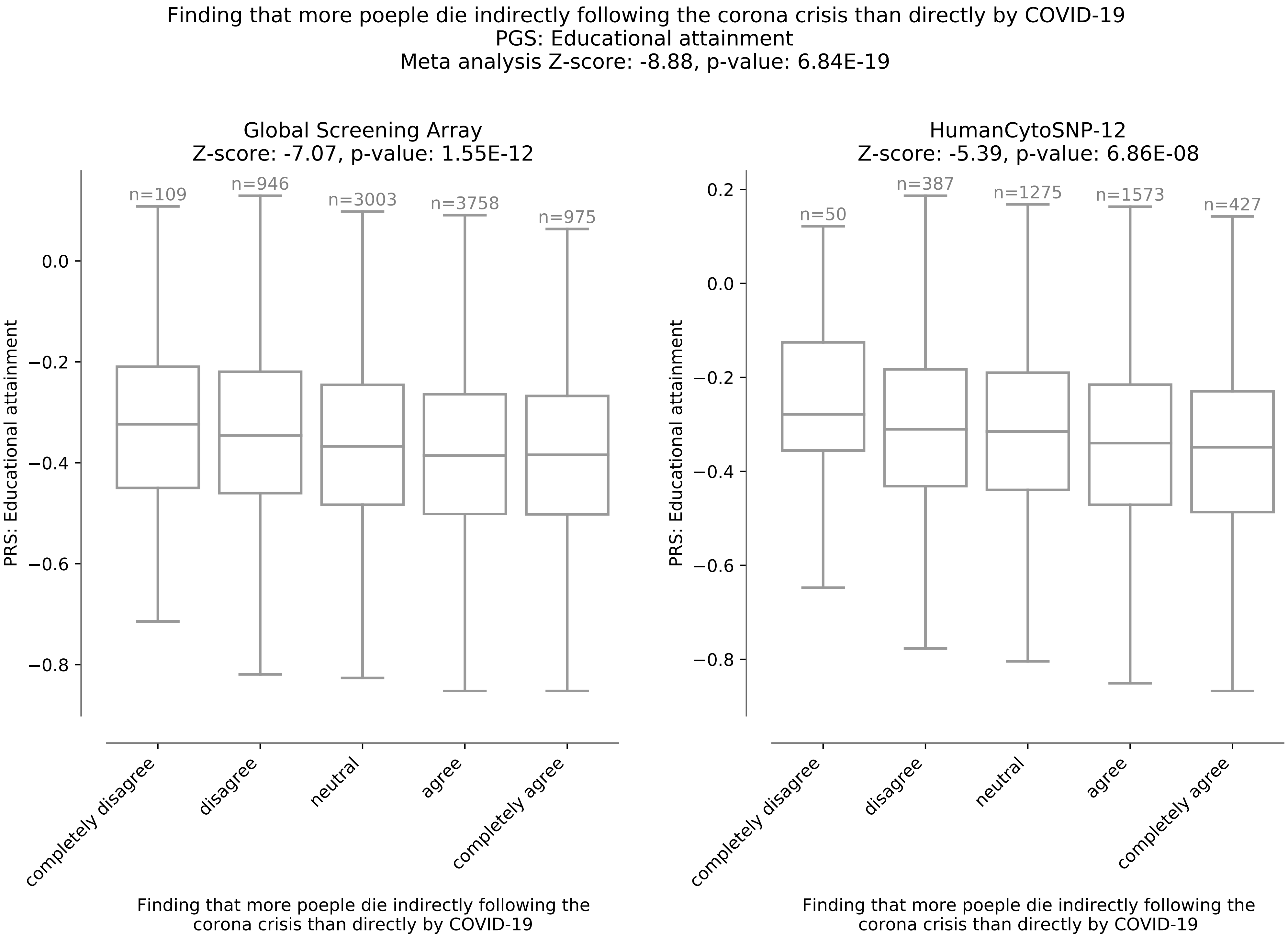

Finding that more poeple die indirectly following the corona crisis than directly by COVID-19
PGS: Educational attainment
Meta analysis Z-score: -8.88, p-value: 6.84E-19
Global Screening Array
Z-score: -7.07, p-value: 1.55E-12
n=109
n=946
n=3003
n=3758
n=975
PRS: Educational attainment
0.0
−0.2
−0.4
−0.6
−0.8
completely disagree
disagree
neutral
agree
completely agree
Finding that more poeple die indirectly following the corona crisis than directly by COVID-19
HumanCytoSNP-12
Z-score: -5.39, p-value: 6.86E-08
n=50
n=387
n=1275
n=1573
n=427
PRS: Educational attainment
0.2
0.0
−0.2
−0.4
−0.6
−0.8
completely disagree
disagree
neutral
agree
completely agree
Finding that more poeple die indirectly following the corona crisis than directly by COVID-19

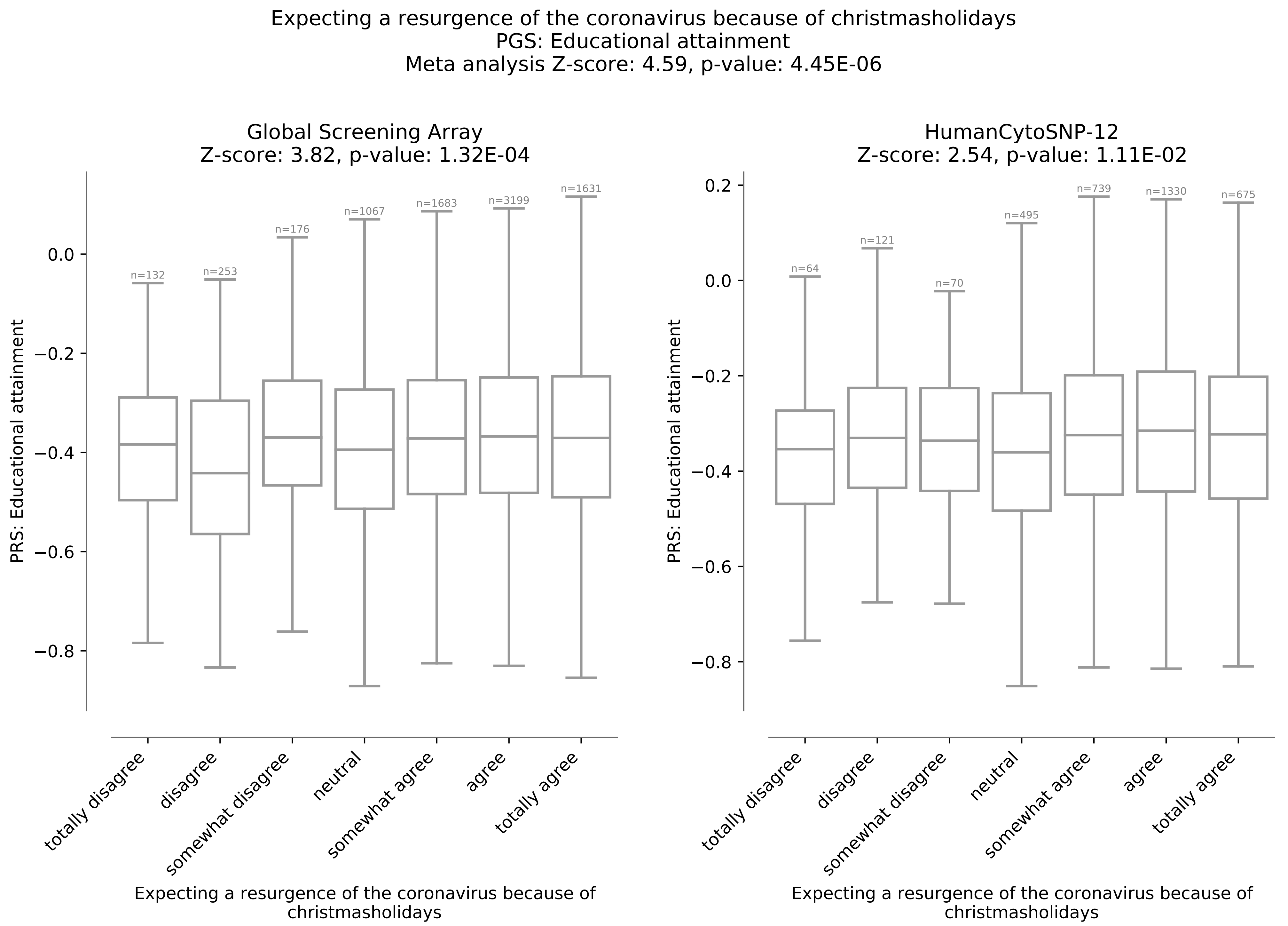

Expecting a resurgence of the coronavirus because of christmasholidays
PGS: Educational attainment
Meta analysis Z-score: 4.59, p-value: 4.45E-06
Global Screening Array
Z-score: 3.82, p-value: 1.32E-04
HumanCytoSNP-12
Z-score: 2.54, p-value: 1.11E-02
PRS: Educational attainment
n=132
n=253
n=176
n=1067
n=1683
n=3199
n=1631
n=64
n=121
n=70
n=495
n=739
n=1330
n=675
0.2
0.0
−0.2
−0.4
−0.6
−0.8
totally disagree
disagree
somewhat disagree
neutral
somewhat agree
agree
totally agree
Expecting a resurgence of the coronavirus because of christmasholidays

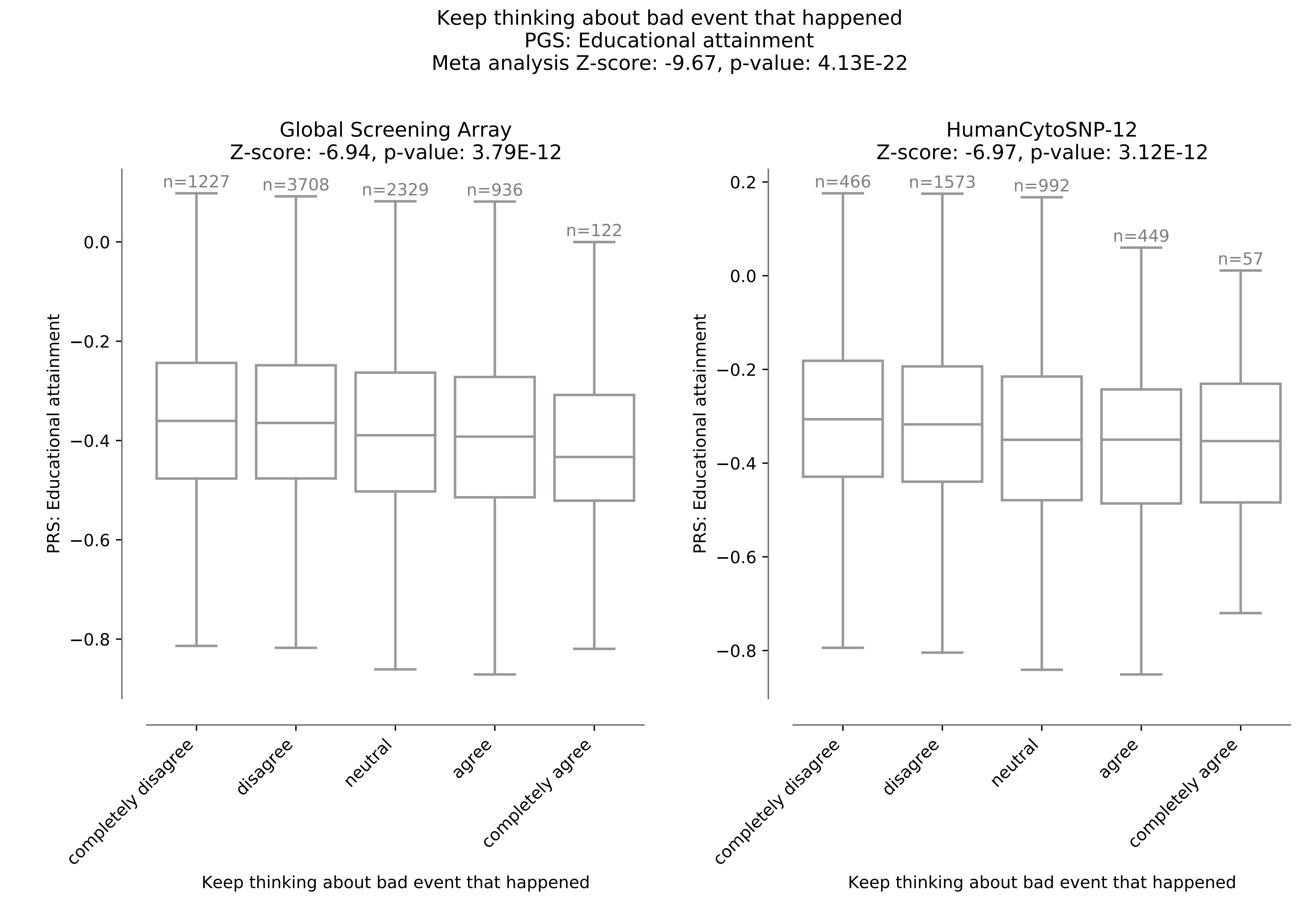

Keep thinking about bad event that happened
PGS: Educational attainment
Meta analysis Z-score: -9.67, p-value: 4.13E-22
Global Screening Array
Z-score: -6.94, p-value: 3.79E-12
n=1227
n=3708
n=2329
n=936
n=122
PRS: Educational attainment
0.0
−0.2
−0.4
−0.6
−0.8
completely disagree
disagree
neutral
agree
completely agree
Keep thinking about bad event that happened
HumanCytoSNP-12
Z-score: -6.97, p-value: 3.12E-12
n=466
n=1573
n=992
n=449
n=57
PRS: Educational attainment
0.2
0.0
−0.2
−0.4
−0.6
−0.8
completely disagree
disagree
neutral
agree
completely agree
Keep thinking about bad event that happened

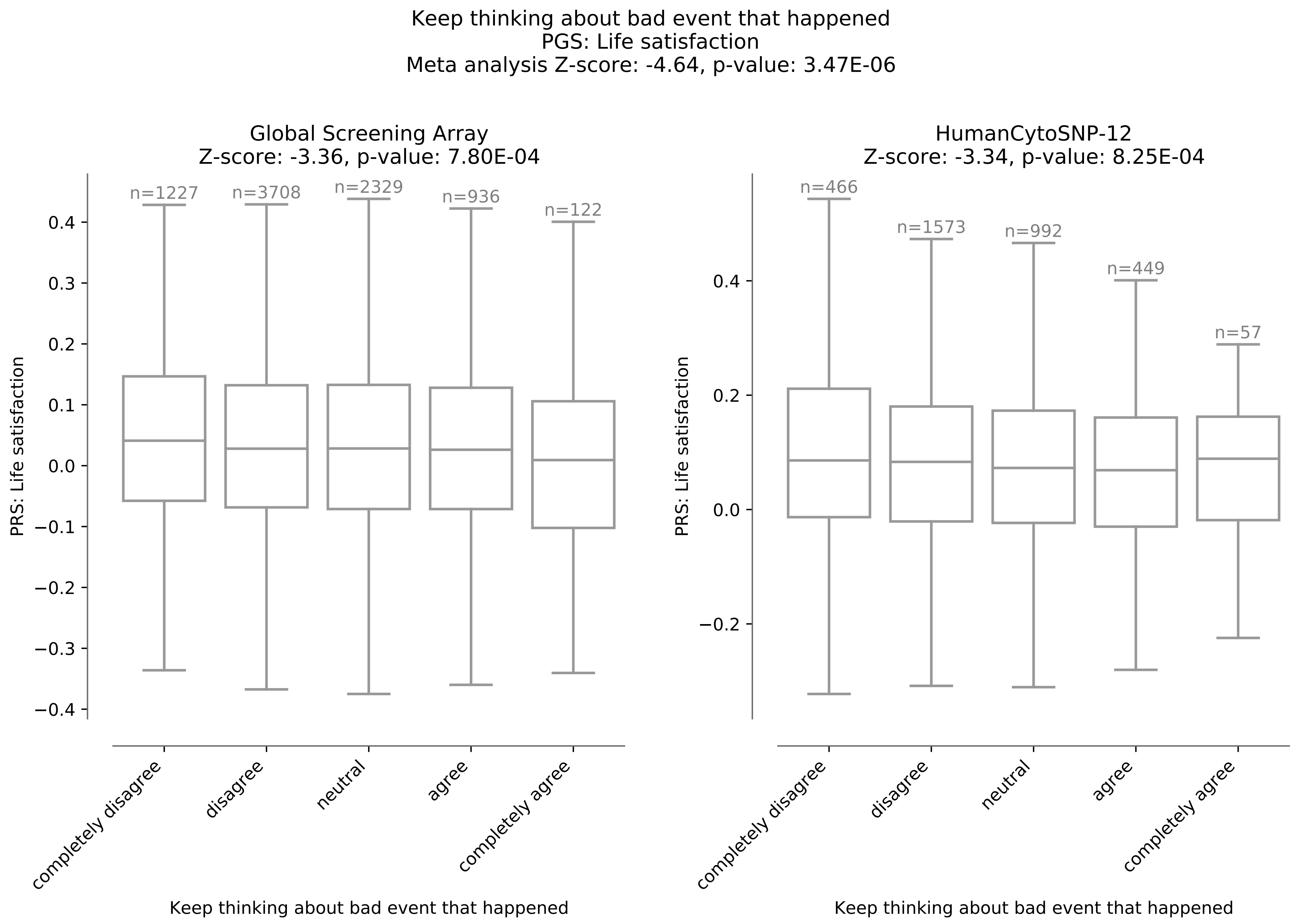
Keep thinking about bad event that happened
PGS: Life satisfaction
Meta analysis Z-score: -4.64, p-value: 3.47E-06
Global Screening Array
Z-score: -3.36, p-value: 7.80E-04
n=1227
n=3708
n=2329
n=936
n=122
PRS: Life satisfaction
0.4
0.3
0.2
0.1
0.0
−0.1
−0.2
−0.3
−0.4
completely disagree
disagree
neutral
agree
completely agree
Keep thinking about bad event that happened
HumanCytoSNP-12
Z-score: -3.34, p-value: 8.25E-04
n=466
n=1573
n=992
n=449
n=57
PRS: Life satisfaction
0.4
0.2
0.0
−0.2
completely disagree
disagree
neutral
agree
completely agree
Keep thinking about bad event that happened

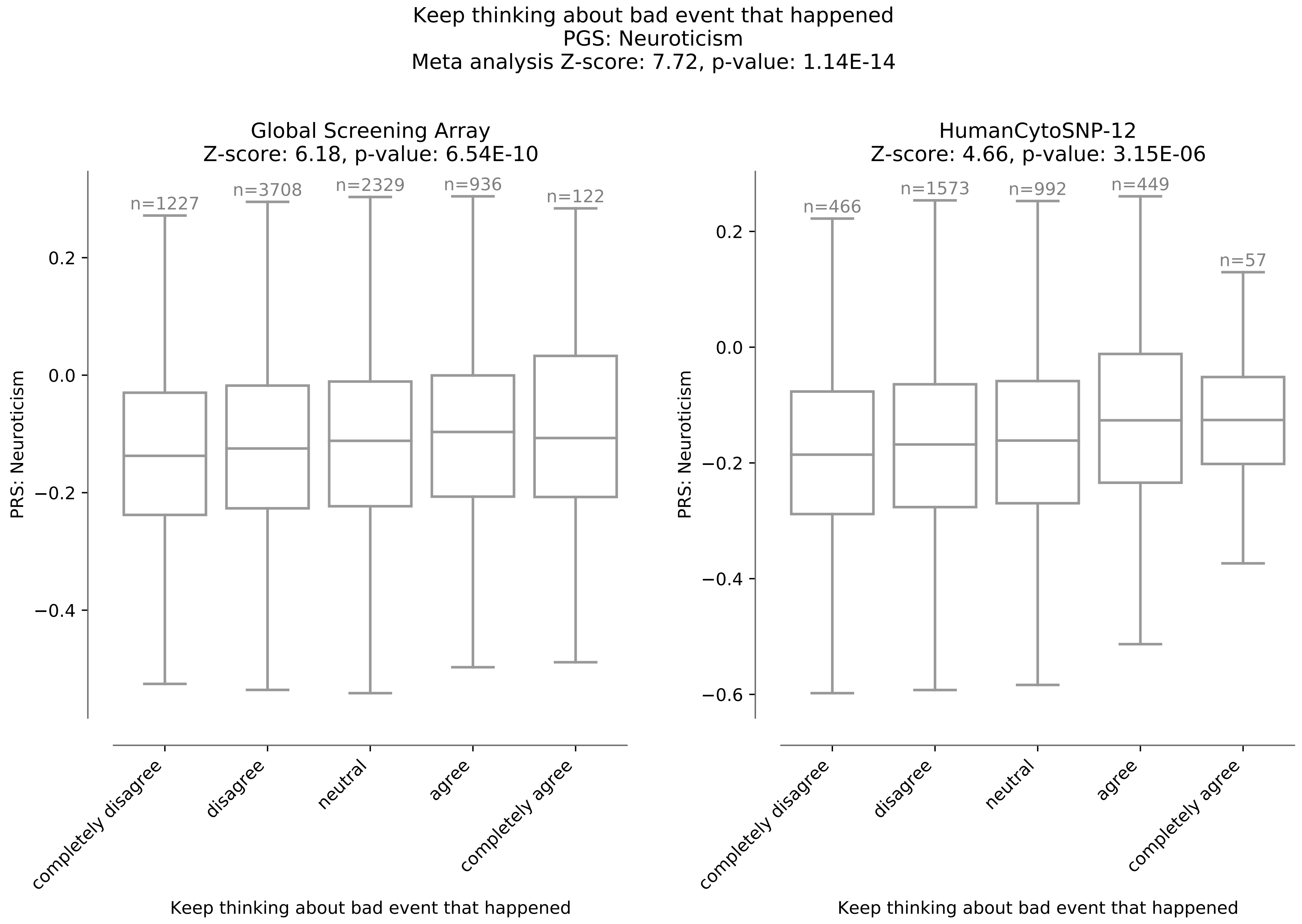
Keep thinking about bad event that happened
PGS: Neuroticism
Meta analysis Z-score: 7.72, p-value: 1.14E-14
Global Screening Array
Z-score: 6.18, p-value: 6.54E-10
n=1227
n=3708
n=2329
n=936
n=122
PRS: Neuroticism
0.2
0.0
−0.2
−0.4
completely disagree
disagree
neutral
agree
completely agree
Keep thinking about bad event that happened
HumanCytoSNP-12
Z-score: 4.66, p-value: 3.15E-06
n=466
n=1573
n=992
n=449
n=57
PRS: Neuroticism
0.2
0.0
−0.2
−0.4
−0.6
completely disagree
disagree
neutral
agree
completely agree
Keep thinking about bad event that happened

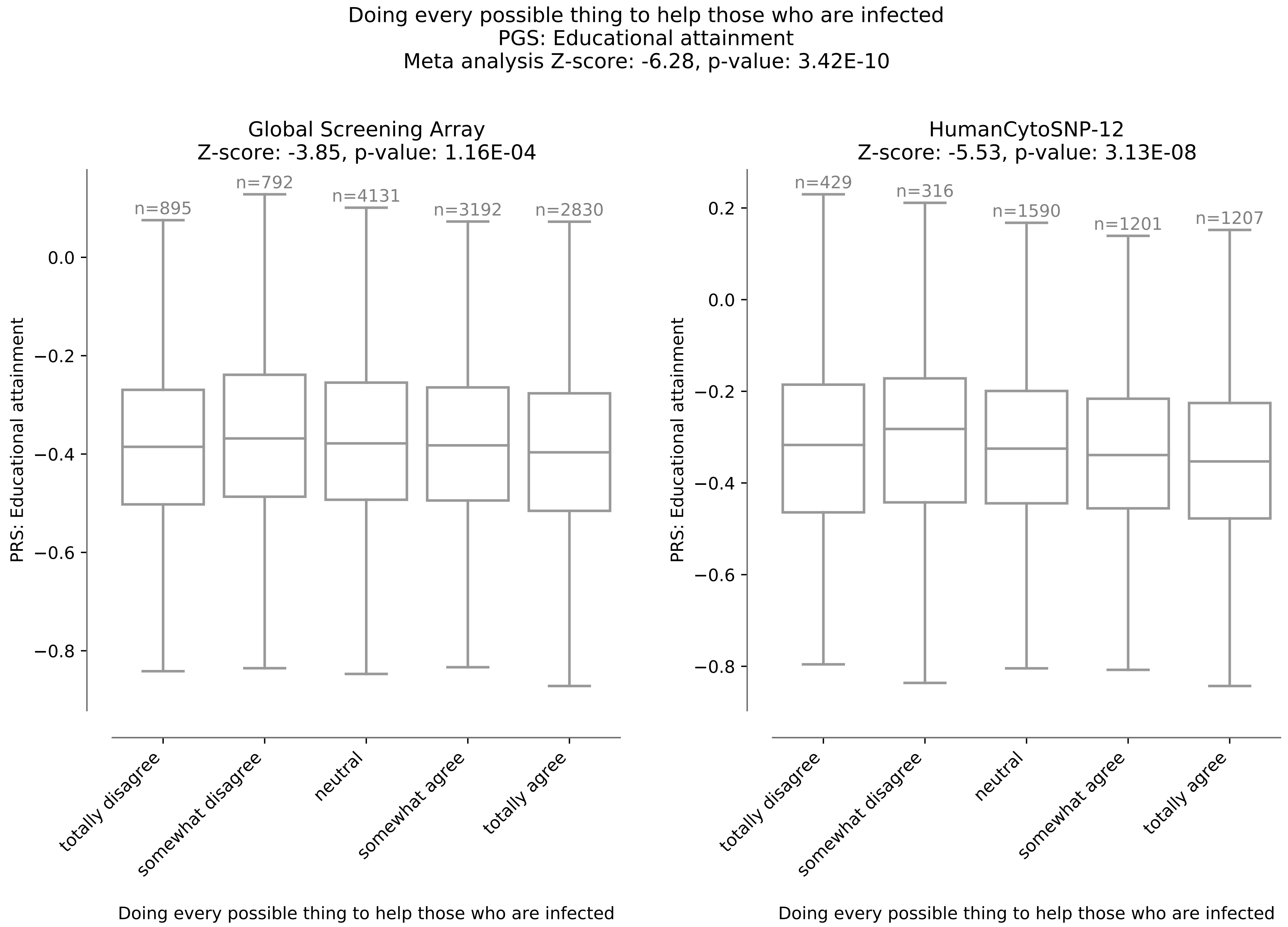
Doing every possible thing to help those who are infected
PGS: Educational attainment
Meta analysis Z-score: -6.28, p-value: 3.42E-10
Global Screening Array
Z-score: -3.85, p-value: 1.16E-04
HumanCytoSNP-12
Z-score: -5.53, p-value: 3.13E-08
PRS: Educational attainment
n=895
n=792
n=4131
n=3192
n=2830
n=429
n=316
n=1590
n=1201
n=1207
0.2
0.0
−0.2
−0.4
−0.6
−0.8
totally disagree
somewhat disagree
neutral
somewhat agree
totally agree
Doing every possible thing to help those who are infected

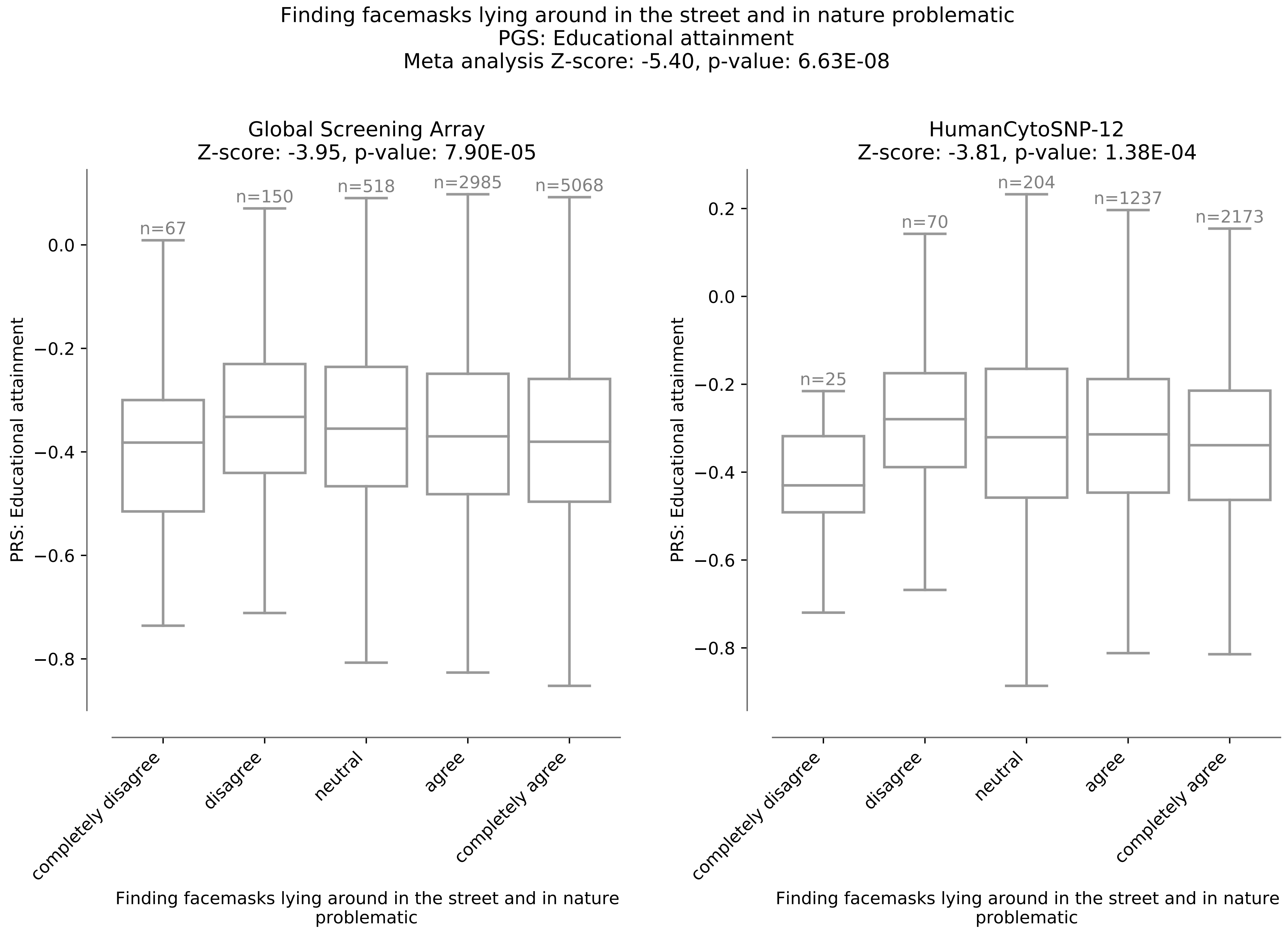

Finding facemasks lying around in the street and in nature problematic
PGS: Educational attainment
Meta analysis Z-score: -5.40, p-value: 6.63E-08
Global Screening Array
Z-score: -3.95, p-value: 7.90E-05
n=67
n=150
n=518
n=2985
n=5068
0.0
−0.2
−0.4
−0.6
−0.8
PRS: Educational attainment
completely disagree
disagree
neutral
agree
completely agree
Finding facemasks lying around in the street and in nature problematic
HumanCytoSNP-12
Z-score: -3.81, p-value: 1.38E-04
n=25
n=70
n=204
n=1237
n=2173
0.2
0.0
−0.2
−0.4
−0.6
−0.8
PRS: Educational attainment
completely disagree
disagree
neutral
agree
completely agree
Finding facemasks lying around in the street and in nature problematic

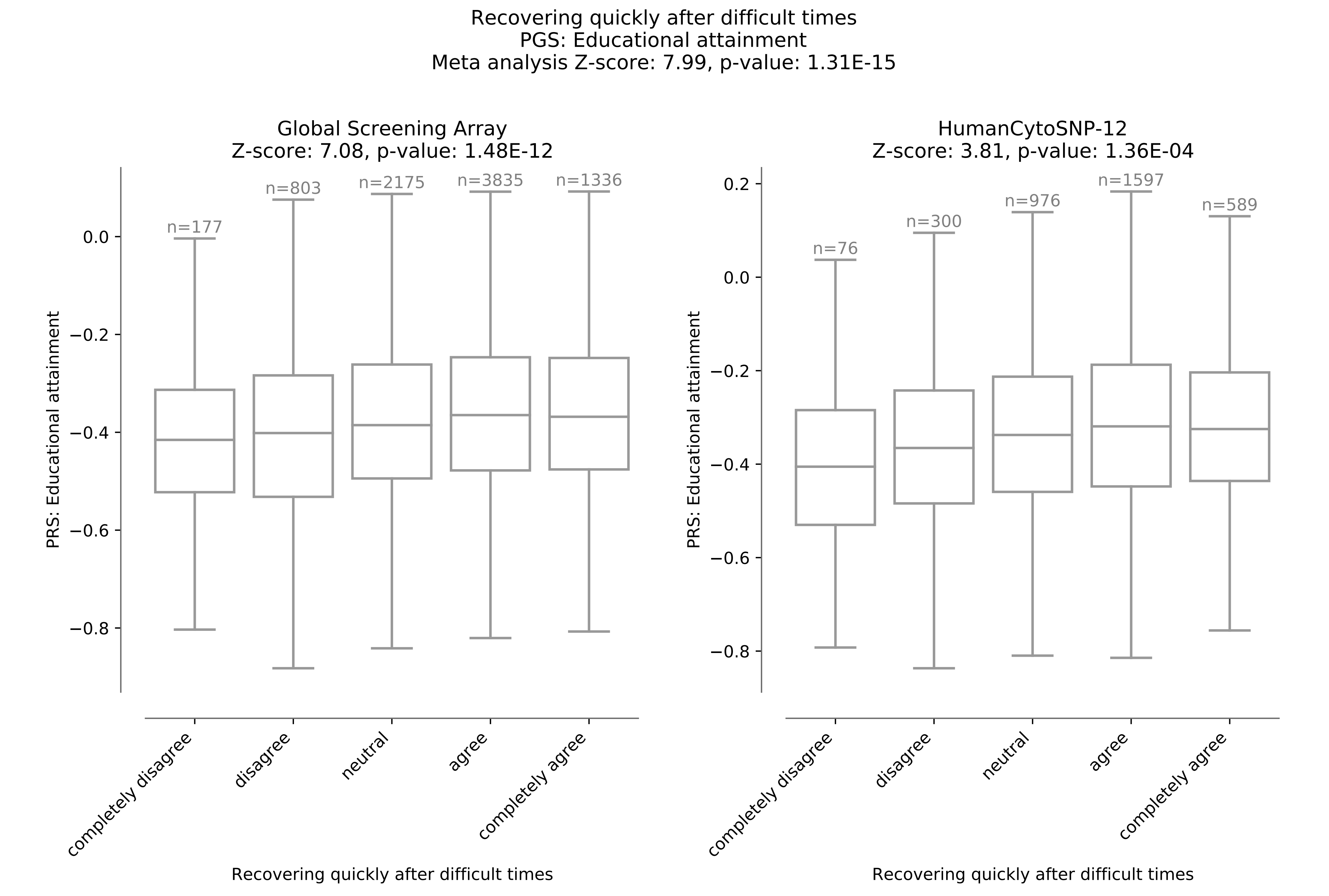
Recovering quickly after difficult times
PGS: Educational attainment
Meta analysis Z-score: 7.99, p-value: 1.31E-15
Global Screening Array
Z-score: 7.08, p-value: 1.48E-12
n=177
n=803
n=2175
n=3835
n=1336
PRS: Educational attainment
0.0
−0.2
−0.4
−0.6
−0.8
completely disagree
disagree
neutral
agree
completely agree
Recovering quickly after difficult times
HumanCytoSNP-12
Z-score: 3.81, p-value: 1.36E-04
n=76
n=300
n=976
n=1597
n=589
PRS: Educational attainment
0.2
0.0
−0.2
−0.4
−0.6
−0.8
completely disagree
disagree
neutral
agree
completely agree
Recovering quickly after difficult times

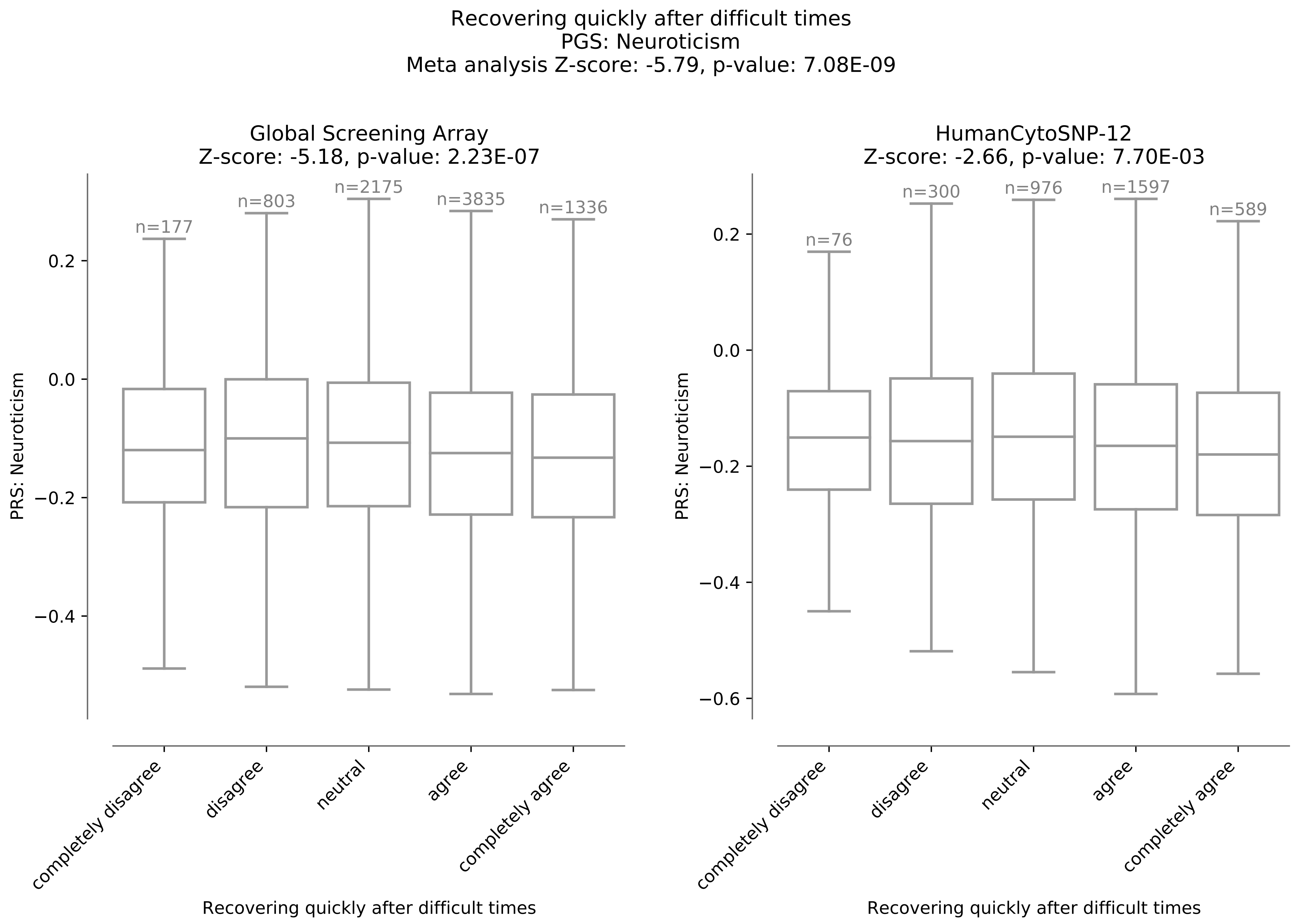

Recovering quickly after difficult times
PGS: Neuroticism
Meta analysis Z-score: -5.79, p-value: 7.08E-09
Global Screening Array
Z-score: -5.18, p-value: 2.23E-07
n=177
n=803
n=2175
n=3835
n=1336
PRS: Neuroticism
0.2
0.0
−0.2
−0.4
completely disagree
disagree
neutral
agree
completely agree
Recovering quickly after difficult times
HumanCytoSNP-12
Z-score: -2.66, p-value: 7.70E-03
n=76
n=300
n=976
n=1597
n=589
PRS: Neuroticism
0.2
0.0
−0.2
−0.4
−0.6
completely disagree
disagree
neutral
agree
completely agree
Recovering quickly after difficult times

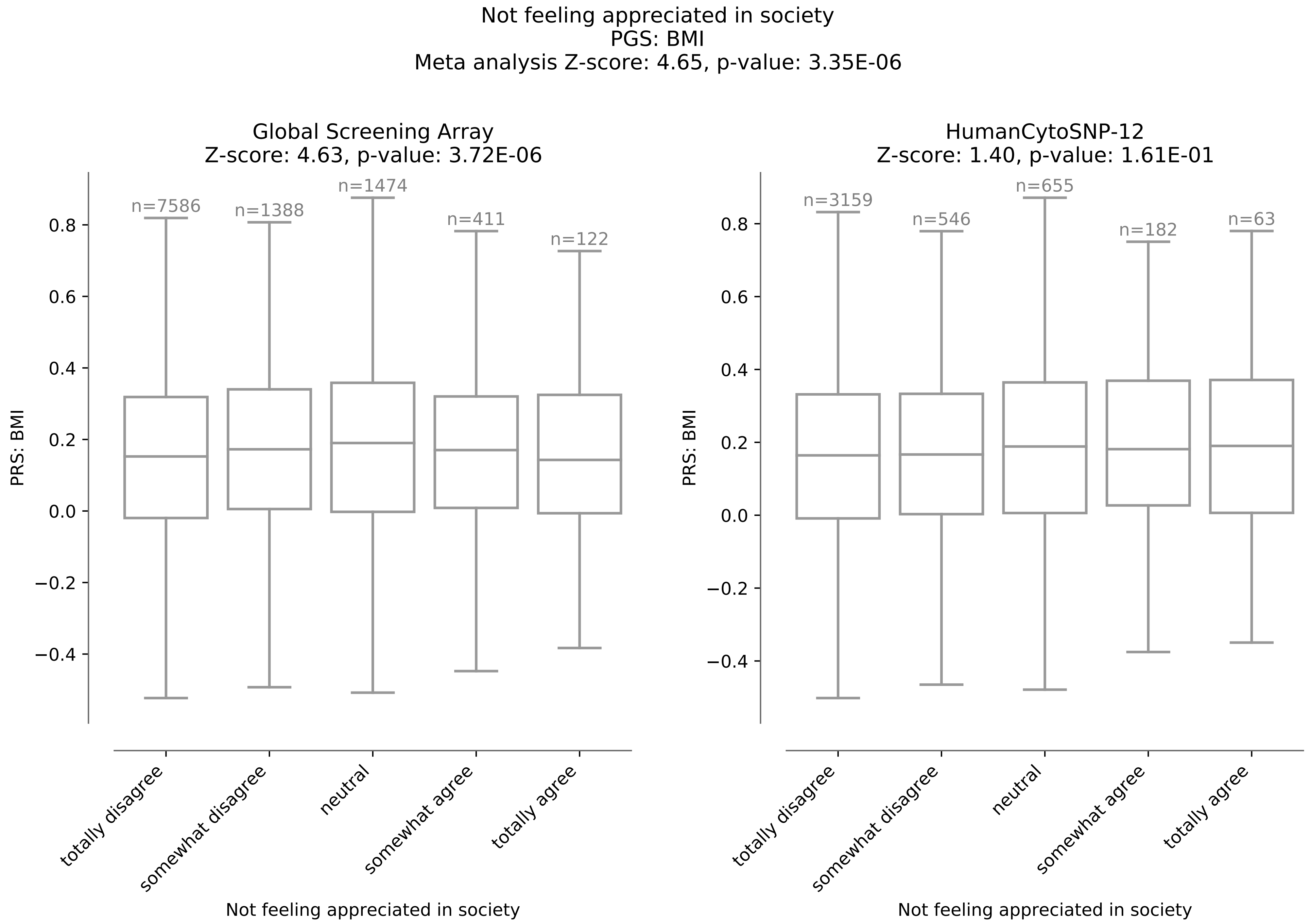
Not feeling appreciated in society
PGS: BMI
Meta analysis Z-score: 4.65, p-value: 3.35E-06
Global Screening Array
Z-score: 4.63, p-value: 3.72E-06
HumanCytoSNP-12
Z-score: 1.40, p-value: 1.61E-01
PRS: BMI
0.8
0.6
0.4
0.2
0.0
−0.2
−0.4
n=7586
n=1388
n=1474
n=411
n=122
n=3159
n=546
n=655
n=182
n=63
totally disagree
somewhat disagree
neutral
somewhat agree
totally agree
Not feeling appreciated in society

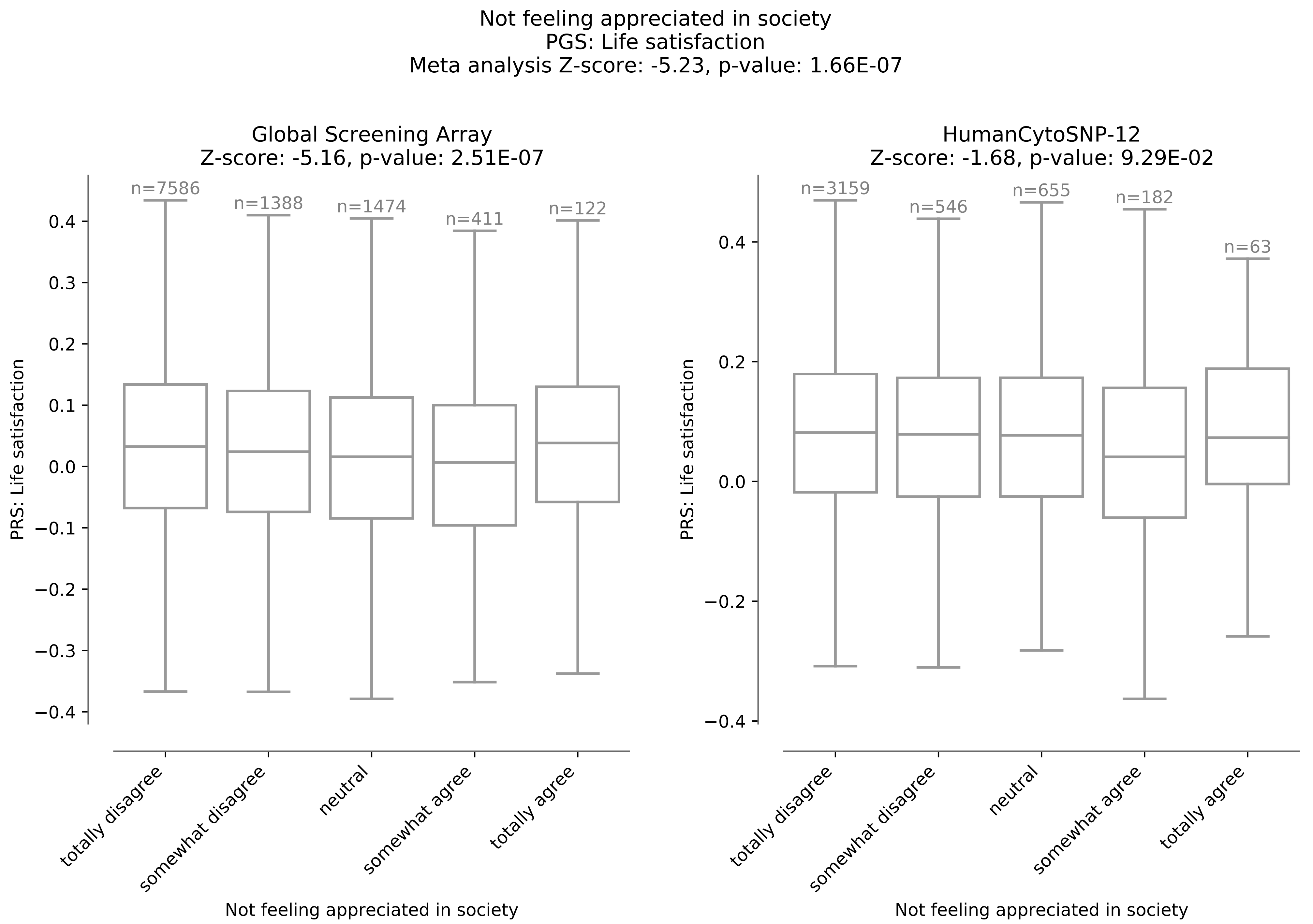

Not feeling appreciated in society
PGS: Life satisfaction
Meta analysis Z-score: -5.23, p-value: 1.66E-07
Global Screening Array
Z-score: -5.16, p-value: 2.51E-07
HumanCytoSNP-12
Z-score: -1.68, p-value: 9.29E-02
PRS: Life satisfaction
n=7586
n=1388
n=1474
n=411
n=122
n=3159
n=546
n=655
n=182
n=63
0.4
0.3
0.2
0.1
0.0
−0.1
−0.2
−0.3
−0.4
totally disagree
somewhat disagree
neutral
somewhat agree
totally agree
Not feeling appreciated in society

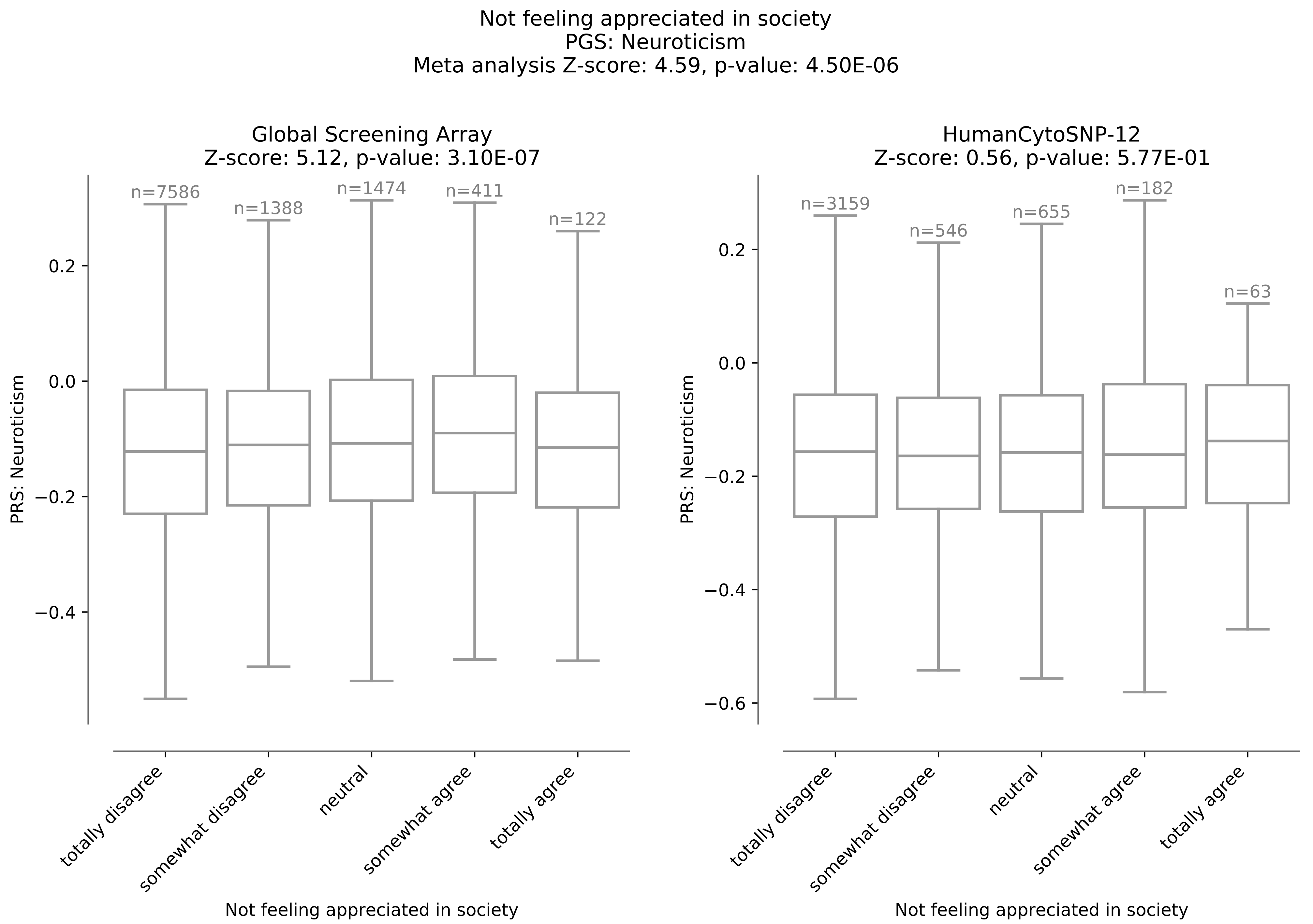
Not feeling appreciated in society
PGS: Neuroticism
Meta analysis Z-score: 4.59, p-value: 4.50E-06
Global Screening Array
Z-score: 5.12, p-value: 3.10E-07
HumanCytoSNP-12
Z-score: 0.56, p-value: 5.77E-01
n=7586
n=1388
n=1474
n=411
n=122
n=3159
n=546
n=655
n=182
n=63
PRS: Neuroticism
0.2
0.0
−0.2
−0.4
−0.6
totally disagree
somewhat disagree
neutral
somewhat agree
totally agree
Not feeling appreciated in society

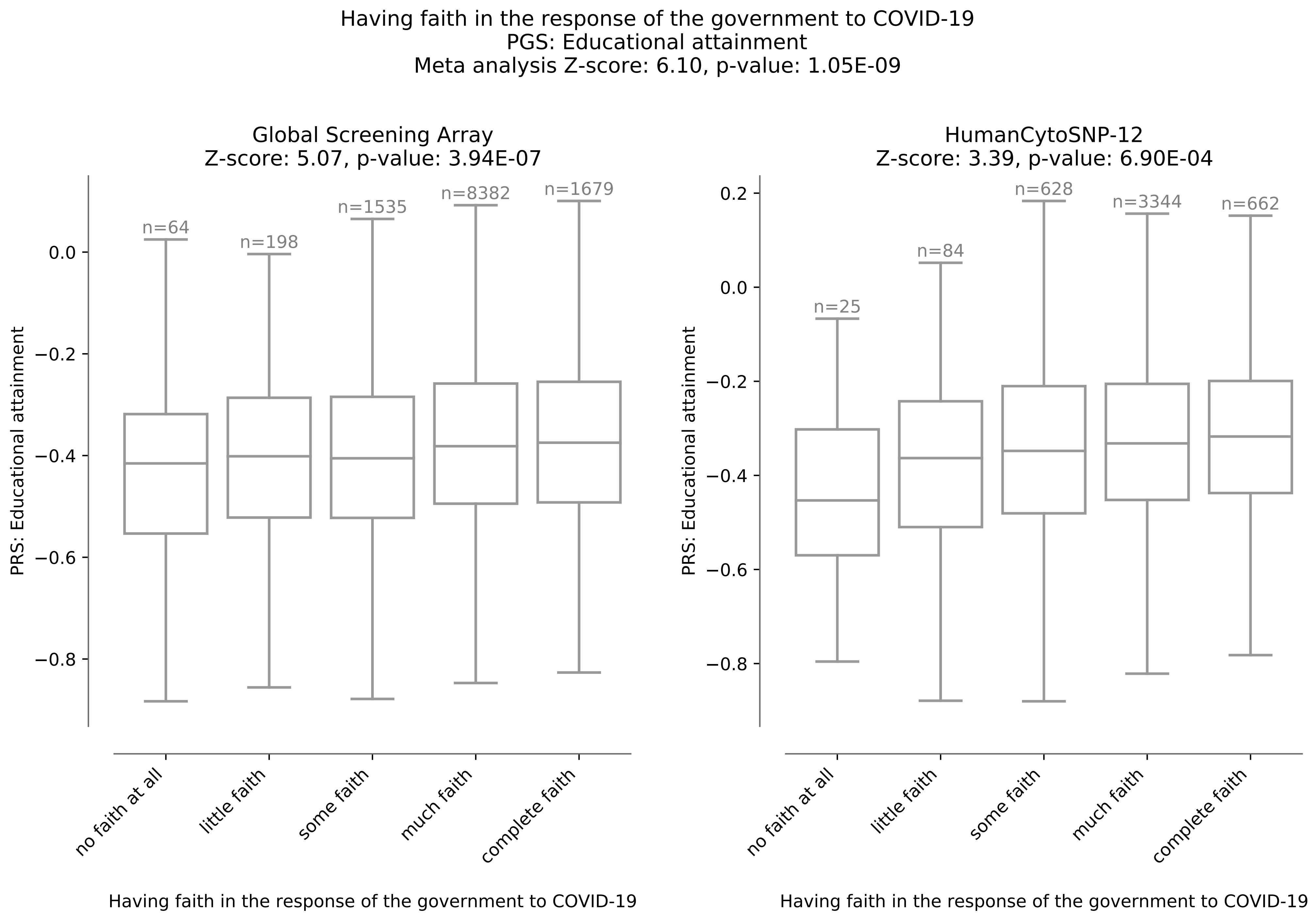
Having faith in the response of the government to COVID-19
PGS: Educational attainment
Meta analysis Z-score: 6.10, p-value: 1.05E-09
Global Screening Array
Z-score: 5.07, p-value: 3.94E-07
HumanCytoSNP-12
Z-score: 3.39, p-value: 6.90E-04
PRS: Educational attainment
n=64
n=198
n=1535
n=8382
n=1679
n=25
n=84
n=628
n=3344
n=662
0.2
0.0
−0.2
−0.4
−0.6
−0.8
no faith at all
little faith
some faith
much faith
complete faith
Having faith in the response of the government to COVID-19

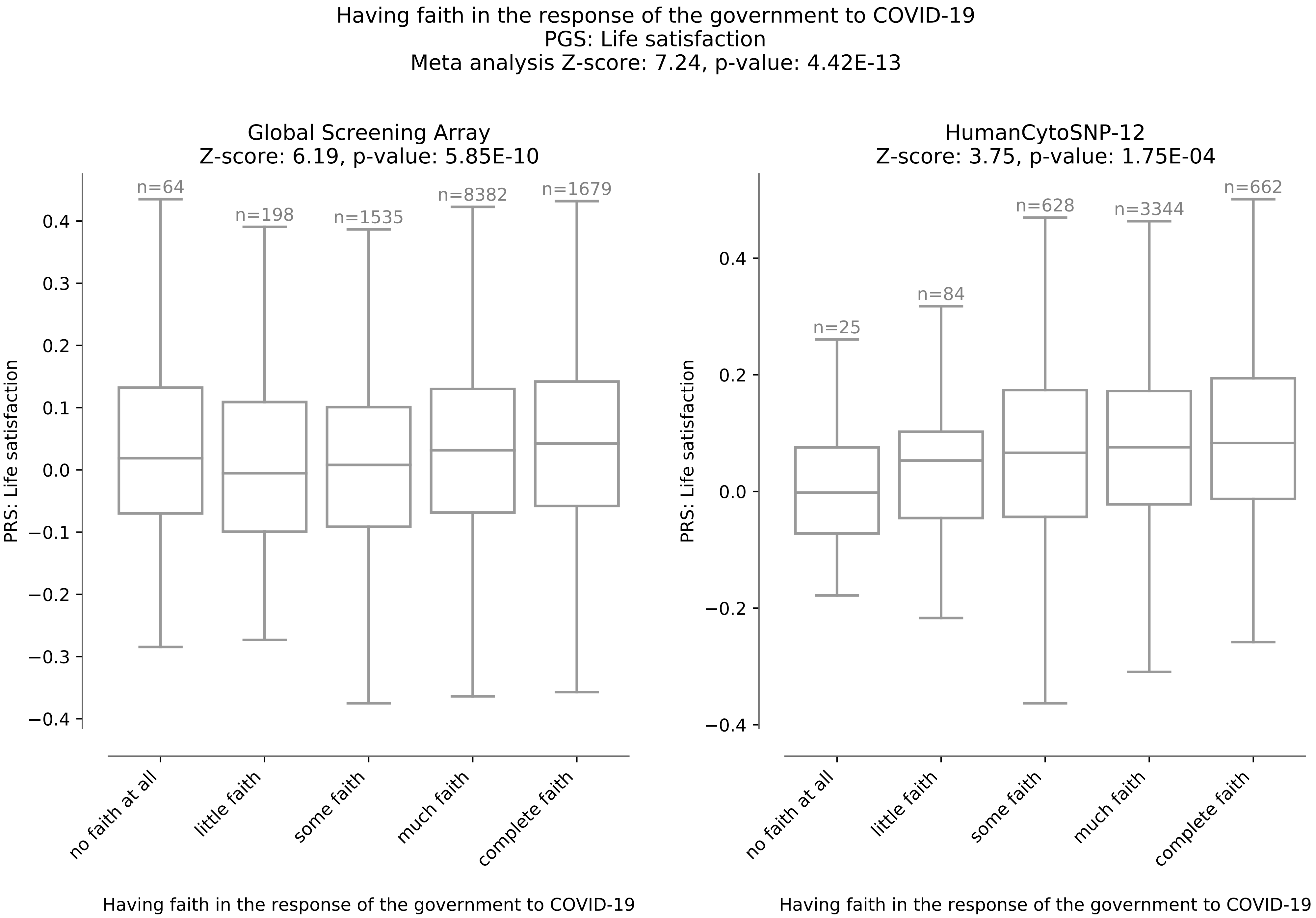
Having faith in the response of the government to COVID-19
PGS: Life satisfaction
Meta analysis Z-score: 7.24, p-value: 4.42E-13
Global Screening Array
Z-score: 6.19, p-value: 5.85E-10
HumanCytoSNP-12
Z-score: 3.75, p-value: 1.75E-04
PRS: Life satisfaction
n=64
n=198
n=1535
n=8382
n=1679
n=25
n=84
n=628
n=3344
n=662
0.4
0.3
0.2
0.1
0.0
−0.1
−0.2
−0.3
−0.4
no faith at all
little faith
some faith
much faith
complete faith
Having faith in the response of the government to COVID-19

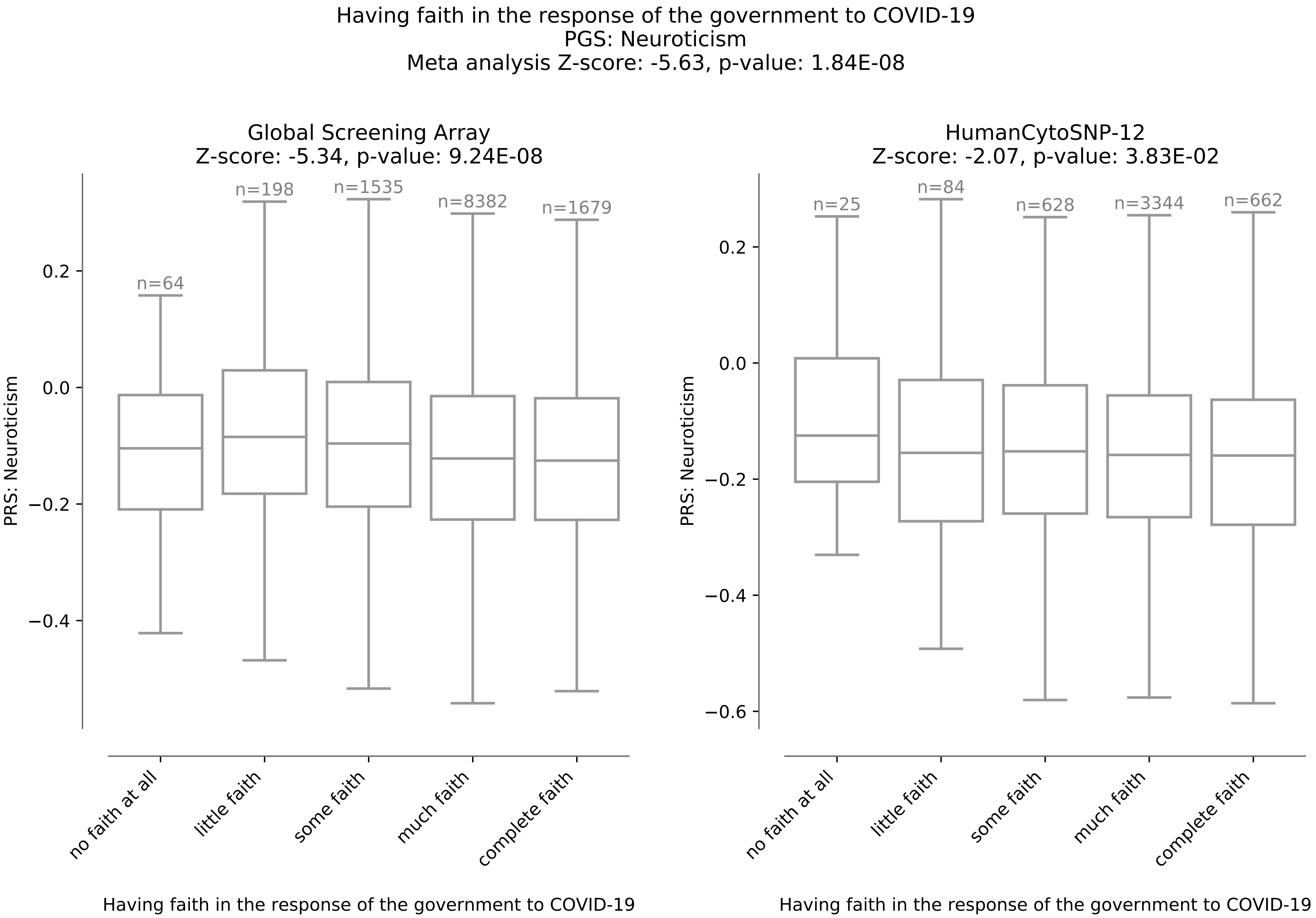
Having faith in the response of the government to COVID-19
PGS: Neuroticism
Meta analysis Z-score: -5.63, p-value: 1.84E-08
Global Screening Array
Z-score: -5.34, p-value: 9.24E-08
HumanCytoSNP-12
Z-score: -2.07, p-value: 3.83E-02
PRS: Neuroticism
n=64
n=198
n=1535
n=8382
n=1679
n=25
n=84
n=628
n=3344
n=662
no faith at all
little faith
some faith
much faith
complete faith
Having faith in the response of the government to COVID-19

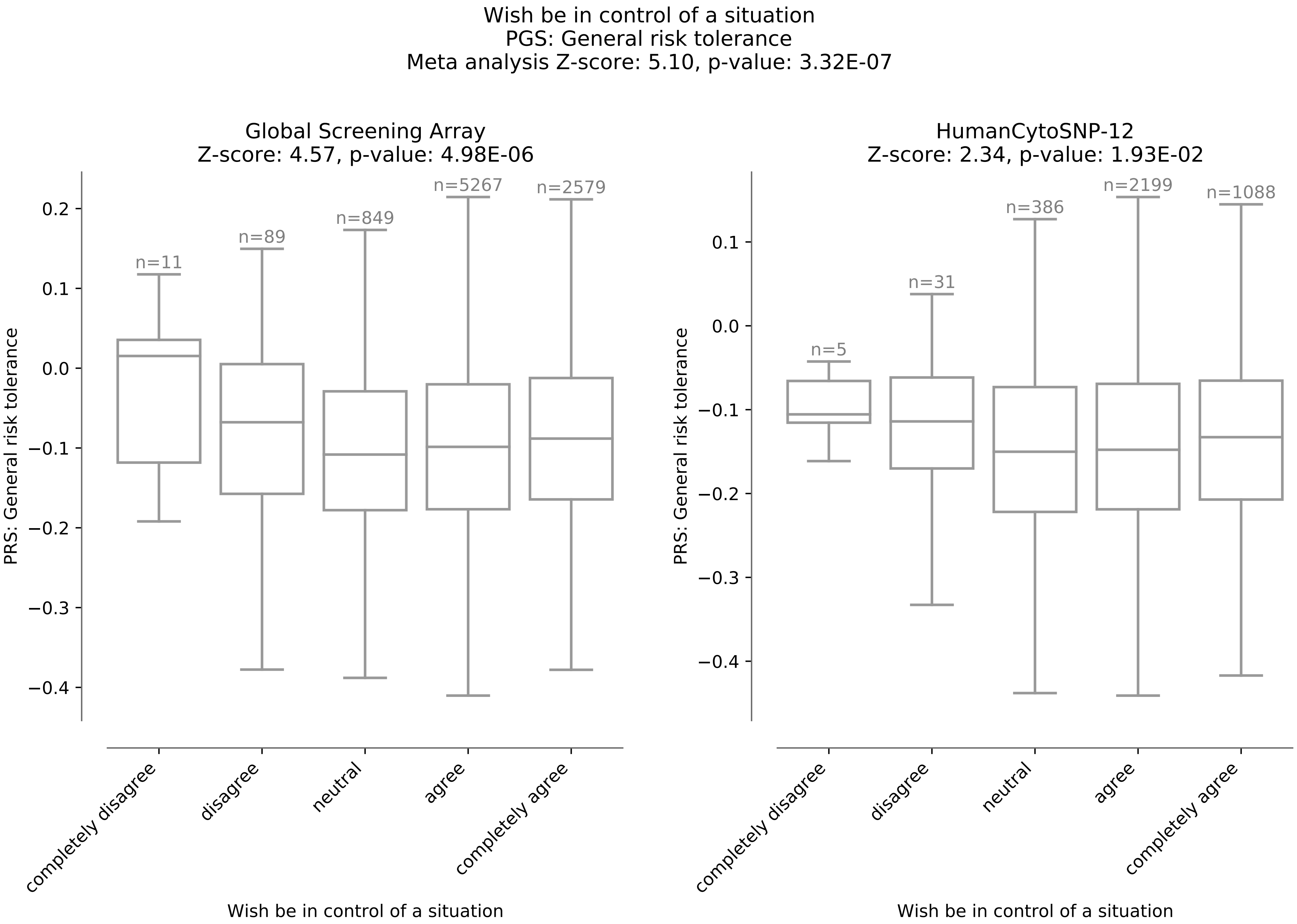

Wish be in control of a situation
PGS: General risk tolerance
Meta analysis Z-score: 5.10, p-value: 3.32E-07
Global Screening Array
Z-score: 4.57, p-value: 4.98E-06
HumanCytoSNP-12
Z-score: 2.34, p-value: 1.93E-02
PRS: General risk tolerance
n=11
n=89
n=849
n=5267
n=2579
n=5
n=31
n=386
n=2199
n=1088
completely disagree
disagree
neutral
agree
completely agree
Wish be in control of a situation

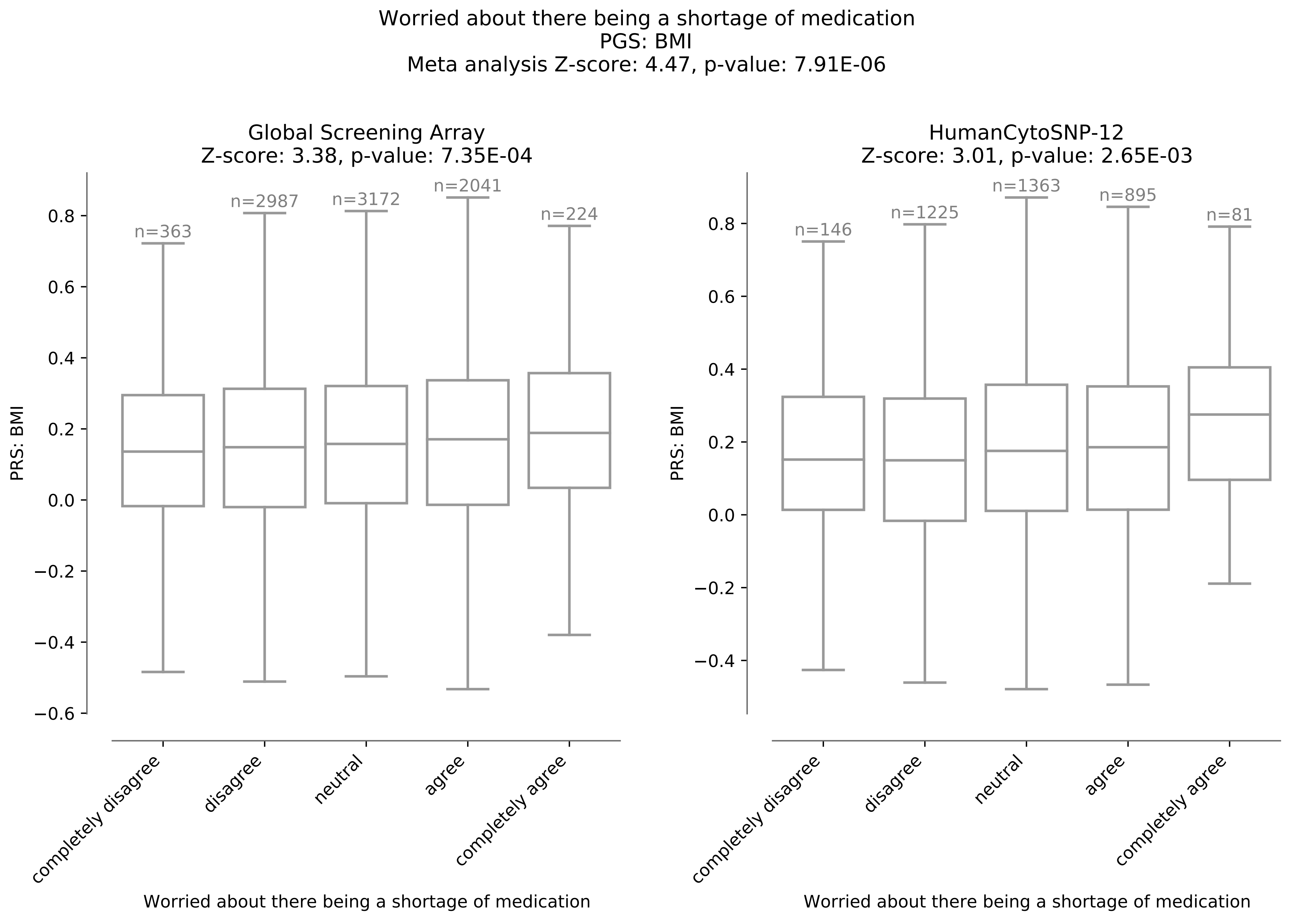
Worried about there being a shortage of medication
PGS: BMI
Meta analysis Z-score: 4.47, p-value: 7.91E-06
Global Screening Array
Z-score: 3.38, p-value: 7.35E-04
HumanCytoSNP-12
Z-score: 3.01, p-value: 2.65E-03
PRS: BMI
n=363
n=2987
n=3172
n=2041
n=224
n=146
n=1225
n=1363
n=895
n=81
completely disagree
disagree
neutral
agree
completely agree
Worried about there being a shortage of medication

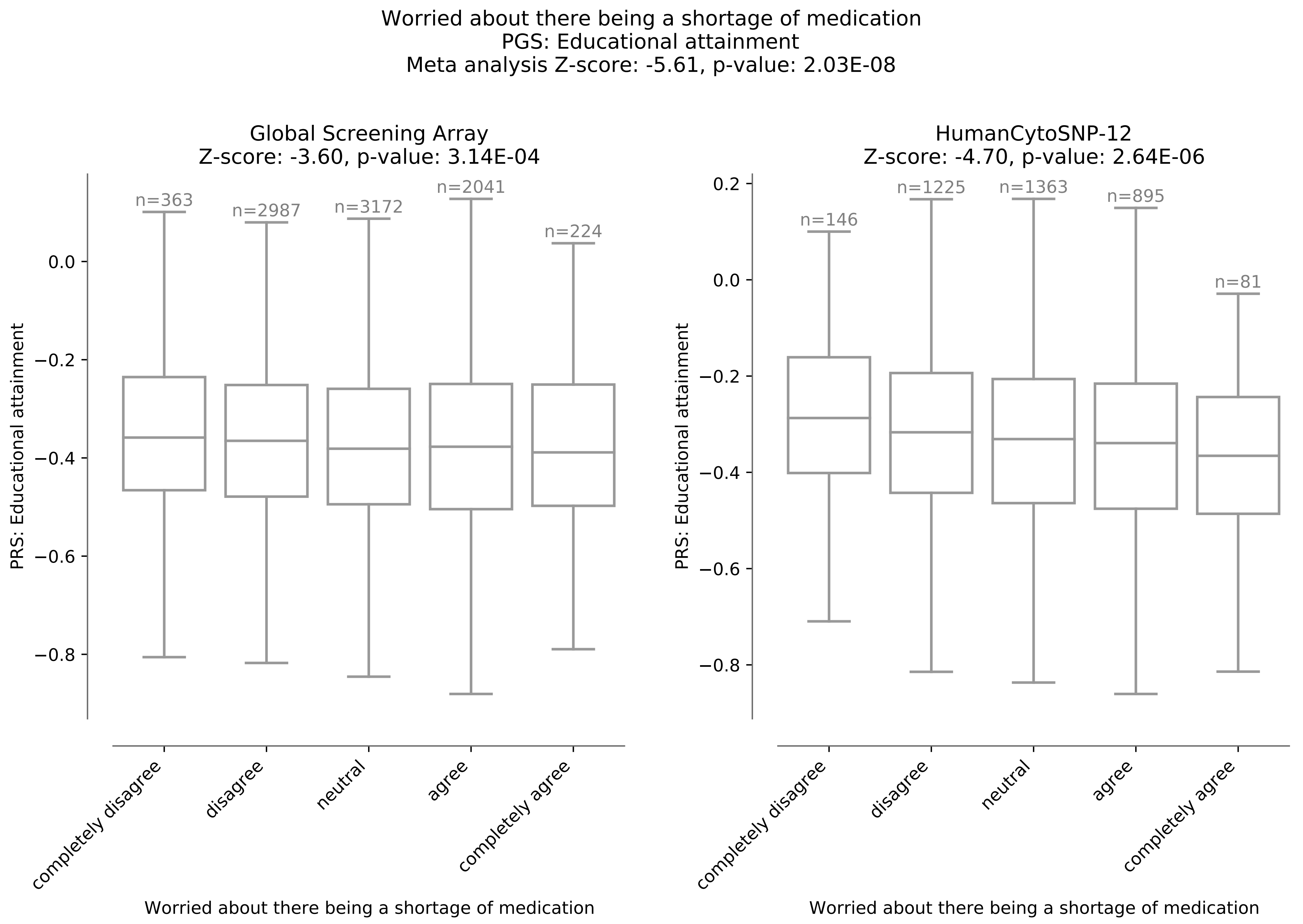
Worried about there being a shortage of medication
PGS: Educational attainment
Meta analysis Z-score: -5.61, p-value: 2.03E-08
Global Screening Array
Z-score: -3.60, p-value: 3.14E-04
n=363
n=2987
n=3172
n=2041
n=224
PRS: Educational attainment
0.0
−0.2
−0.4
−0.6
−0.8
completely disagree
disagree
neutral
agree
completely agree
Worried about there being a shortage of medication
HumanCytoSNP-12
Z-score: -4.70, p-value: 2.64E-06
n=146
n=1225
n=1363
n=895
n=81
PRS: Educational attainment
0.2
0.0
−0.2
−0.4
−0.6
−0.8
completely disagree
disagree
neutral
agree
completely agree
Worried about there being a shortage of medication

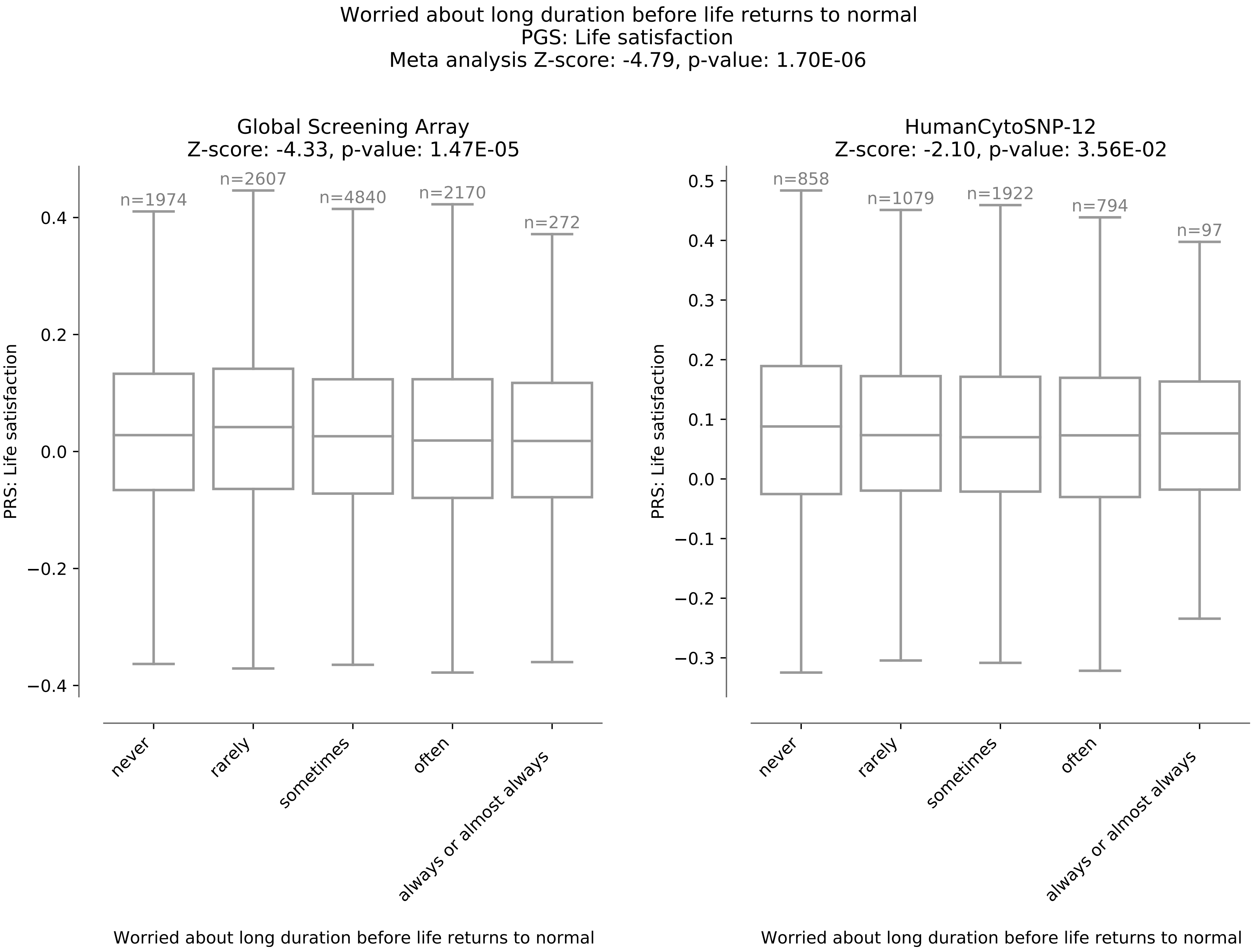

Worried about long duration before life returns to normal
PGS: Life satisfaction
Meta analysis Z-score: -4.79, p-value: 1.70E-06
Global Screening Array
Z-score: -4.33, p-value: 1.47E-05
n=1974
n=2607
n=4840
n=2170
n=272
PRS: Life satisfaction
0.4
0.2
0.0
−0.2
−0.4
never
rarely
sometimes
often
always or almost always
Worried about long duration before life returns to normal
HumanCytoSNP-12
Z-score: -2.10, p-value: 3.56E-02
n=858
n=1079
n=1922
n=794
n=97
PRS: Life satisfaction
0.5
0.4
0.3
0.2
0.1
0.0
−0.1
−0.2
−0.3
never
rarely
sometimes
often
always or almost always
Worried about long duration before life returns to normal

Worried about long duration before life returns to normal
PGS: Neuroticism
Meta analysis Z-score: 6.17, p-value: 6.71E-10

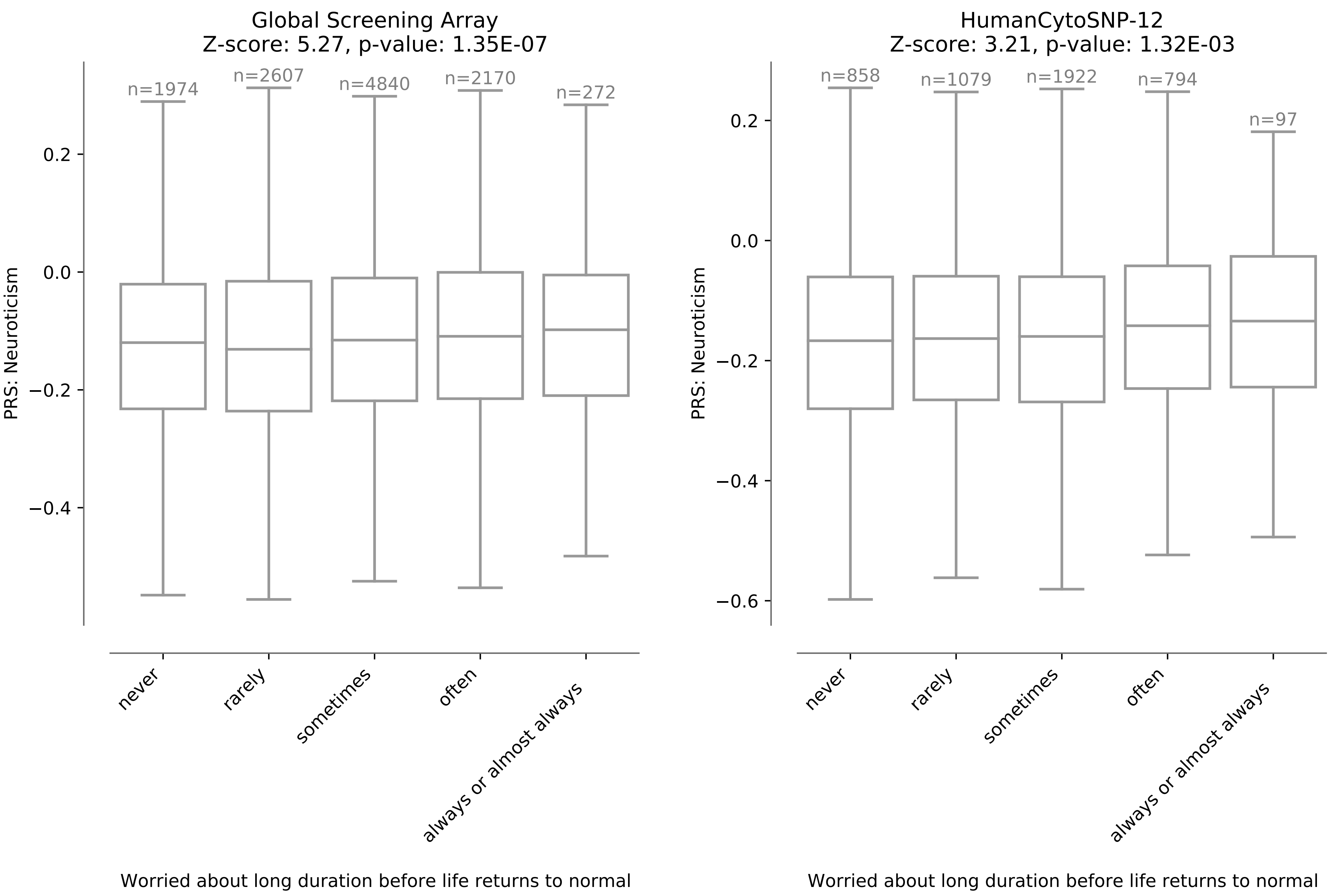

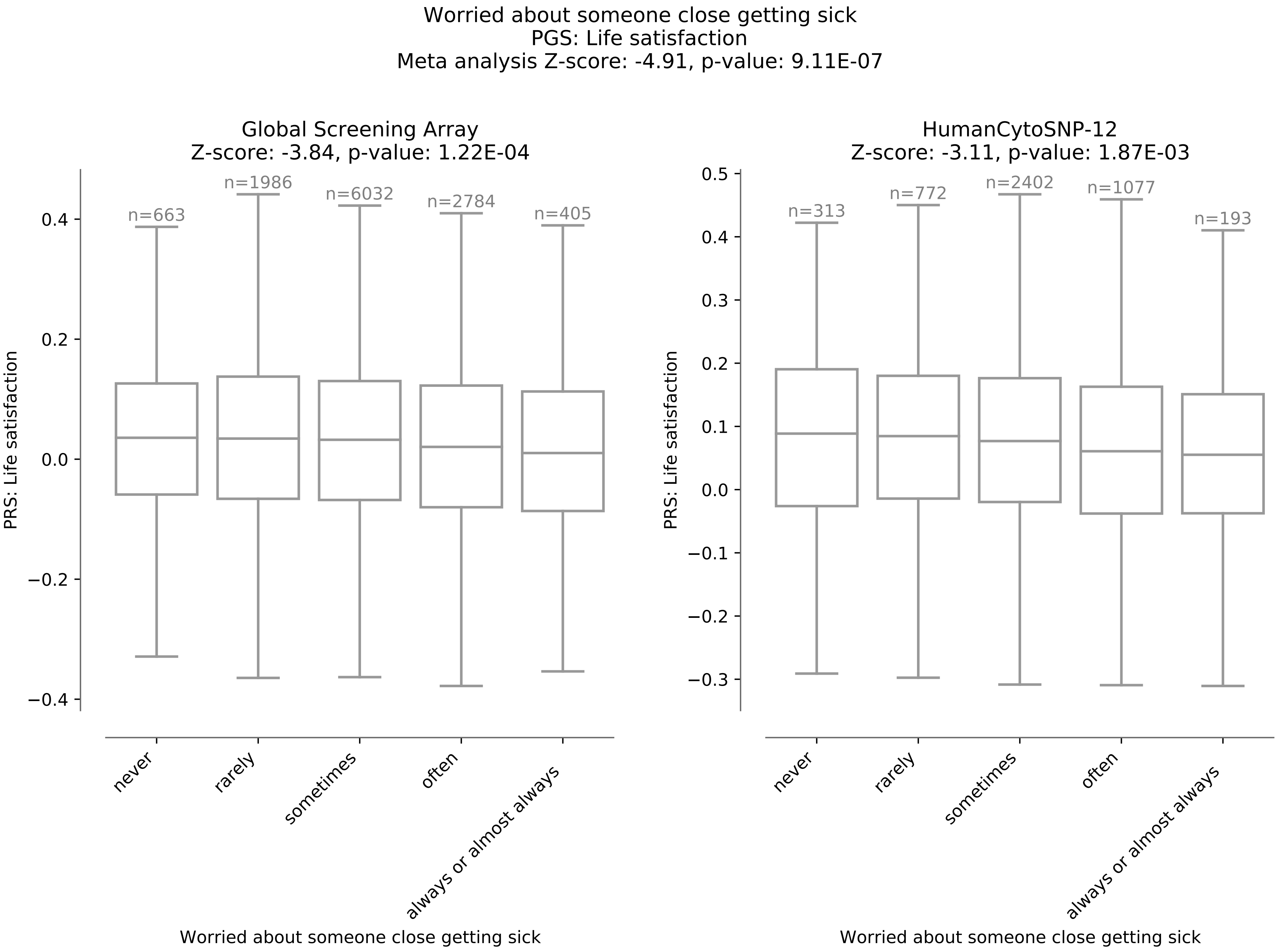

Worried about someone close getting sick
PGS: Life satisfaction
Meta analysis Z-score: -4.91, p-value: 9.11E-07
Global Screening Array
Z-score: -3.84, p-value: 1.22E-04
n=663
n=1986
n=6032
n=2784
n=405
PRS: Life satisfaction
0.4
0.2
0.0
−0.2
−0.4
never
rarely
sometimes
often
always or almost always
Worried about someone close getting sick
HumanCytoSNP-12
Z-score: -3.11, p-value: 1.87E-03
n=313
n=772
n=2402
n=1077
n=193
PRS: Life satisfaction
0.5
0.4
0.3
0.2
0.1
0.0
−0.1
−0.2
−0.3
never
rarely
sometimes
often
always or almost always
Worried about someone close getting sick

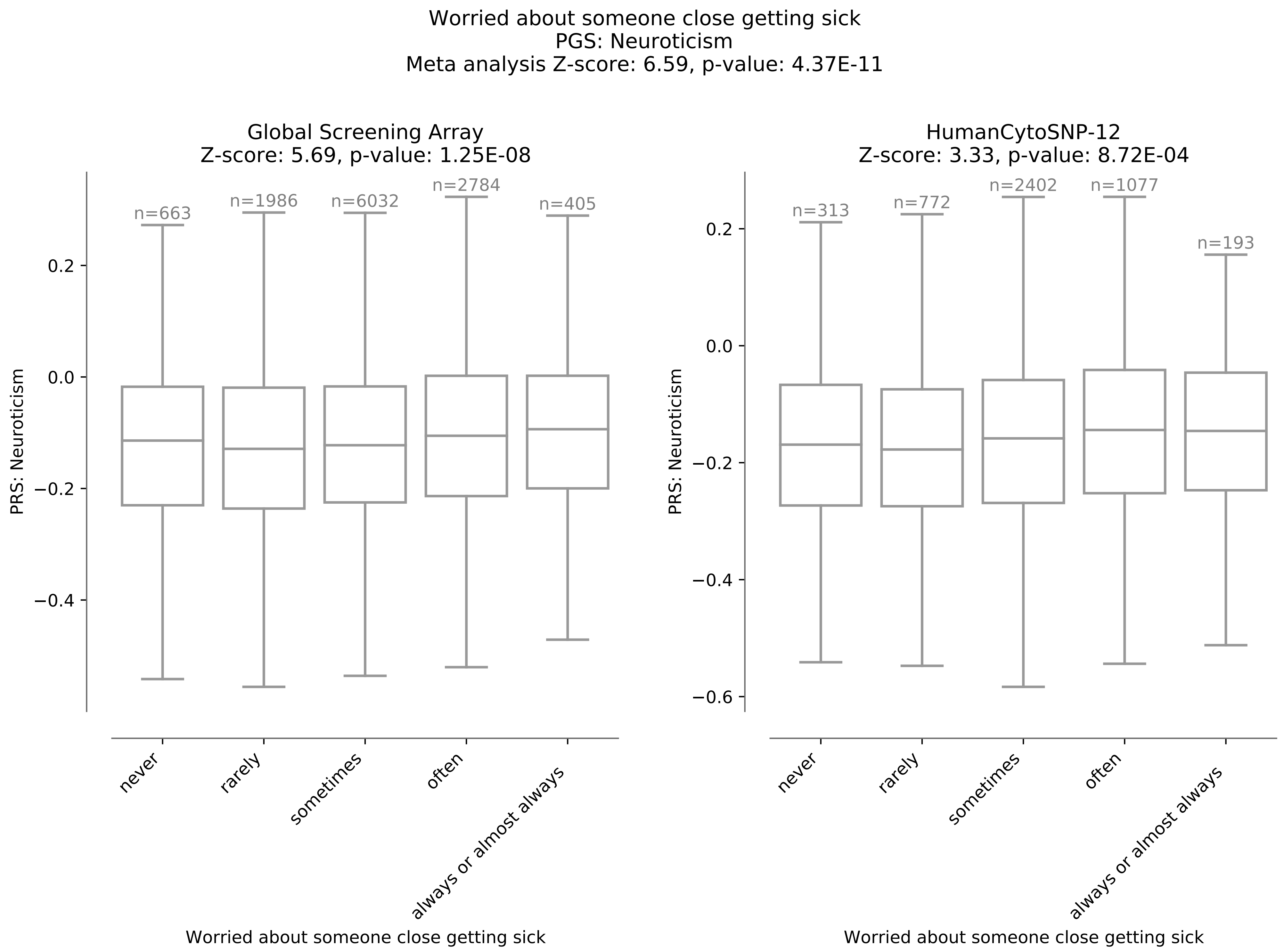

Worried about someone close getting sick
PGS: Neuroticism
Meta analysis Z-score: 6.59, p-value: 4.37E-11
Global Screening Array
Z-score: 5.69, p-value: 1.25E-08
HumanCytoSNP-12
Z-score: 3.33, p-value: 8.72E-04
n=663
n=1986
n=6032
n=2784
n=405
n=313
n=772
n=2402
n=1077
n=193
0.2
0.0
−0.2
−0.4
−0.6
PRS: Neuroticism
never
rarely
sometimes
often
always or almost always
Worried about someone close getting sick

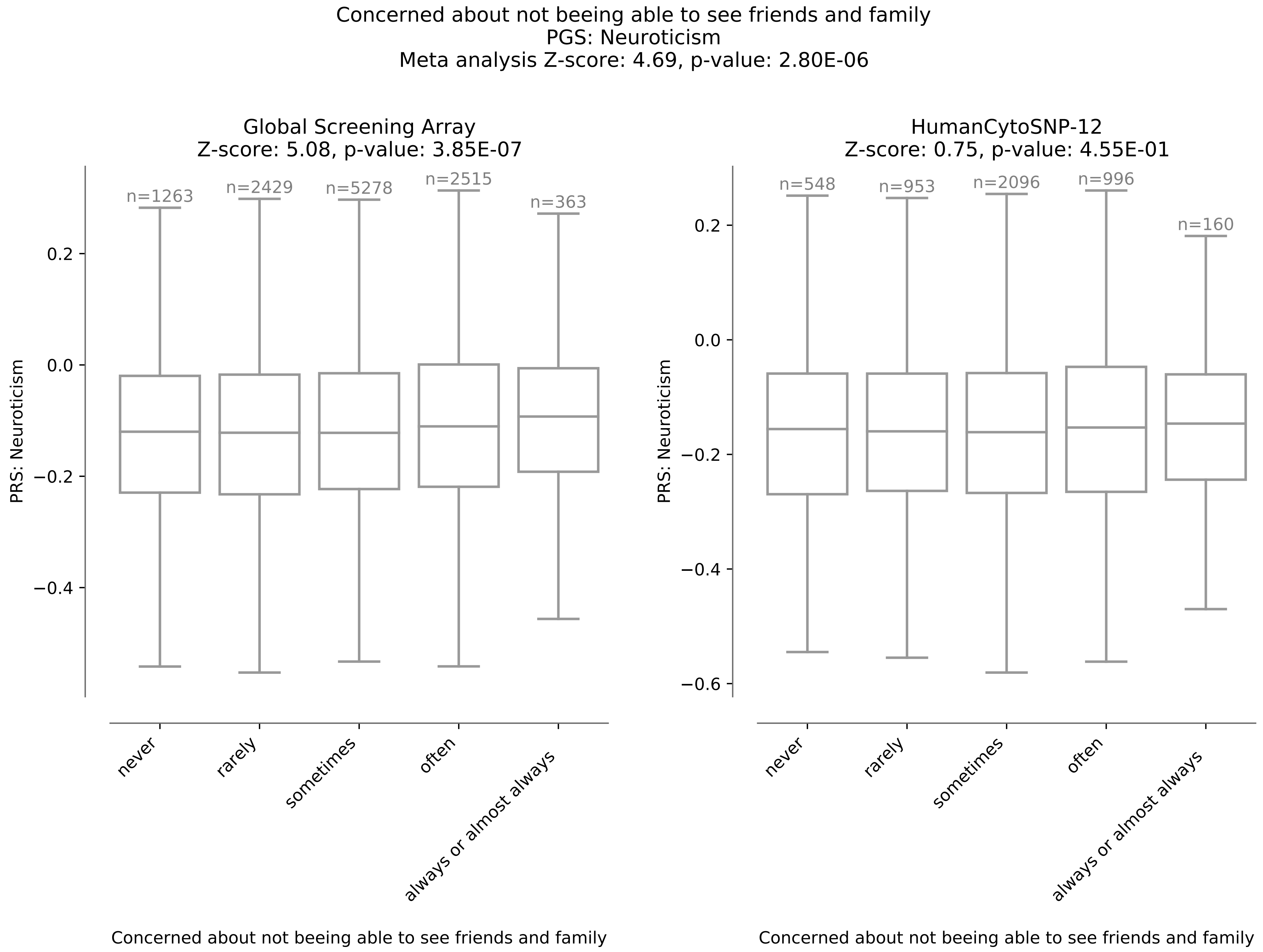

Concerned about not beeing able to see friends and family
PGS: Neuroticism
Meta analysis Z-score: 4.69, p-value: 2.80E-06
Global Screening Array
Z-score: 5.08, p-value: 3.85E-07
HumanCytoSNP-12
Z-score: 0.75, p-value: 4.55E-01
PRS: Neuroticism
n=1263
n=2429
n=5278
n=2515
n=363
n=548
n=953
n=2096
n=996
n=160
0.2
0.0
−0.2
−0.4
−0.6
never
rarely
sometimes
often
always or almost always
Concerned about not beeing able to see friends and family

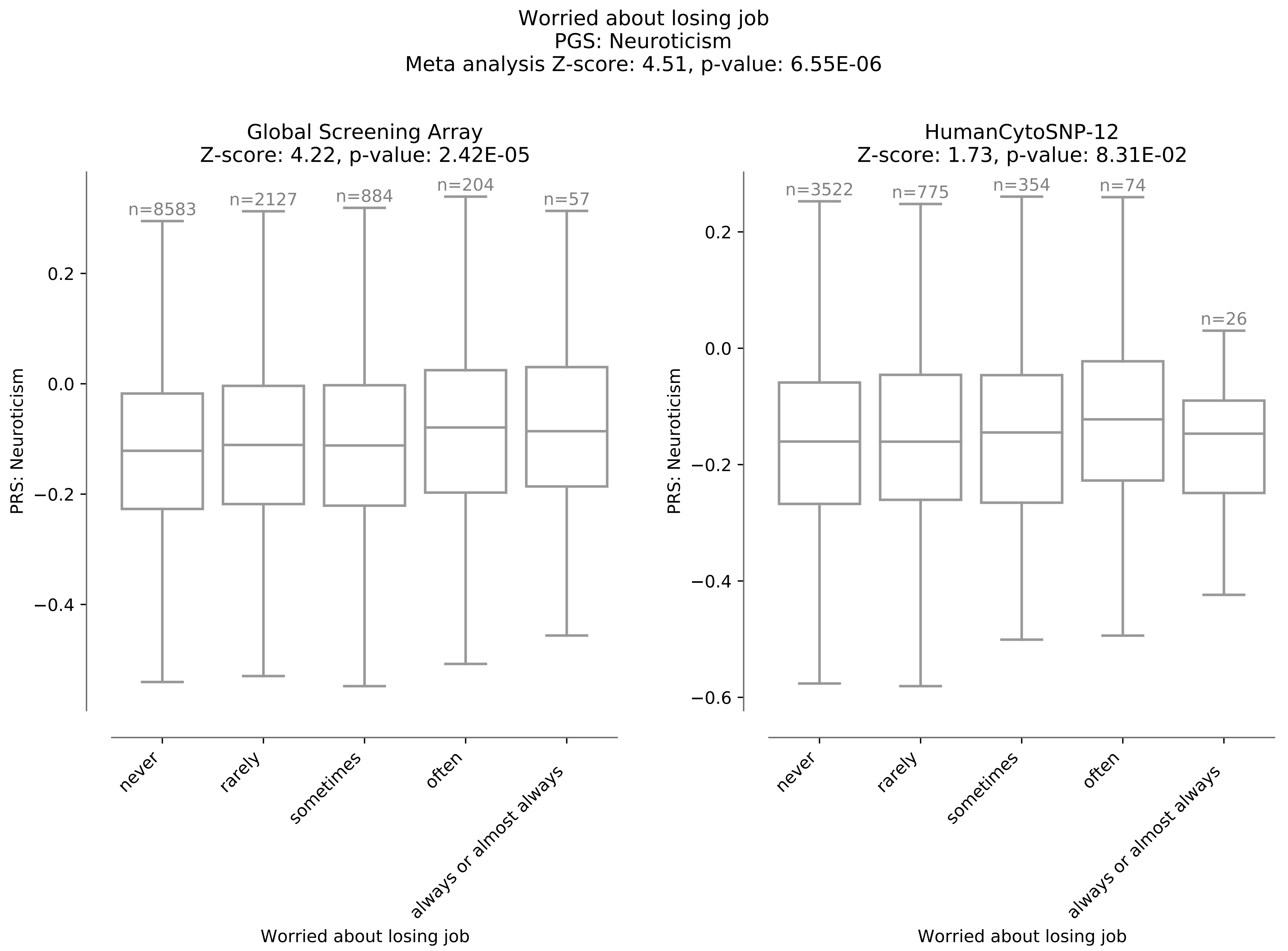
Worried about losing job
PGS: Neuroticism
Meta analysis Z-score: 4.51, p-value: 6.55E-06
Global Screening Array
Z-score: 4.22, p-value: 2.42E-05
n=8583
n=2127
n=884
n=204
n=57
0.2
0.0
−0.2
−0.4
PRS: Neuroticism
never
rarely
sometimes
often
always or almost always
Worried about losing job
HumanCytoSNP-12
Z-score: 1.73, p-value: 8.31E-02
n=3522
n=775
n=354
n=74
n=26
0.2
0.0
−0.2
−0.4
−0.6
PRS: Neuroticism
never
rarely
sometimes
often
always or almost always
Worried about losing job

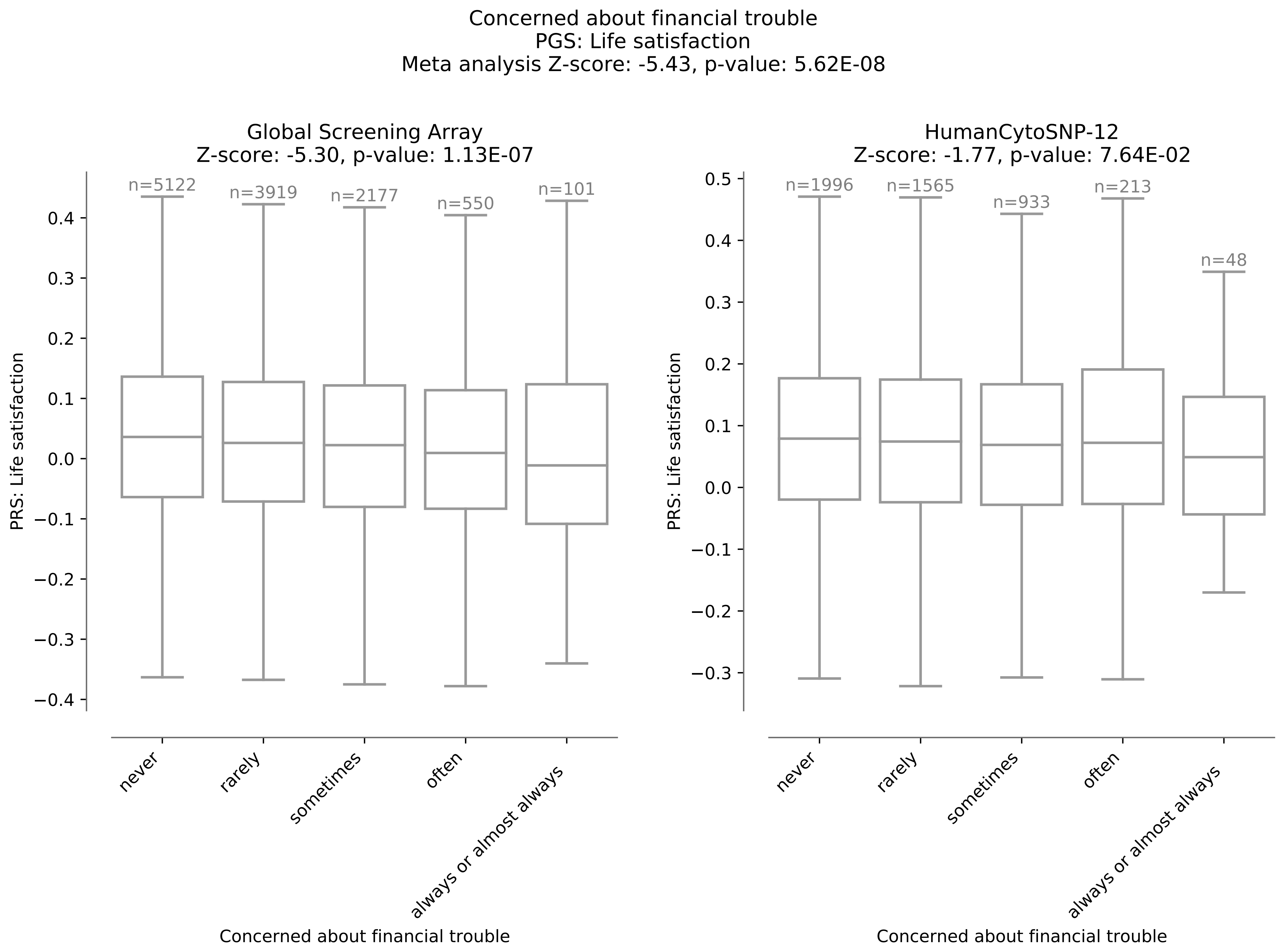

Concerned about financial trouble
PGS: Life satisfaction
Meta analysis Z-score: -5.43, p-value: 5.62E-08
Global Screening Array
Z-score: -5.30, p-value: 1.13E-07
HumanCytoSNP-12
Z-score: -1.77, p-value: 7.64E-02
PRS: Life satisfaction
n=5122
n=3919
n=2177
n=550
n=101
n=1996
n=1565
n=933
n=213
n=48
never
rarely
sometimes
often
always or almost always
Concerned about financial trouble

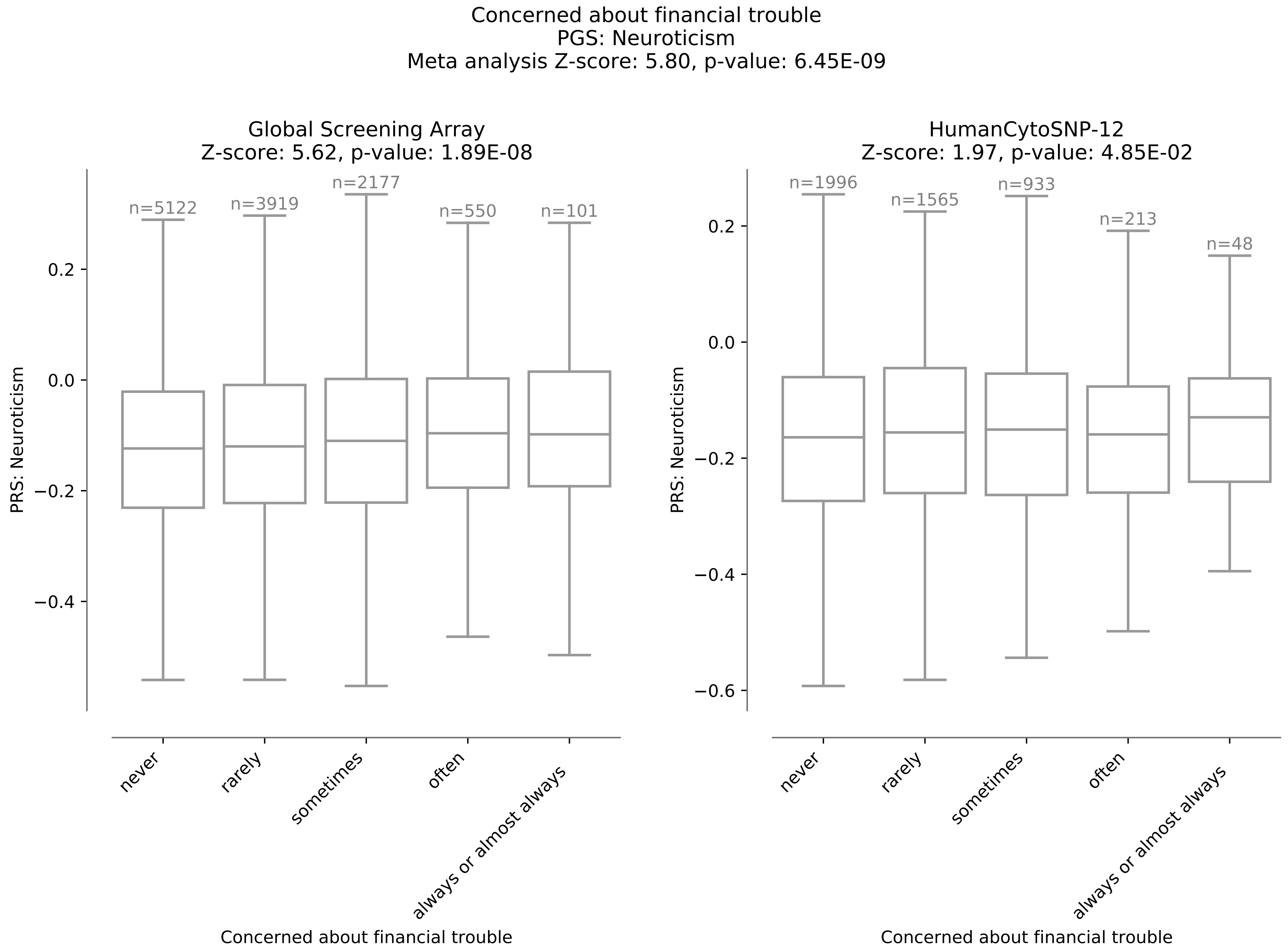
Concerned about financial trouble
PGS: Neuroticism
Meta analysis Z-score: 5.80, p-value: 6.45E-09
Global Screening Array
Z-score: 5.62, p-value: 1.89E-08
n=5122
n=3919
n=2177
n=550
n=101
PRS: Neuroticism
0.2
0.0
−0.2
−0.4
never
rarely
sometimes
often
always or almost always
Concerned about financial trouble
HumanCytoSNP-12
Z-score: 1.97, p-value: 4.85E-02
n=1996
n=1565
n=933
n=213
n=48
PRS: Neuroticism
0.2
0.0
−0.2
−0.4
−0.6
never
rarely
sometimes
often
always or almost always
Concerned about financial trouble

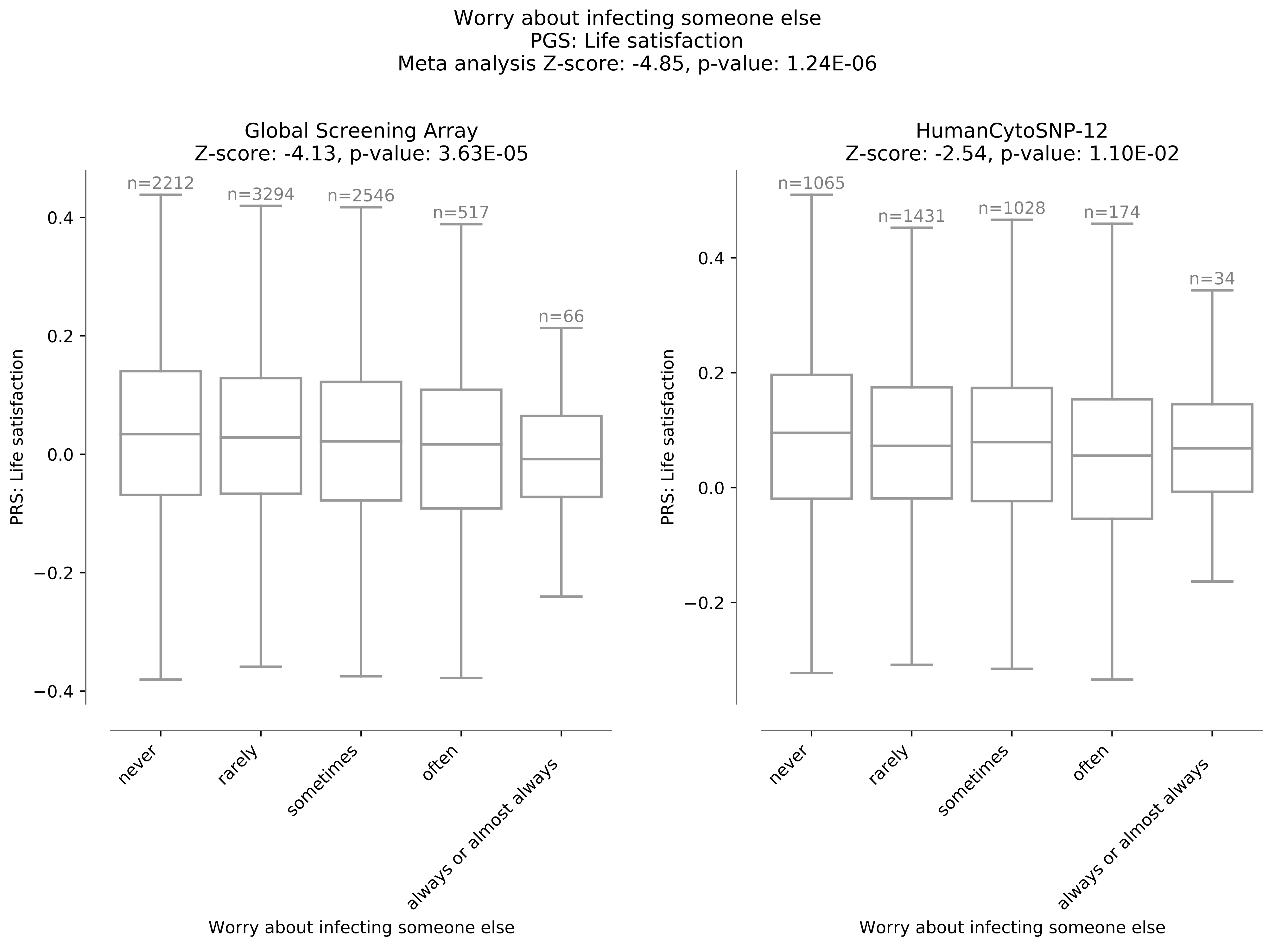

Worry about infecting someone else
PGS: Life satisfaction
Meta analysis Z-score: -4.85, p-value: 1.24E-06
Global Screening Array
Z-score: -4.13, p-value: 3.63E-05
HumanCytoSNP-12
Z-score: -2.54, p-value: 1.10E-02
PRS: Life satisfaction
n=2212
n=3294
n=2546
n=517
n=66
n=1065
n=1431
n=1028
n=174
n=34
0.4
0.2
0.0
−0.2
−0.4
never
rarely
sometimes
often
always or almost always
Worry about infecting someone else

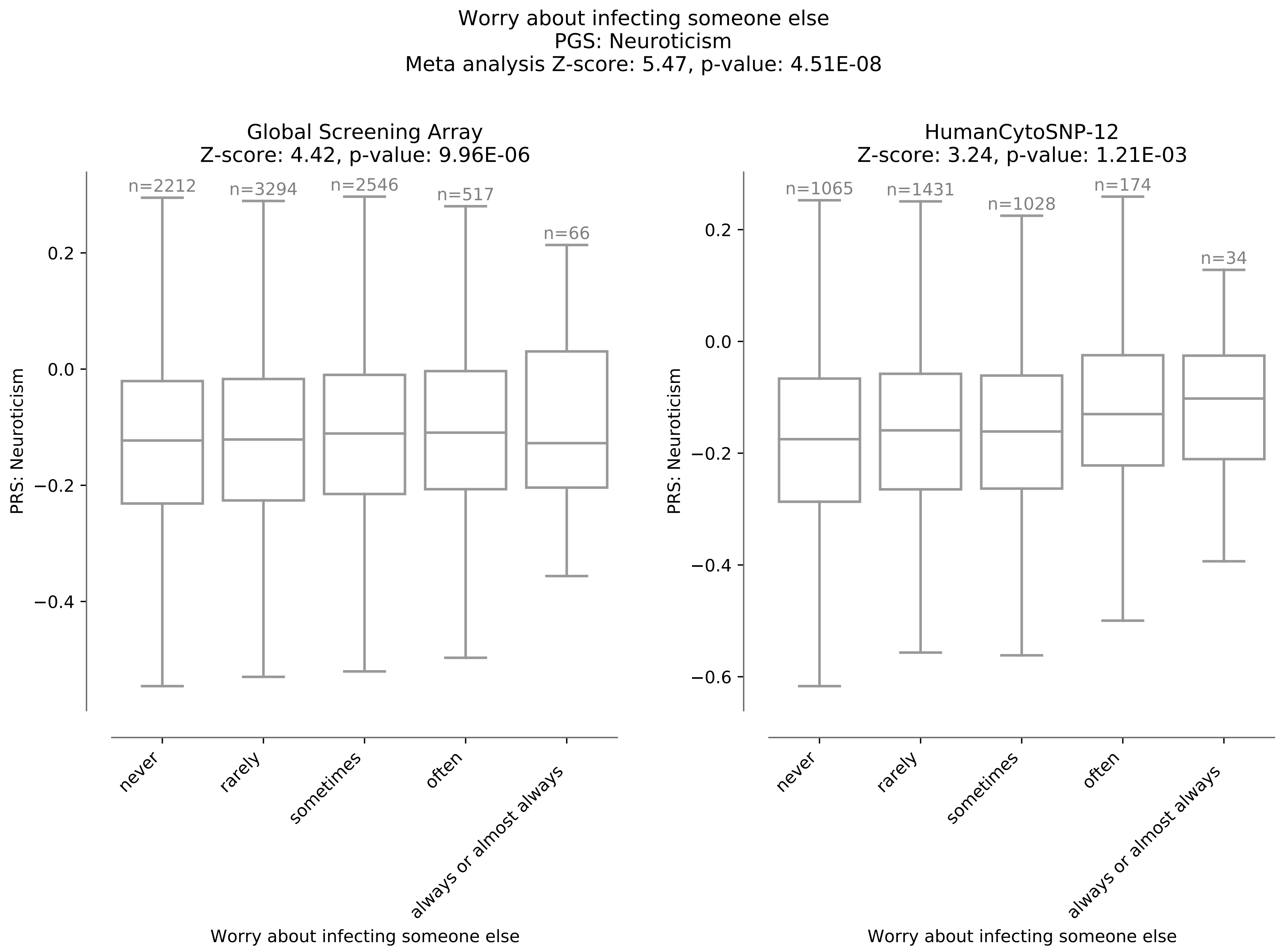
Worry about infecting someone else
PGS: Neuroticism
Meta analysis Z-score: 5.47, p-value: 4.51E-08
Global Screening Array
Z-score: 4.42, p-value: 9.96E-06
n=2212
n=3294
n=2546
n=517
n=66
0.2
0.0
−0.2
−0.4
PRS: Neuroticism
never
rarely
sometimes
often
always or almost always
Worry about infecting someone else
HumanCytoSNP-12
Z-score: 3.24, p-value: 1.21E-03
n=1065
n=1431
n=1028
n=174
n=34
0.2
0.0
−0.2
−0.4
−0.6
PRS: Neuroticism
never
rarely
sometimes
often
always or almost always
Worry about infecting someone else

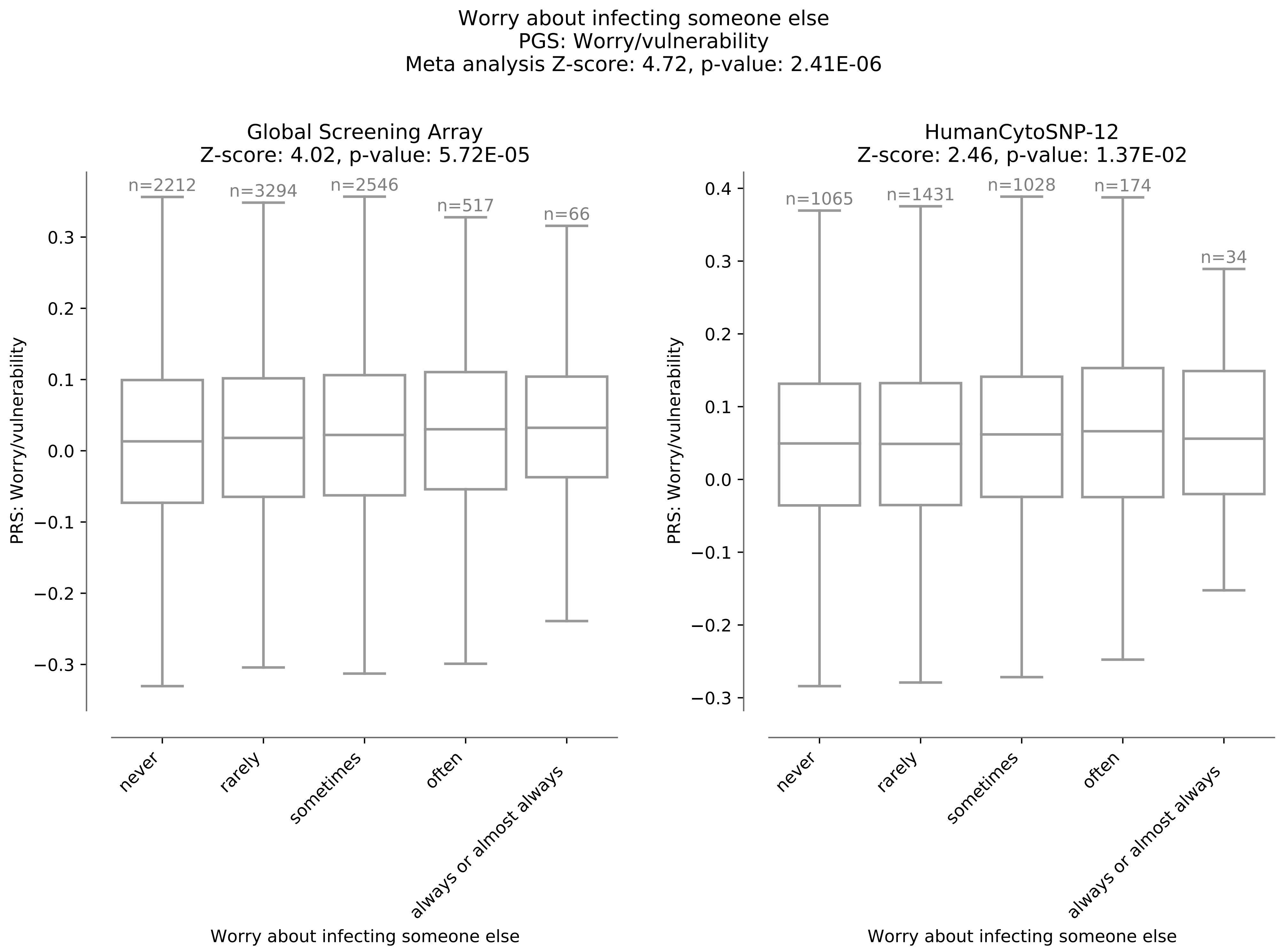
Worry about infecting someone else
PGS: Worry/vulnerability
Meta analysis Z-score: 4.72, p-value: 2.41E-06
Global Screening Array
Z-score: 4.02, p-value: 5.72E-05
HumanCytoSNP-12
Z-score: 2.46, p-value: 1.37E-02
PRS: Worry/vulnerability
n=2212
n=3294
n=2546
n=517
n=66
n=1065
n=1431
n=1028
n=174
n=34
never
rarely
sometimes
often
always or almost always
Worry about infecting someone else

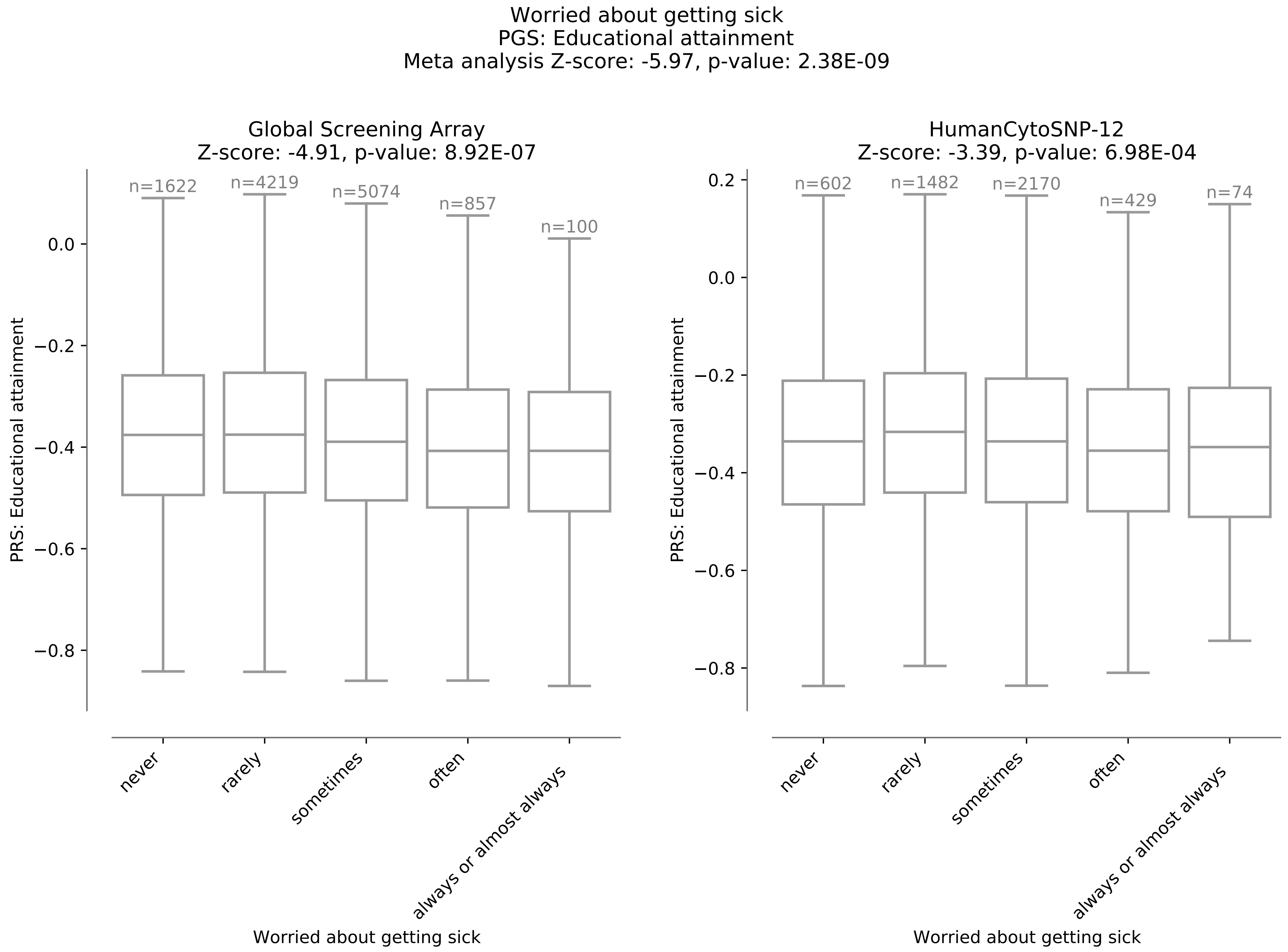
Worried about getting sick
PGS: Educational attainment
Meta analysis Z-score: -5.97, p-value: 2.38E-09
Global Screening Array
Z-score: -4.91, p-value: 8.92E-07
n=1622
n=4219
n=5074
n=857
n=100
PRS: Educational attainment
0.0
−0.2
−0.4
−0.6
−0.8
never
rarely
sometimes
often
always or almost always
Worried about getting sick
HumanCytoSNP-12
Z-score: -3.39, p-value: 6.98E-04
n=602
n=1482
n=2170
n=429
n=74
PRS: Educational attainment
0.2
0.0
−0.2
−0.4
−0.6
−0.8
never
rarely
sometimes
often
always or almost always
Worried about getting sick

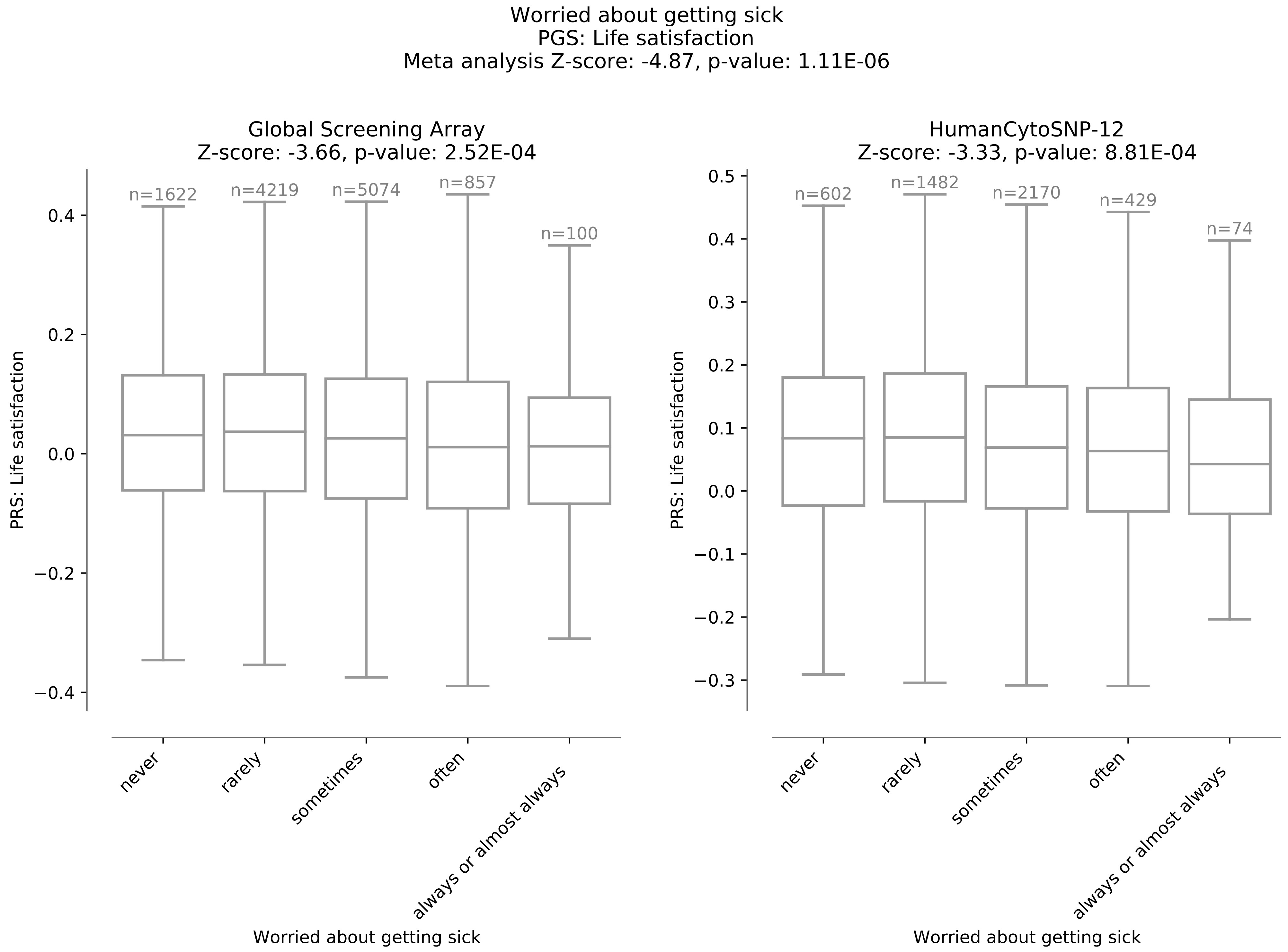

Worried about getting sick
PGS: Life satisfaction
Meta analysis Z-score: -4.87, p-value: 1.11E-06
Global Screening Array
Z-score: -3.66, p-value: 2.52E-04
n=1622
n=4219
n=5074
n=857
n=100
PRS: Life satisfaction
0.4
0.2
0.0
−0.2
−0.4
never
rarely
sometimes
often
always or almost always
Worried about getting sick
HumanCytoSNP-12
Z-score: -3.33, p-value: 8.81E-04
n=602
n=1482
n=2170
n=429
n=74
PRS: Life satisfaction
0.5
0.4
0.3
0.2
0.1
0.0
−0.1
−0.2
−0.3
never
rarely
sometimes
often
always or almost always
Worried about getting sick

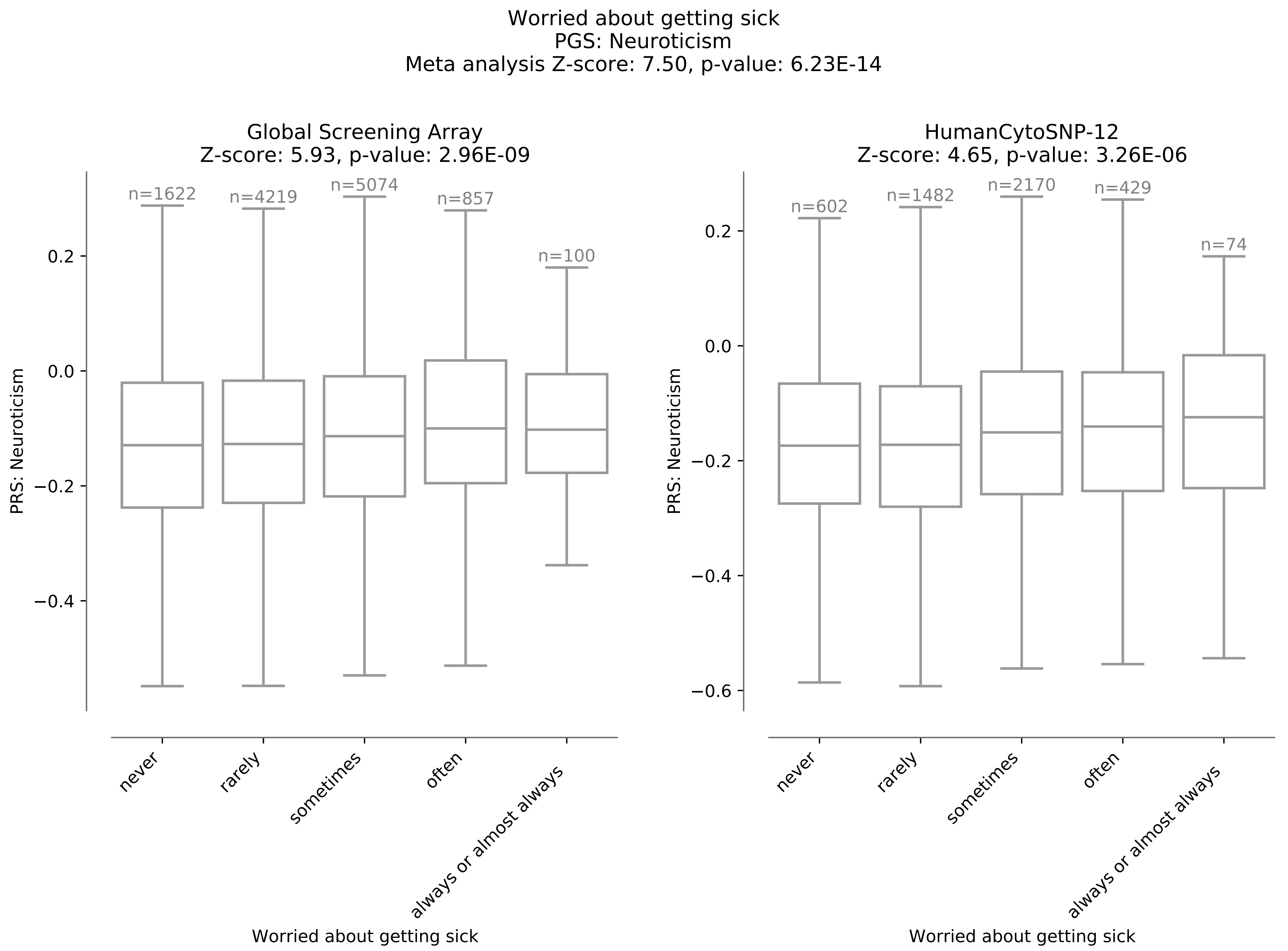

Worried about getting sick
PGS: Neuroticism
Meta analysis Z-score: 7.50, p-value: 6.23E-14
Global Screening Array
Z-score: 5.93, p-value: 2.96E-09
n=1622
n=4219
n=5074
n=857
n=100
0.2
0.0
−0.2
−0.4
PRS: Neuroticism
never
rarely
sometimes
often
always or almost always
Worried about getting sick
HumanCytoSNP-12
Z-score: 4.65, p-value: 3.26E-06
n=602
n=1482
n=2170
n=429
n=74
0.2
0.0
−0.2
−0.4
−0.6
PRS: Neuroticism
never
rarely
sometimes
often
always or almost always
Worried about getting sick

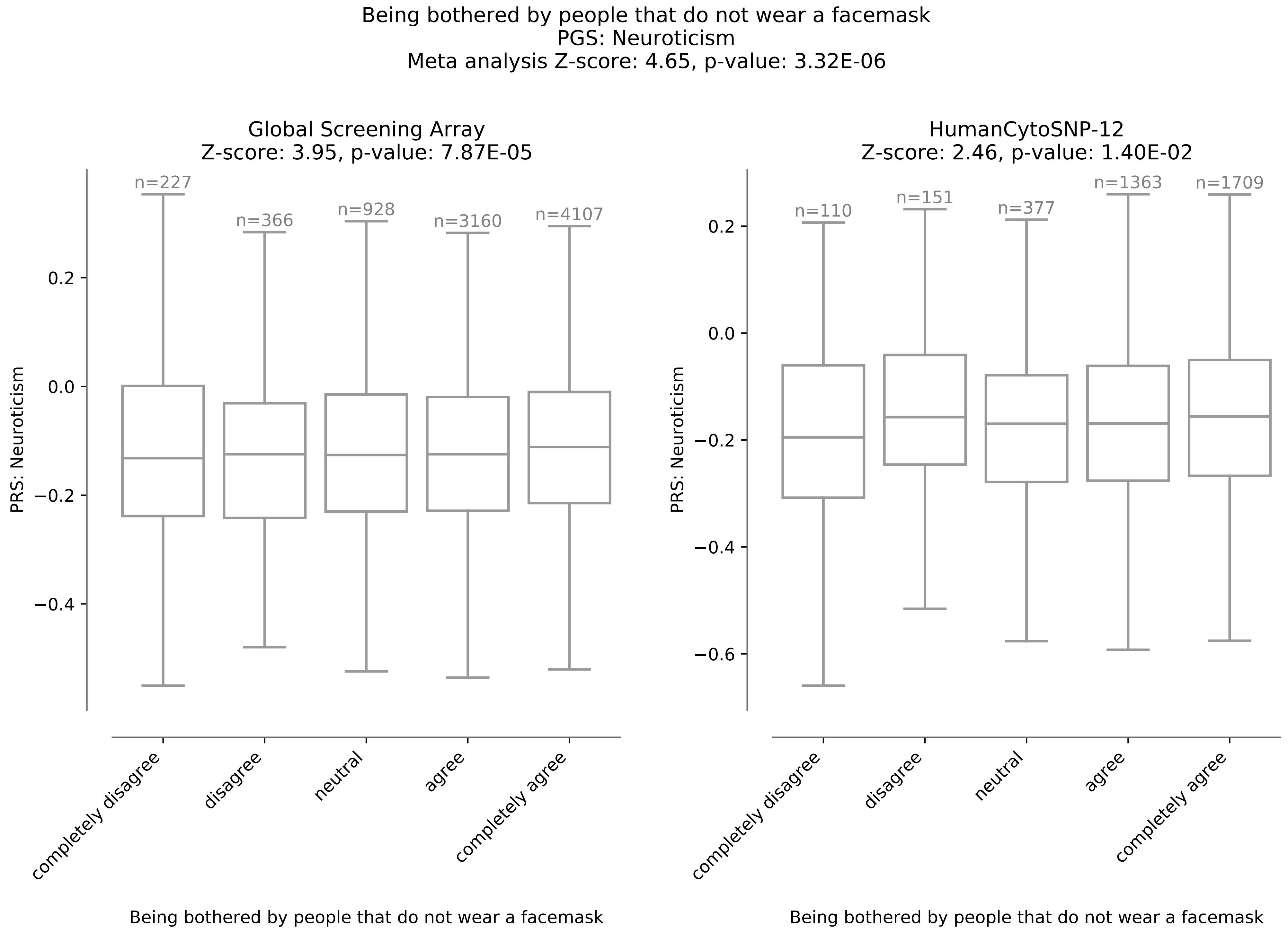
Being bothered by people that do not wear a facemask
PGS: Neuroticism
Meta analysis Z-score: 4.65, p-value: 3.32E-06
Global Screening Array
Z-score: 3.95, p-value: 7.87E-05
HumanCytoSNP-12
Z-score: 2.46, p-value: 1.40E-02
PRS: Neuroticism
n=227
n=366
n=928
n=3160
n=4107
n=110
n=151
n=377
n=1363
n=1709
0.2
0.0
−0.2
−0.4
−0.6
completely disagree
disagree
neutral
agree
completely agree
Being bothered by people that do not wear a facemask

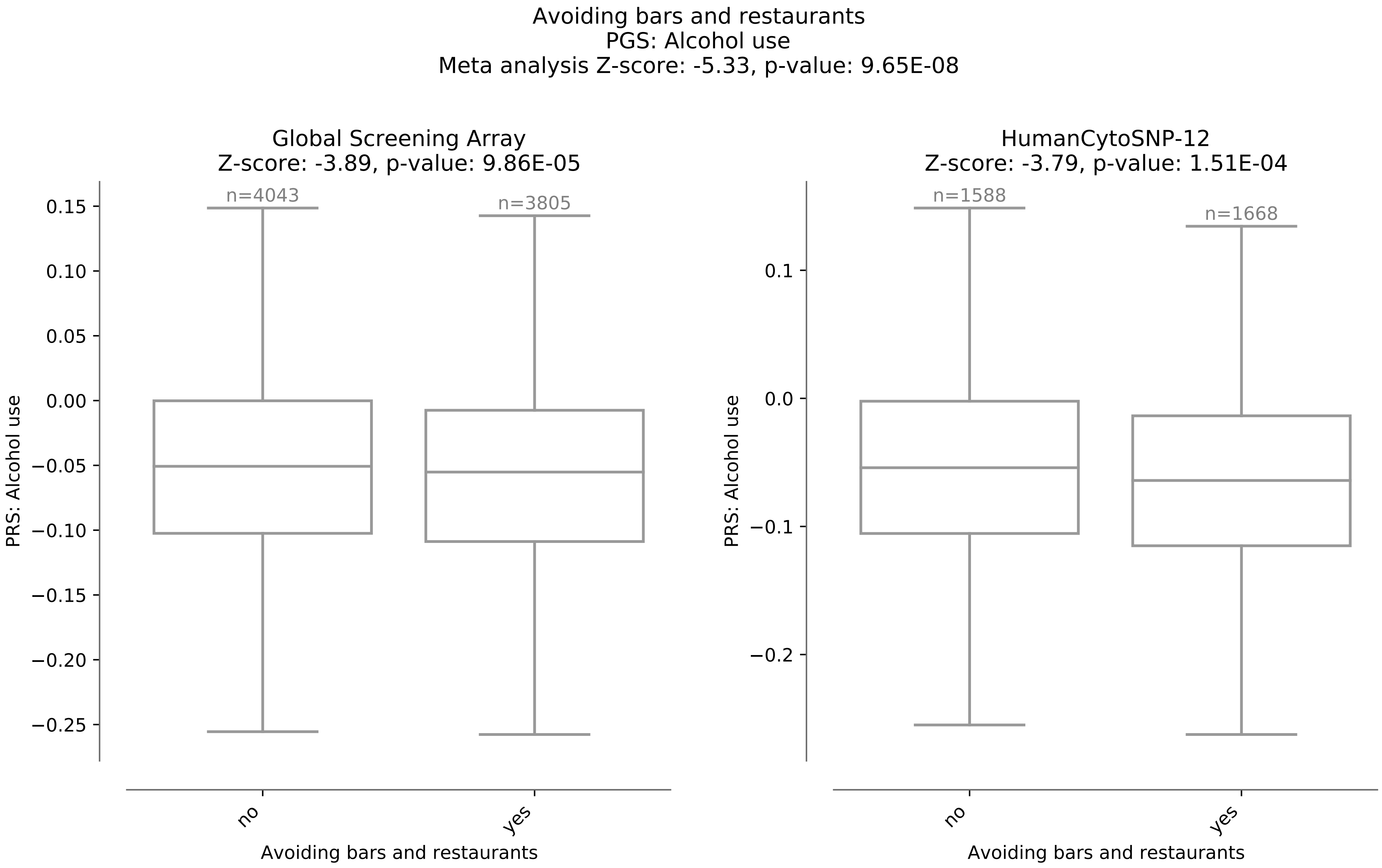
Avoiding bars and restaurants
PGS: Alcohol use
Meta analysis Z-score: -5.33, p-value: 9.65E-08
Global Screening Array
Z-score: -3.89, p-value: 9.86E-05
n=4043
n=3805
0.15
0.10
0.05
0.00
−0.05
−0.10
−0.15
−0.20
−0.25
PRS: Alcohol use
no
yes
Avoiding bars and restaurants
HumanCytoSNP-12
Z-score: -3.79, p-value: 1.51E-04
n=1588
n=1668
0.1
0.0
−0.1
−0.2
PRS: Alcohol use
no
yes
Avoiding bars and restaurants

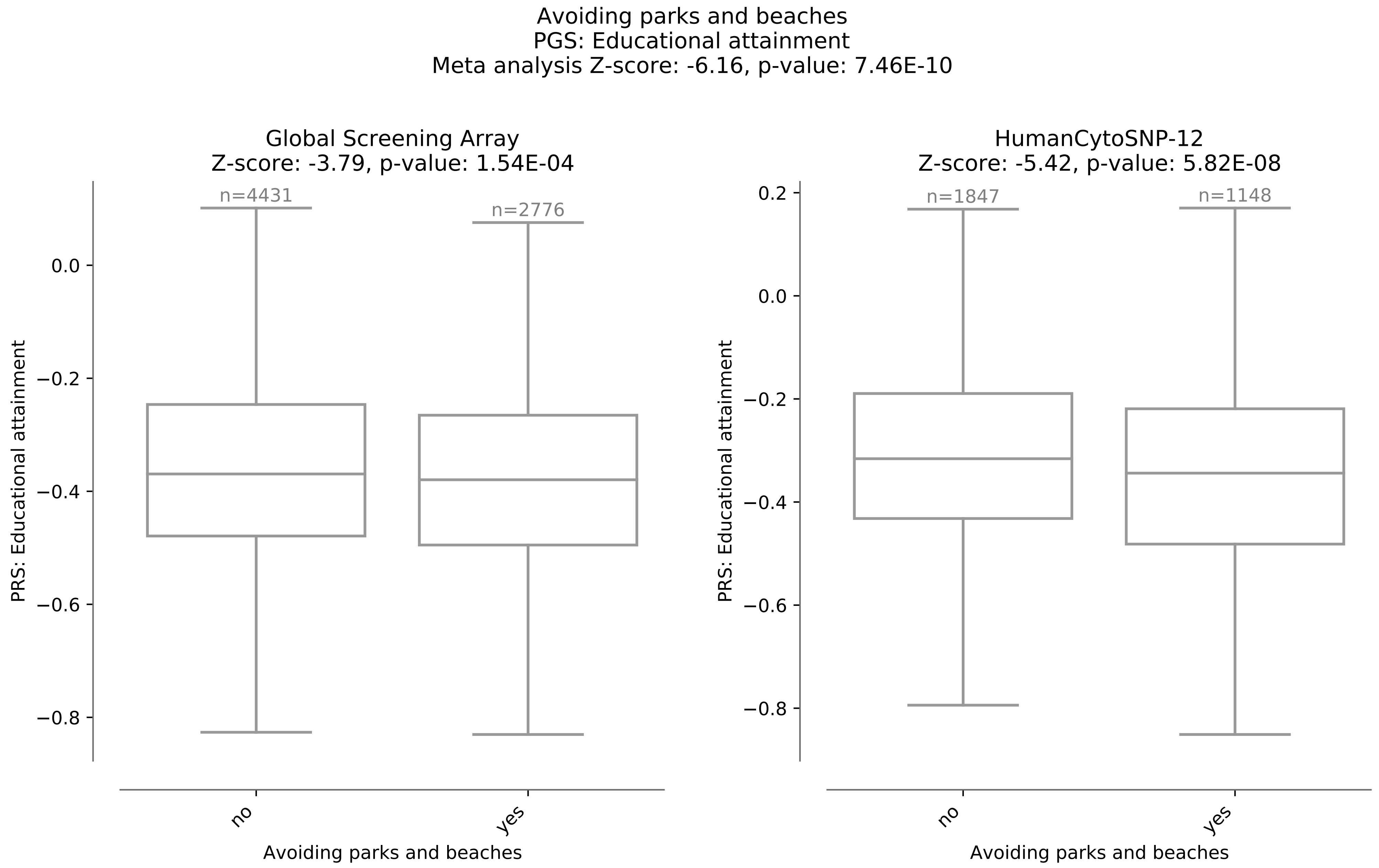

Avoiding parks and beaches
PGS: Educational attainment
Meta analysis Z-score: -6.16, p-value: 7.46E-10
Global Screening Array
Z-score: -3.79, p-value: 1.54E-04
n=4431
n=2776
0.0
−0.2
−0.4
−0.6
−0.8
PRS: Educational attainment
no
yes
Avoiding parks and beaches
HumanCytoSNP-12
Z-score: -5.42, p-value: 5.82E-08
n=1847
n=1148
0.2
0.0
−0.2
−0.4
−0.6
−0.8
PRS: Educational attainment
no
yes
Avoiding parks and beaches

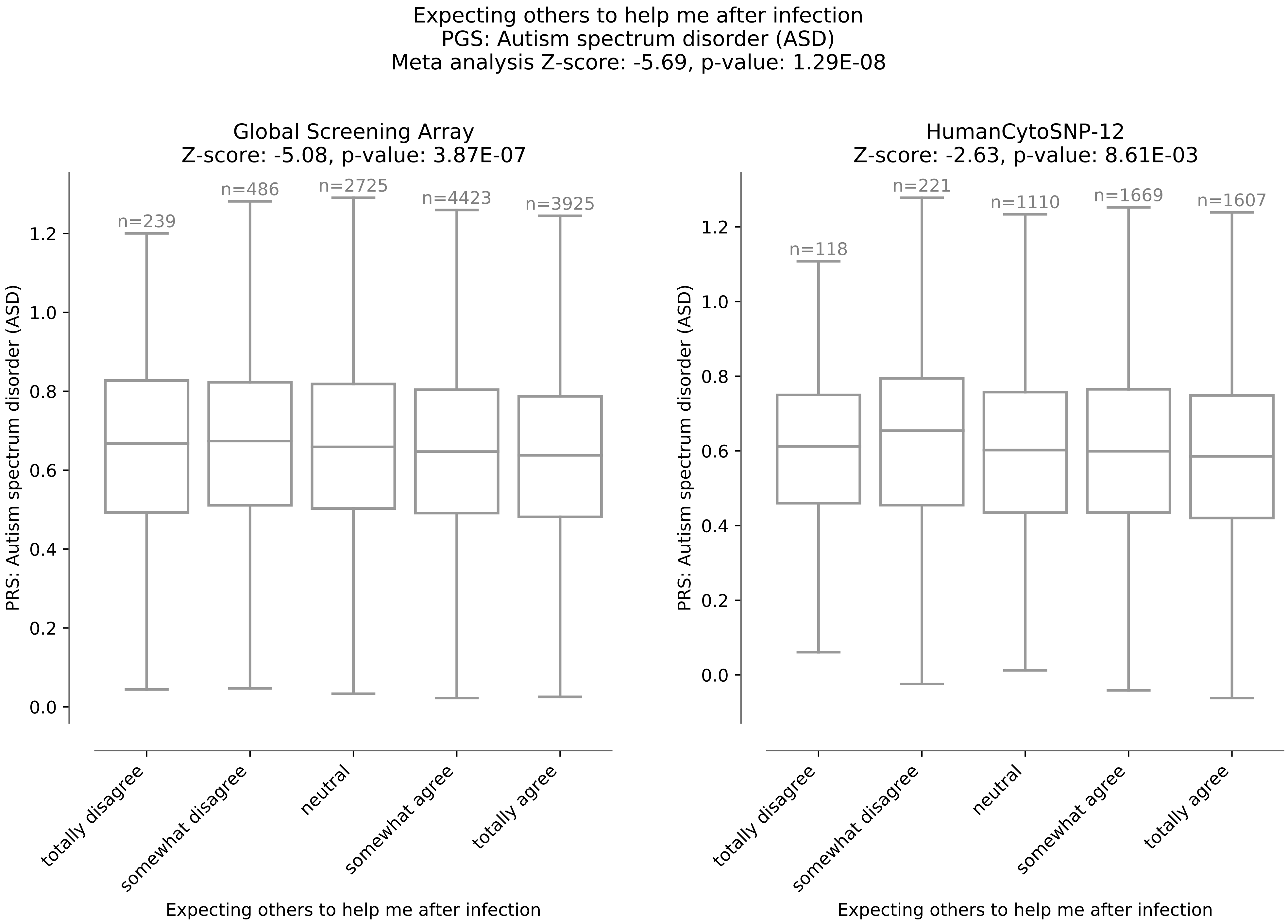

Expecting others to help me after infection
PGS: Autism spectrum disorder (ASD)
Meta analysis Z-score: -5.69, p-value: 1.29E-08
Global Screening Array
Z-score: -5.08, p-value: 3.87E-07
HumanCytoSNP-12
Z-score: -2.63, p-value: 8.61E-03
PRS: Autism spectrum disorder (ASD)
n=239
n=486
n=2725
n=4423
n=3925
n=118
n=221
n=1110
n=1669
n=1607
0.0
0.2
0.4
0.6
0.8
1.0
1.2
totally disagree
somewhat disagree
neutral
somewhat agree
totally agree
Expecting others to help me after infection

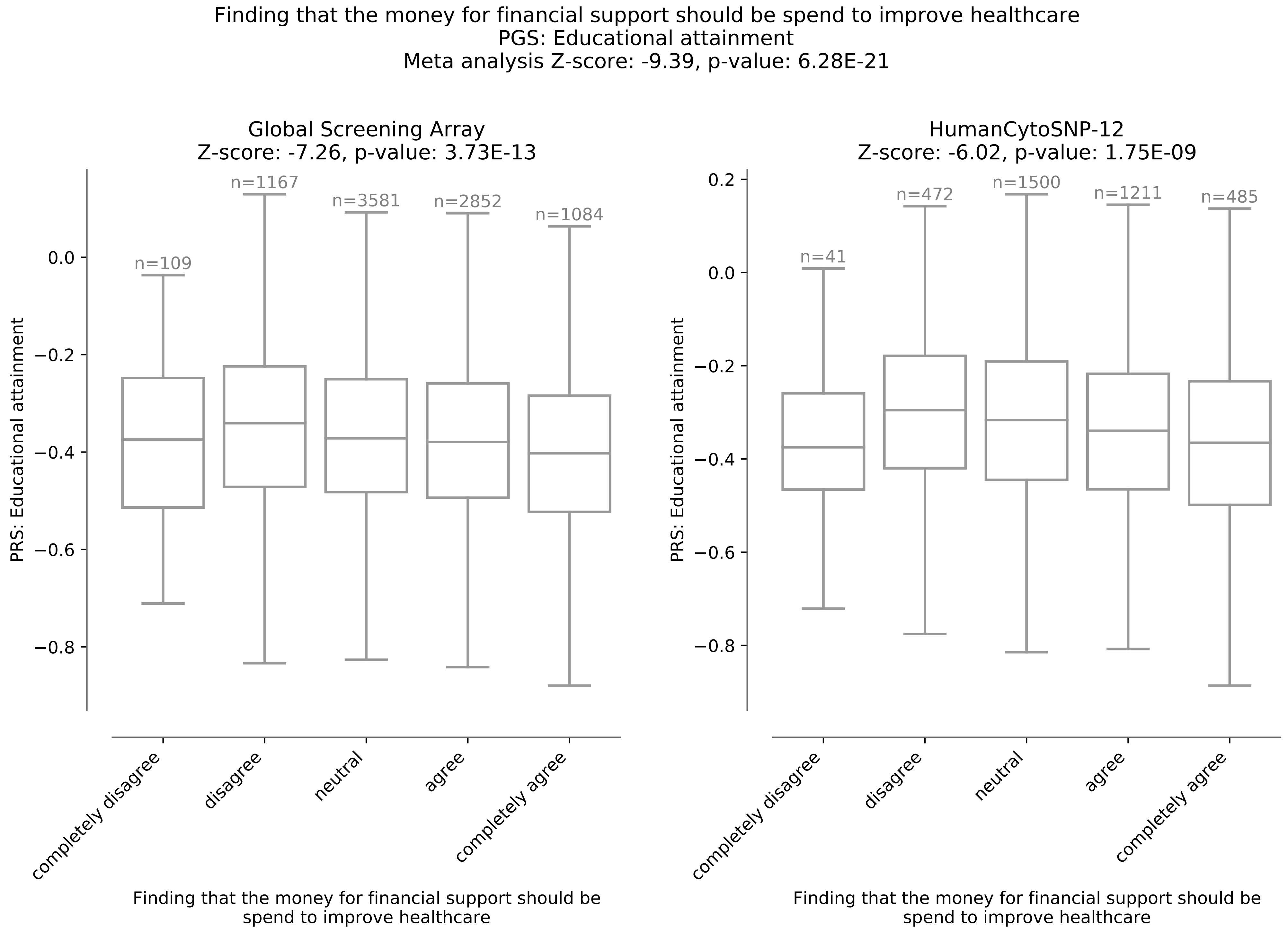
Finding that the money for financial support should be spend to improve healthcare
PGS: Educational attainment
Meta analysis Z-score: -9.39, p-value: 6.28E-21
Global Screening Array
Z-score: -7.26, p-value: 3.73E-13
n=109
n=1167
n=3581
n=2852
n=1084
PRS: Educational attainment
completely disagree
disagree
neutral
agree
completely agree
Finding that the money for financial support should be spend to improve healthcare
HumanCytoSNP-12
Z-score: -6.02, p-value: 1.75E-09
n=41
n=472
n=1500
n=1211
n=485
PRS: Educational attainment
completely disagree
disagree
neutral
agree
completely agree
Finding that the money for financial support should be spend to improve healthcare

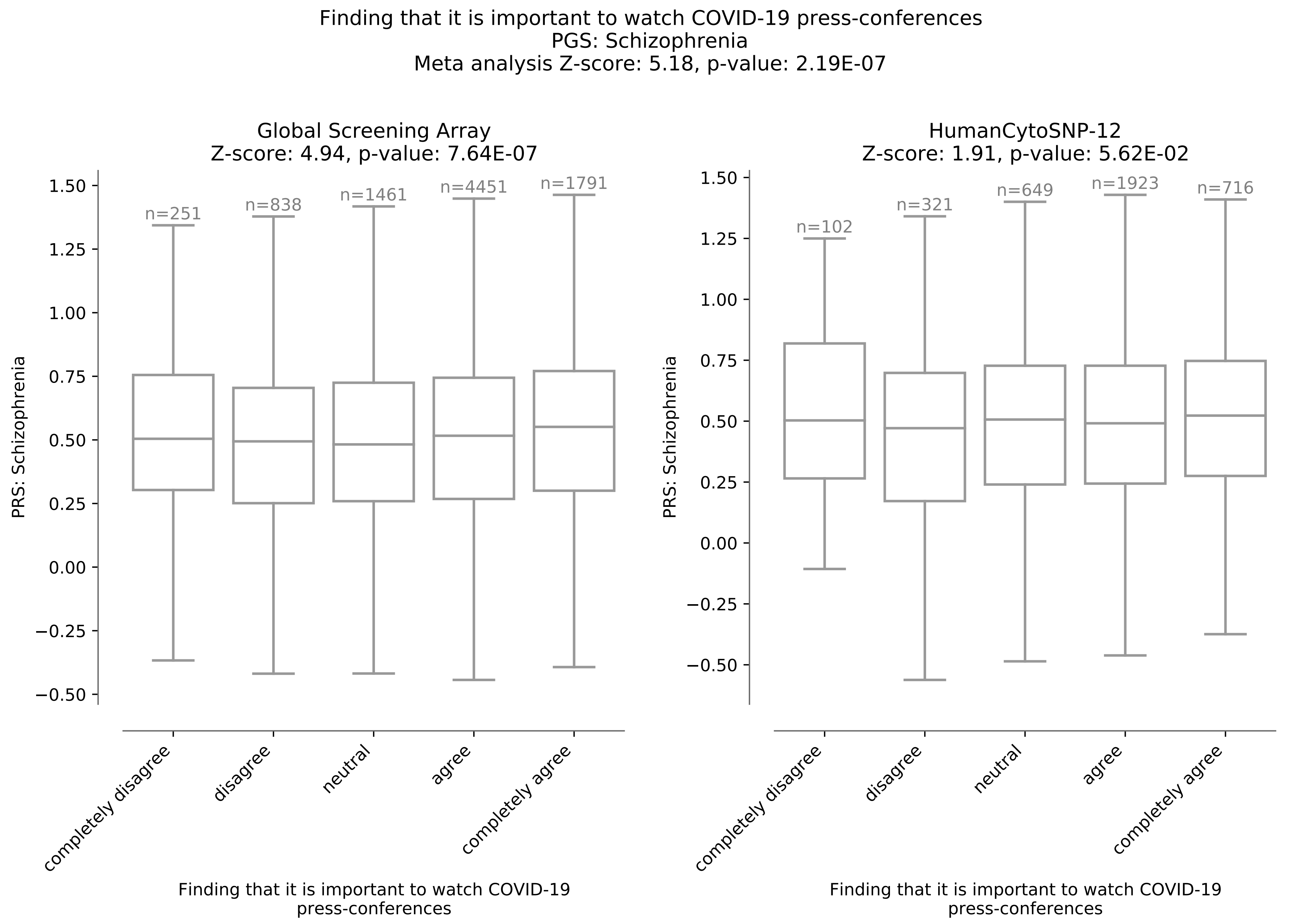
Finding that it is important to watch COVID-19 press-conferences
PGS: Schizophrenia
Meta analysis Z-score: 5.18, p-value: 2.19E-07
Global Screening Array
Z-score: 4.94, p-value: 7.64E-07
HumanCytoSNP-12
Z-score: 1.91, p-value: 5.62E-02
PRS: Schizophrenia
n=251
n=838
n=1461
n=4451
n=1791
n=102
n=321
n=649
n=1923
n=716
completely disagree
disagree
neutral
agree
completely agree
Finding that it is important to watch COVID-19 press-conferences

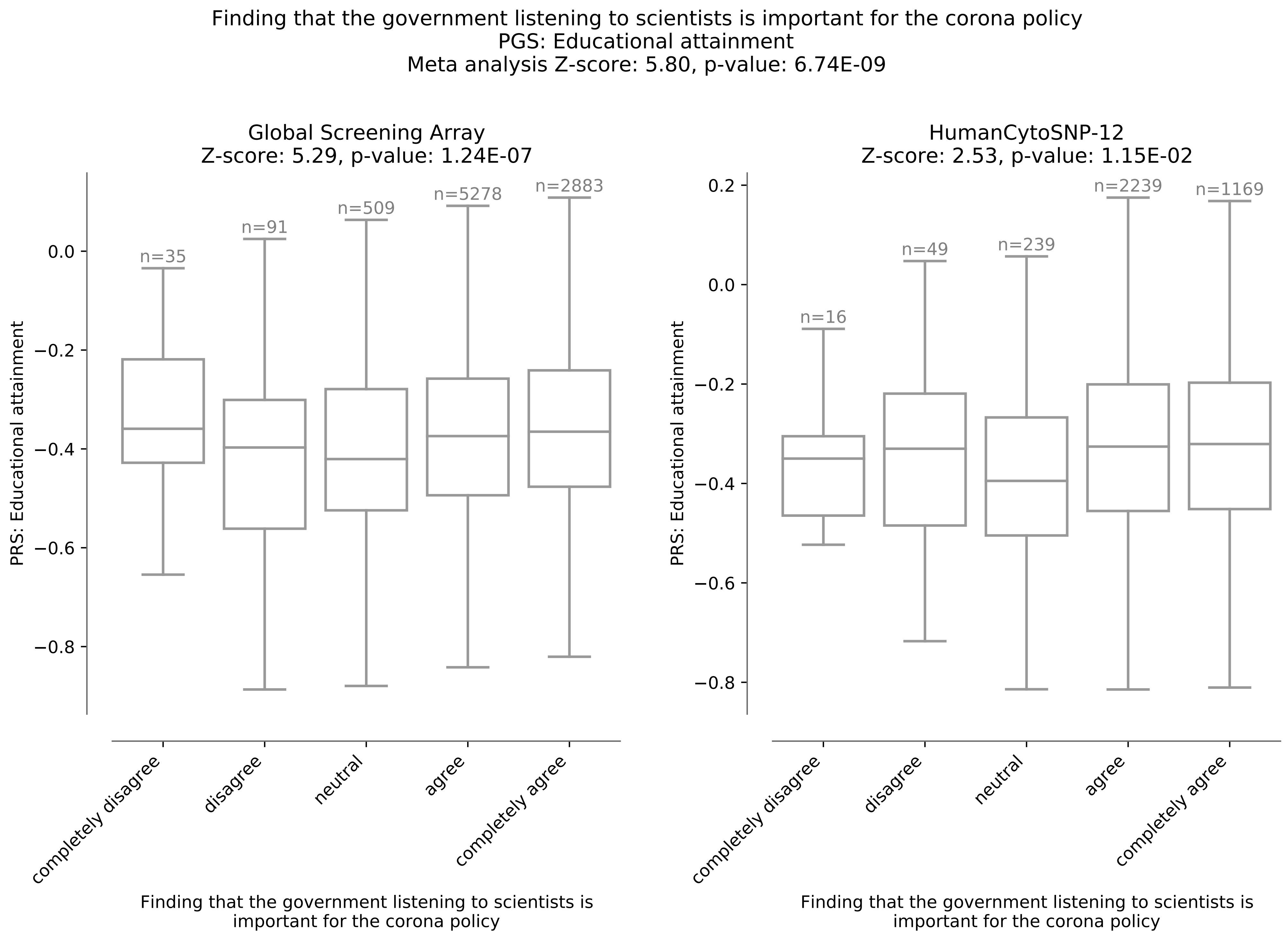
Finding that the government listening to scientists is important for the corona policy
PGS: Educational attainment
Meta analysis Z-score: 5.80, p-value: 6.74E-09
Global Screening Array
Z-score: 5.29, p-value: 1.24E-07
HumanCytoSNP-12
Z-score: 2.53, p-value: 1.15E-02
PRS: Educational attainment
n=35
n=91
n=509
n=5278
n=2883
n=16
n=49
n=239
n=2239
n=1169
0.2
0.0
−0.2
−0.4
−0.6
−0.8
completely disagree
disagree
neutral
agree
completely agree
Finding that the government listening to scientists is important for the corona policy

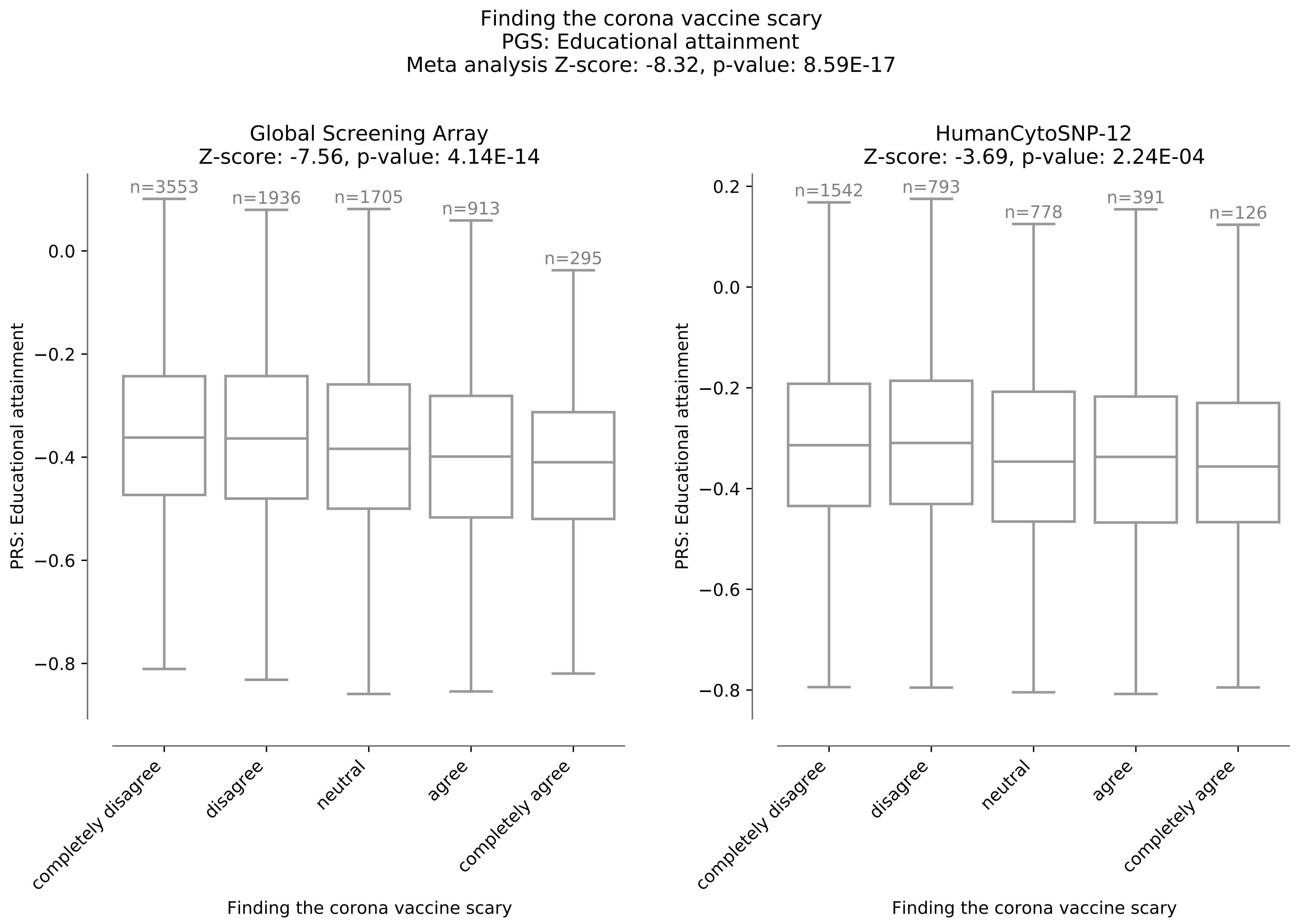
Finding the corona vaccine scary
PGS: Educational attainment
Meta analysis Z-score: -8.32, p-value: 8.59E-17
Global Screening Array
Z-score: -7.56, p-value: 4.14E-14
n=3553
n=1936
n=1705
n=913
n=295
0.0
−0.2
−0.4
−0.6
−0.8
PRS: Educational attainment
completely disagree
disagree
neutral
agree
completely agree
Finding the corona vaccine scary
HumanCytoSNP-12
Z-score: -3.69, p-value: 2.24E-04
n=1542
n=793
n=778
n=391
n=126
0.2
0.0
−0.2
−0.4
−0.6
−0.8
PRS: Educational attainment
completely disagree
disagree
neutral
agree
completely agree
Finding the corona vaccine scary

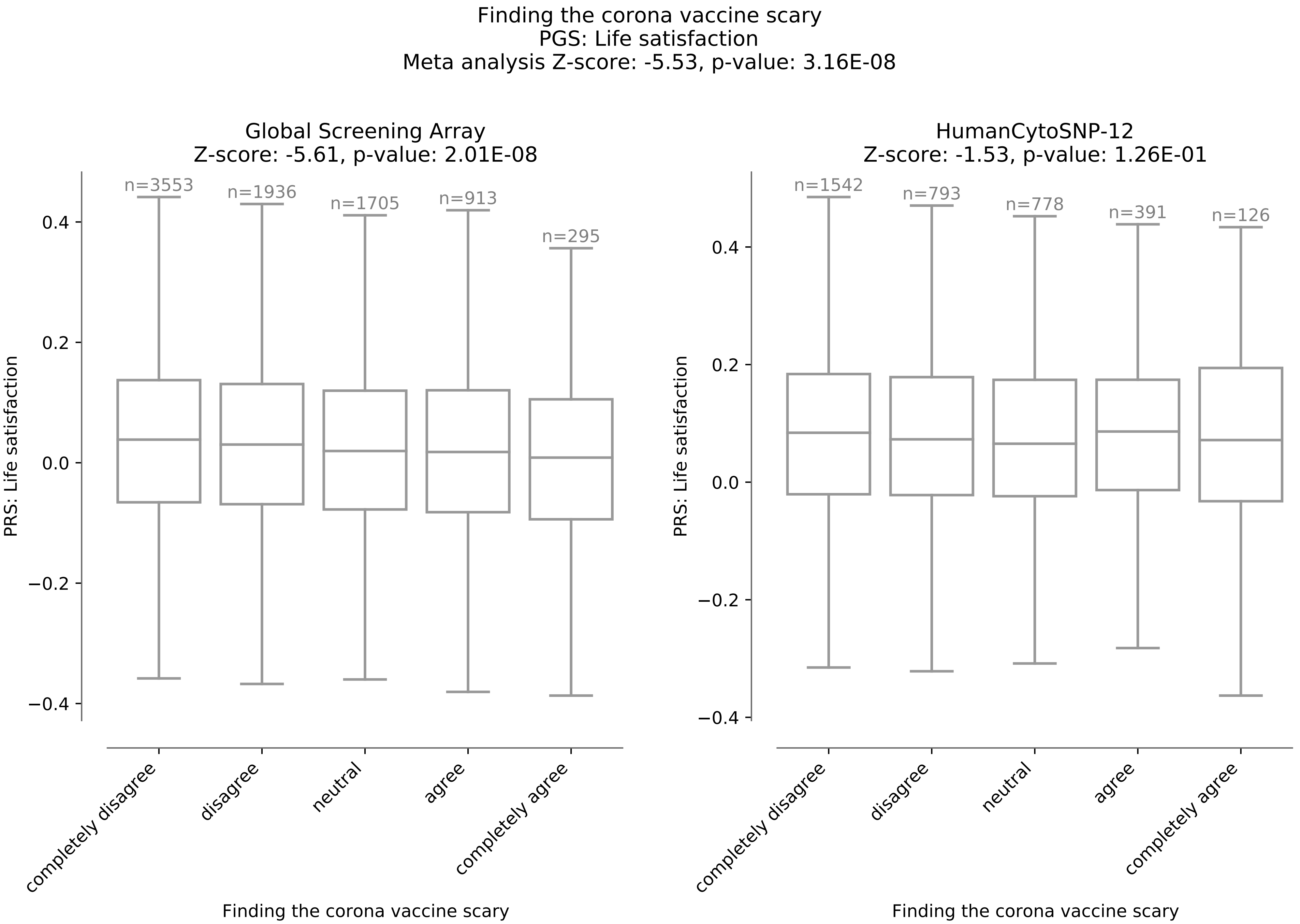
Finding the corona vaccine scary
PGS: Life satisfaction
Meta analysis Z-score: -5.53, p-value: 3.16E-08
Global Screening Array
Z-score: -5.61, p-value: 2.01E-08
n=3553
n=1936
n=1705
n=913
n=295
0.4
0.2
0.0
−0.2
−0.4
PRS: Life satisfaction
completely disagree
disagree
neutral
agree
completely agree
Finding the corona vaccine scary
HumanCytoSNP-12
Z-score: -1.53, p-value: 1.26E-01
n=1542
n=793
n=778
n=391
n=126
0.4
0.2
0.0
−0.2
−0.4
PRS: Life satisfaction
completely disagree
disagree
neutral
agree
completely agree
Finding the corona vaccine scary

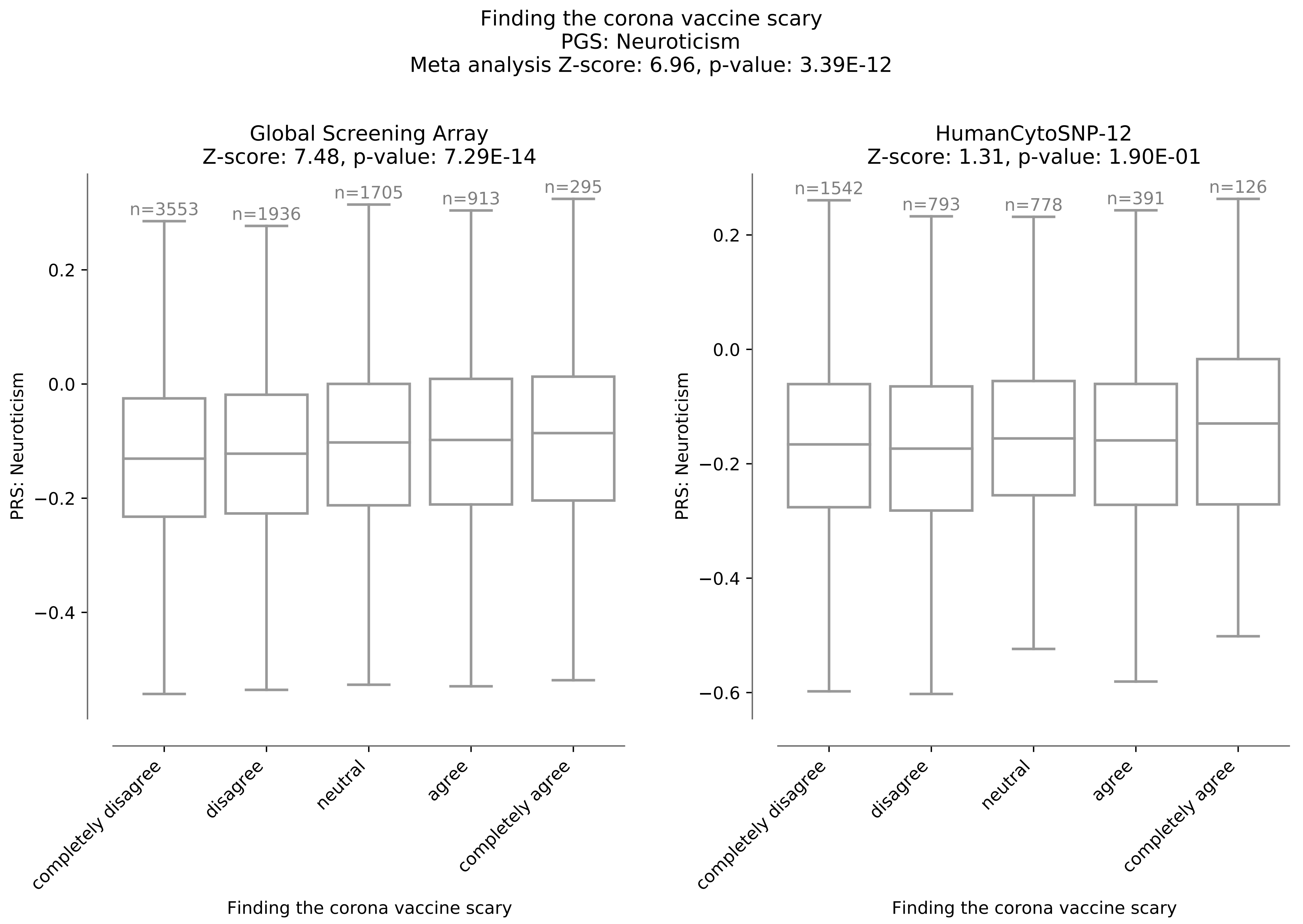
Finding the corona vaccine scary
PGS: Neuroticism
Meta analysis Z-score: 6.96, p-value: 3.39E-12
Global Screening Array
Z-score: 7.48, p-value: 7.29E-14
HumanCytoSNP-12
Z-score: 1.31, p-value: 1.90E-01
PRS: Neuroticism
n=3553
n=1936
n=1705
n=913
n=295
n=1542
n=793
n=778
n=391
n=126
0.2
0.0
−0.2
−0.4
−0.6
completely disagree
disagree
neutral
agree
completely agree
Finding the corona vaccine scary

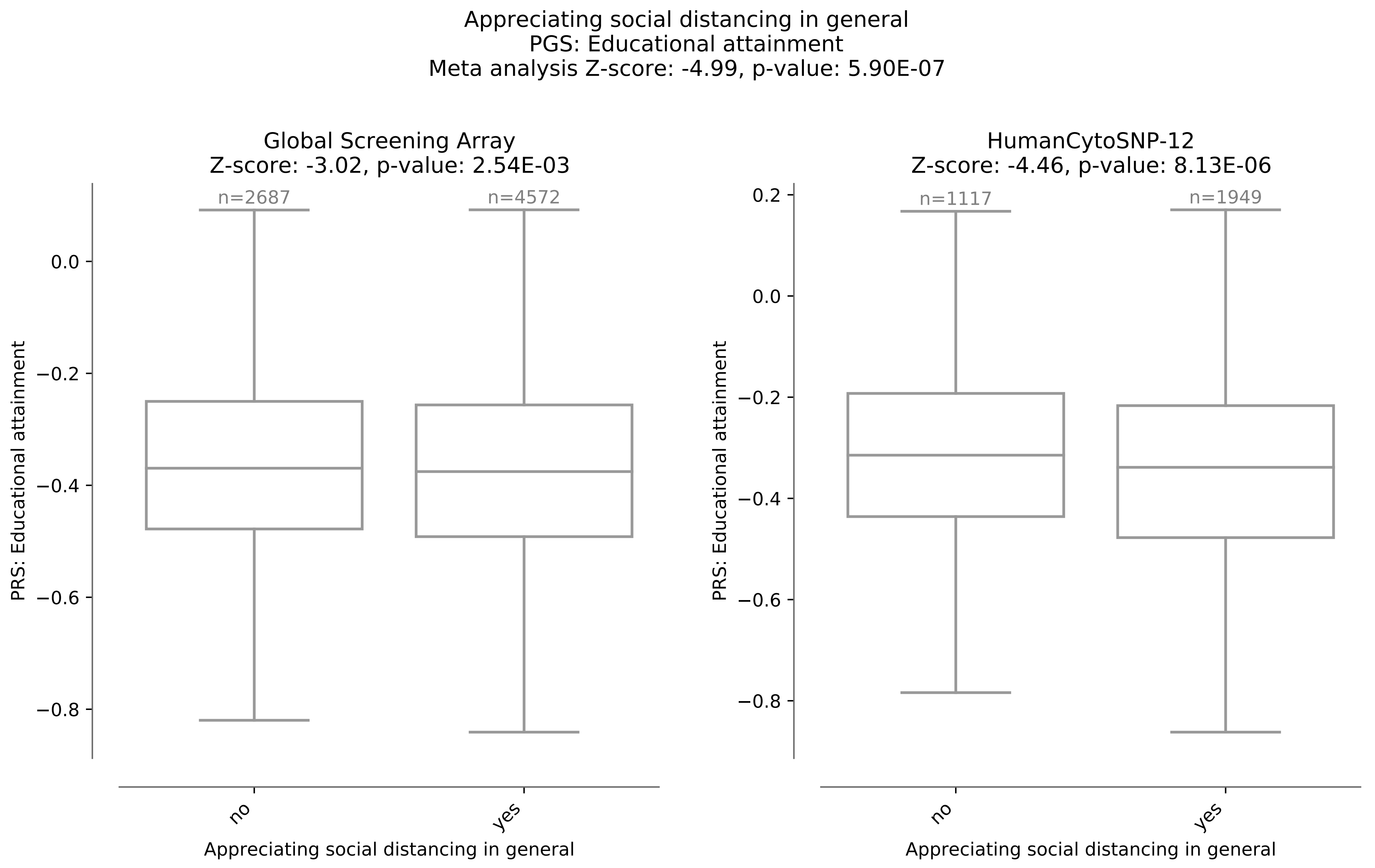
Appreciating social distancing in general
PGS: Educational attainment
Meta analysis Z-score: -4.99, p-value: 5.90E-07
Global Screening Array
Z-score: -3.02, p-value: 2.54E-03
n=2687
n=4572
PRS: Educational attainment
0.0
−0.2
−0.4
−0.6
−0.8
no
yes
Appreciating social distancing in general
HumanCytoSNP-12
Z-score: -4.46, p-value: 8.13E-06
n=1117
n=1949
PRS: Educational attainment
0.2
0.0
−0.2
−0.4
−0.6
−0.8
no
yes
Appreciating social distancing in general

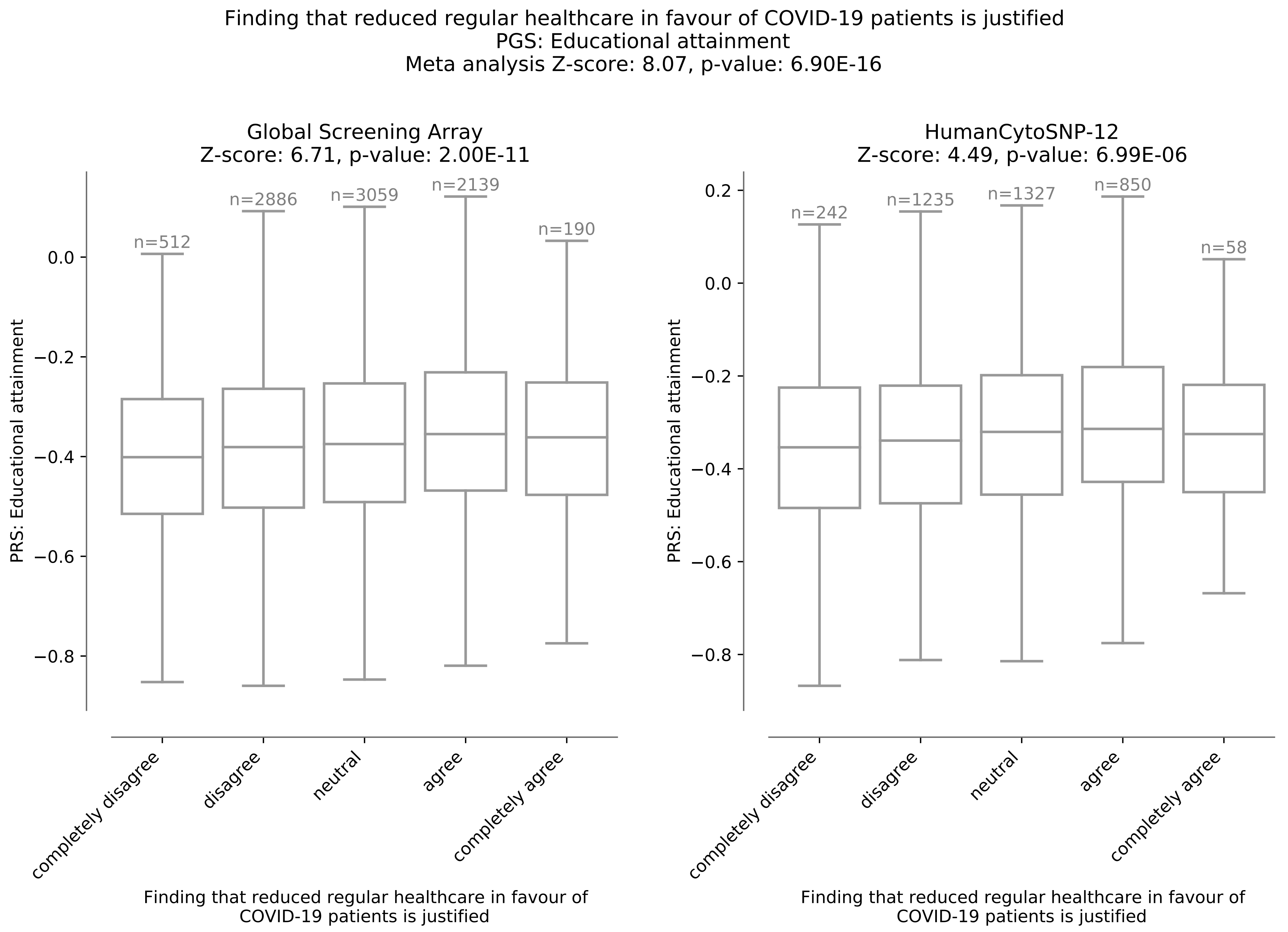

Finding that reduced regular healthcare in favour of COVID-19 patients is justified
PGS: Educational attainment
Meta analysis Z-score: 8.07, p-value: 6.90E-16
Global Screening Array
Z-score: 6.71, p-value: 2.00E-11
HumanCytoSNP-12
Z-score: 4.49, p-value: 6.99E-06
PRS: Educational attainment
n=512
n=2886
n=3059
n=2139
n=190
n=242
n=1235
n=1327
n=850
n=58
0.2
0.0
−0.2
−0.4
−0.6
−0.8
completely disagree
disagree
neutral
agree
completely agree
Finding that reduced regular healthcare in favour of COVID-19 patients is justified

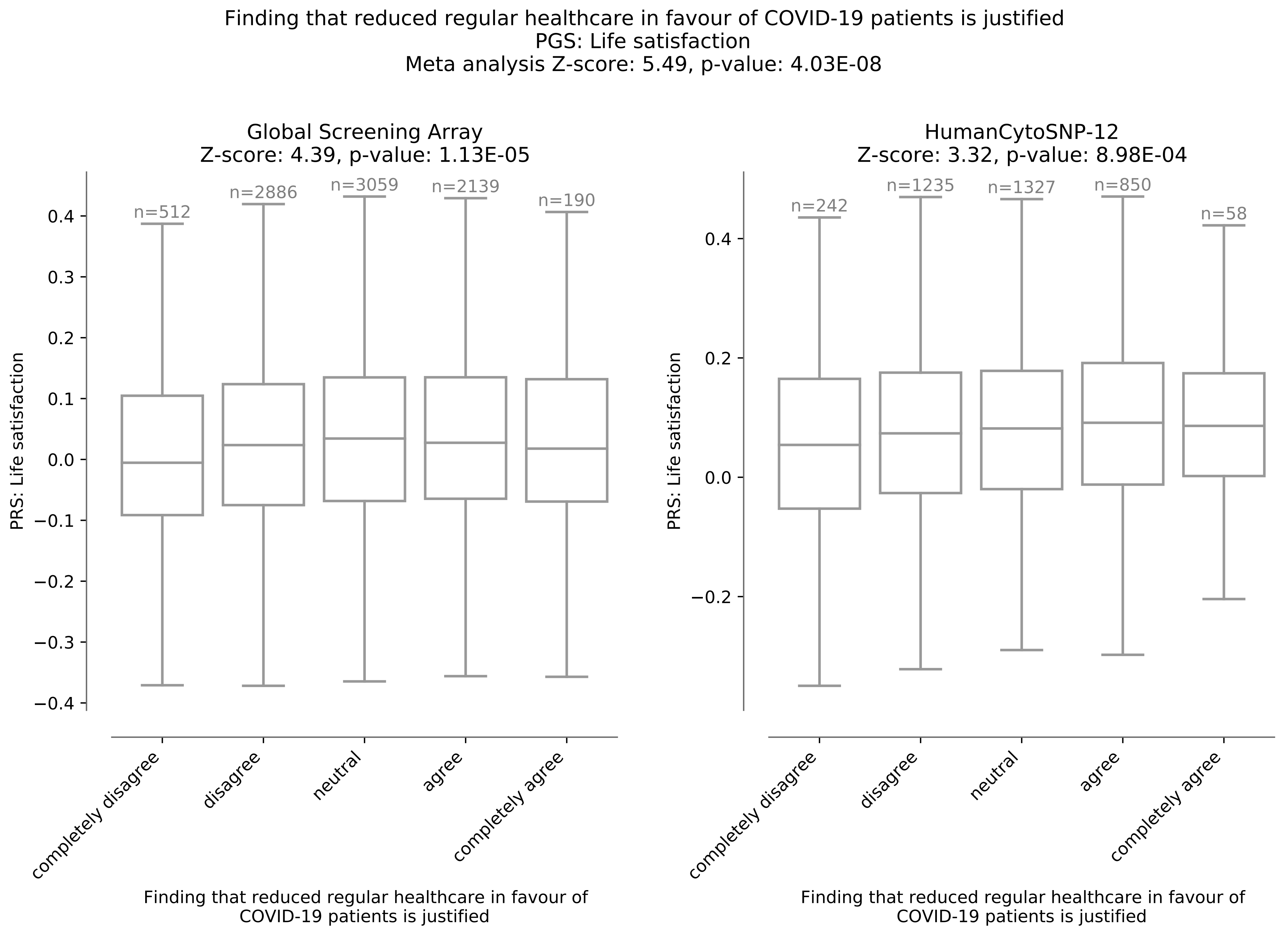
Finding that reduced regular healthcare in favour of COVID-19 patients is justified
PGS: Life satisfaction
Meta analysis Z-score: 5.49, p-value: 4.03E-08
Global Screening Array
Z-score: 4.39, p-value: 1.13E-05
n=512
n=2886
n=3059
n=2139
n=190
PRS: Life satisfaction
0.4
0.3
0.2
0.1
0.0
−0.1
−0.2
−0.3
−0.4
completely disagree
disagree
neutral
agree
completely agree
Finding that reduced regular healthcare in favour of COVID-19 patients is justified
HumanCytoSNP-12
Z-score: 3.32, p-value: 8.98E-04
n=242
n=1235
n=1327
n=850
n=58
PRS: Life satisfaction
0.4
0.2
0.0
−0.2
completely disagree
disagree
neutral
agree
completely agree
Finding that reduced regular healthcare in favour of COVID-19 patients is justified

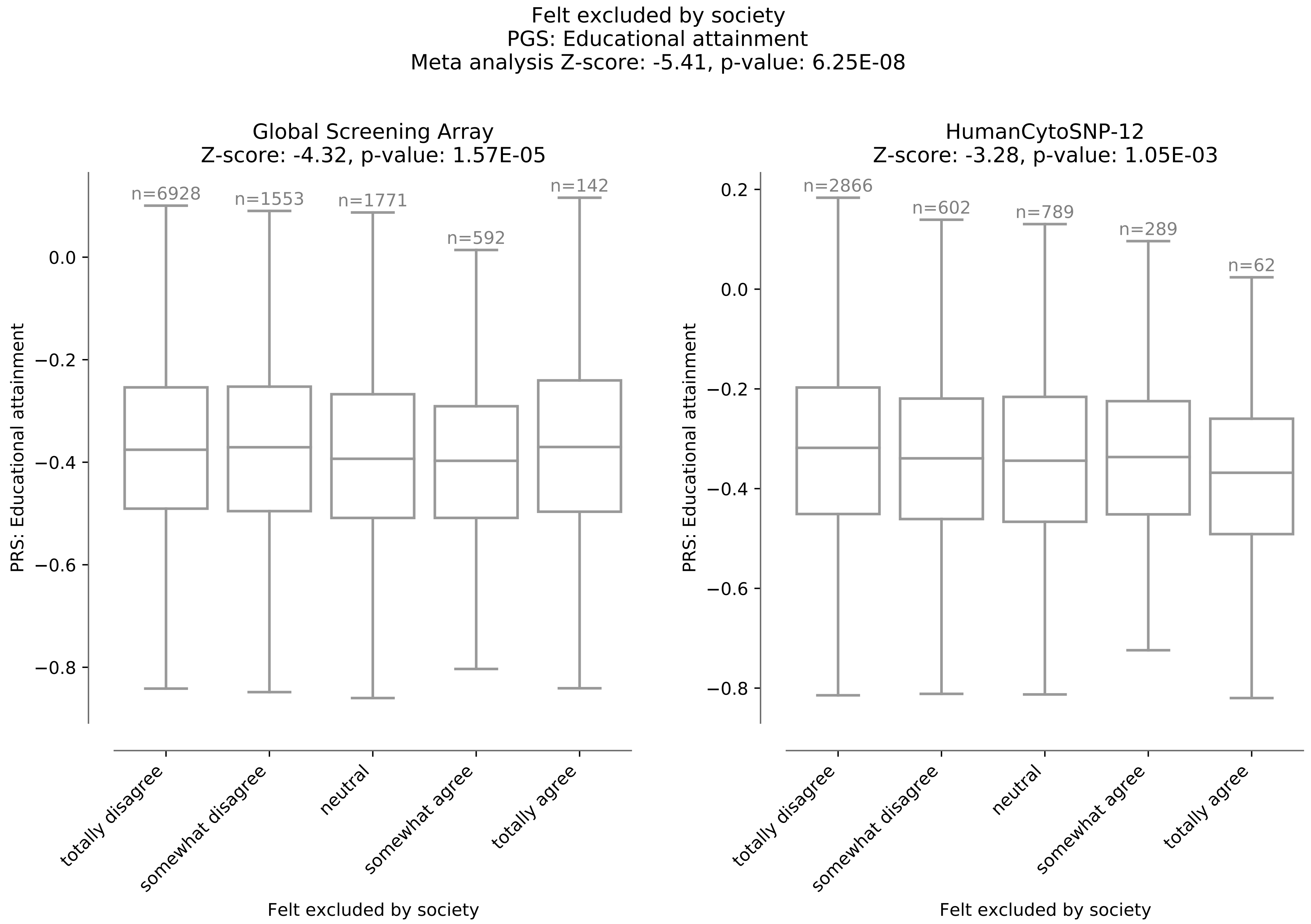

Felt excluded by society
PGS: Educational attainment
Meta analysis Z-score: -5.41, p-value: 6.25E-08
Global Screening Array
Z-score: -4.32, p-value: 1.57E-05
n=6928
n=1553
n=1771
n=592
n=142
0.0
−0.2
−0.4
−0.6
−0.8
PRS: Educational attainment
totally disagree
somewhat disagree
neutral
somewhat agree
totally agree
Felt excluded by society
HumanCytoSNP-12
Z-score: -3.28, p-value: 1.05E-03
n=2866
n=602
n=789
n=289
n=62
0.2
0.0
−0.2
−0.4
−0.6
−0.8
PRS: Educational attainment
totally disagree
somewhat disagree
neutral
somewhat agree
totally agree
Felt excluded by society

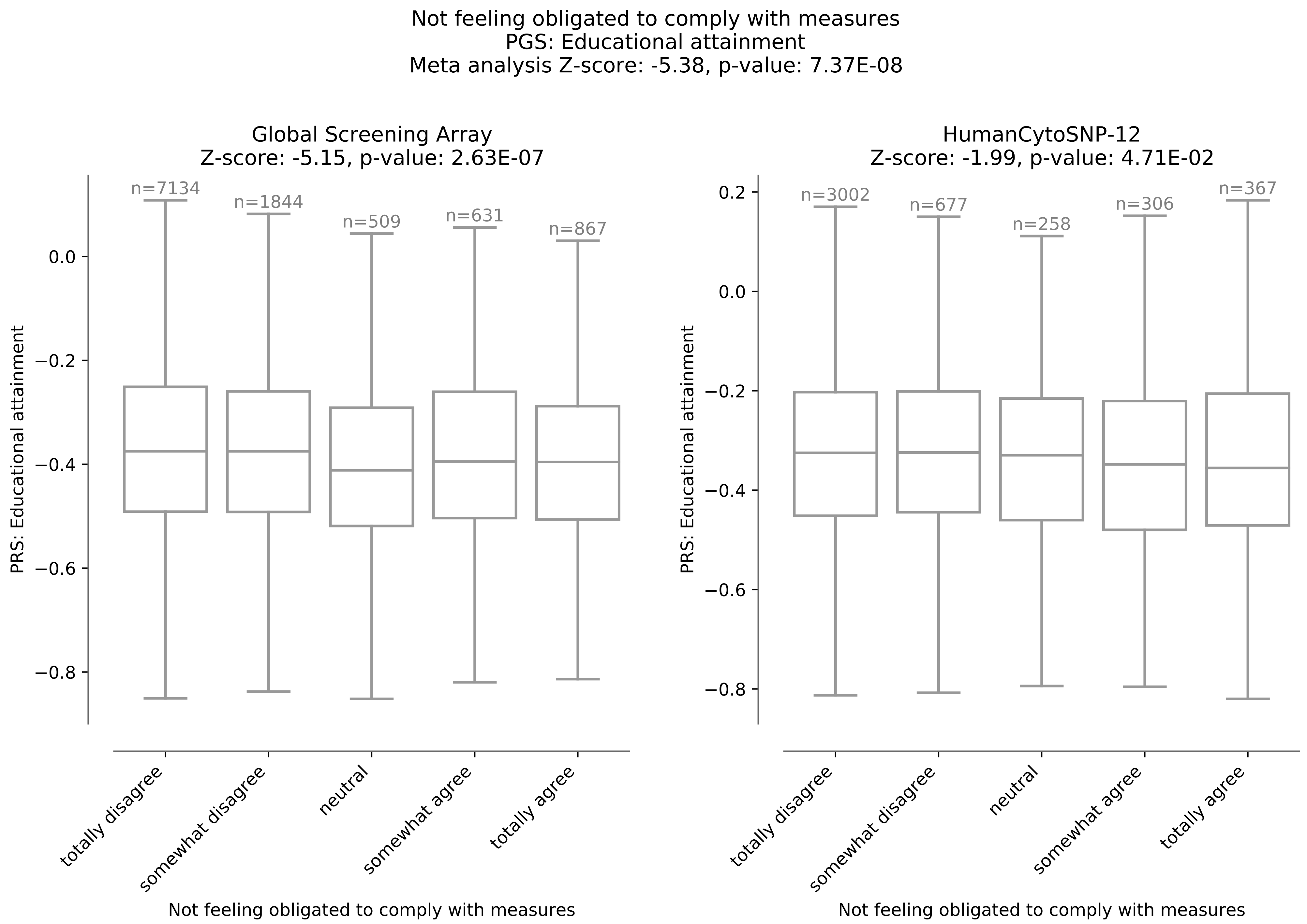
Not feeling obligated to comply with measures
PGS: Educational attainment
Meta analysis Z-score: -5.38, p-value: 7.37E-08
Global Screening Array
Z-score: -5.15, p-value: 2.63E-07
n=7134
n=1844
n=509
n=631
n=867
PRS: Educational attainment
0.0
−0.2
−0.4
−0.6
−0.8
totally disagree
somewhat disagree
neutral
somewhat agree
totally agree
Not feeling obligated to comply with measures
HumanCytoSNP-12
Z-score: -1.99, p-value: 4.71E-02
n=3002
n=677
n=258
n=306
n=367
PRS: Educational attainment
0.2
0.0
−0.2
−0.4
−0.6
−0.8
totally disagree
somewhat disagree
neutral
somewhat agree
totally agree
Not feeling obligated to comply with measures

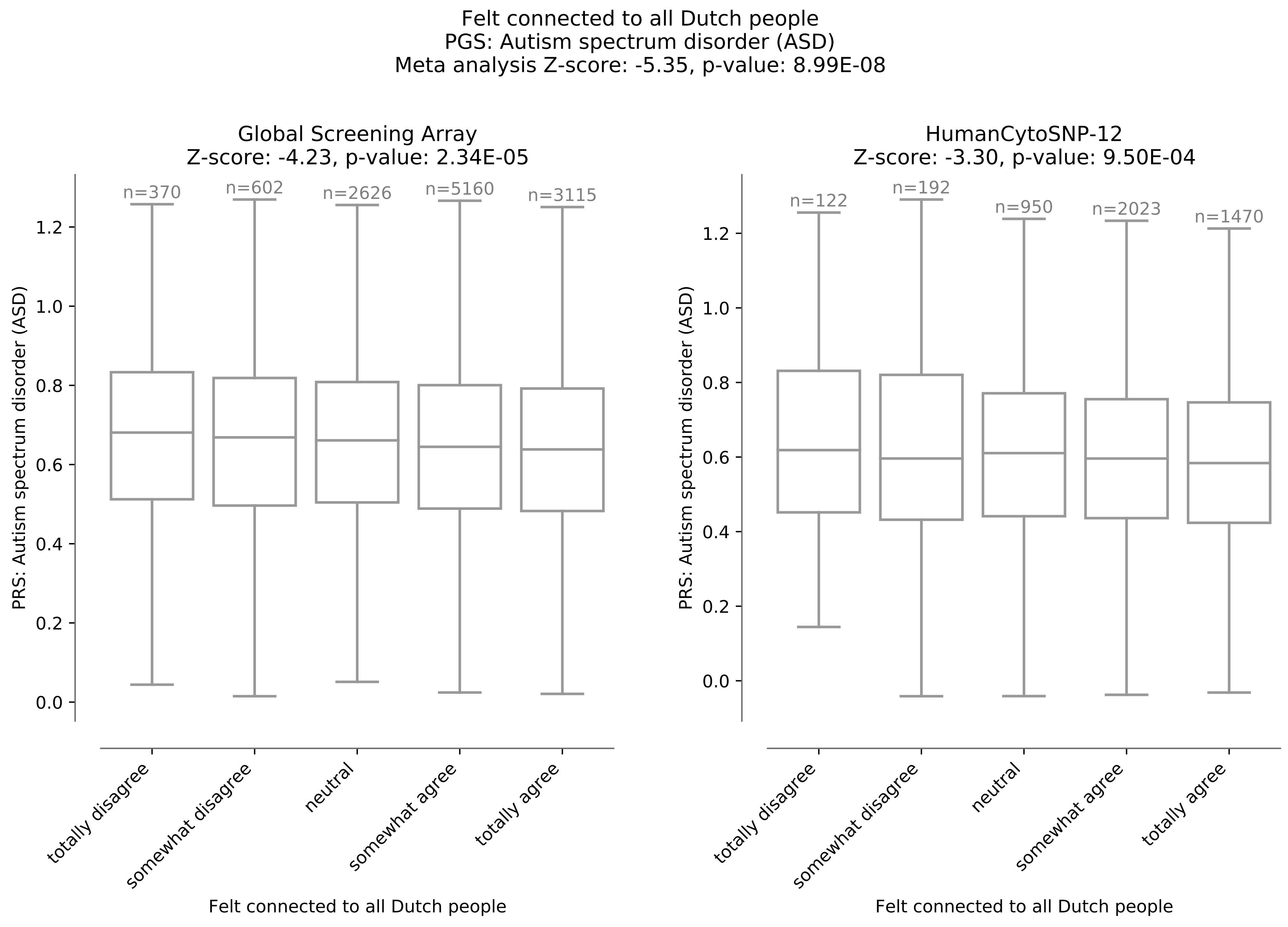
Felt connected to all Dutch people
PGS: Autism spectrum disorder (ASD)
Meta analysis Z-score: -5.35, p-value: 8.99E-08
Global Screening Array
Z-score: -4.23, p-value: 2.34E-05
n=370
n=602
n=2626
n=5160
n=3115
PRS: Autism spectrum disorder (ASD)
0.0
0.2
0.4
0.6
0.8
1.0
1.2
totally disagree
somewhat disagree
neutral
somewhat agree
totally agree
Felt connected to all Dutch people
HumanCytoSNP-12
Z-score: -3.30, p-value: 9.50E-04
n=122
n=192
n=950
n=2023
n=1470
PRS: Autism spectrum disorder (ASD)
0.0
0.2
0.4
0.6
0.8
1.0
1.2
totally disagree
somewhat disagree
neutral
somewhat agree
totally agree
Felt connected to all Dutch people

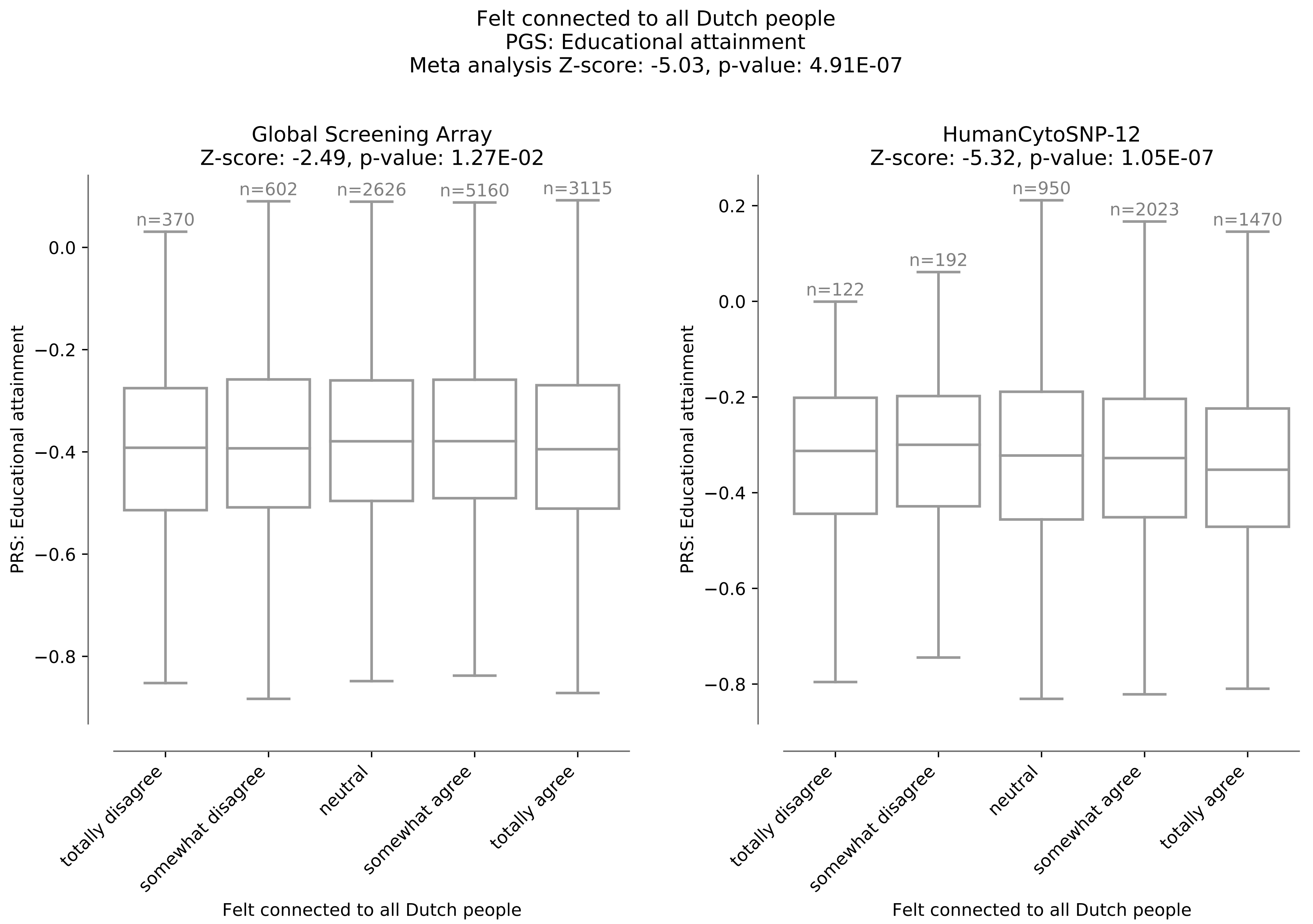

Felt connected to all Dutch people
PGS: Educational attainment
Meta analysis Z-score: -5.03, p-value: 4.91E-07
Global Screening Array
Z-score: -2.49, p-value: 1.27E-02
HumanCytoSNP-12
Z-score: -5.32, p-value: 1.05E-07
n=370
n=602
n=2626
n=5160
n=3115
n=122
n=192
n=950
n=2023
n=1470
PRS: Educational attainment
0.2
0.0
−0.2
−0.4
−0.6
−0.8
totally disagree
somewhat disagree
neutral
somewhat agree
totally agree
Felt connected to all Dutch people

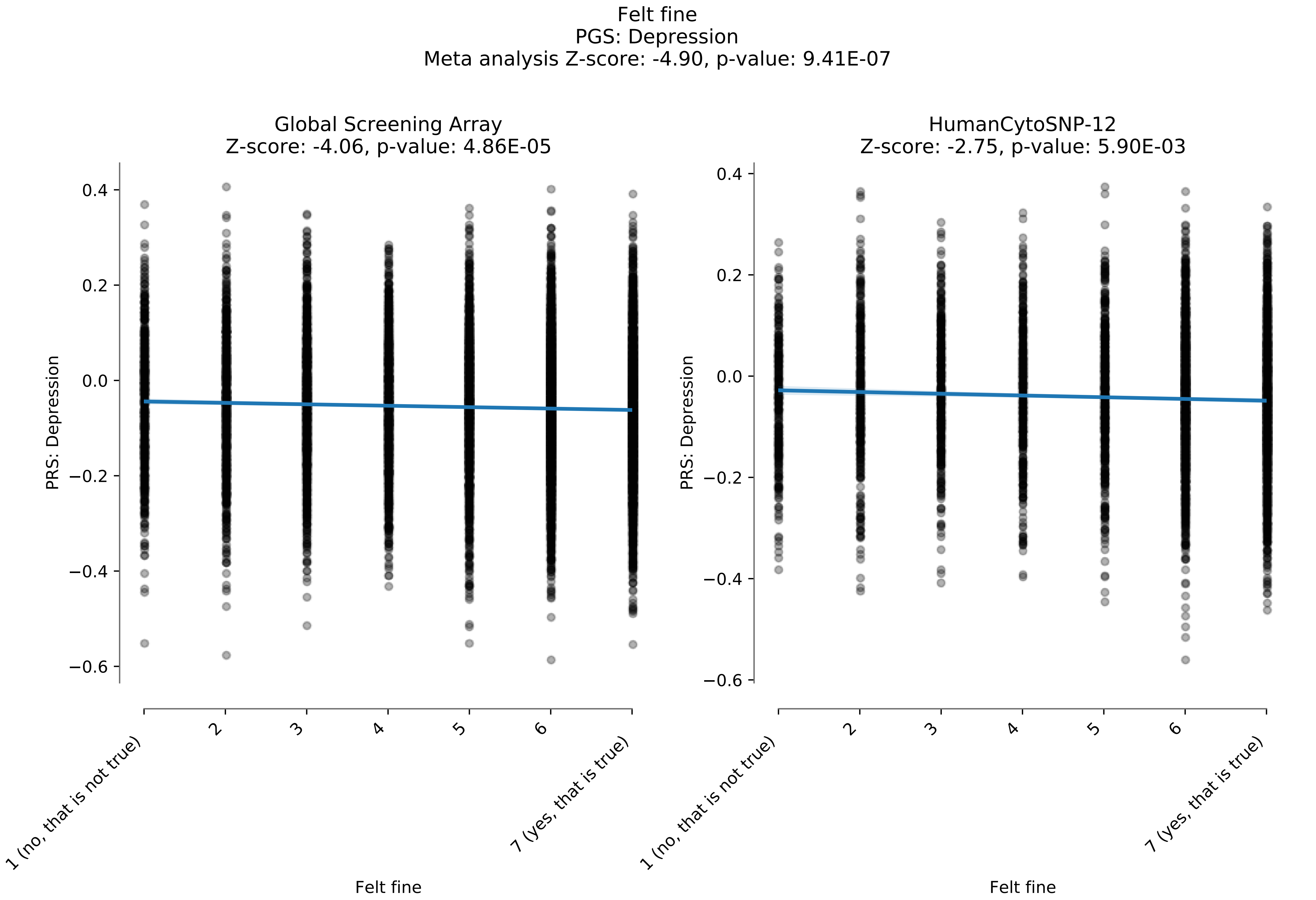

Felt fine
PGS: Depression
Meta analysis Z-score: -4.90, p-value: 9.41E-07
Global Screening Array
Z-score: -4.06, p-value: 4.86E-05
HumanCytoSNP-12
Z-score: -2.75, p-value: 5.90E-03
PRS: Depression
0.4
0.2
0.0
−0.2
−0.4
−0.6
1 (no, that is not true)
2
3
4
5
6
7 (yes, that is true)
Felt fine

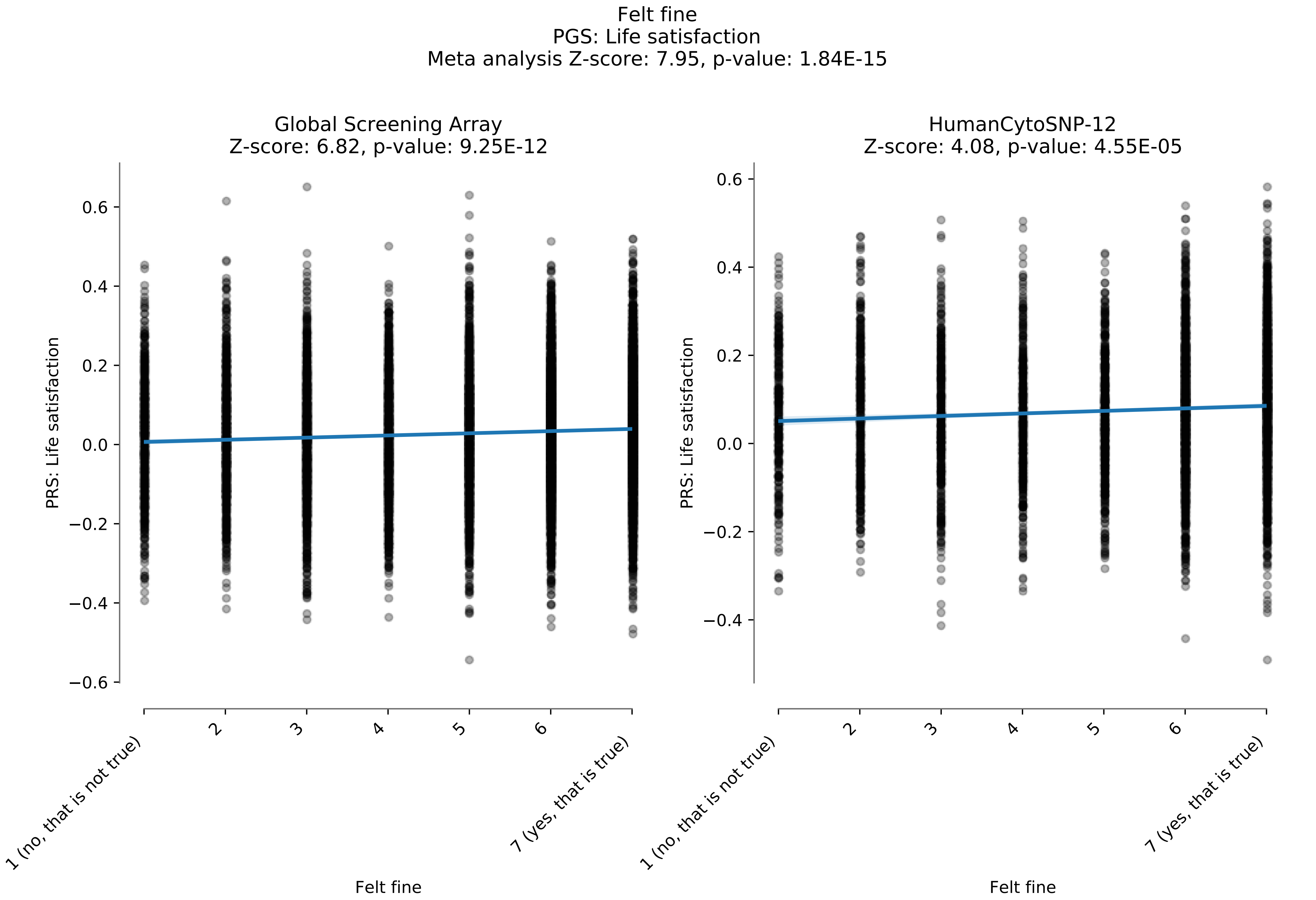
Felt fine
PGS: Life satisfaction
Meta analysis Z-score: 7.95, p-value: 1.84E-15
Global Screening Array
Z-score: 6.82, p-value: 9.25E-12
HumanCytoSNP-12
Z-score: 4.08, p-value: 4.55E-05
PRS: Life satisfaction
0.6
0.4
0.2
0.0
−0.2
−0.4
−0.6
1 (no, that is not true)
2
3
4
5
6
7 (yes, that is true)
Felt fine

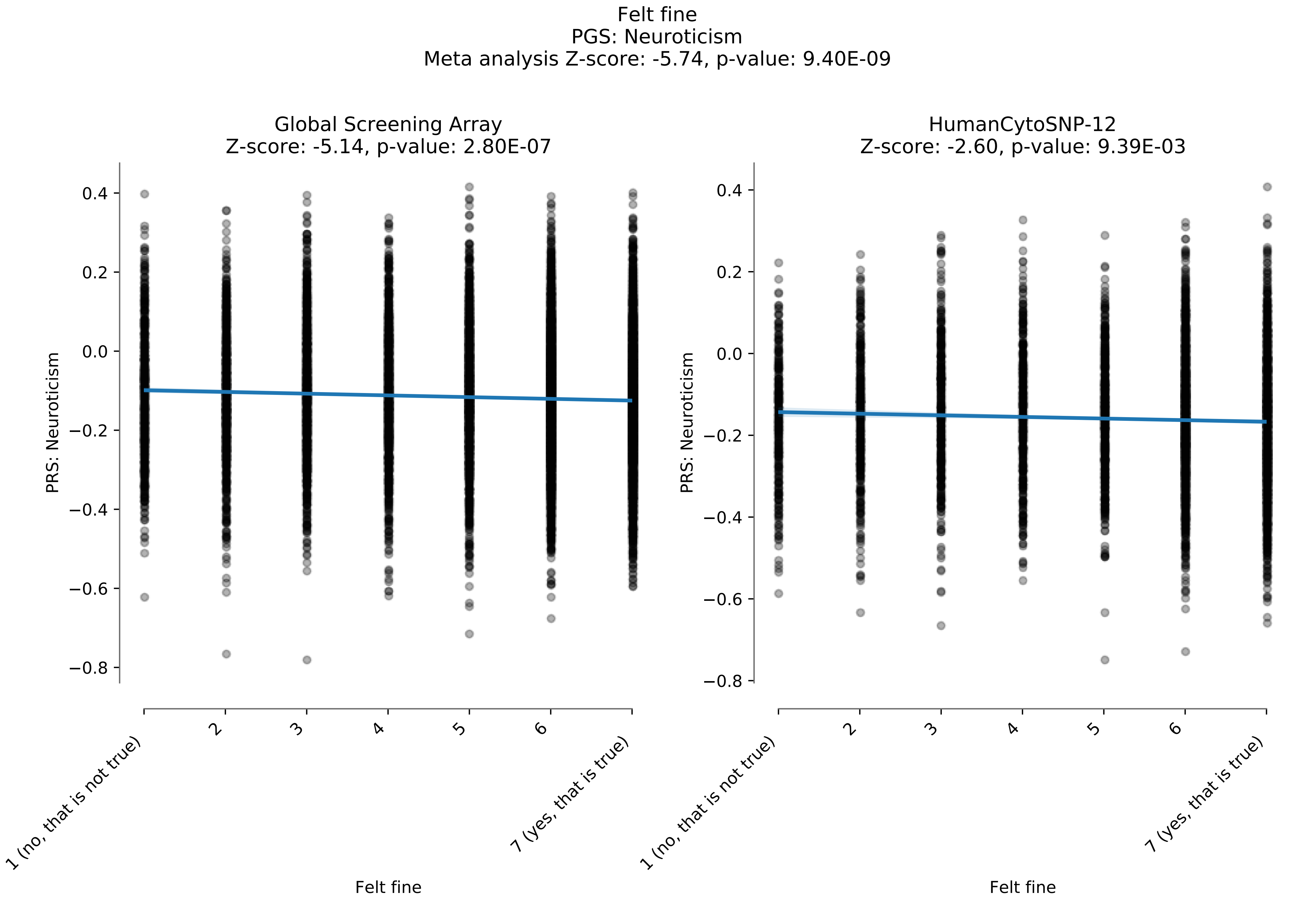
Felt fine
PGS: Neuroticism
Meta analysis Z-score: -5.74, p-value: 9.40E-09
Global Screening Array
Z-score: -5.14, p-value: 2.80E-07
HumanCytoSNP-12
Z-score: -2.60, p-value: 9.39E-03
PRS: Neuroticism
0.4
0.2
0.0
−0.2
−0.4
−0.6
−0.8
1 (no, that is not true)
2
3
4
5
6
7 (yes, that is true)
Felt fine

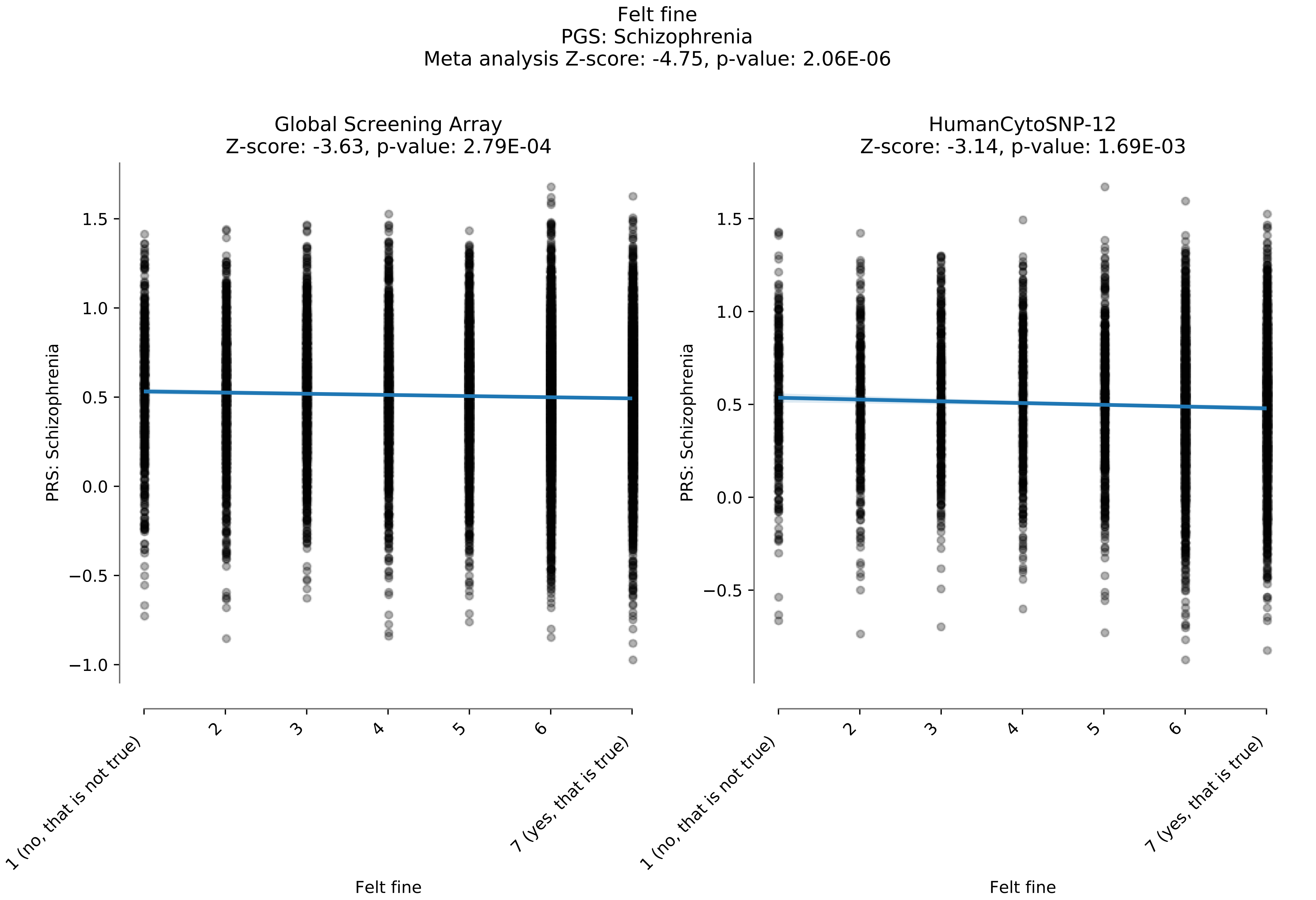
Felt fine
PGS: Schizophrenia
Meta analysis Z-score: -4.75, p-value: 2.06E-06
Global Screening Array
Z-score: -3.63, p-value: 2.79E-04
HumanCytoSNP-12
Z-score: -3.14, p-value: 1.69E-03
PRS: Schizophrenia
1.5
1.0
0.5
0.0
−0.5
−1.0
1 (no, that is not true)
2
3
4
5
6
7 (yes, that is true)
Felt fine

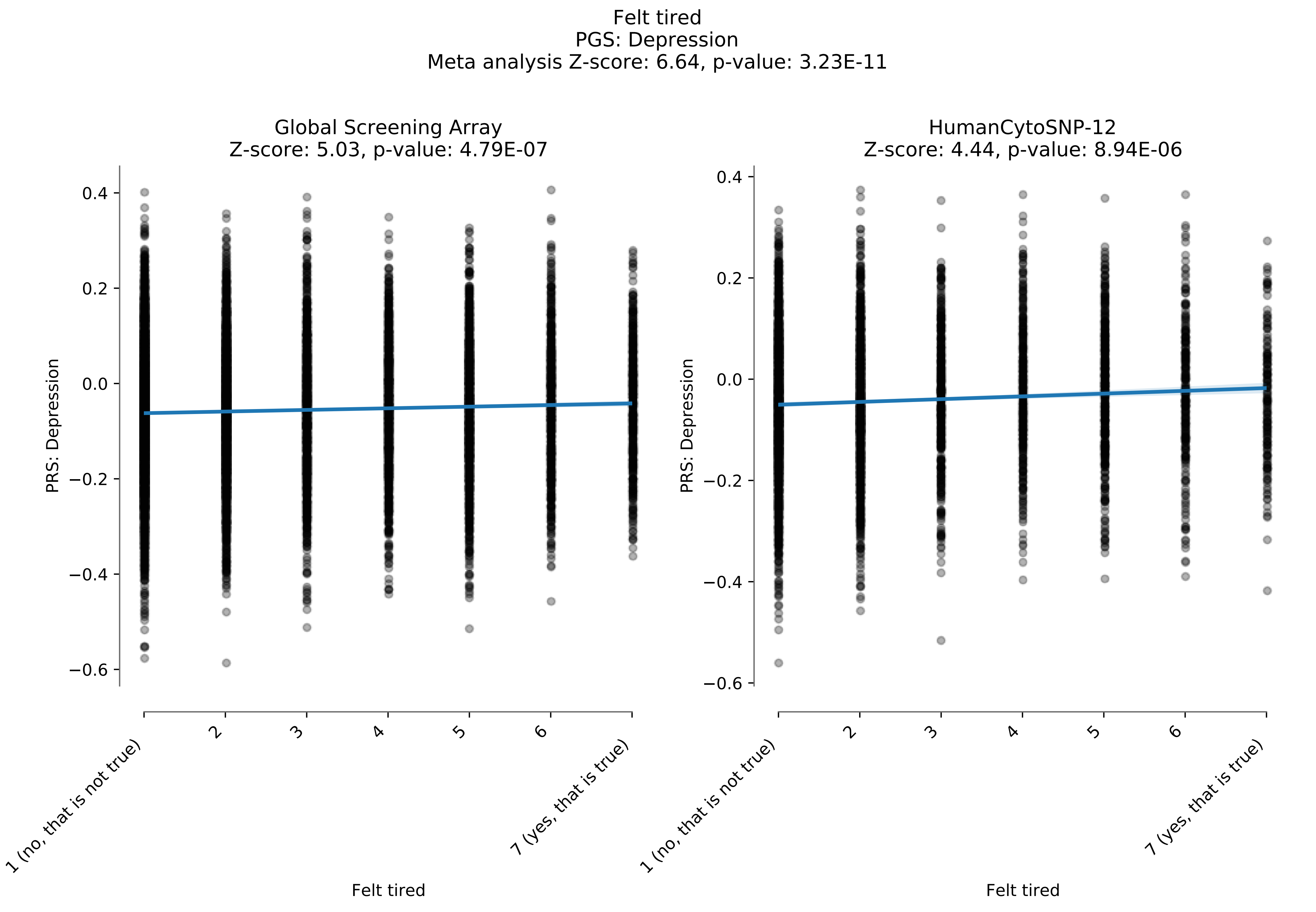
Felt tired
PGS: Depression
Meta analysis Z-score: 6.64, p-value: 3.23E-11
Global Screening Array
Z-score: 5.03, p-value: 4.79E-07
HumanCytoSNP-12
Z-score: 4.44, p-value: 8.94E-06
PRS: Depression
0.4
0.2
0.0
−0.2
−0.4
−0.6
1 (no, that is not true)
2
3
4
5
6
7 (yes, that is true)
Felt tired

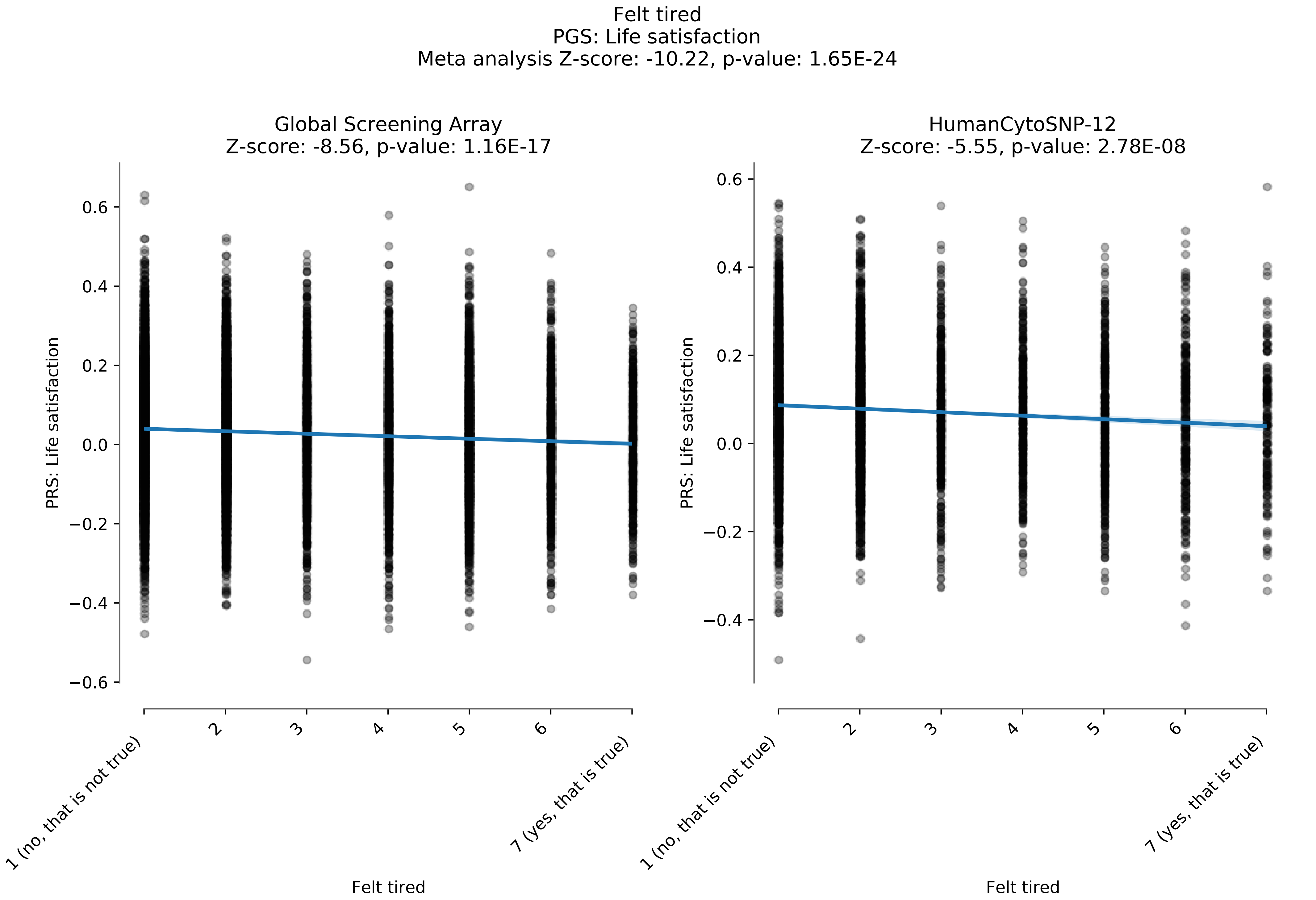

Felt tired
PGS: Life satisfaction
Meta analysis Z-score: -10.22, p-value: 1.65E-24
Global Screening Array
Z-score: -8.56, p-value: 1.16E-17
HumanCytoSNP-12
Z-score: -5.55, p-value: 2.78E-08
PRS: Life satisfaction
Felt tired
1 (no, that is not true)
2
3
4
5
6
7 (yes, that is true)
0.6
0.4
0.2
0.0
−0.2
−0.4
−0.6

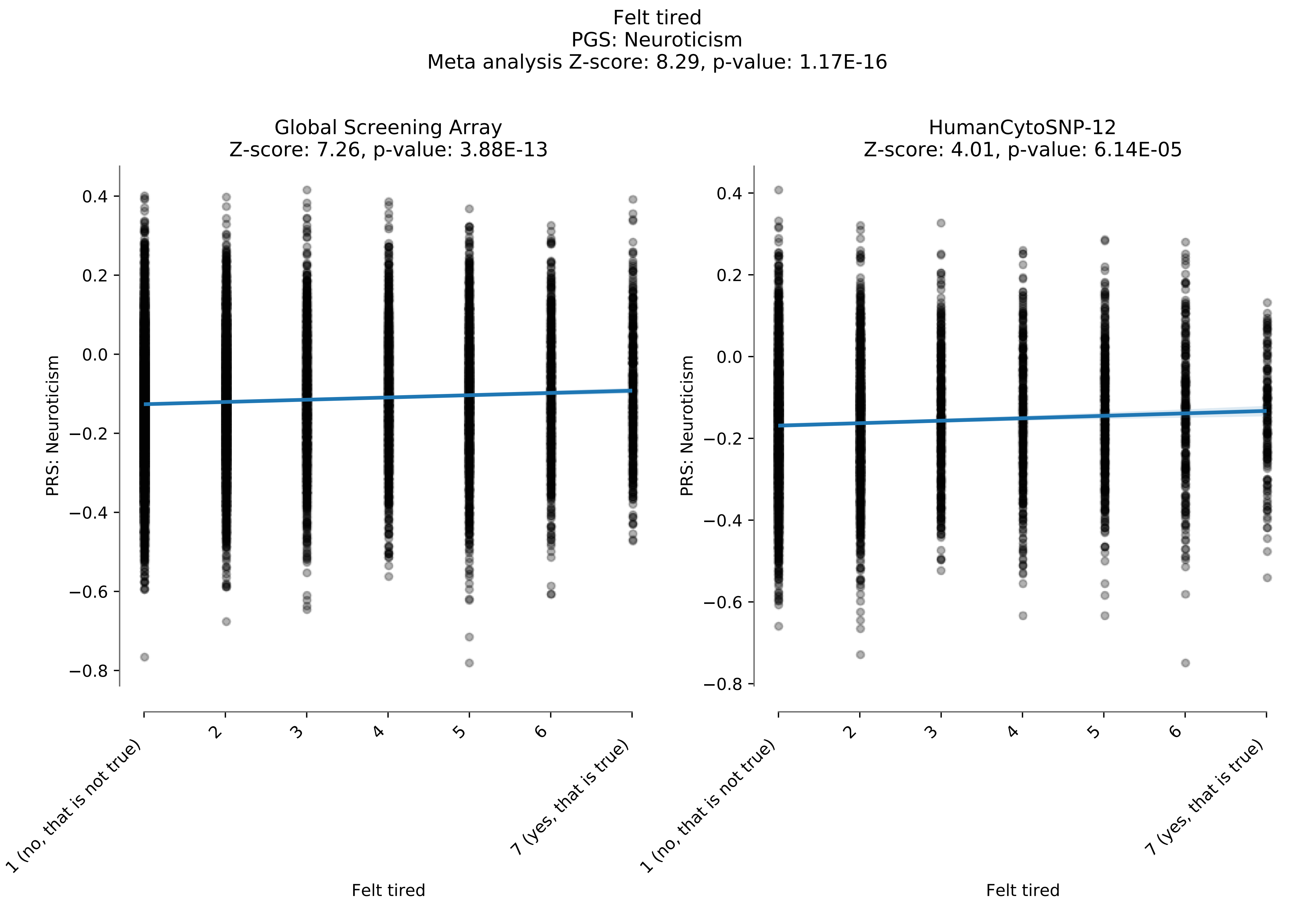

Felt tired
PGS: Neuroticism
Meta analysis Z-score: 8.29, p-value: 1.17E-16
Global Screening Array
Z-score: 7.26, p-value: 3.88E-13
HumanCytoSNP-12
Z-score: 4.01, p-value: 6.14E-05
PRS: Neuroticism
0.4
0.2
0.0
−0.2
−0.4
−0.6
−0.8
1 (no, that is not true)
2
3
4
5
6
7 (yes, that is true)
Felt tired

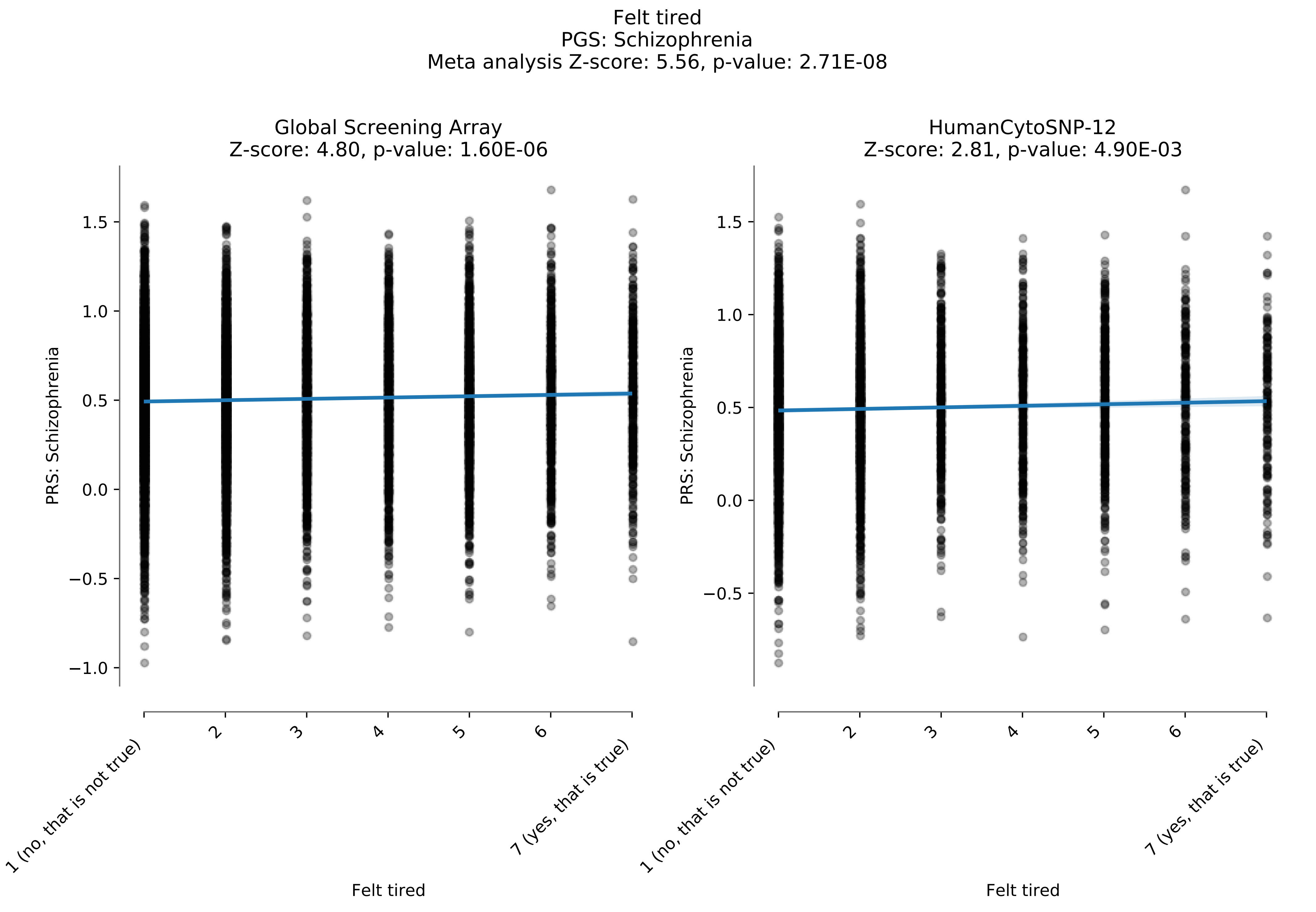
Felt tired
PGS: Schizophrenia
Meta analysis Z-score: 5.56, p-value: 2.71E-08
Global Screening Array
Z-score: 4.80, p-value: 1.60E-06
HumanCytoSNP-12
Z-score: 2.81, p-value: 4.90E-03
PRS: Schizophrenia
1.5
1.0
0.5
0.0
−0.5
−1.0
1 (no, that is not true)
2
3
4
5
6
7 (yes, that is true)
Felt tired

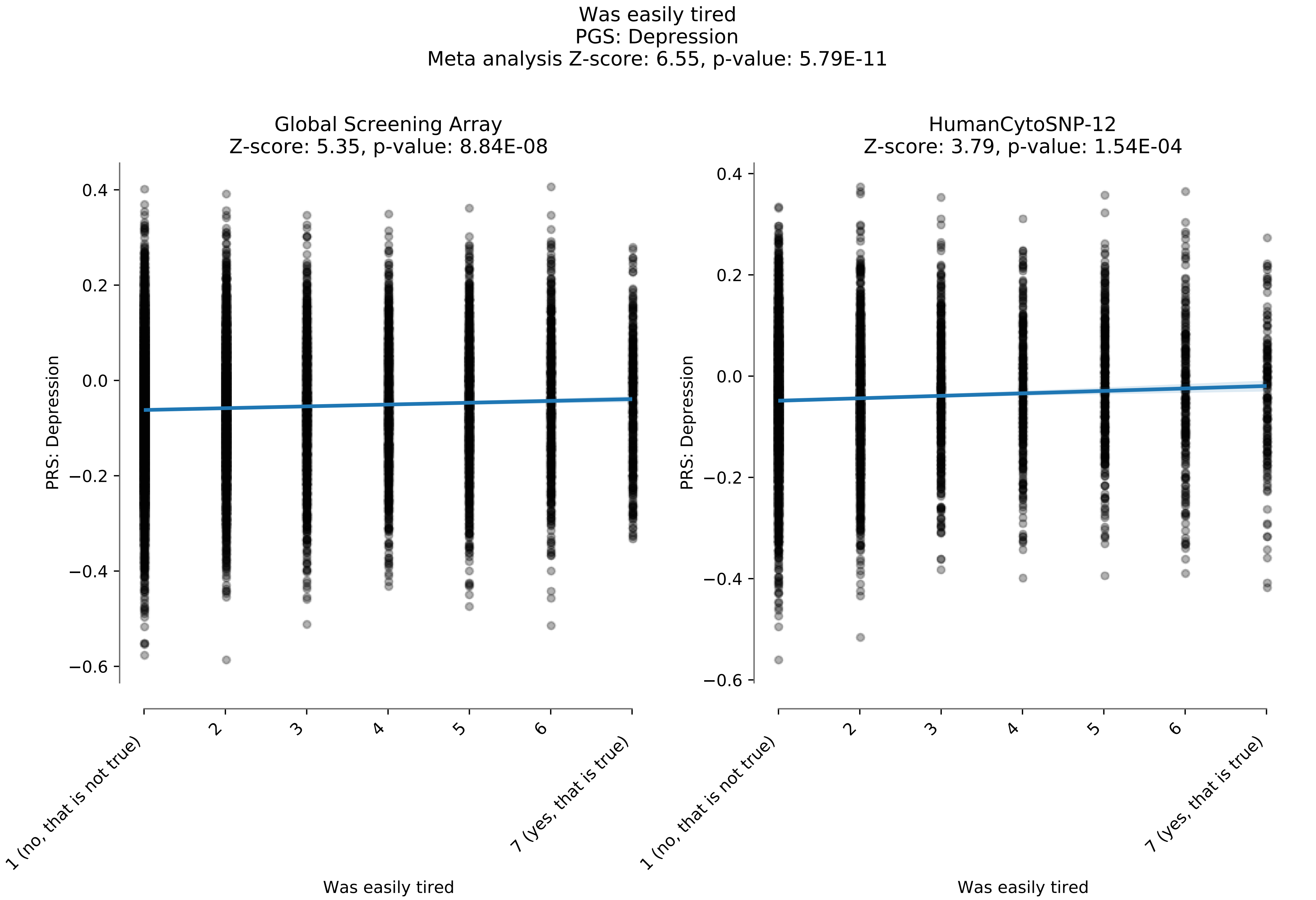

Was easily tired
PGS: Depression
Meta analysis Z-score: 6.55, p-value: 5.79E-11
Global Screening Array
Z-score: 5.35, p-value: 8.84E-08
HumanCytoSNP-12
Z-score: 3.79, p-value: 1.54E-04
PRS: Depression
0.4
0.2
0.0
−0.2
−0.4
−0.6
1 (no, that is not true)
2
3
4
5
6
7 (yes, that is true)
Was easily tired

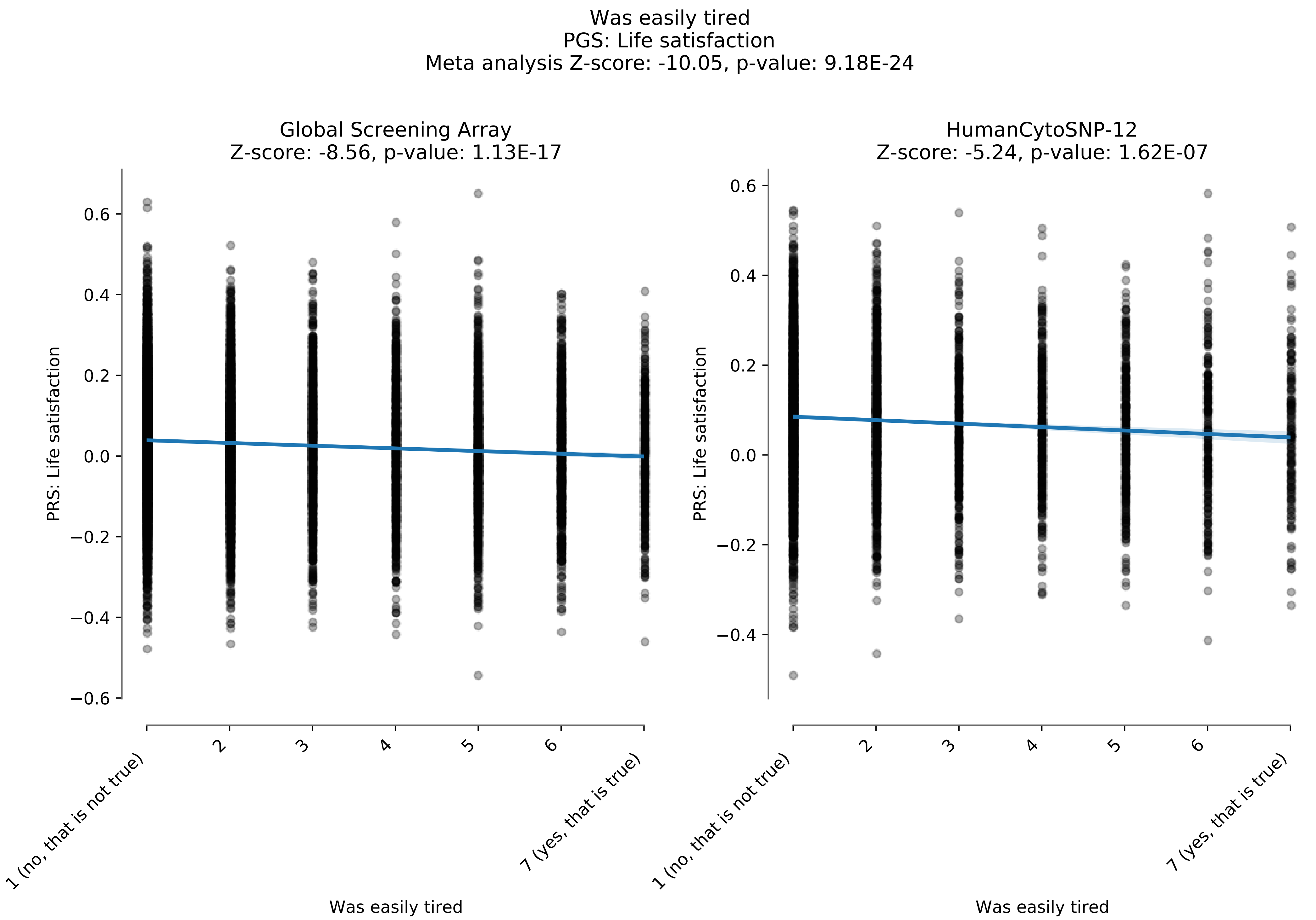

Was easily tired
PGS: Life satisfaction
Meta analysis Z-score: -10.05, p-value: 9.18E-24
Global Screening Array
Z-score: -8.56, p-value: 1.13E-17
HumanCytoSNP-12
Z-score: -5.24, p-value: 1.62E-07
PRS: Life satisfaction
0.6
0.4
0.2
0.0
−0.2
−0.4
−0.6
1 (no, that is not true)
2
3
4
5
6
7 (yes, that is true)
Was easily tired

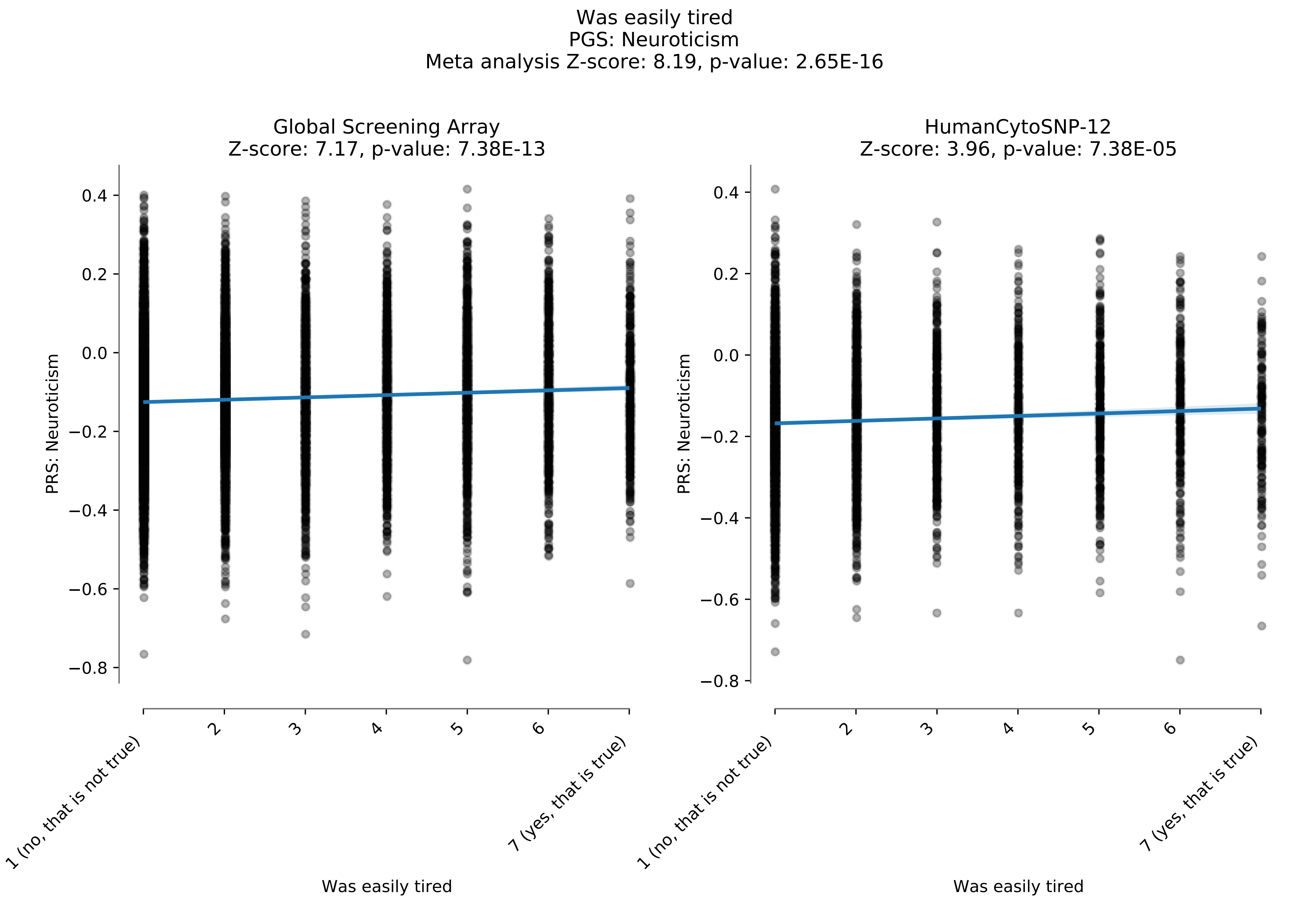

Was easily tired
PGS: Neuroticism
Meta analysis Z-score: 8.19, p-value: 2.65E-16
Global Screening Array
Z-score: 7.17, p-value: 7.38E-13
HumanCytoSNP-12
Z-score: 3.96, p-value: 7.38E-05
PRS: Neuroticism
0.4
0.2
0.0
−0.2
−0.4
−0.6
−0.8
1 (no, that is not true)
2
3
4
5
6
7 (yes, that is true)
Was easily tired

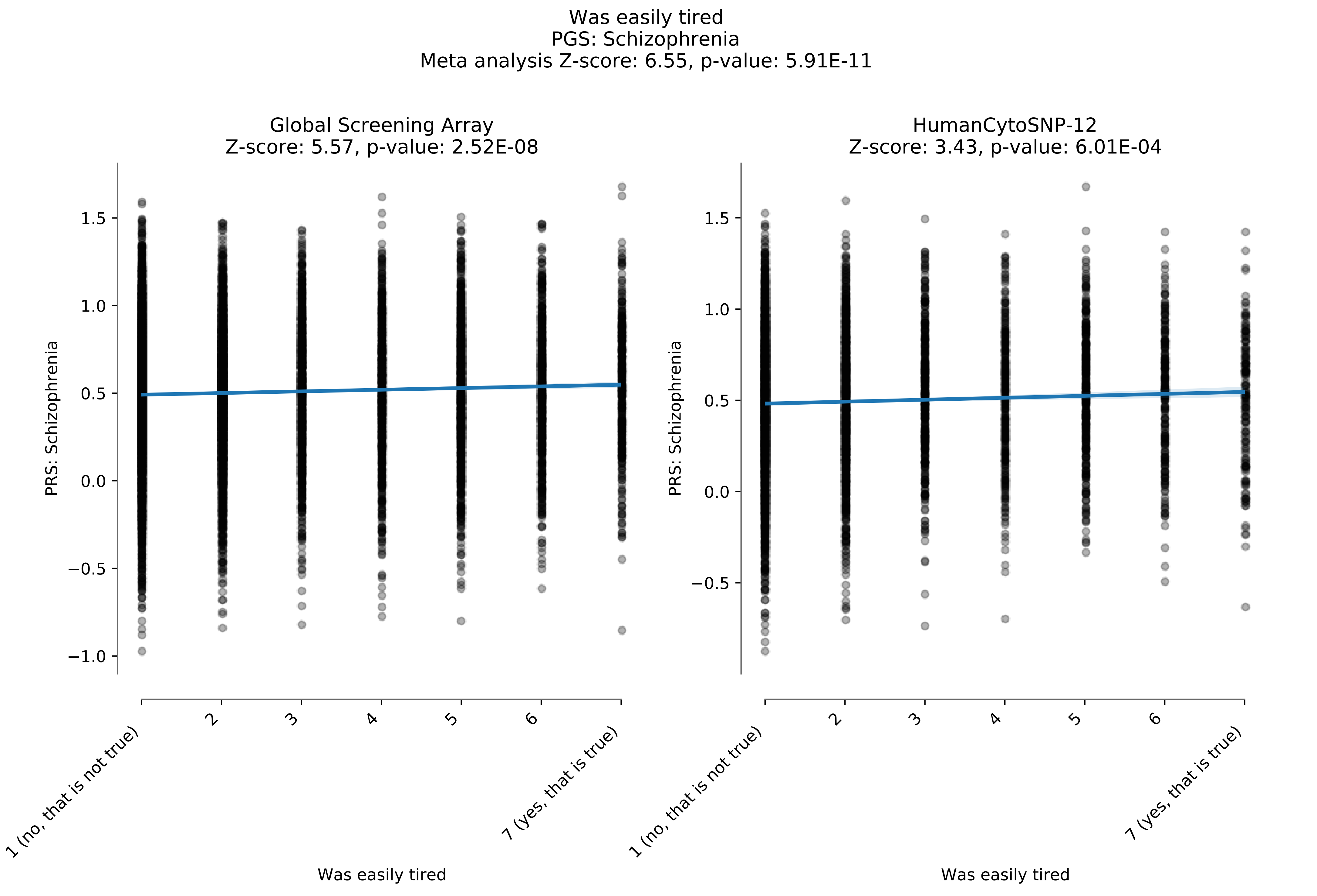

Was easily tired
PGS: Schizophrenia
Meta analysis Z-score: 6.55, p-value: 5.91E-11
Global Screening Array
Z-score: 5.57, p-value: 2.52E-08
HumanCytoSNP-12
Z-score: 3.43, p-value: 6.01E-04
PRS: Schizophrenia
1.5
1.0
0.5
0.0
−0.5
−1.0
1 (no, that is not true)
2
3
4
5
6
7 (yes, that is true)
Was easily tired

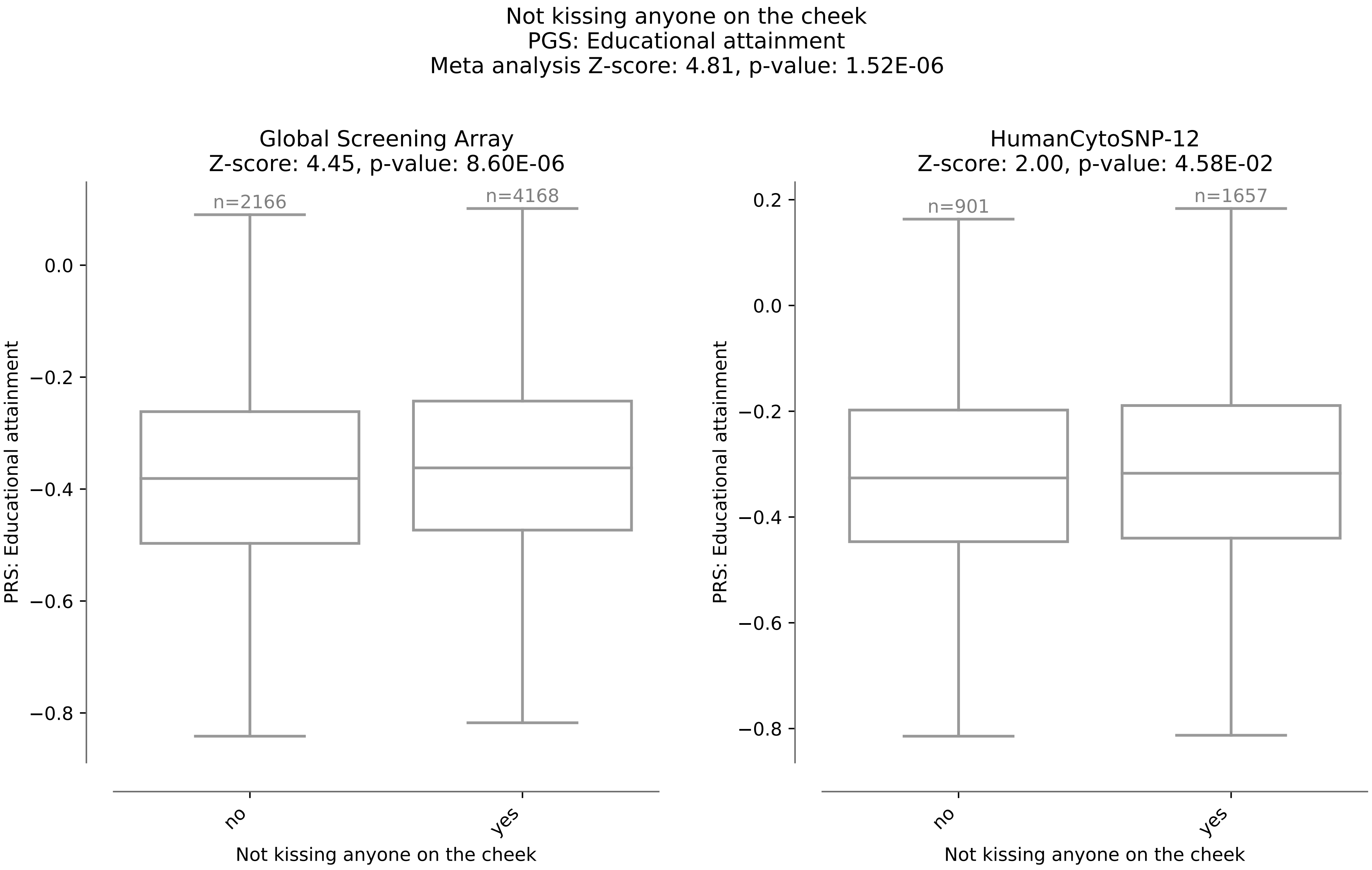

Not kissing anyone on the cheek
PGS: Educational attainment
Meta analysis Z-score: 4.81, p-value: 1.52E-06
Global Screening Array
Z-score: 4.45, p-value: 8.60E-06
n=2166
n=4168
0.0
−0.2
−0.4
−0.6
−0.8
PRS: Educational attainment
no
yes
Not kissing anyone on the cheek
HumanCytoSNP-12
Z-score: 2.00, p-value: 4.58E-02
n=901
n=1657
0.2
0.0
−0.2
−0.4
−0.6
−0.8
PRS: Educational attainment
no
yes
Not kissing anyone on the cheek

Being bothered about getting infected with COVID-19
PGS: Educational attainment
Meta analysis Z-score: -7.90, p-value: 2.86E-15

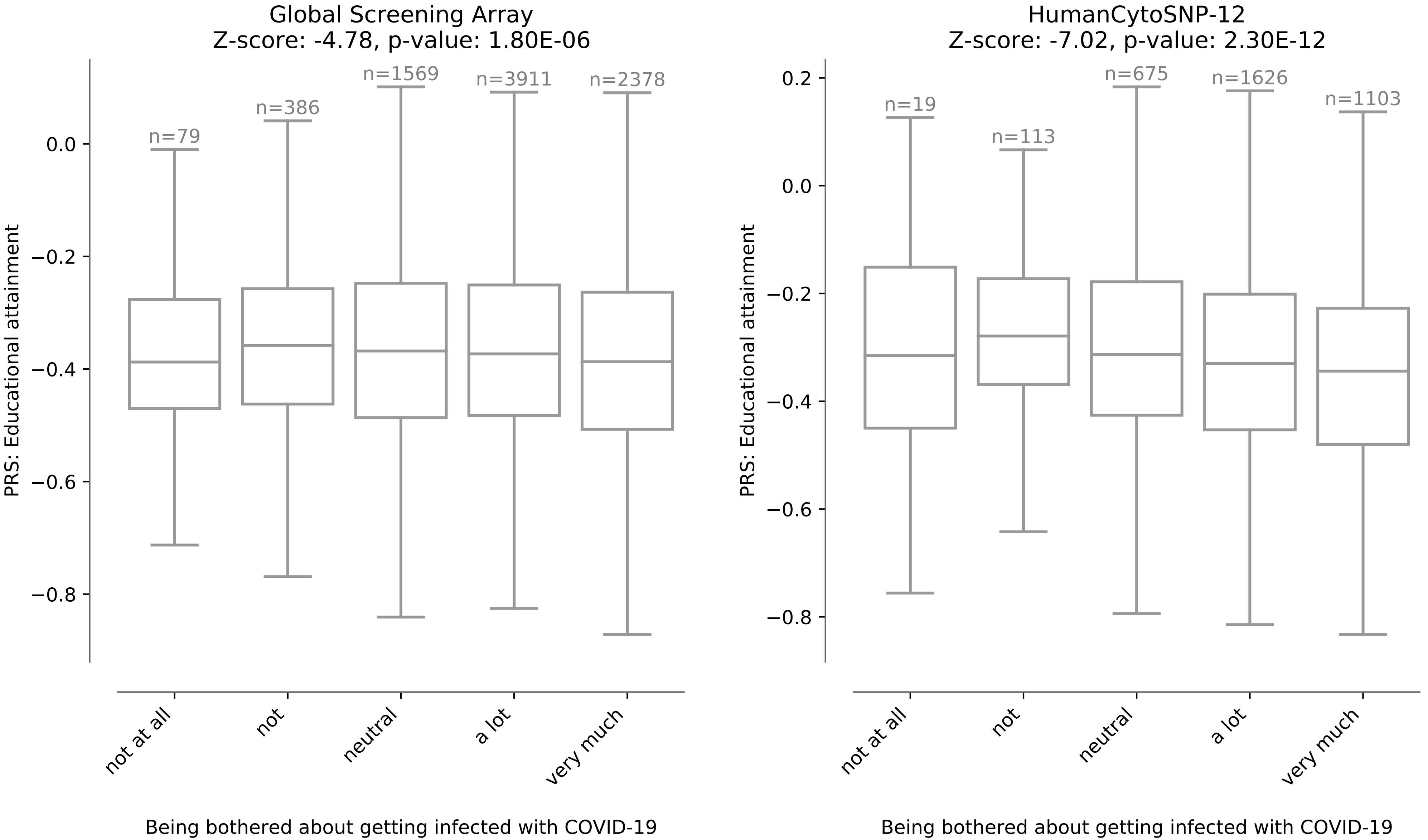

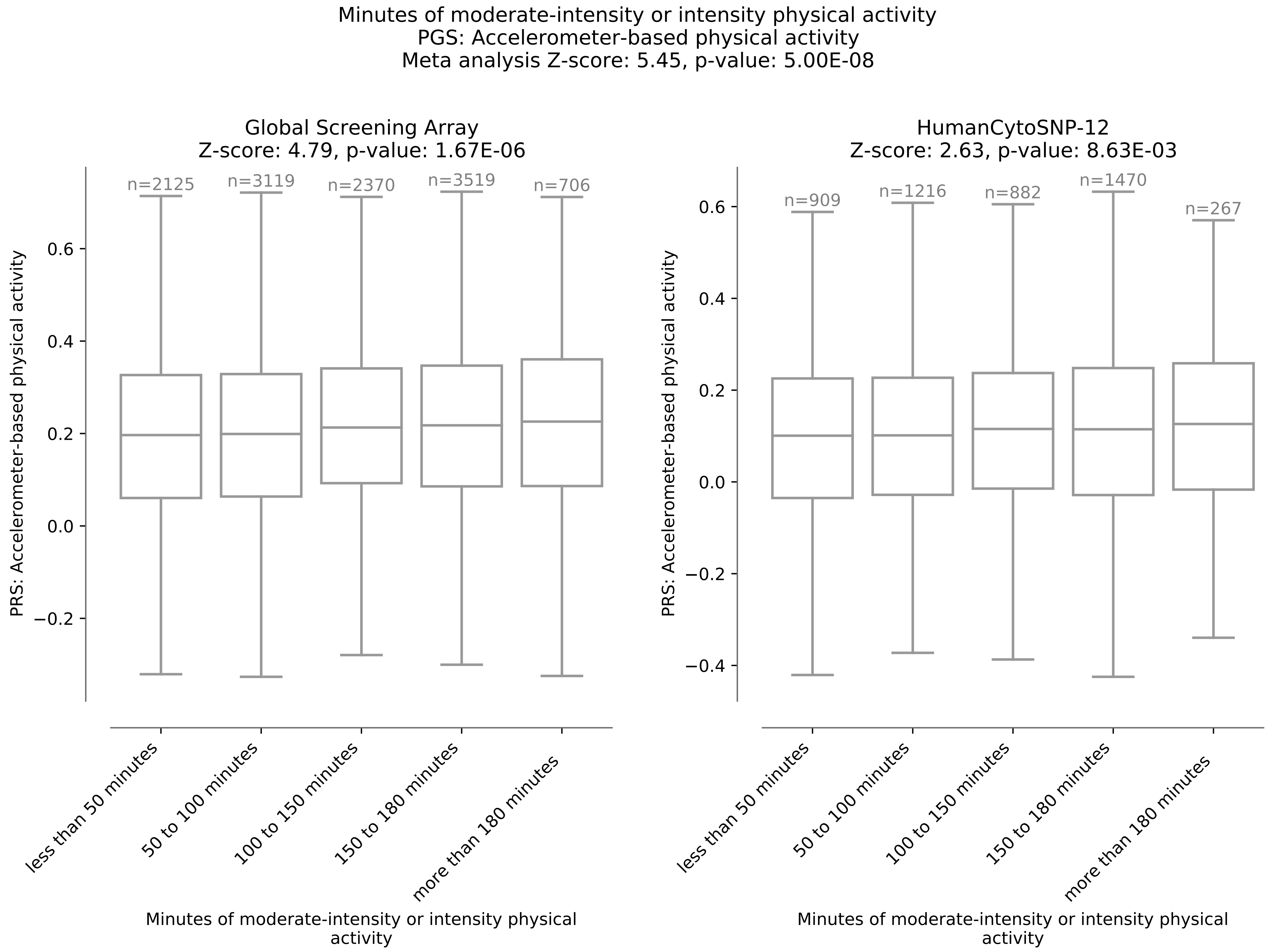
Minutes of moderate-intensity or intensity physical activity
PGS: Accelerometer-based physical activity
Meta analysis Z-score: 5.45, p-value: 5.00E-08
Global Screening Array
Z-score: 4.79, p-value: 1.67E-06
n=2125
n=3119
n=2370
n=3519
n=706
PRS: Accelerometer-based physical activity
0.6
0.4
0.2
0.0
−0.2
less than 50 minutes
50 to 100 minutes
100 to 150 minutes
150 to 180 minutes
more than 180 minutes
Minutes of moderate-intensity or intensity physical activity
HumanCytoSNP-12
Z-score: 2.63, p-value: 8.63E-03
n=909
n=1216
n=882
n=1470
n=267
PRS: Accelerometer-based physical activity
0.6
0.4
0.2
0.0
−0.2
−0.4
less than 50 minutes
50 to 100 minutes
100 to 150 minutes
150 to 180 minutes
more than 180 minutes
Minutes of moderate-intensity or intensity physical activity

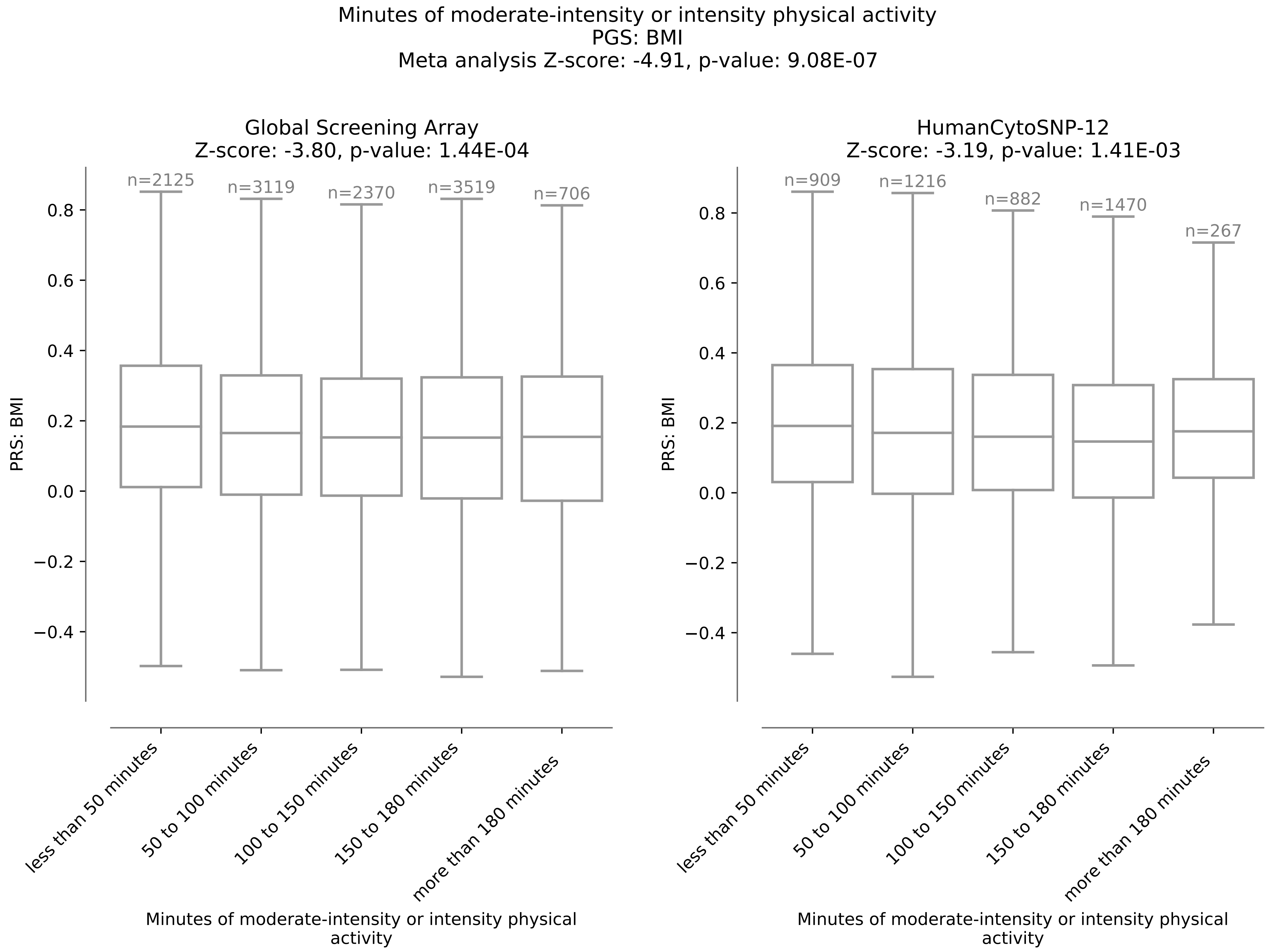

Minutes of moderate-intensity or intensity physical activity
PGS: BMI
Meta analysis Z-score: -4.91, p-value: 9.08E-07
Global Screening Array
Z-score: -3.80, p-value: 1.44E-04
n=2125
n=3119
n=2370
n=3519
n=706
PRS: BMI
0.8
0.6
0.4
0.2
0.0
−0.2
−0.4
less than 50 minutes
50 to 100 minutes
100 to 150 minutes
150 to 180 minutes
more than 180 minutes
Minutes of moderate-intensity or intensity physical activity
HumanCytoSNP-12
Z-score: -3.19, p-value: 1.41E-03
n=909
n=1216
n=882
n=1470
n=267
PRS: BMI
0.8
0.6
0.4
0.2
0.0
−0.2
−0.4
less than 50 minutes
50 to 100 minutes
100 to 150 minutes
150 to 180 minutes
more than 180 minutes
Minutes of moderate-intensity or intensity physical activity

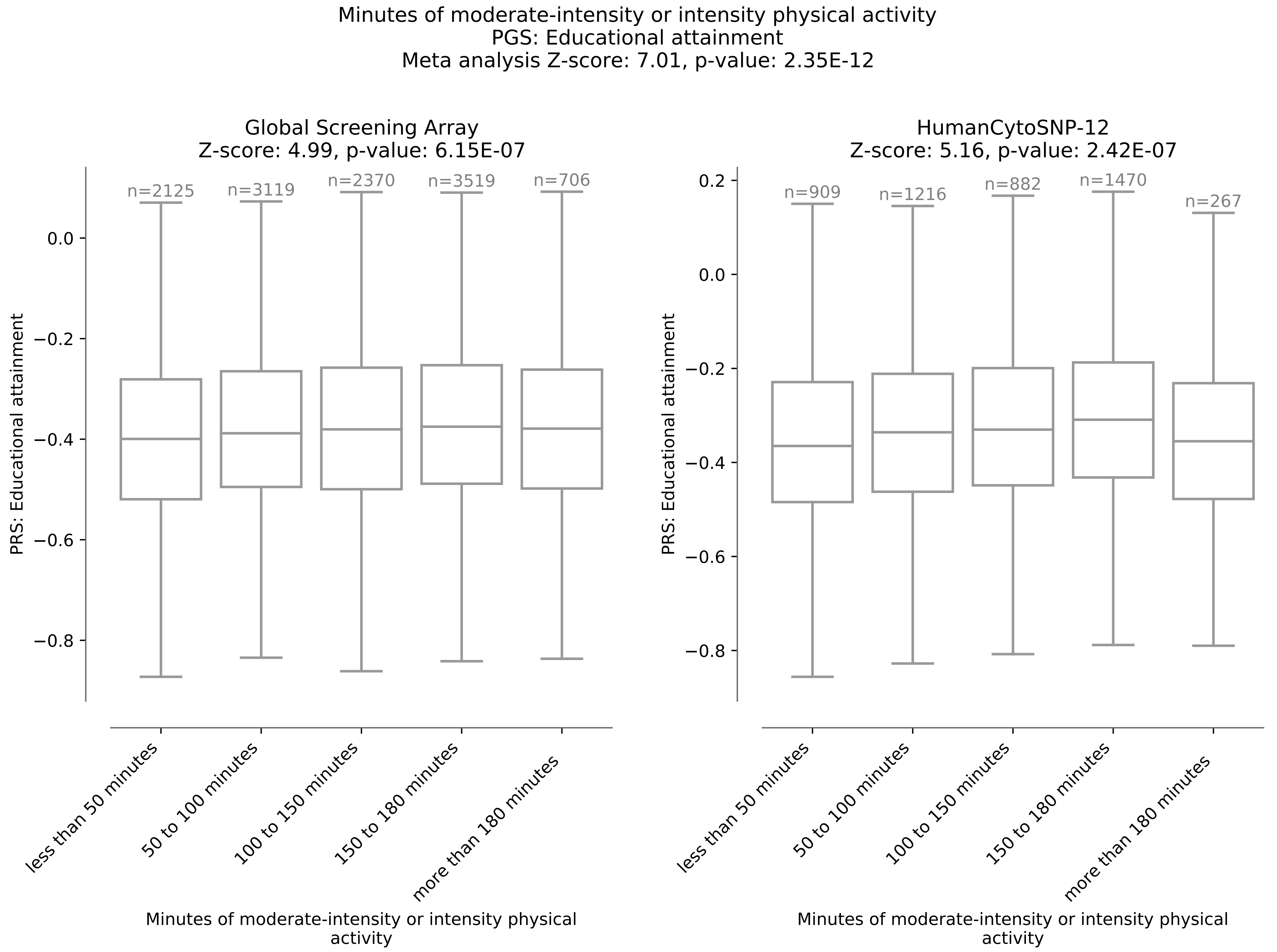
Minutes of moderate-intensity or intensity physical activity
PGS: Educational attainment
Meta analysis Z-score: 7.01, p-value: 2.35E-12
Global Screening Array
Z-score: 4.99, p-value: 6.15E-07
HumanCytoSNP-12
Z-score: 5.16, p-value: 2.42E-07
PRS: Educational attainment
n=2125
n=3119
n=2370
n=3519
n=706
n=909
n=1216
n=882
n=1470
n=267
less than 50 minutes
50 to 100 minutes
100 to 150 minutes
150 to 180 minutes
more than 180 minutes
Minutes of moderate-intensity or intensity physical activity

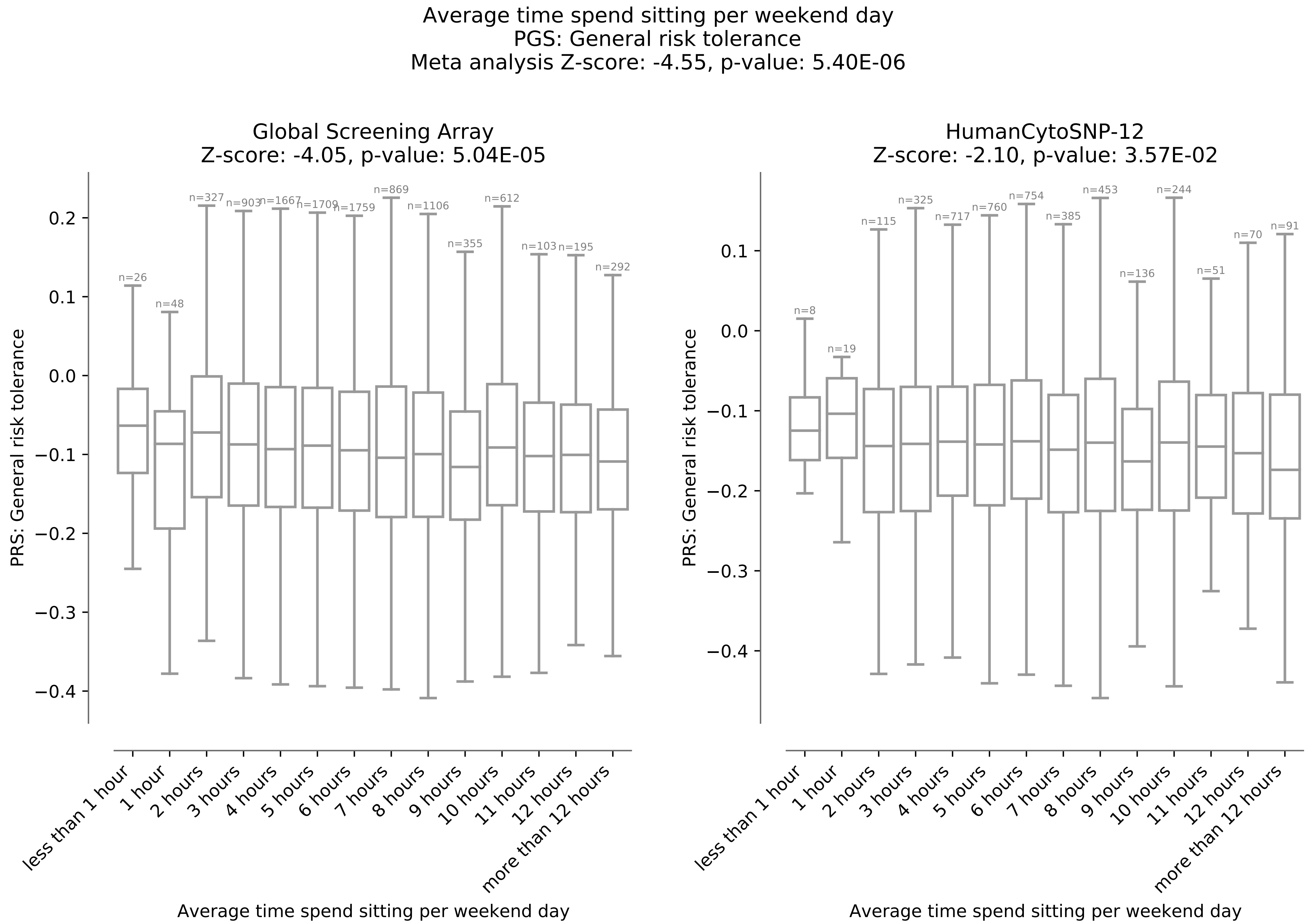
Average time spend sitting per weekend day
PGS: General risk tolerance
Meta analysis Z-score: -4.55, p-value: 5.40E-06
Global Screening Array
Z-score: -4.05, p-value: 5.04E-05
HumanCytoSNP-12
Z-score: -2.10, p-value: 3.57E-02
PRS: General risk tolerance
Average time spend sitting per weekend day
less than 1 hour
1 hour
2 hours
3 hours
4 hours
5 hours
6 hours
7 hours
8 hours
9 hours
10 hours
11 hours
12 hours
more than 12 hours
n=26
n=48
n=327
n=903
n=1667
n=1709
n=1759
n=869
n=1106
n=355
n=612
n=103
n=195
n=292
n=8
n=19
n=115
n=325
n=717
n=760
n=754
n=385
n=453
n=136
n=244
n=51
n=70
n=91

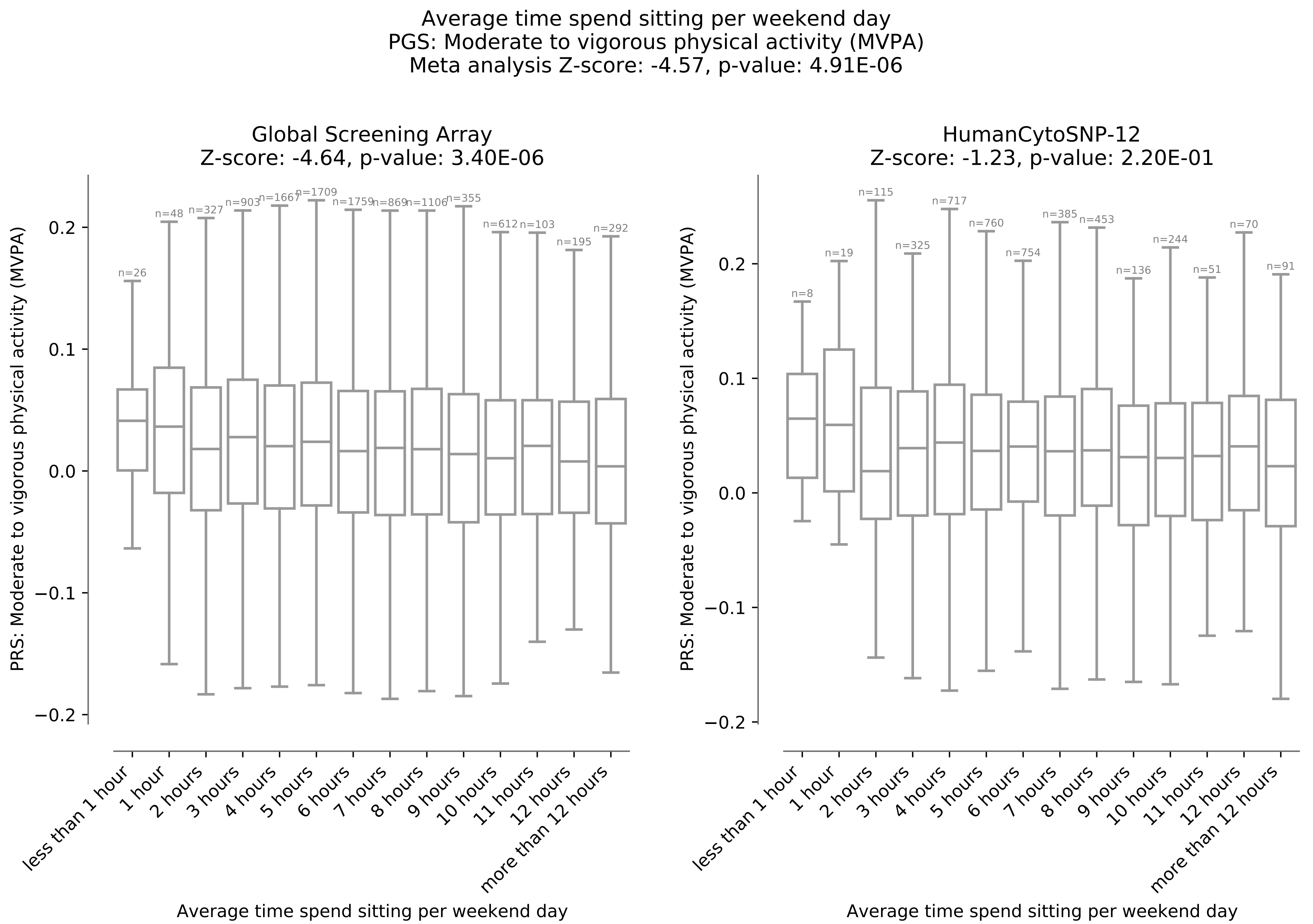

Average time spend sitting per weekend day
PGS: Moderate to vigorous physical activity (MVPA)
Meta analysis Z-score: -4.57, p-value: 4.91E-06
Global Screening Array
Z-score: -4.64, p-value: 3.40E-06
HumanCytoSNP-12
Z-score: -1.23, p-value: 2.20E-01
PRS: Moderate to vigorous physical activity (MVPA)
Average time spend sitting per weekend day
less than 1 hour
1 hour
2 hours
3 hours
4 hours
5 hours
6 hours
7 hours
8 hours
9 hours
10 hours
11 hours
12 hours
more than 12 hours
n=26
n=48
n=327
n=903
n=1667
n=1709
n=1759
n=869
n=1106
n=355
n=612
n=103
n=195
n=292
n=8
n=19
n=115
n=325
n=717
n=760
n=754
n=385
n=453
n=136
n=244
n=51
n=70
n=91

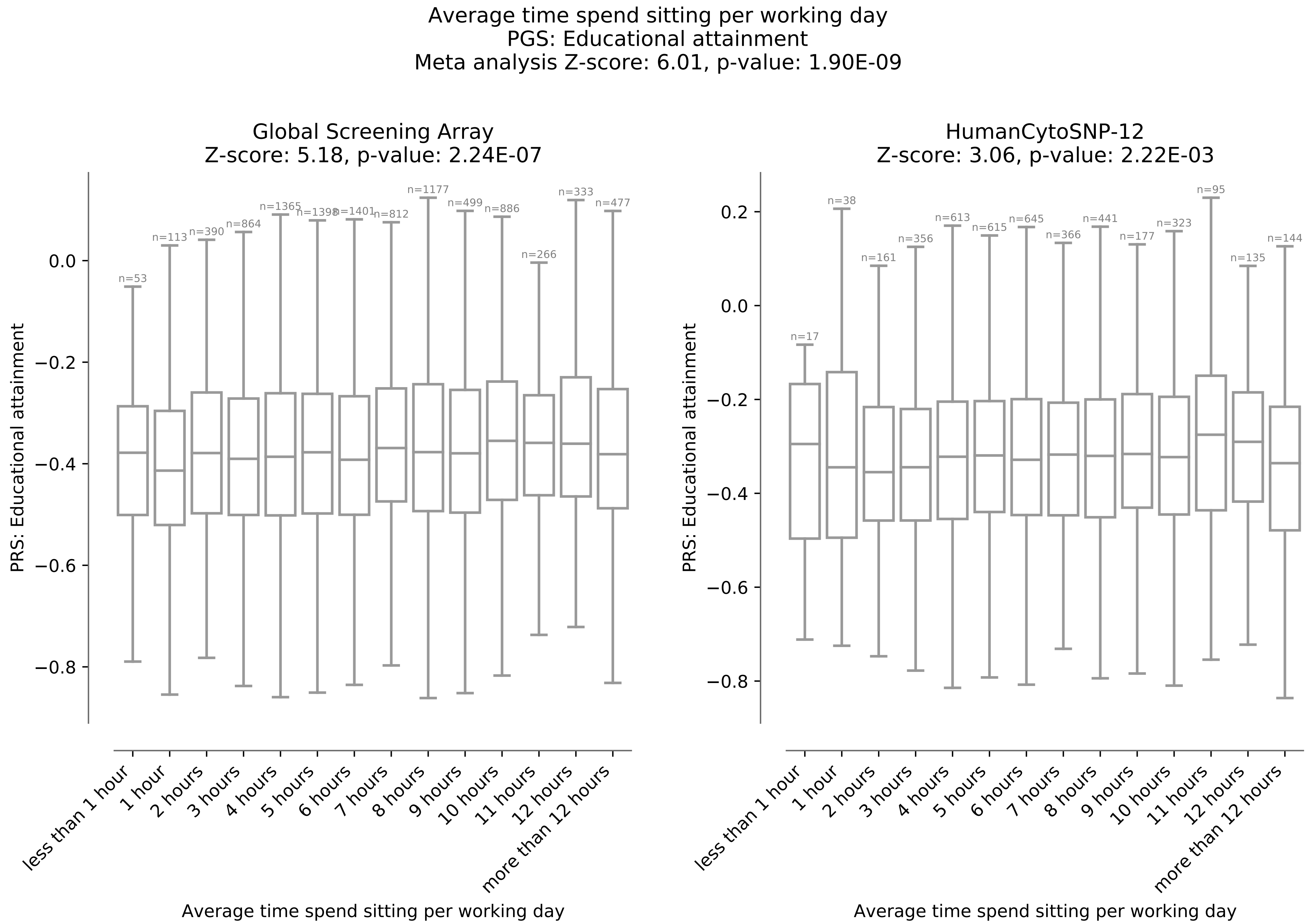

Average time spend sitting per working day
PGS: Educational attainment
Meta analysis Z-score: 6.01, p-value: 1.90E-09
Global Screening Array
Z-score: 5.18, p-value: 2.24E-07
HumanCytoSNP-12
Z-score: 3.06, p-value: 2.22E-03
PRS: Educational attainment
n=53
n=113
n=390
n=864
n=1365
n=1398
n=1401
n=812
n=1177
n=499
n=886
n=266
n=333
n=477
n=17
n=38
n=161
n=356
n=613
n=615
n=645
n=366
n=441
n=177
n=323
n=95
n=135
n=144
less than 1 hour
1 hour
2 hours
3 hours
4 hours
5 hours
6 hours
7 hours
8 hours
9 hours
10 hours
11 hours
12 hours
more than 12 hours
Average time spend sitting per working day

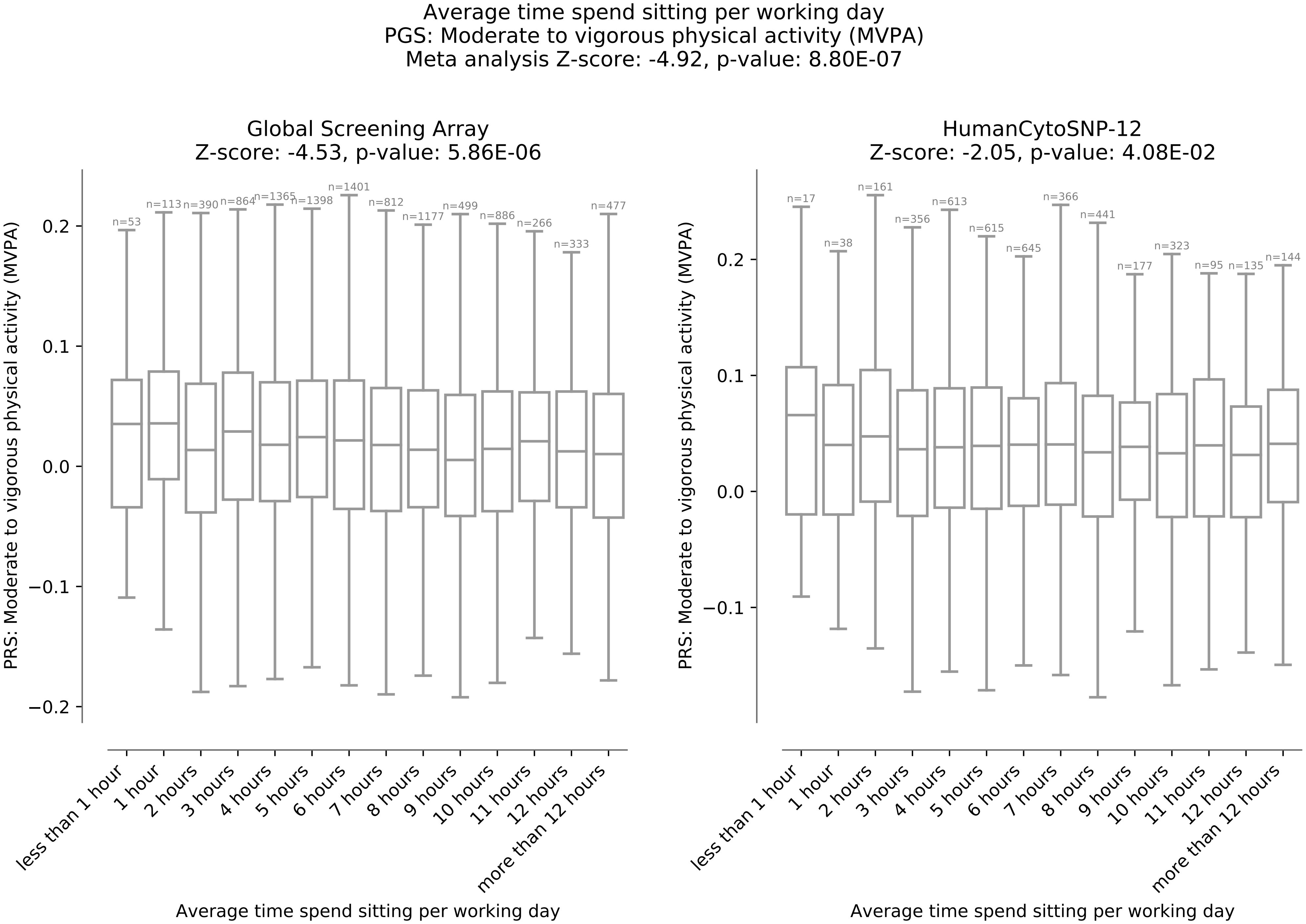
Average time spend sitting per working day
PGS: Moderate to vigorous physical activity (MVPA)
Meta analysis Z-score: -4.92, p-value: 8.80E-07
Global Screening Array
Z-score: -4.53, p-value: 5.86E-06
HumanCytoSNP-12
Z-score: -2.05, p-value: 4.08E-02
PRS: Moderate to vigorous physical activity (MVPA)
n=53
n=113
n=390
n=864
n=1365
n=1398
n=1401
n=812
n=1177
n=499
n=886
n=266
n=333
n=477
n=17
n=38
n=161
n=356
n=613
n=615
n=645
n=366
n=441
n=177
n=323
n=95
n=135
n=144
0.2
0.1
0.0
−0.1
−0.2
less than 1 hour
1 hour
2 hours
3 hours
4 hours
5 hours
6 hours
7 hours
8 hours
9 hours
10 hours
11 hours
12 hours
more than 12 hours
Average time spend sitting per working day

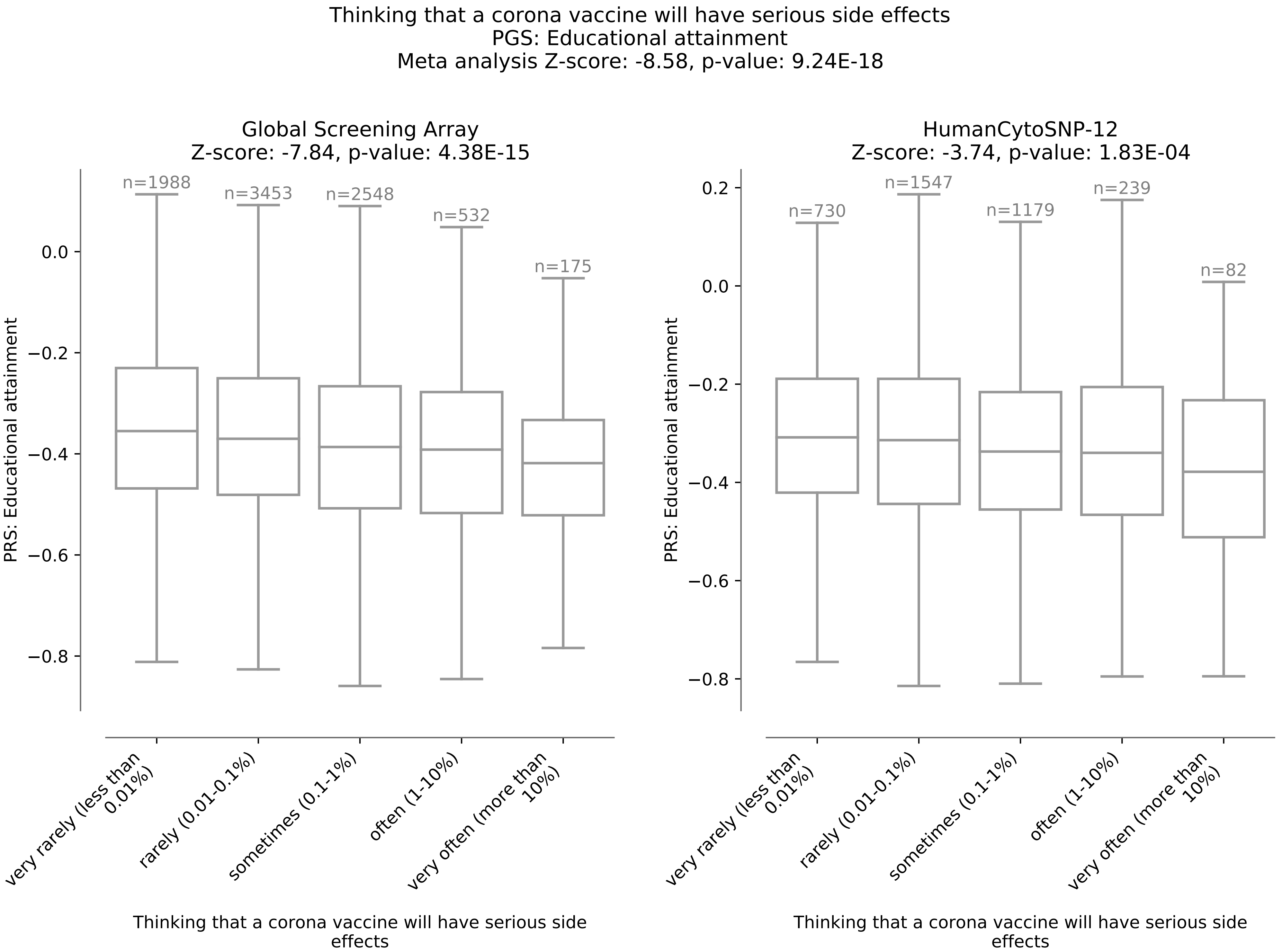
Thinking that a corona vaccine will have serious side effects
PGS: Educational attainment
Meta analysis Z-score: -8.58, p-value: 9.24E-18
Global Screening Array
Z-score: -7.84, p-value: 4.38E-15
n=1988
n=3453
n=2548
n=532
n=175
0.0
−0.2
−0.4
−0.6
−0.8
PRS: Educational attainment
very rarely (less than 0.01%)
rarely (0.01-0.1%)
sometimes (0.1-1%)
often (1-10%)
very often (more than 10%)
Thinking that a corona vaccine will have serious side effects
HumanCytoSNP-12
Z-score: -3.74, p-value: 1.83E-04
n=730
n=1547
n=1179
n=239
n=82
0.2
0.0
−0.2
−0.4
−0.6
−0.8
PRS: Educational attainment
very rarely (less than 0.01%)
rarely (0.01-0.1%)
sometimes (0.1-1%)
often (1-10%)
very often (more than 10%)
Thinking that a corona vaccine will have serious side effects

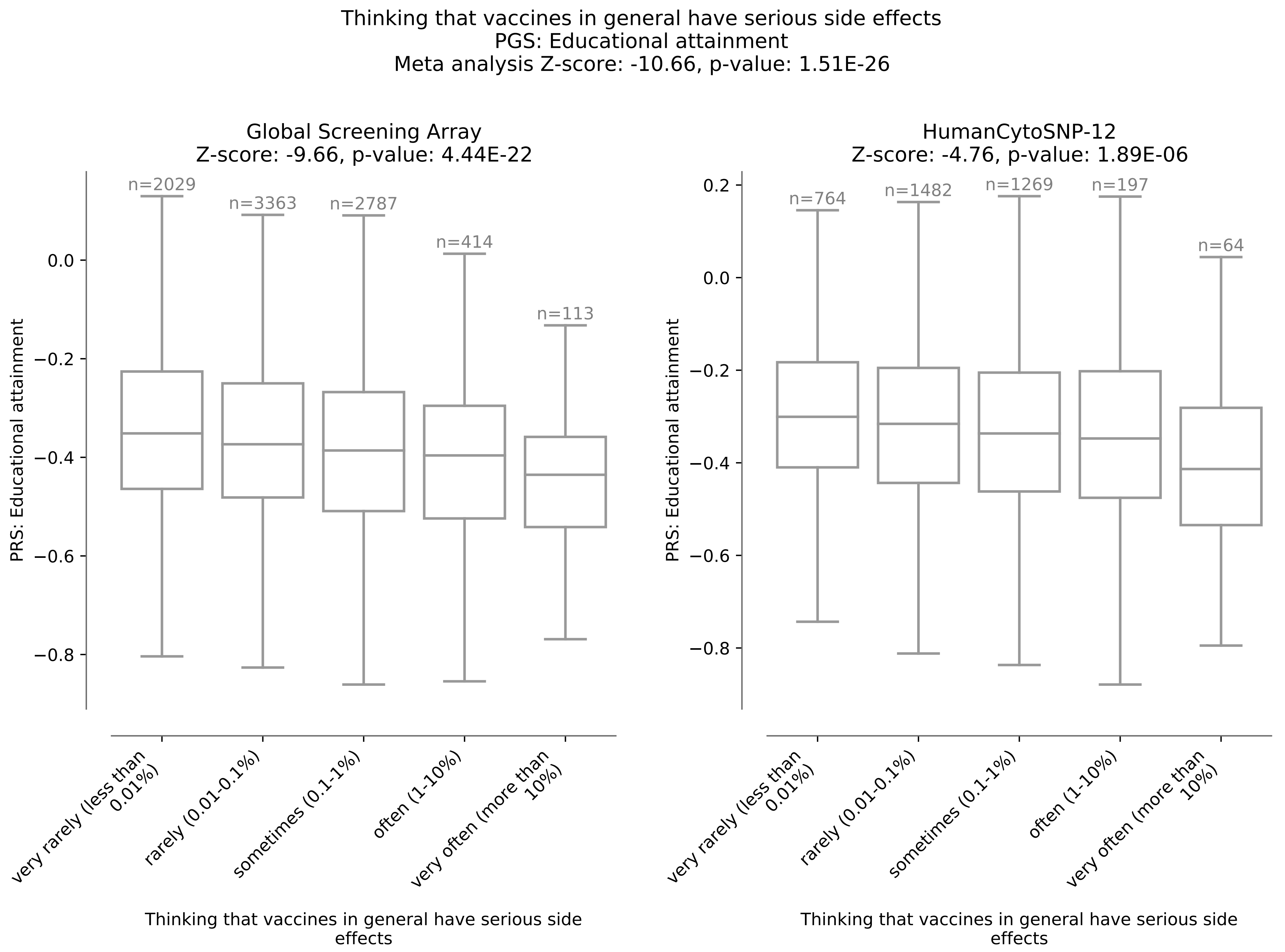
Thinking that vaccines in general have serious side effects
PGS: Educational attainment
Meta analysis Z-score: -10.66, p-value: 1.51E-26
Global Screening Array
Z-score: -9.66, p-value: 4.44E-22
n=2029
n=3363
n=2787
n=414
n=113
PRS: Educational attainment
0.0
−0.2
−0.4
−0.6
−0.8
very rarely (less than 0.01%)
rarely (0.01-0.1%)
sometimes (0.1-1%)
often (1-10%)
very often (more than 10%)
Thinking that vaccines in general have serious side effects
HumanCytoSNP-12
Z-score: -4.76, p-value: 1.89E-06
n=764
n=1482
n=1269
n=197
n=64
0.2
0.0
−0.2
−0.4
−0.6
−0.8
very rarely (less than 0.01%)
rarely (0.01-0.1%)
sometimes (0.1-1%)
often (1-10%)
very often (more than 10%)
Thinking that vaccines in general have serious side effects

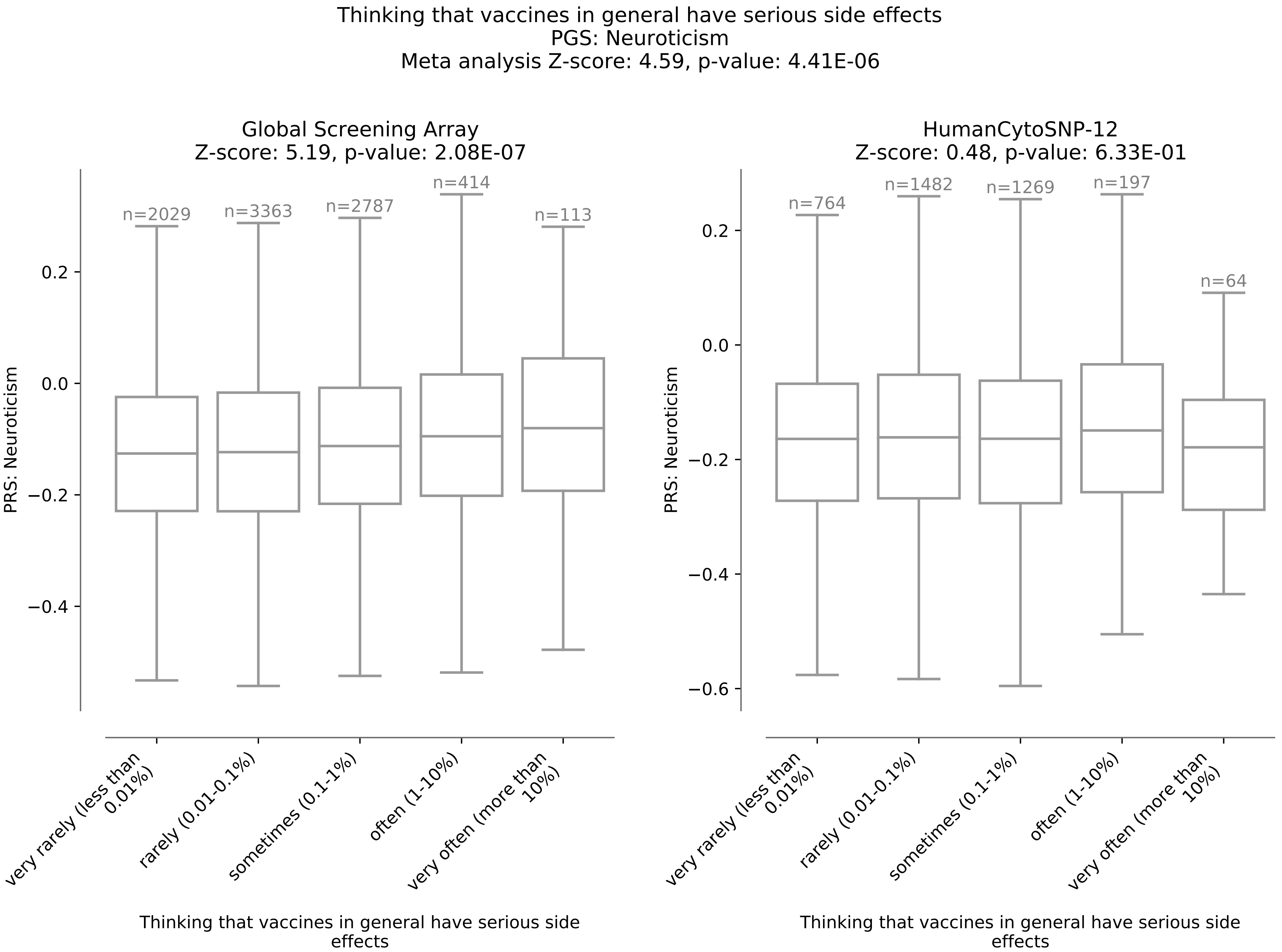
Thinking that vaccines in general have serious side effects
PGS: Neuroticism
Meta analysis Z-score: 4.59, p-value: 4.41E-06
Global Screening Array
Z-score: 5.19, p-value: 2.08E-07
HumanCytoSNP-12
Z-score: 0.48, p-value: 6.33E-01
PRS: Neuroticism
n=2029
n=3363
n=2787
n=414
n=113
n=764
n=1482
n=1269
n=197
n=64
0.2
0.0
−0.2
−0.4
−0.6
very rarely (less than 0.01%)
rarely (0.01-0.1%)
sometimes (0.1-1%)
often (1-10%)
very often (more than 10%)
Thinking that vaccines in general have serious side effects

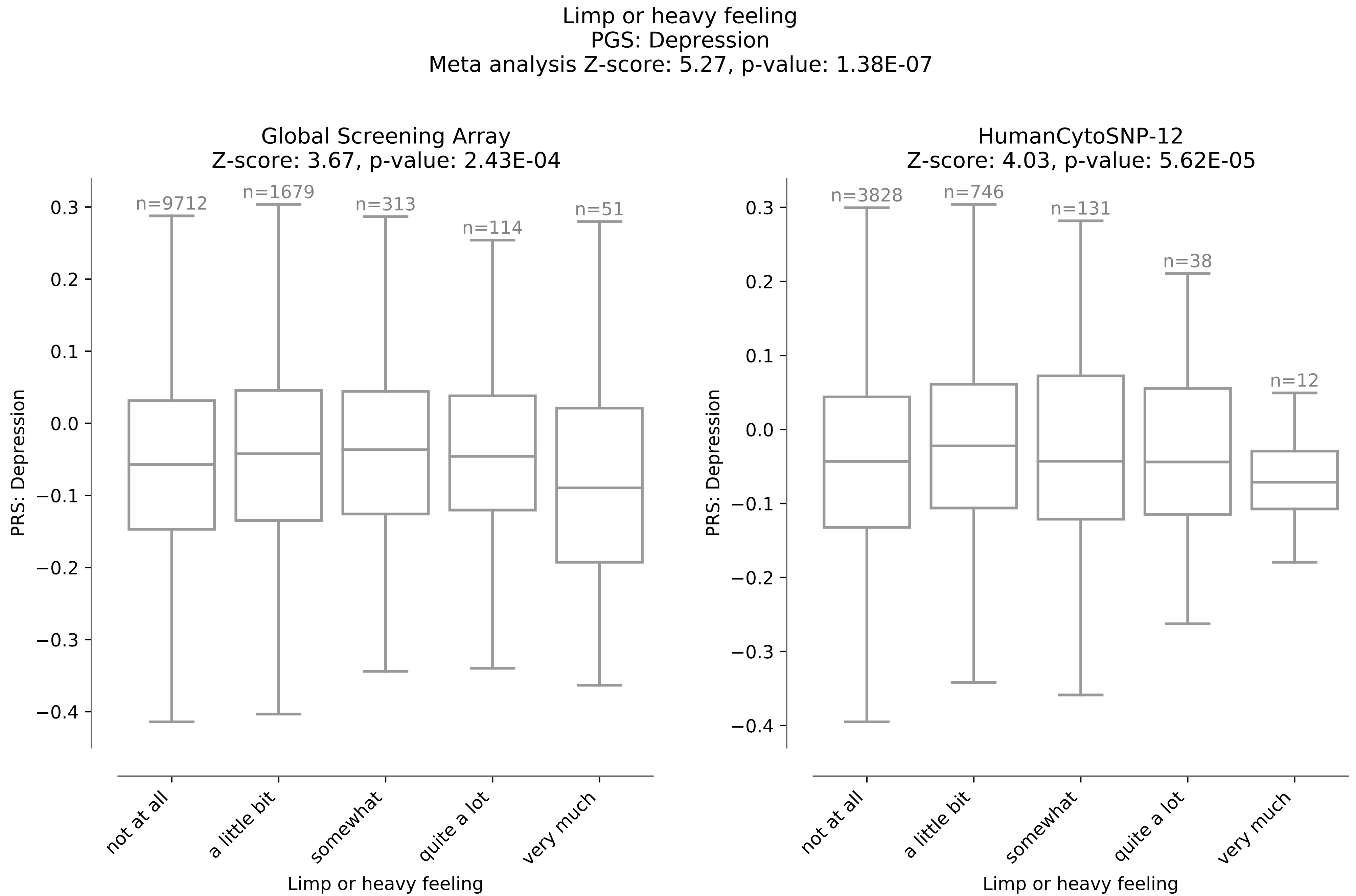

Limp or heavy feeling
PGS: Depression
Meta analysis Z-score: 5.27, p-value: 1.38E-07
Global Screening Array
Z-score: 3.67, p-value: 2.43E-04
n=9712
n=1679
n=313
n=114
n=51
PRS: Depression
0.3
0.2
0.1
0.0
−0.1
−0.2
−0.3
−0.4
not at all
a little bit
somewhat
quite a lot
very much
Limp or heavy feeling
HumanCytoSNP-12
Z-score: 4.03, p-value: 5.62E-05
n=3828
n=746
n=131
n=38
n=12
PRS: Depression
0.3
0.2
0.1
0.0
−0.1
−0.2
−0.3
−0.4
not at all
a little bit
somewhat
quite a lot
very much
Limp or heavy feeling

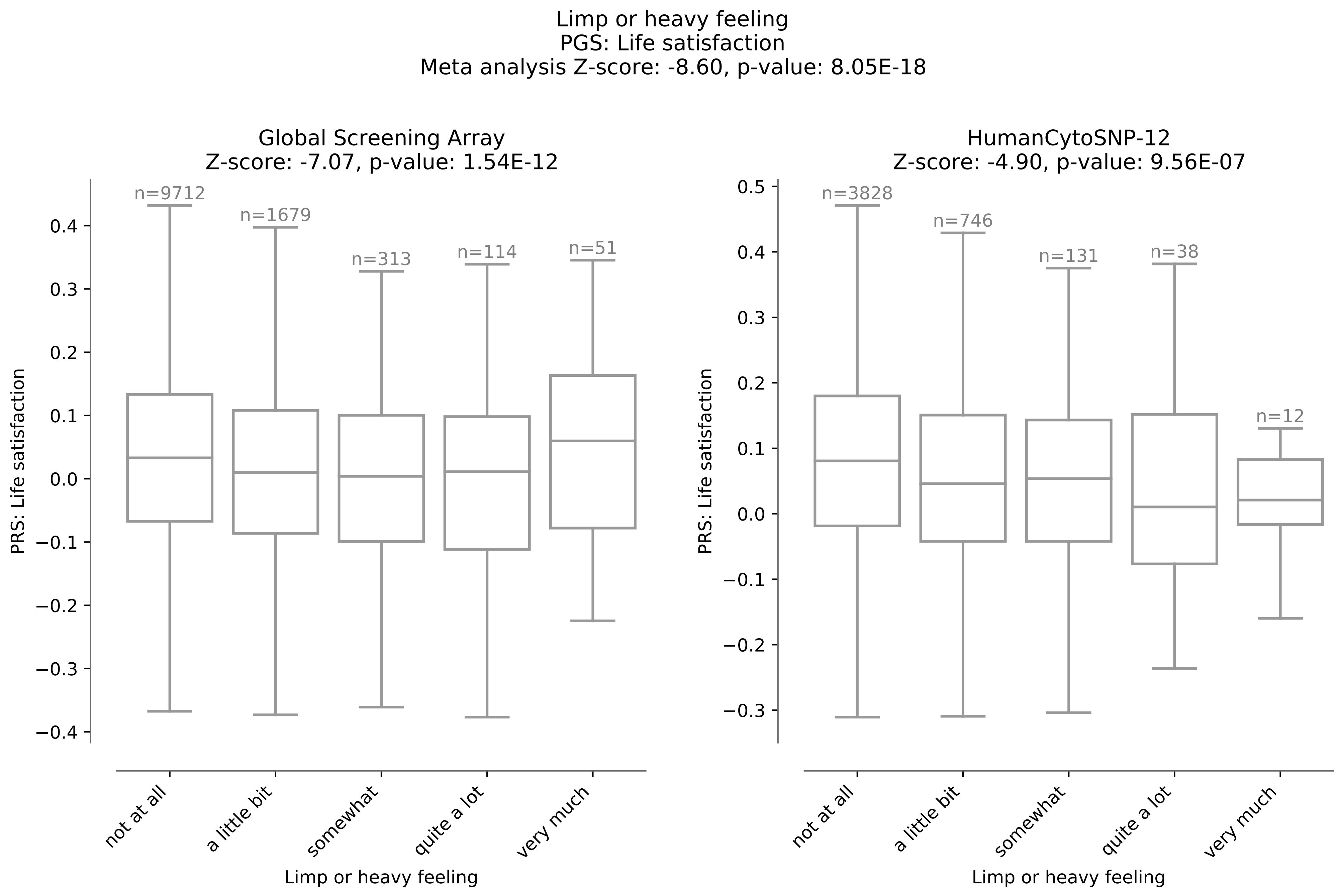
Limp or heavy feeling
PGS: Life satisfaction
Meta analysis Z-score: -8.60, p-value: 8.05E-18
Global Screening Array
Z-score: -7.07, p-value: 1.54E-12
n=9712
n=1679
n=313
n=114
n=51
PRS: Life satisfaction
not at all
a little bit
somewhat
quite a lot
very much
Limp or heavy feeling
HumanCytoSNP-12
Z-score: -4.90, p-value: 9.56E-07
n=3828
n=746
n=131
n=38
n=12
PRS: Life satisfaction
not at all
a little bit
somewhat
quite a lot
very much
Limp or heavy feeling

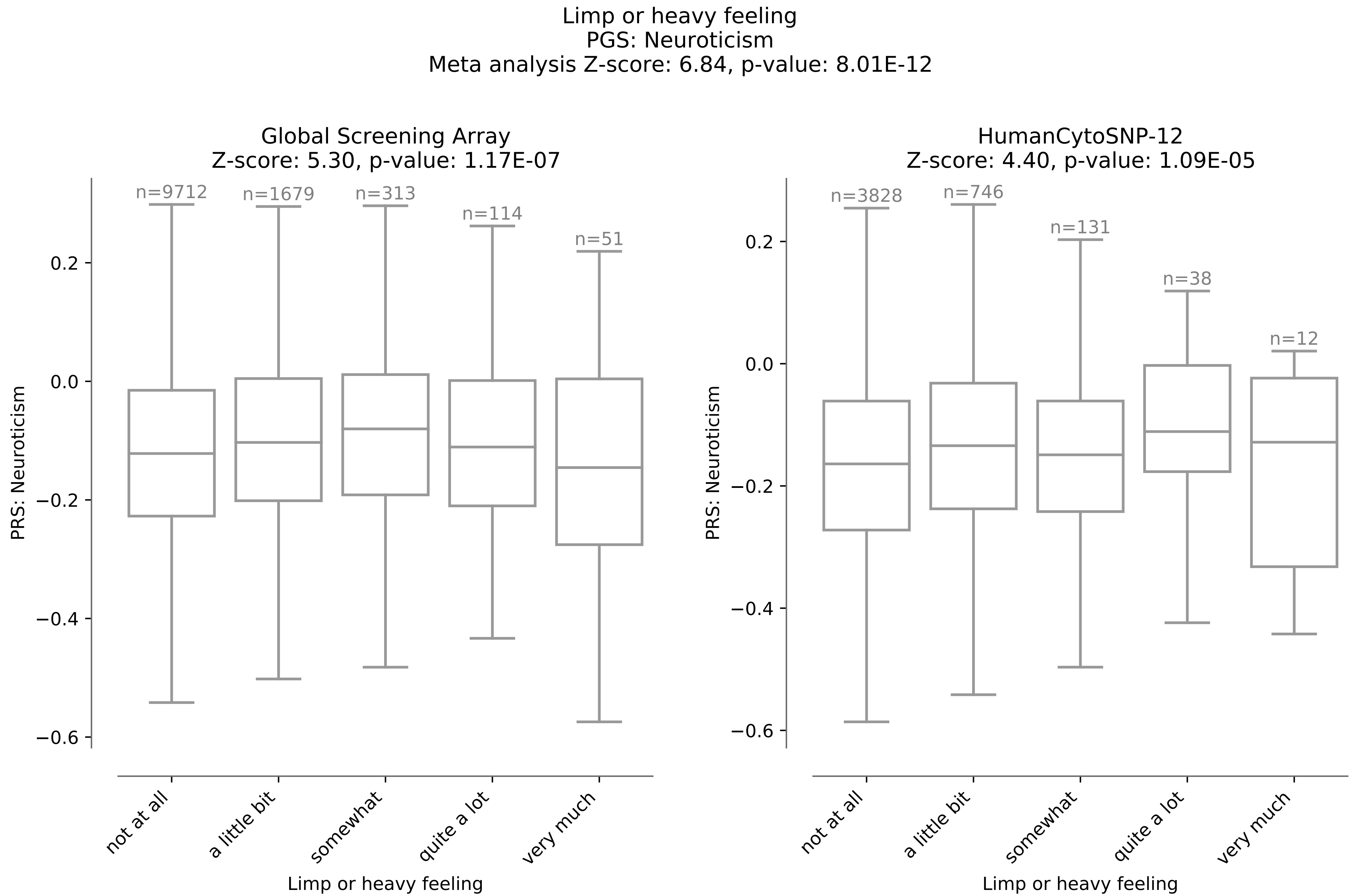

Limp or heavy feeling
PGS: Neuroticism
Meta analysis Z-score: 6.84, p-value: 8.01E-12
Global Screening Array
Z-score: 5.30, p-value: 1.17E-07
n=9712
n=1679
n=313
n=114
n=51
PRS: Neuroticism
0.2
0.0
−0.2
−0.4
−0.6
not at all
a little bit
somewhat
quite a lot
very much
Limp or heavy feeling
HumanCytoSNP-12
Z-score: 4.40, p-value: 1.09E-05
n=3828
n=746
n=131
n=38
n=12
PRS: Neuroticism
0.2
0.0
−0.2
−0.4
−0.6
not at all
a little bit
somewhat
quite a lot
very much
Limp or heavy feeling

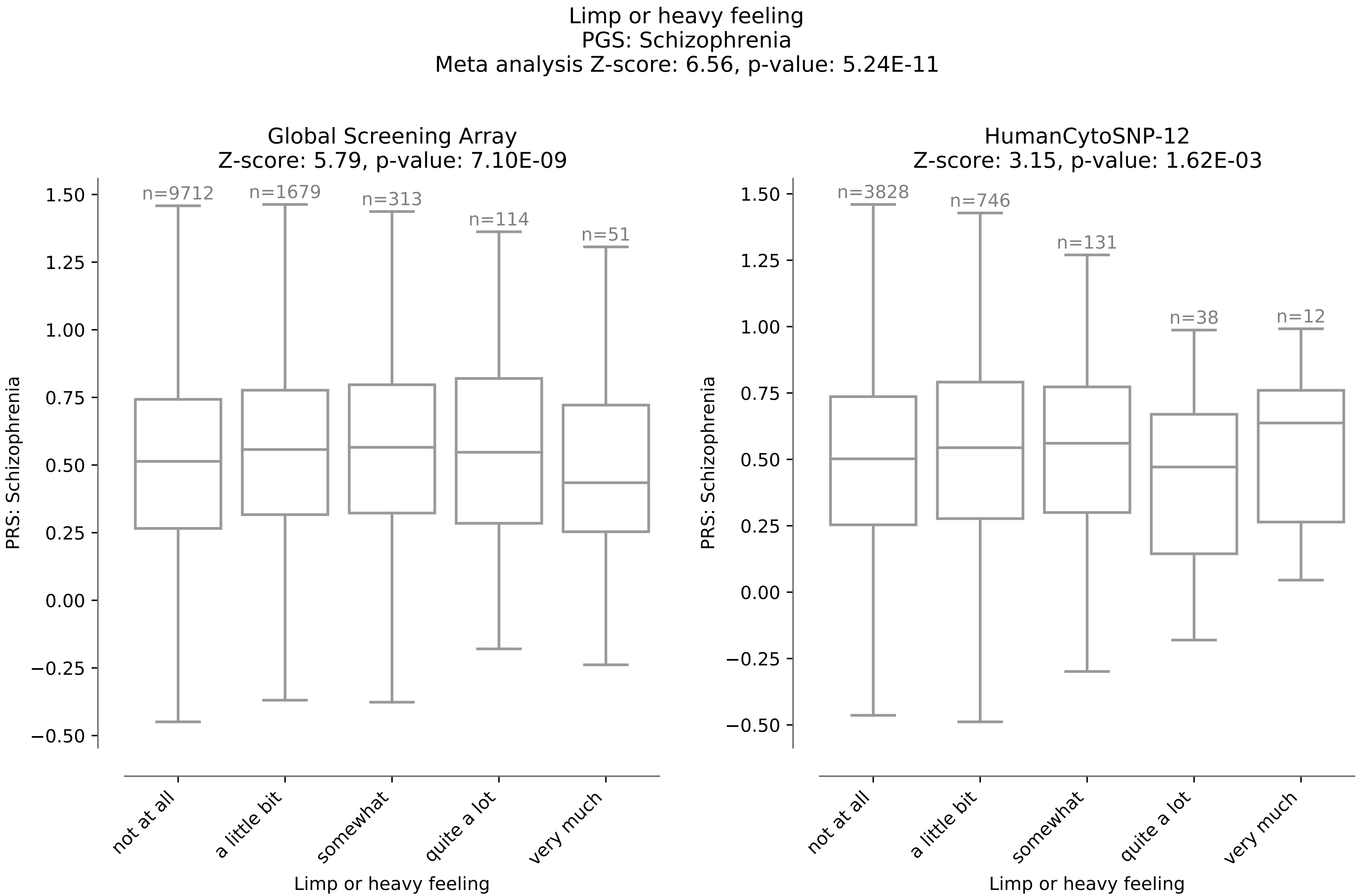

Limp or heavy feeling
PGS: Schizophrenia
Meta analysis Z-score: 6.56, p-value: 5.24E-11
Global Screening Array
Z-score: 5.79, p-value: 7.10E-09
HumanCytoSNP-12
Z-score: 3.15, p-value: 1.62E-03
PRS: Schizophrenia
n=9712
n=1679
n=313
n=114
n=51
n=3828
n=746
n=131
n=38
n=12
1.50
1.25
1.00
0.75
0.50
0.25
0.00
−0.25
−0.50
not at all
a little bit
somewhat
quite a lot
very much
Limp or heavy feeling

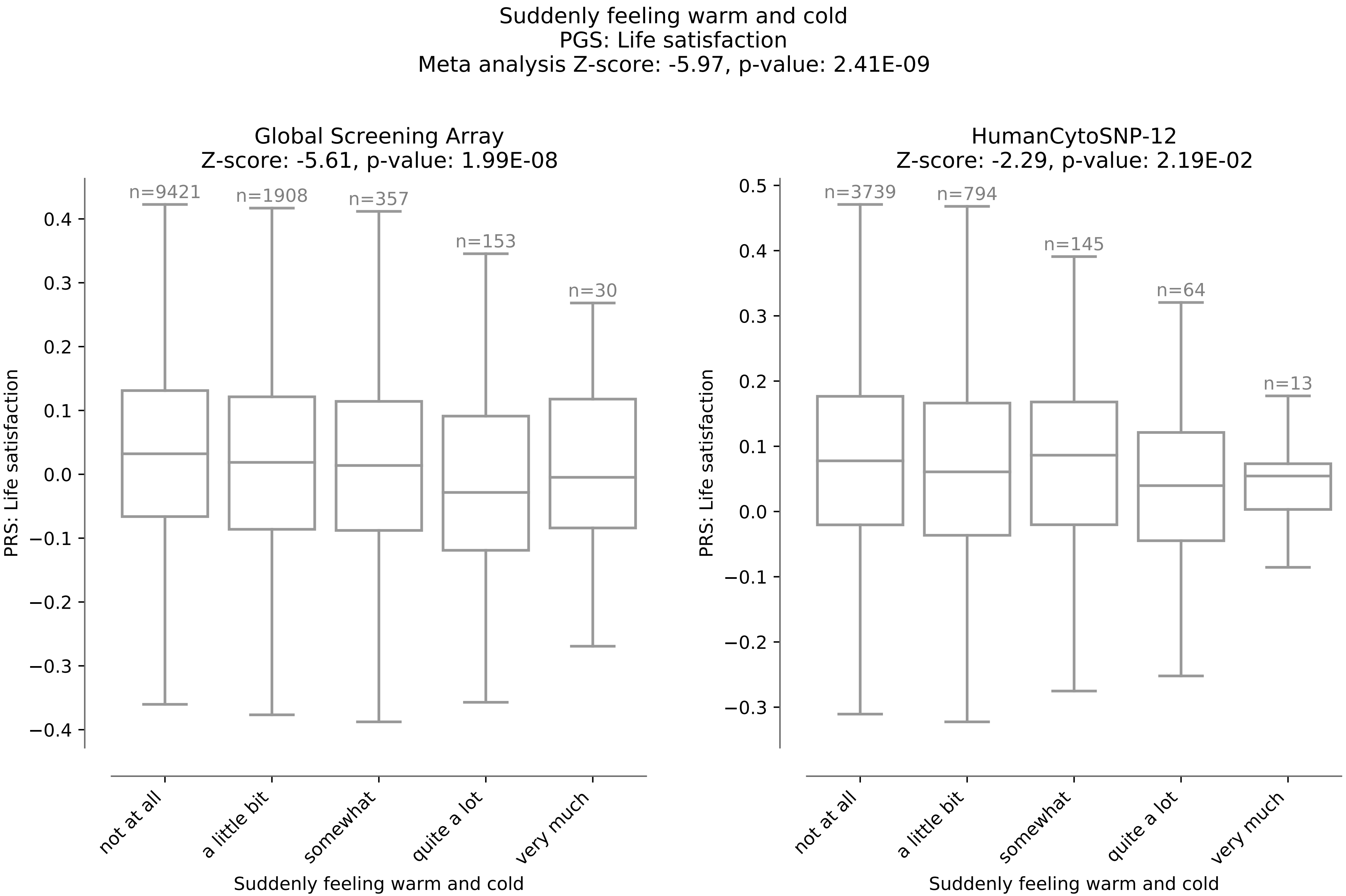
Suddenly feeling warm and cold
PGS: Life satisfaction
Meta analysis Z-score: -5.97, p-value: 2.41E-09
Global Screening Array
Z-score: -5.61, p-value: 1.99E-08
n=9421
n=1908
n=357
n=153
n=30
PRS: Life satisfaction
not at all
a little bit
somewhat
quite a lot
very much
Suddenly feeling warm and cold
HumanCytoSNP-12
Z-score: -2.29, p-value: 2.19E-02
n=3739
n=794
n=145
n=64
n=13
PRS: Life satisfaction
not at all
a little bit
somewhat
quite a lot
very much
Suddenly feeling warm and cold

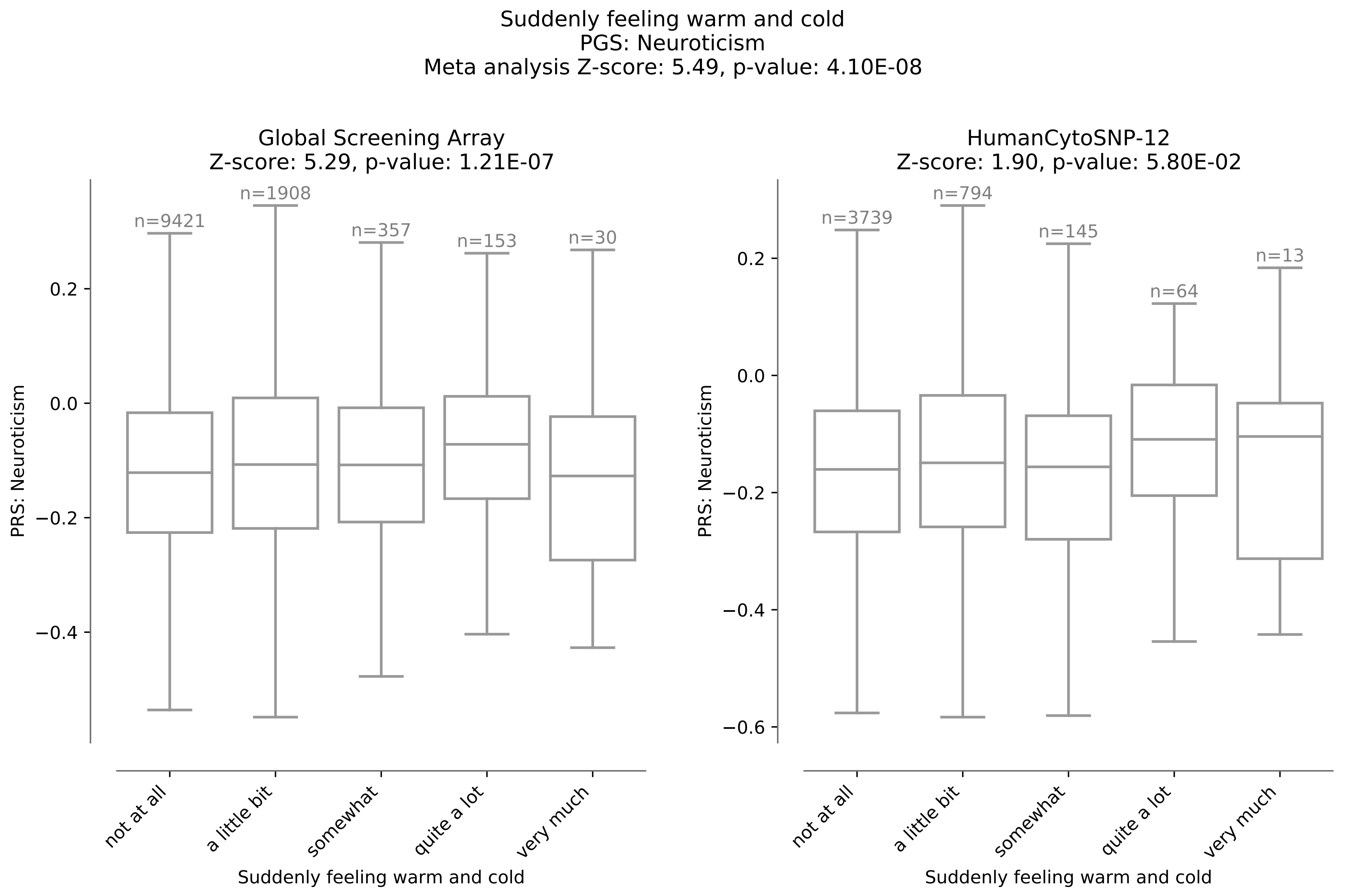
Suddenly feeling warm and cold
PGS: Neuroticism
Meta analysis Z-score: 5.49, p-value: 4.10E-08
Global Screening Array
Z-score: 5.29, p-value: 1.21E-07
HumanCytoSNP-12
Z-score: 1.90, p-value: 5.80E-02
PRS: Neuroticism
n=9421
n=1908
n=357
n=153
n=30
n=3739
n=794
n=145
n=64
n=13
0.2
0.0
−0.2
−0.4
−0.6
not at all
a little bit
somewhat
quite a lot
very much
Suddenly feeling warm and cold

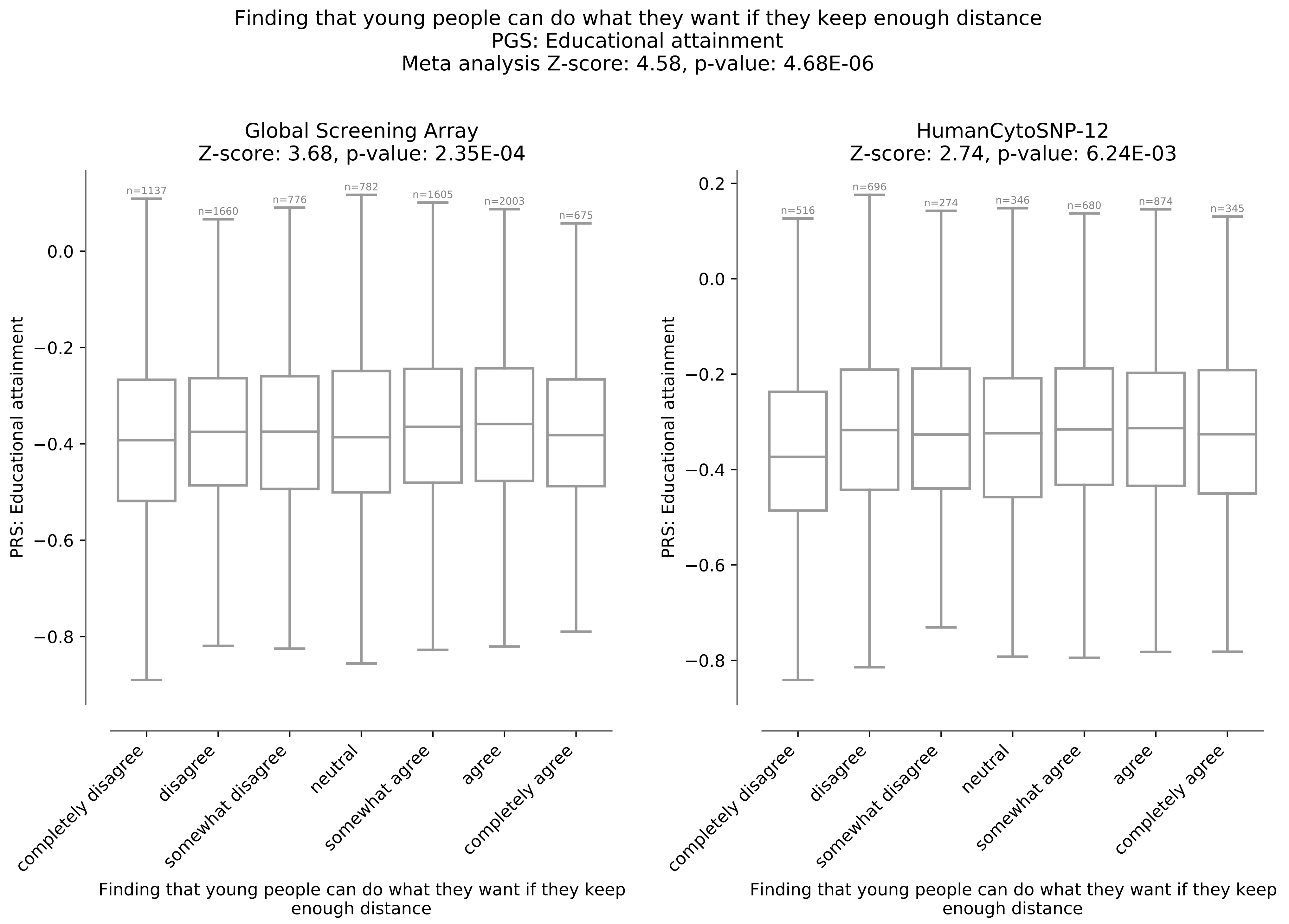

Finding that young people can do what they want if they keep enough distance
PGS: Educational attainment
Meta analysis Z-score: 4.58, p-value: 4.68E-06
Global Screening Array
Z-score: 3.68, p-value: 2.35E-04
HumanCytoSNP-12
Z-score: 2.74, p-value: 6.24E-03
PRS: Educational attainment
n=1137
n=1660
n=776
n=782
n=1605
n=2003
n=675
n=516
n=696
n=274
n=346
n=680
n=874
n=345
completely disagree
disagree
somewhat disagree
neutral
somewhat agree
agree
completely agree
Finding that young people can do what they want if they keep enough distance

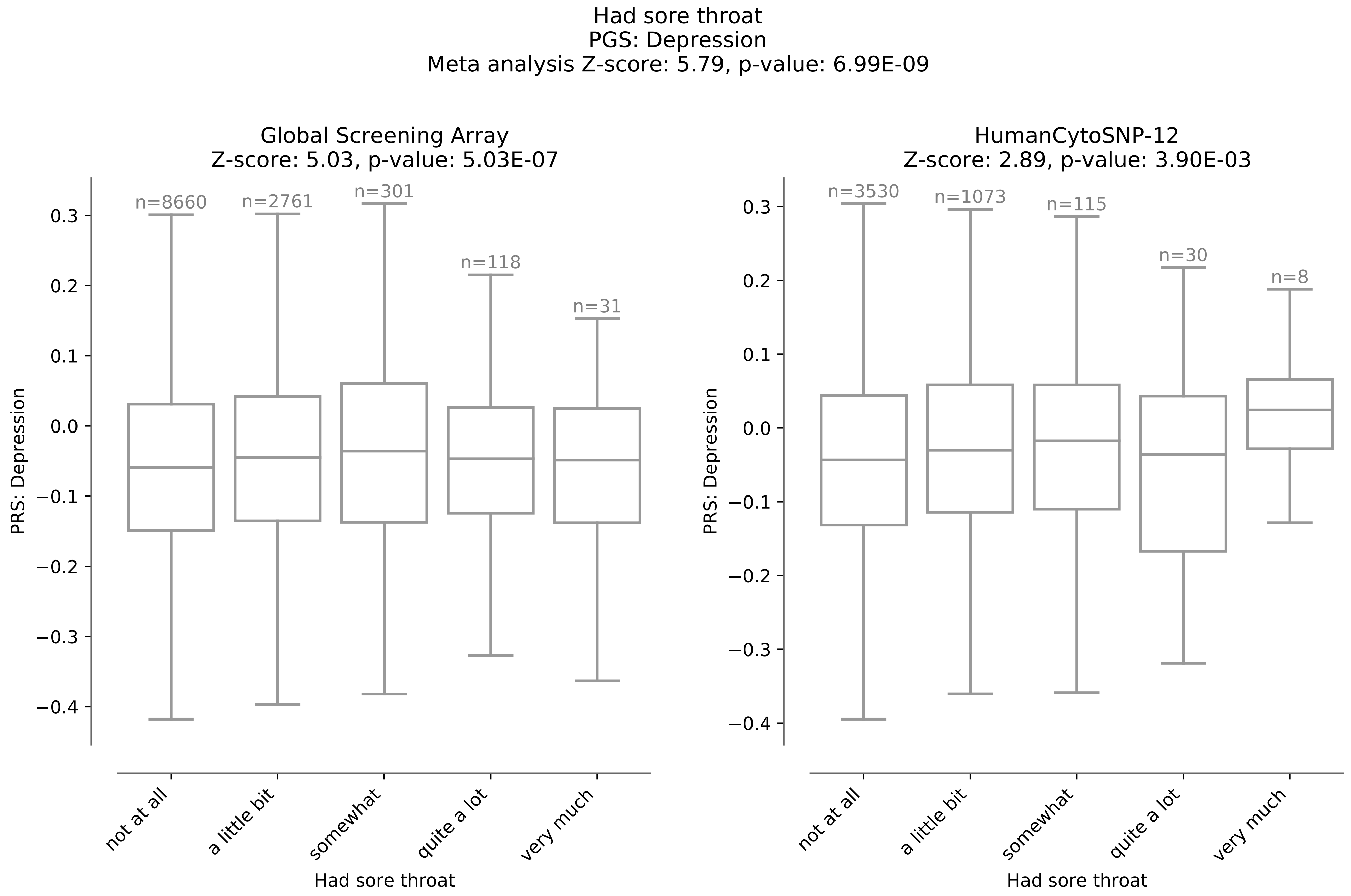

Had sore throat
PGS: Depression
Meta analysis Z-score: 5.79, p-value: 6.99E-09
Global Screening Array
Z-score: 5.03, p-value: 5.03E-07
n=8660
n=2761
n=301
n=118
n=31
PRS: Depression
not at all
a little bit
somewhat
quite a lot
very much
Had sore throat
HumanCytoSNP-12
Z-score: 2.89, p-value: 3.90E-03
n=3530
n=1073
n=115
n=30
n=8
PRS: Depression
not at all
a little bit
somewhat
quite a lot
very much
Had sore throat

Had sore throat
PGS: General risk tolerance
Meta analysis Z-score: 4.45, p-value: 8.53E-06

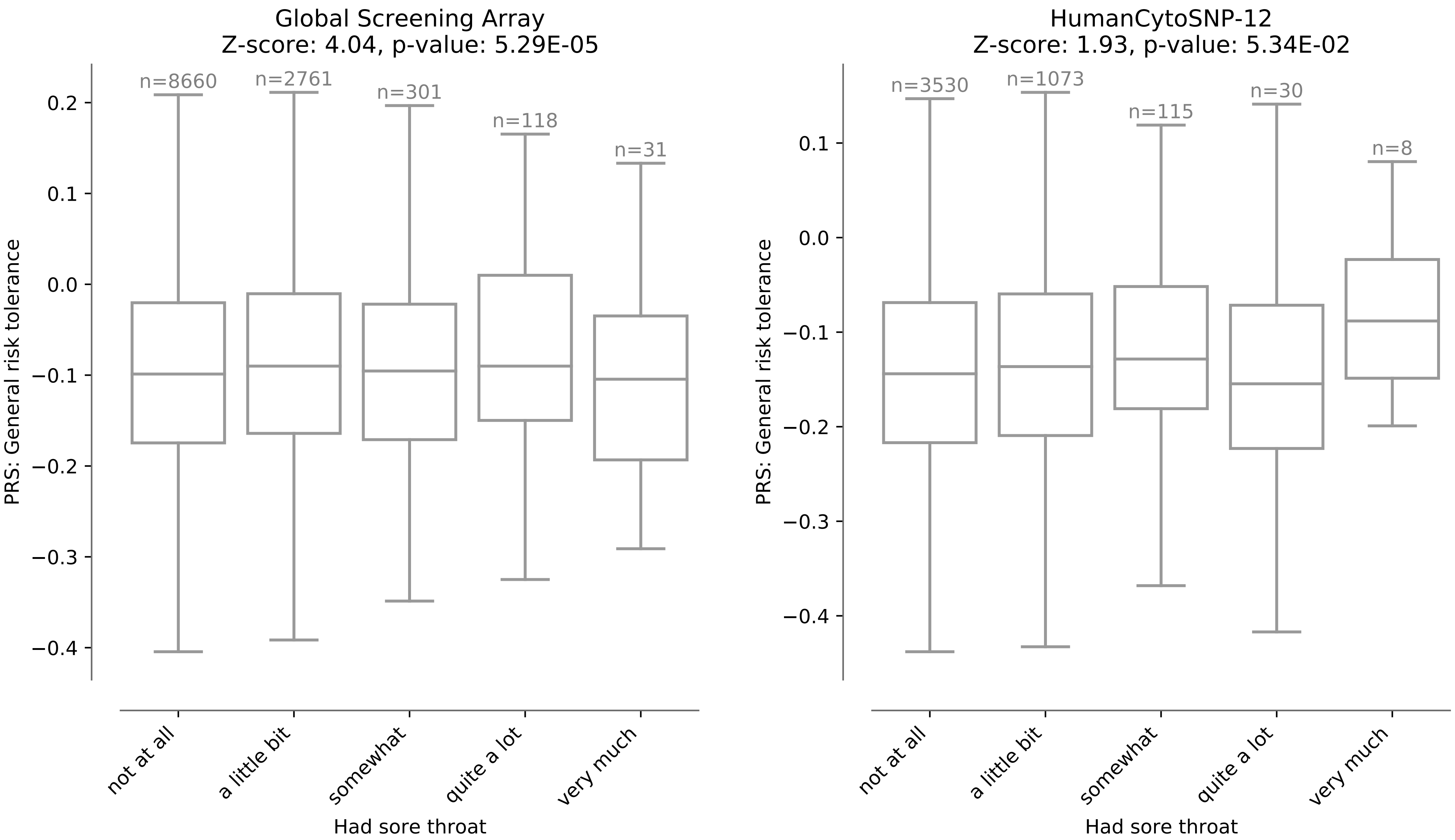

Had sore throat
PGS: Life satisfaction
Meta analysis Z-score: -5.64, p-value: 1.66E-08

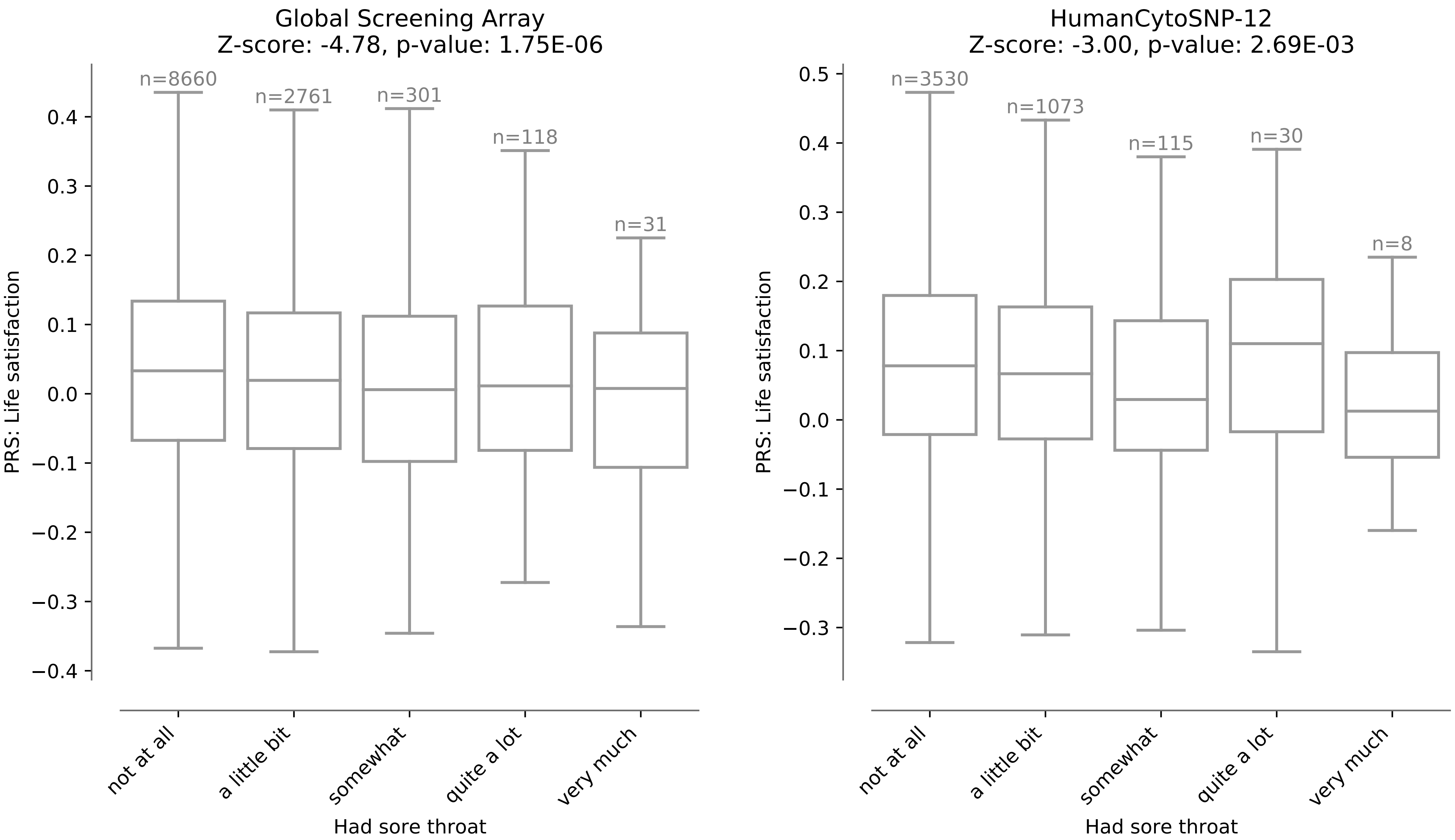

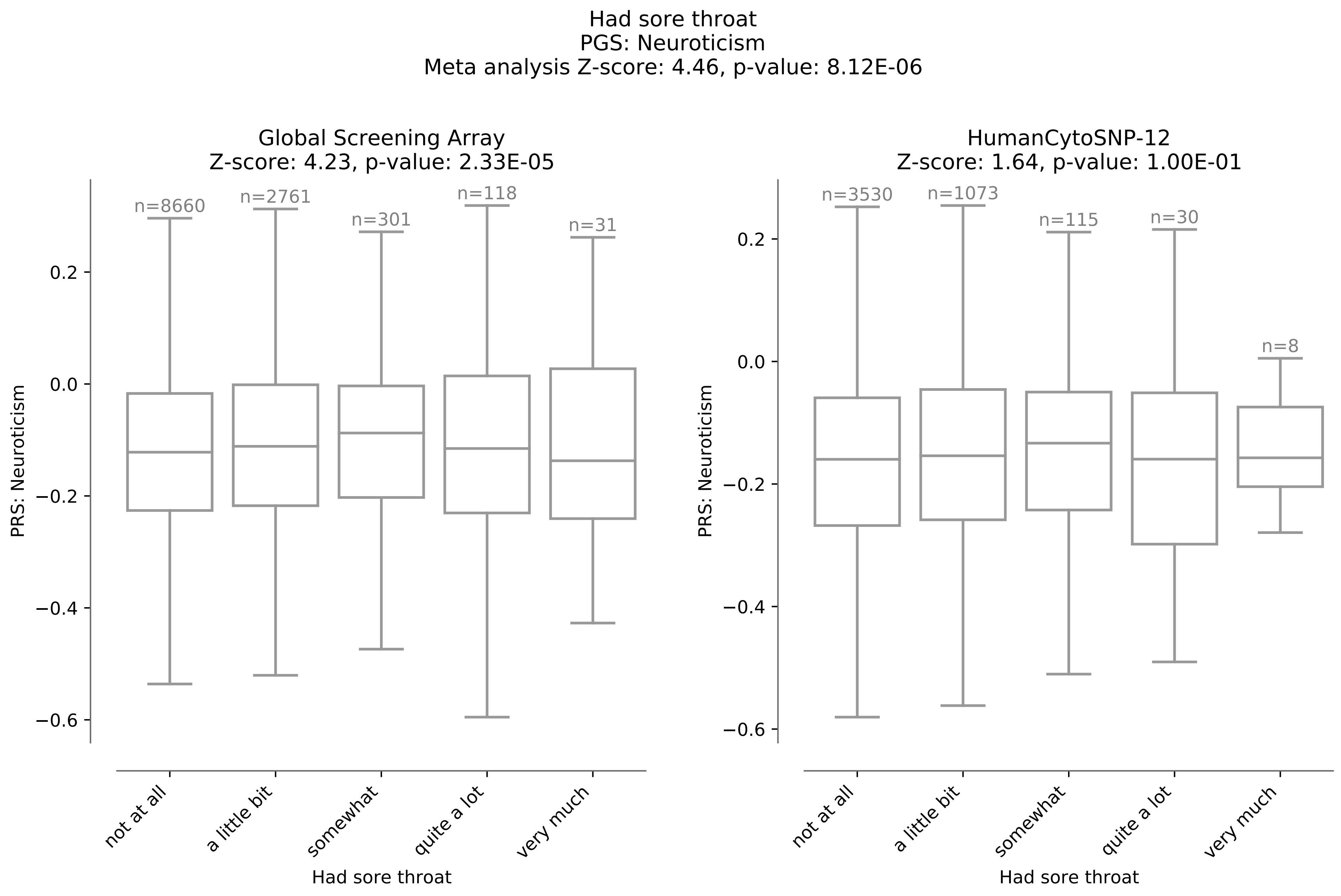

Had sore throat
PGS: Neuroticism
Meta analysis Z-score: 4.46, p-value: 8.12E-06
Global Screening Array
Z-score: 4.23, p-value: 2.33E-05
n=8660
n=2761
n=301
n=118
n=31
0.2
0.0
−0.2
−0.4
−0.6
PRS: Neuroticism
not at all
a little bit
somewhat
quite a lot
very much
Had sore throat
HumanCytoSNP-12
Z-score: 1.64, p-value: 1.00E-01
n=3530
n=1073
n=115
n=30
n=8
0.2
0.0
−0.2
−0.4
−0.6
PRS: Neuroticism
not at all
a little bit
somewhat
quite a lot
very much
Had sore throat

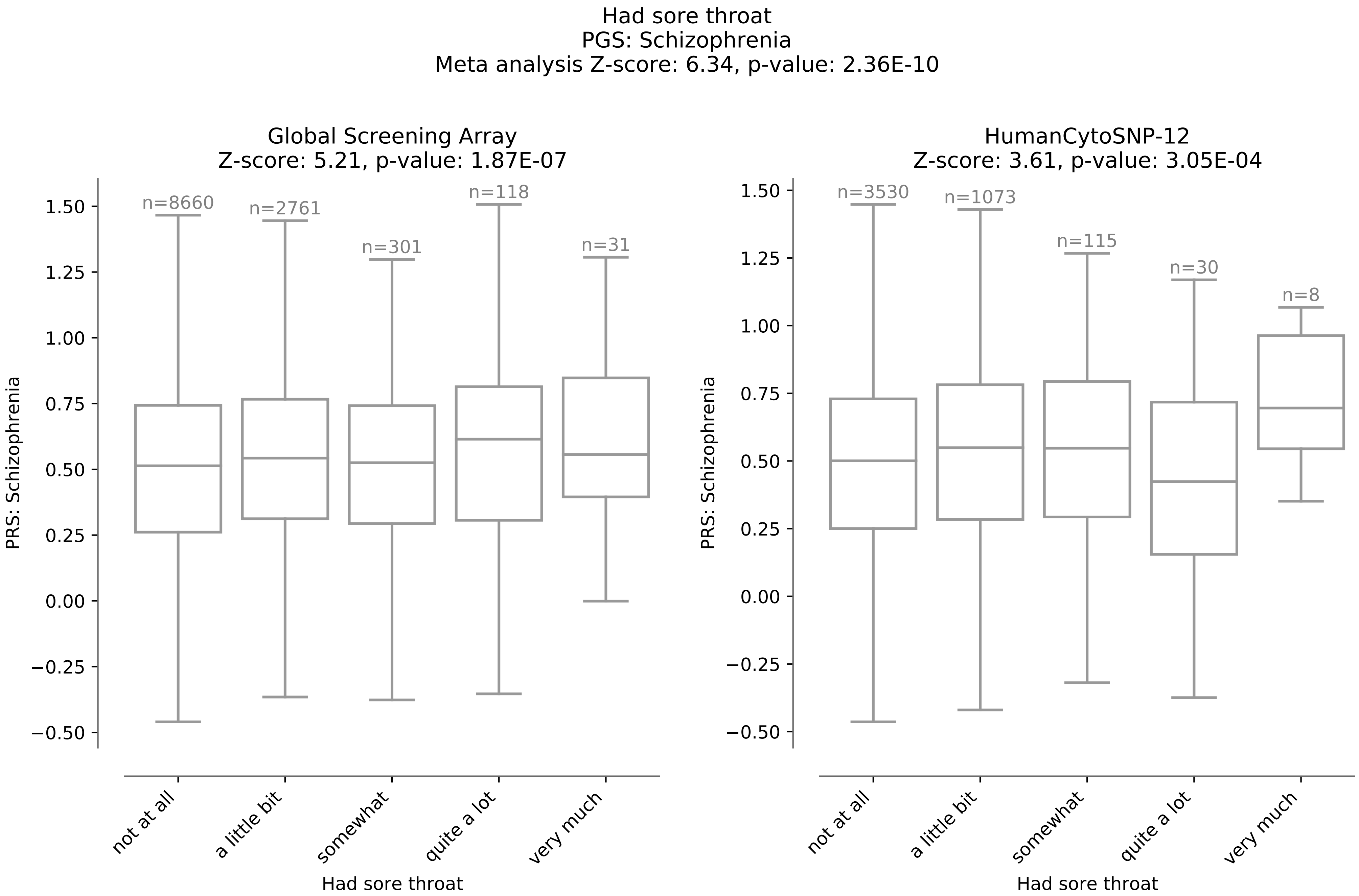
Had sore throat
PGS: Schizophrenia
Meta analysis Z-score: 6.34, p-value: 2.36E-10
Global Screening Array
Z-score: 5.21, p-value: 1.87E-07
HumanCytoSNP-12
Z-score: 3.61, p-value: 3.05E-04
PRS: Schizophrenia
n=8660
n=2761
n=301
n=118
n=31
n=3530
n=1073
n=115
n=30
n=8
1.50
1.25
1.00
0.75
0.50
0.25
0.00
−0.25
−0.50
not at all
a little bit
somewhat
quite a lot
very much
Had sore throat

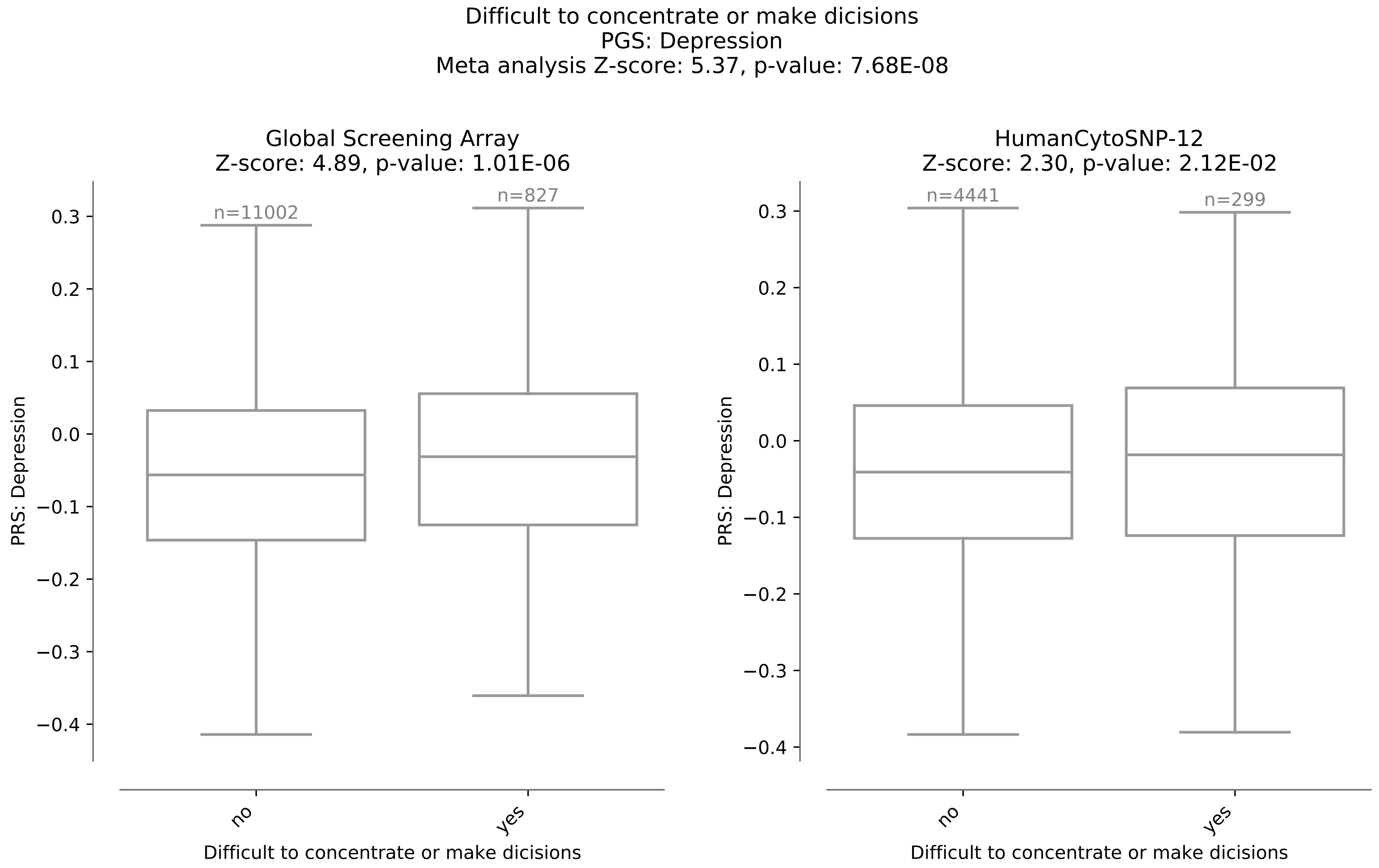
Difficult to concentrate or make dicisions
PGS: Depression
Meta analysis Z-score: 5.37, p-value: 7.68E-08
Global Screening Array
Z-score: 4.89, p-value: 1.01E-06
n=11002
n=827
PRS: Depression
0.3
0.2
0.1
0.0
−0.1
−0.2
−0.3
−0.4
no
yes
Difficult to concentrate or make dicisions
HumanCytoSNP-12
Z-score: 2.30, p-value: 2.12E-02
n=4441
n=299
PRS: Depression
0.3
0.2
0.1
0.0
−0.1
−0.2
−0.3
−0.4
no
yes
Difficult to concentrate or make dicisions

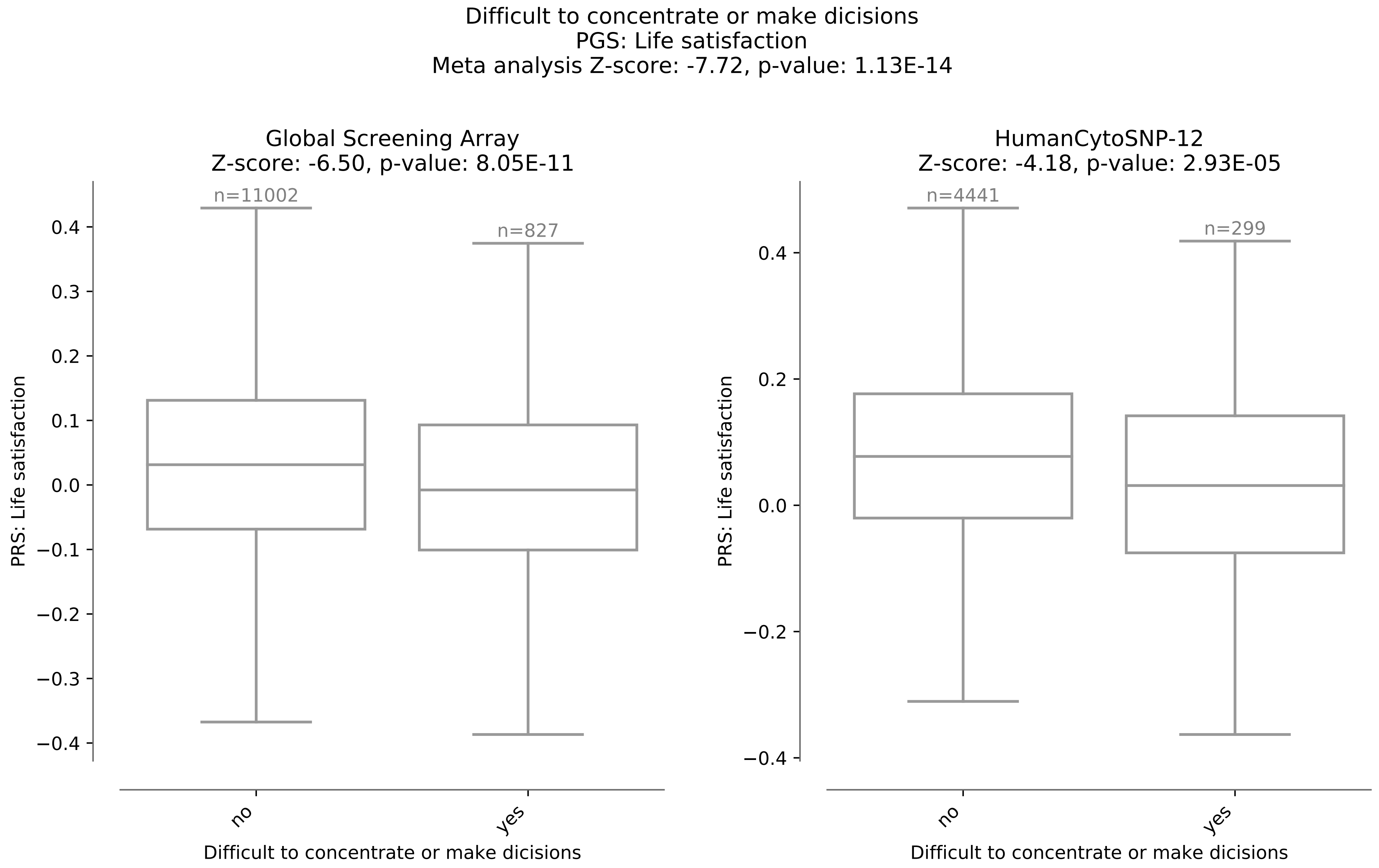

Difficult to concentrate or make dicisions
PGS: Life satisfaction
Meta analysis Z-score: -7.72, p-value: 1.13E-14
Global Screening Array
Z-score: -6.50, p-value: 8.05E-11
n=11002
n=827
PRS: Life satisfaction
0.4
0.3
0.2
0.1
0.0
−0.1
−0.2
−0.3
−0.4
no
yes
Difficult to concentrate or make dicisions
HumanCytoSNP-12
Z-score: -4.18, p-value: 2.93E-05
n=4441
n=299
PRS: Life satisfaction
0.4
0.2
0.0
−0.2
−0.4
no
yes
Difficult to concentrate or make dicisions

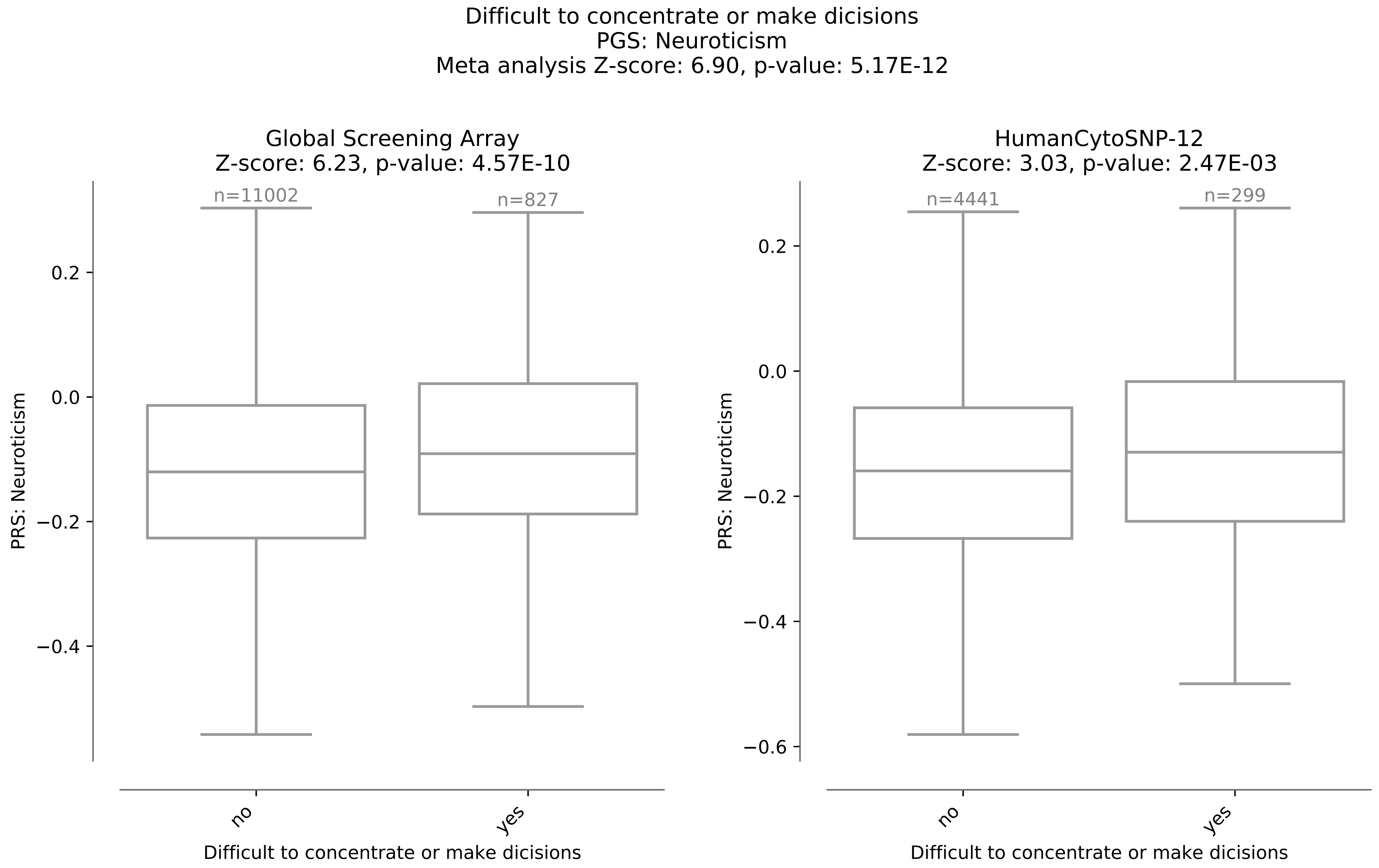
Difficult to concentrate or make dicisions
PGS: Neuroticism
Meta analysis Z-score: 6.90, p-value: 5.17E-12
Global Screening Array
Z-score: 6.23, p-value: 4.57E-10
n=11002
n=827
PRS: Neuroticism
0.2
0.0
−0.2
−0.4
no
yes
Difficult to concentrate or make dicisions
HumanCytoSNP-12
Z-score: 3.03, p-value: 2.47E-03
n=4441
n=299
PRS: Neuroticism
0.2
0.0
−0.2
−0.4
−0.6
no
yes
Difficult to concentrate or make dicisions

Had shortness of breath
PGS: BMI
Meta analysis Z-score: 4.84, p-value: 1.33E-06

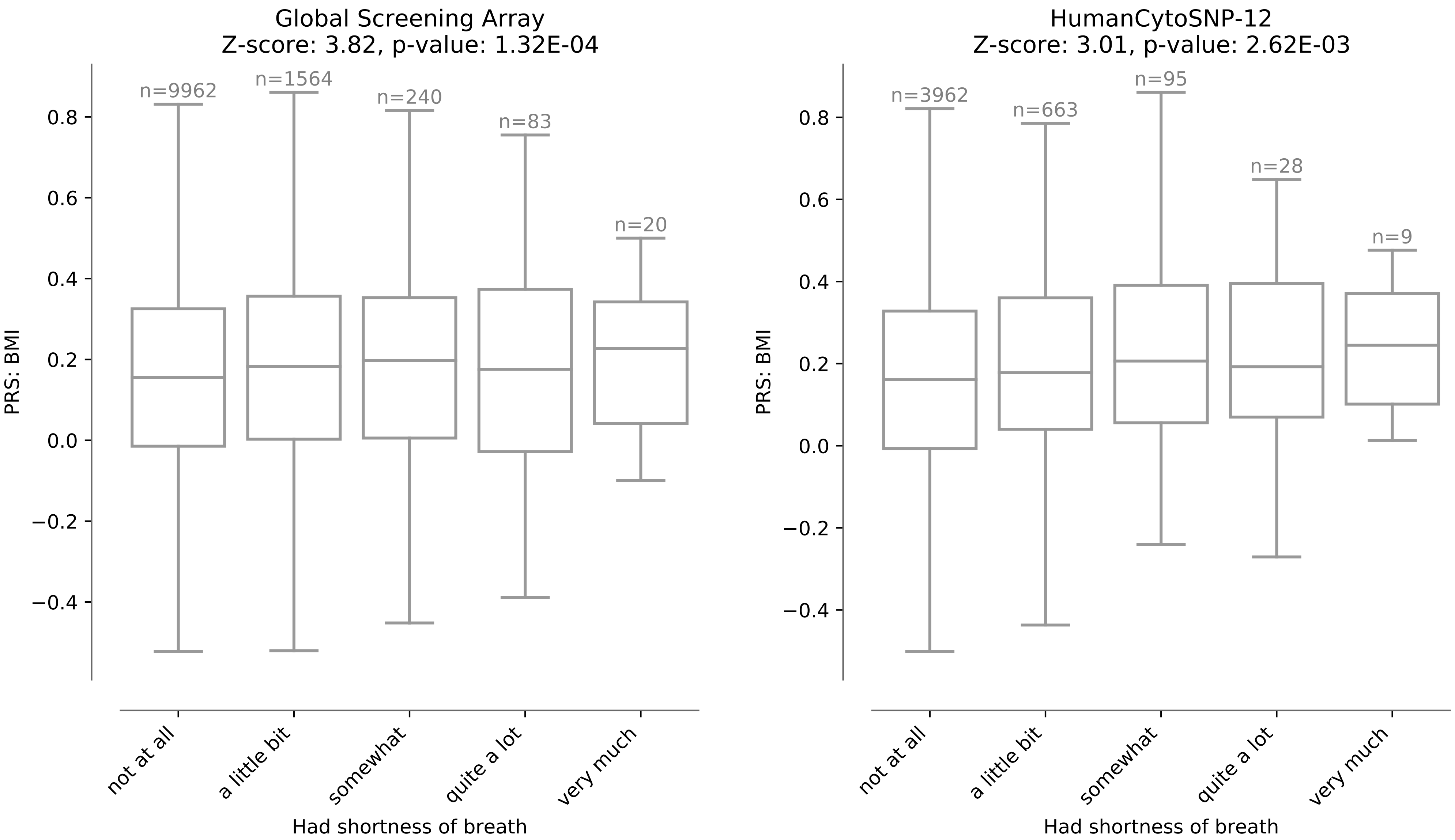

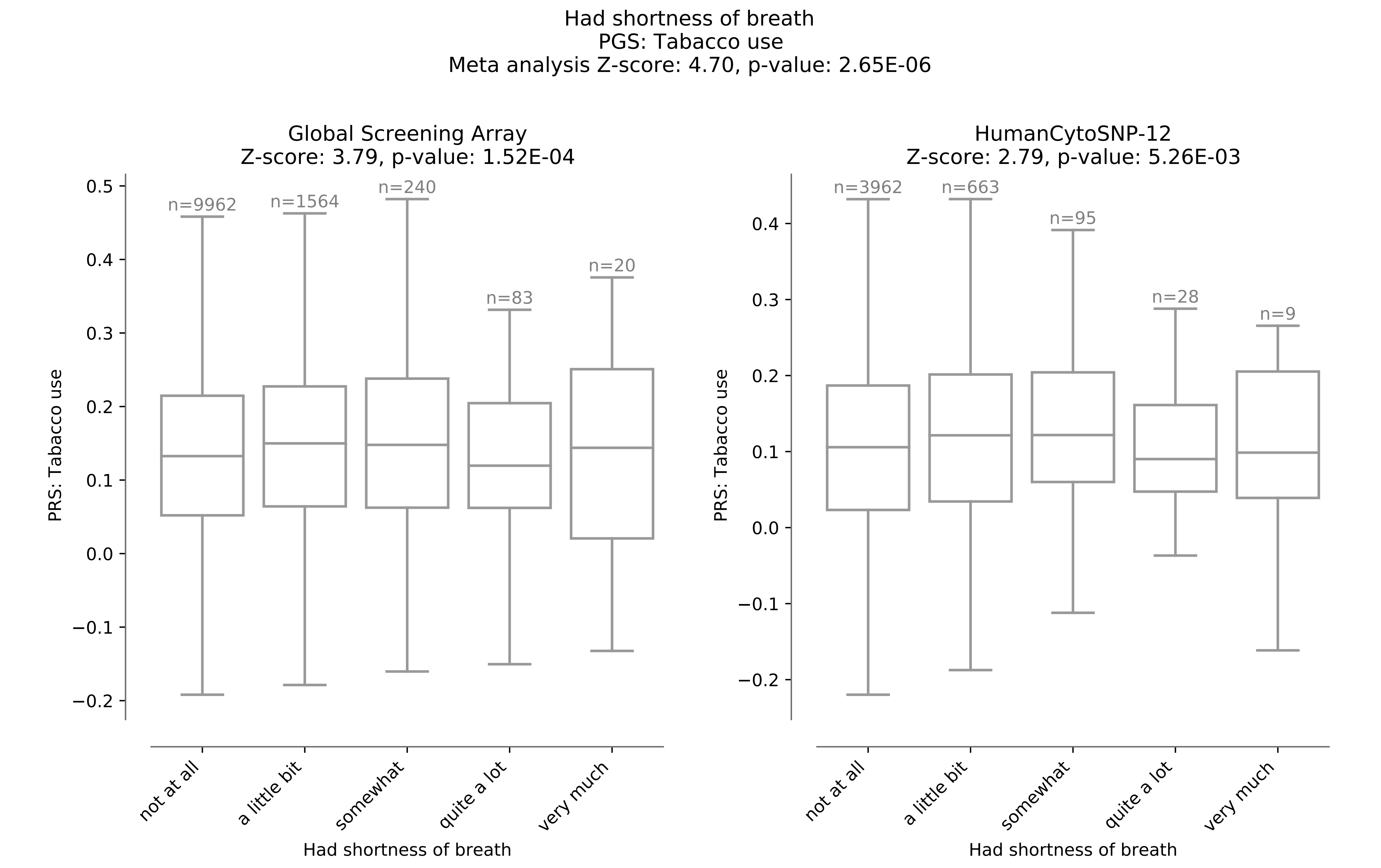
Had shortness of breath
PGS: Tabacco use
Meta analysis Z-score: 4.70, p-value: 2.65E-06
Global Screening Array
Z-score: 3.79, p-value: 1.52E-04
HumanCytoSNP-12
Z-score: 2.79, p-value: 5.26E-03
PRS: Tabacco use
n=9962
n=1564
n=240
n=83
n=20
n=3962
n=663
n=95
n=28
n=9
0.5
0.4
0.3
0.2
0.1
0.0
−0.1
−0.2
not at all
a little bit
somewhat
quite a lot
very much
Had shortness of breath

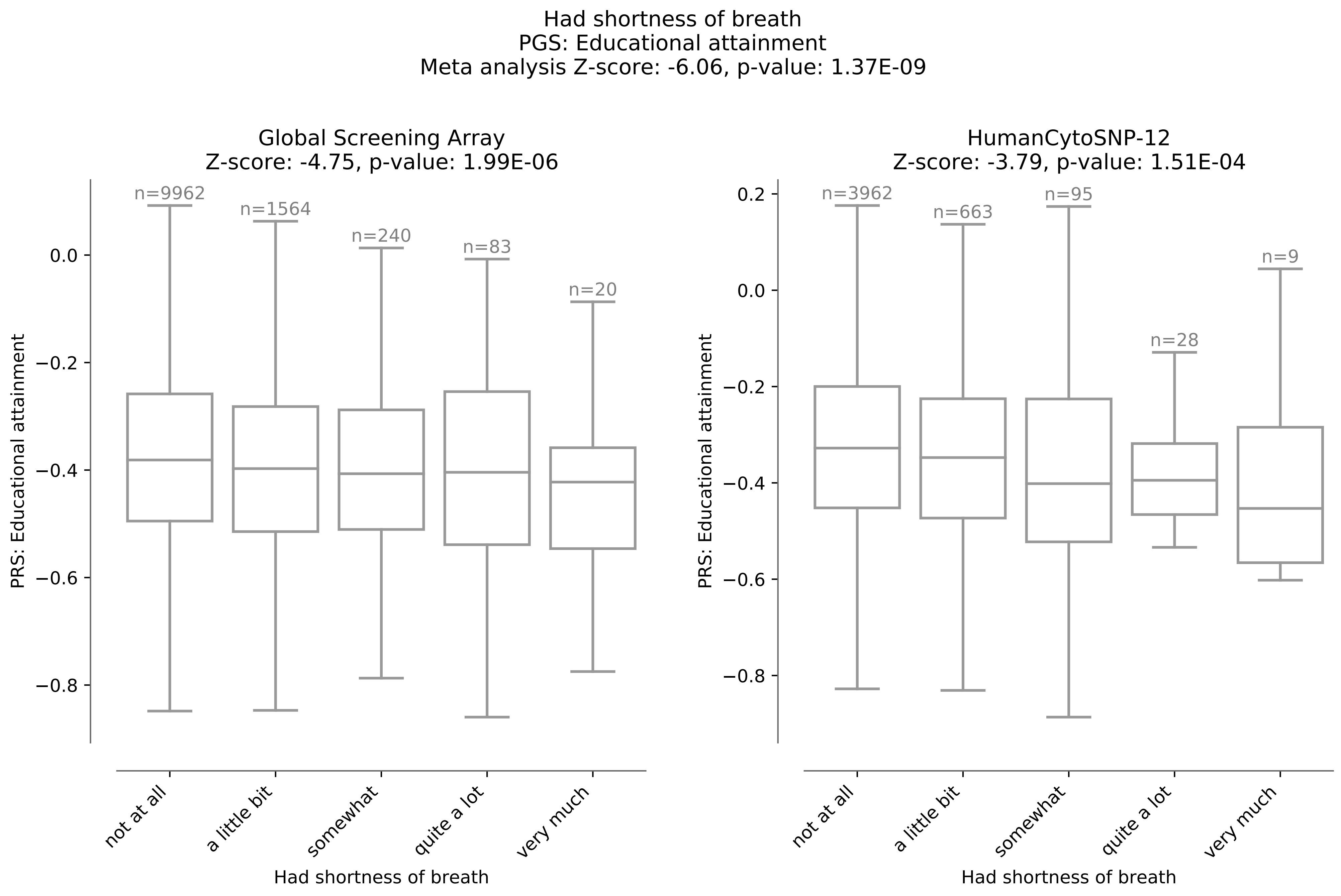

Had shortness of breath
PGS: Educational attainment
Meta analysis Z-score: -6.06, p-value: 1.37E-09
Global Screening Array
Z-score: -4.75, p-value: 1.99E-06
HumanCytoSNP-12
Z-score: -3.79, p-value: 1.51E-04
PRS: Educational attainment
n=9962
n=1564
n=240
n=83
n=20
n=3962
n=663
n=95
n=28
n=9
0.2
0.0
−0.2
−0.4
−0.6
−0.8
not at all
a little bit
somewhat
quite a lot
very much
Had shortness of breath

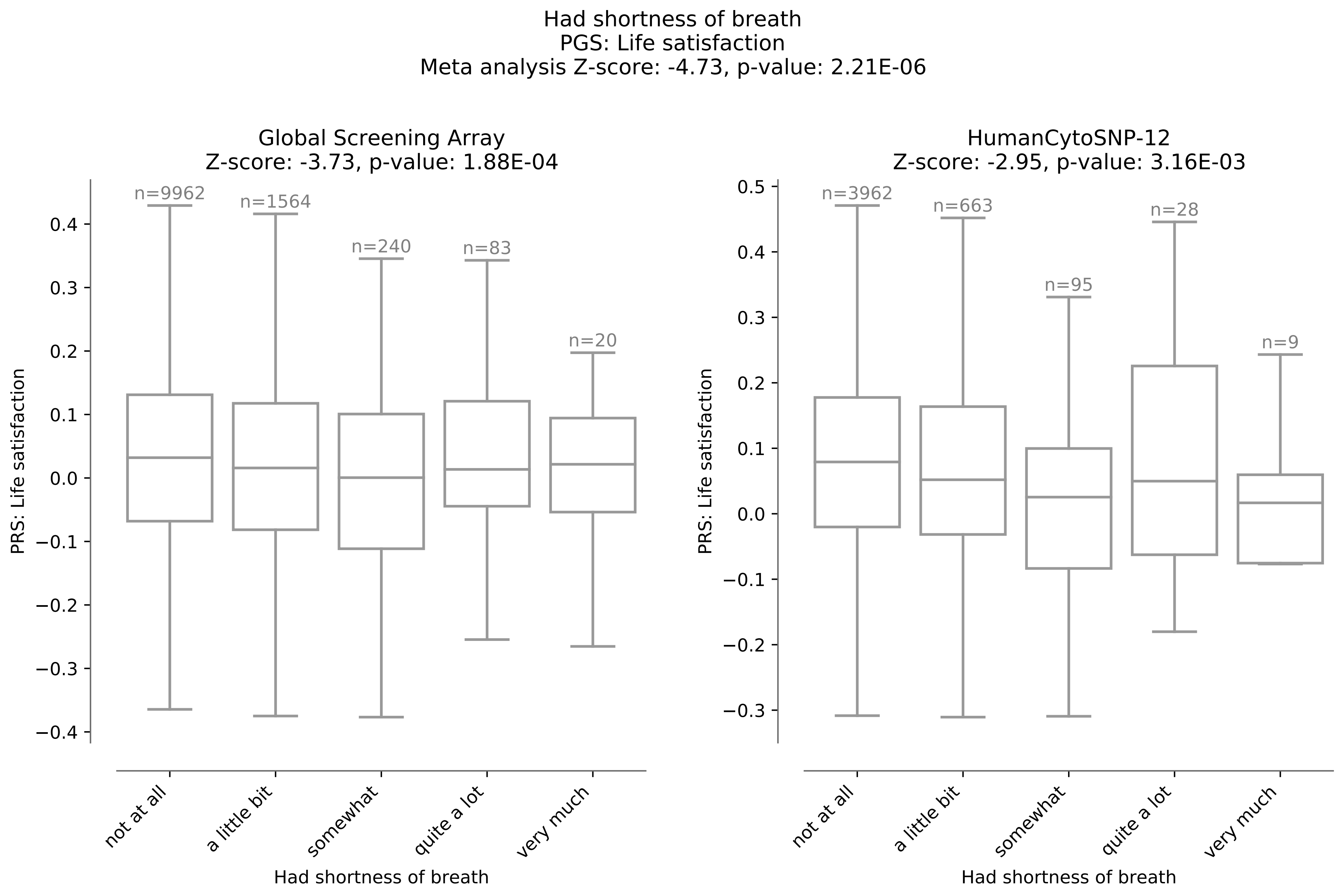
Had shortness of breath
PGS: Life satisfaction
Meta analysis Z-score: -4.73, p-value: 2.21E-06
Global Screening Array
Z-score: -3.73, p-value: 1.88E-04
n=9962
n=1564
n=240
n=83
n=20
PRS: Life satisfaction
not at all
a little bit
somewhat
quite a lot
very much
Had shortness of breath
HumanCytoSNP-12
Z-score: -2.95, p-value: 3.16E-03
n=3962
n=663
n=95
n=28
n=9
PRS: Life satisfaction
not at all
a little bit
somewhat
quite a lot
very much
Had shortness of breath

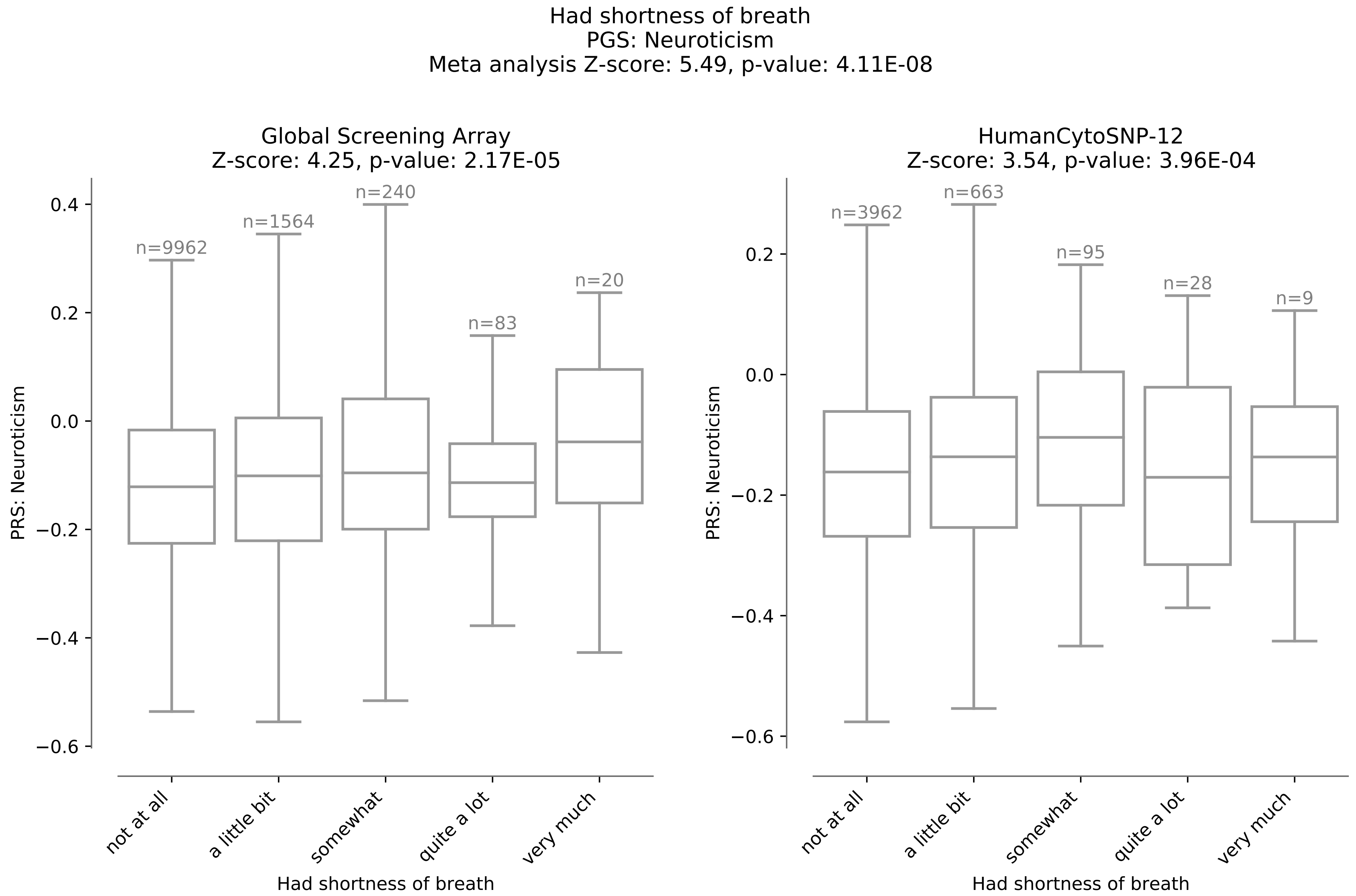
Had shortness of breath
PGS: Neuroticism
Meta analysis Z-score: 5.49, p-value: 4.11E-08
Global Screening Array
Z-score: 4.25, p-value: 2.17E-05
n=9962
n=1564
n=240
n=83
n=20
PRS: Neuroticism
0.4
0.2
0.0
−0.2
−0.4
−0.6
not at all
a little bit
somewhat
quite a lot
very much
Had shortness of breath
HumanCytoSNP-12
Z-score: 3.54, p-value: 3.96E-04
n=3962
n=663
n=95
n=28
n=9
PRS: Neuroticism
0.2
0.0
−0.2
−0.4
−0.6
not at all
a little bit
somewhat
quite a lot
very much
Had shortness of breath

Had shortness of breath
PGS: Schizophrenia
Meta analysis Z-score: 4.45, p-value: 8.54E-06

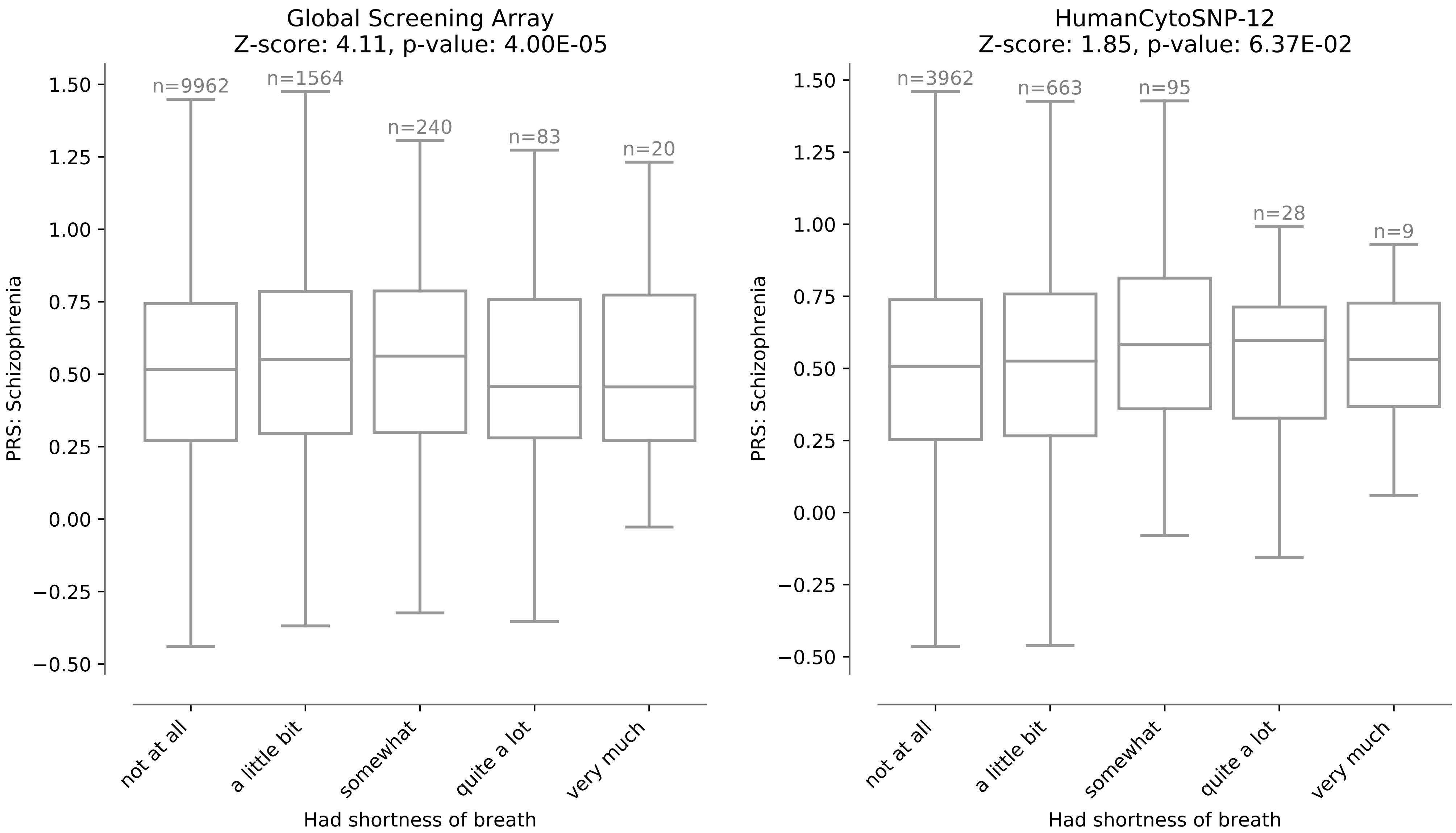

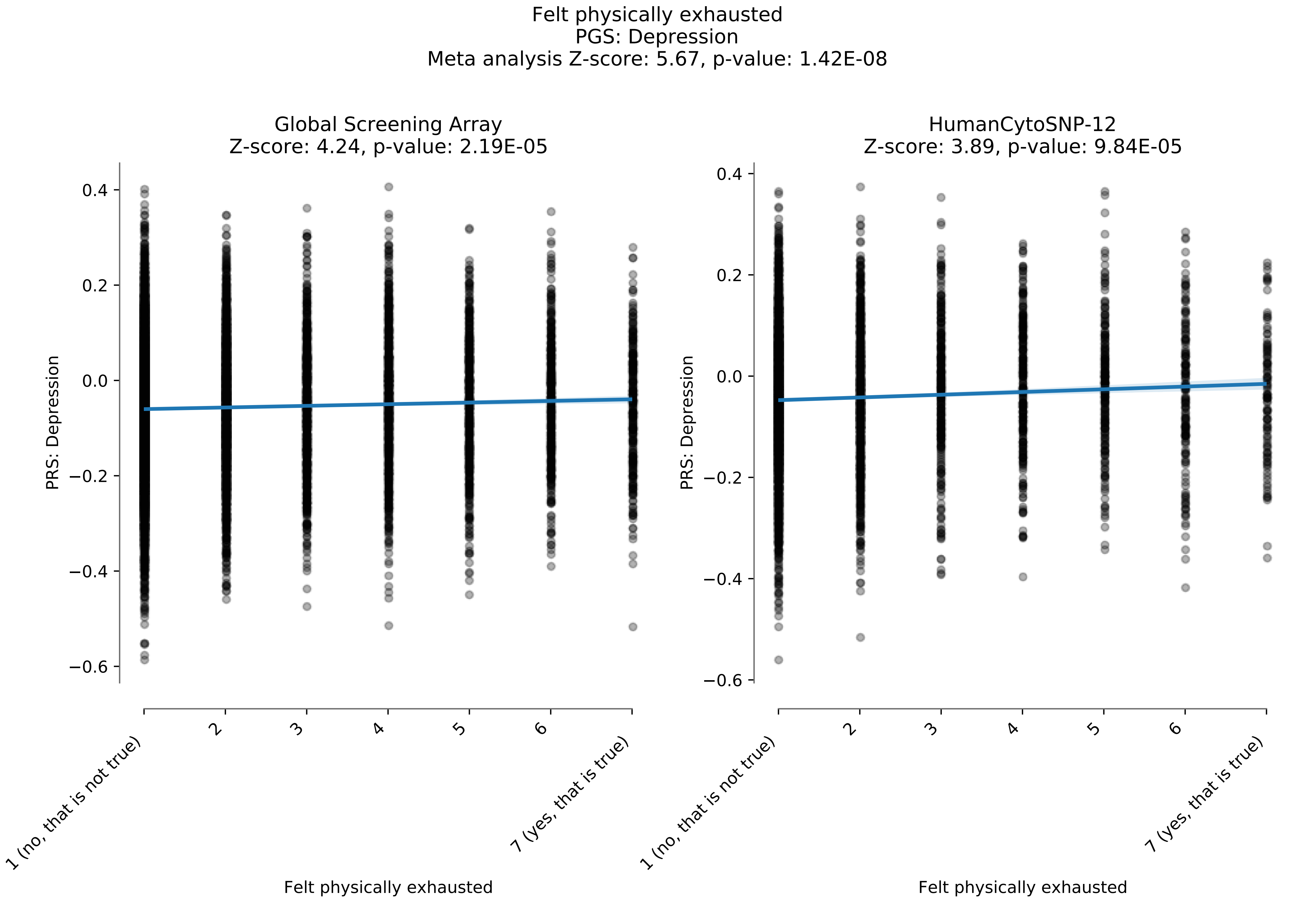
Felt physically exhausted
PGS: Depression
Meta analysis Z-score: 5.67, p-value: 1.42E-08
Global Screening Array
Z-score: 4.24, p-value: 2.19E-05
HumanCytoSNP-12
Z-score: 3.89, p-value: 9.84E-05
PRS: Depression
0.4
0.2
0.0
−0.2
−0.4
−0.6
1 (no, that is not true)
2
3
4
5
6
7 (yes, that is true)
Felt physically exhausted

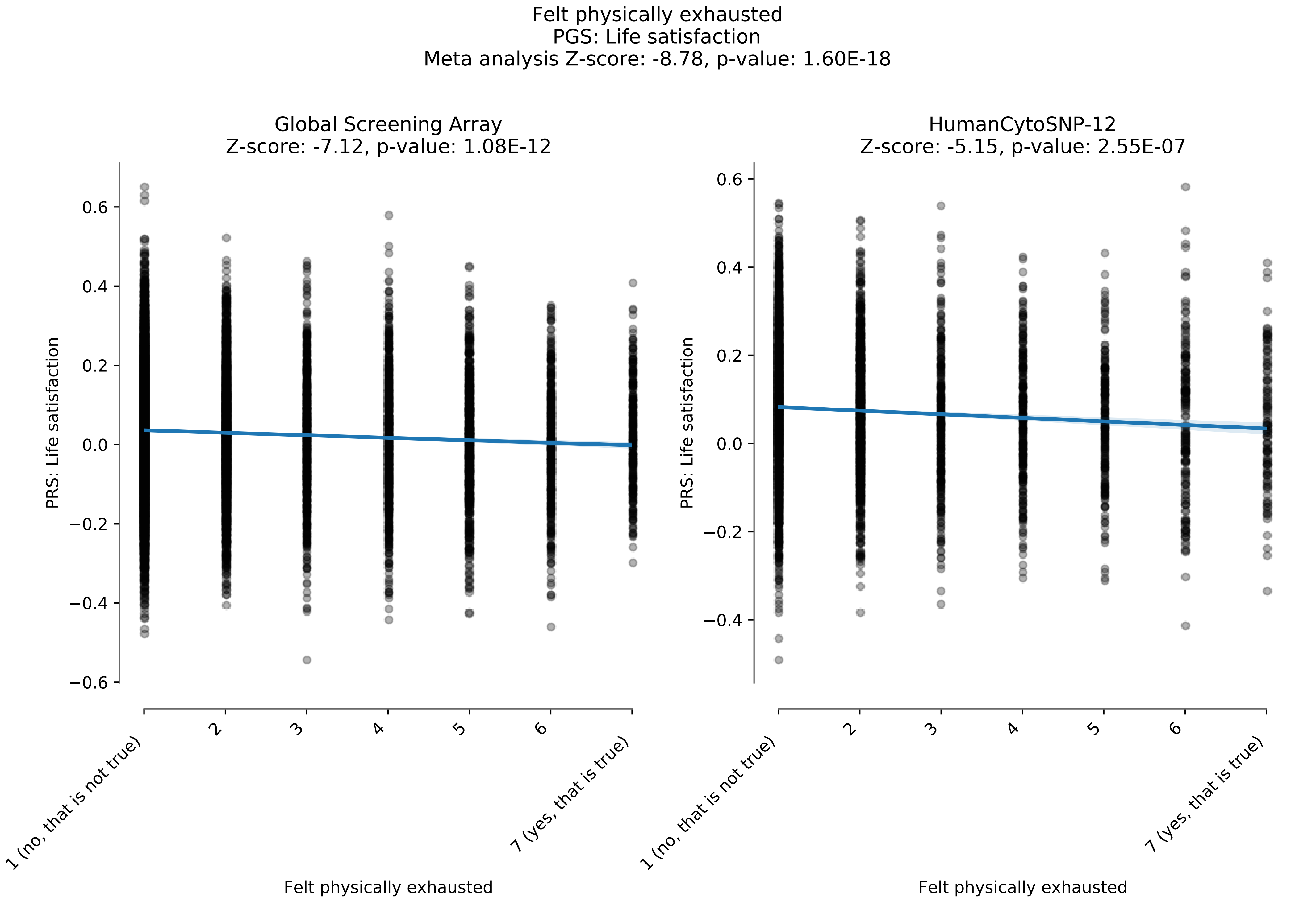

Felt physically exhausted
PGS: Life satisfaction
Meta analysis Z-score: -8.78, p-value: 1.60E-18
Global Screening Array
Z-score: -7.12, p-value: 1.08E-12
HumanCytoSNP-12
Z-score: -5.15, p-value: 2.55E-07
PRS: Life satisfaction
1 (no, that is not true)
2
3
4
5
6
7 (yes, that is true)
Felt physically exhausted

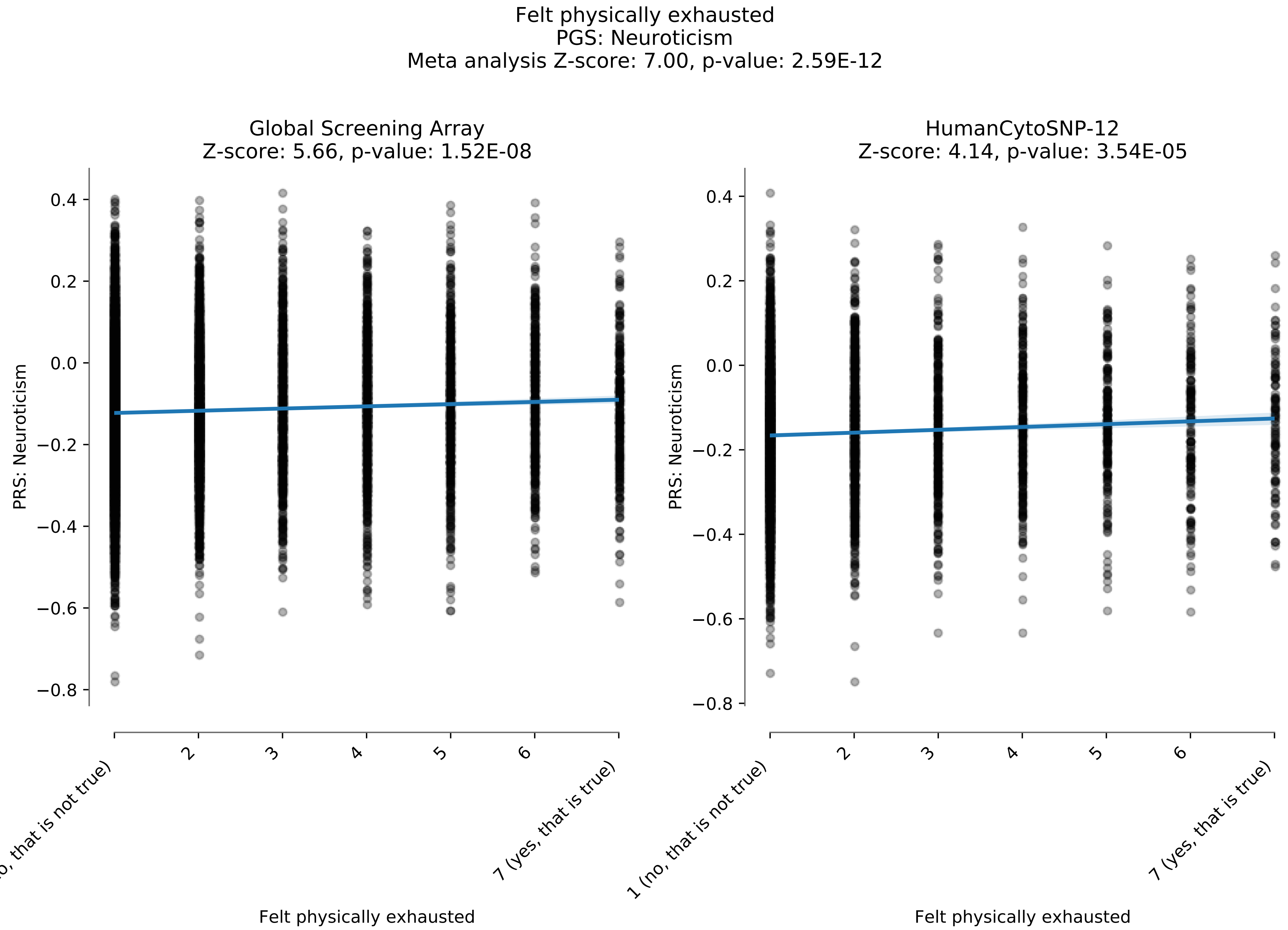
Felt physically exhausted
PGS: Neuroticism
Meta analysis Z-score: 7.00, p-value: 2.59E-12
Global Screening Array
Z-score: 5.66, p-value: 1.52E-08
HumanCytoSNP-12
Z-score: 4.14, p-value: 3.54E-05
PRS: Neuroticism
0.4
0.2
0.0
−0.2
−0.4
−0.6
−0.8
1 (no, that is not true)
2
3
4
5
6
7 (yes, that is true)
Felt physically exhausted

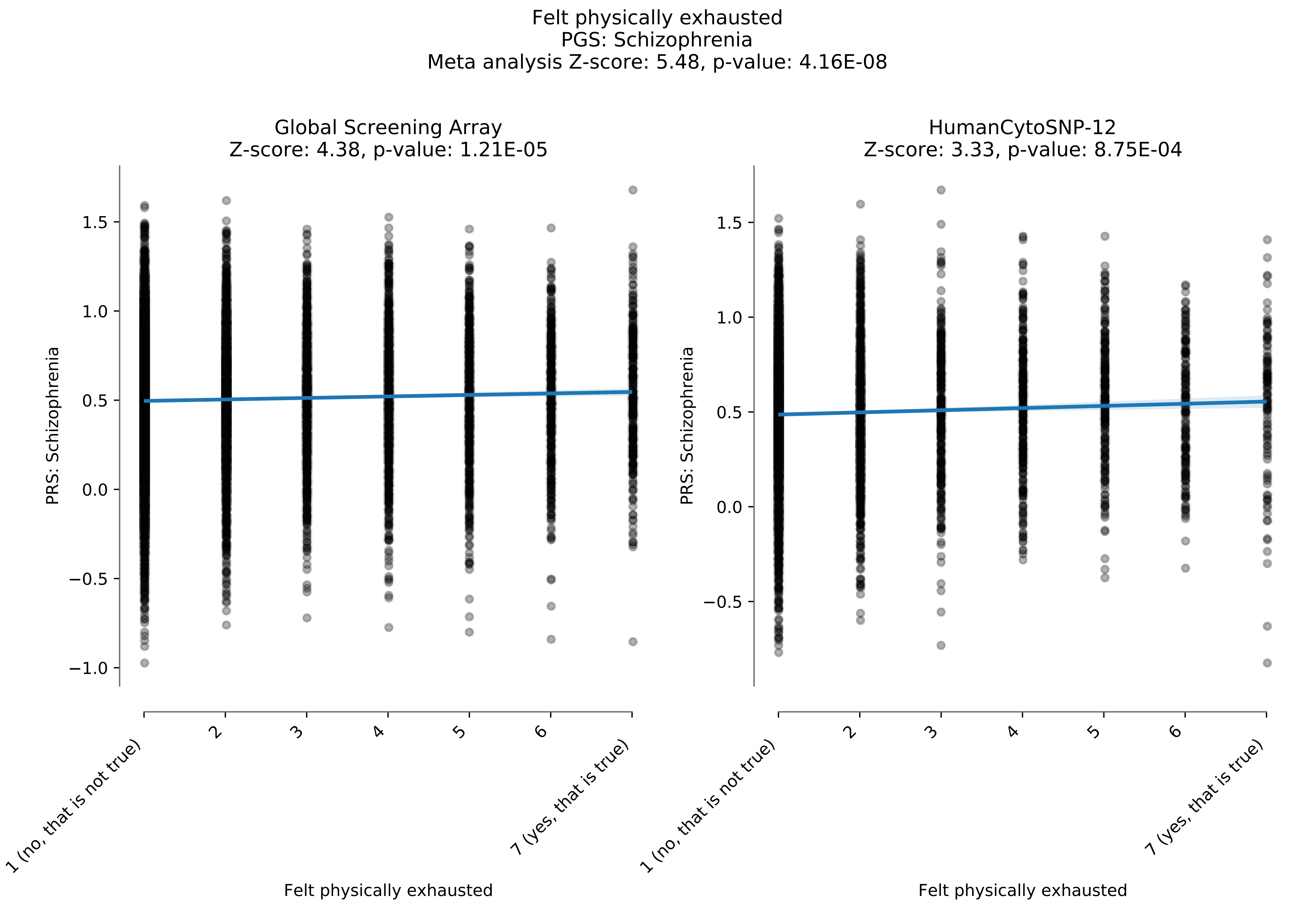
Felt physically exhausted
PGS: Schizophrenia
Meta analysis Z-score: 5.48, p-value: 4.16E-08
Global Screening Array
Z-score: 4.38, p-value: 1.21E-05
HumanCytoSNP-12
Z-score: 3.33, p-value: 8.75E-04
PRS: Schizophrenia
1.5
1.0
0.5
0.0
−0.5
−1.0
1 (no, that is not true)
2
3
4
5
6
7 (yes, that is true)
Felt physically exhausted

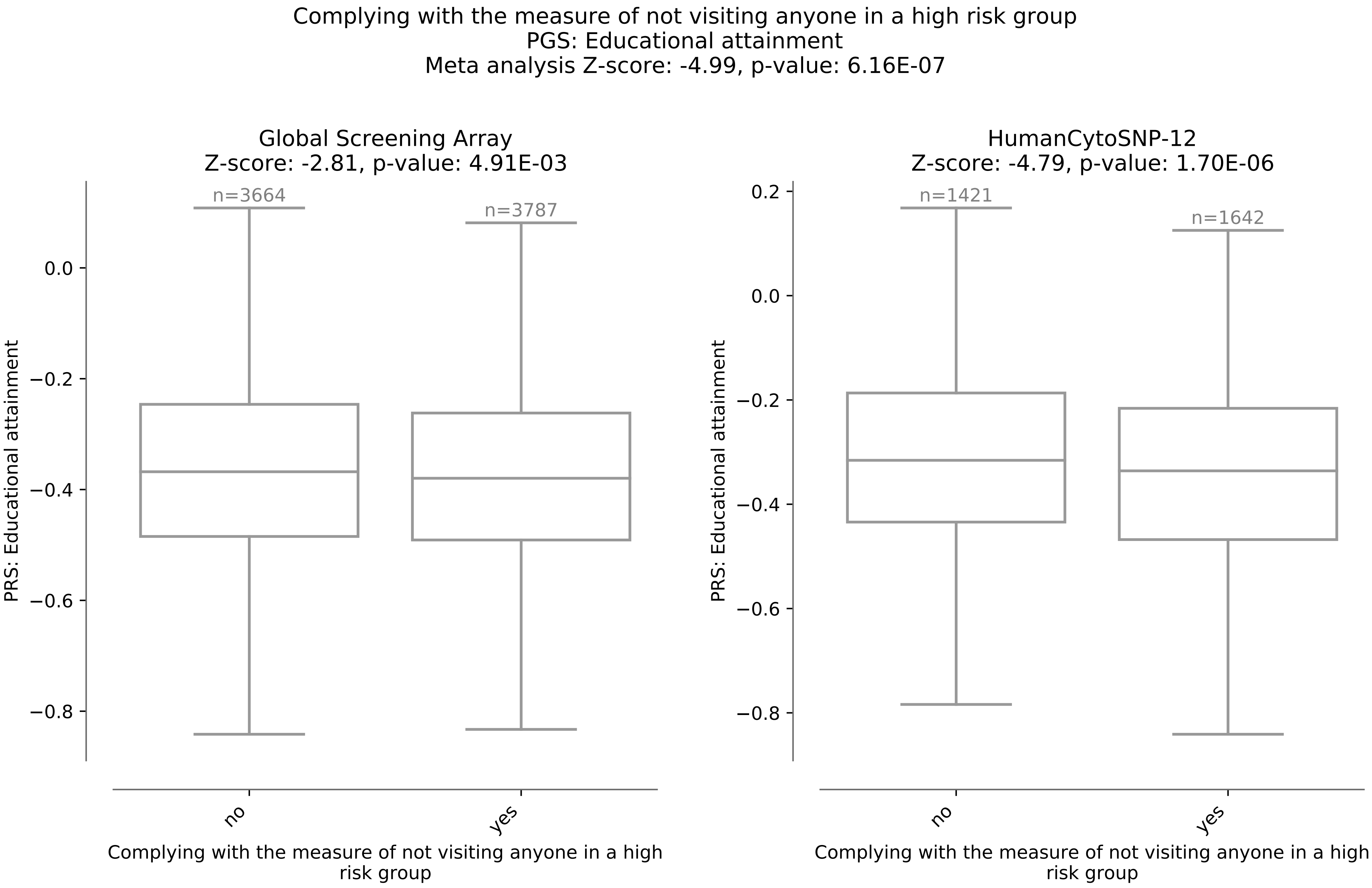
Complying with the measure of not visiting anyone in a high risk group
PGS: Educational attainment
Meta analysis Z-score: -4.99, p-value: 6.16E-07
Global Screening Array
Z-score: -2.81, p-value: 4.91E-03
n=3664
n=3787
PRS: Educational attainment
0.0
−0.2
−0.4
−0.6
−0.8
no
yes
Complying with the measure of not visiting anyone in a high risk group
HumanCytoSNP-12
Z-score: -4.79, p-value: 1.70E-06
n=1421
n=1642
PRS: Educational attainment
0.2
0.0
−0.2
−0.4
−0.6
−0.8
no
yes
Complying with the measure of not visiting anyone in a high risk group

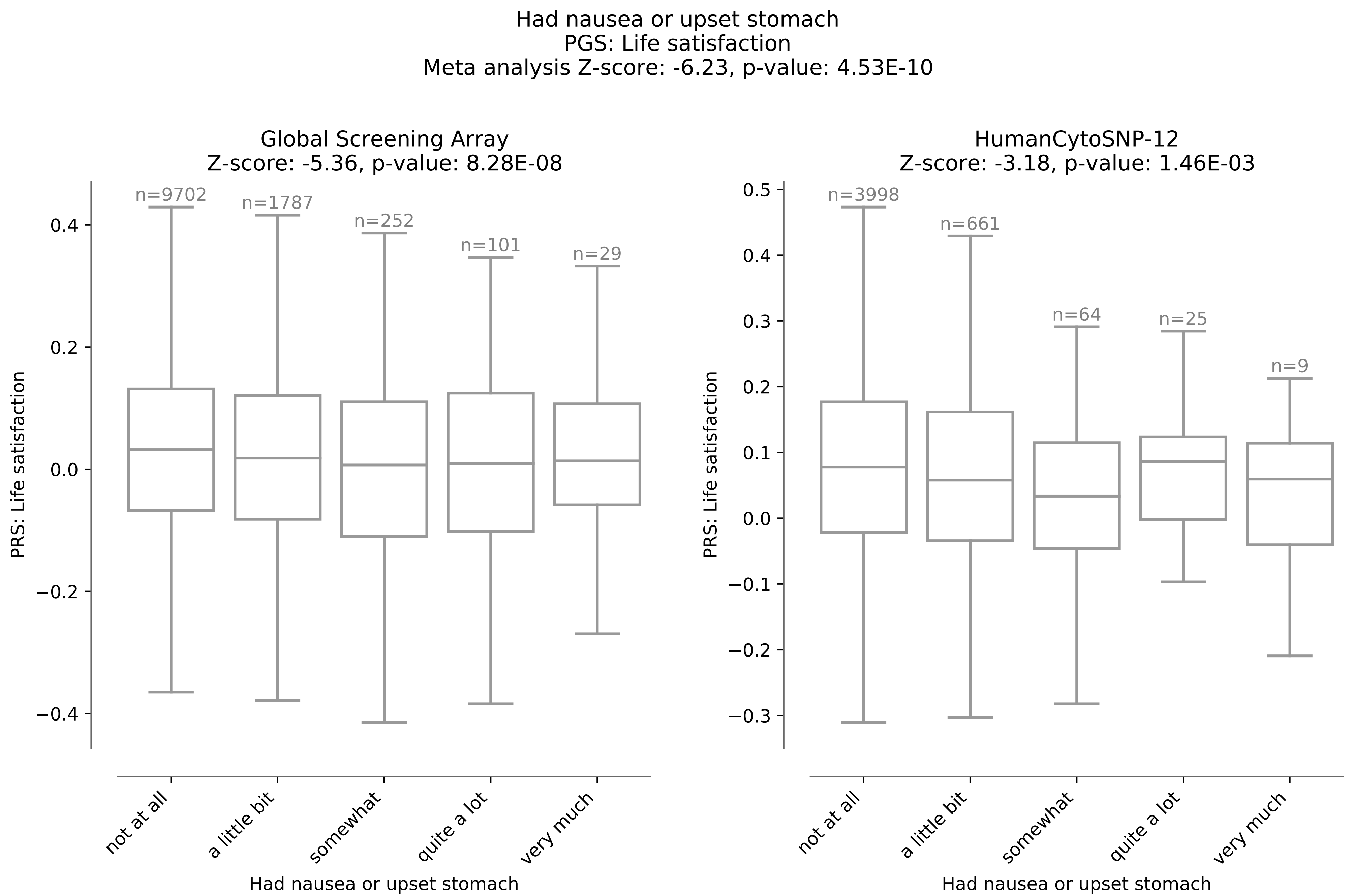

Had nausea or upset stomach
PGS: Life satisfaction
Meta analysis Z-score: -6.23, p-value: 4.53E-10
Global Screening Array
Z-score: -5.36, p-value: 8.28E-08
n=9702
n=1787
n=252
n=101
n=29
PRS: Life satisfaction
not at all
a little bit
somewhat
quite a lot
very much
Had nausea or upset stomach
HumanCytoSNP-12
Z-score: -3.18, p-value: 1.46E-03
n=3998
n=661
n=64
n=25
n=9
PRS: Life satisfaction
not at all
a little bit
somewhat
quite a lot
very much
Had nausea or upset stomach

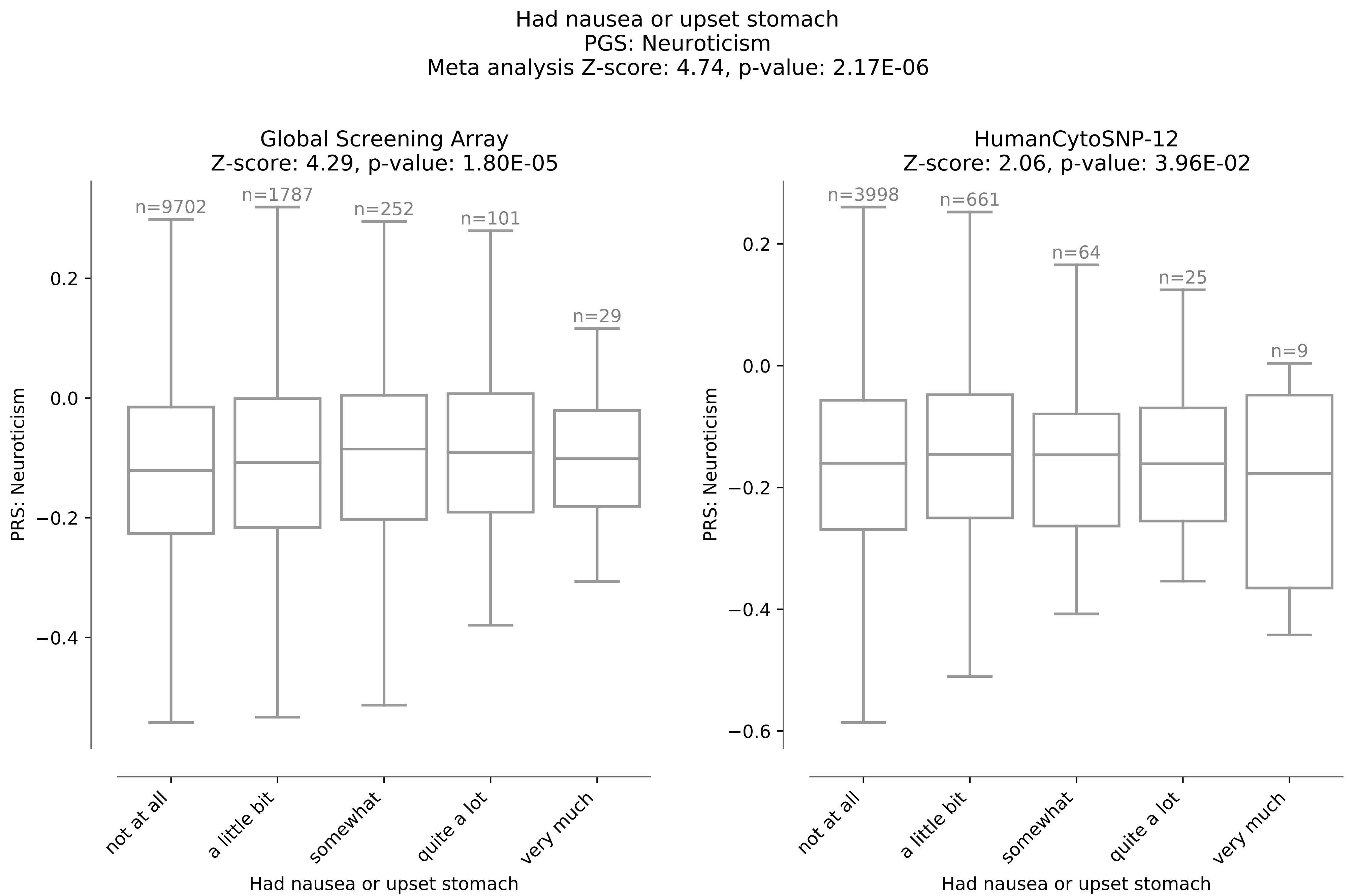

Had nausea or upset stomach
PGS: Neuroticism
Meta analysis Z-score: 4.74, p-value: 2.17E-06
Global Screening Array
Z-score: 4.29, p-value: 1.80E-05
HumanCytoSNP-12
Z-score: 2.06, p-value: 3.96E-02
PRS: Neuroticism
n=9702
n=1787
n=252
n=101
n=29
n=3998
n=661
n=64
n=25
n=9
0.2
0.0
−0.2
−0.4
−0.6
not at all
a little bit
somewhat
quite a lot
very much
Had nausea or upset stomach

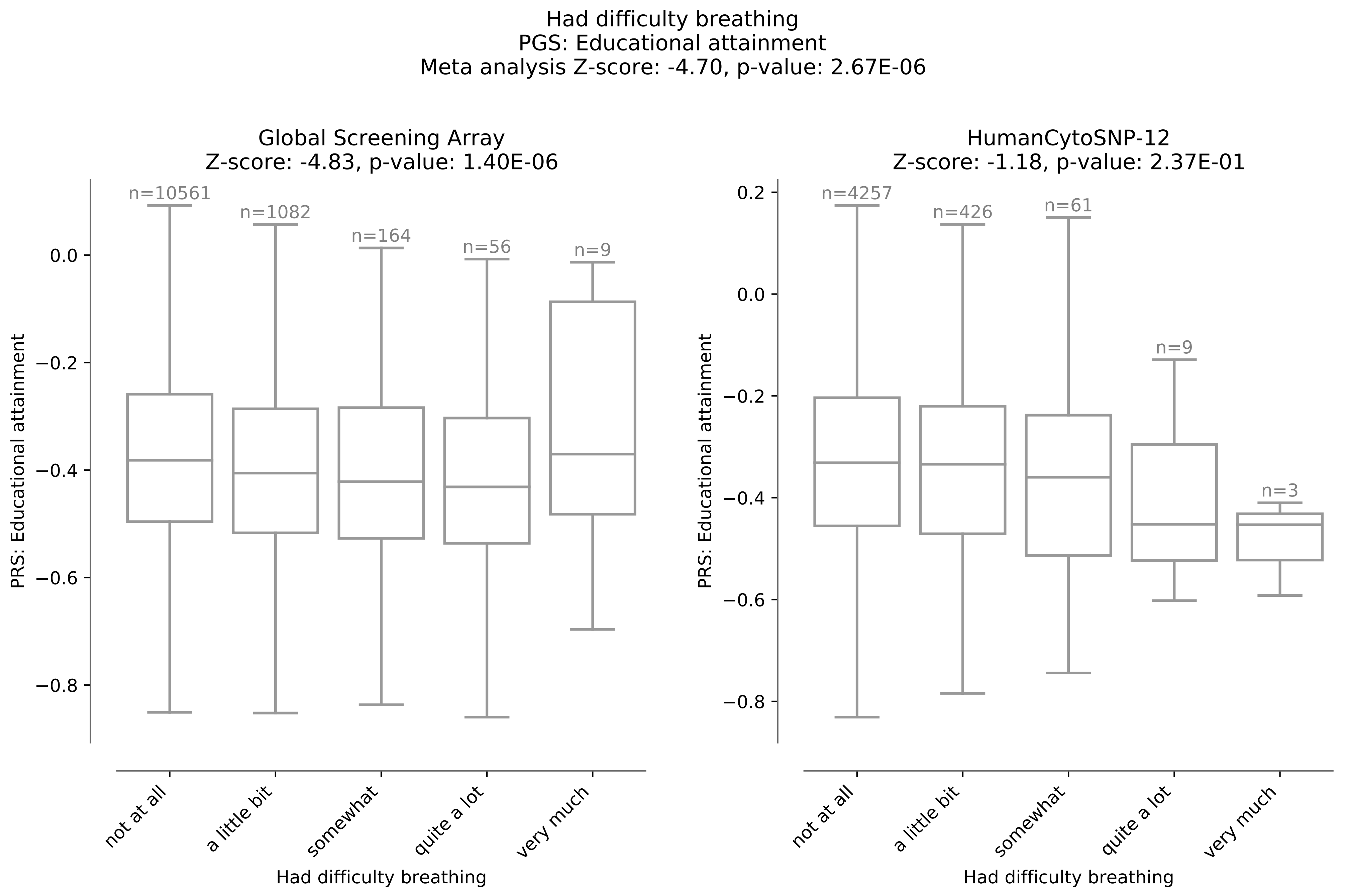
Had difficulty breathing
PGS: Educational attainment
Meta analysis Z-score: -4.70, p-value: 2.67E-06
Global Screening Array
Z-score: -4.83, p-value: 1.40E-06
HumanCytoSNP-12
Z-score: -1.18, p-value: 2.37E-01
PRS: Educational attainment
n=10561
n=1082
n=164
n=56
n=9
n=4257
n=426
n=61
n=9
n=3
0.2
0.0
−0.2
−0.4
−0.6
−0.8
not at all
a little bit
somewhat
quite a lot
very much
Had difficulty breathing

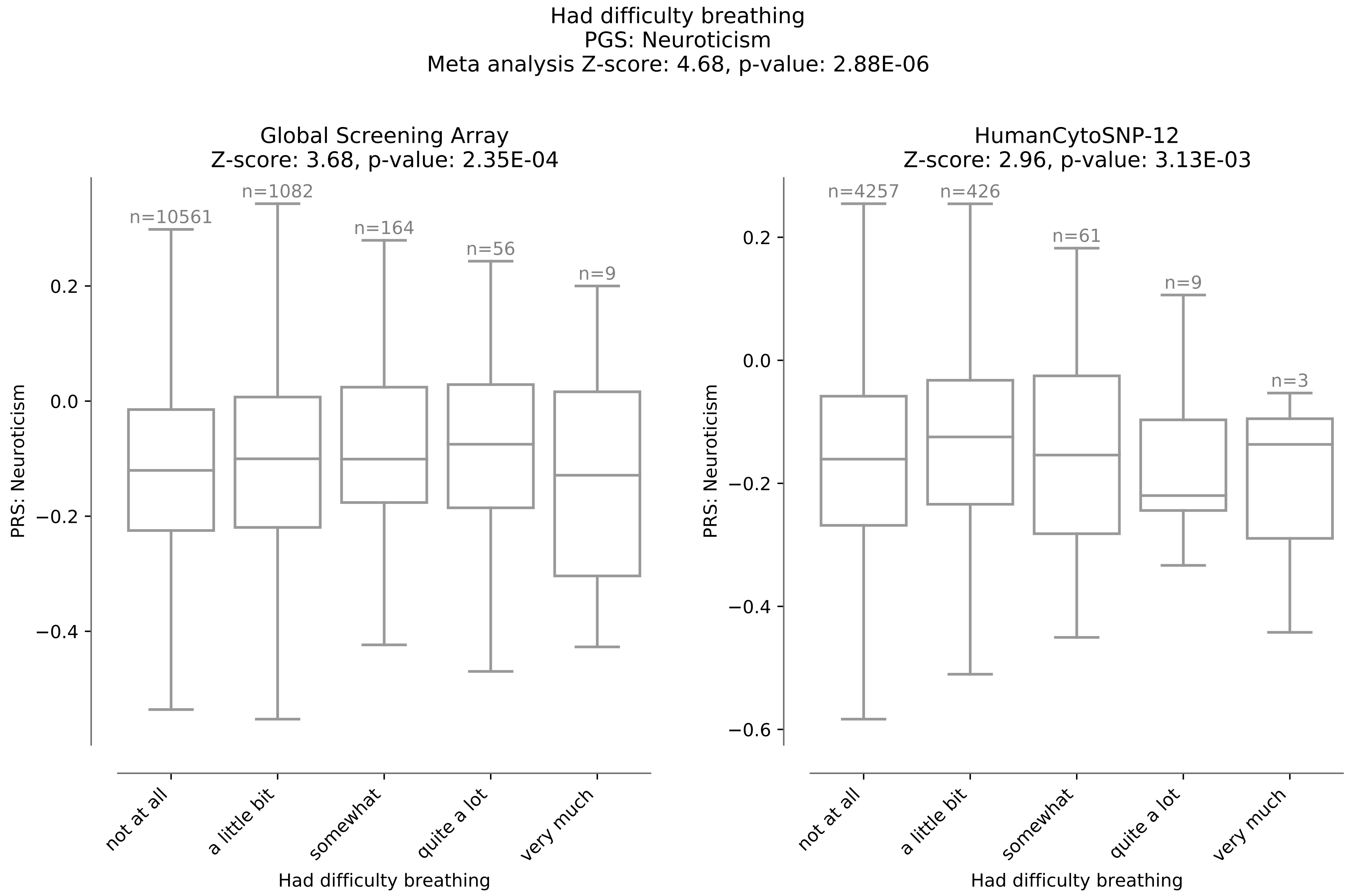
Had difficulty breathing
PGS: Neuroticism
Meta analysis Z-score: 4.68, p-value: 2.88E-06
Global Screening Array
Z-score: 3.68, p-value: 2.35E-04
n=10561
n=1082
n=164
n=56
n=9
0.2
0.0
−0.2
−0.4
PRS: Neuroticism
not at all
a little bit
somewhat
quite a lot
very much
Had difficulty breathing
HumanCytoSNP-12
Z-score: 2.96, p-value: 3.13E-03
n=4257
n=426
n=61
n=9
n=3
0.2
0.0
−0.2
−0.4
−0.6
PRS: Neuroticism
not at all
a little bit
somewhat
quite a lot
very much
Had difficulty breathing

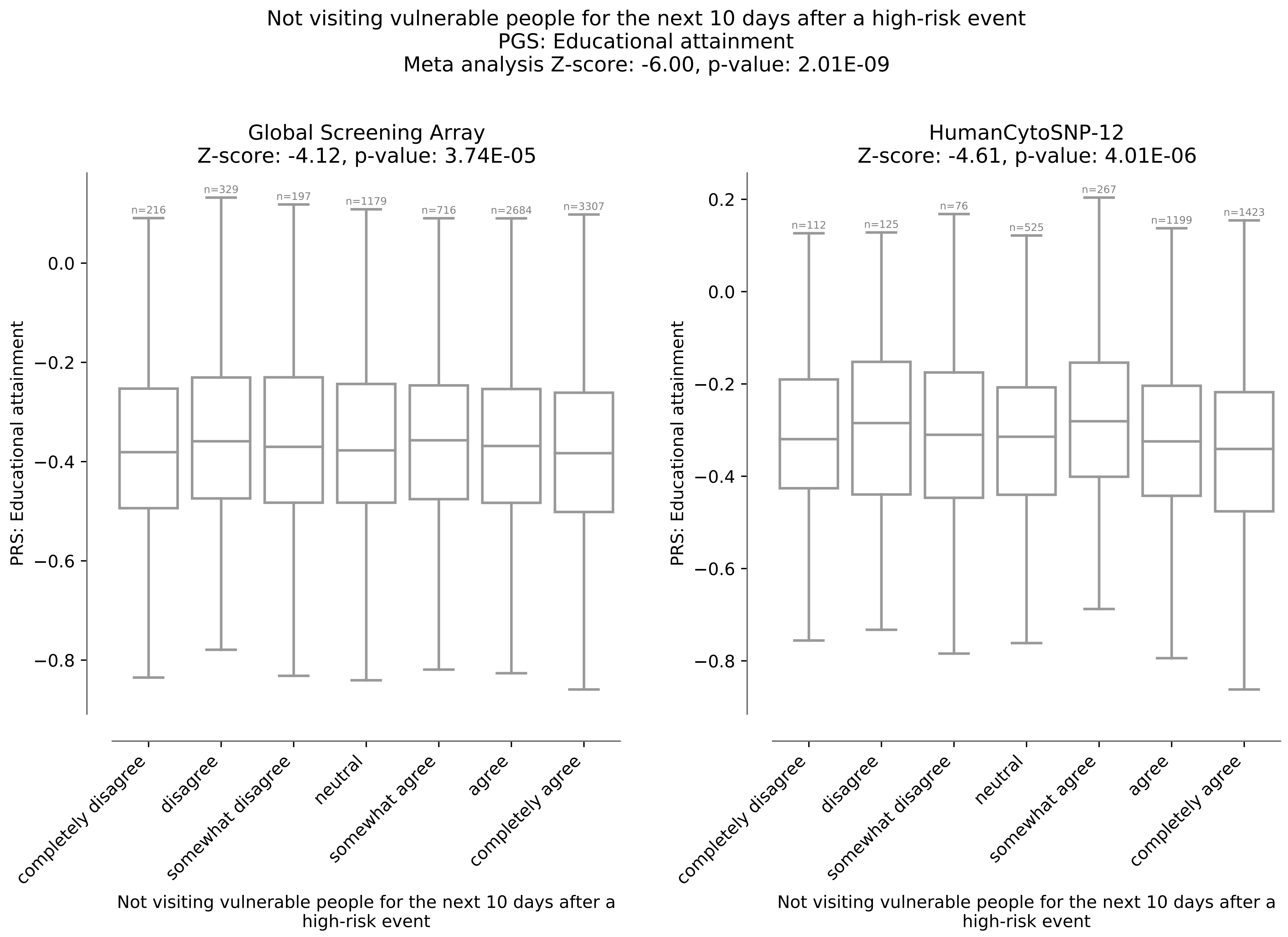

Not visiting vulnerable people for the next 10 days after a high-risk event
PGS: Educational attainment
Meta analysis Z-score: -6.00, p-value: 2.01E-09
Global Screening Array
Z-score: -4.12, p-value: 3.74E-05
n=216
n=329
n=197
n=1179
n=716
n=2684
n=3307
PRS: Educational attainment
0.0
−0.2
−0.4
−0.6
−0.8
completely disagree
disagree
somewhat disagree
neutral
somewhat agree
agree
completely agree
Not visiting vulnerable people for the next 10 days after a high-risk event
HumanCytoSNP-12
Z-score: -4.61, p-value: 4.01E-06
n=112
n=125
n=76
n=525
n=267
n=1199
n=1423
PRS: Educational attainment
0.2
0.0
−0.2
−0.4
−0.6
−0.8
completely disagree
disagree
somewhat disagree
neutral
somewhat agree
agree
completely agree
Not visiting vulnerable people for the next 10 days after a high-risk event

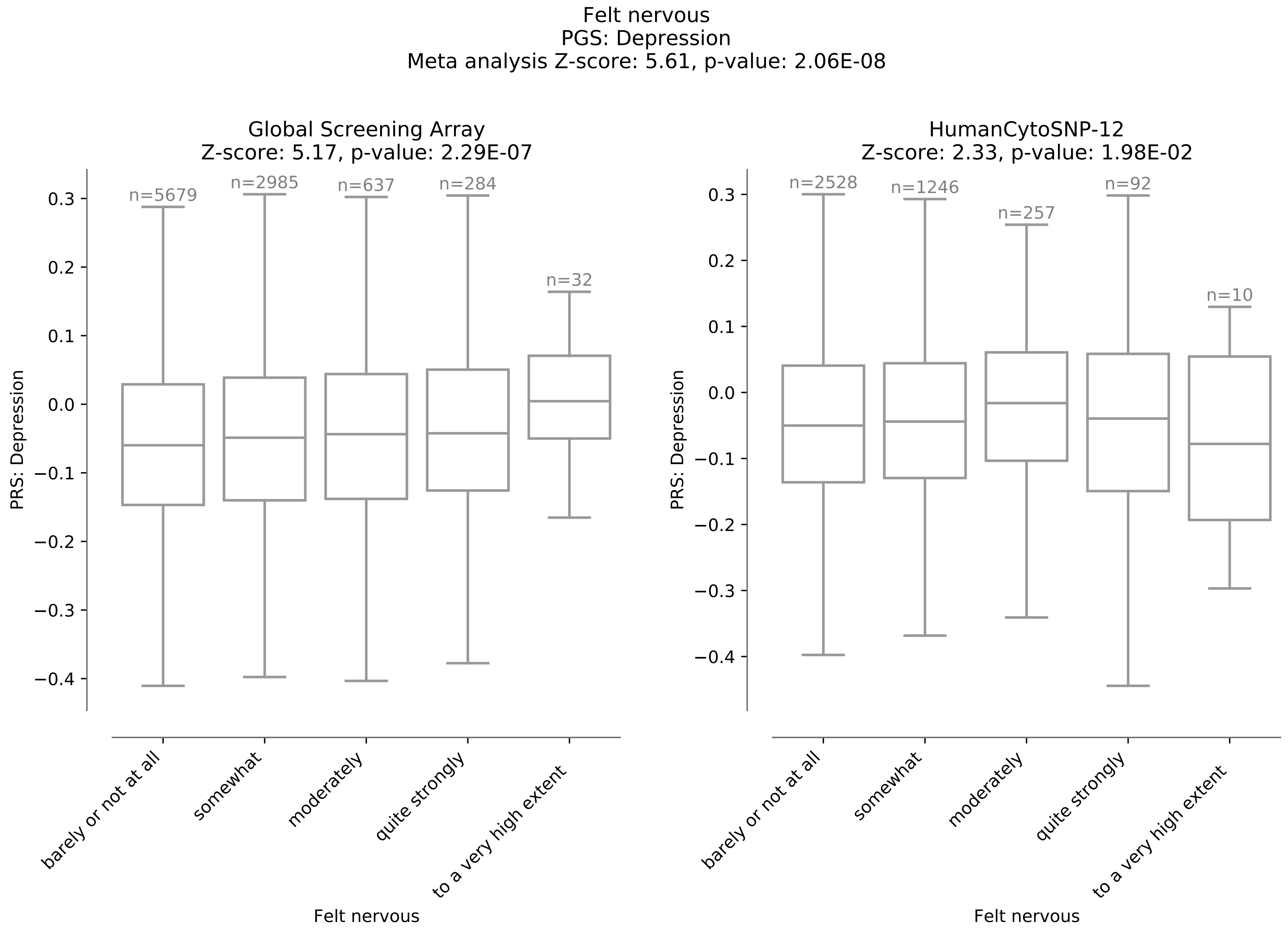
Felt nervous
PGS: Depression
Meta analysis Z-score: 5.61, p-value: 2.06E-08
Global Screening Array
Z-score: 5.17, p-value: 2.29E-07
HumanCytoSNP-12
Z-score: 2.33, p-value: 1.98E-02
PRS: Depression
n=5679
n=2985
n=637
n=284
n=32
n=2528
n=1246
n=257
n=92
n=10
0.3
0.2
0.1
0.0
−0.1
−0.2
−0.3
−0.4
barely or not at all
somewhat
moderately
quite strongly
to a very high extent
Felt nervous

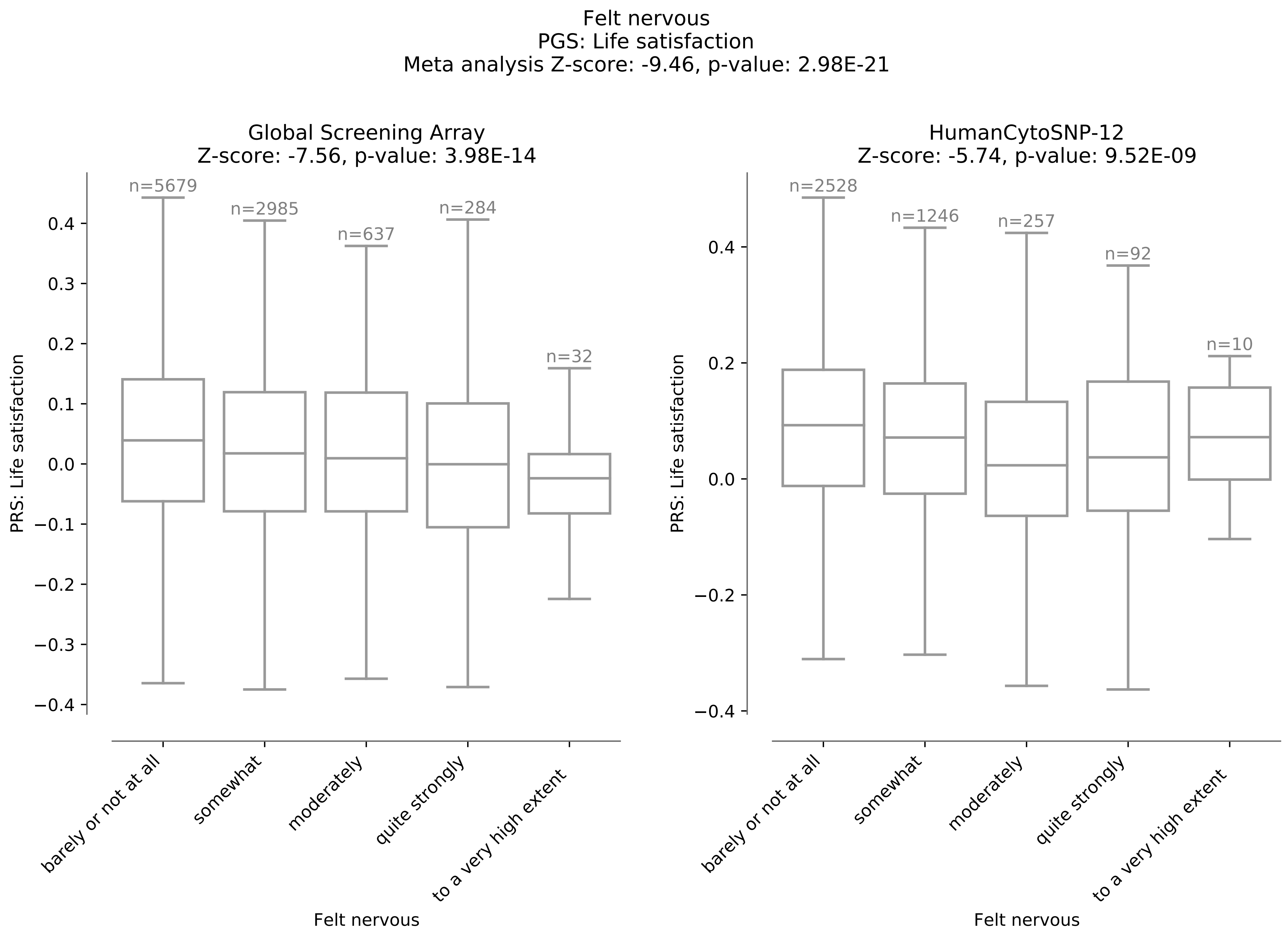

Felt nervous
PGS: Life satisfaction
Meta analysis Z-score: -9.46, p-value: 2.98E-21
Global Screening Array
Z-score: -7.56, p-value: 3.98E-14
n=5679
n=2985
n=637
n=284
n=32
PRS: Life satisfaction
0.4
0.3
0.2
0.1
0.0
−0.1
−0.2
−0.3
−0.4
barely or not at all
somewhat
moderately
quite strongly
to a very high extent
Felt nervous
HumanCytoSNP-12
Z-score: -5.74, p-value: 9.52E-09
n=2528
n=1246
n=257
n=92
n=10
PRS: Life satisfaction
0.4
0.2
0.0
−0.2
−0.4
barely or not at all
somewhat
moderately
quite strongly
to a very high extent
Felt nervous

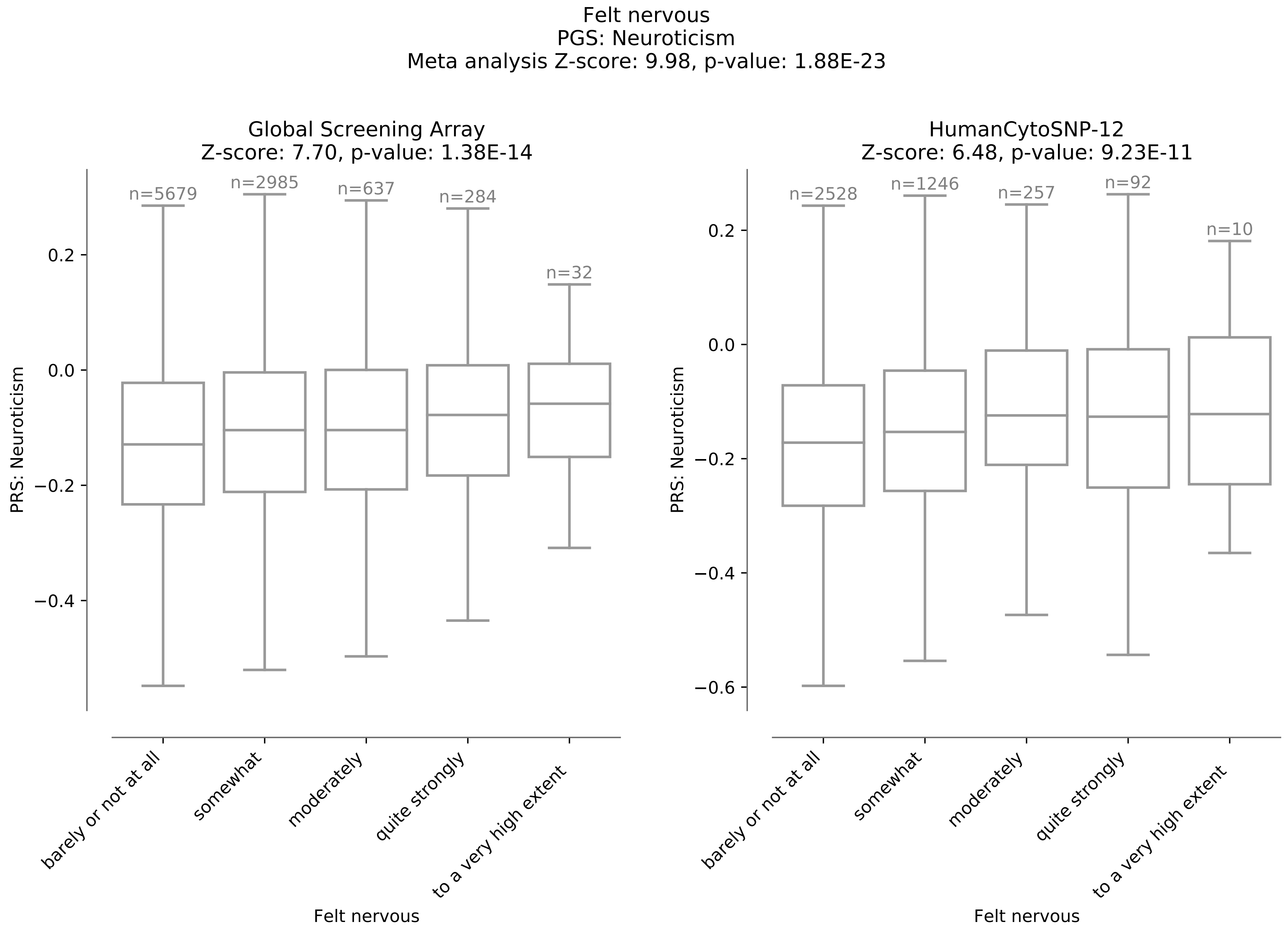

Felt nervous
PGS: Neuroticism
Meta analysis Z-score: 9.98, p-value: 1.88E-23
Global Screening Array
Z-score: 7.70, p-value: 1.38E-14
n=5679
n=2985
n=637
n=284
n=32
0.2
0.0
−0.2
−0.4
PRS: Neuroticism
barely or not at all
somewhat
moderately
quite strongly
to a very high extent
Felt nervous
HumanCytoSNP-12
Z-score: 6.48, p-value: 9.23E-11
n=2528
n=1246
n=257
n=92
n=10
0.2
0.0
−0.2
−0.4
−0.6
PRS: Neuroticism
barely or not at all
somewhat
moderately
quite strongly
to a very high extent
Felt nervous

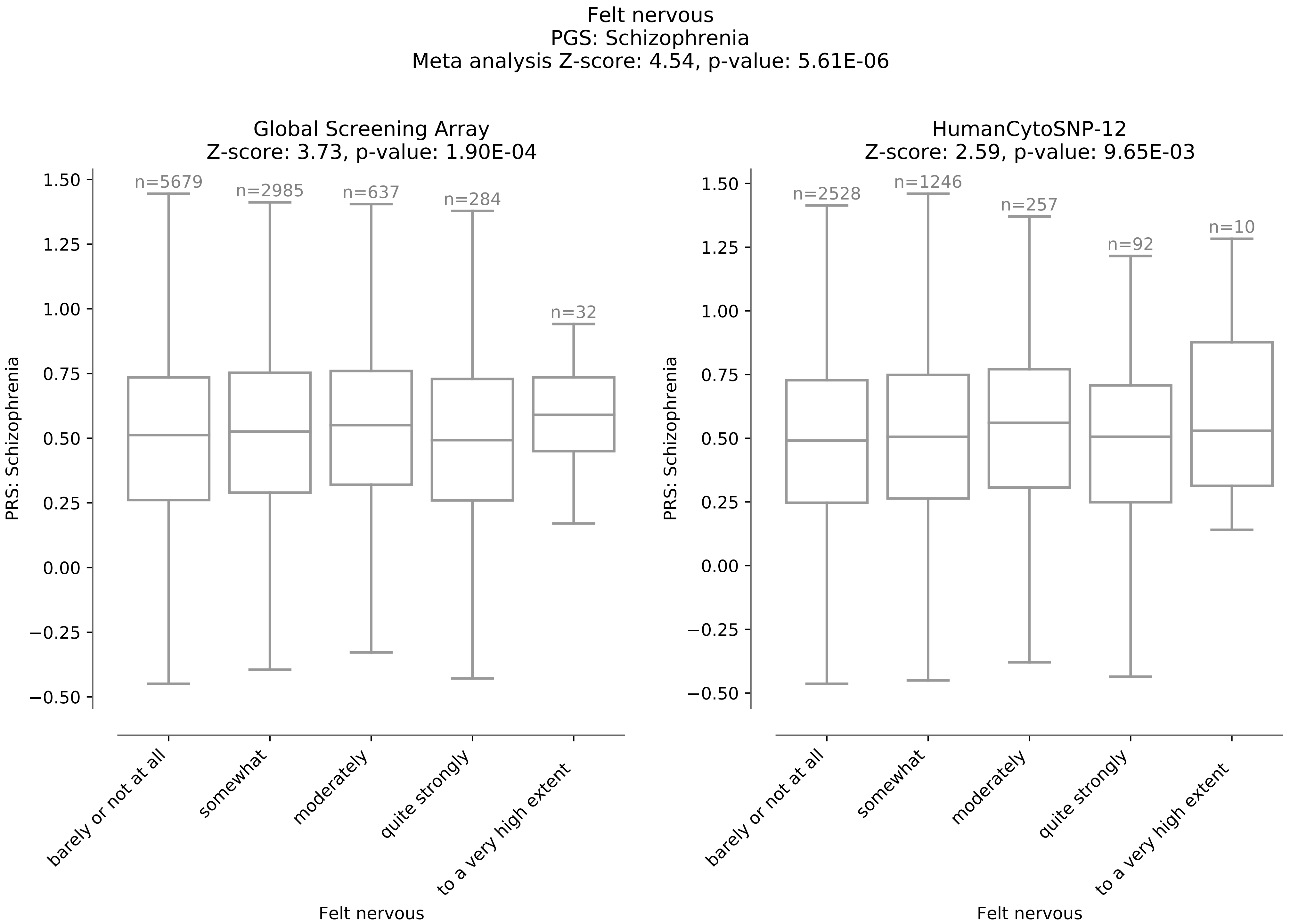

Felt nervous
PGS: Schizophrenia
Meta analysis Z-score: 4.54, p-value: 5.61E-06
Global Screening Array
Z-score: 3.73, p-value: 1.90E-04
n=5679
n=2985
n=637
n=284
n=32
PRS: Schizophrenia
1.50
1.25
1.00
0.75
0.50
0.25
0.00
−0.25
−0.50
barely or not at all
somewhat
moderately
quite strongly
to a very high extent
Felt nervous
HumanCytoSNP-12
Z-score: 2.59, p-value: 9.65E-03
n=2528
n=1246
n=257
n=92
n=10
PRS: Schizophrenia
1.50
1.25
1.00
0.75
0.50
0.25
0.00
−0.25
−0.50
barely or not at all
somewhat
moderately
quite strongly
to a very high extent
Felt nervous

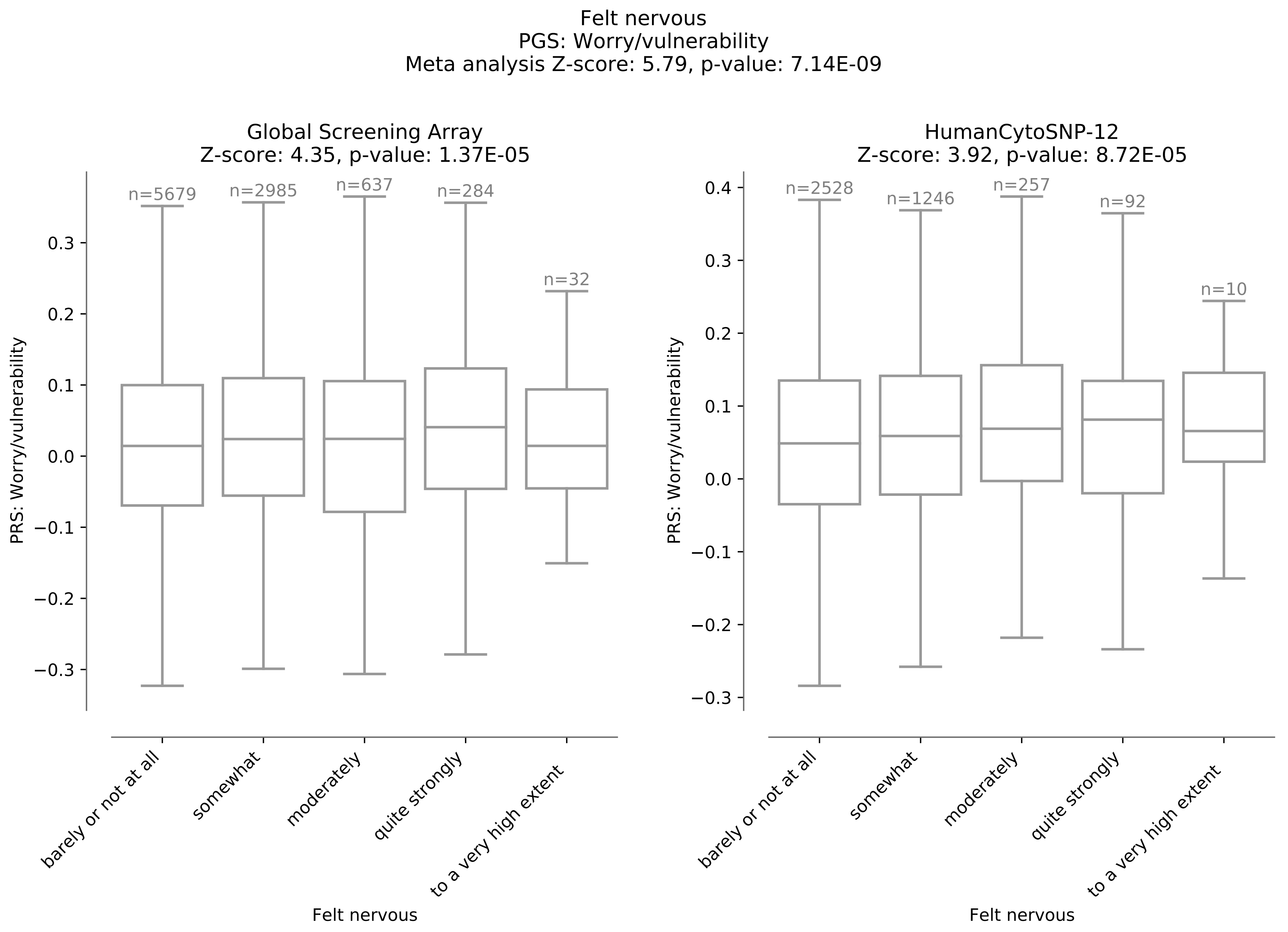
Felt nervous
PGS: Worry/vulnerability
Meta analysis Z-score: 5.79, p-value: 7.14E-09
Global Screening Array
Z-score: 4.35, p-value: 1.37E-05
HumanCytoSNP-12
Z-score: 3.92, p-value: 8.72E-05
PRS: Worry/vulnerability
n=5679
n=2985
n=637
n=284
n=32
n=2528
n=1246
n=257
n=92
n=10
0.4
0.3
0.2
0.1
0.0
−0.1
−0.2
−0.3
barely or not at all
somewhat
moderately
quite strongly
to a very high extent
Felt nervous

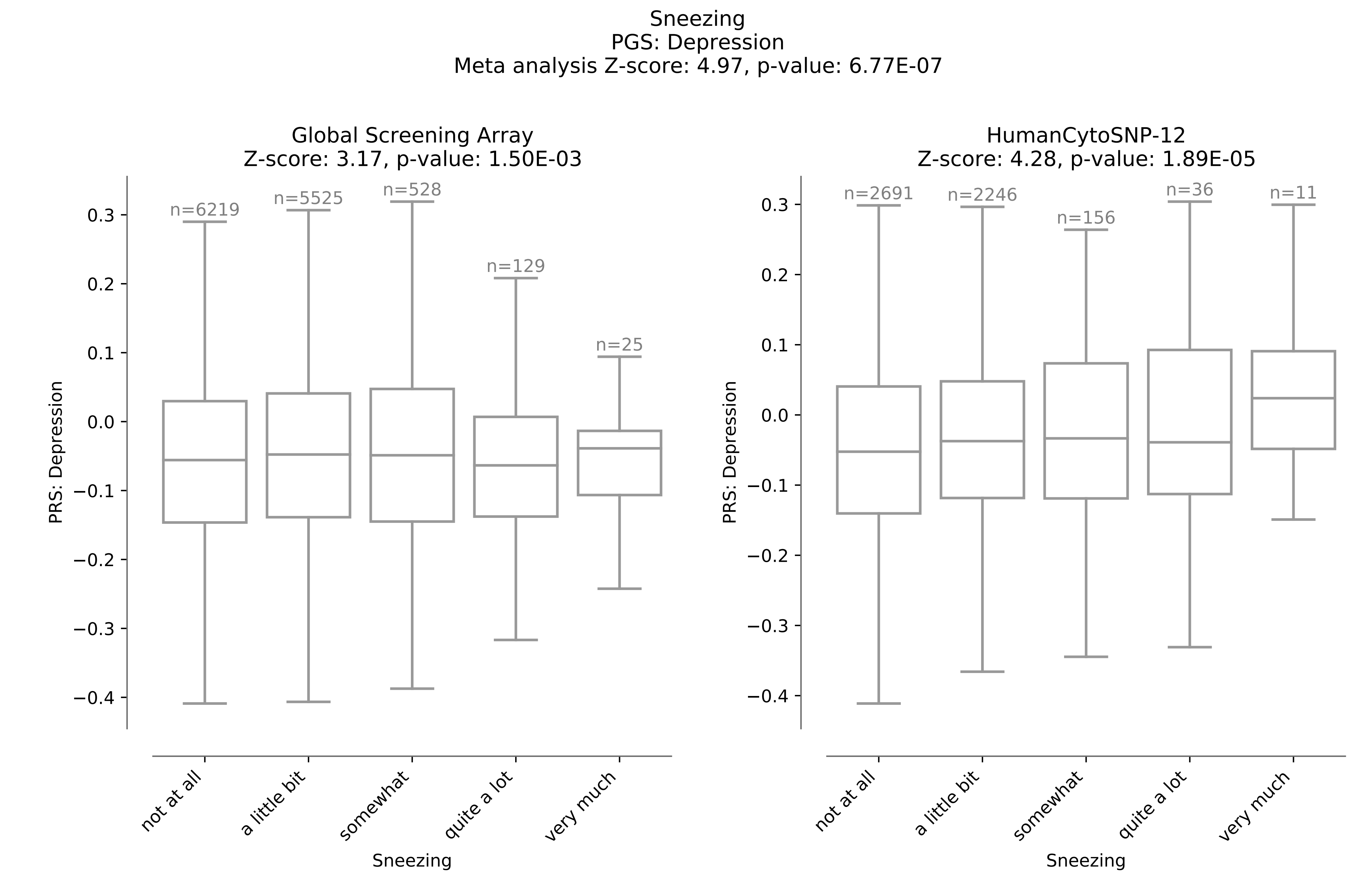
Sneezing
PGS: Depression
Meta analysis Z-score: 4.97, p-value: 6.77E-07
Global Screening Array
Z-score: 3.17, p-value: 1.50E-03
n=6219
n=5525
n=528
n=129
n=25
PRS: Depression
0.3
0.2
0.1
0.0
−0.1
−0.2
−0.3
−0.4
not at all
a little bit
somewhat
quite a lot
very much
Sneezing
HumanCytoSNP-12
Z-score: 4.28, p-value: 1.89E-05
n=2691
n=2246
n=156
n=36
n=11
PRS: Depression
not at all
a little bit
somewhat
quite a lot
very much
Sneezing

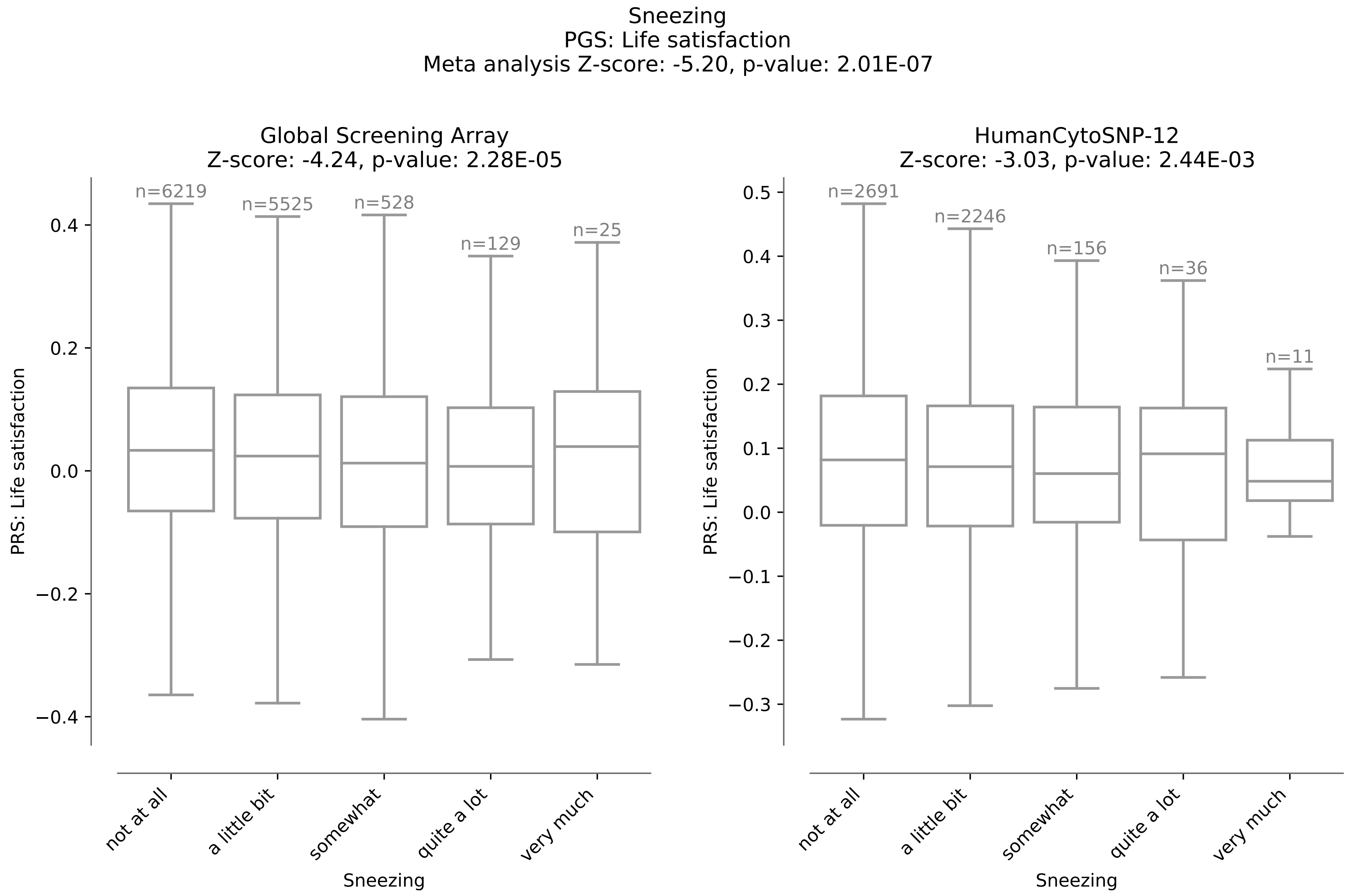

Sneezing
PGS: Life satisfaction
Meta analysis Z-score: -5.20, p-value: 2.01E-07
Global Screening Array
Z-score: -4.24, p-value: 2.28E-05
HumanCytoSNP-12
Z-score: -3.03, p-value: 2.44E-03
PRS: Life satisfaction
n=6219
n=5525
n=528
n=129
n=25
n=2691
n=2246
n=156
n=36
n=11
0.5
0.4
0.3
0.2
0.1
0.0
−0.1
−0.2
−0.3
−0.4
not at all
a little bit
somewhat
quite a lot
very much
Sneezing

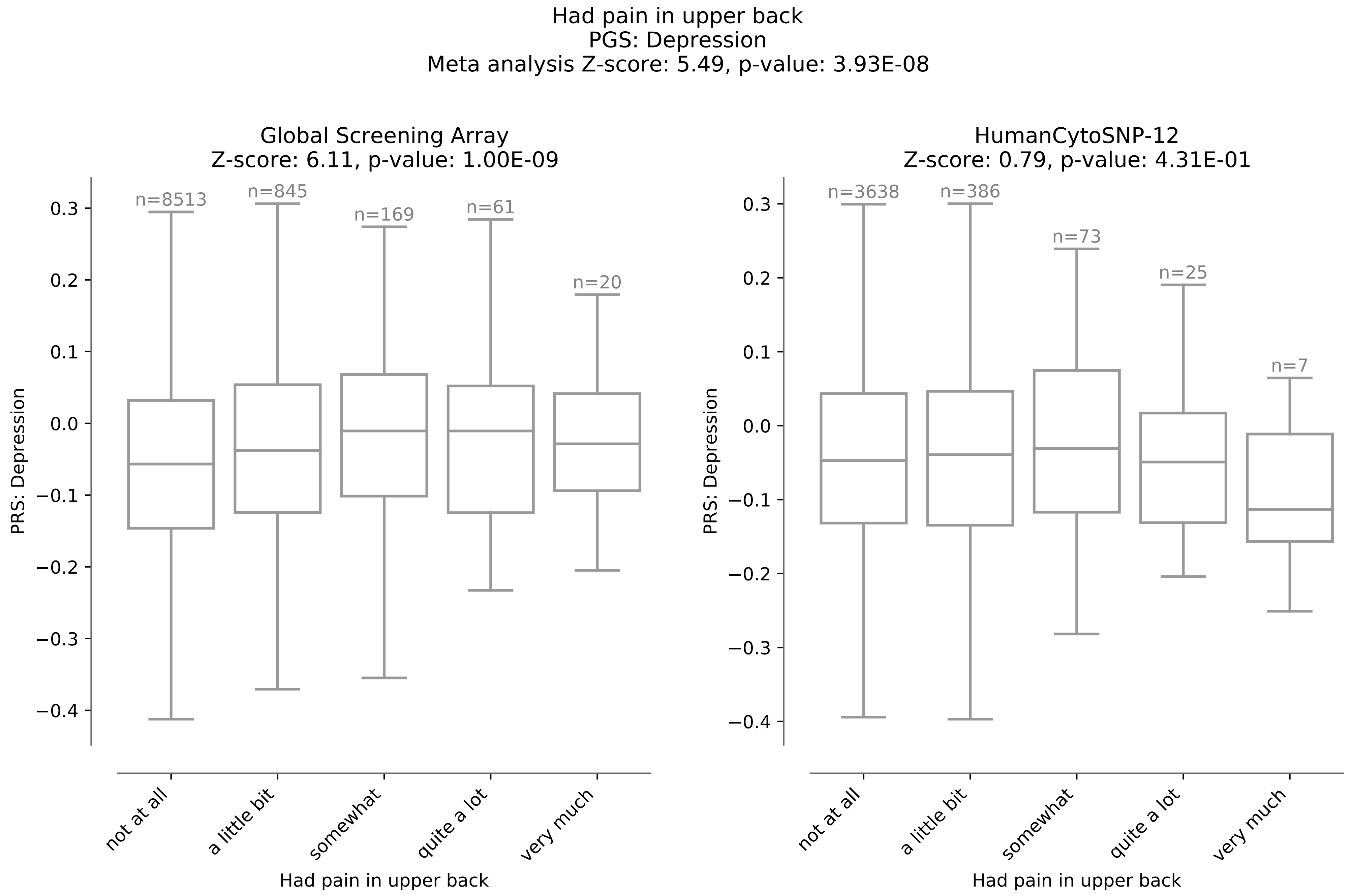

Had pain in upper back
PGS: Depression
Meta analysis Z-score: 5.49, p-value: 3.93E-08
Global Screening Array
Z-score: 6.11, p-value: 1.00E-09
HumanCytoSNP-12
Z-score: 0.79, p-value: 4.31E-01
n=8513
n=845
n=169
n=61
n=20
n=3638
n=386
n=73
n=25
n=7
PRS: Depression
not at all
a little bit
somewhat
quite a lot
very much
Had pain in upper back

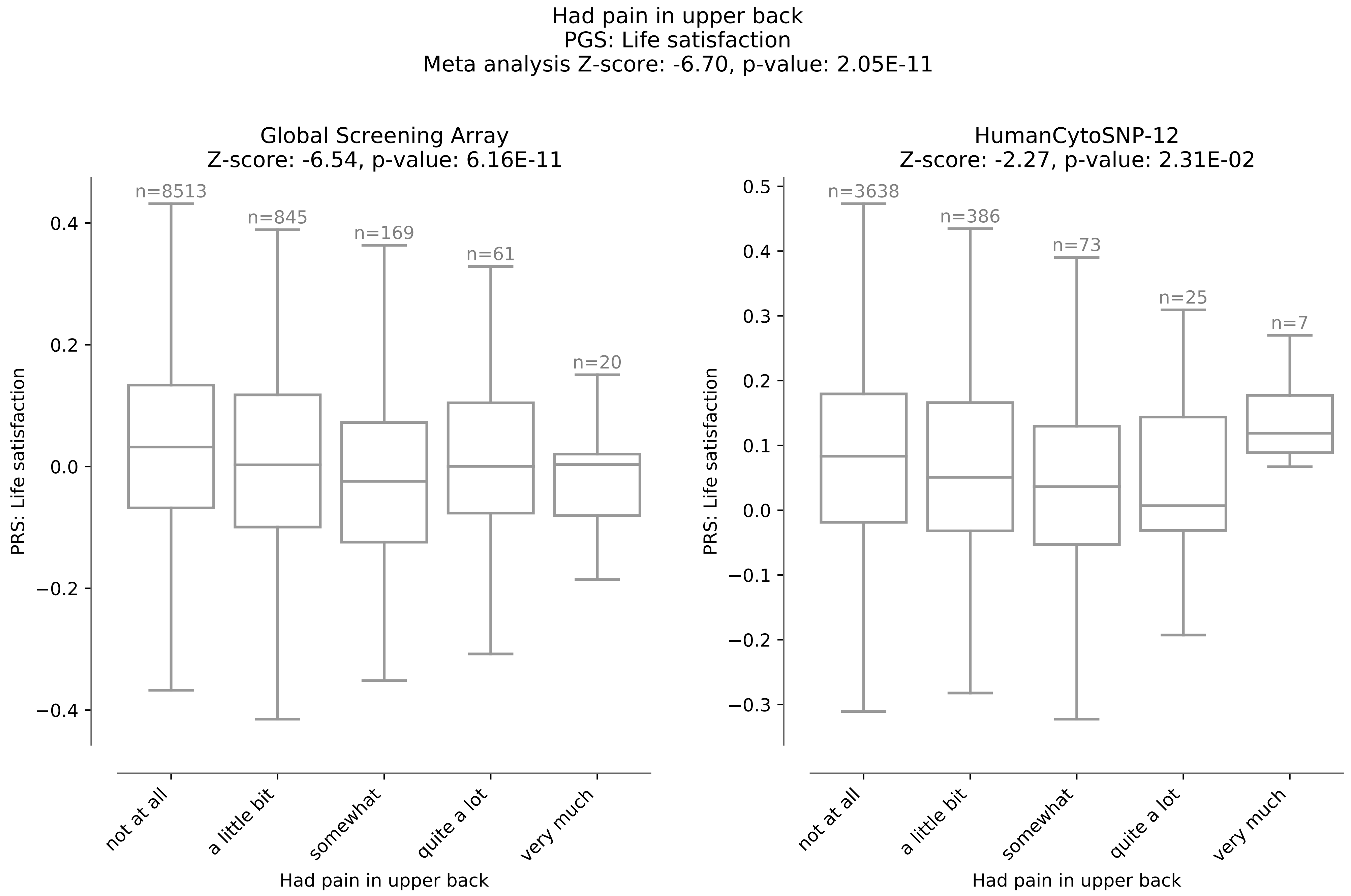
Had pain in upper back
PGS: Life satisfaction
Meta analysis Z-score: -6.70, p-value: 2.05E-11
Global Screening Array
Z-score: -6.54, p-value: 6.16E-11
n=8513
n=845
n=169
n=61
n=20
PRS: Life satisfaction
not at all
a little bit
somewhat
quite a lot
very much
Had pain in upper back
HumanCytoSNP-12
Z-score: -2.27, p-value: 2.31E-02
n=3638
n=386
n=73
n=25
n=7
PRS: Life satisfaction
not at all
a little bit
somewhat
quite a lot
very much
Had pain in upper back

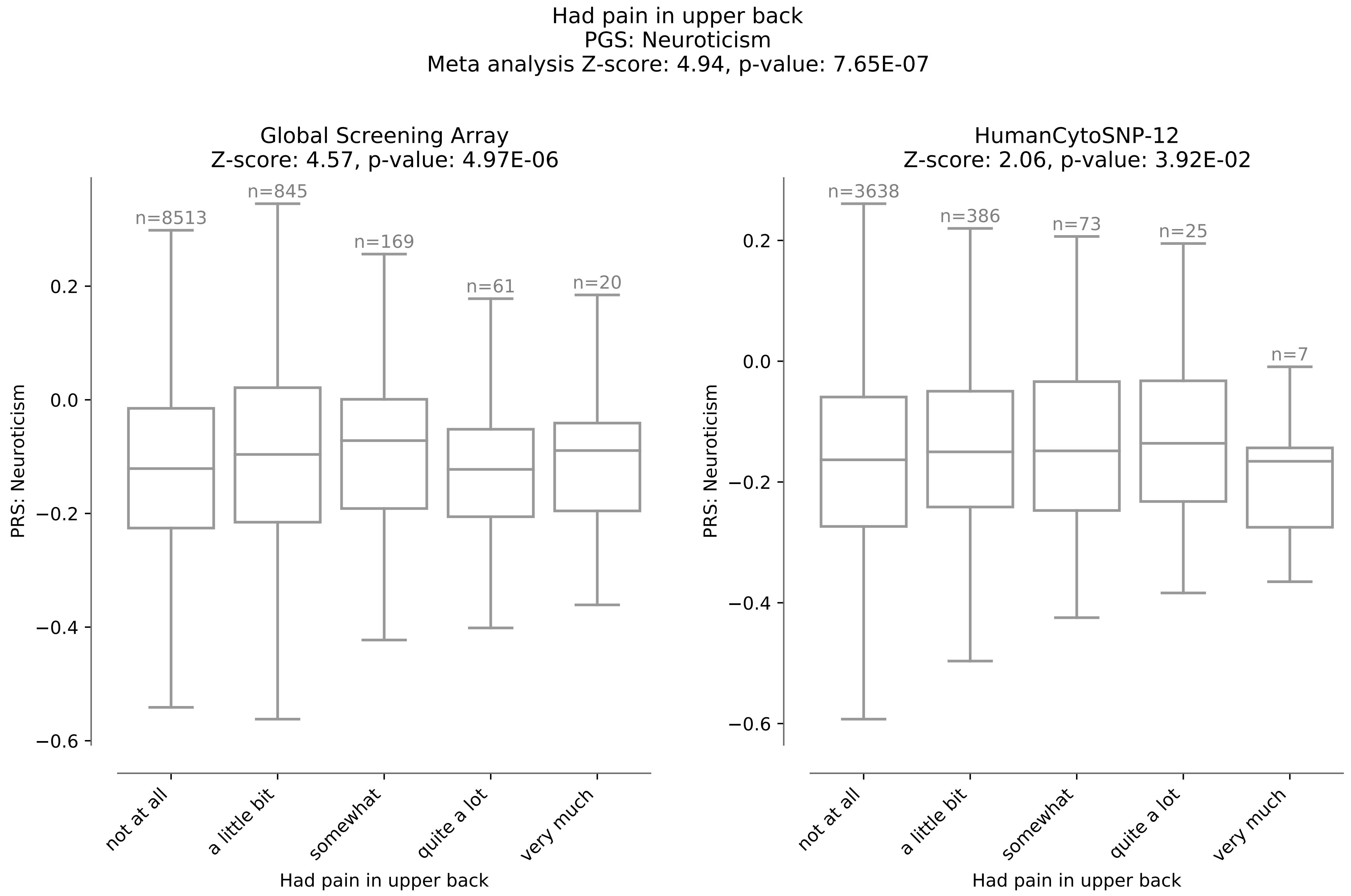
Had pain in upper back
PGS: Neuroticism
Meta analysis Z-score: 4.94, p-value: 7.65E-07
Global Screening Array
Z-score: 4.57, p-value: 4.97E-06
HumanCytoSNP-12
Z-score: 2.06, p-value: 3.92E-02
PRS: Neuroticism
n=8513
n=845
n=169
n=61
n=20
n=3638
n=386
n=73
n=25
n=7
0.2
0.0
−0.2
−0.4
−0.6
not at all
a little bit
somewhat
quite a lot
very much
Had pain in upper back

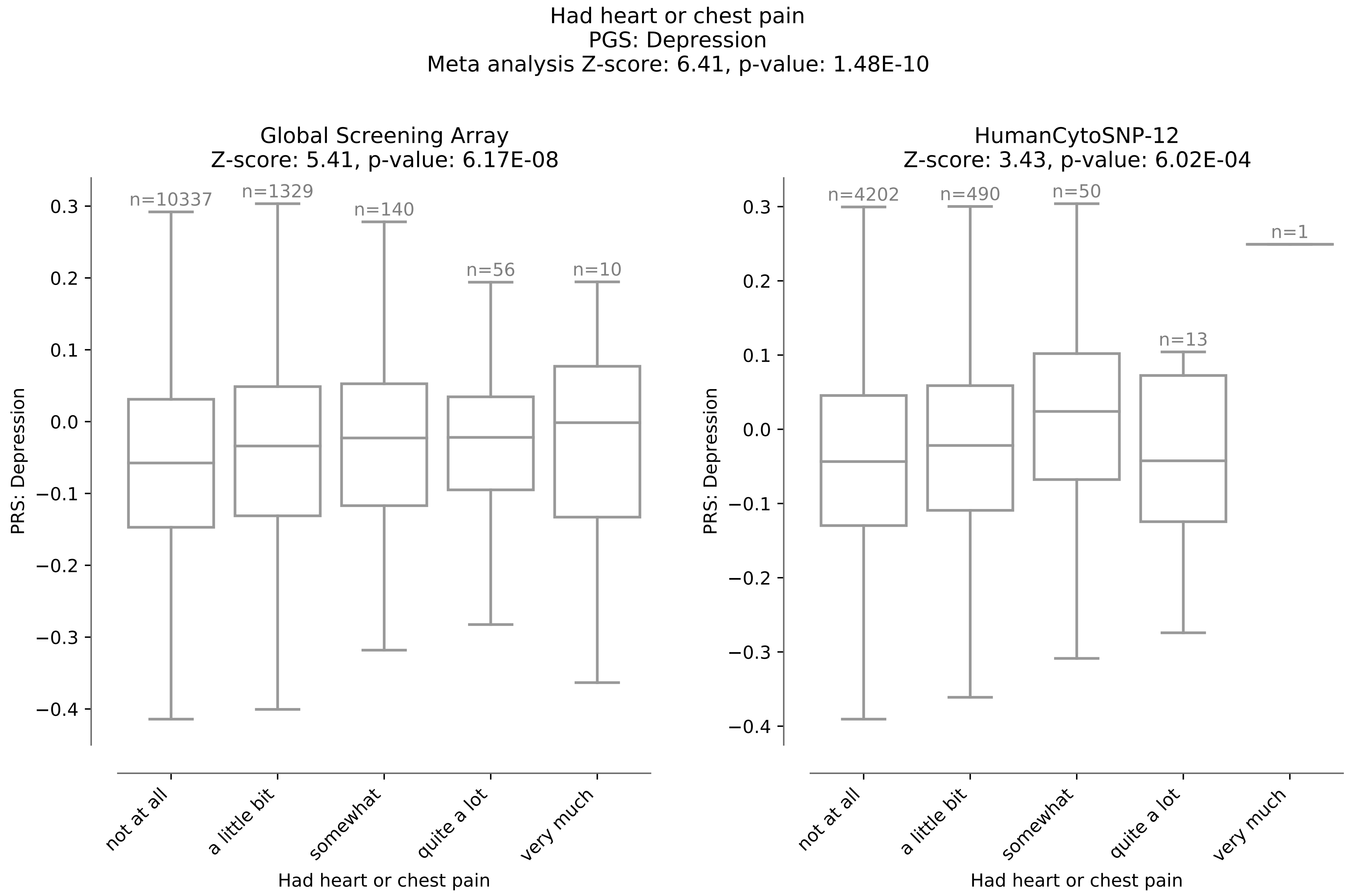

Had heart or chest pain
PGS: Depression
Meta analysis Z-score: 6.41, p-value: 1.48E-10
Global Screening Array
Z-score: 5.41, p-value: 6.17E-08
HumanCytoSNP-12
Z-score: 3.43, p-value: 6.02E-04
PRS: Depression
n=10337
n=1329
n=140
n=56
n=10
n=4202
n=490
n=50
n=13
n=1
0.3
0.2
0.1
0.0
−0.1
−0.2
−0.3
−0.4
not at all
a little bit
somewhat
quite a lot
very much
Had heart or chest pain

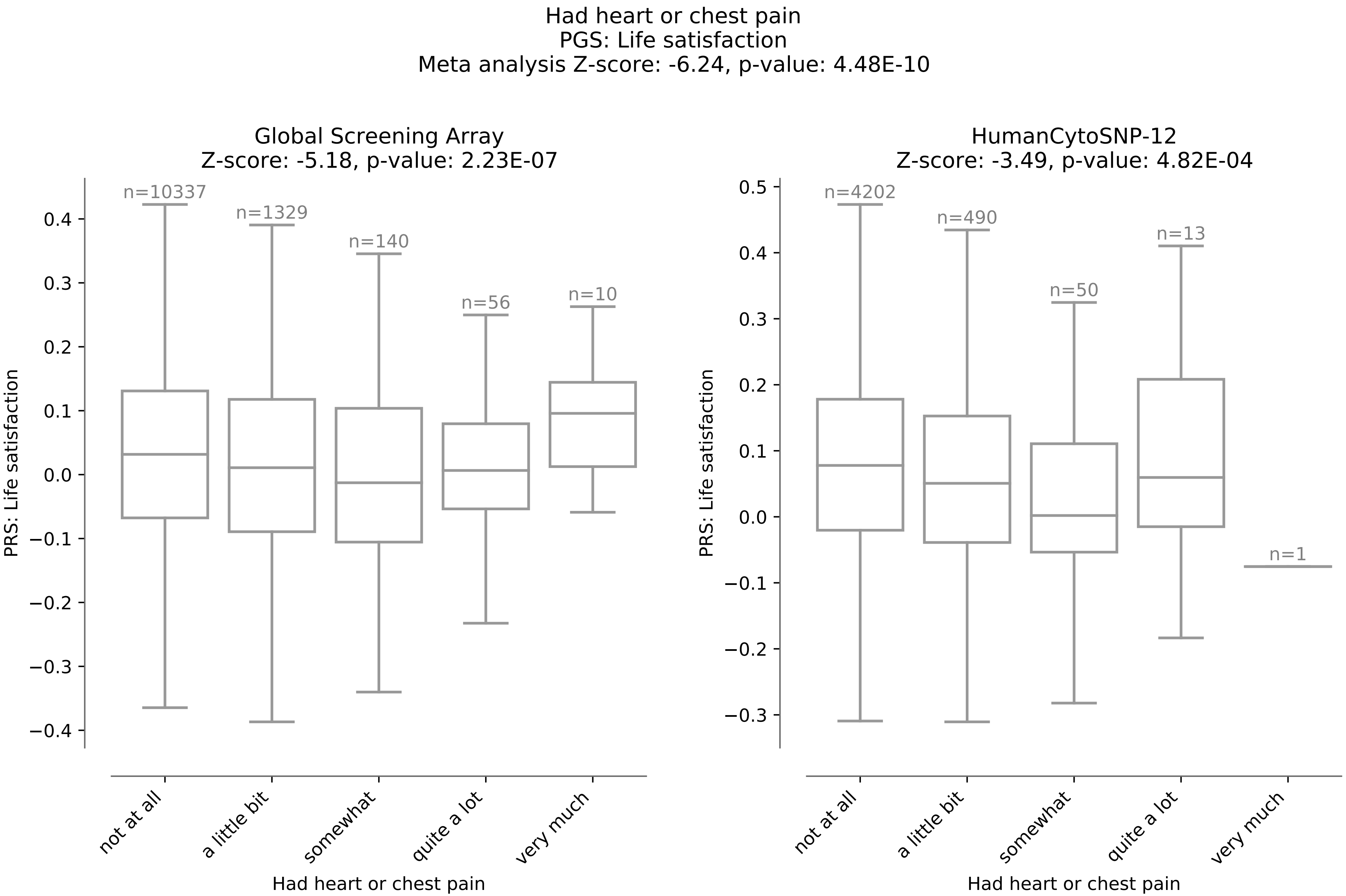
Had heart or chest pain
PGS: Life satisfaction
Meta analysis Z-score: -6.24, p-value: 4.48E-10
Global Screening Array
Z-score: -5.18, p-value: 2.23E-07
n=10337
n=1329
n=140
n=56
n=10
0.4
0.3
0.2
0.1
0.0
−0.1
−0.2
−0.3
−0.4
PRS: Life satisfaction
not at all
a little bit
somewhat
quite a lot
very much
Had heart or chest pain
HumanCytoSNP-12
Z-score: -3.49, p-value: 4.82E-04
n=4202
n=490
n=50
n=13
n=1
0.5
0.4
0.3
0.2
0.1
0.0
−0.1
−0.2
−0.3
PRS: Life satisfaction
not at all
a little bit
somewhat
quite a lot
very much
Had heart or chest pain

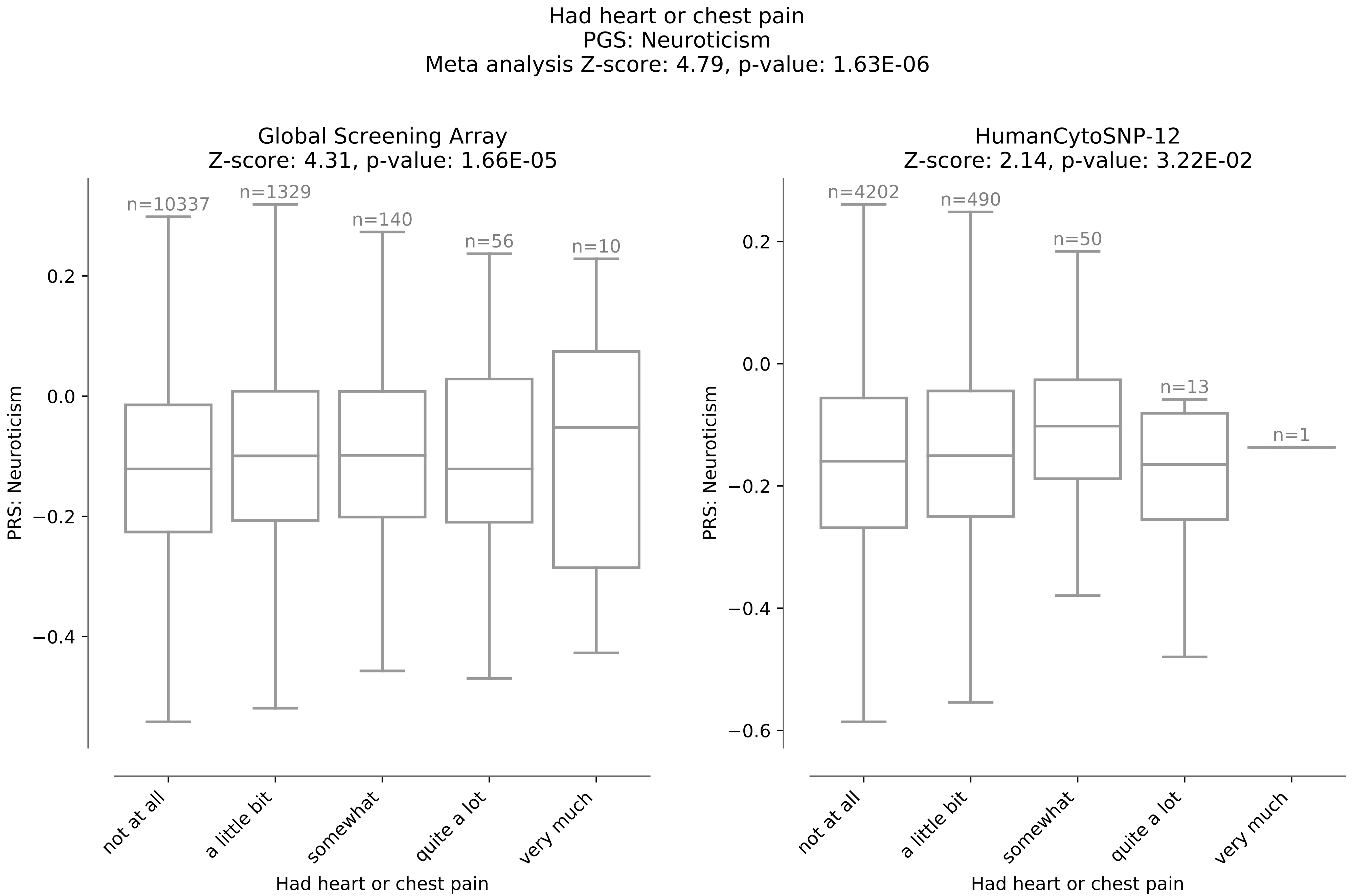
Had heart or chest pain
PGS: Neuroticism
Meta analysis Z-score: 4.79, p-value: 1.63E-06
Global Screening Array
Z-score: 4.31, p-value: 1.66E-05
n=10337
n=1329
n=140
n=56
n=10
0.2
0.0
−0.2
−0.4
PRS: Neuroticism
not at all
a little bit
somewhat
quite a lot
very much
Had heart or chest pain
HumanCytoSNP-12
Z-score: 2.14, p-value: 3.22E-02
n=4202
n=490
n=50
n=13
n=1
0.2
0.0
−0.2
−0.4
−0.6
PRS: Neuroticism
not at all
a little bit
somewhat
quite a lot
very much
Had heart or chest pain

Had heart or chest pain
PGS: Schizophrenia
Meta analysis Z-score: 5.66, p-value: 1.48E-08

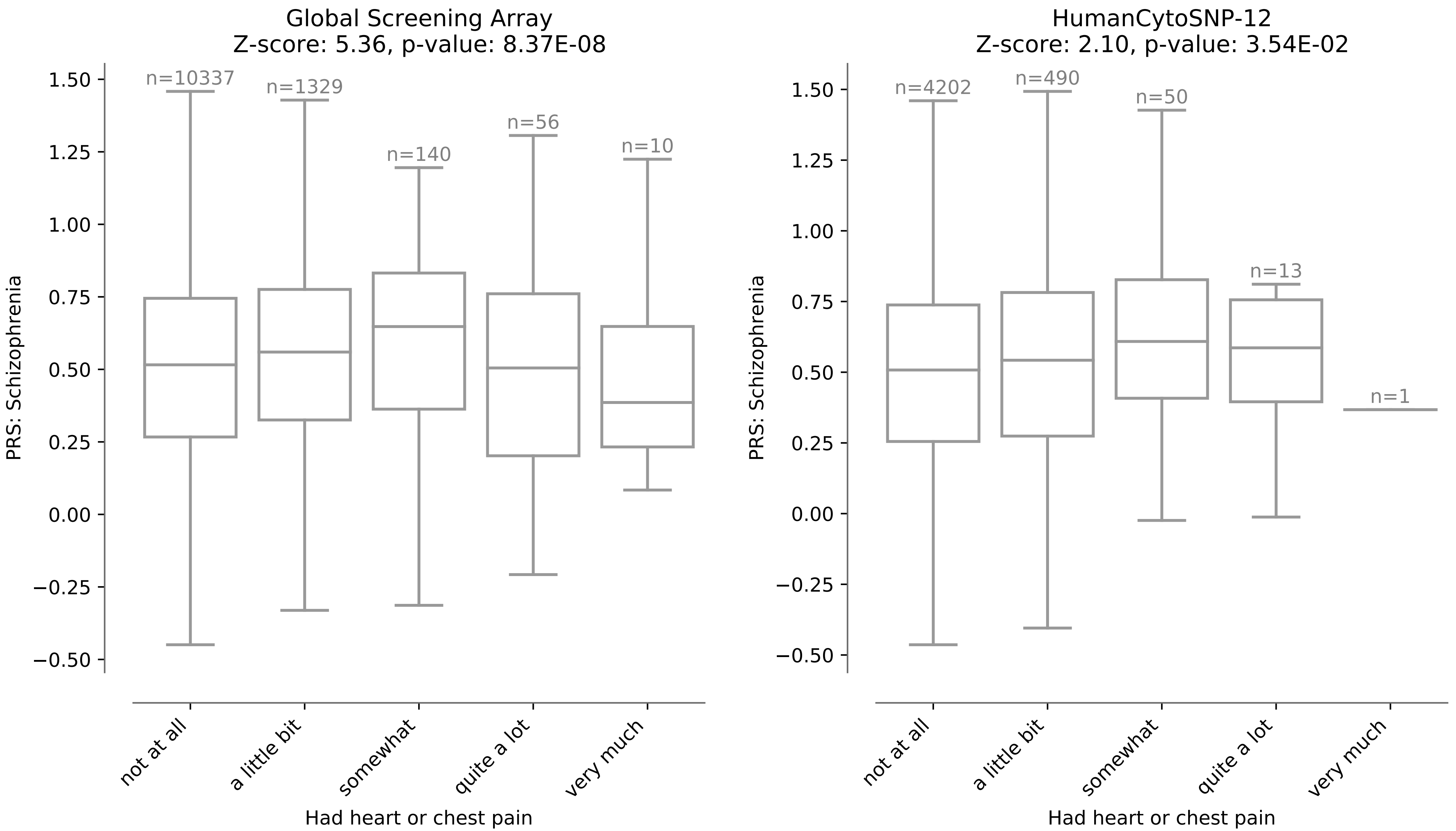

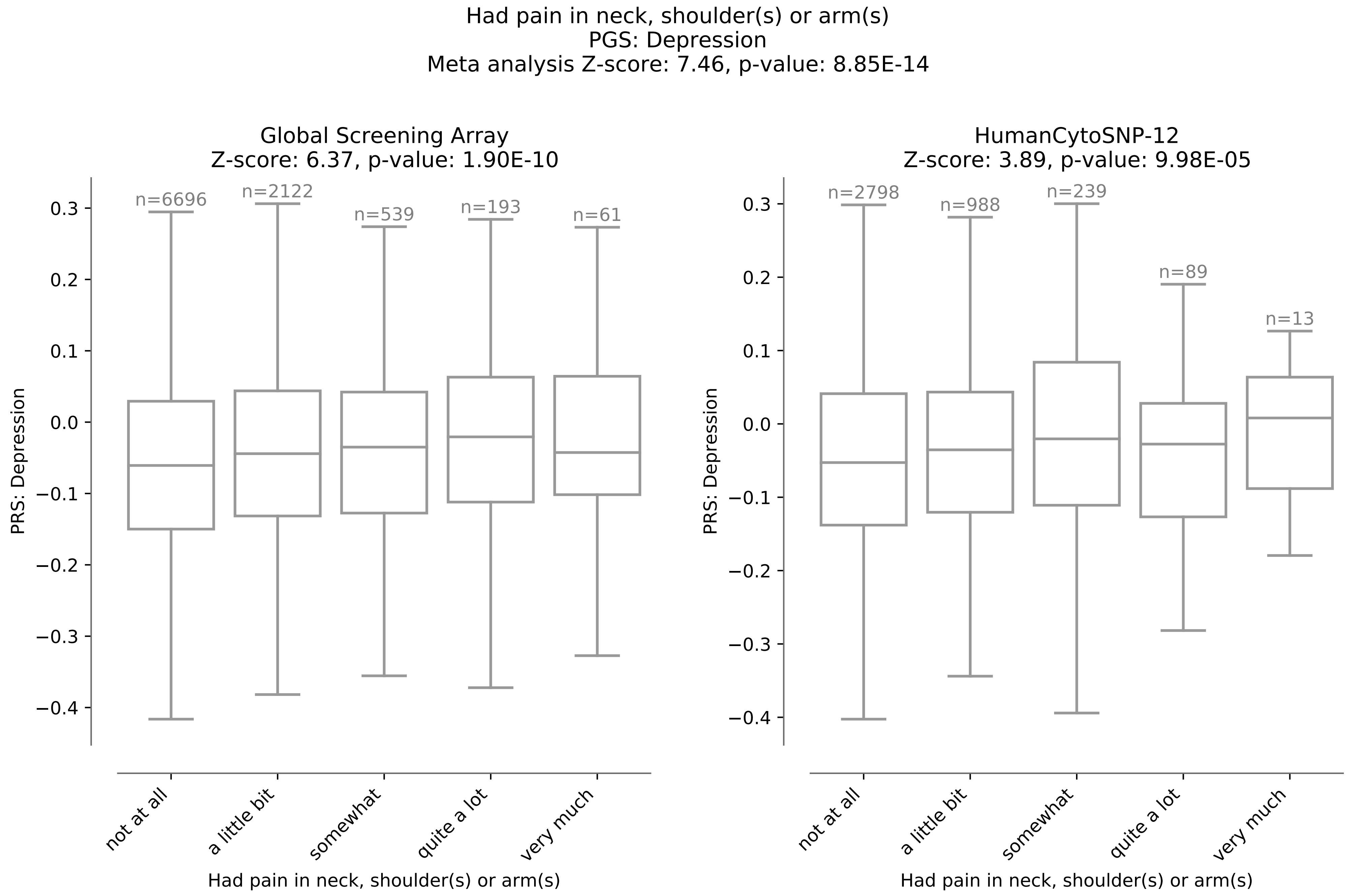
Had pain in neck, shoulder(s) or arm(s)
PGS: Depression
Meta analysis Z-score: 7.46, p-value: 8.85E-14
Global Screening Array
Z-score: 6.37, p-value: 1.90E-10
HumanCytoSNP-12
Z-score: 3.89, p-value: 9.98E-05
PRS: Depression
n=6696
n=2122
n=539
n=193
n=61
n=2798
n=988
n=239
n=89
n=13
0.3
0.2
0.1
0.0
−0.1
−0.2
−0.3
−0.4
not at all
a little bit
somewhat
quite a lot
very much
Had pain in neck, shoulder(s) or arm(s)

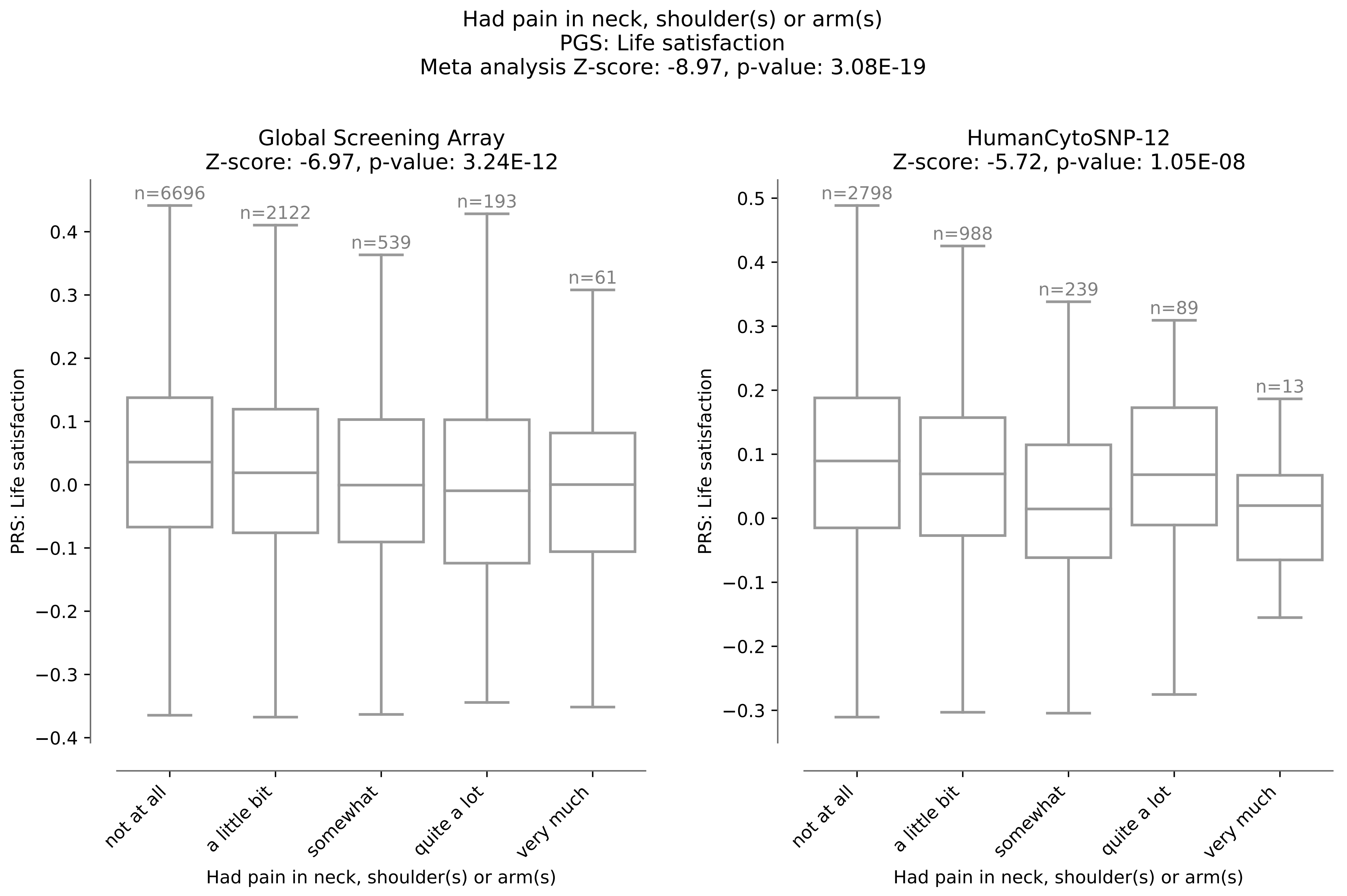
Had pain in neck, shoulder(s) or arm(s)
PGS: Life satisfaction
Meta analysis Z-score: -8.97, p-value: 3.08E-19
Global Screening Array
Z-score: -6.97, p-value: 3.24E-12
HumanCytoSNP-12
Z-score: -5.72, p-value: 1.05E-08
PRS: Life satisfaction
n=6696
n=2122
n=539
n=193
n=61
n=2798
n=988
n=239
n=89
n=13
not at all
a little bit
somewhat
quite a lot
very much
Had pain in neck, shoulder(s) or arm(s)

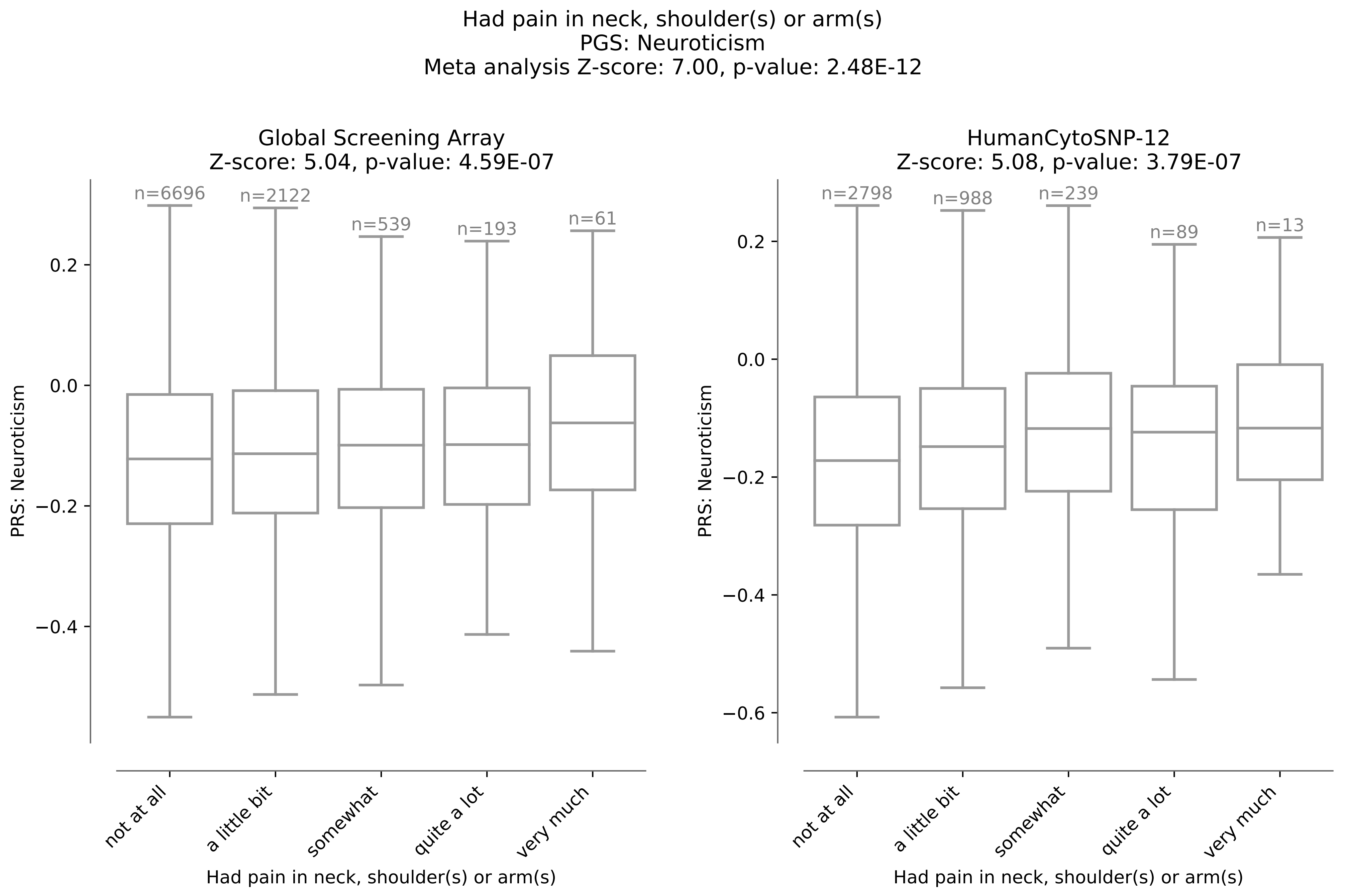
Had pain in neck, shoulder(s) or arm(s)
PGS: Neuroticism
Meta analysis Z-score: 7.00, p-value: 2.48E-12
Global Screening Array
Z-score: 5.04, p-value: 4.59E-07
n=6696
n=2122
n=539
n=193
n=61
0.2
0.0
−0.2
−0.4
PRS: Neuroticism
not at all
a little bit
somewhat
quite a lot
very much
Had pain in neck, shoulder(s) or arm(s)
HumanCytoSNP-12
Z-score: 5.08, p-value: 3.79E-07
n=2798
n=988
n=239
n=89
n=13
0.2
0.0
−0.2
−0.4
−0.6
PRS: Neuroticism
not at all
a little bit
somewhat
quite a lot
very much
Had pain in neck, shoulder(s) or arm(s)

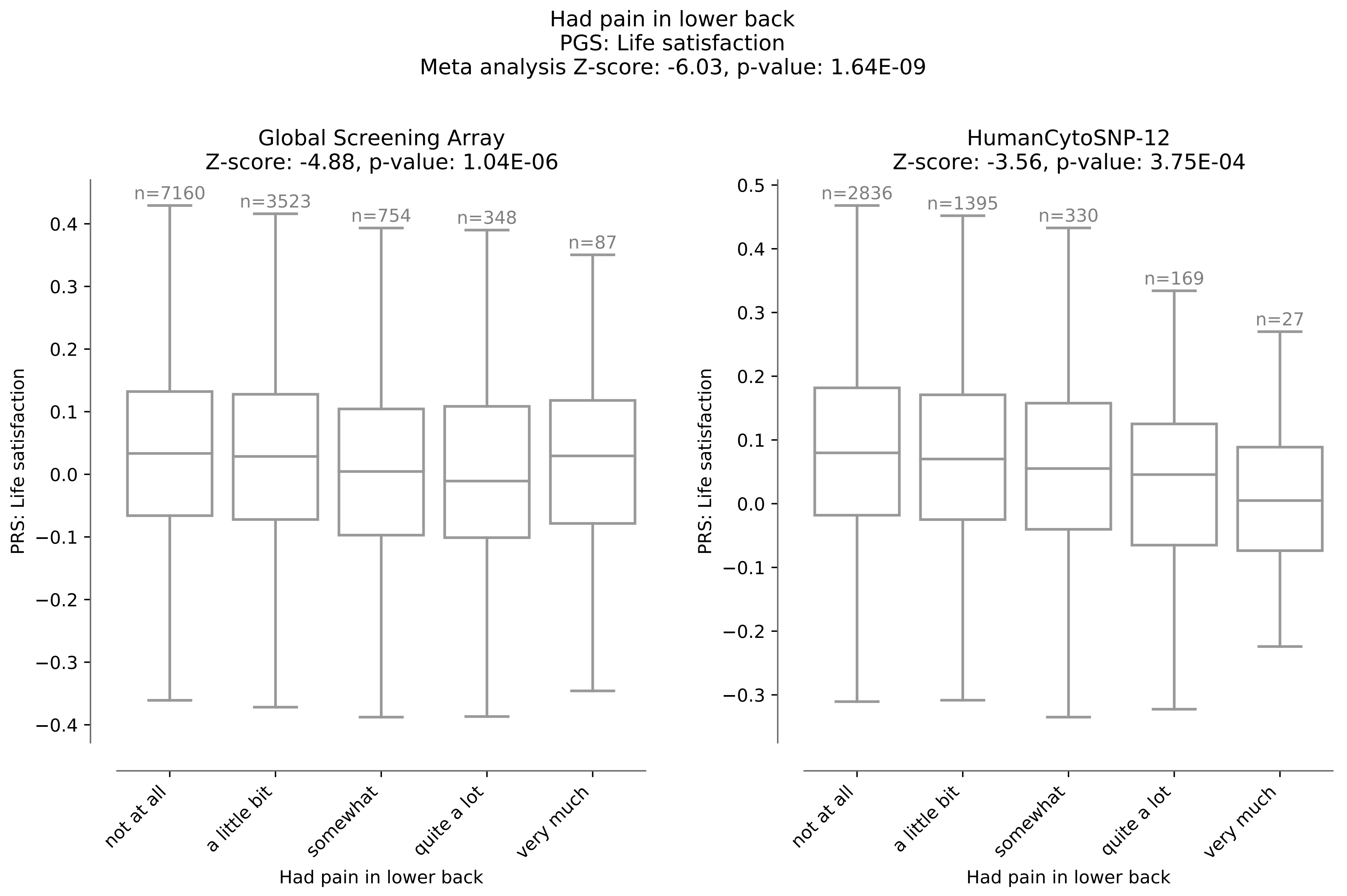
Had pain in lower back
PGS: Life satisfaction
Meta analysis Z-score: -6.03, p-value: 1.64E-09
Global Screening Array
Z-score: -4.88, p-value: 1.04E-06
HumanCytoSNP-12
Z-score: -3.56, p-value: 3.75E-04
PRS: Life satisfaction
Had pain in lower back
n=7160
n=3523
n=754
n=348
n=87
n=2836
n=1395
n=330
n=169
n=27
not at all
a little bit
somewhat
quite a lot
very much

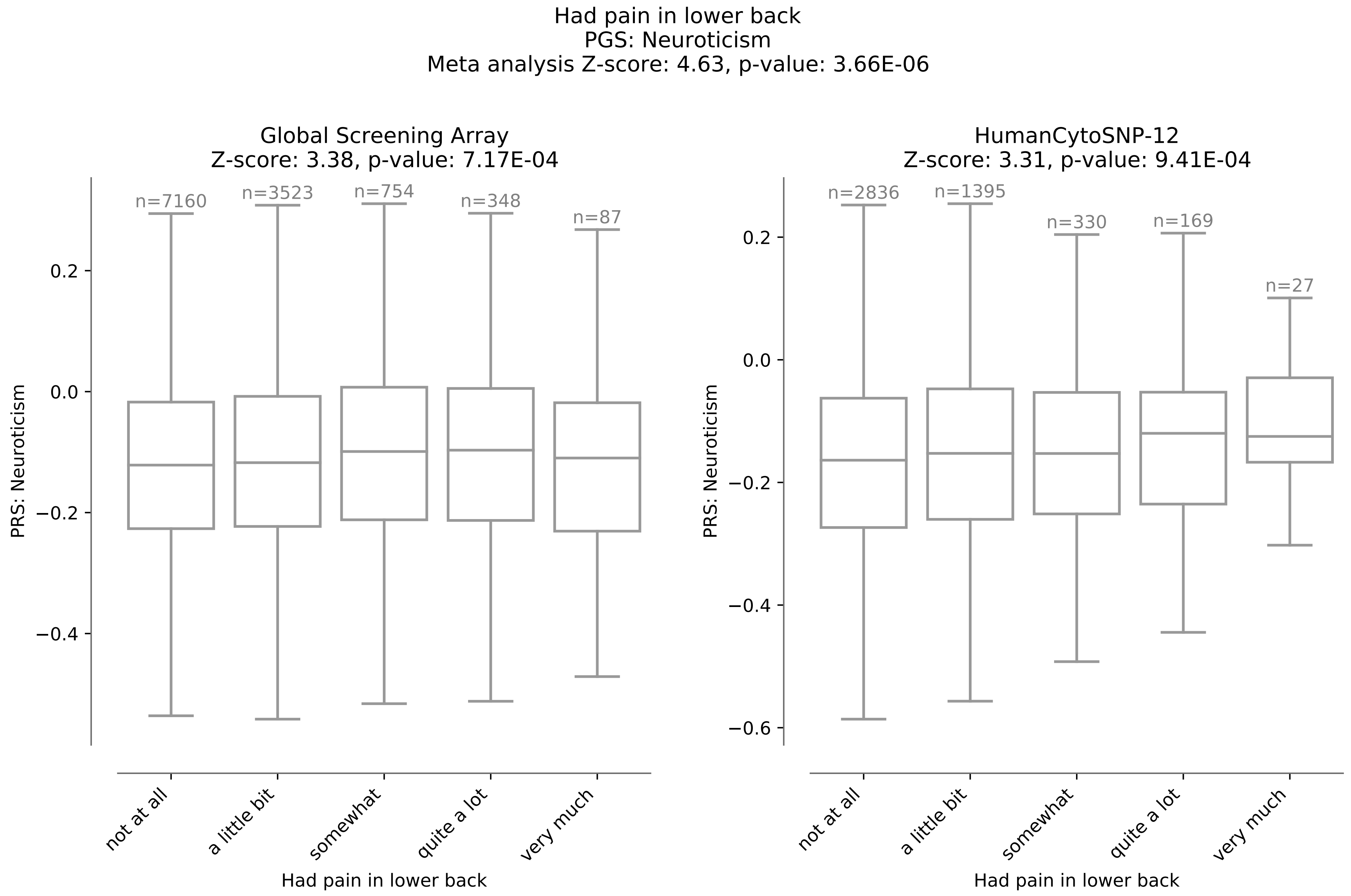
Had pain in lower back
PGS: Neuroticism
Meta analysis Z-score: 4.63, p-value: 3.66E-06
Global Screening Array
Z-score: 3.38, p-value: 7.17E-04
n=7160
n=3523
n=754
n=348
n=87
0.2
0.0
−0.2
−0.4
PRS: Neuroticism
not at all
a little bit
somewhat
quite a lot
very much
Had pain in lower back
HumanCytoSNP-12
Z-score: 3.31, p-value: 9.41E-04
n=2836
n=1395
n=330
n=169
n=27
0.2
0.0
−0.2
−0.4
−0.6
PRS: Neuroticism
not at all
a little bit
somewhat
quite a lot
very much
Had pain in lower back

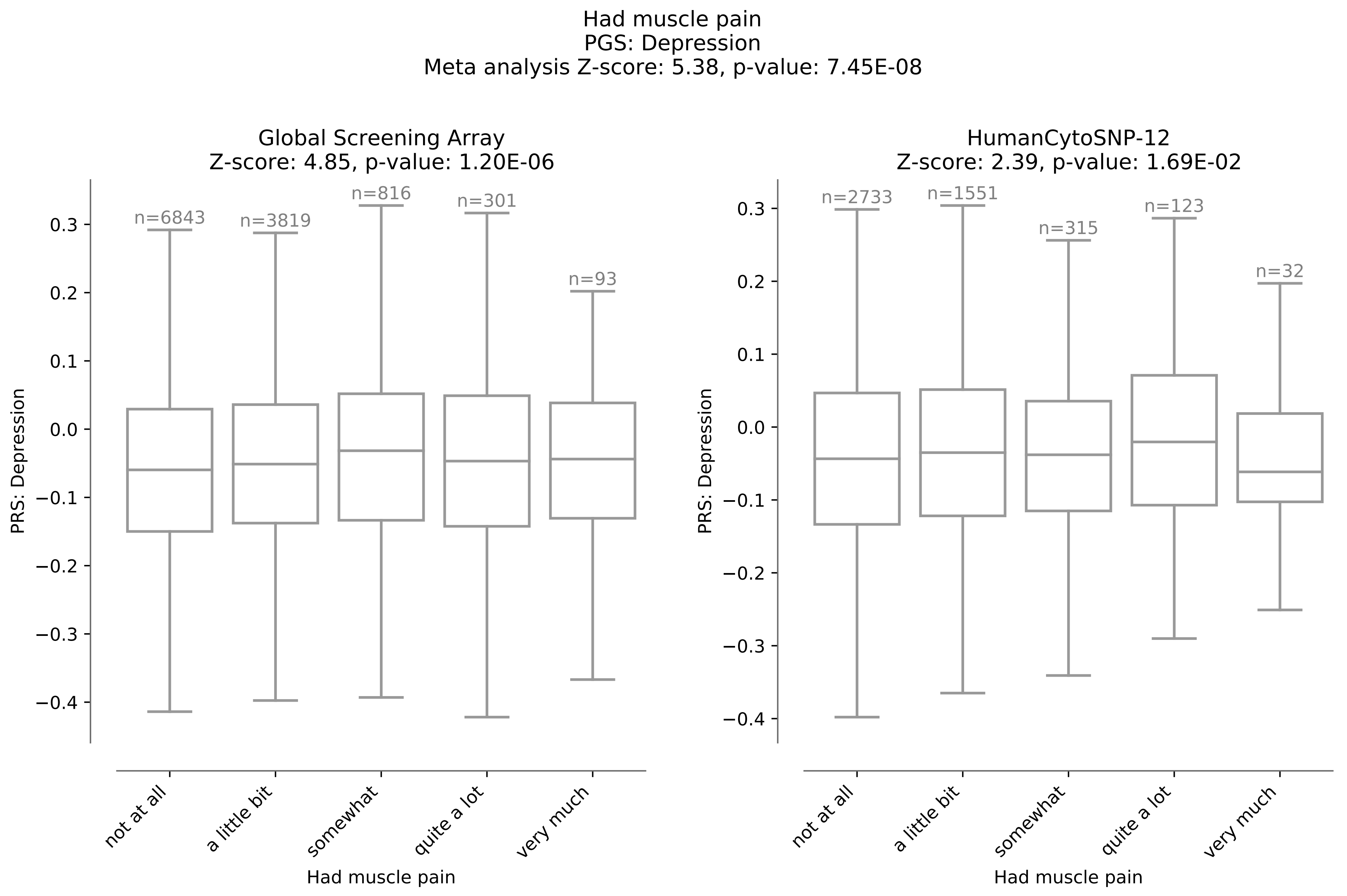

Had muscle pain
PGS: Depression
Meta analysis Z-score: 5.38, p-value: 7.45E-08
Global Screening Array
Z-score: 4.85, p-value: 1.20E-06
HumanCytoSNP-12
Z-score: 2.39, p-value: 1.69E-02
PRS: Depression
n=6843
n=3819
n=816
n=301
n=93
n=2733
n=1551
n=315
n=123
n=32
0.3
0.2
0.1
0.0
−0.1
−0.2
−0.3
−0.4
not at all
a little bit
somewhat
quite a lot
very much
Had muscle pain

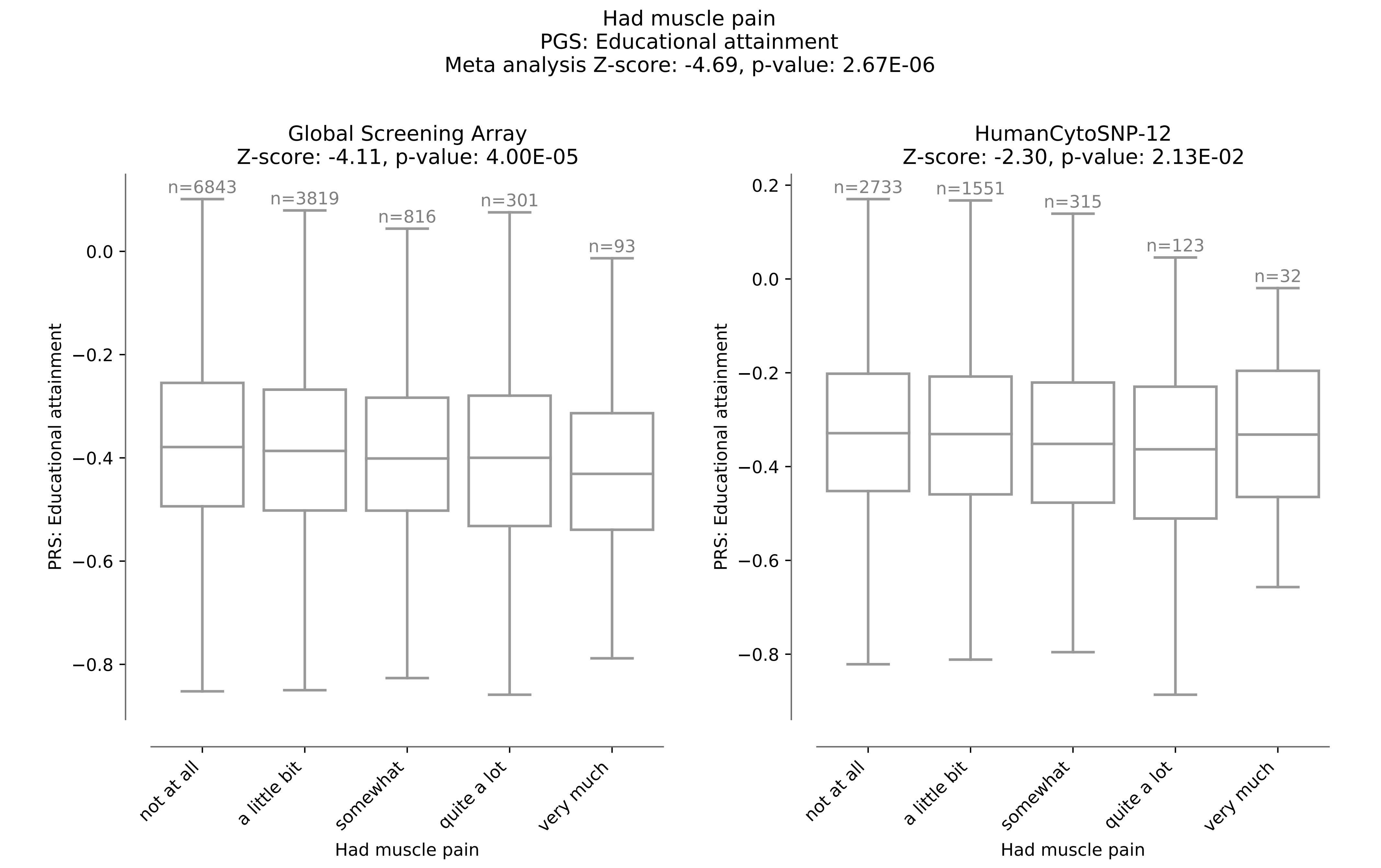
Had muscle pain
PGS: Educational attainment
Meta analysis Z-score: -4.69, p-value: 2.67E-06
Global Screening Array
Z-score: -4.11, p-value: 4.00E-05
n=6843
n=3819
n=816
n=301
n=93
PRS: Educational attainment
0.0
−0.2
−0.4
−0.6
−0.8
not at all
a little bit
somewhat
quite a lot
very much
Had muscle pain
HumanCytoSNP-12
Z-score: -2.30, p-value: 2.13E-02
n=2733
n=1551
n=315
n=123
n=32
PRS: Educational attainment
0.2
0.0
−0.2
−0.4
−0.6
−0.8
not at all
a little bit
somewhat
quite a lot
very much
Had muscle pain

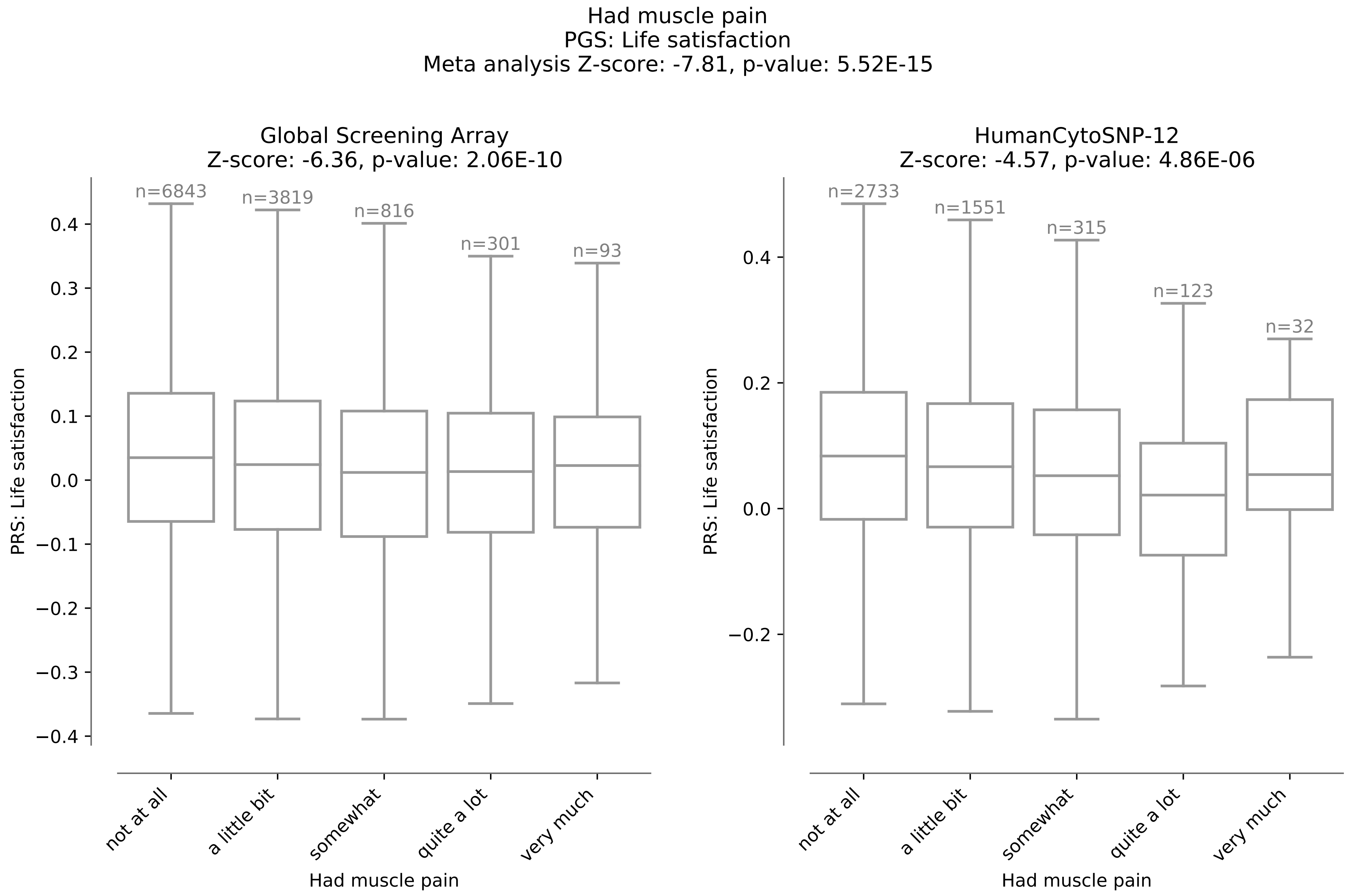
Had muscle pain
PGS: Life satisfaction
Meta analysis Z-score: -7.81, p-value: 5.52E-15
Global Screening Array
Z-score: -6.36, p-value: 2.06E-10
HumanCytoSNP-12
Z-score: -4.57, p-value: 4.86E-06
n=6843
n=3819
n=816
n=301
n=93
n=2733
n=1551
n=315
n=123
n=32
PRS: Life satisfaction
0.4
0.3
0.2
0.1
0.0
−0.1
−0.2
−0.3
−0.4
not at all
a little bit
somewhat
quite a lot
very much
Had muscle pain

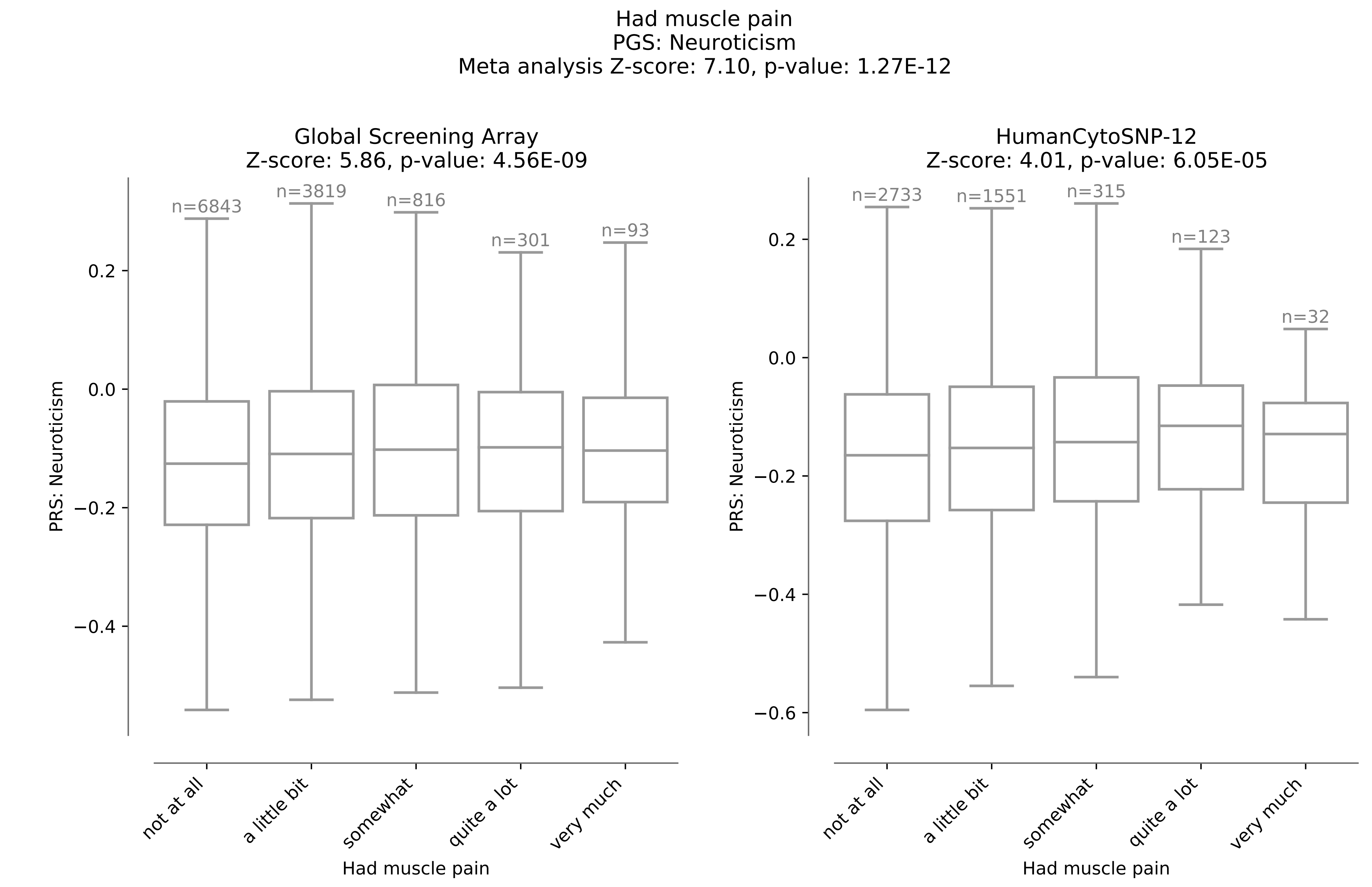

Had muscle pain
PGS: Neuroticism
Meta analysis Z-score: 7.10, p-value: 1.27E-12
Global Screening Array
Z-score: 5.86, p-value: 4.56E-09
HumanCytoSNP-12
Z-score: 4.01, p-value: 6.05E-05
n=6843
n=3819
n=816
n=301
n=93
n=2733
n=1551
n=315
n=123
n=32
PRS: Neuroticism
0.2
0.0
−0.2
−0.4
−0.6
not at all
a little bit
somewhat
quite a lot
very much
Had muscle pain

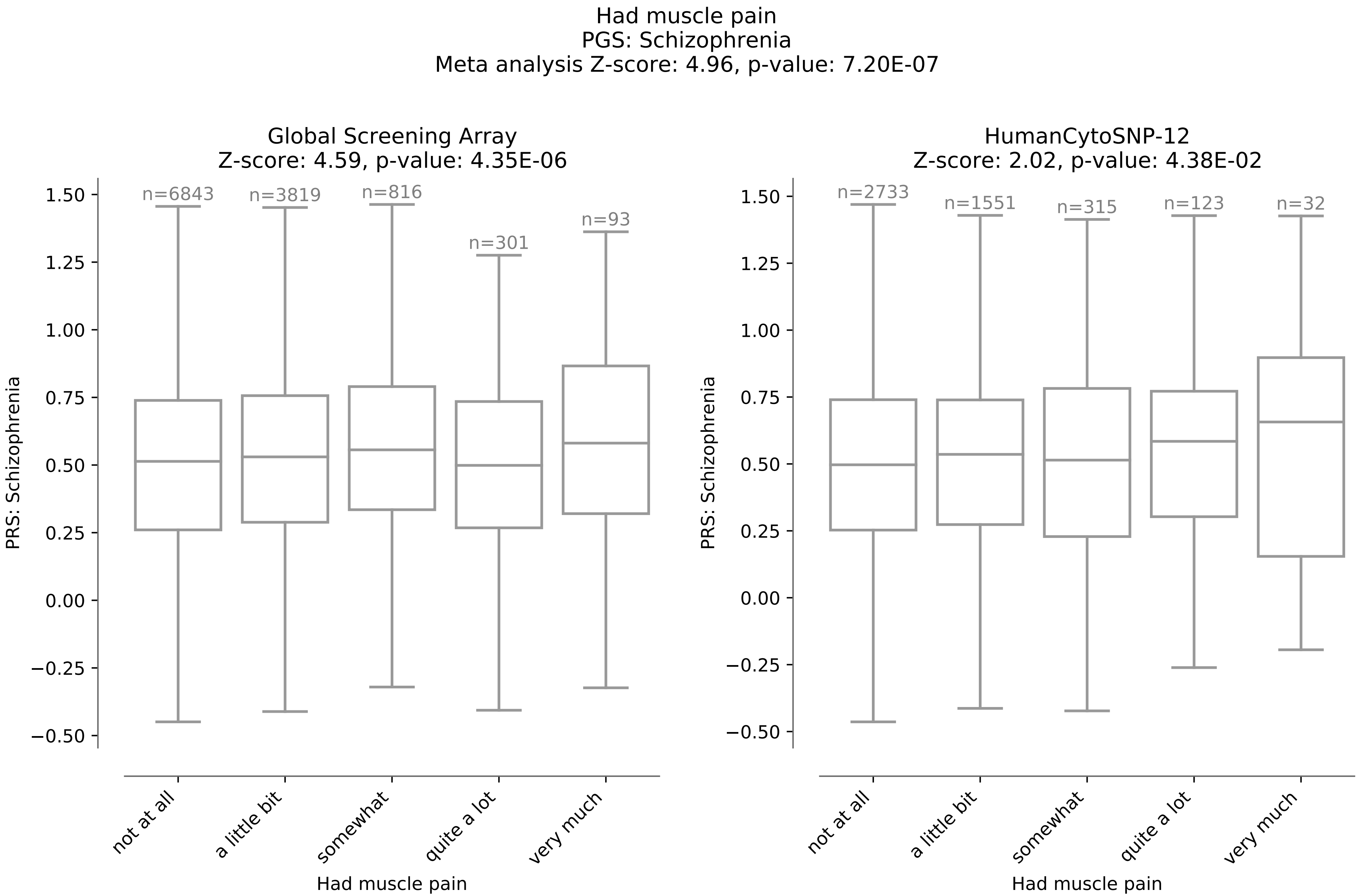

Had muscle pain
PGS: Schizophrenia
Meta analysis Z-score: 4.96, p-value: 7.20E-07
Global Screening Array
Z-score: 4.59, p-value: 4.35E-06
HumanCytoSNP-12
Z-score: 2.02, p-value: 4.38E-02
PRS: Schizophrenia
n=6843
n=3819
n=816
n=301
n=93
n=2733
n=1551
n=315
n=123
n=32
1.50
1.25
1.00
0.75
0.50
0.25
0.00
−0.25
−0.50
not at all
a little bit
somewhat
quite a lot
very much
Had muscle pain

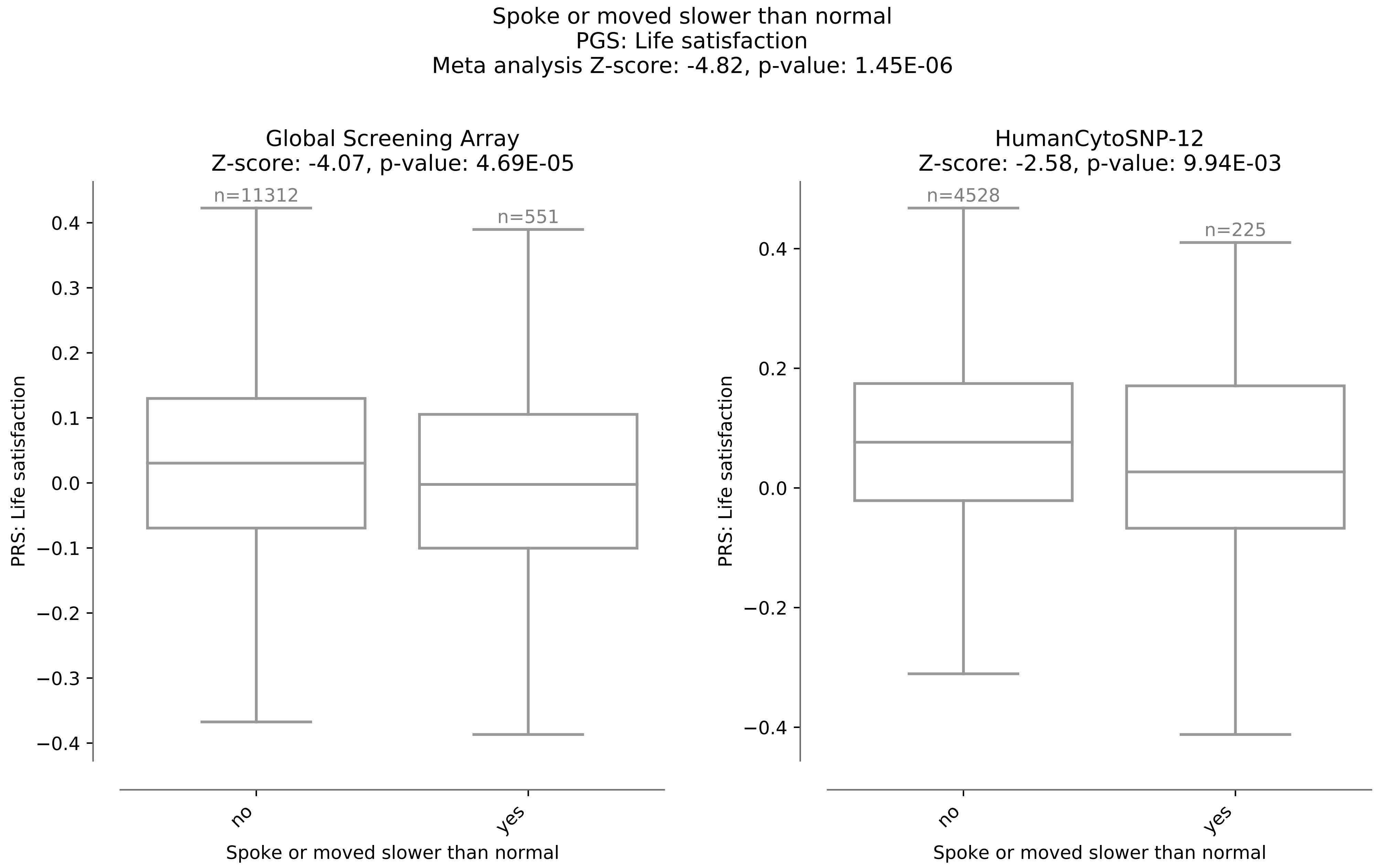
Spoke or moved slower than normal
PGS: Life satisfaction
Meta analysis Z-score: -4.82, p-value: 1.45E-06
Global Screening Array
Z-score: -4.07, p-value: 4.69E-05
n=11312
n=551
0.4
0.3
0.2
0.1
0.0
−0.1
−0.2
−0.3
−0.4
PRS: Life satisfaction
no
yes
Spoke or moved slower than normal
HumanCytoSNP-12
Z-score: -2.58, p-value: 9.94E-03
n=4528
n=225
0.4
0.2
0.0
−0.2
−0.4
PRS: Life satisfaction
no
yes
Spoke or moved slower than normal

Perceiving others as a threat to personal well-being
PGS: Educational attainment
Meta analysis Z-score: -7.44, p-value: 9.79E-14

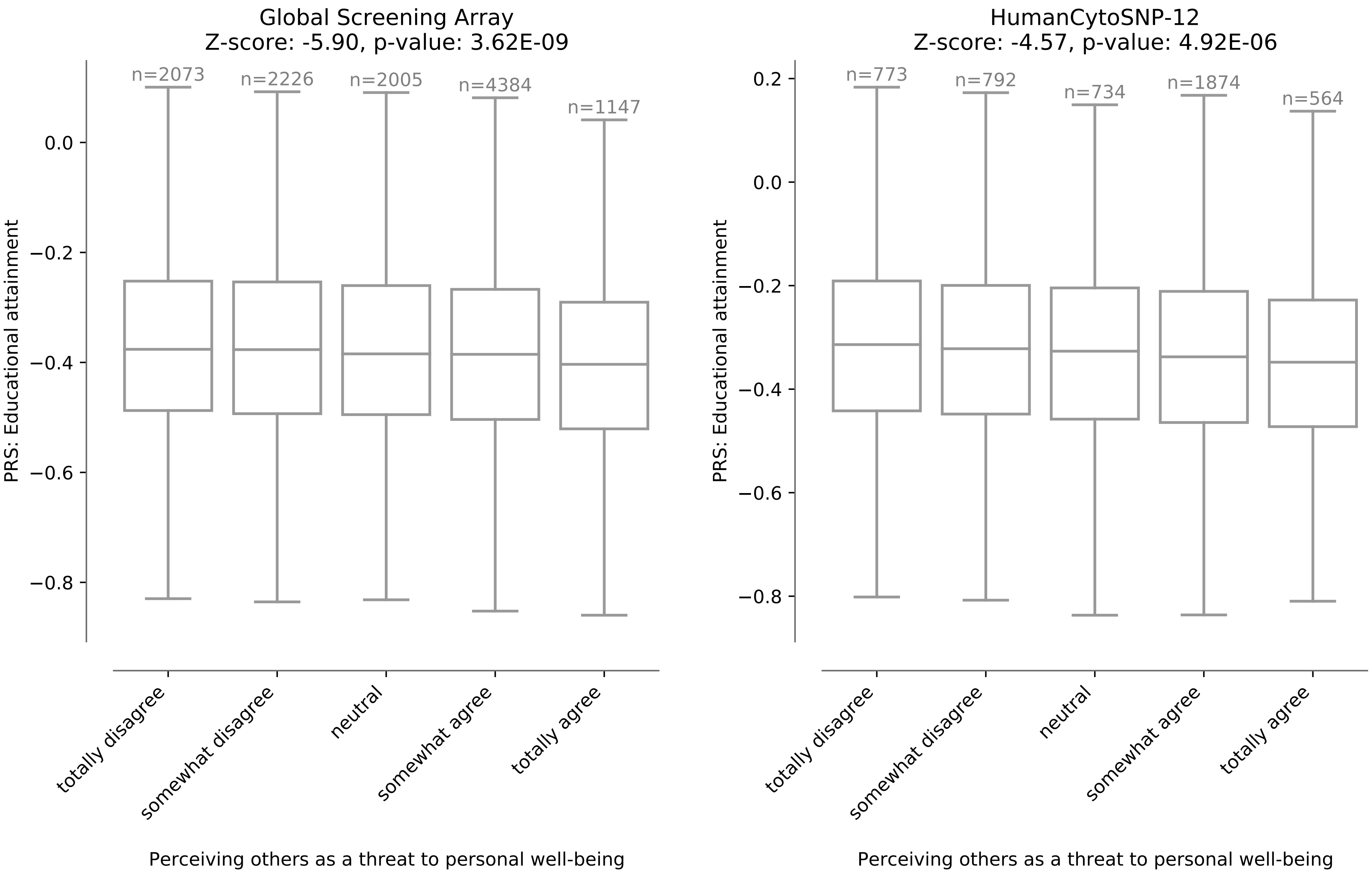

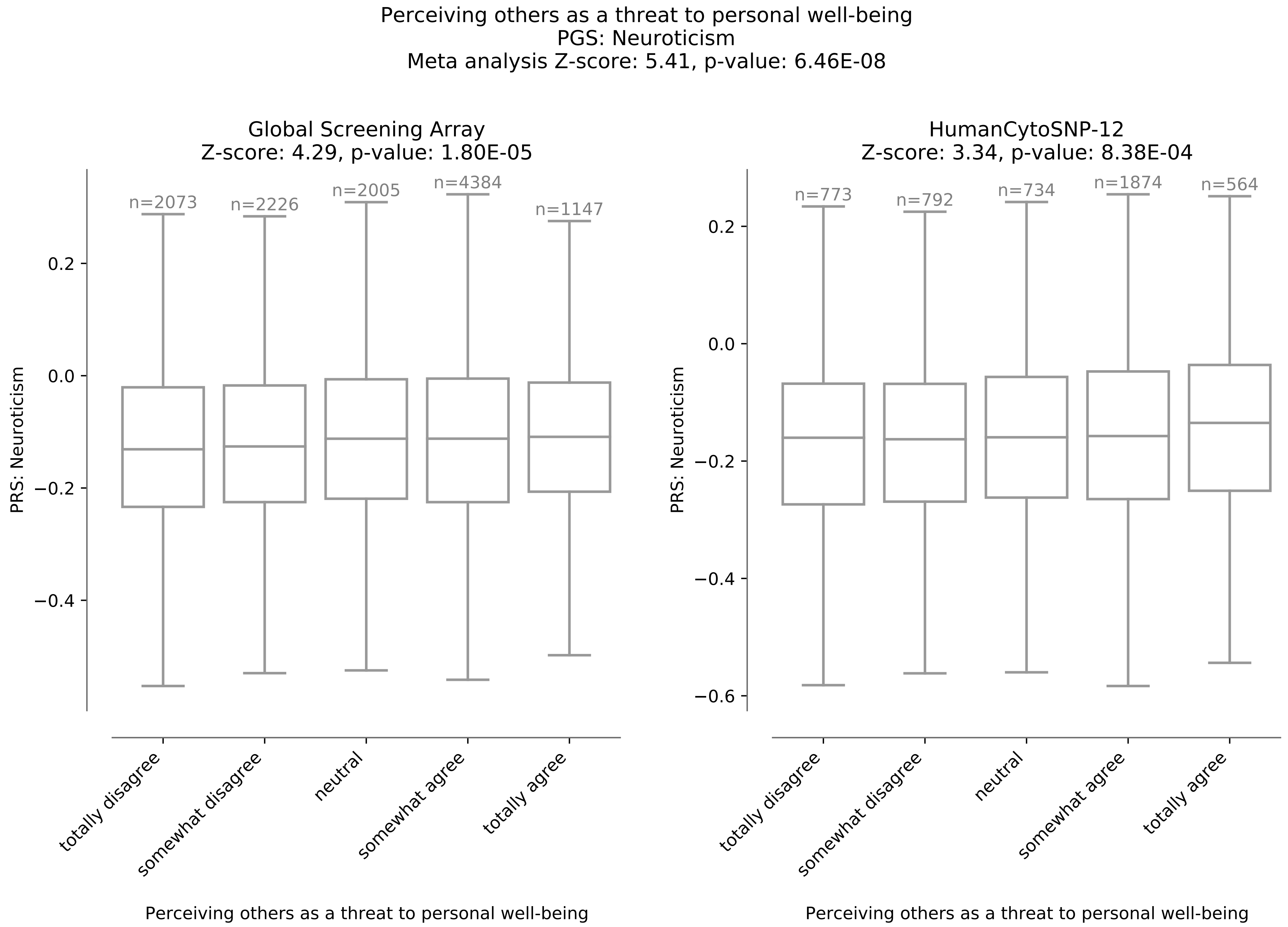
Perceiving others as a threat to personal well-being
PGS: Neuroticism
Meta analysis Z-score: 5.41, p-value: 6.46E-08
Global Screening Array
Z-score: 4.29, p-value: 1.80E-05
HumanCytoSNP-12
Z-score: 3.34, p-value: 8.38E-04
PRS: Neuroticism
n=2073
n=2226
n=2005
n=4384
n=1147
n=773
n=792
n=734
n=1874
n=564
0.2
0.0
−0.2
−0.4
−0.6
totally disagree
somewhat disagree
neutral
somewhat agree
totally agree
Perceiving others as a threat to personal well-being

Being able to comply with the 1.5 meter rule when visited at home
PGS: Educational attainment
Meta analysis Z-score: -4.52, p-value: 6.20E-06

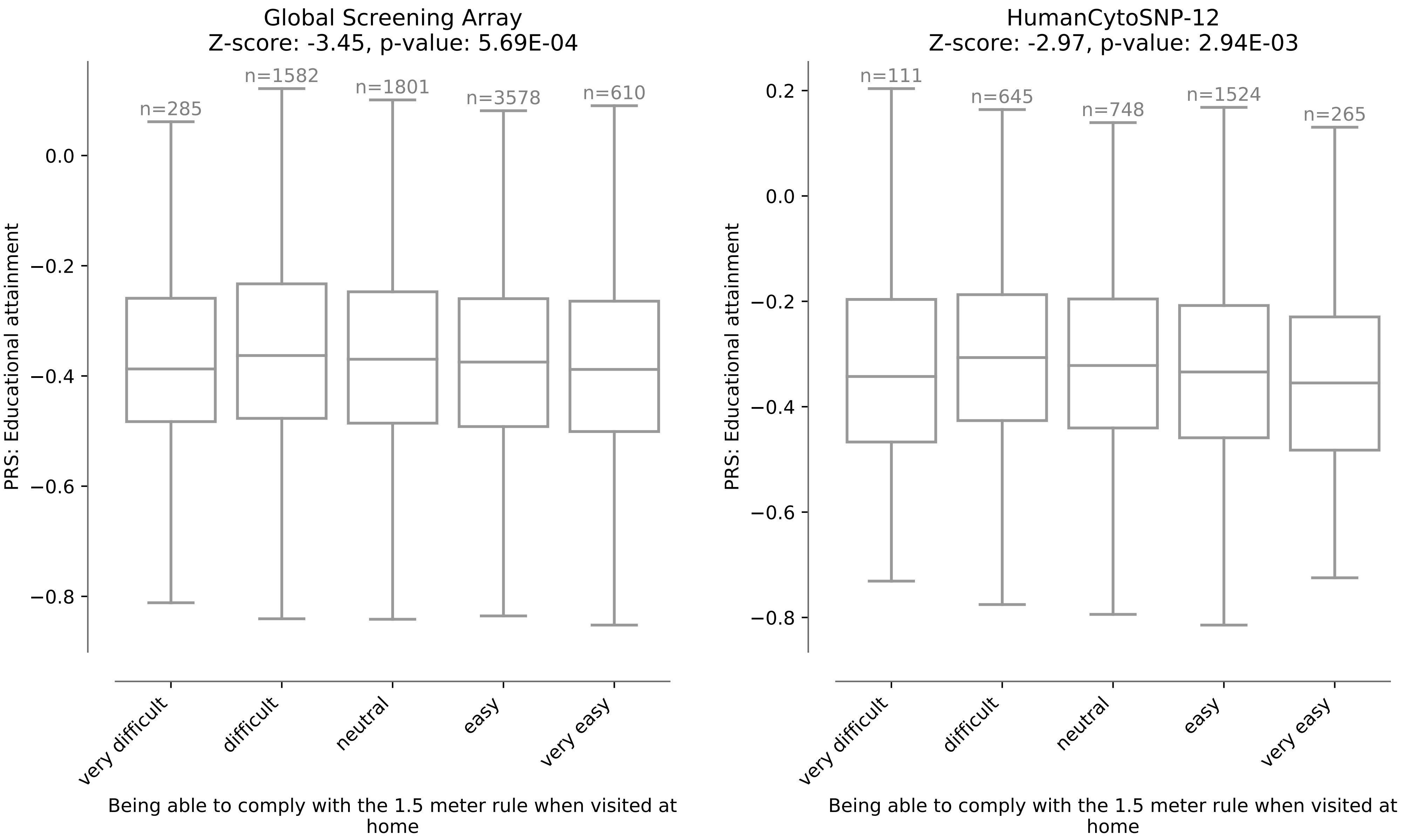

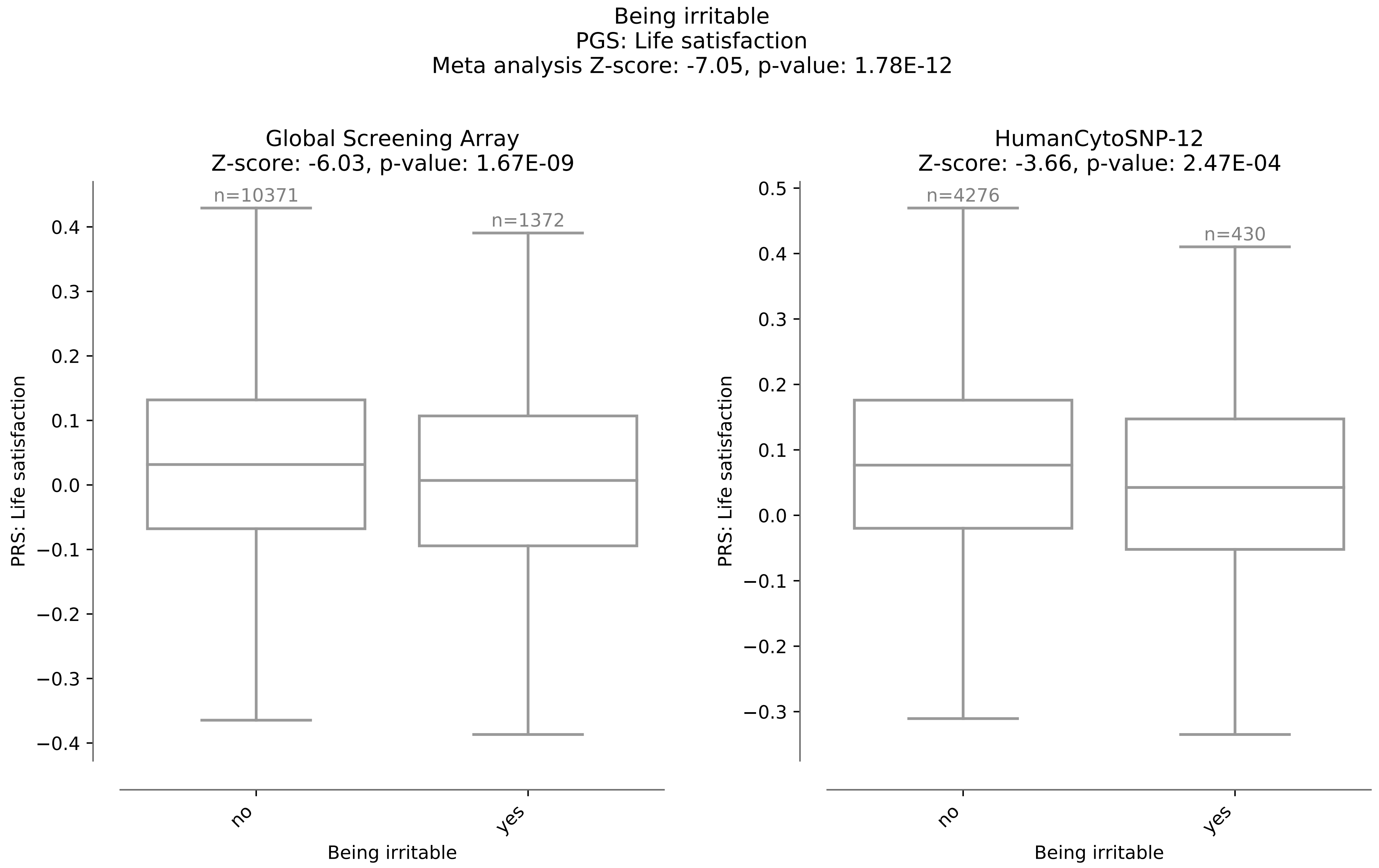
Being irritable
PGS: Life satisfaction
Meta analysis Z-score: -7.05, p-value: 1.78E-12
Global Screening Array
Z-score: -6.03, p-value: 1.67E-09
n=10371
n=1372
PRS: Life satisfaction
0.4
0.3
0.2
0.1
0.0
−0.1
−0.2
−0.3
−0.4
no
yes
Being irritable
HumanCytoSNP-12
Z-score: -3.66, p-value: 2.47E-04
n=4276
n=430
PRS: Life satisfaction
0.5
0.4
0.3
0.2
0.1
0.0
−0.1
−0.2
−0.3
no
yes
Being irritable

Being irritable
PGS: Neuroticism
Meta analysis Z-score: 5.36, p-value: 8.18E-08

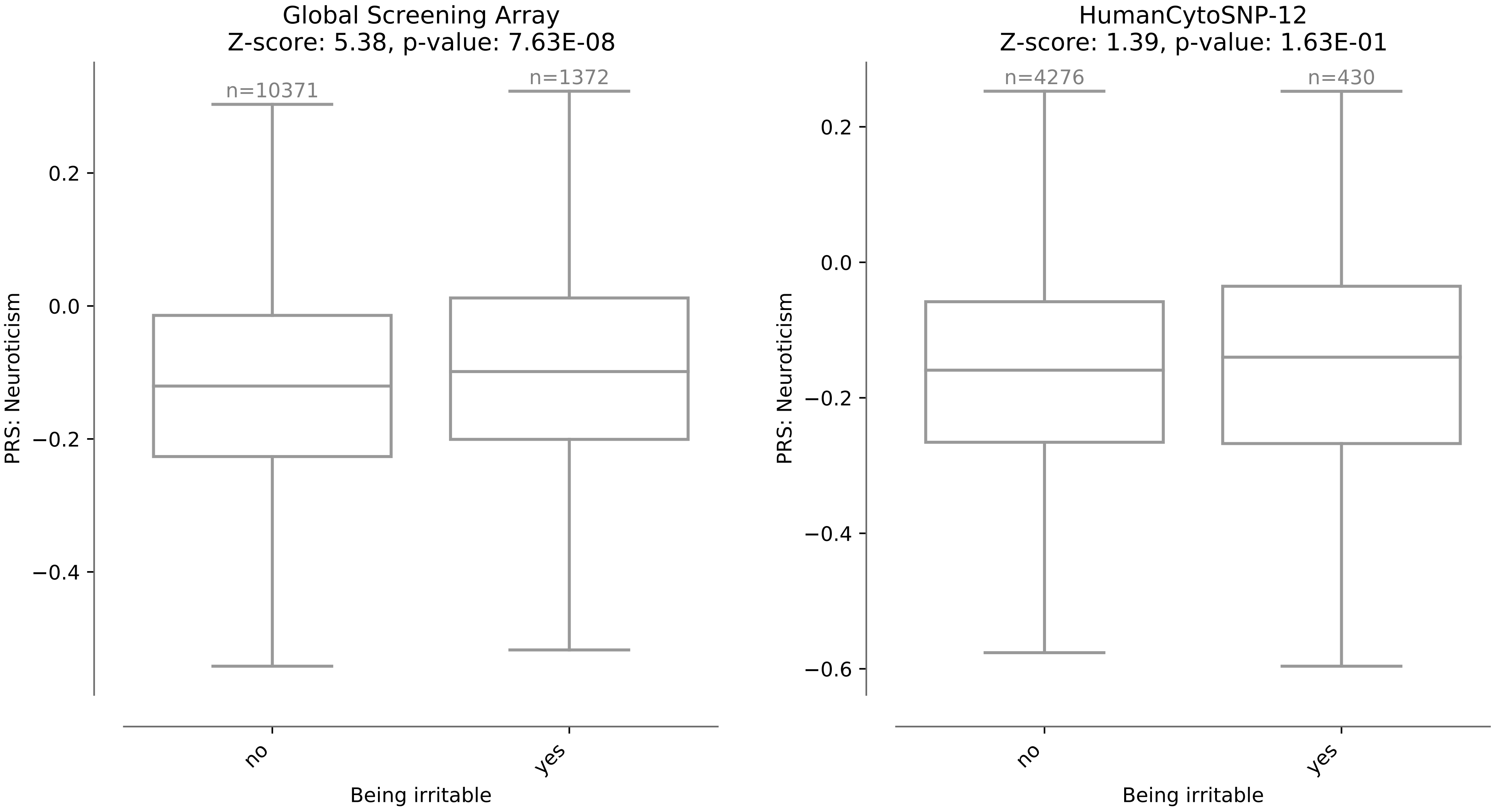

Felt tense
PGS: Depression
Meta analysis Z-score: 5.82, p-value: 5.91E-09

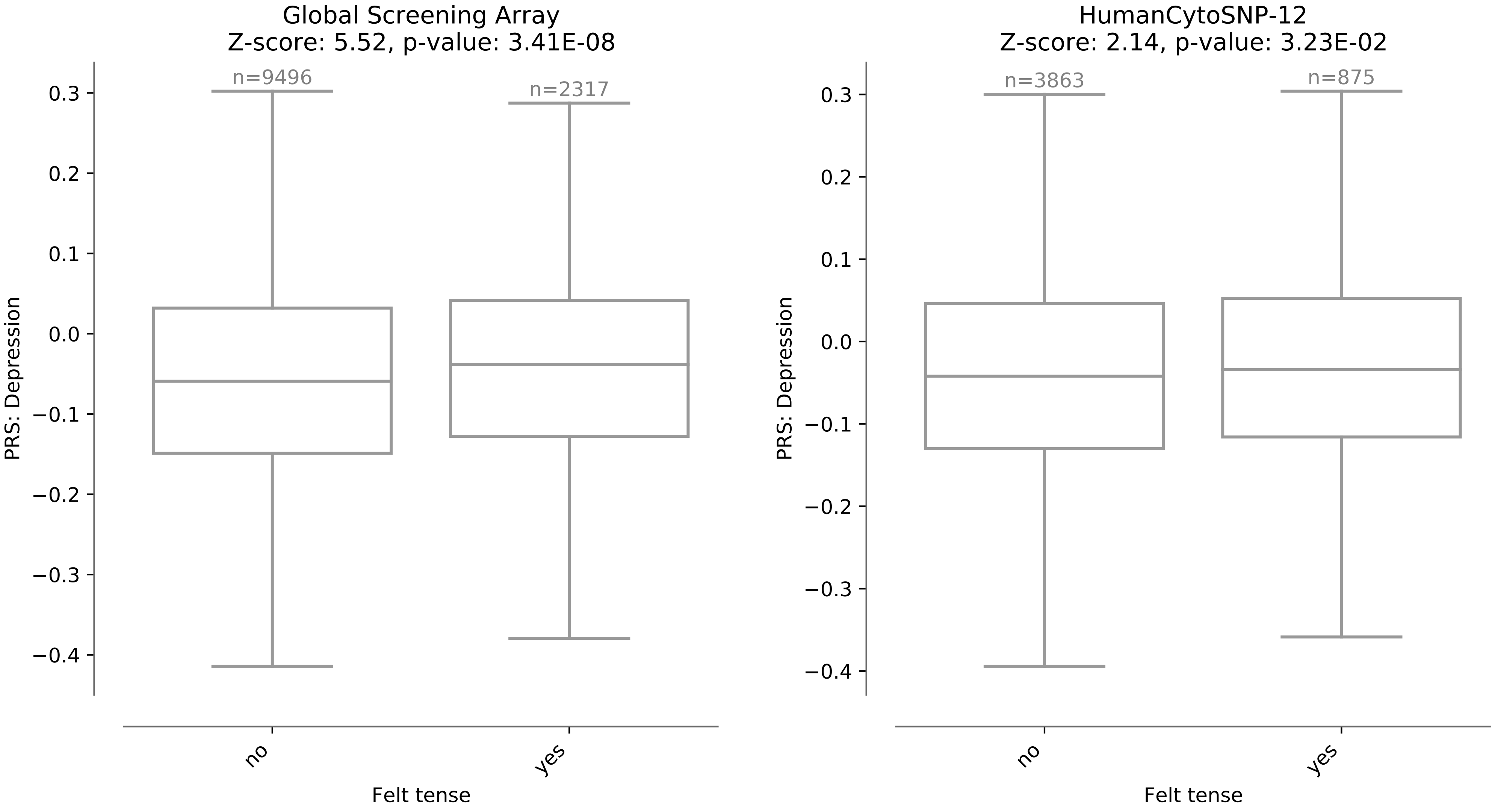

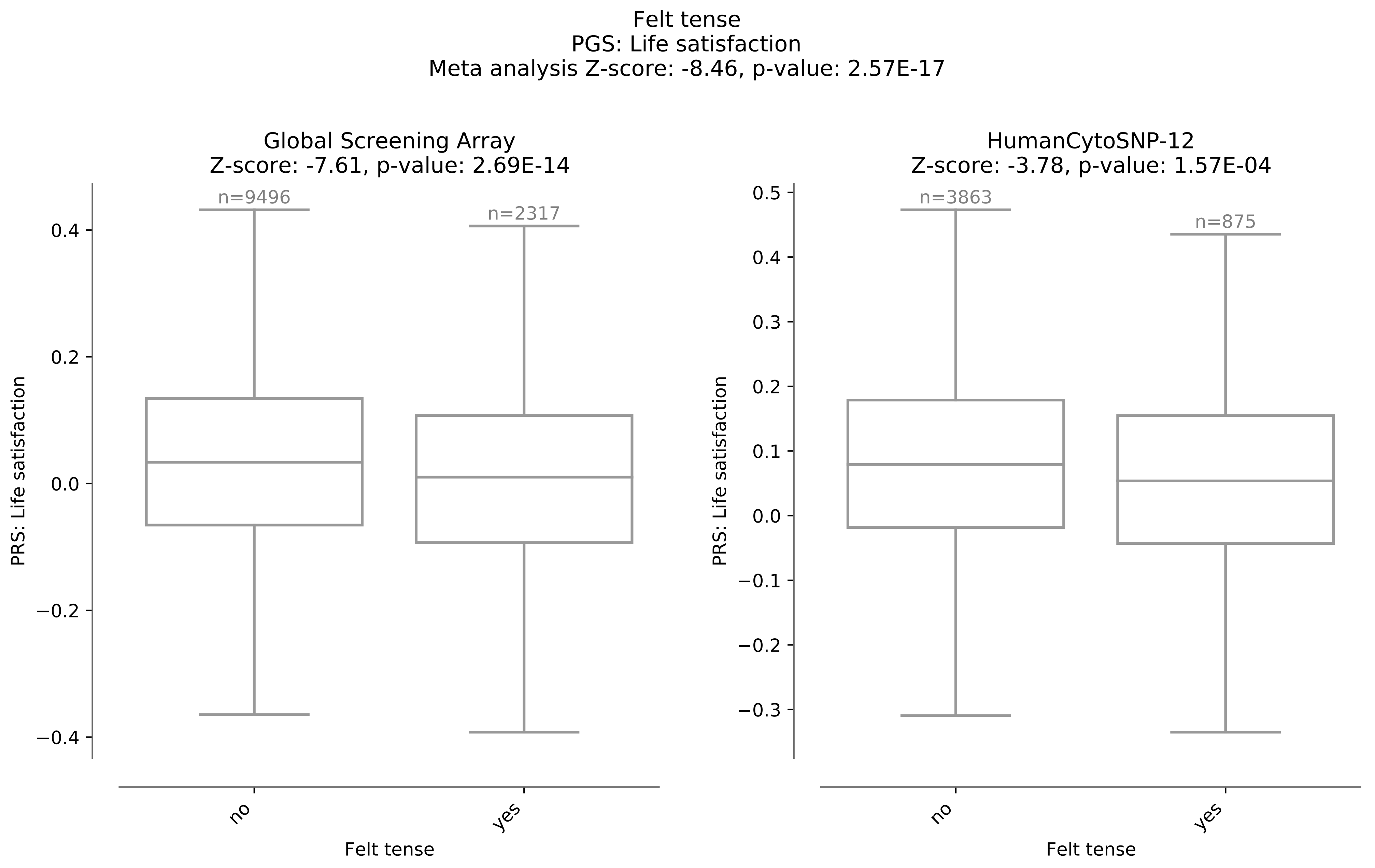
Felt tense
PGS: Life satisfaction
Meta analysis Z-score: -8.46, p-value: 2.57E-17
Global Screening Array
Z-score: -7.61, p-value: 2.69E-14
n=9496
n=2317
PRS: Life satisfaction
0.4
0.2
0.0
−0.2
−0.4
no
yes
Felt tense
HumanCytoSNP-12
Z-score: -3.78, p-value: 1.57E-04
n=3863
n=875
PRS: Life satisfaction
0.5
0.4
0.3
0.2
0.1
0.0
−0.1
−0.2
−0.3
no
yes
Felt tense

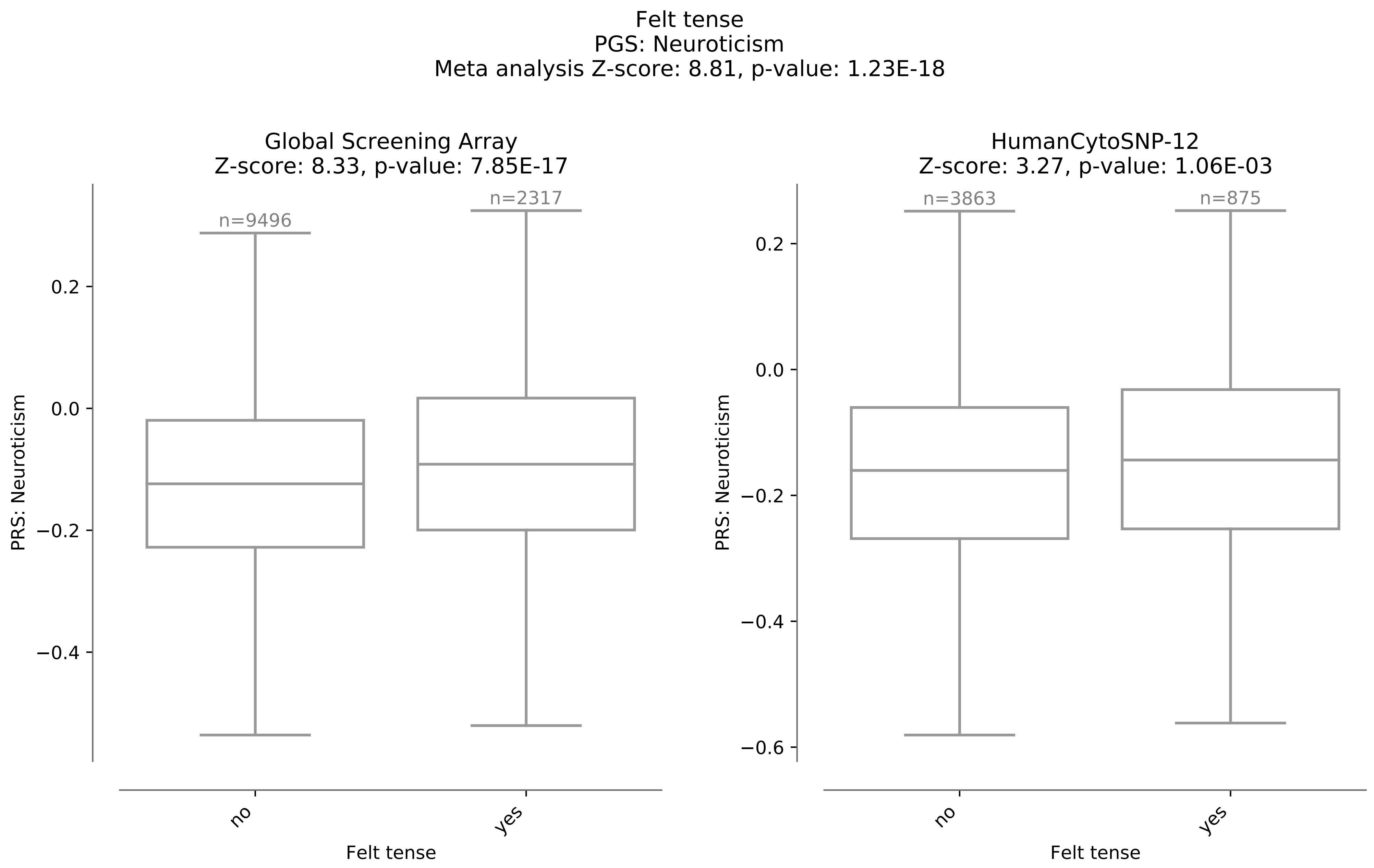
Felt tense
PGS: Neuroticism
Meta analysis Z-score: 8.81, p-value: 1.23E-18
Global Screening Array
Z-score: 8.33, p-value: 7.85E-17
n=9496
n=2317
PRS: Neuroticism
0.2
0.0
−0.2
−0.4
no
yes
Felt tense
HumanCytoSNP-12
Z-score: 3.27, p-value: 1.06E-03
n=3863
n=875
PRS: Neuroticism
0.2
0.0
−0.2
−0.4
−0.6
no
yes
Felt tense

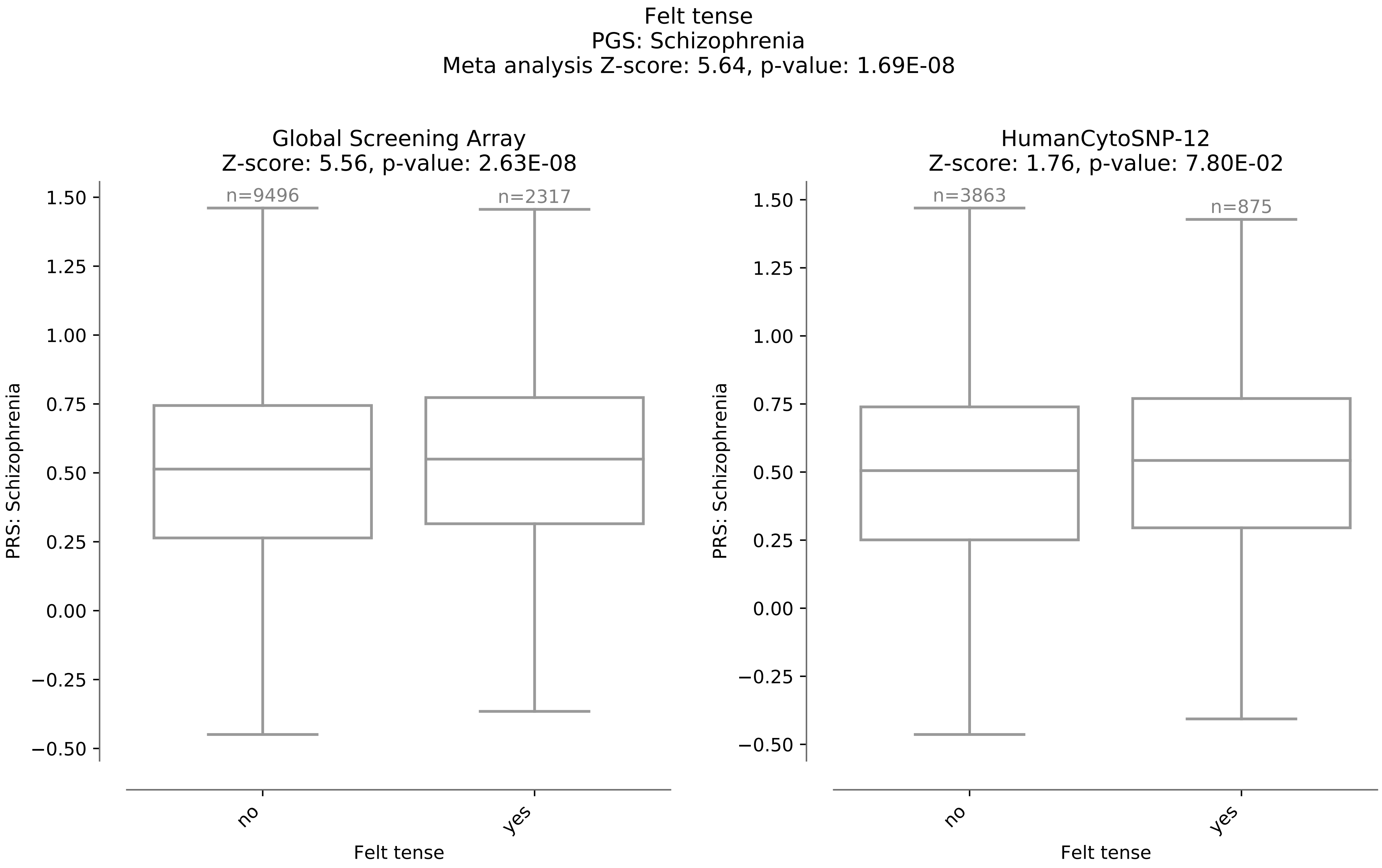
Felt tense
PGS: Schizophrenia
Meta analysis Z-score: 5.64, p-value: 1.69E-08
Global Screening Array
Z-score: 5.56, p-value: 2.63E-08
n=9496
n=2317
PRS: Schizophrenia
1.50
1.25
1.00
0.75
0.50
0.25
0.00
−0.25
−0.50
no
yes
Felt tense
HumanCytoSNP-12
Z-score: 1.76, p-value: 7.80E-02
n=3863
n=875
PRS: Schizophrenia
no
yes
Felt tense

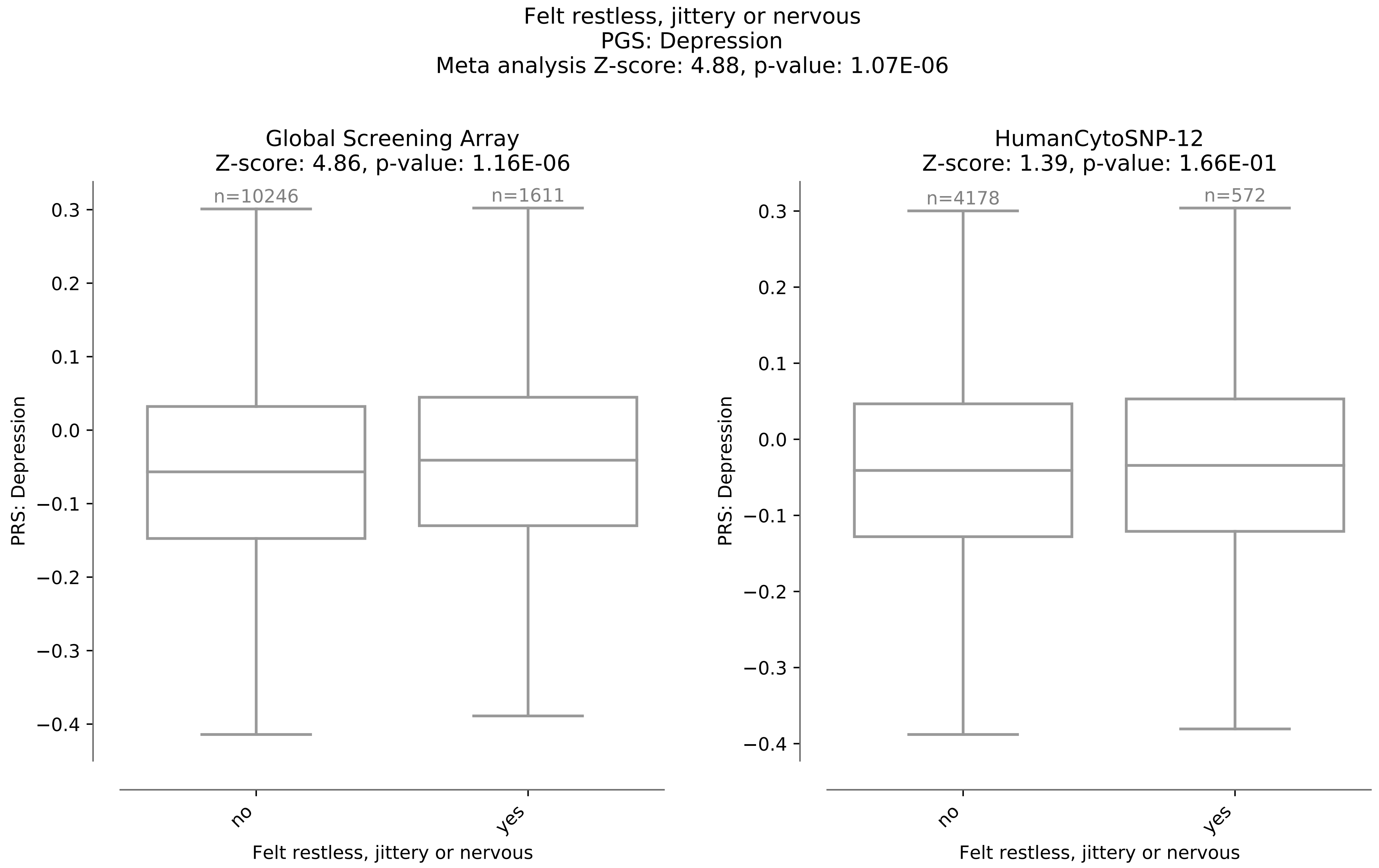
Felt restless, jittery or nervous
PGS: Depression
Meta analysis Z-score: 4.88, p-value: 1.07E-06
Global Screening Array
Z-score: 4.86, p-value: 1.16E-06
n=10246
n=1611
0.3
0.2
0.1
0.0
−0.1
−0.2
−0.3
−0.4
PRS: Depression
no
yes
Felt restless, jittery or nervous
HumanCytoSNP-12
Z-score: 1.39, p-value: 1.66E-01
n=4178
n=572
0.3
0.2
0.1
0.0
−0.1
−0.2
−0.3
−0.4
PRS: Depression
no
yes
Felt restless, jittery or nervous

Felt restless, jittery or nervous
PGS: Life satisfaction
Meta analysis Z-score: -7.57, p-value: 3.82E-14

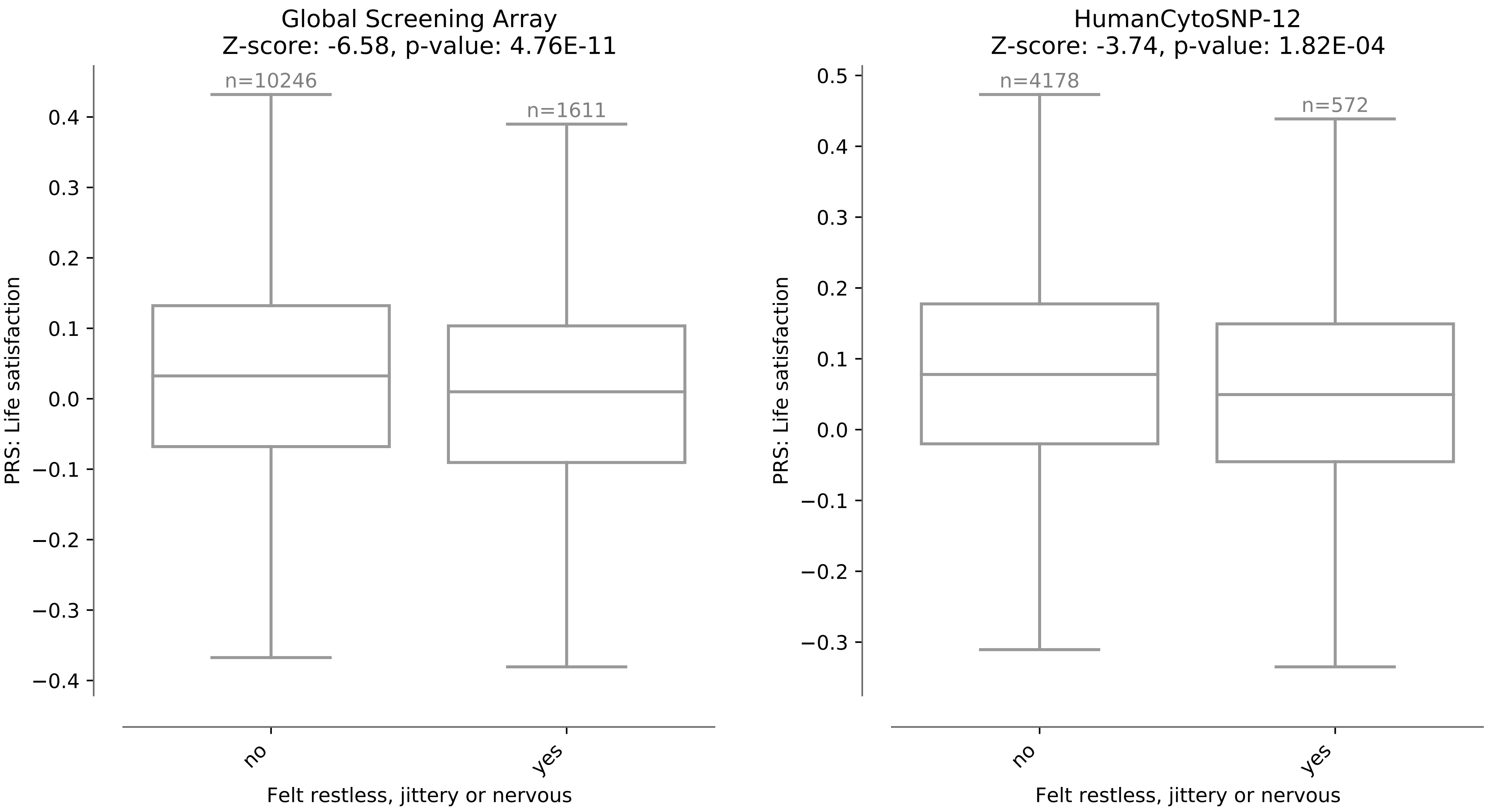

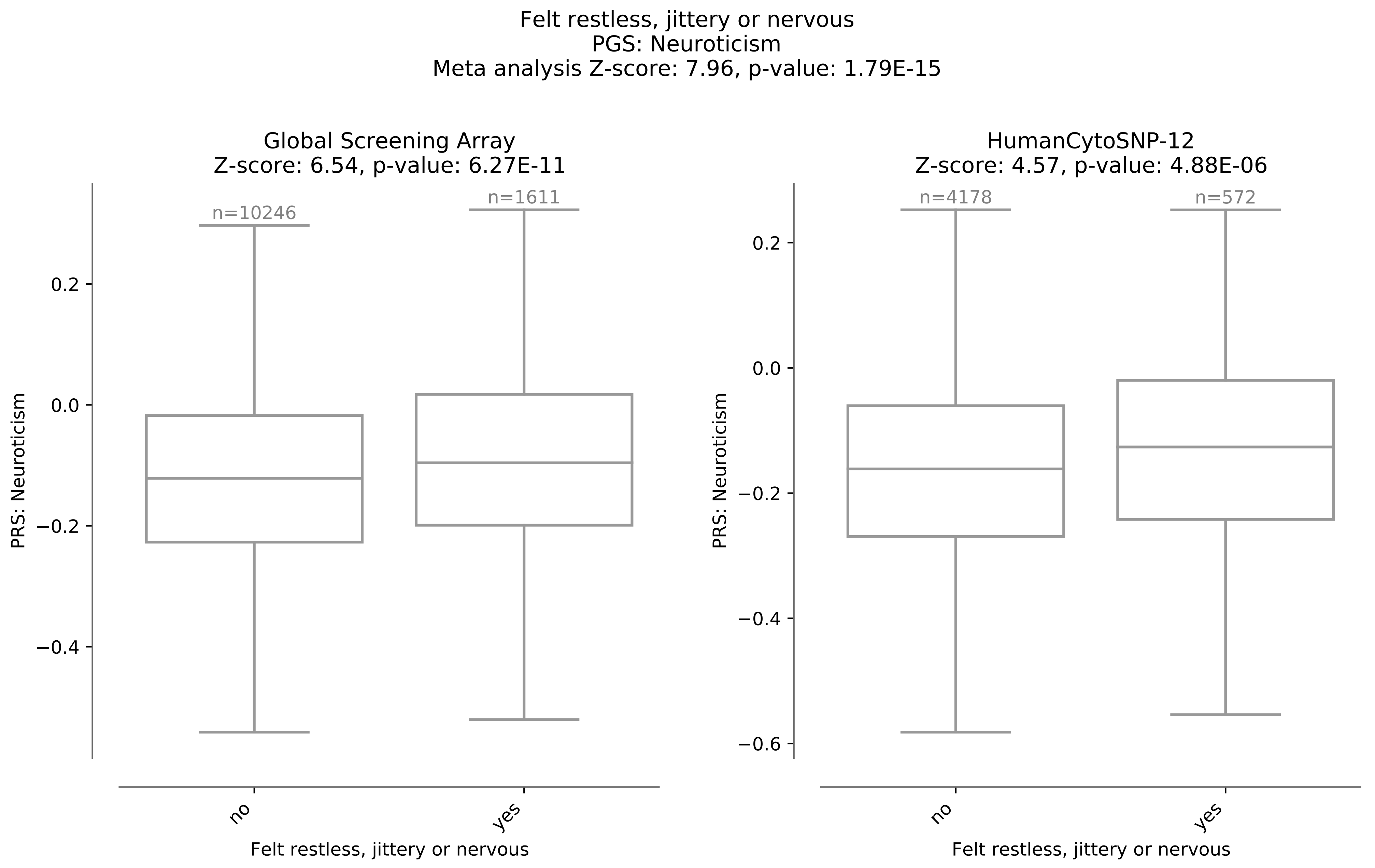
Felt restless, jittery or nervous
PGS: Neuroticism
Meta analysis Z-score: 7.96, p-value: 1.79E-15
Global Screening Array
Z-score: 6.54, p-value: 6.27E-11
n=10246
n=1611
0.2
0.0
−0.2
−0.4
PRS: Neuroticism
no
yes
Felt restless, jittery or nervous
HumanCytoSNP-12
Z-score: 4.57, p-value: 4.88E-06
n=4178
n=572
0.2
0.0
−0.2
−0.4
−0.6
PRS: Neuroticism
no
yes
Felt restless, jittery or nervous

Celebrating christmas in church before the pandemic
PGS: Anxiety/tension
Meta analysis Z-score: 4.80, p-value: 1.57E-06

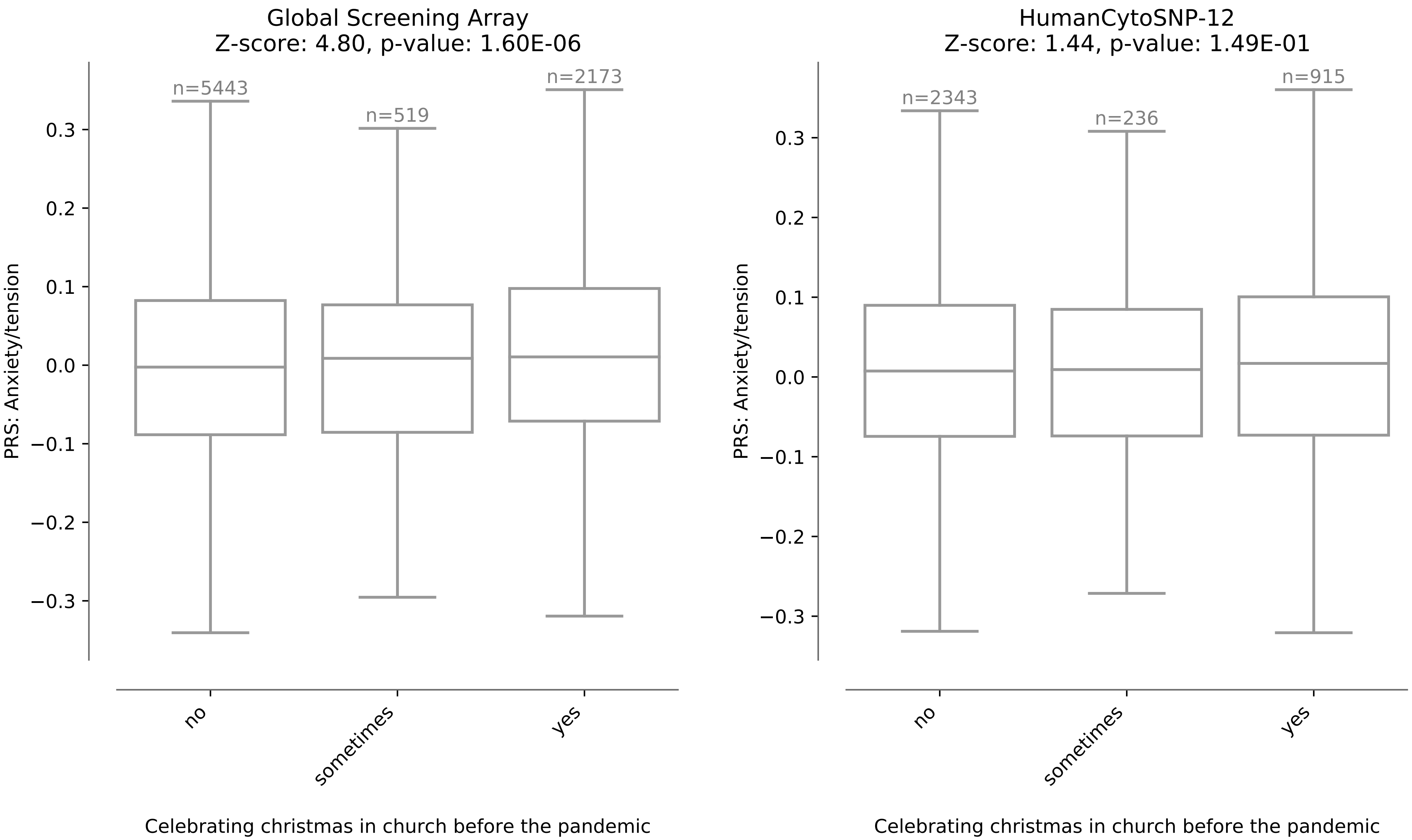

Celebrating christmas in church before the pandemic
PGS: Educational attainment
Meta analysis Z-score: 11.79, p-value: 4.54E-32

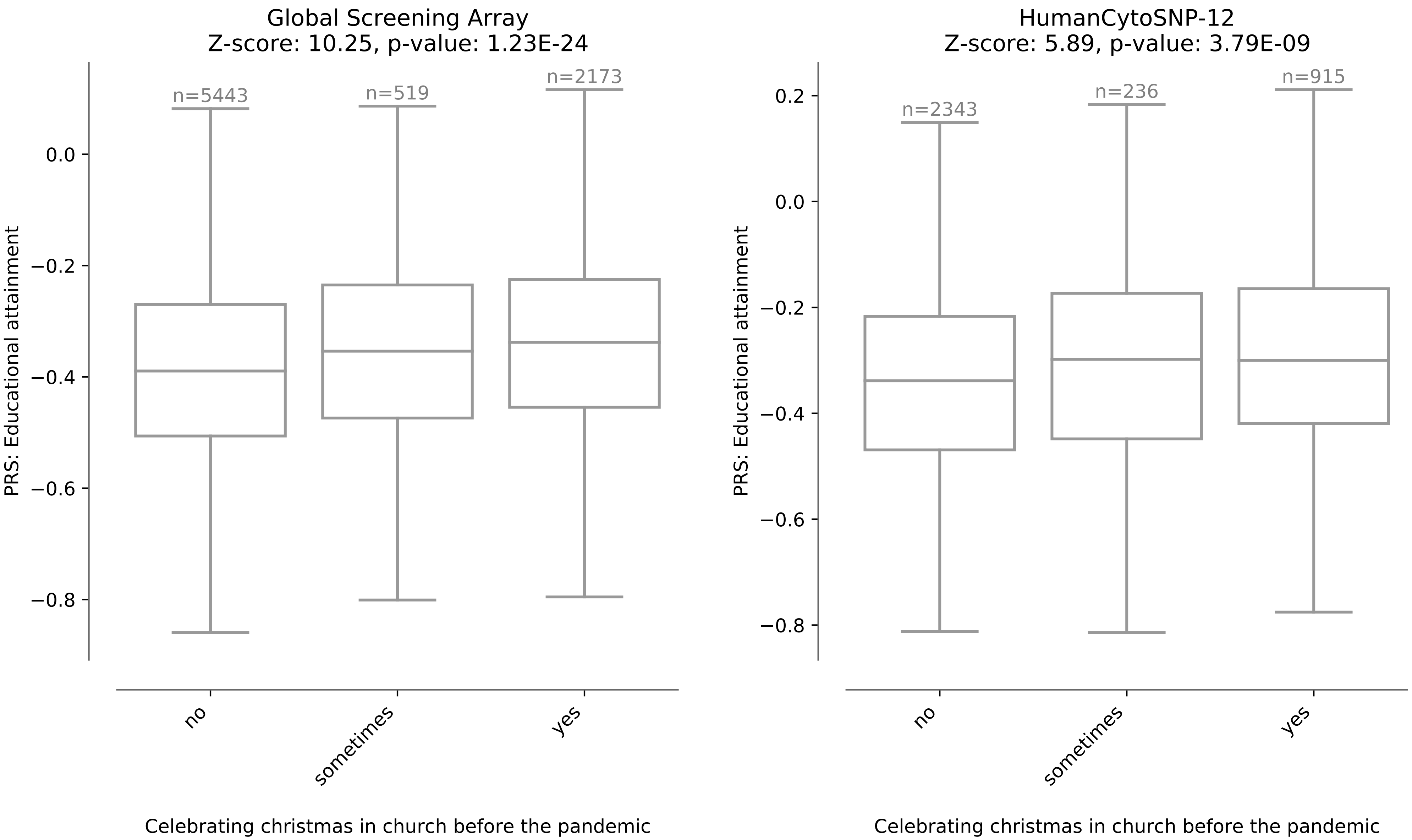

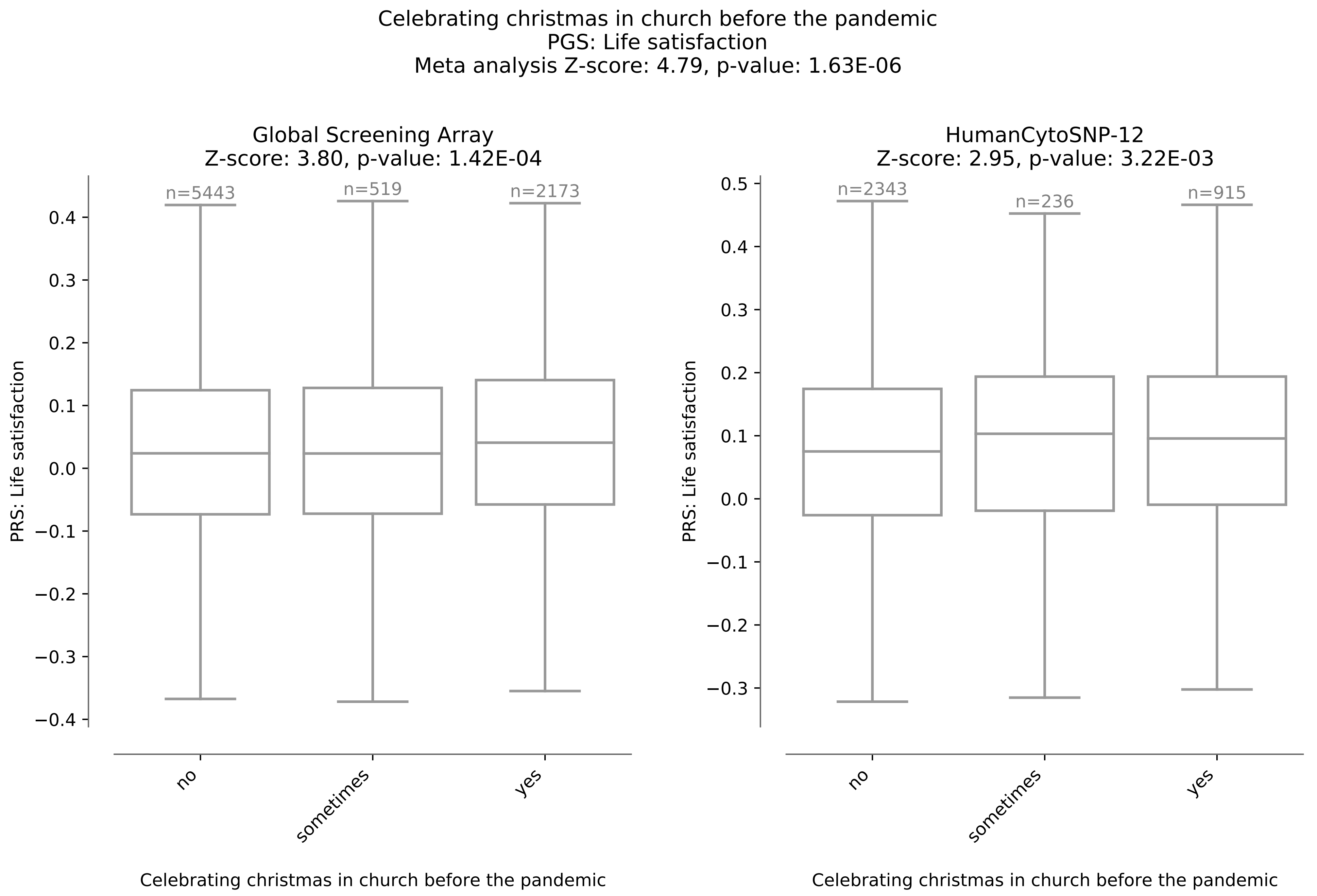
Celebrating christmas in church before the pandemic
PGS: Life satisfaction
Meta analysis Z-score: 4.79, p-value: 1.63E-06
Global Screening Array
Z-score: 3.80, p-value: 1.42E-04
n=5443
n=519
n=2173
PRS: Life satisfaction
0.4
0.3
0.2
0.1
0.0
−0.1
−0.2
−0.3
−0.4
no
sometimes
yes
Celebrating christmas in church before the pandemic
HumanCytoSNP-12
Z-score: 2.95, p-value: 3.22E-03
n=2343
n=236
n=915
PRS: Life satisfaction
0.5
0.4
0.3
0.2
0.1
0.0
−0.1
−0.2
−0.3
no
sometimes
yes
Celebrating christmas in church before the pandemic

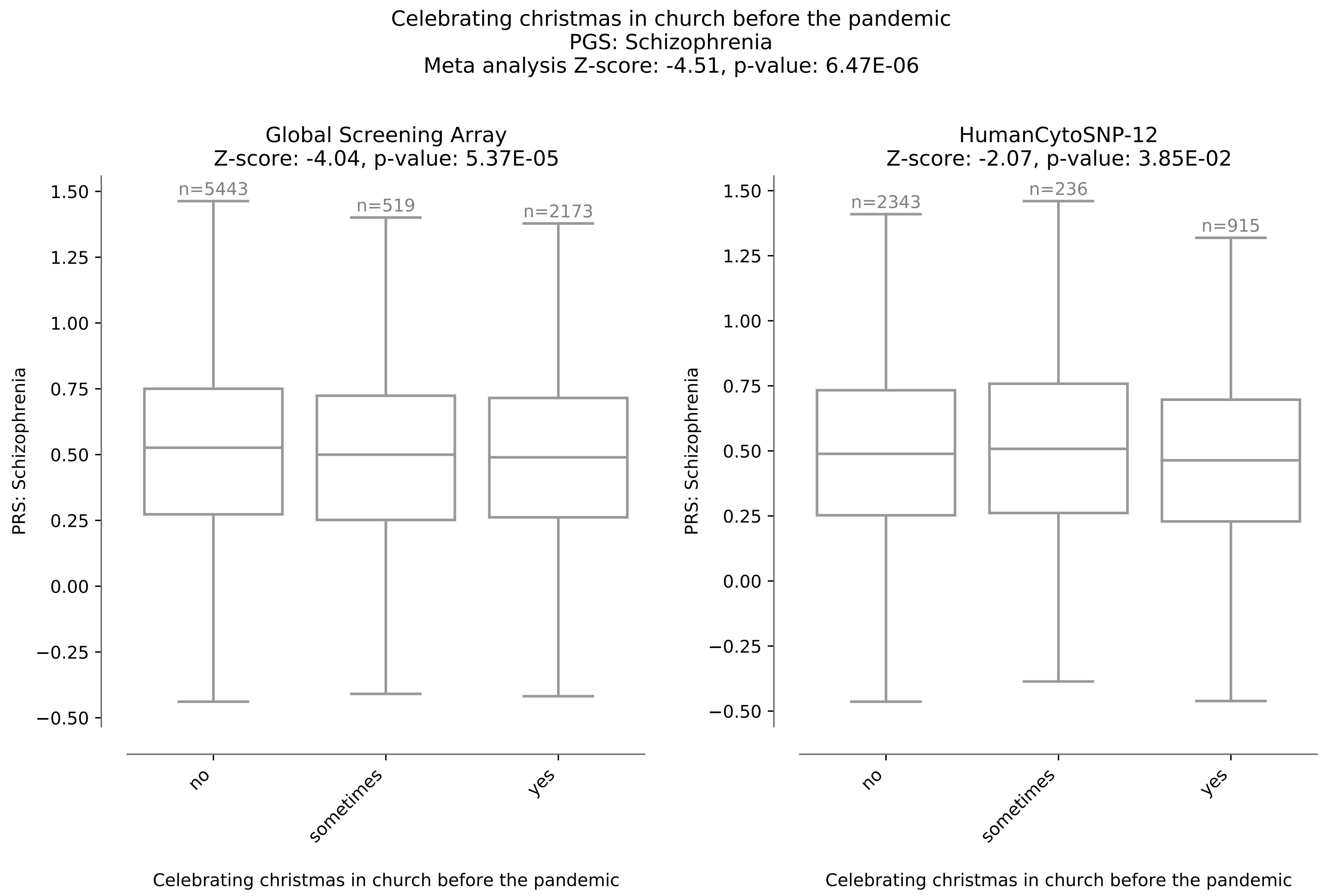

Celebrating christmas in church before the pandemic
PGS: Schizophrenia
Meta analysis Z-score: -4.51, p-value: 6.47E-06
Global Screening Array
Z-score: -4.04, p-value: 5.37E-05
HumanCytoSNP-12
Z-score: -2.07, p-value: 3.85E-02
PRS: Schizophrenia
n=5443
n=519
n=2173
n=2343
n=236
n=915
1.50
1.25
1.00
0.75
0.50
0.25
0.00
−0.25
−0.50
no
sometimes
yes
Celebrating christmas in church before the pandemic

Celebrating christmas in church before the pandemic
PGS: Worry/vulnerability
Meta analysis Z-score: 5.34, p-value: 9.13E-08

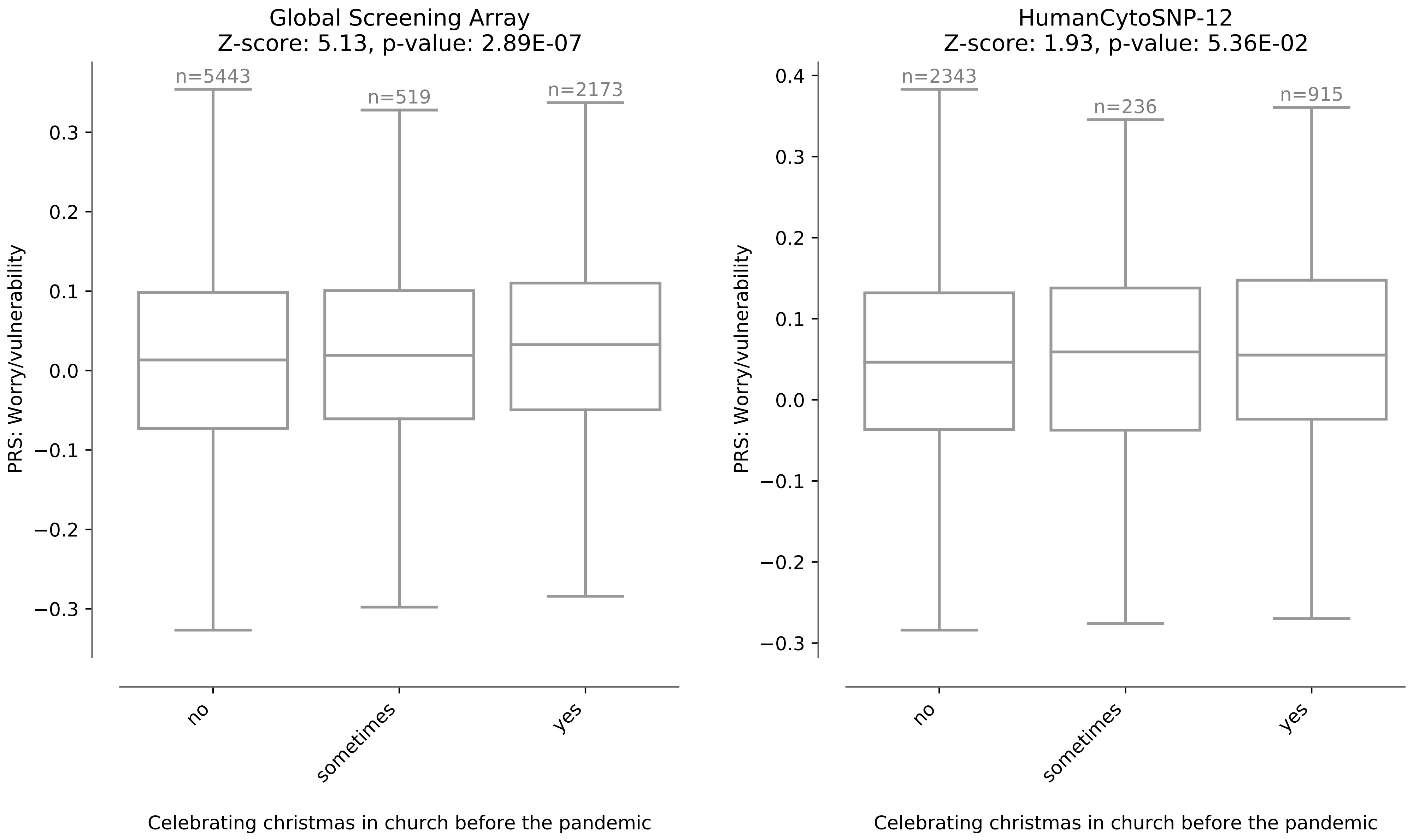

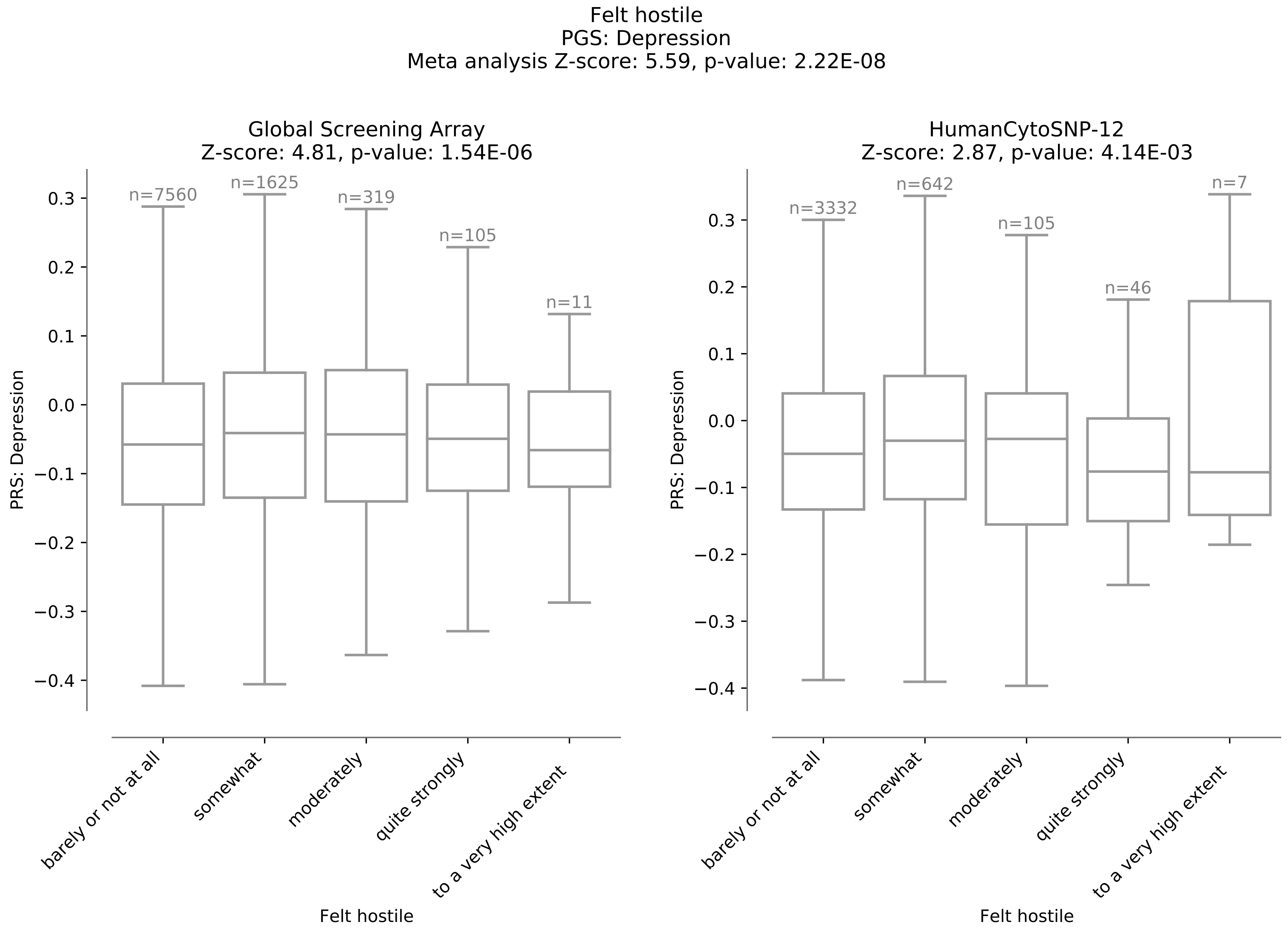
Felt hostile
PGS: Depression
Meta analysis Z-score: 5.59, p-value: 2.22E-08
Global Screening Array
Z-score: 4.81, p-value: 1.54E-06
HumanCytoSNP-12
Z-score: 2.87, p-value: 4.14E-03
PRS: Depression
0.3
0.2
0.1
0.0
−0.1
−0.2
−0.3
−0.4
n=7560
n=1625
n=319
n=105
n=11
n=3332
n=642
n=105
n=46
n=7
barely or not at all
somewhat
moderately
quite strongly
to a very high extent
Felt hostile

Felt hostile
PGS: Life satisfaction
Meta analysis Z-score: -7.34, p-value: 2.15E-13

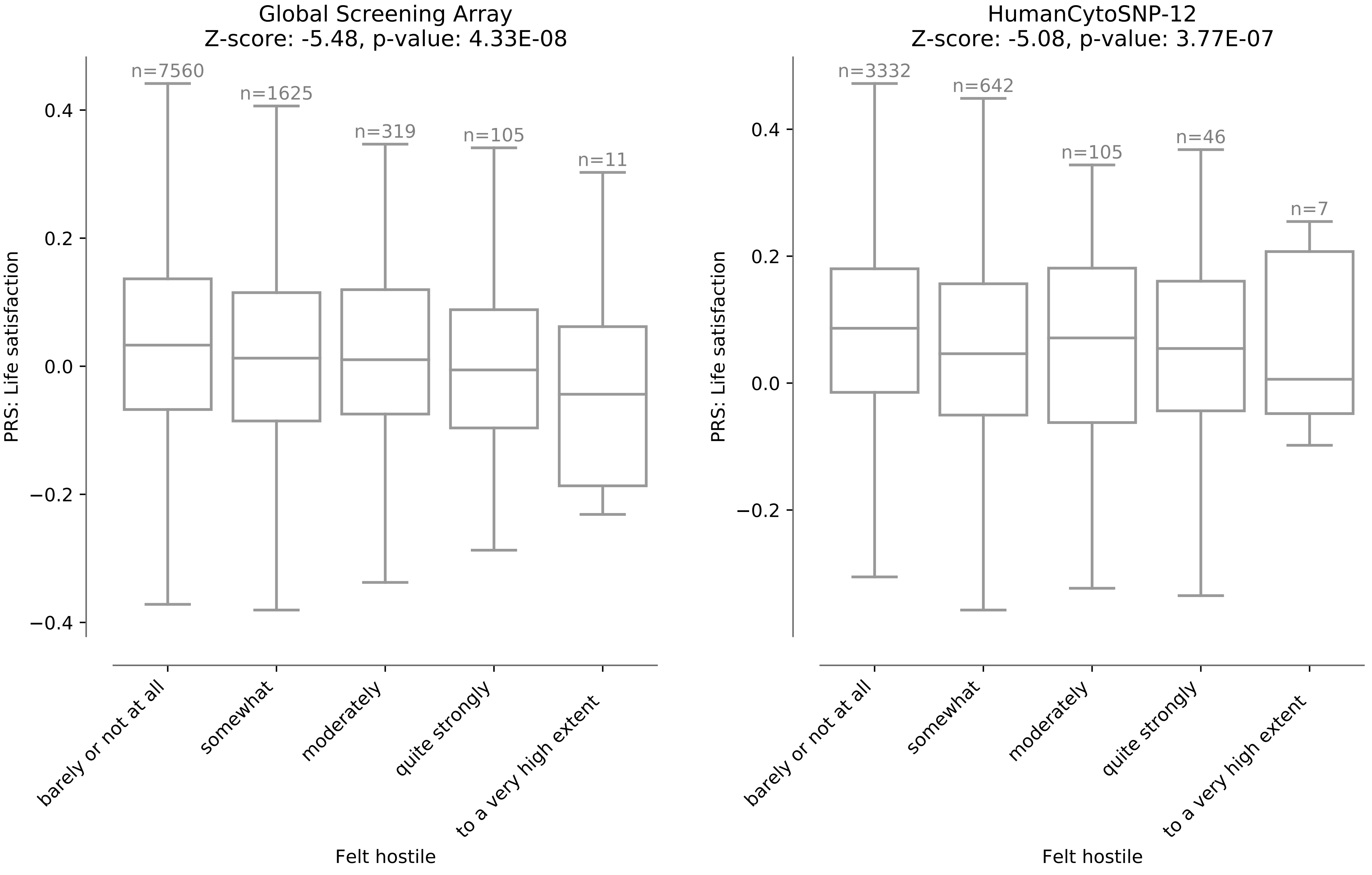

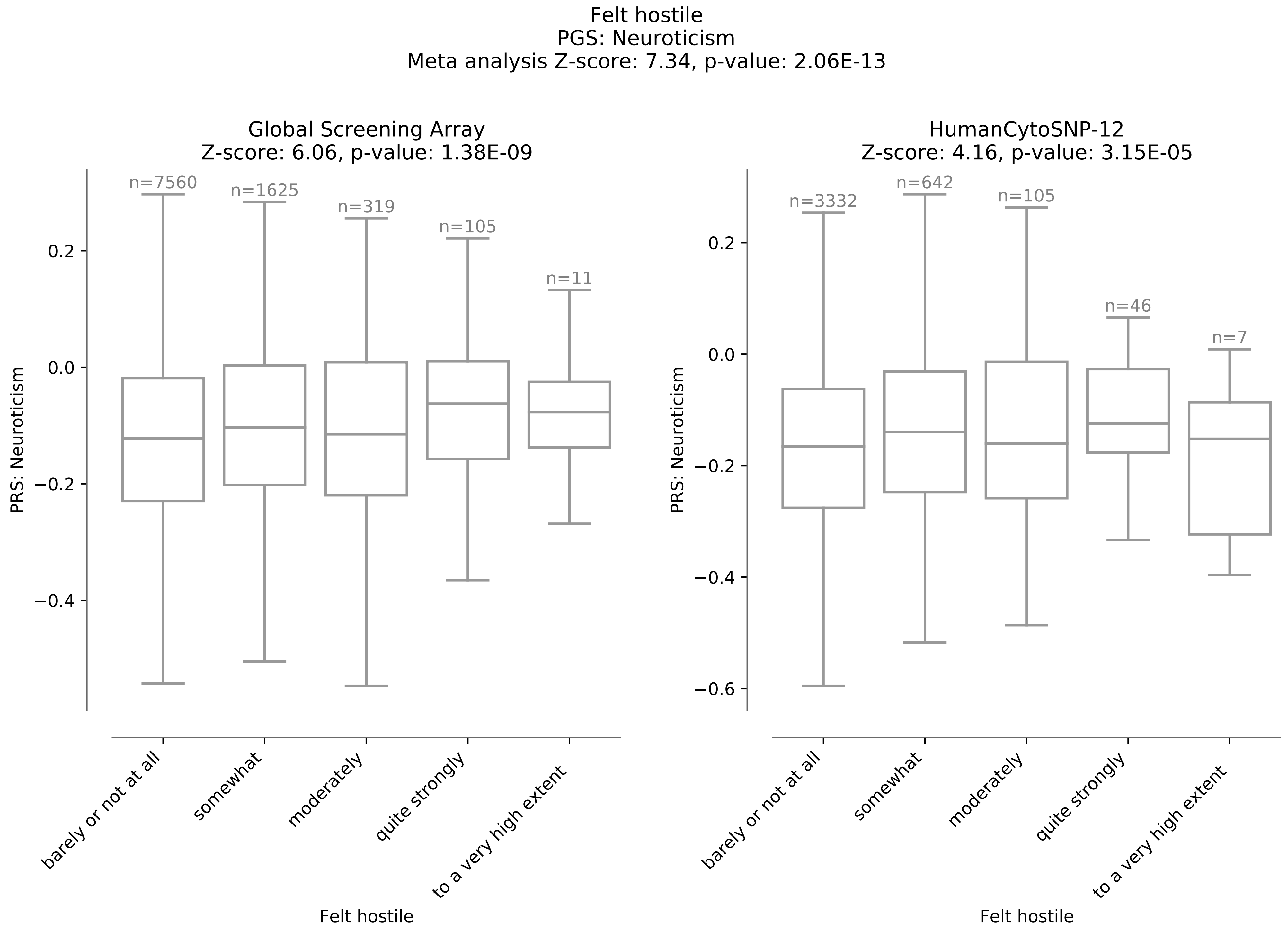
Felt hostile
PGS: Neuroticism
Meta analysis Z-score: 7.34, p-value: 2.06E-13
Global Screening Array
Z-score: 6.06, p-value: 1.38E-09
n=7560
n=1625
n=319
n=105
n=11
0.2
0.0
−0.2
−0.4
PRS: Neuroticism
barely or not at all
somewhat
moderately
quite strongly
to a very high extent
Felt hostile
HumanCytoSNP-12
Z-score: 4.16, p-value: 3.15E-05
n=3332
n=642
n=105
n=46
n=7
0.2
0.0
−0.2
−0.4
−0.6
PRS: Neuroticism
barely or not at all
somewhat
moderately
quite strongly
to a very high extent
Felt hostile

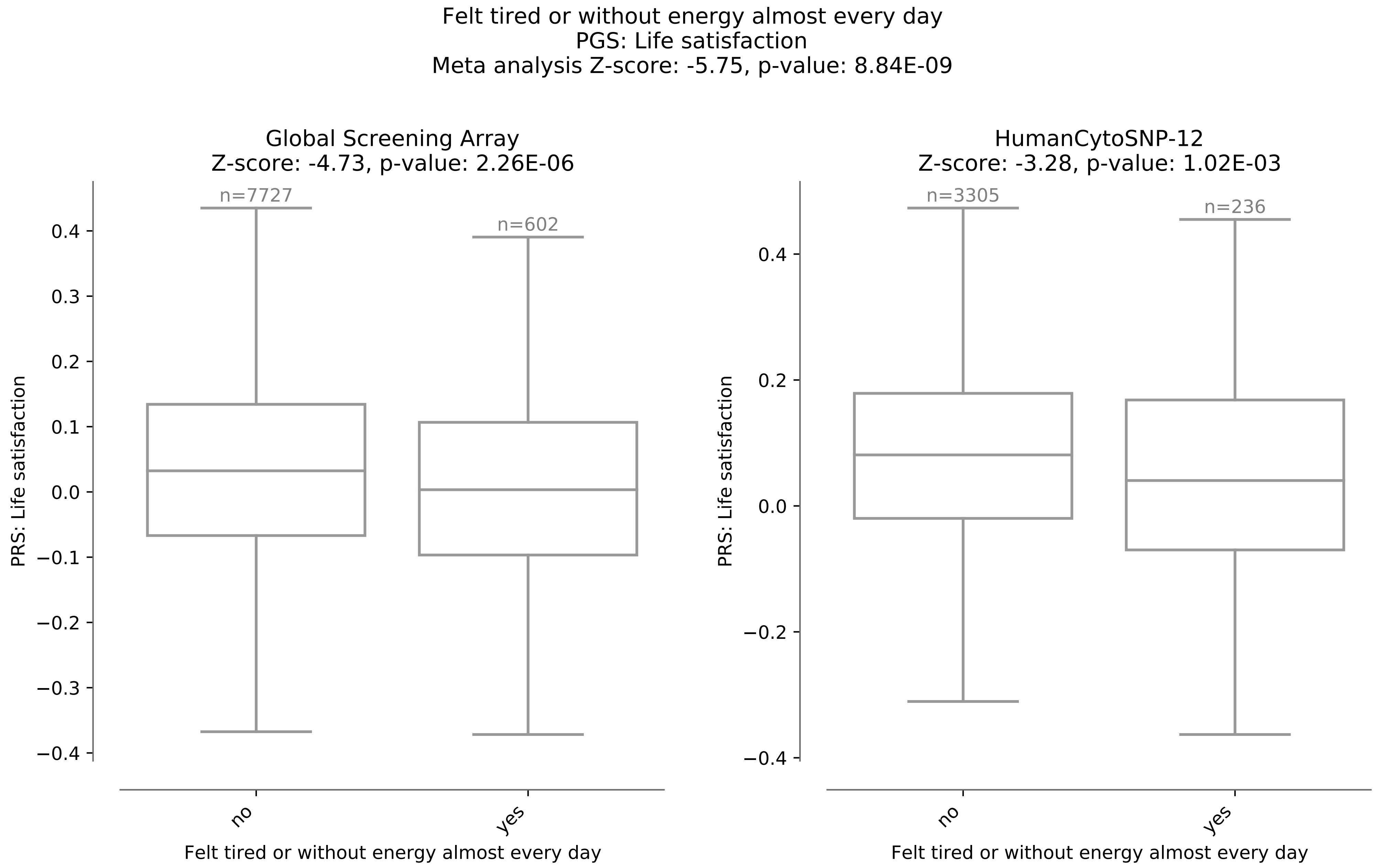
Felt tired or without energy almost every day
PGS: Life satisfaction
Meta analysis Z-score: -5.75, p-value: 8.84E-09
Global Screening Array
Z-score: -4.73, p-value: 2.26E-06
n=7727
n=602
PRS: Life satisfaction
0.4
0.3
0.2
0.1
0.0
−0.1
−0.2
−0.3
−0.4
no
yes
Felt tired or without energy almost every day
HumanCytoSNP-12
Z-score: -3.28, p-value: 1.02E-03
n=3305
n=236
PRS: Life satisfaction
0.4
0.2
0.0
−0.2
−0.4
no
yes
Felt tired or without energy almost every day

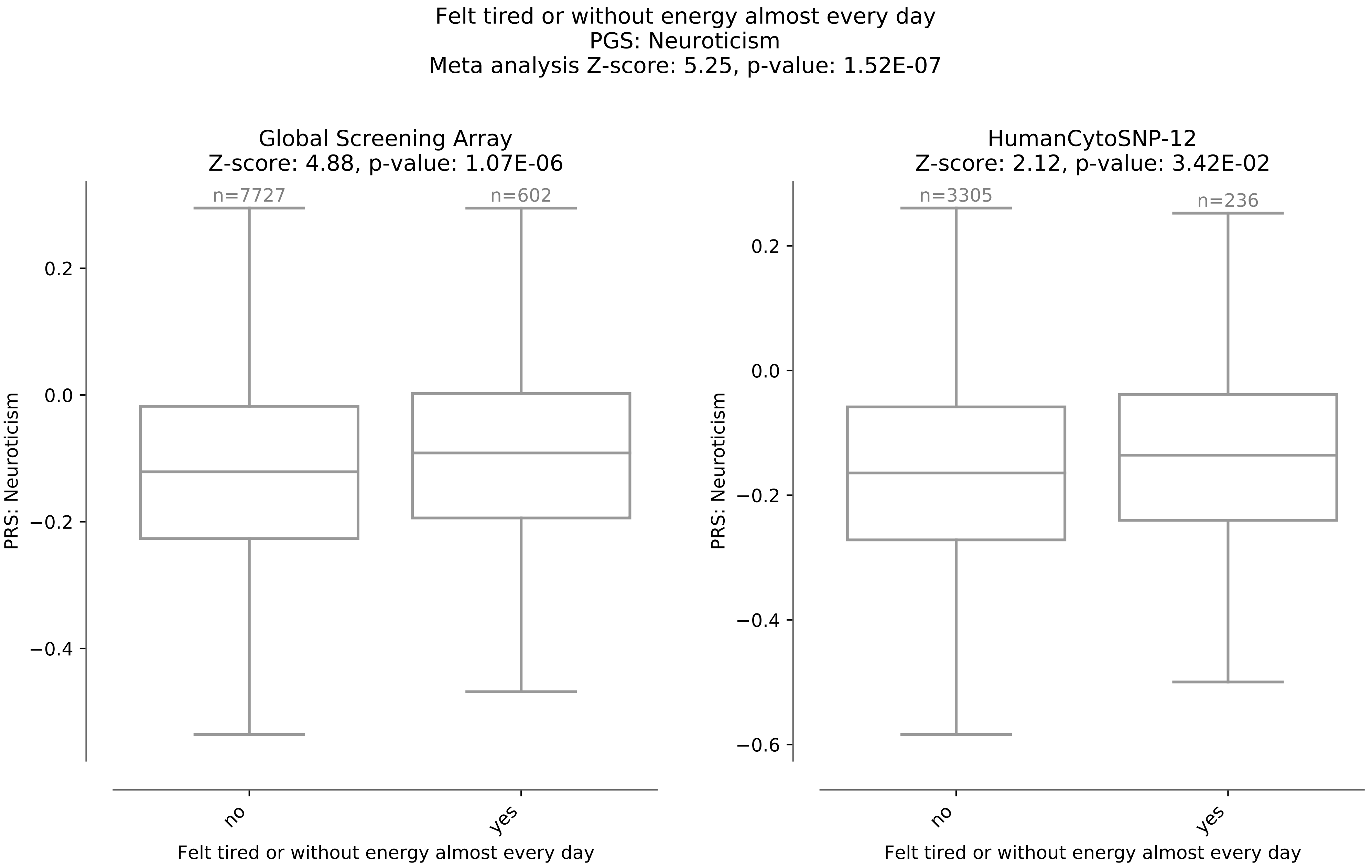

Felt tired or without energy almost every day
PGS: Neuroticism
Meta analysis Z-score: 5.25, p-value: 1.52E-07
Global Screening Array
Z-score: 4.88, p-value: 1.07E-06
n=7727
n=602
0.2
0.0
−0.2
−0.4
PRS: Neuroticism
no
yes
Felt tired or without energy almost every day
HumanCytoSNP-12
Z-score: 2.12, p-value: 3.42E-02
n=3305
n=236
0.2
0.0
−0.2
−0.4
−0.6
PRS: Neuroticism
no
yes
Felt tired or without energy almost every day

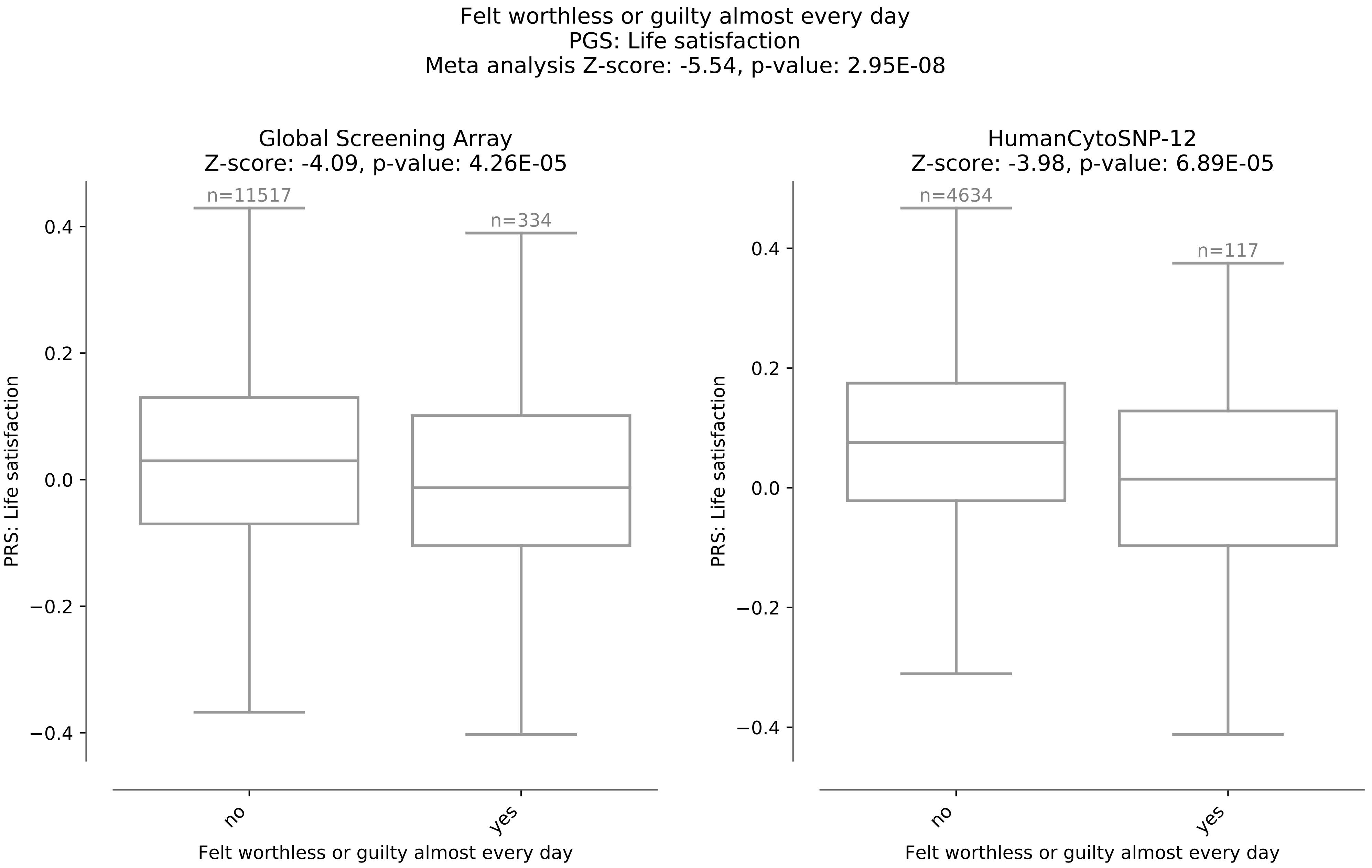

Felt worthless or guilty almost every day
PGS: Life satisfaction
Meta analysis Z-score: -5.54, p-value: 2.95E-08
Global Screening Array
Z-score: -4.09, p-value: 4.26E-05
n=11517
n=334
0.4
0.2
0.0
−0.2
−0.4
PRS: Life satisfaction
no
yes
Felt worthless or guilty almost every day
HumanCytoSNP-12
Z-score: -3.98, p-value: 6.89E-05
n=4634
n=117
0.4
0.2
0.0
−0.2
−0.4
PRS: Life satisfaction
no
yes
Felt worthless or guilty almost every day

Pre-pandemic (moderate) intensity physical activity
PGS: Accelerometer-based physical activity
Meta analysis Z-score: 5.32, p-value: 1.01E-07

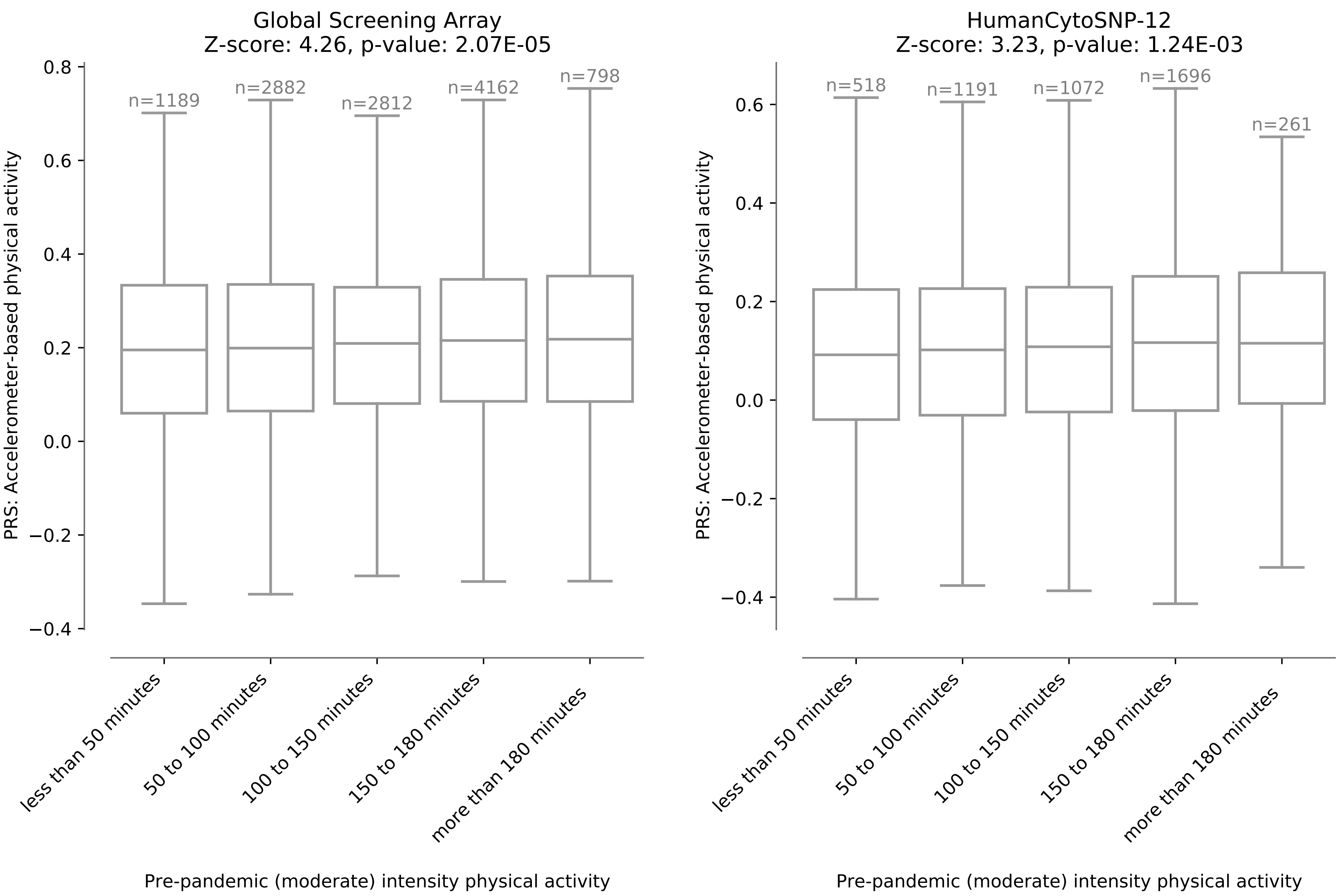

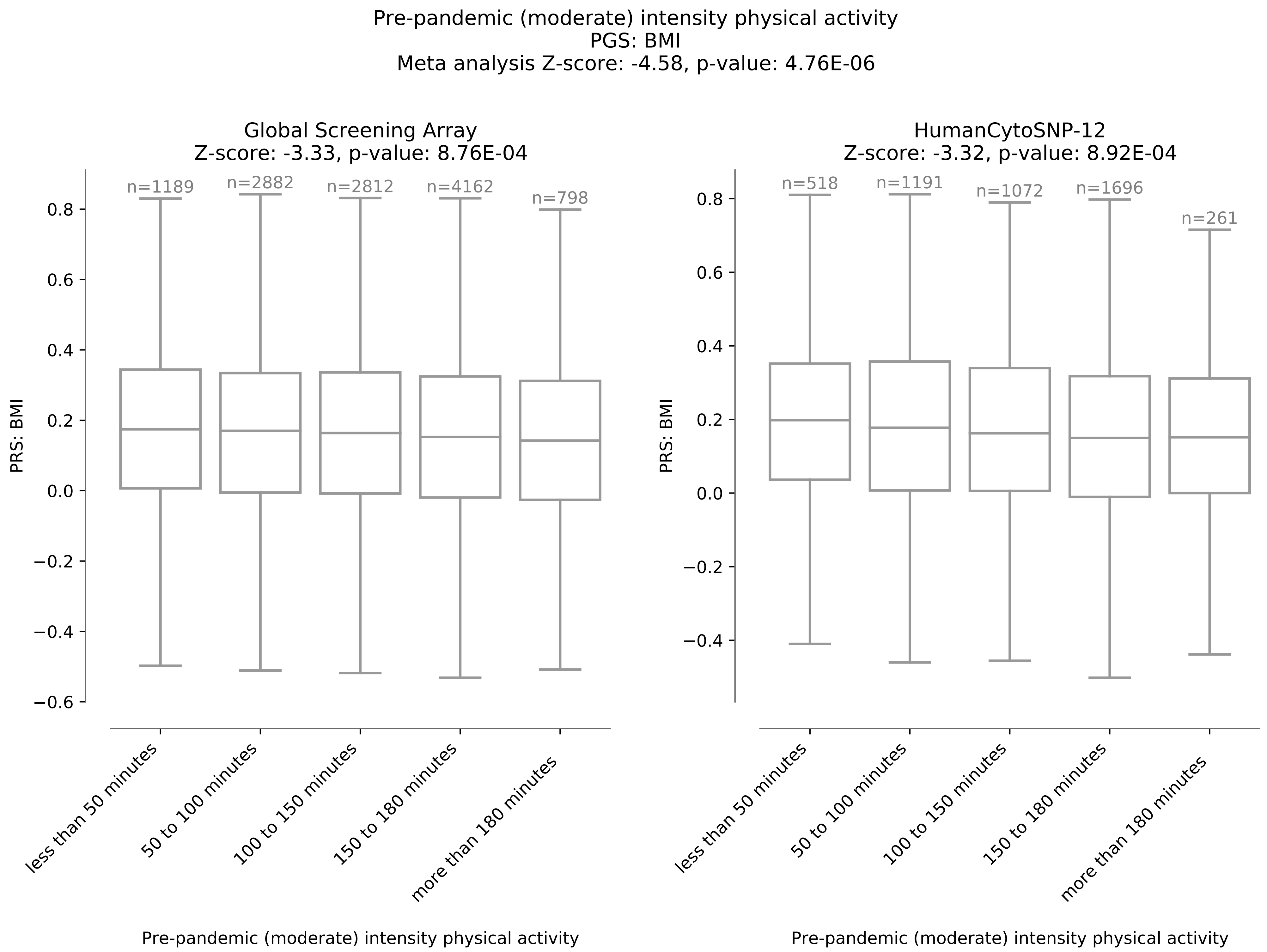
Pre-pandemic (moderate) intensity physical activity
PGS: BMI
Meta analysis Z-score: -4.58, p-value: 4.76E-06
Global Screening Array
Z-score: -3.33, p-value: 8.76E-04
n=1189
n=2882
n=2812
n=4162
n=798
PRS: BMI
0.8
0.6
0.4
0.2
0.0
−0.2
−0.4
−0.6
less than 50 minutes
50 to 100 minutes
100 to 150 minutes
150 to 180 minutes
more than 180 minutes
Pre-pandemic (moderate) intensity physical activity
HumanCytoSNP-12
Z-score: -3.32, p-value: 8.92E-04
n=518
n=1191
n=1072
n=1696
n=261
PRS: BMI
0.8
0.6
0.4
0.2
0.0
−0.2
−0.4
less than 50 minutes
50 to 100 minutes
100 to 150 minutes
150 to 180 minutes
more than 180 minutes
Pre-pandemic (moderate) intensity physical activity

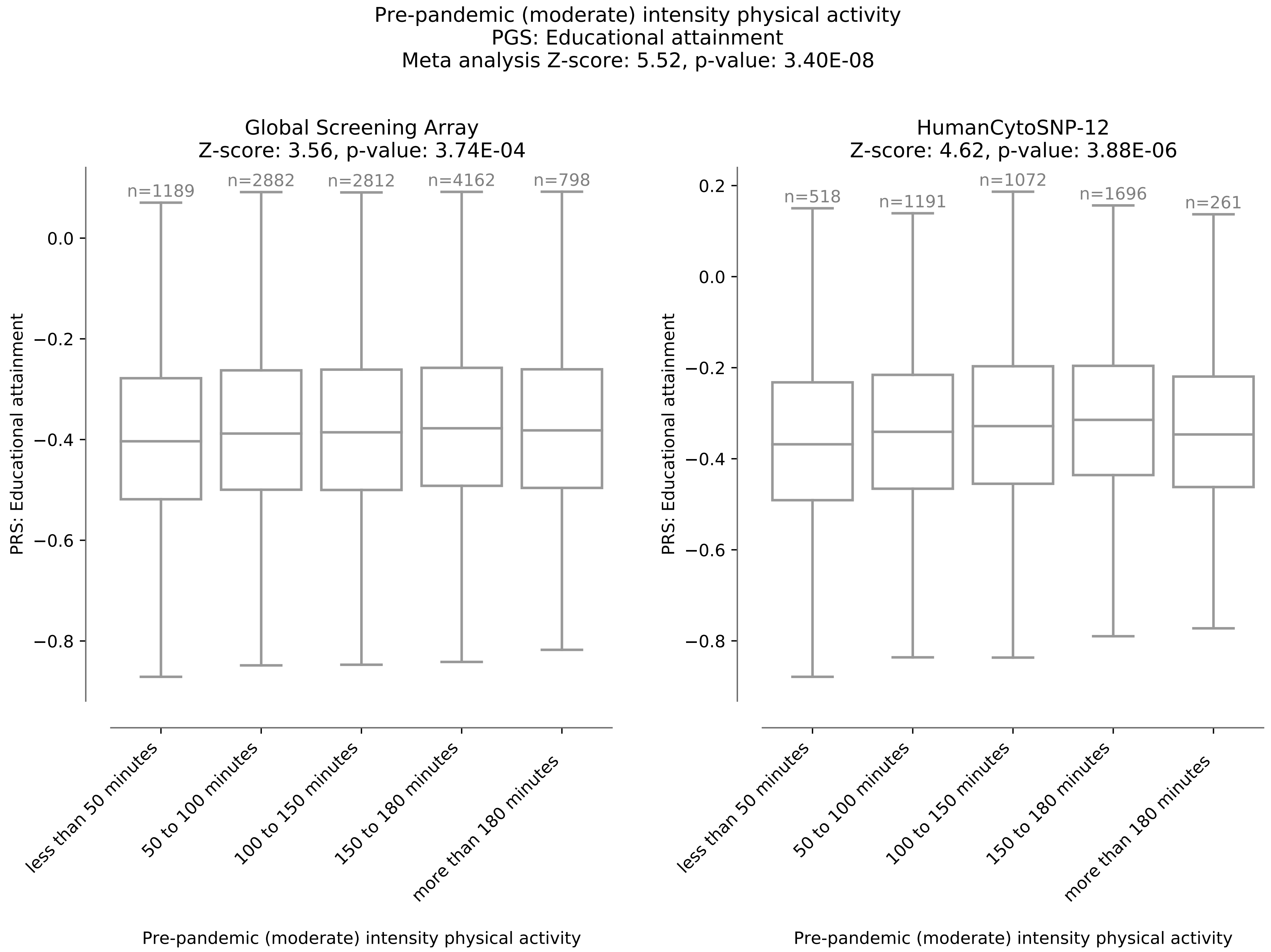
Pre-pandemic (moderate) intensity physical activity
PGS: Educational attainment
Meta analysis Z-score: 5.52, p-value: 3.40E-08
Global Screening Array
Z-score: 3.56, p-value: 3.74E-04
HumanCytoSNP-12
Z-score: 4.62, p-value: 3.88E-06
PRS: Educational attainment
n=1189
n=2882
n=2812
n=4162
n=798
n=518
n=1191
n=1072
n=1696
n=261
0.2
0.0
−0.2
−0.4
−0.6
−0.8
less than 50 minutes
50 to 100 minutes
100 to 150 minutes
150 to 180 minutes
more than 180 minutes
Pre-pandemic (moderate) intensity physical activity

Noticeable appetite change or unintended weight change
PGS: BMI
Meta analysis Z-score: 5.36, p-value: 8.38E-08

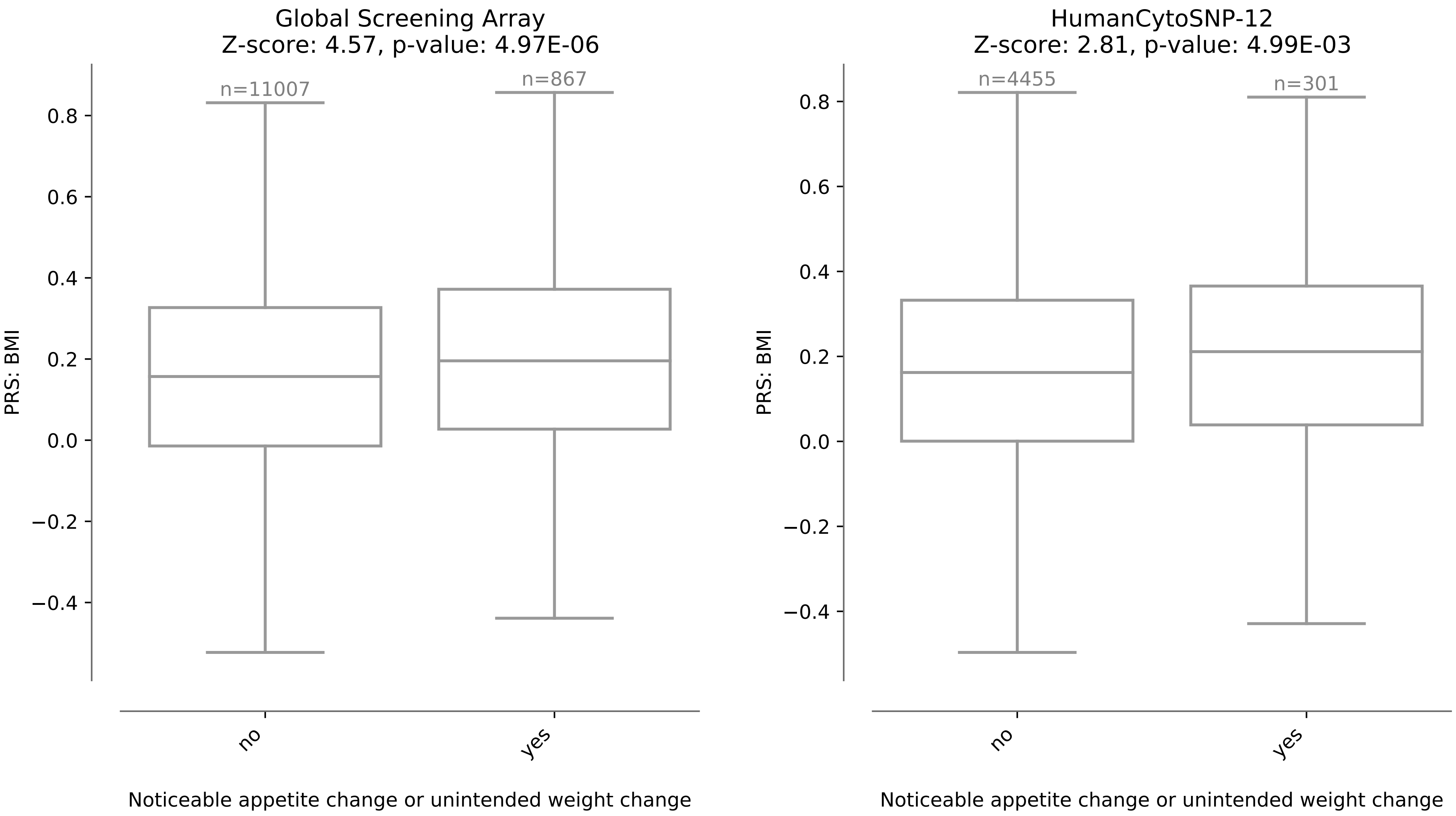

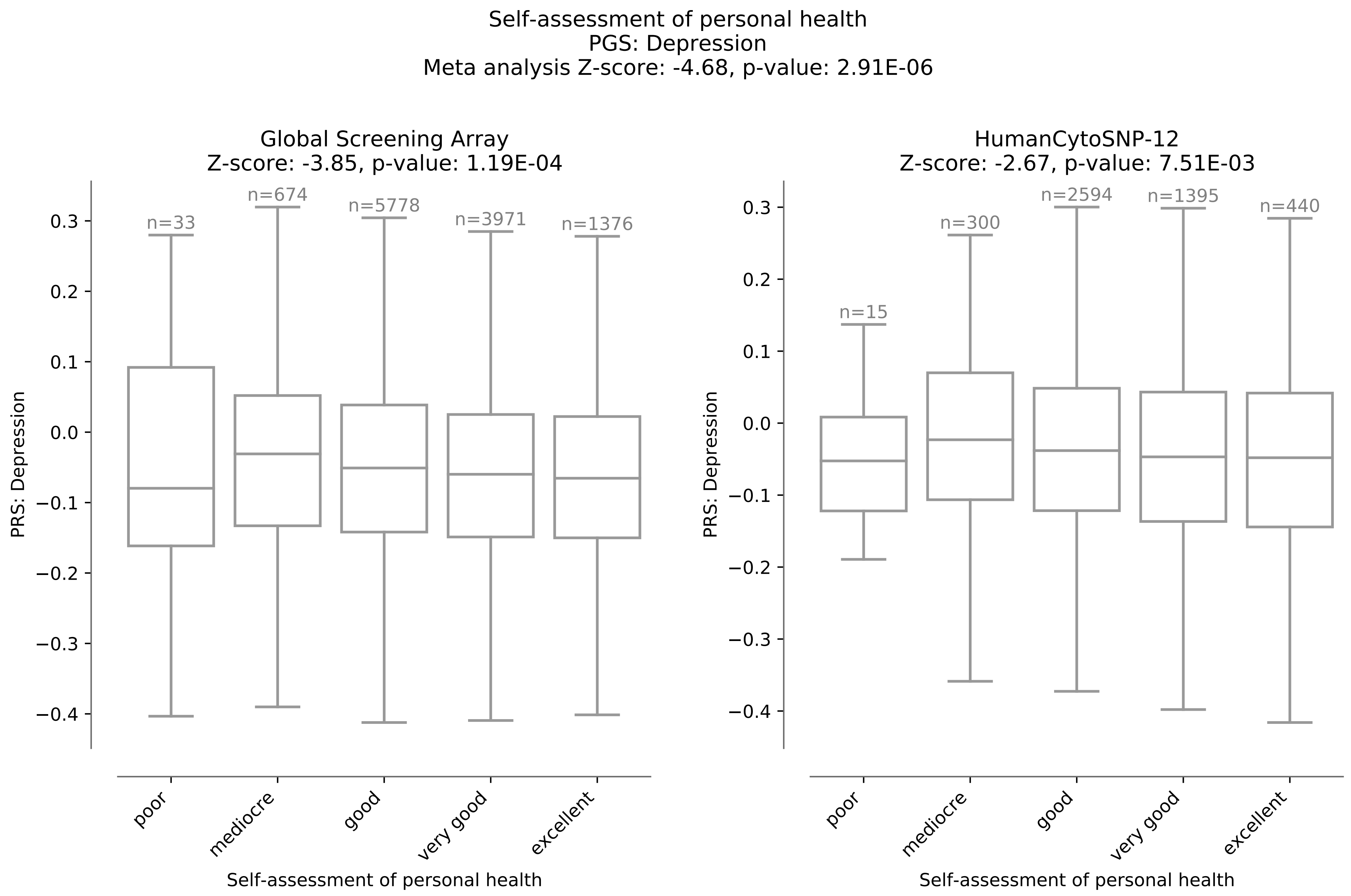
Self-assessment of personal health
PGS: Depression
Meta analysis Z-score: -4.68, p-value: 2.91E-06
Global Screening Array
Z-score: -3.85, p-value: 1.19E-04
n=33
n=674
n=5778
n=3971
n=1376
PRS: Depression
poor
mediocre
good
very good
excellent
Self-assessment of personal health
HumanCytoSNP-12
Z-score: -2.67, p-value: 7.51E-03
n=15
n=300
n=2594
n=1395
n=440
PRS: Depression
poor
mediocre
good
very good
excellent
Self-assessment of personal health

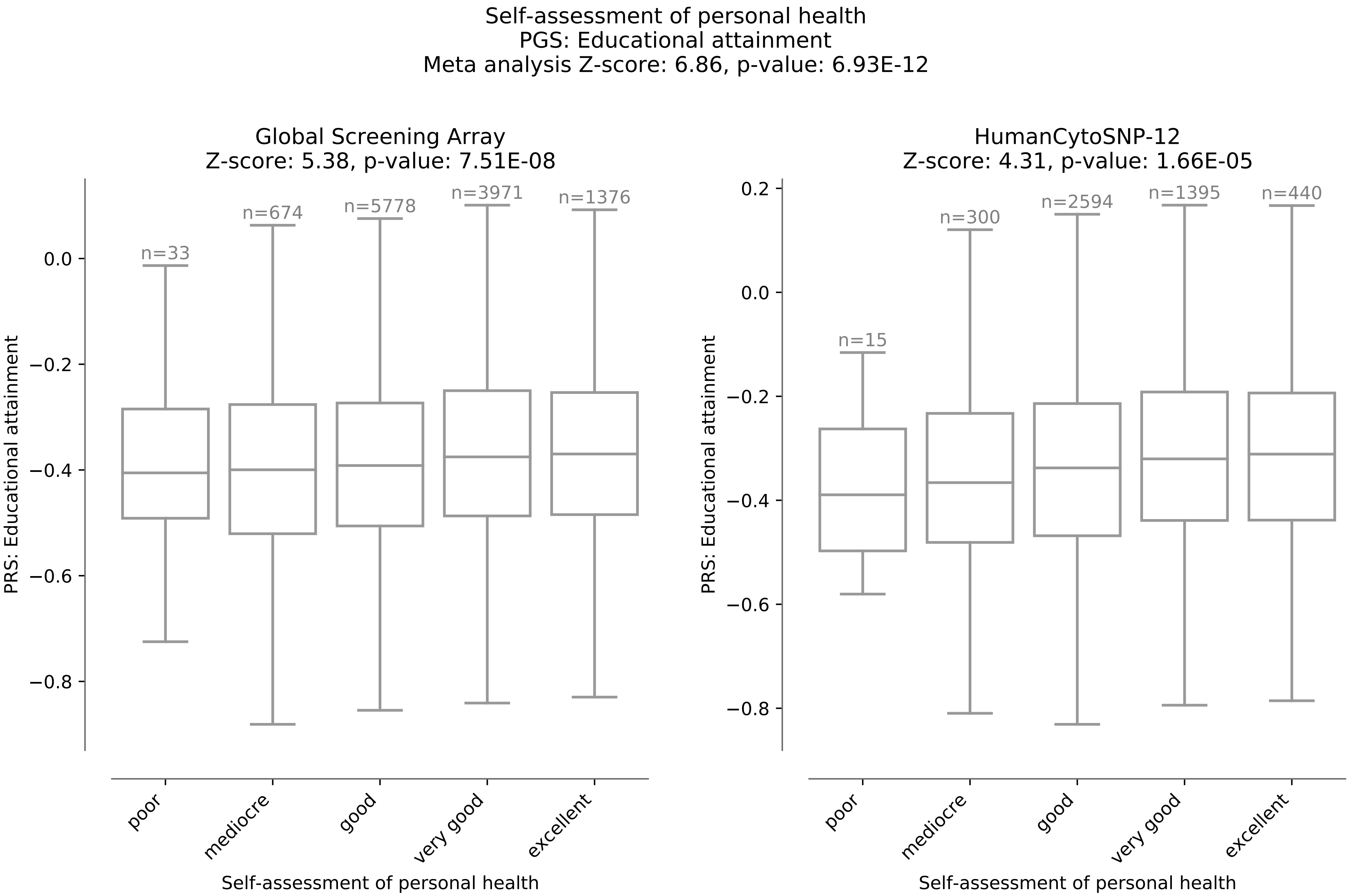
Self-assessment of personal health
PGS: Educational attainment
Meta analysis Z-score: 6.86, p-value: 6.93E-12
Global Screening Array
Z-score: 5.38, p-value: 7.51E-08
HumanCytoSNP-12
Z-score: 4.31, p-value: 1.66E-05
PRS: Educational attainment
n=33
n=674
n=5778
n=3971
n=1376
n=15
n=300
n=2594
n=1395
n=440
0.2
0.0
−0.2
−0.4
−0.6
−0.8
poor
mediocre
good
very good
excellent
Self-assessment of personal health

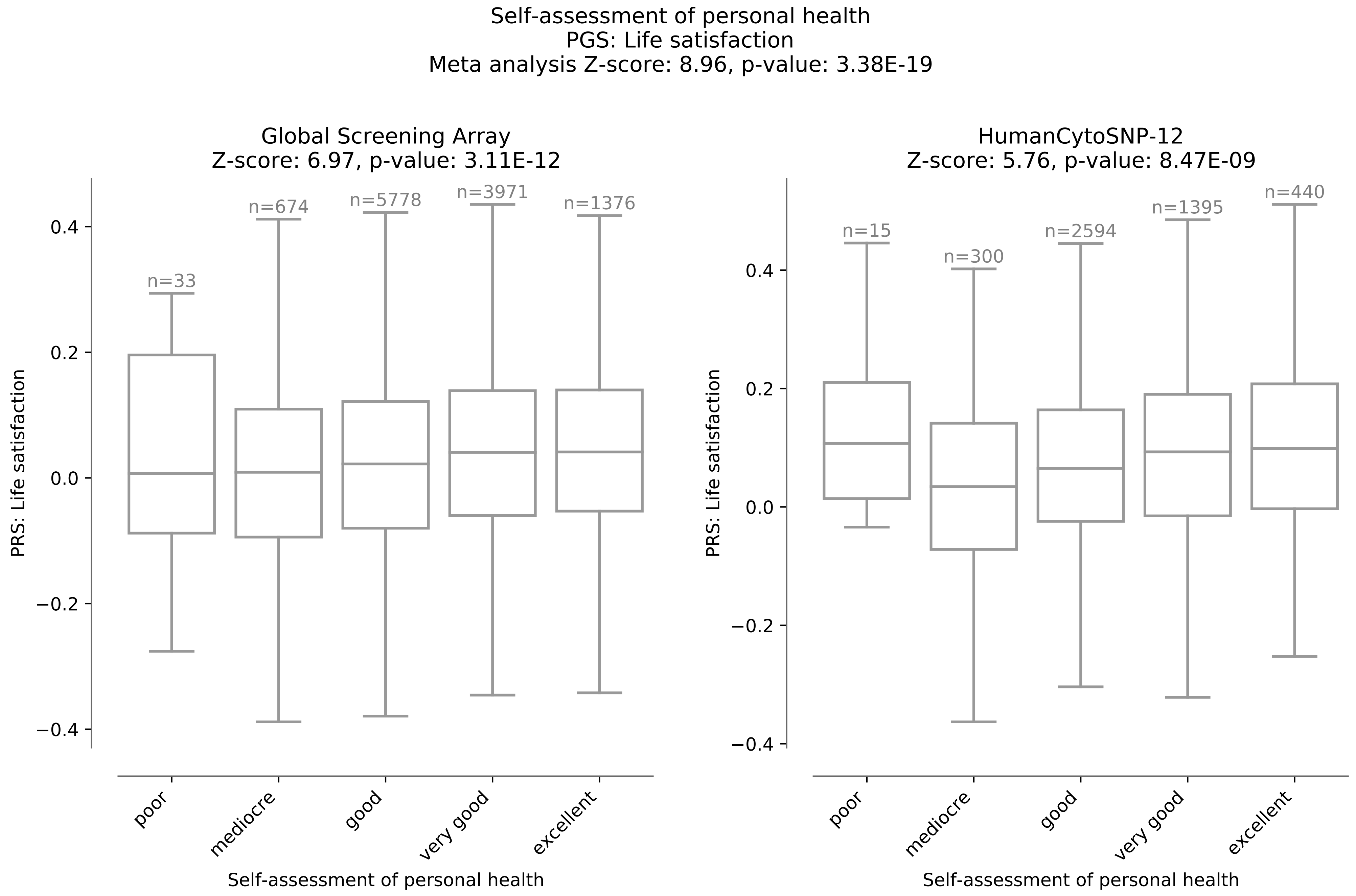
Self-assessment of personal health
PGS: Life satisfaction
Meta analysis Z-score: 8.96, p-value: 3.38E-19
Global Screening Array
Z-score: 6.97, p-value: 3.11E-12
HumanCytoSNP-12
Z-score: 5.76, p-value: 8.47E-09
PRS: Life satisfaction
n=33
n=674
n=5778
n=3971
n=1376
n=15
n=300
n=2594
n=1395
n=440
0.4
0.2
0.0
−0.2
−0.4
poor
mediocre
good
very good
excellent
Self-assessment of personal health

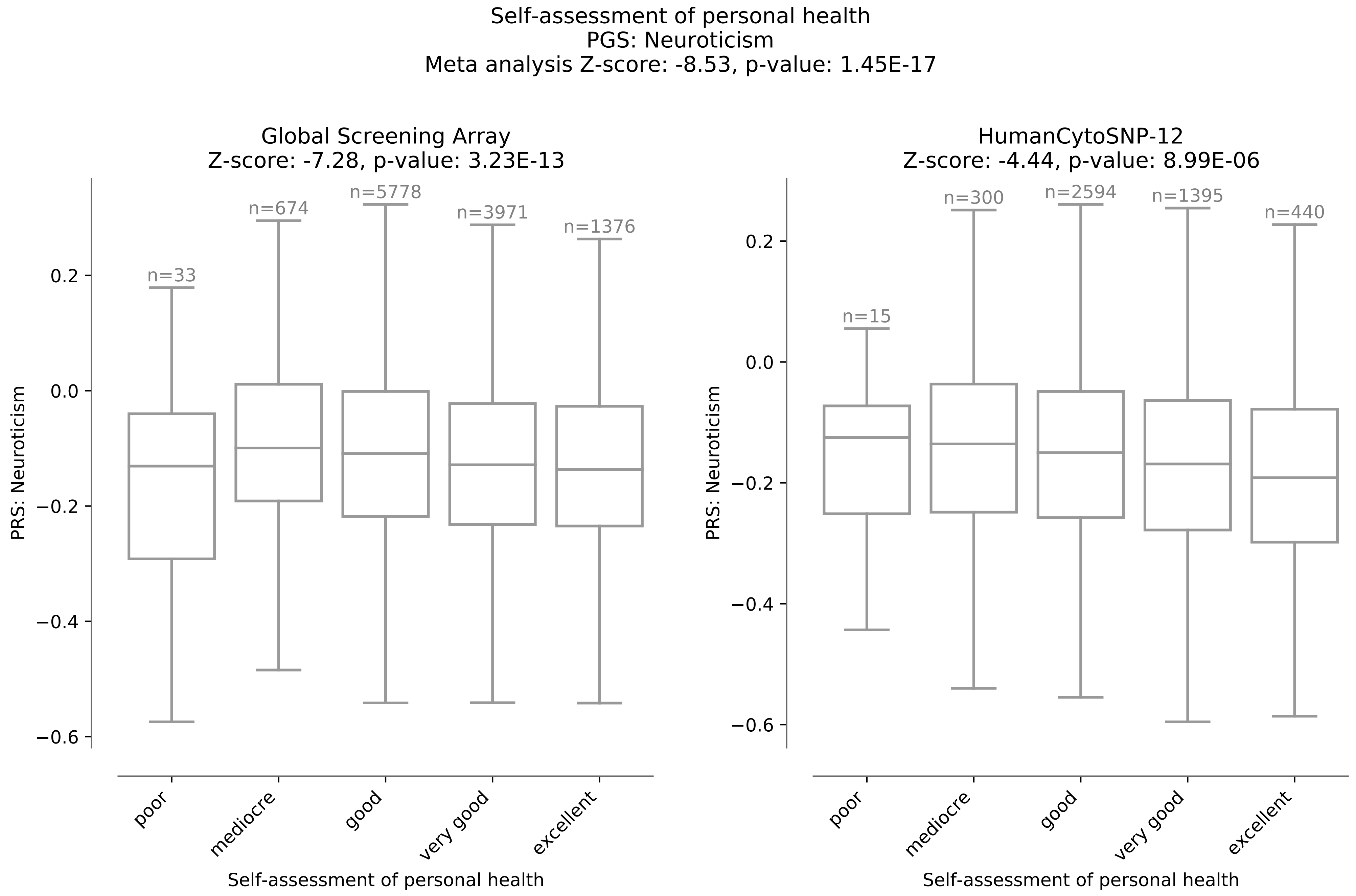
Self-assessment of personal health
PGS: Neuroticism
Meta analysis Z-score: -8.53, p-value: 1.45E-17
Global Screening Array
Z-score: -7.28, p-value: 3.23E-13
HumanCytoSNP-12
Z-score: -4.44, p-value: 8.99E-06
PRS: Neuroticism
0.2
0.0
−0.2
−0.4
−0.6
n=33
n=674
n=5778
n=3971
n=1376
n=15
n=300
n=2594
n=1395
n=440
poor
mediocre
good
very good
excellent
Self-assessment of personal health

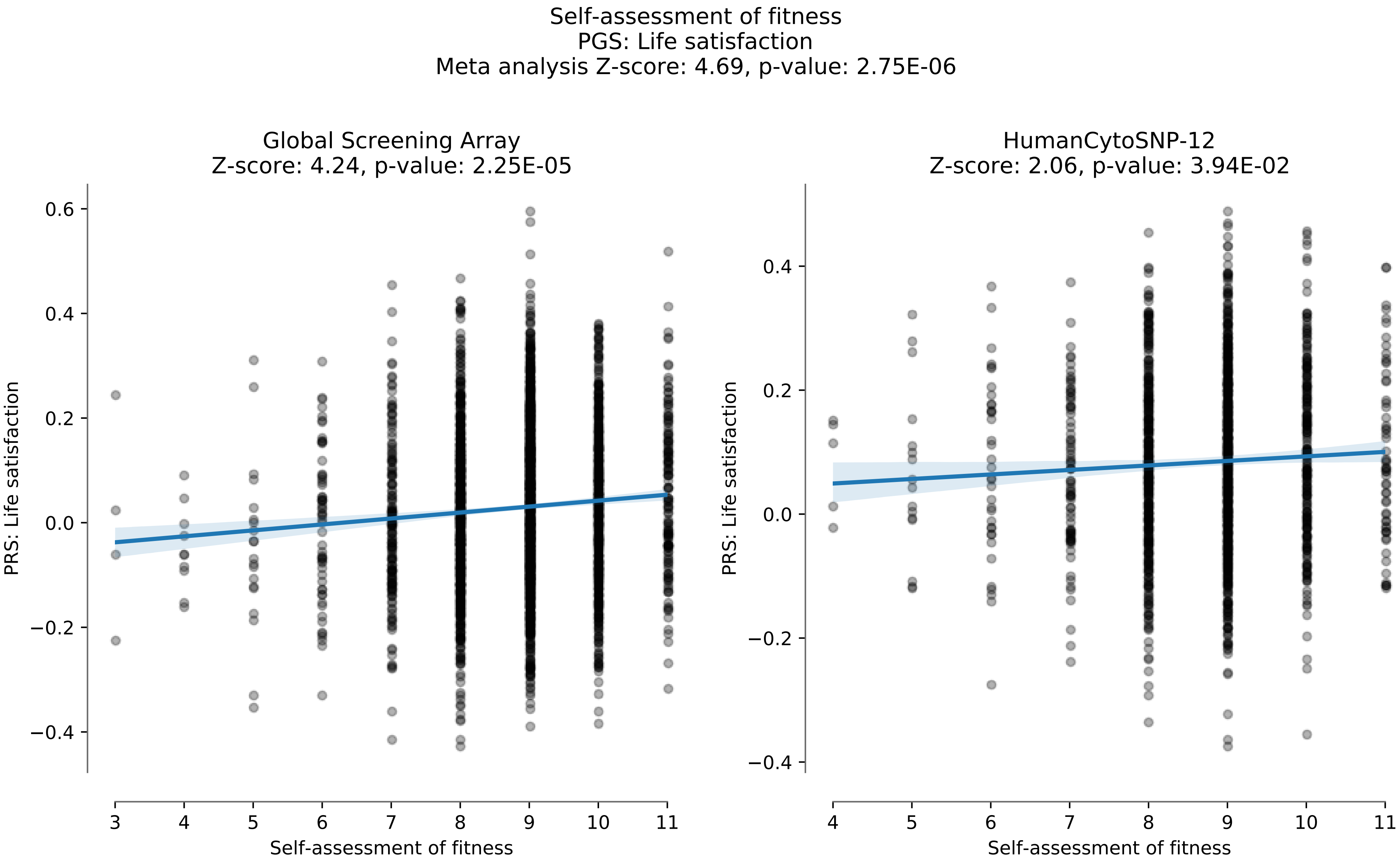

Self-assessment of fitness
PGS: Life satisfaction
Meta analysis Z-score: 4.69, p-value: 2.75E-06
Global Screening Array
Z-score: 4.24, p-value: 2.25E-05
HumanCytoSNP-12
Z-score: 2.06, p-value: 3.94E-02
PRS: Life satisfaction
Self-assessment of fitness
0.6
0.4
0.2
0.0
−0.2
−0.4
3
4
5
6
7
8
9
10
11

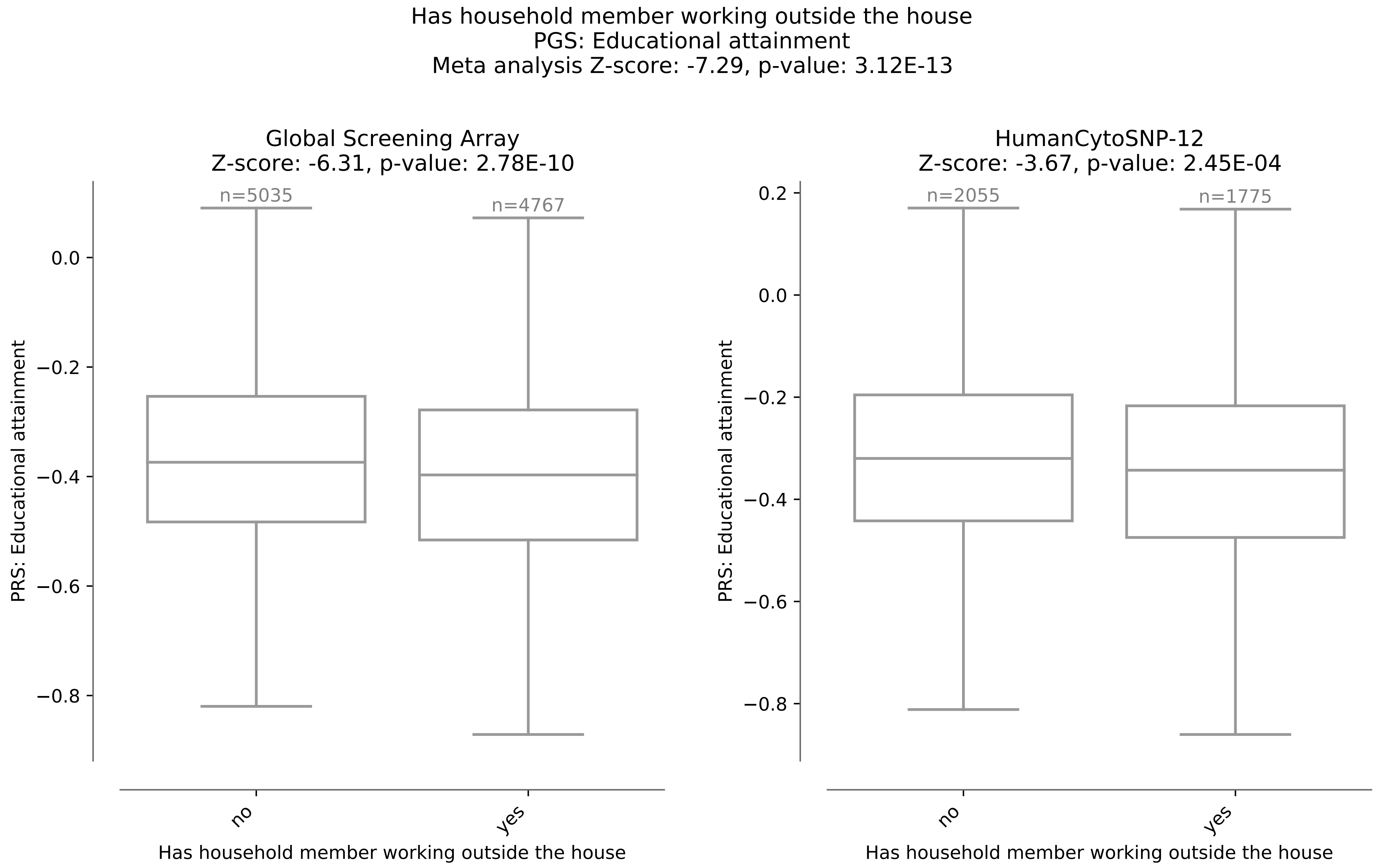
Has household member working outside the house
PGS: Educational attainment
Meta analysis Z-score: -7.29, p-value: 3.12E-13
Global Screening Array
Z-score: -6.31, p-value: 2.78E-10
n=5035
n=4767
PRS: Educational attainment
0.0
−0.2
−0.4
−0.6
−0.8
no
yes
Has household member working outside the house
HumanCytoSNP-12
Z-score: -3.67, p-value: 2.45E-04
n=2055
n=1775
PRS: Educational attainment
0.2
0.0
−0.2
−0.4
−0.6
−0.8
no
yes
Has household member working outside the house

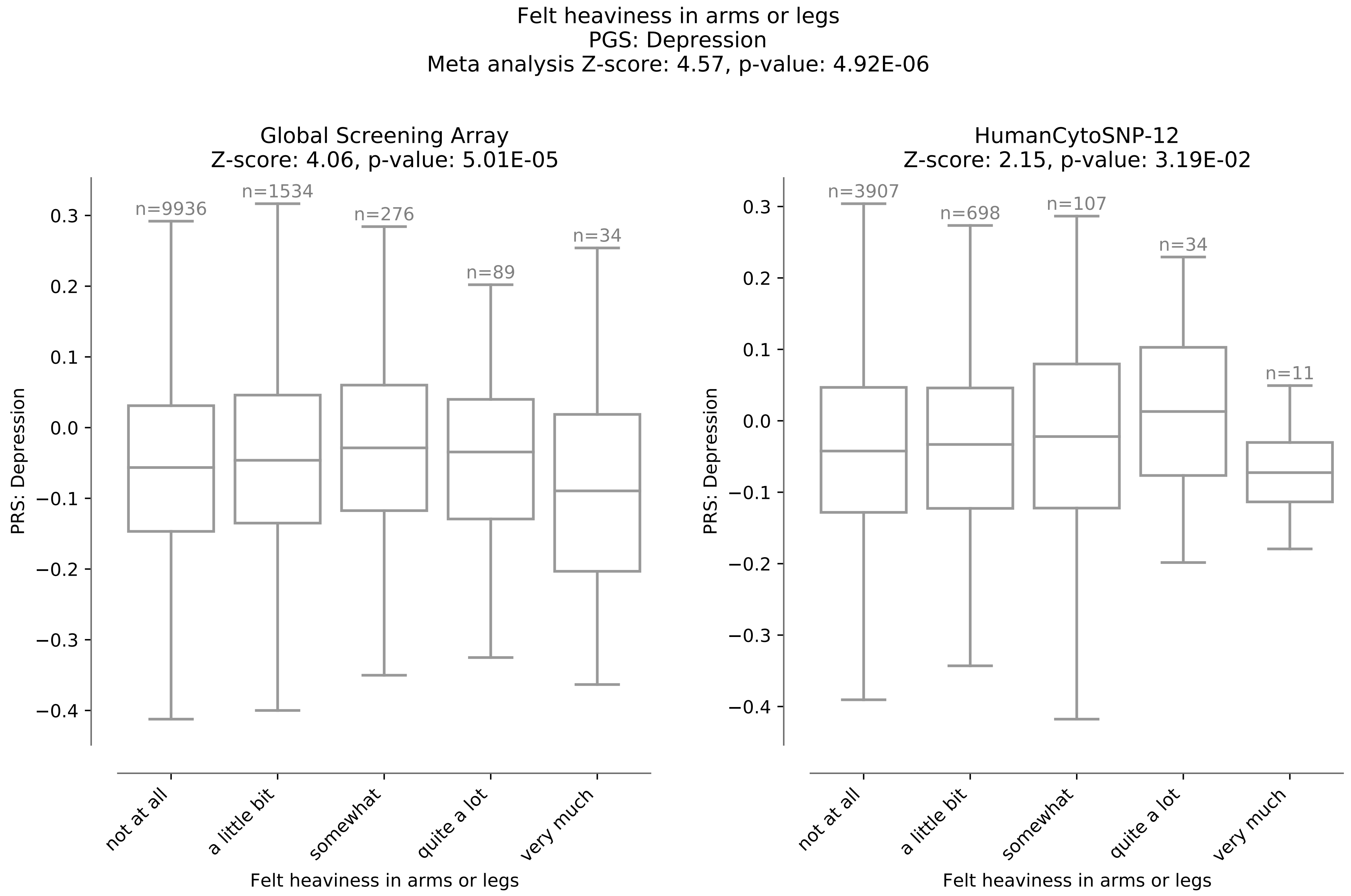

Felt heaviness in arms or legs
PGS: Depression
Meta analysis Z-score: 4.57, p-value: 4.92E-06
Global Screening Array
Z-score: 4.06, p-value: 5.01E-05
HumanCytoSNP-12
Z-score: 2.15, p-value: 3.19E-02
PRS: Depression
n=9936
n=1534
n=276
n=89
n=34
n=3907
n=698
n=107
n=34
n=11
0.3
0.2
0.1
0.0
−0.1
−0.2
−0.3
−0.4
not at all
a little bit
somewhat
quite a lot
very much
Felt heaviness in arms or legs

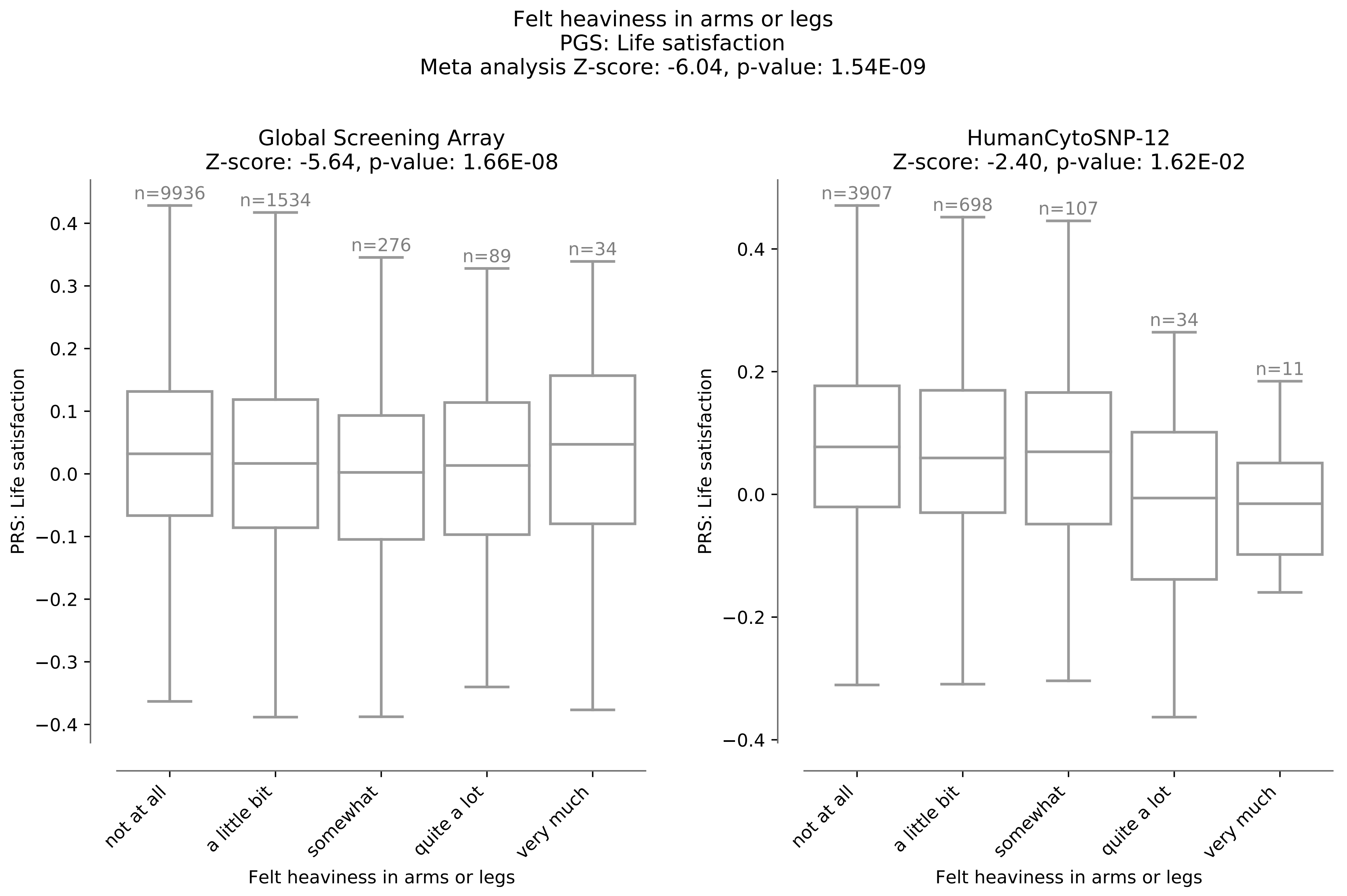
Felt heaviness in arms or legs
PGS: Life satisfaction
Meta analysis Z-score: -6.04, p-value: 1.54E-09
Global Screening Array
Z-score: -5.64, p-value: 1.66E-08
n=9936
n=1534
n=276
n=89
n=34
PRS: Life satisfaction
0.4
0.3
0.2
0.1
0.0
−0.1
−0.2
−0.3
−0.4
not at all
a little bit
somewhat
quite a lot
very much
Felt heaviness in arms or legs
HumanCytoSNP-12
Z-score: -2.40, p-value: 1.62E-02
n=3907
n=698
n=107
n=34
n=11
PRS: Life satisfaction
0.4
0.2
0.0
−0.2
−0.4
not at all
a little bit
somewhat
quite a lot
very much
Felt heaviness in arms or legs

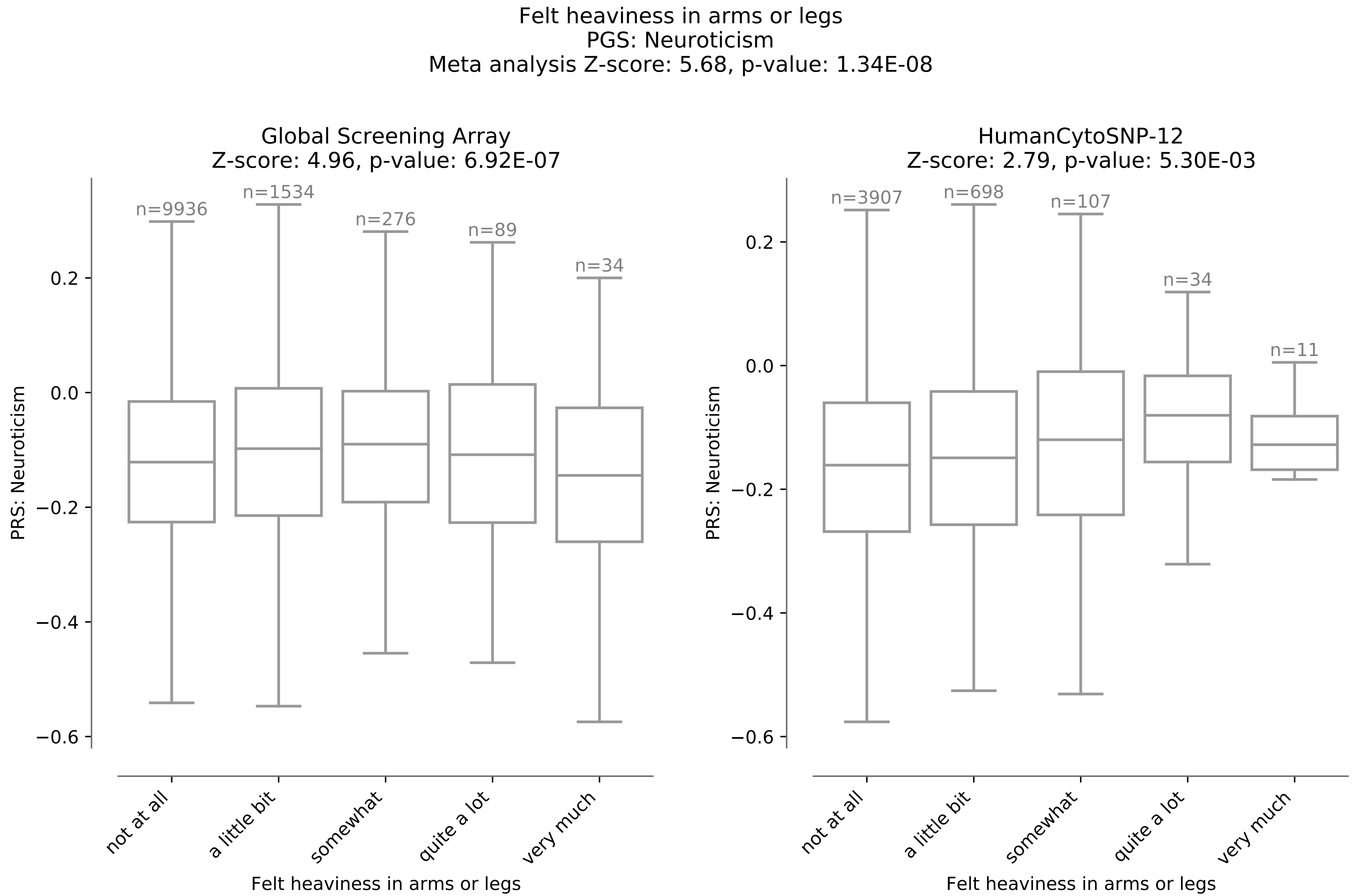
Felt heaviness in arms or legs
PGS: Neuroticism
Meta analysis Z-score: 5.68, p-value: 1.34E-08
Global Screening Array
Z-score: 4.96, p-value: 6.92E-07
n=9936
n=1534
n=276
n=89
n=34
PRS: Neuroticism
0.2
0.0
−0.2
−0.4
−0.6
not at all
a little bit
somewhat
quite a lot
very much
Felt heaviness in arms or legs
HumanCytoSNP-12
Z-score: 2.79, p-value: 5.30E-03
n=3907
n=698
n=107
n=34
n=11
PRS: Neuroticism
0.2
0.0
−0.2
−0.4
−0.6
not at all
a little bit
somewhat
quite a lot
very much
Felt heaviness in arms or legs
