# Supplementary material for: Increased genetic contribution to wellbeing during the COVID-19 pandemic
Source: PLoS Genet. 2022 May 19;18(5):e1010135. doi: 10.1371/journal.pgen.1010135 (PMC9119461; doi:10.1371/journal.pgen.1010135)
Supplement: S4 Fig — Each significant interaction between time (denoted in days starting from March 30, 2020) and PGS visualized over time, stratified by the PGS for which the interaction was observed to be significant. The 10th percentile, the median and the 90th percentile illustrate how the PGS interacts with time. On top the complete model for its respective outcome measure is presented. Herein the contributions of all terms are considered, and the temporal aspect of the outcome variable can be seen and compared with the interaction effect. The shaded areas represent the 95% confidence interval of the model fit. On the bottom only the relative contribution of the PGS is taken into consideration. Converging percentile lines indicate that the PGSs have a decreasing effect on the outcome measure. Contrariwise, diverging percentile lines indicate that a PGS has an increasing effect on the outcome measure showing that genetics play an increasingly important role. (PDF) [file pgen.1010135.s017.pdf]

Model fitted on 'Quality of life' stratified by 'Life satisfaction'

Interaction P−value: 3.1×10^{-3} Z−score: 2.96

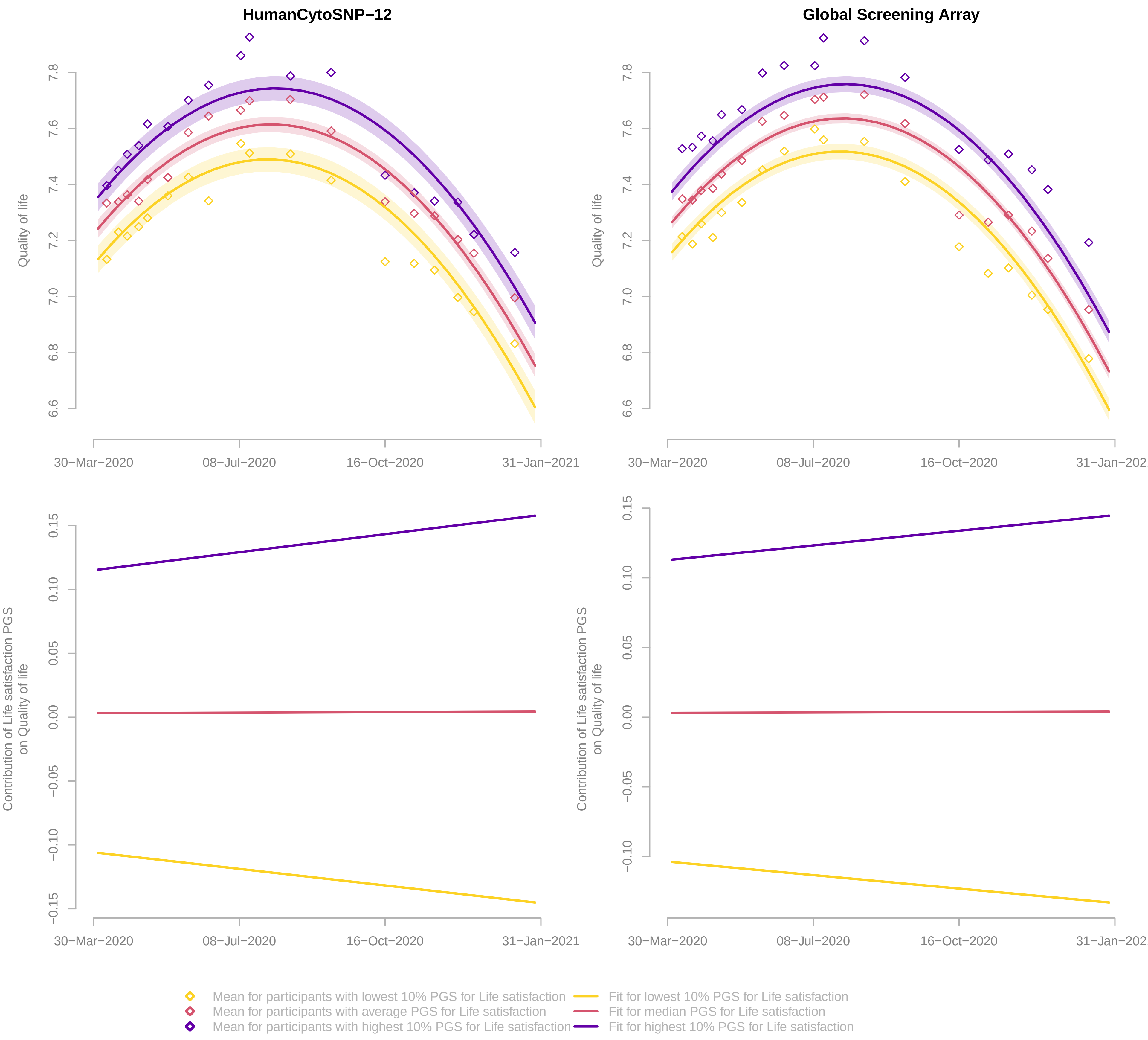

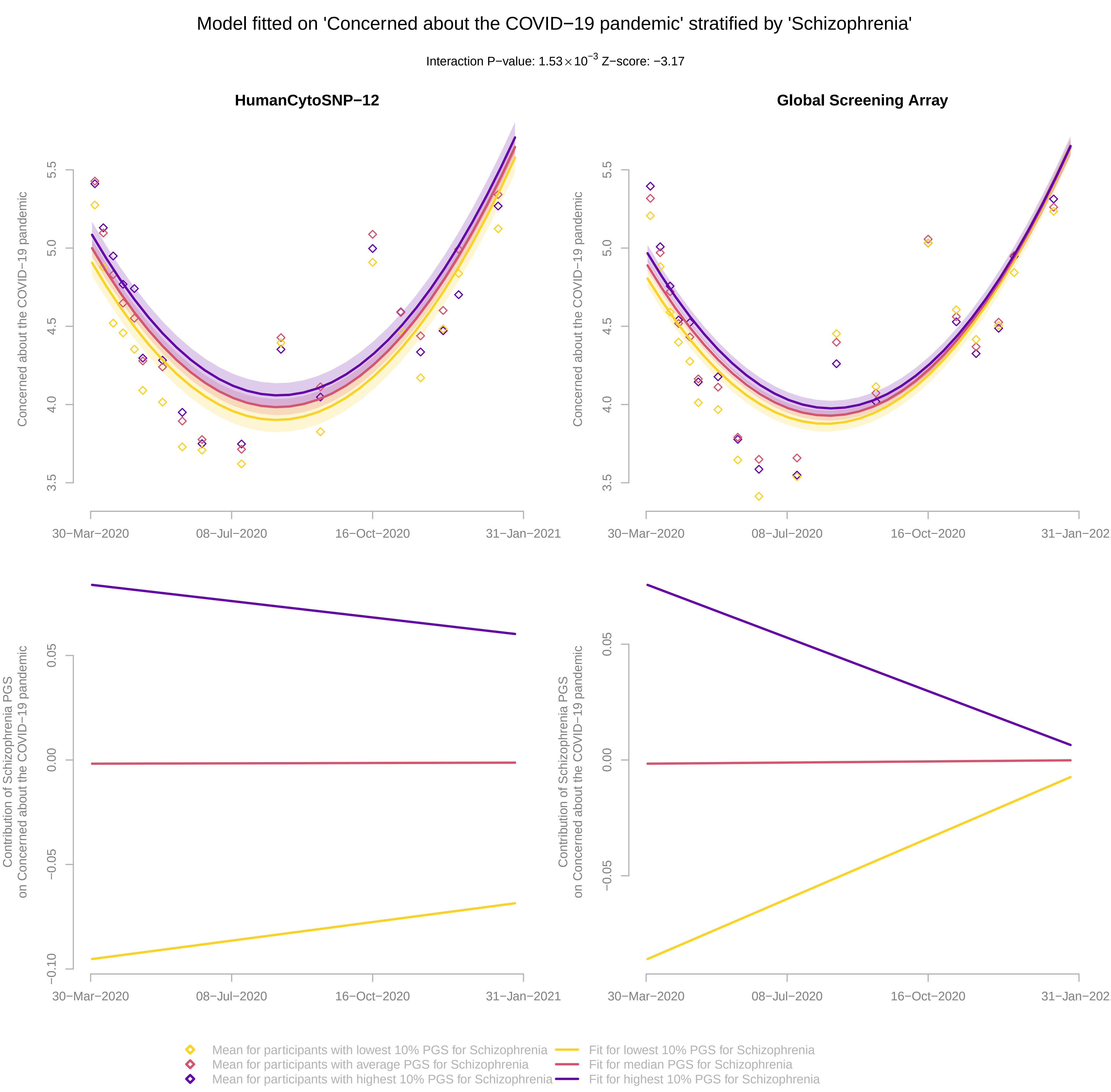

Model fitted on 'Concerned about the COVID−19 pandemic' stratified by 'Schizophrenia'
Interaction P−value: 1.53 × 10^−3 Z−score: −3.17
HumanCytoSNP−12
Global Screening Array
Concerned about the COVID−19 pandemic
Contribution of Schizophrenia PGS on Concerned about the COVID−19 pandemic
30−Mar−2020
08−Jul−2020
16−Oct−2020
31−Jan−2021
Mean for participants with lowest 10% PGS for Schizophrenia
Mean for participants with average PGS for Schizophrenia
Mean for participants with highest 10% PGS for Schizophrenia
Fit for lowest 10% PGS for Schizophrenia
Fit for median PGS for Schizophrenia
Fit for highest 10% PGS for Schizophrenia

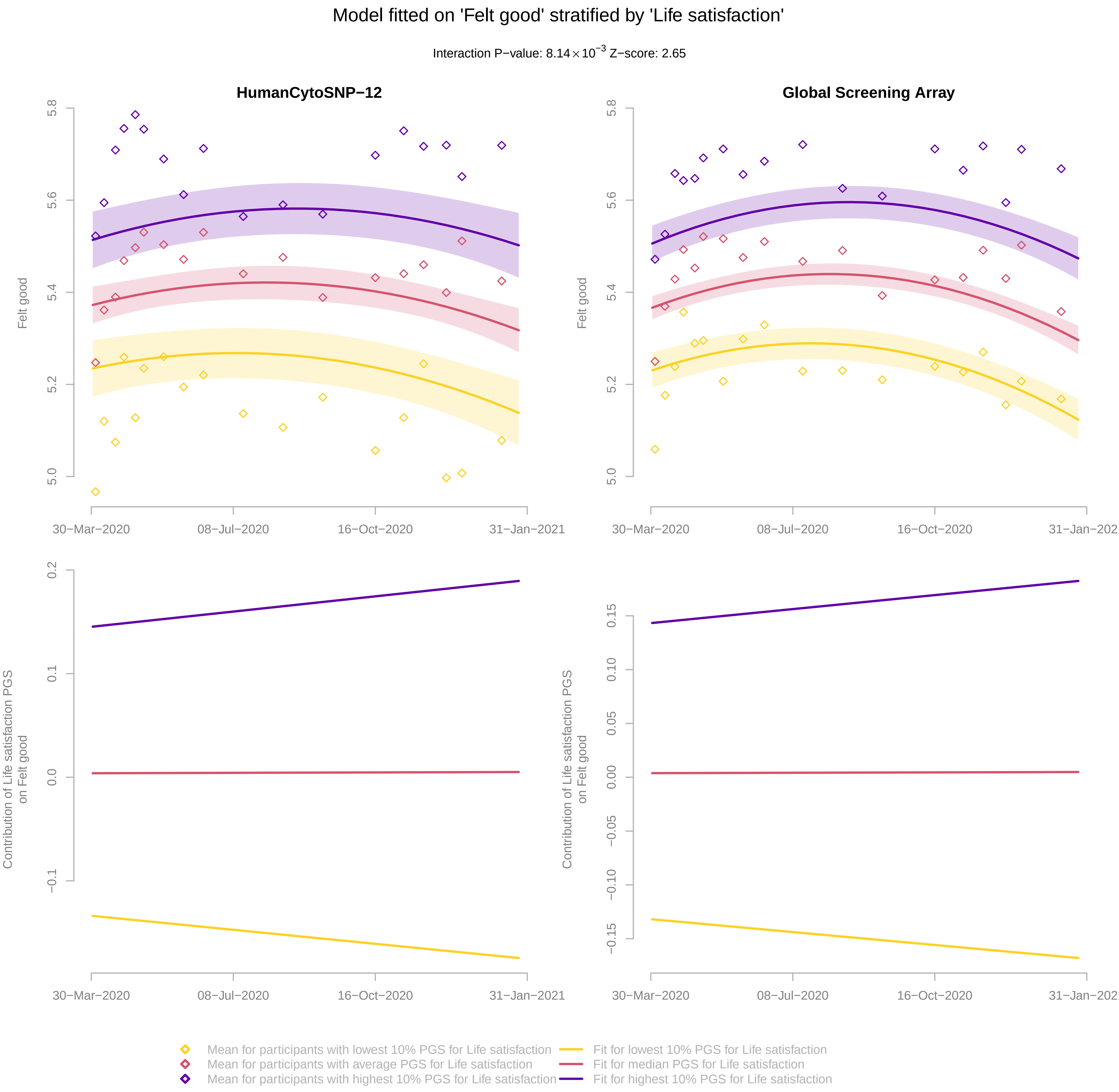
Model fitted on 'Felt good' stratified by 'Life satisfaction'
Interaction P−value: 8.14 × 10^−3 Z−score: 2.65
HumanCytoSNP−12
Global Screening Array
Felt good
5.0
5.2
5.4
5.6
5.8
30−Mar−2020
08−Jul−2020
16−Oct−2020
31−Jan−2021
Contribution of Life satisfaction PGS on Felt good
−0.1
0.0
0.1
0.2
−0.15
−0.10
−0.05
0.00
0.05
0.10
0.15
Mean for participants with lowest 10% PGS for Life satisfaction
Mean for participants with average PGS for Life satisfaction
Mean for participants with highest 10% PGS for Life satisfaction
Fit for lowest 10% PGS for Life satisfaction
Fit for median PGS for Life satisfaction
Fit for highest 10% PGS for Life satisfaction

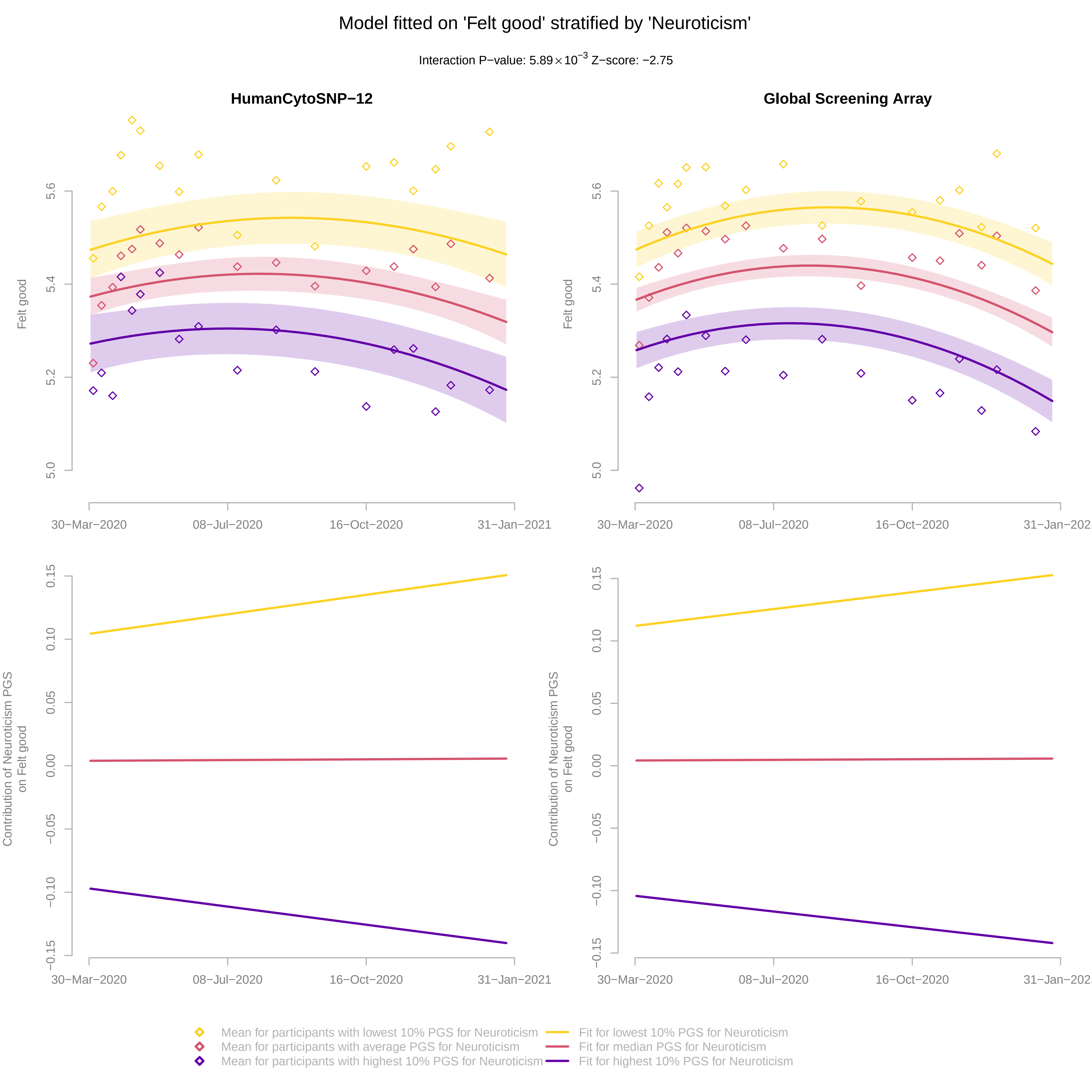

Model fitted on 'Felt good' stratified by 'Neuroticism'
Interaction P−value: 5.89×10−3 Z−score: −2.75
HumanCytoSNP−12
Global Screening Array
Felt good
5.0
5.2
5.4
5.6
30−Mar−2020
08−Jul−2020
16−Oct−2020
31−Jan−2021
Contribution of Neuroticism PGS on Felt good
−0.15
−0.10
−0.05
0.00
0.05
0.10
0.15
Mean for participants with lowest 10% PGS for Neuroticism
Mean for participants with average PGS for Neuroticism
Mean for participants with highest 10% PGS for Neuroticism
Fit for lowest 10% PGS for Neuroticism
Fit for median PGS for Neuroticism
Fit for highest 10% PGS for Neuroticism

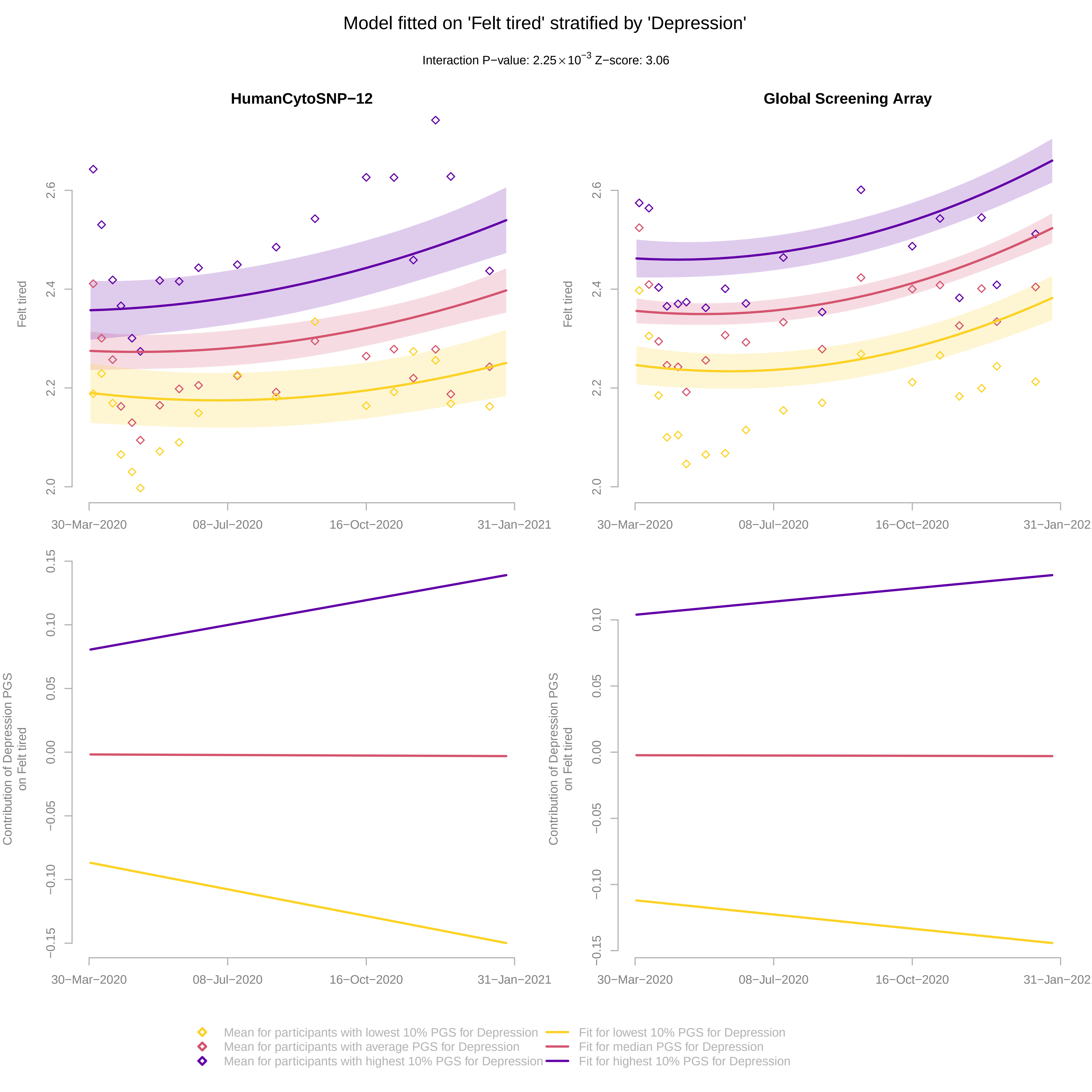

Model fitted on 'Felt tired' stratified by 'Depression'
Interaction P−value: 2.25 × 10^−3 Z−score: 3.06
HumanCytoSNP−12
Global Screening Array
Felt tired
Contribution of Depression PGS on Felt tired
30−Mar−2020
08−Jul−2020
16−Oct−2020
31−Jan−2021
Mean for participants with lowest 10% PGS for Depression
Mean for participants with average PGS for Depression
Mean for participants with highest 10% PGS for Depression
Fit for lowest 10% PGS for Depression
Fit for median PGS for Depression
Fit for highest 10% PGS for Depression

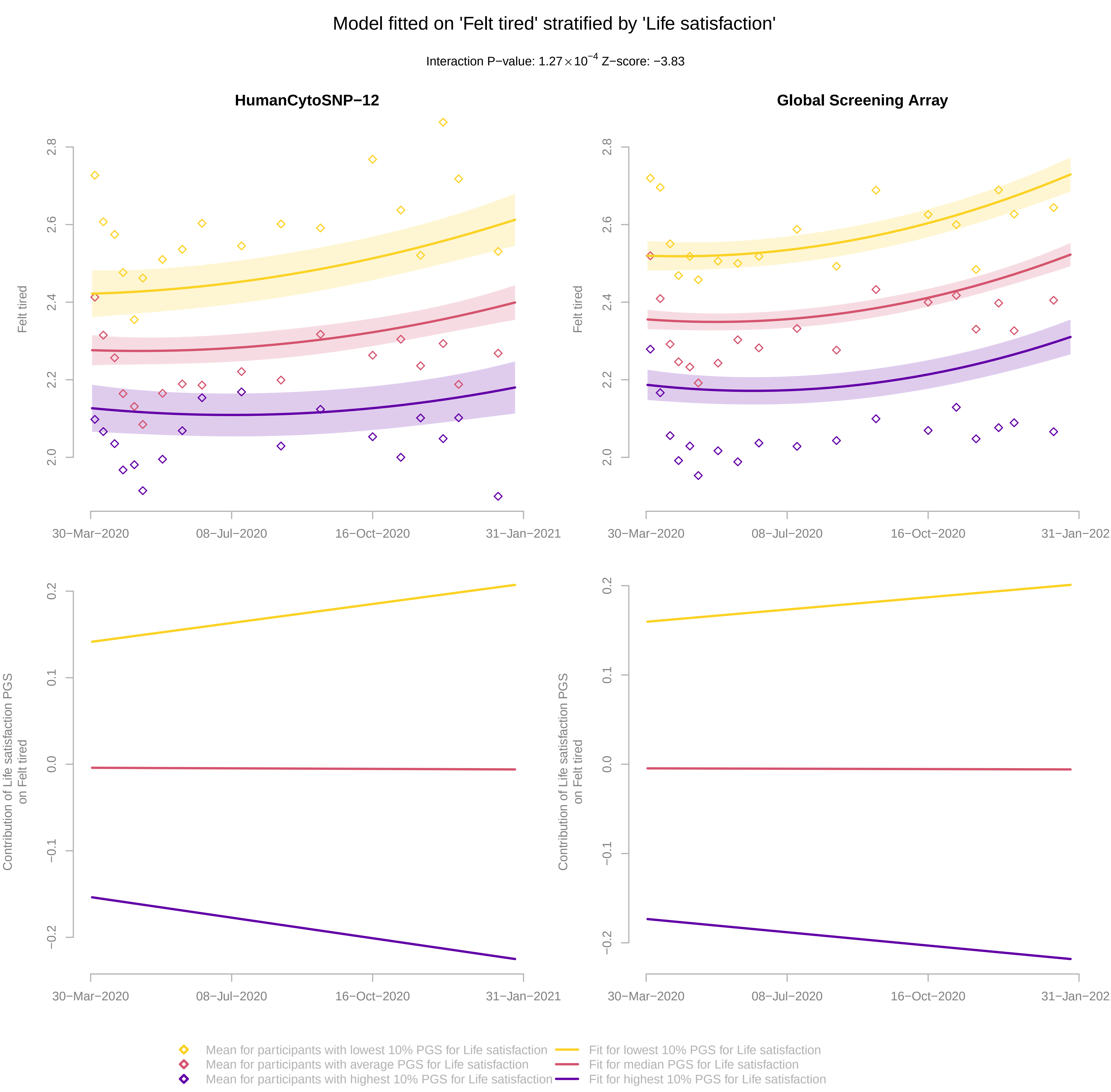

Model fitted on 'Felt tired' stratified by 'Life satisfaction'
Interaction P−value: 1.27×10^{-4} Z−score: −3.83
HumanCytoSNP−12
Global Screening Array
Felt tired
Contribution of Life satisfaction PGS on Felt tired
30−Mar−2020
08−Jul−2020
16−Oct−2020
31−Jan−2021
Mean for participants with lowest 10% PGS for Life satisfaction
Mean for participants with average PGS for Life satisfaction
Mean for participants with highest 10% PGS for Life satisfaction
Fit for lowest 10% PGS for Life satisfaction
Fit for median PGS for Life satisfaction
Fit for highest 10% PGS for Life satisfaction

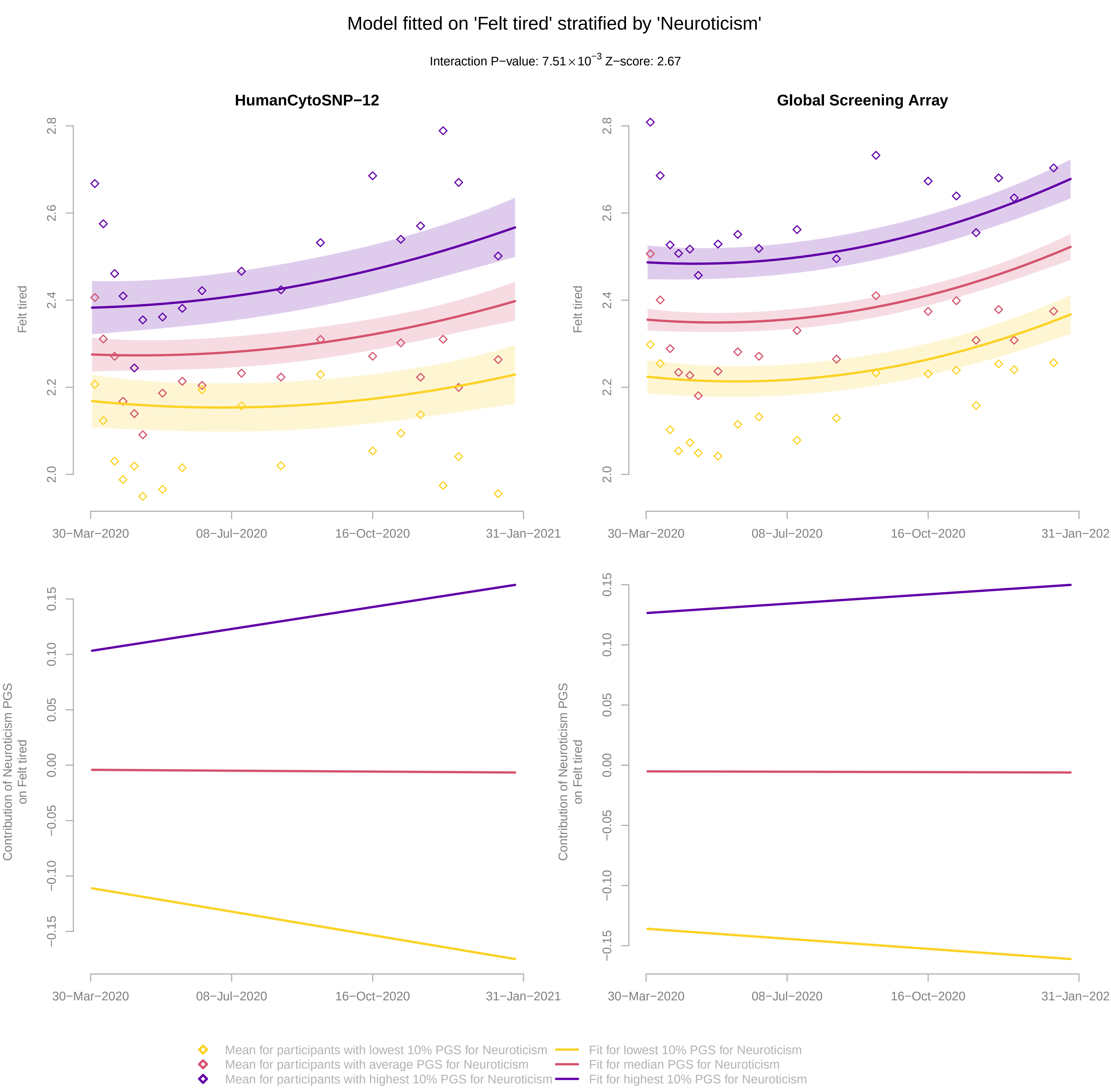
Model fitted on 'Felt tired' stratified by 'Neuroticism'
Interaction P−value: 7.51×10^{-3} Z−score: 2.67
HumanCytoSNP−12
Global Screening Array
Felt tired
2.0
2.2
2.4
2.6
2.8
30−Mar−2020
08−Jul−2020
16−Oct−2020
31−Jan−2021
Contribution of Neuroticism PGS on Felt tired
−0.15
−0.10
−0.05
0.00
0.05
0.10
0.15
Mean for participants with lowest 10% PGS for Neuroticism
Mean for participants with average PGS for Neuroticism
Mean for participants with highest 10% PGS for Neuroticism
Fit for lowest 10% PGS for Neuroticism
Fit for median PGS for Neuroticism
Fit for highest 10% PGS for Neuroticism

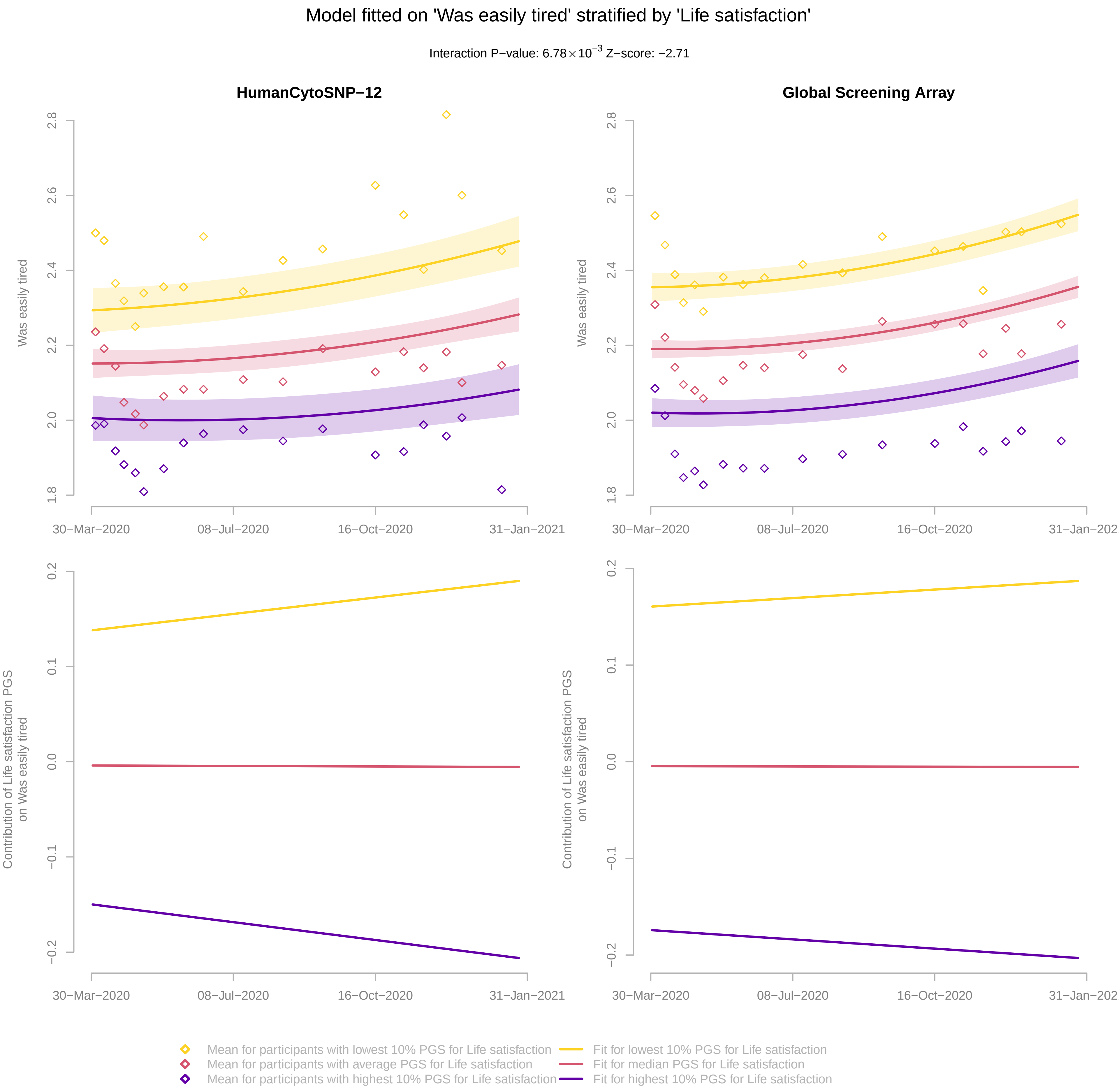

Model fitted on 'Was easily tired' stratified by 'Life satisfaction'
Interaction P−value: 6.78 × 10−3 Z−score: −2.71
HumanCytoSNP−12
Global Screening Array
Was easily tired
Contribution of Life satisfaction PGS on Was easily tired
30−Mar−2020
08−Jul−2020
16−Oct−2020
31−Jan−2021
Mean for participants with lowest 10% PGS for Life satisfaction
Mean for participants with average PGS for Life satisfaction
Mean for participants with highest 10% PGS for Life satisfaction
Fit for lowest 10% PGS for Life satisfaction
Fit for median PGS for Life satisfaction
Fit for highest 10% PGS for Life satisfaction

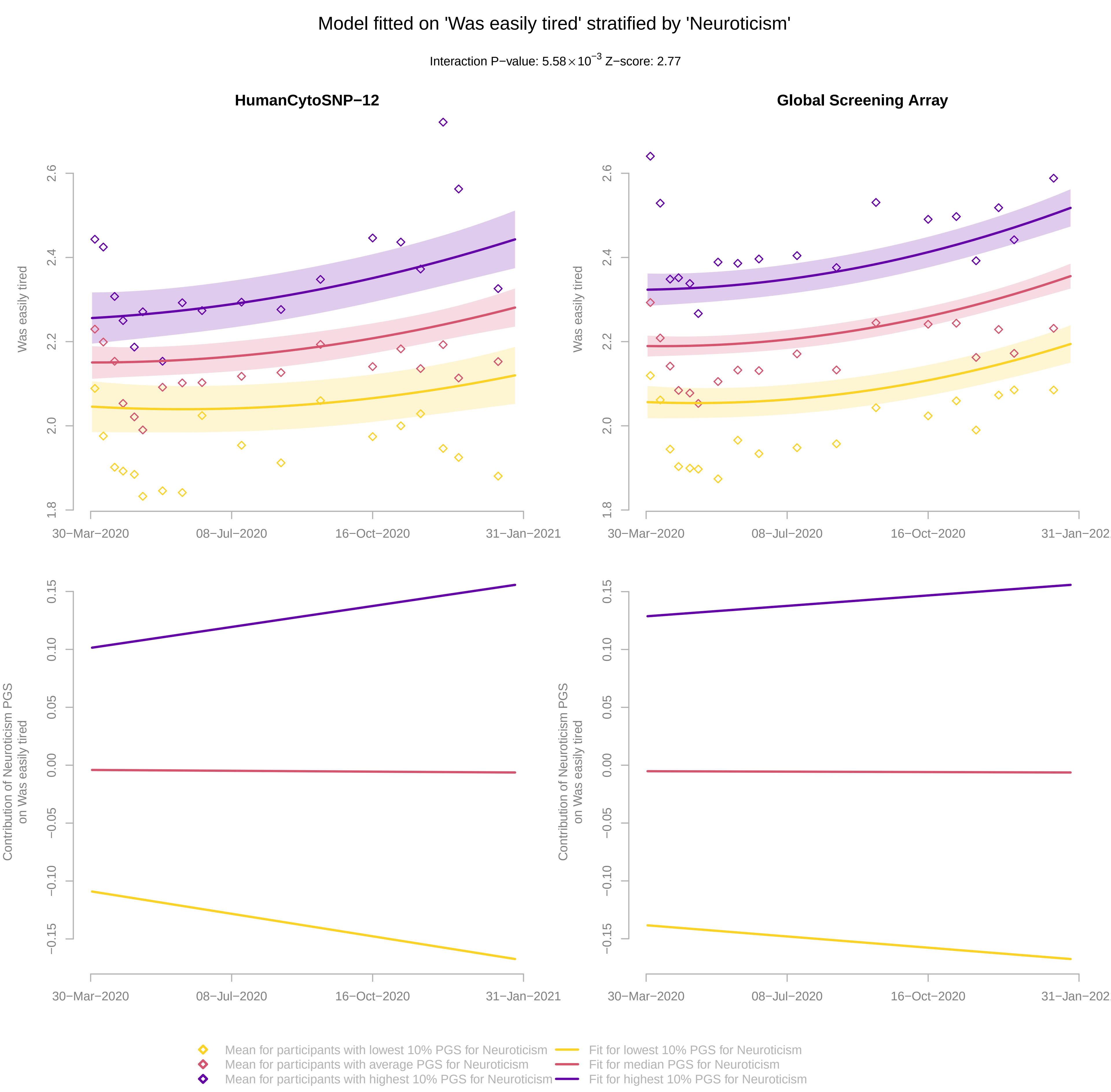

Model fitted on 'Was easily tired' stratified by 'Neuroticism'
Interaction P−value: 5.58 × 10^−3 Z−score: 2.77
HumanCytoSNP−12
Global Screening Array
Was easily tired
Contribution of Neuroticism PGS on Was easily tired
1.8
2.0
2.2
2.4
2.6
−0.15
−0.10
−0.05
0.00
0.05
0.10
0.15
30−Mar−2020
08−Jul−2020
16−Oct−2020
31−Jan−2021
Mean for participants with lowest 10% PGS for Neuroticism
Mean for participants with average PGS for Neuroticism
Mean for participants with highest 10% PGS for Neuroticism
Fit for lowest 10% PGS for Neuroticism
Fit for median PGS for Neuroticism
Fit for highest 10% PGS for Neuroticism

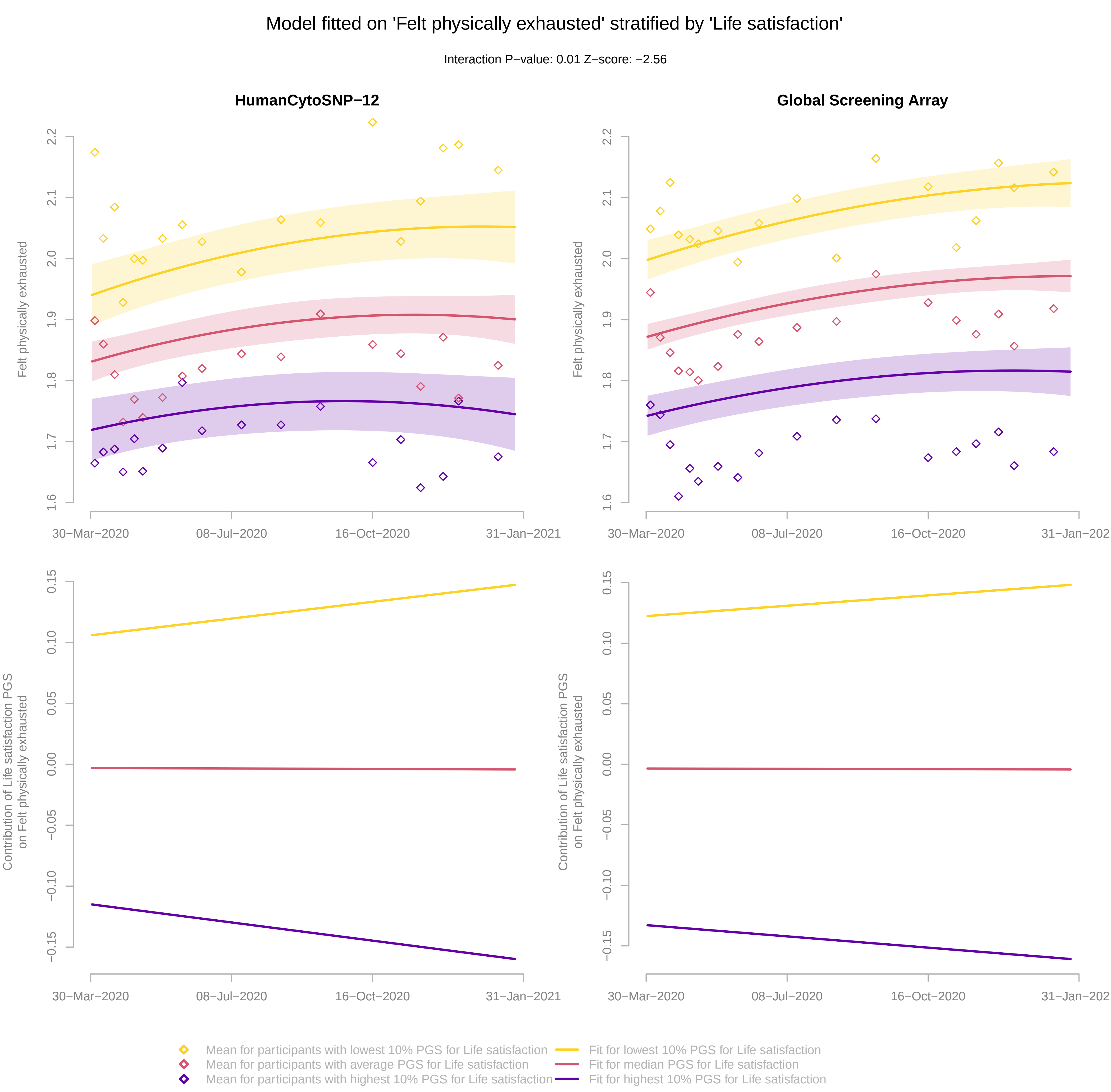

Model fitted on 'Felt physically exhausted' stratified by 'Life satisfaction'
Interaction P−value: 0.01 Z−score: −2.56
HumanCytoSNP−12
Global Screening Array
Felt physically exhausted
Contribution of Life satisfaction PGS on Felt physically exhausted
30−Mar−2020
08−Jul−2020
16−Oct−2020
31−Jan−2021
Mean for participants with lowest 10% PGS for Life satisfaction
Mean for participants with average PGS for Life satisfaction
Mean for participants with highest 10% PGS for Life satisfaction
Fit for lowest 10% PGS for Life satisfaction
Fit for median PGS for Life satisfaction
Fit for highest 10% PGS for Life satisfaction

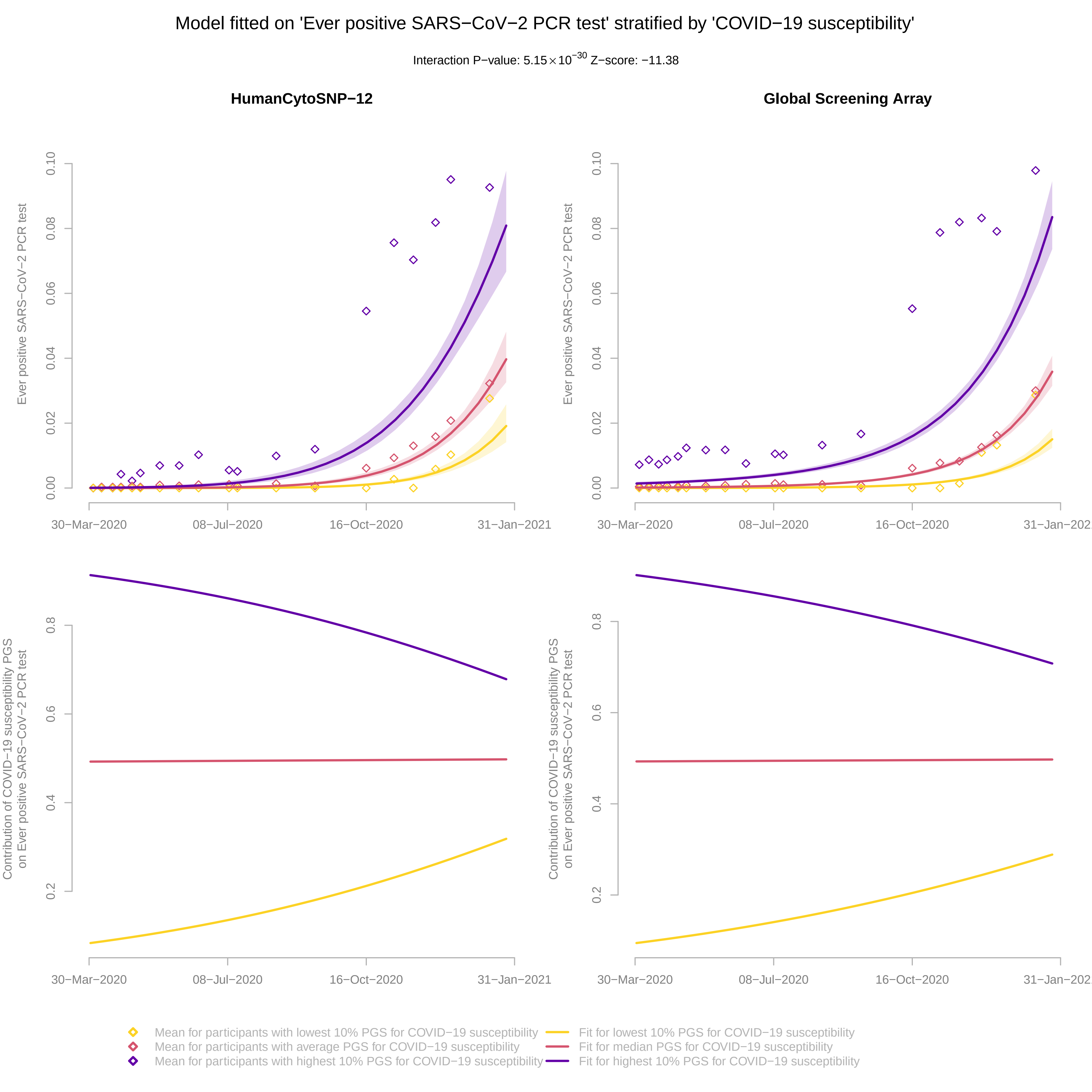

Model fitted on 'Ever positive SARS−CoV−2 PCR test' stratified by 'COVID−19 susceptibility'
Interaction P−value: 5.15×10^−30 Z−score: −11.38
HumanCytoSNP−12
Global Screening Array
Ever positive SARS−CoV−2 PCR test
Contribution of COVID−19 susceptibility PGS on Ever positive SARS−CoV−2 PCR test
30−Mar−2020
08−Jul−2020
16−Oct−2020
31−Jan−2021
Mean for participants with lowest 10% PGS for COVID−19 susceptibility
Mean for participants with average PGS for COVID−19 susceptibility
Mean for participants with highest 10% PGS for COVID−19 susceptibility
Fit for lowest 10% PGS for COVID−19 susceptibility
Fit for median PGS for COVID−19 susceptibility
Fit for highest 10% PGS for COVID−19 susceptibility
