# Supplementary material for: Increased genetic contribution to wellbeing during the COVID-19 pandemic
Source: PLoS Genet. 2022 May 19;18(5):e1010135. doi: 10.1371/journal.pgen.1010135 (PMC9119461; doi:10.1371/journal.pgen.1010135)
Supplement: S8 Fig — We validated our findings by fitting models for each PGS and each question separately for each questionnaire. The regression coefficients of the PGS were extracted from the models and plotted according to the date of each questionnaire. The error bars represent the 95% confidence interval for the regression coefficients. The plots showed a change in regression coefficient over time which indicates that the PGS explains less or more variance of the question outcome over time. (PDF) [file pgen.1010135.s021.pdf]

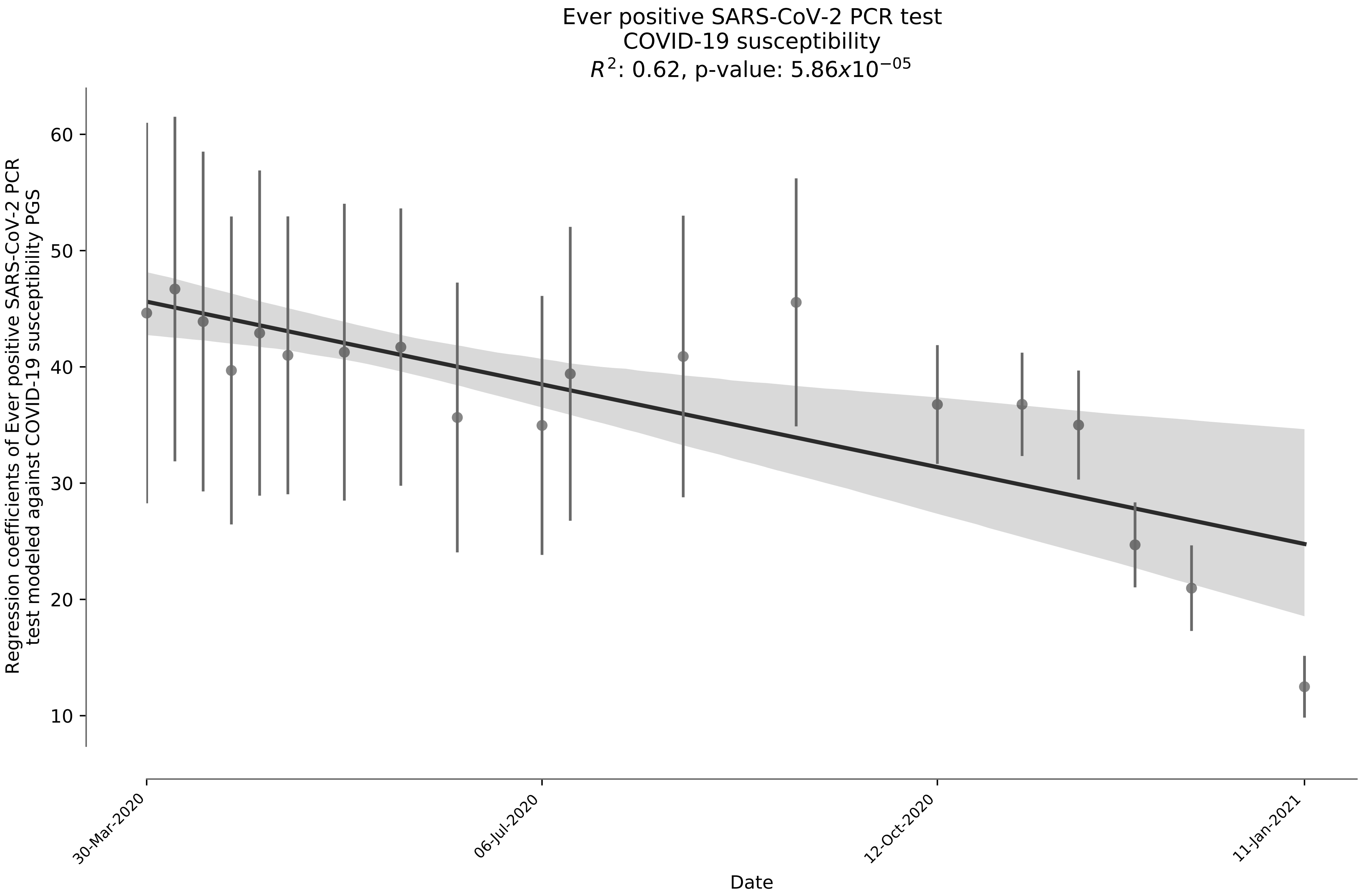

Ever positive SARS-CoV-2 PCR test
COVID-19 susceptibility
R^2: 0.62, p-value: $5.86x10^{-05}$
Regression coefficients of Ever positive SARS-CoV-2 PCR test modeled against COVID-19 susceptibility PGS
60
50
40
30
20
10
30-Mar-2020
06-Jul-2020
12-Oct-2020
11-Jan-2021
Date

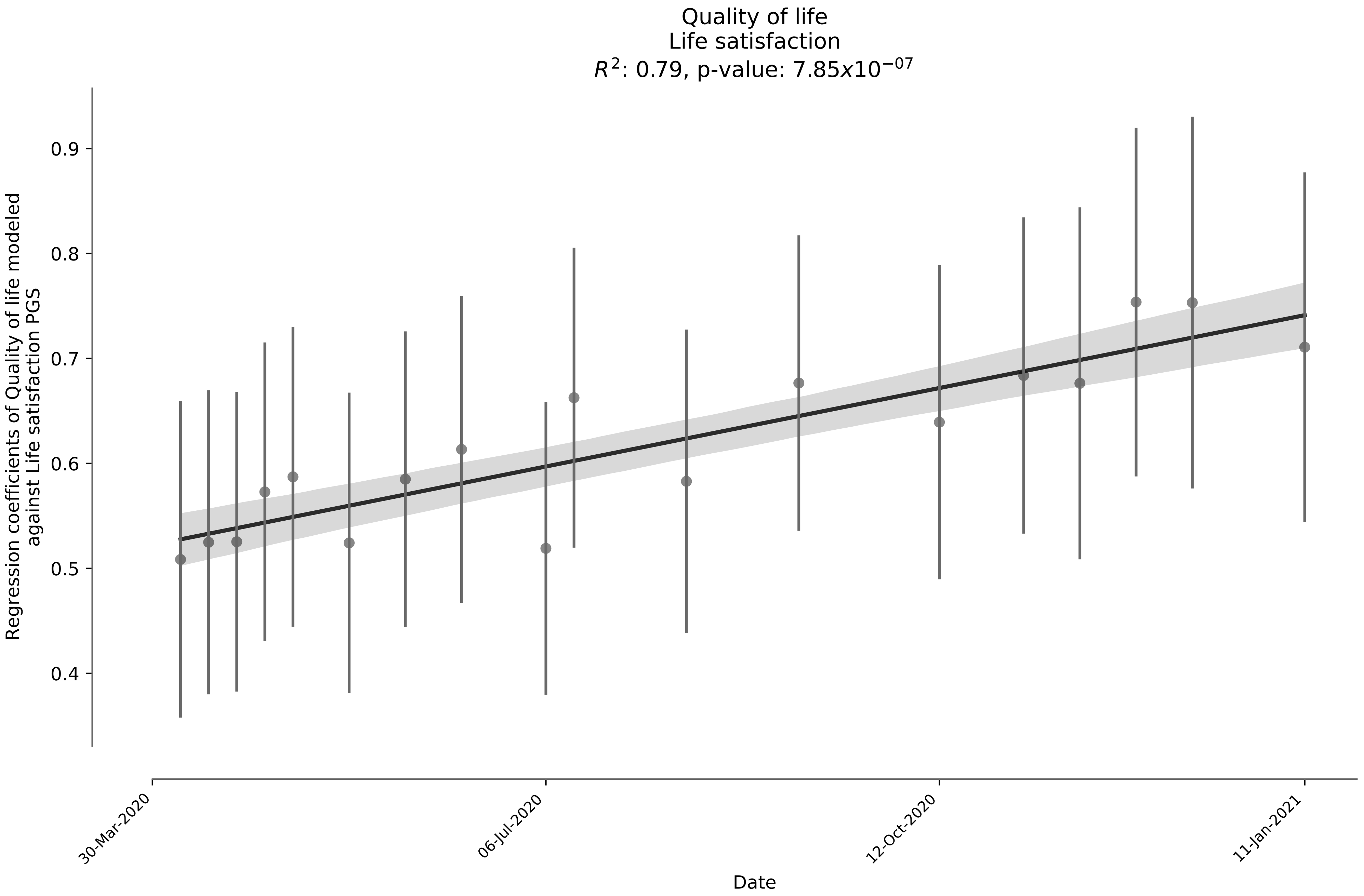
Quality of life
Life satisfaction
R^2: 0.79, p-value: $7.85x10^{-07}$
Regression coefficients of Quality of life modeled against Life satisfaction PGS
0.4
0.5
0.6
0.7
0.8
0.9
30-Mar-2020
06-Jul-2020
12-Oct-2020
11-Jan-2021
Date

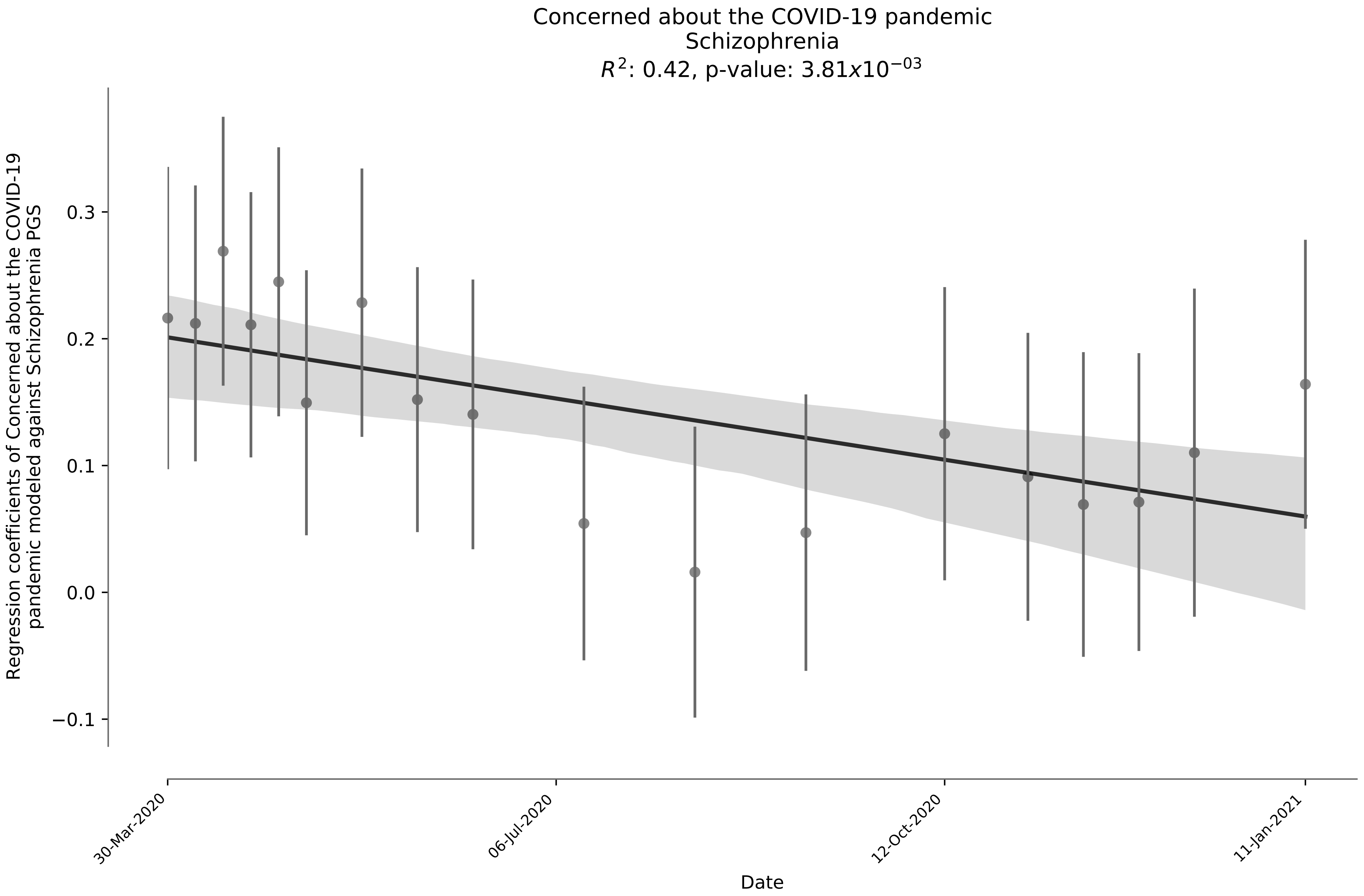

Concerned about the COVID-19 pandemic
Schizophrenia
R^2: 0.42, p-value: $3.81x10^{-03}$
Regression coefficients of Concerned about the COVID-19 pandemic modeled against Schizophrenia PGS
0.3
0.2
0.1
0.0
−0.1
30-Mar-2020
06-Jul-2020
12-Oct-2020
11-Jan-2021
Date

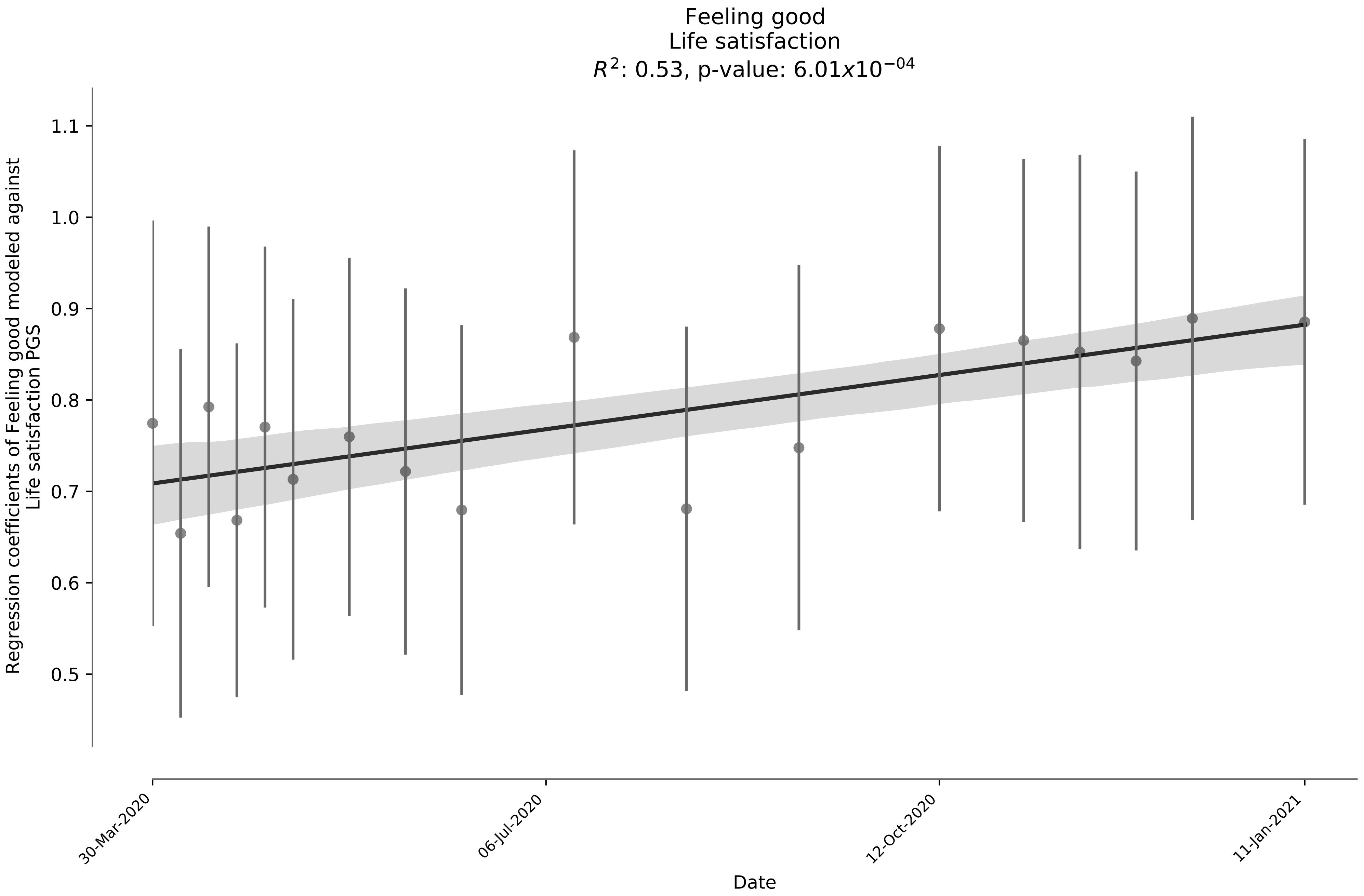
Feeling good
Life satisfaction
R^2: 0.53, p-value: $6.01x10^{-04}$
Regression coefficients of Feeling good modeled against
Life satisfaction PGS
1.1
1.0
0.9
0.8
0.7
0.6
0.5
30-Mar-2020
06-Jul-2020
12-Oct-2020
11-Jan-2021
Date

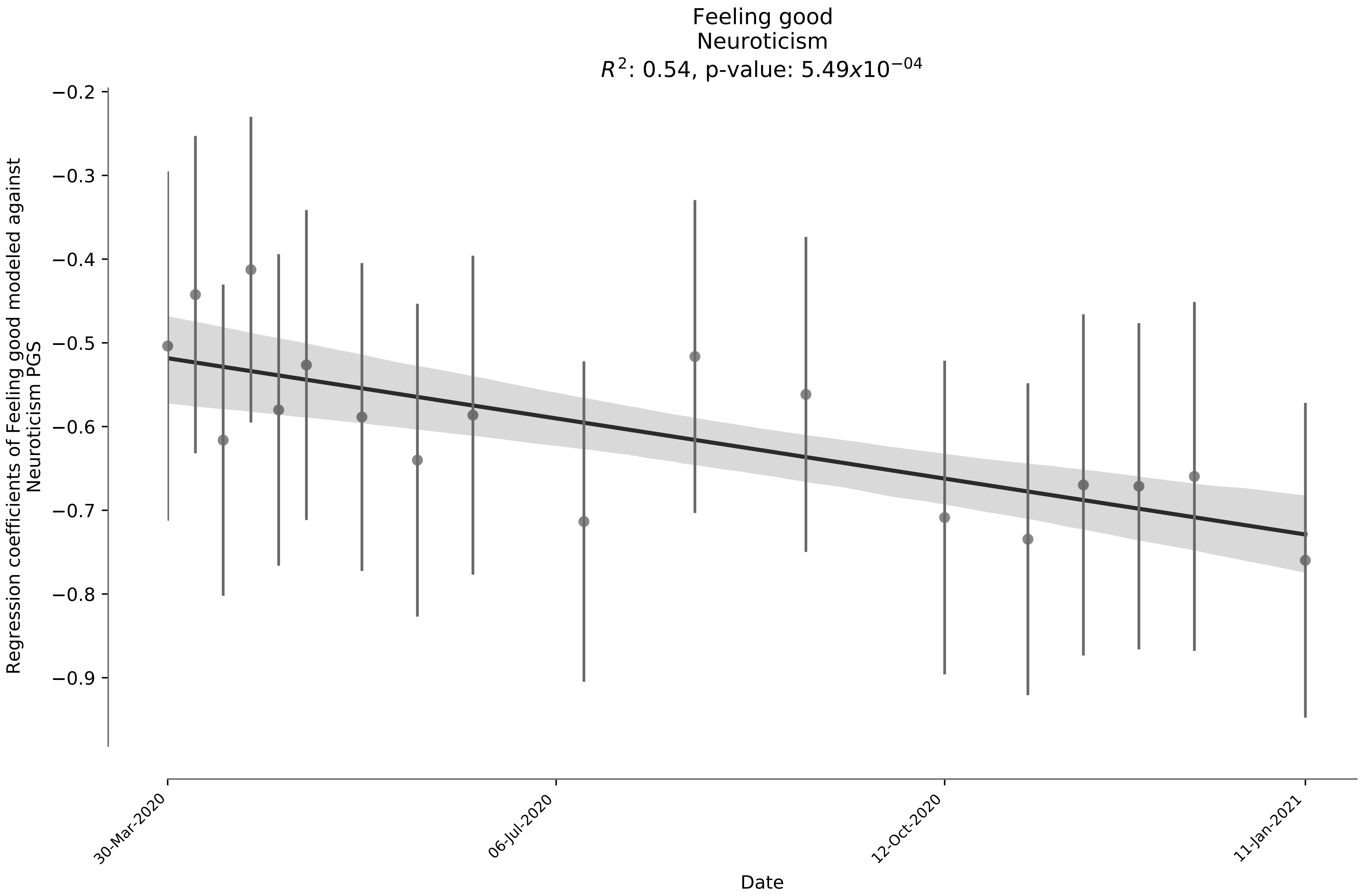
Feeling good
Neuroticism
R^2: 0.54, p-value: $5.49x10^{-04}$
Regression coefficients of Feeling good modeled against Neuroticism PGS
−0.2
−0.3
−0.4
−0.5
−0.6
−0.7
−0.8
−0.9
30-Mar-2020
06-Jul-2020
12-Oct-2020
11-Jan-2021
Date

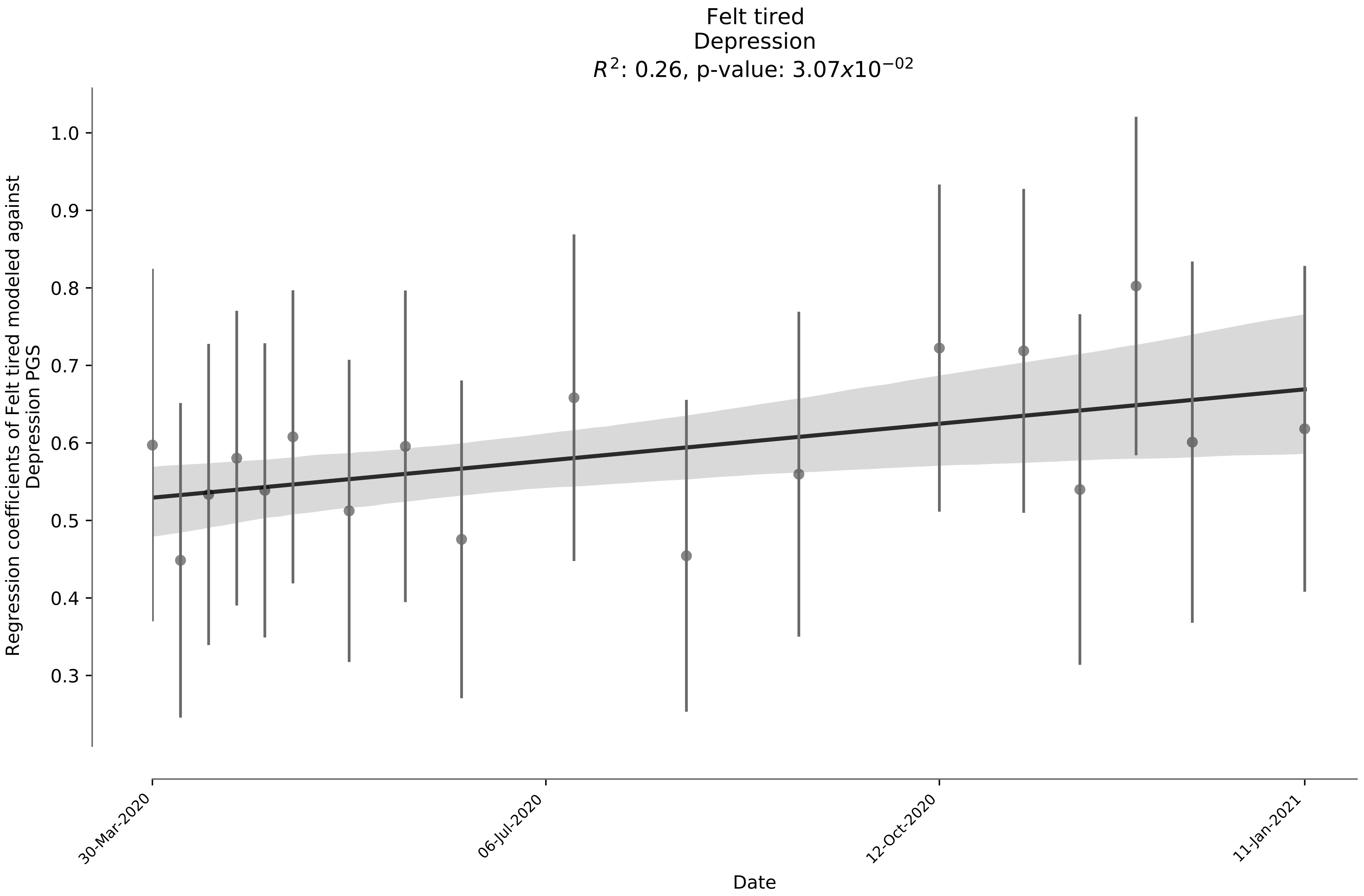
Felt tired
Depression
R^2: 0.26, p-value: $3.07x10^{-02}$
Regression coefficients of Felt tired modeled against
Depression PGS
1.0
0.9
0.8
0.7
0.6
0.5
0.4
0.3
30-Mar-2020
06-Jul-2020
12-Oct-2020
11-Jan-2021
Date

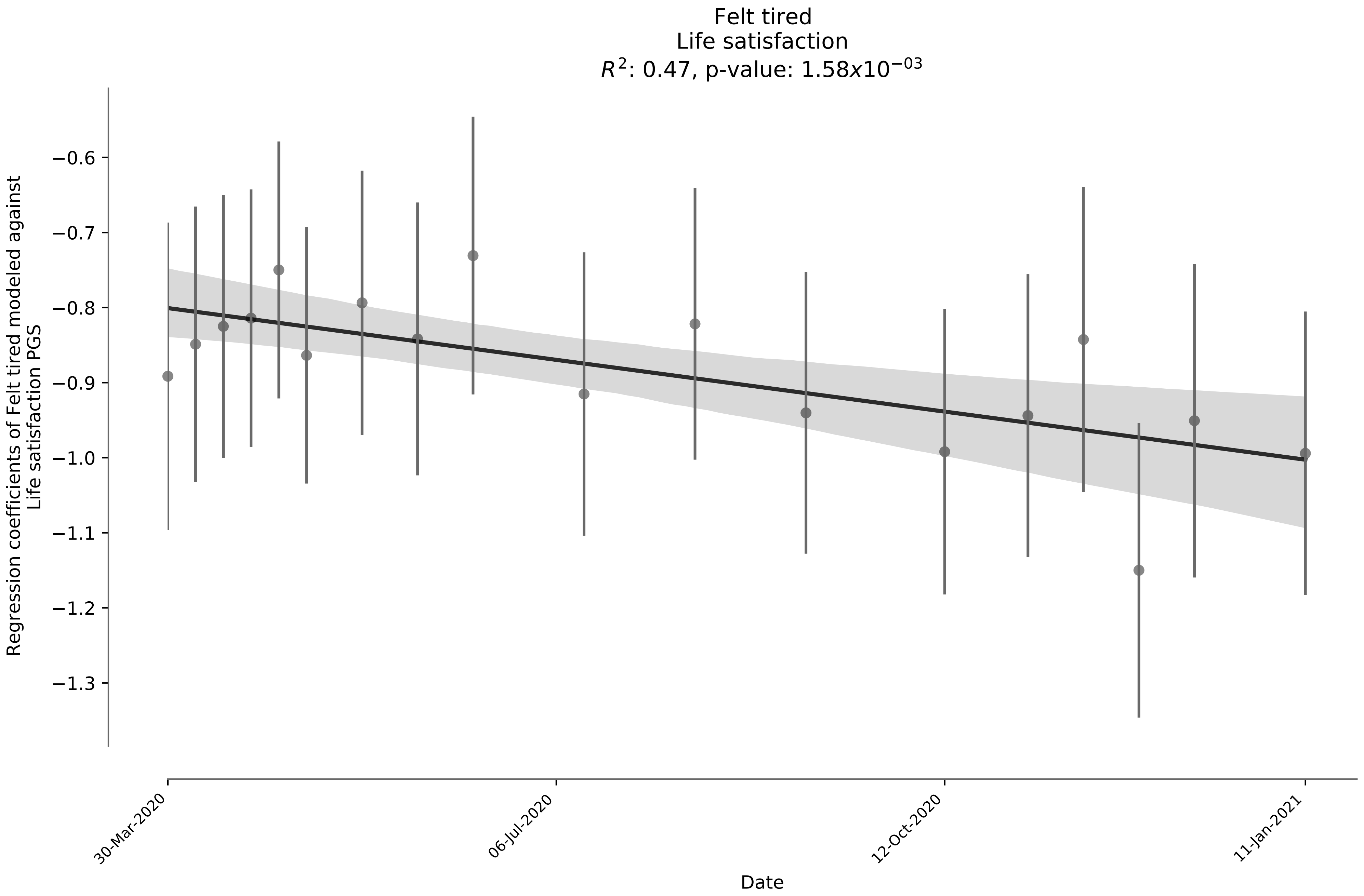
Felt tired
Life satisfaction
R^2: 0.47, p-value: $1.58x10^{-03}$
Regression coefficients of Felt tired modeled against
Life satisfaction PGS
−0.6
−0.7
−0.8
−0.9
−1.0
−1.1
−1.2
−1.3
30-Mar-2020
06-Jul-2020
12-Oct-2020
11-Jan-2021
Date

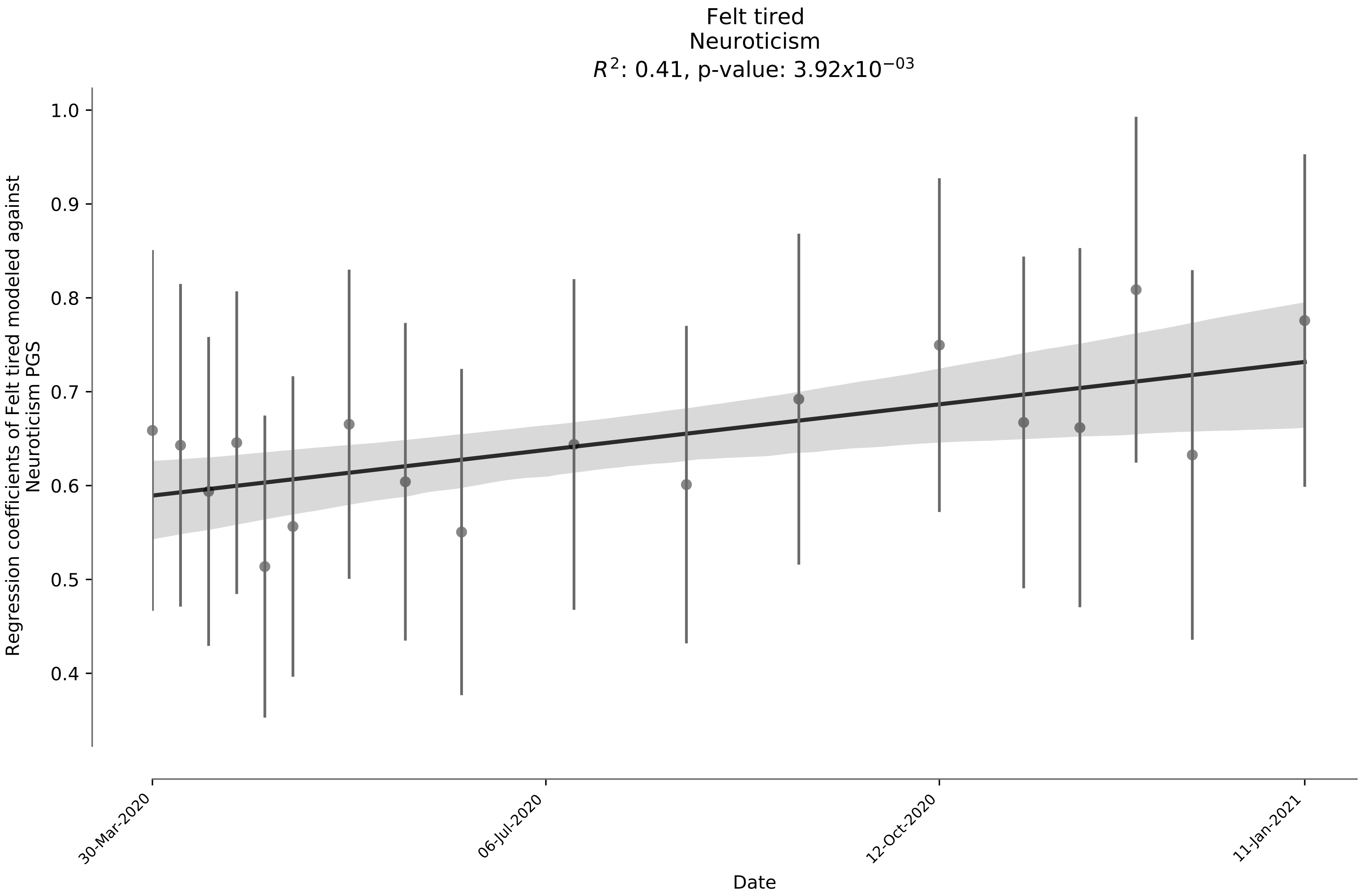

Felt tired
Neuroticism
R^2: 0.41, p-value: $3.92x10^{-03}$
Regression coefficients of Felt tired modeled against Neuroticism PGS
1.0
0.9
0.8
0.7
0.6
0.5
0.4
30-Mar-2020
06-Jul-2020
12-Oct-2020
11-Jan-2021
Date

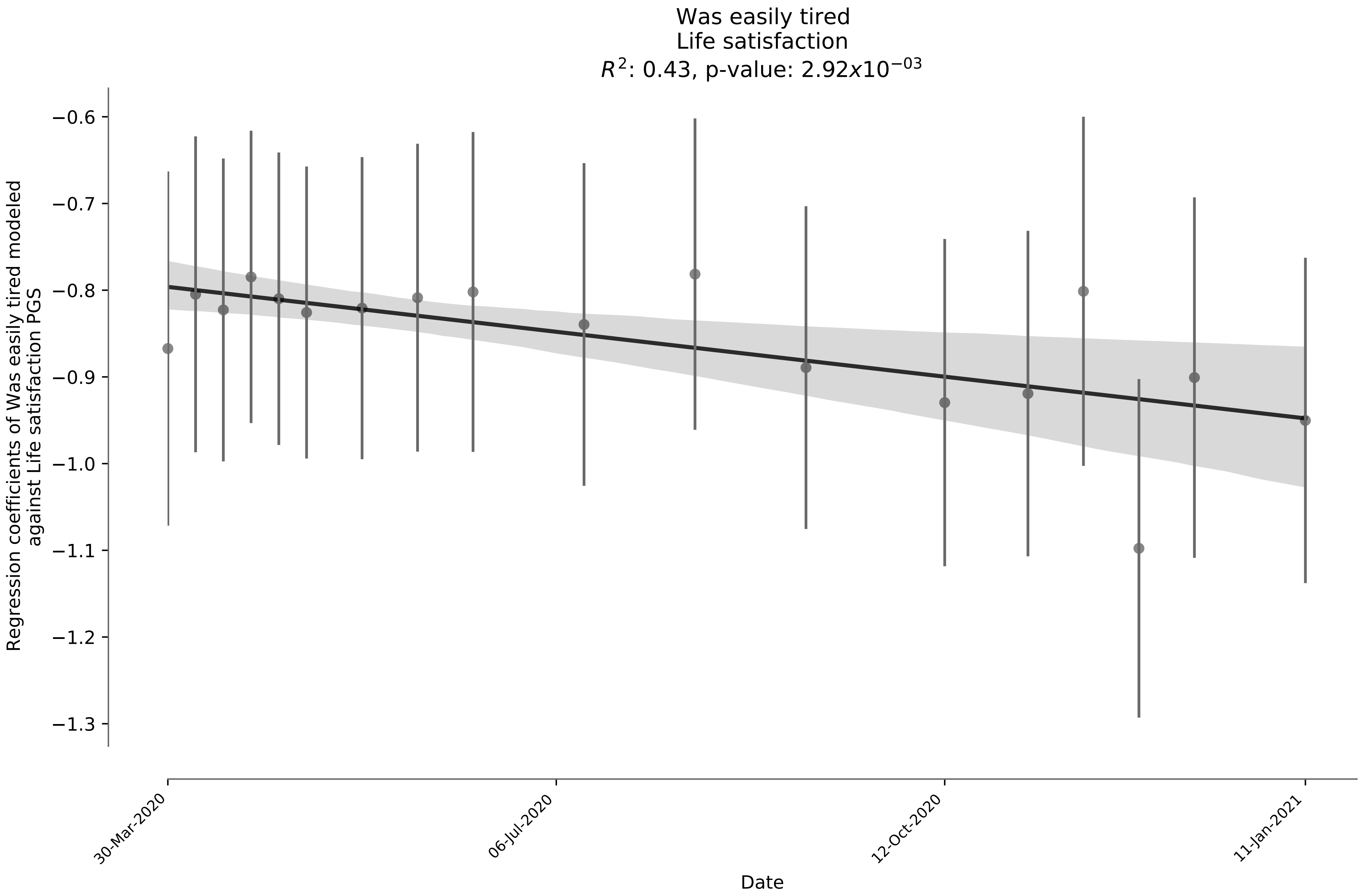
Was easily tired
Life satisfaction
R^2: 0.43, p-value: $2.92x10^{-03}$
Regression coefficients of Was easily tired modeled against Life satisfaction PGS
−0.6
−0.7
−0.8
−0.9
−1.0
−1.1
−1.2
−1.3
30-Mar-2020
06-Jul-2020
12-Oct-2020
11-Jan-2021
Date

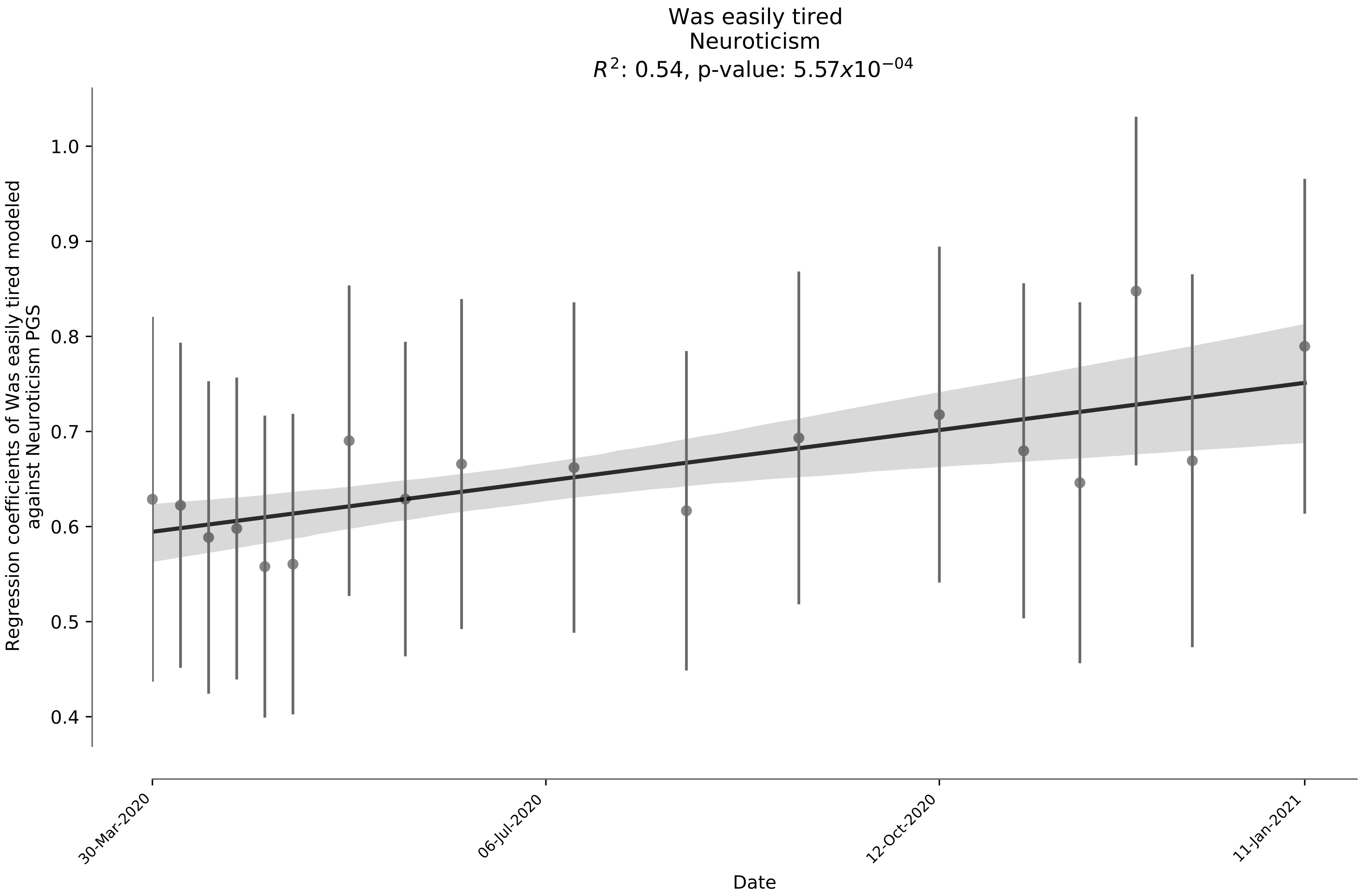

Was easily tired
Neuroticism
R^2: 0.54, p-value: $5.57x10^{-04}$
Regression coefficients of Was easily tired modeled against Neuroticism PGS
1.0
0.9
0.8
0.7
0.6
0.5
0.4
30-Mar-2020
06-Jul-2020
12-Oct-2020
11-Jan-2021
Date

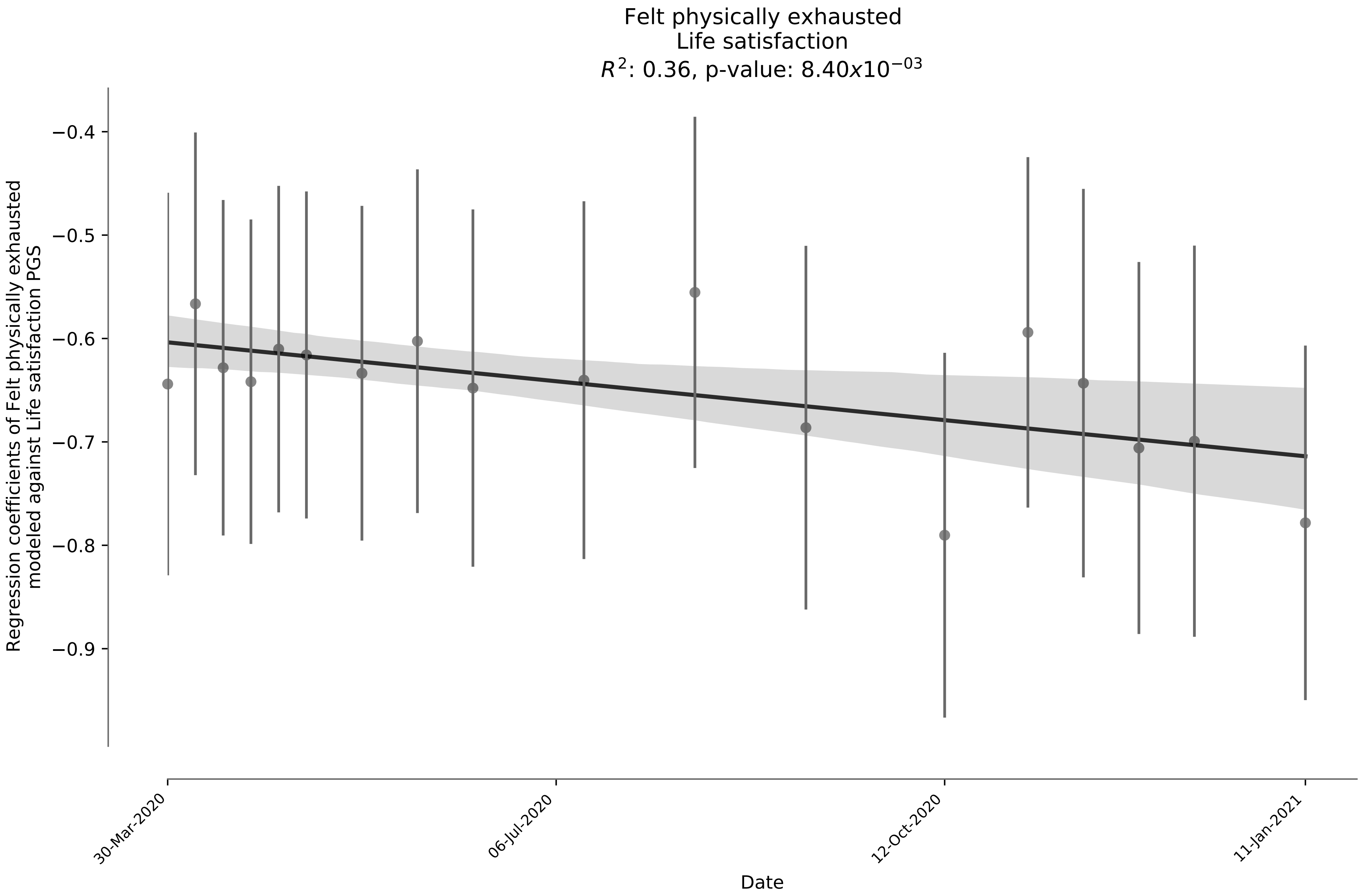

Felt physically exhausted
Life satisfaction
R^2: 0.36, p-value: $8.40x10^{-03}$
Regression coefficients of Felt physically exhausted
modeled against Life satisfaction PGS
−0.4
−0.5
−0.6
−0.7
−0.8
−0.9
30-Mar-2020
06-Jul-2020
12-Oct-2020
11-Jan-2021
Date
