# Supplementary material for: Increased genetic contribution to wellbeing during the COVID-19 pandemic
Source: PLoS Genet. 2022 May 19;18(5):e1010135. doi: 10.1371/journal.pgen.1010135 (PMC9119461; doi:10.1371/journal.pgen.1010135)
Supplement: S9 Fig — Polygenic scores (PGSs) for the participants that were included in this study compared to those that were invited to take part in the Lifelines COVID-19 questionnaires, but that were not included because they either did not respond or did not pass quality control. The p-values for Welch’s t-tests are shown to indicate whether the two groups differ significantly or not. Panel A, C and E show the participants that were genotyped using the Global Screening Array. Panel B, D and F show the participants that were genotyped using the HumanCytoSNP-12 array. Panel A and B show the 27,537 baseline samples compared to all other invited samples. Panel C and D show the 17,831 samples used in longitudinal analysis compared to all other invited samples. From these, 10 out of 34 p-values are smaller than an a priori Bonferroni corrected alpha of 0.05. This indicates that a small genetic bias is introduced for the willingness to fill-in the COVID-19 questions. Panel E and F show the 27,537 baseline samples compared to the 17,831 samples used in longitudinal analysis. Herein, the differences in Educational attainment and schizophrenia are highly significant. Should all baseline samples be included in the longitudinal analysis, such differential attrition would have biased our results. This suggests that selecting a confined set of samples for longitudinal analysis was appropriate. (PDF) [file pgen.1010135.s022.pdf]

A: Baseline samples versus not included samples (GSA)

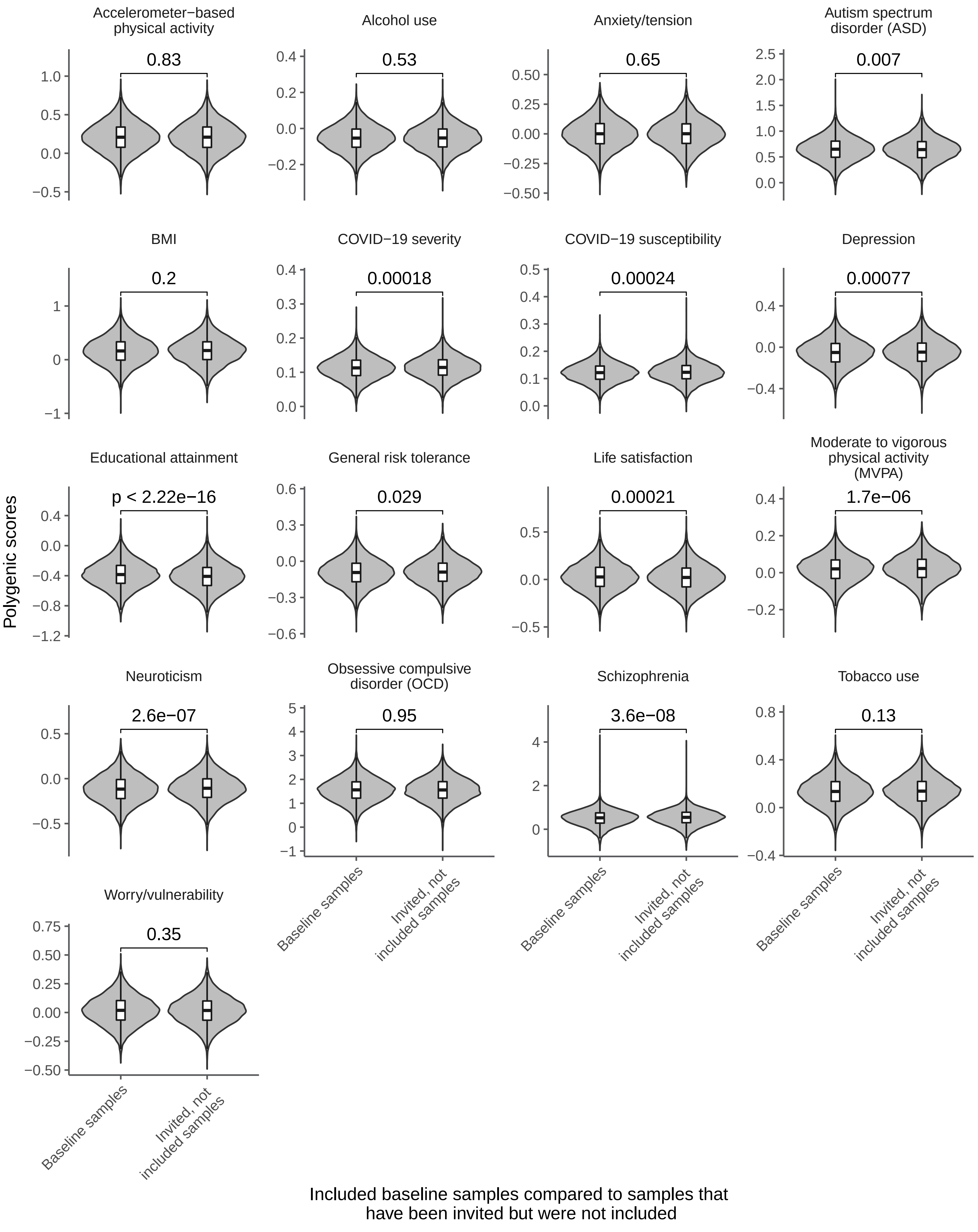

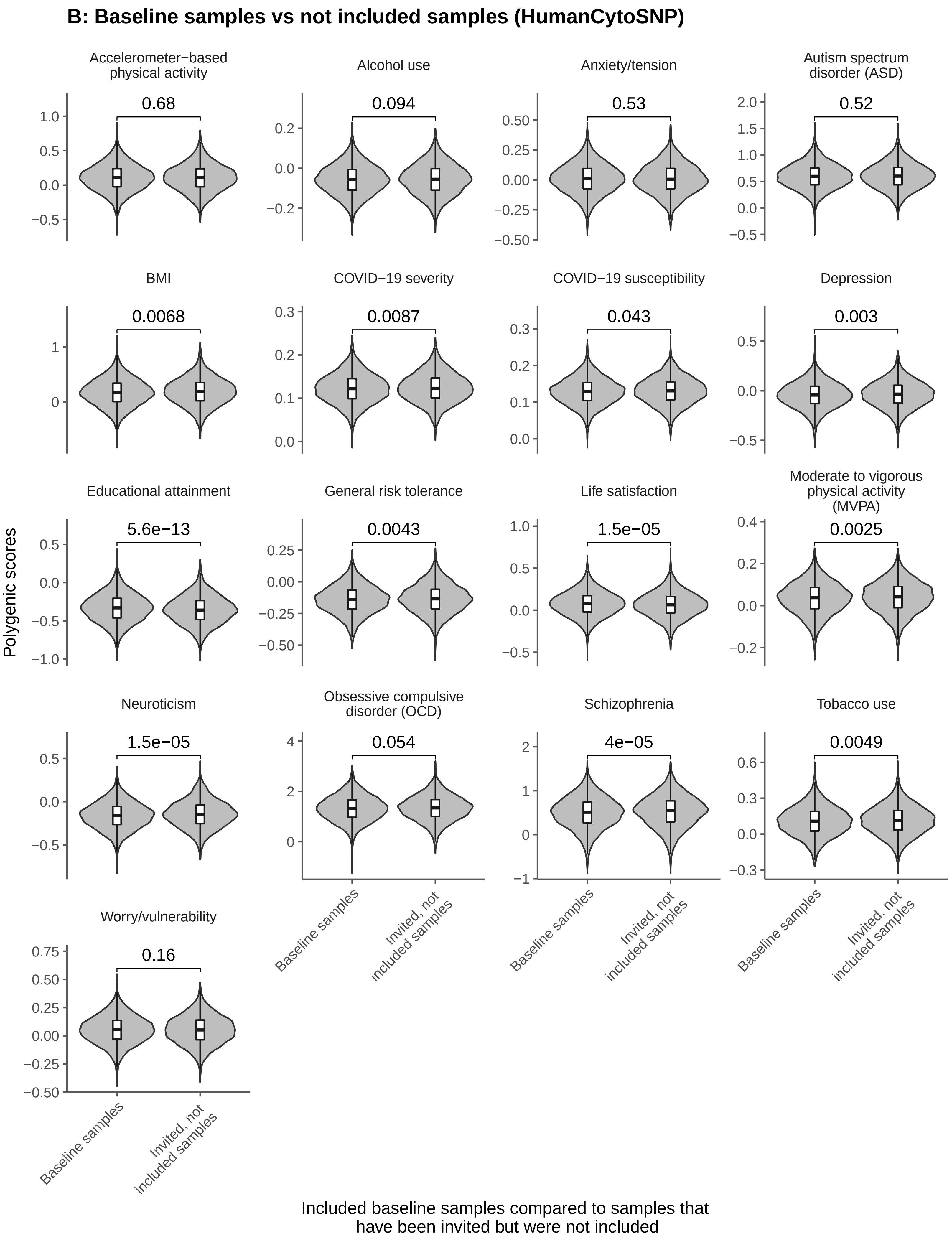
B: Baseline samples vs not included samples (HumanCytoSNP)
Accelerometer−based physical activity
0.68
Alcohol use
0.094
Anxiety/tension
0.53
Autism spectrum disorder (ASD)
0.52
BMI
0.0068
COVID−19 severity
0.0087
COVID−19 susceptibility
0.043
Depression
0.003
Educational attainment
5.6e−13
General risk tolerance
0.0043
Life satisfaction
1.5e−05
Moderate to vigorous physical activity (MVPA)
0.0025
Neuroticism
1.5e−05
Obsessive compulsive disorder (OCD)
0.054
Schizophrenia
4e−05
Tobacco use
0.0049
Worry/vulnerability
0.16
Polygenic scores
Baseline samples
Invited, not included samples
Included baseline samples compared to samples that have been invited but were not included

C: Longitudinal samples vs not included samples (GSA)

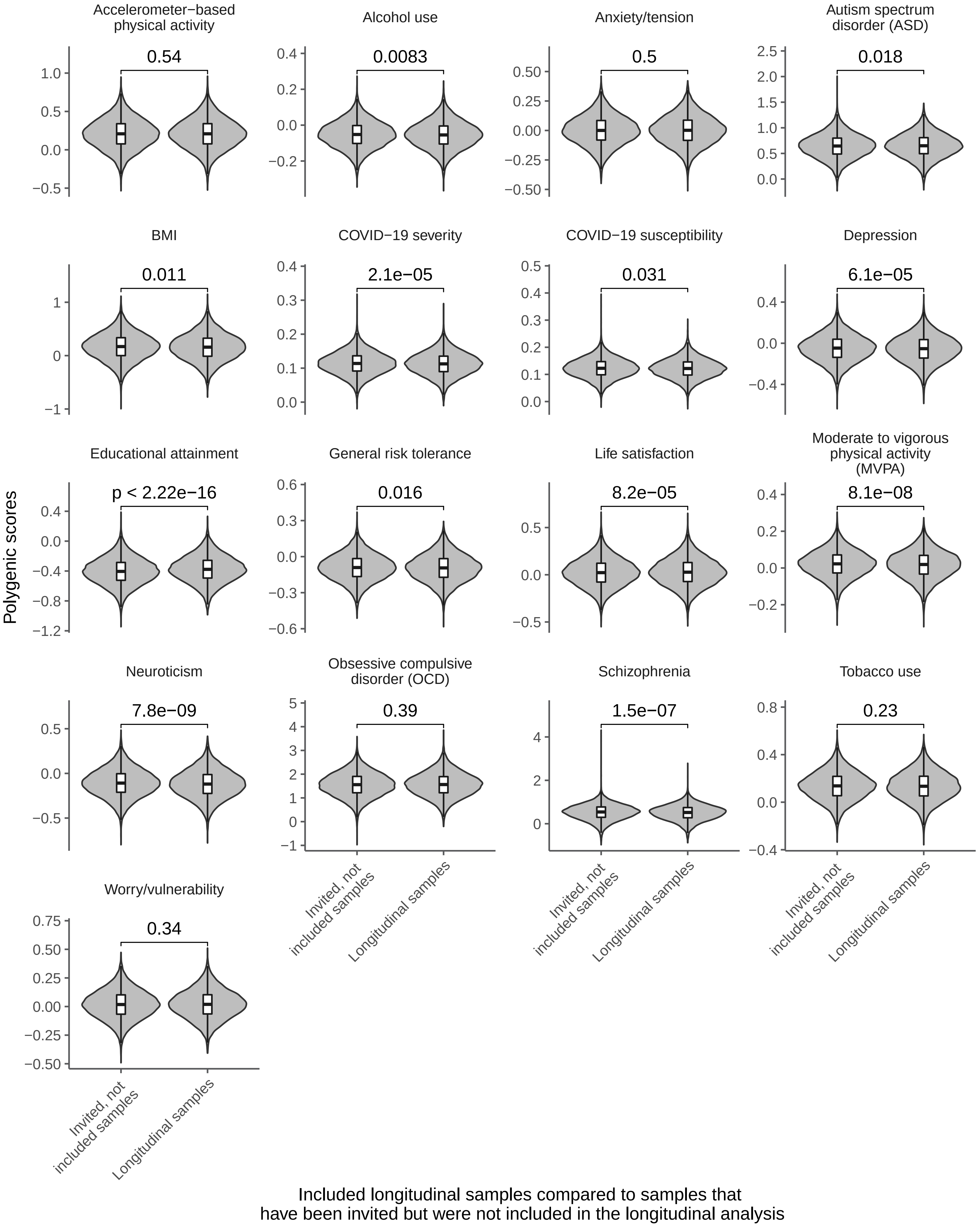

D: Longitudinal samples vs not included samples (HumanCytoSNP)

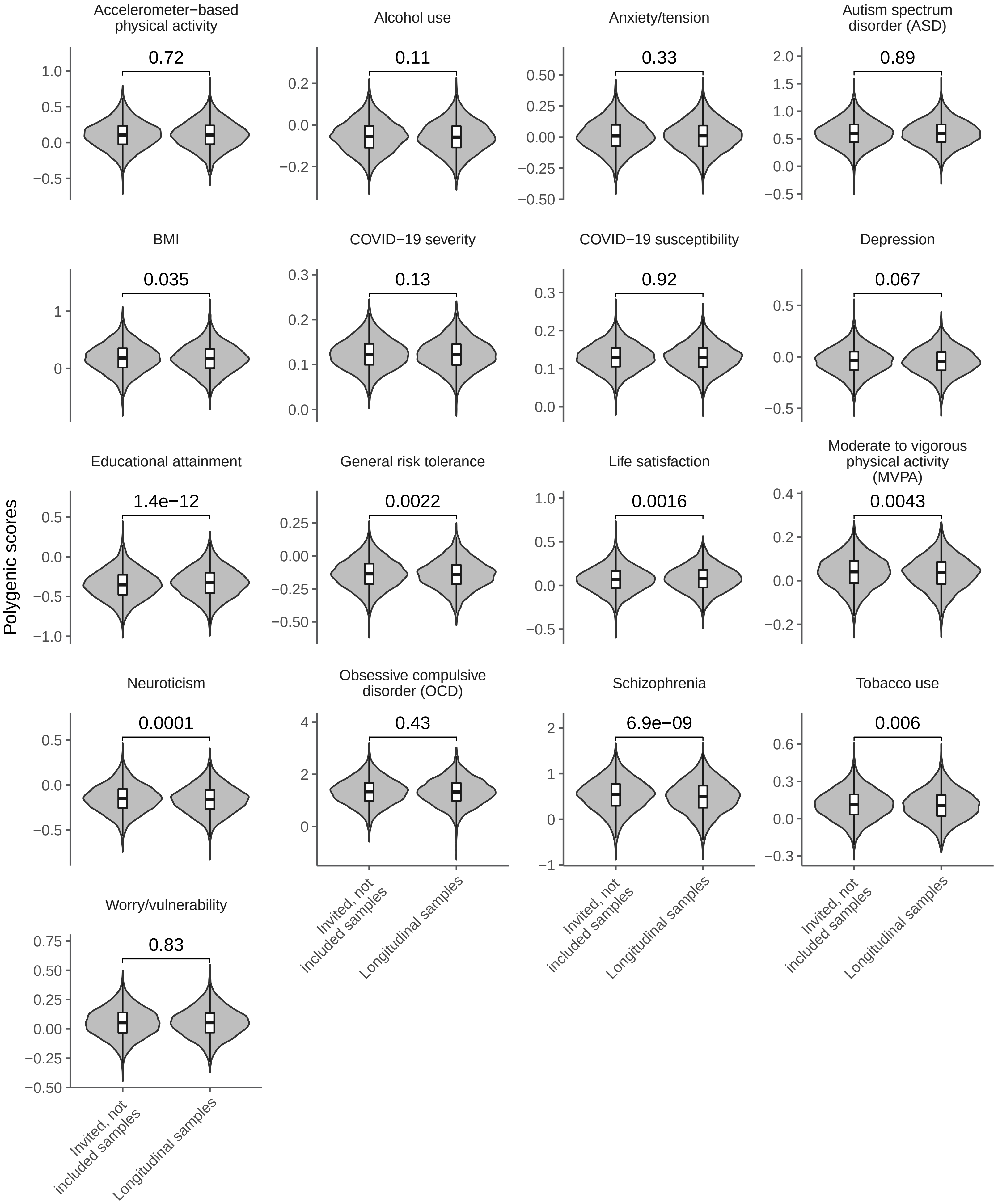

Included longitudinal samples compared to samples that have been invited but were not included in the longitudinal analysis

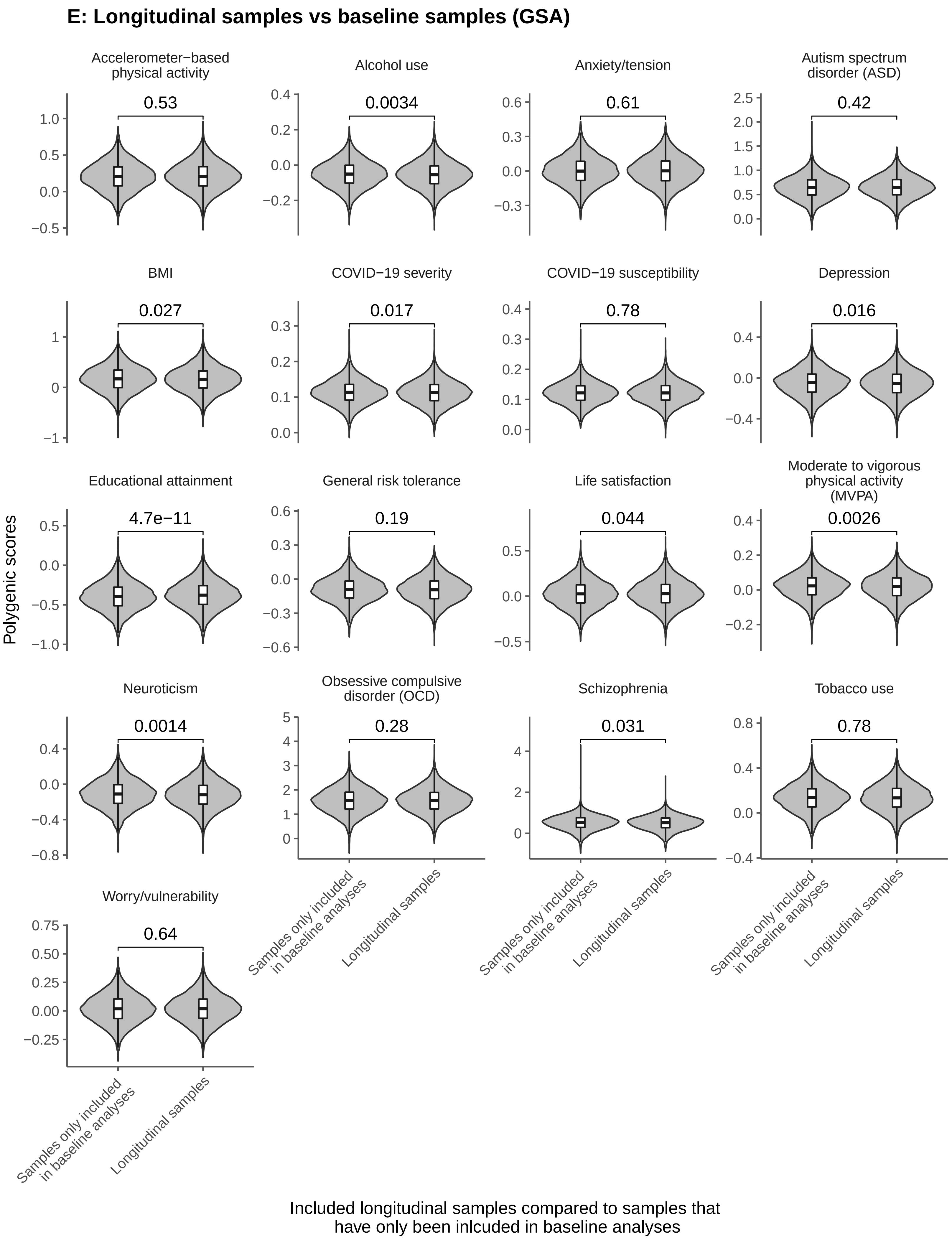
E: Longitudinal samples vs baseline samples (GSA)
Accelerometer−based physical activity
0.53
Alcohol use
0.0034
Anxiety/tension
0.61
Autism spectrum disorder (ASD)
0.42
BMI
0.027
COVID−19 severity
0.017
COVID−19 susceptibility
0.78
Depression
0.016
Educational attainment
4.7e−11
General risk tolerance
0.19
Life satisfaction
0.044
Moderate to vigorous physical activity (MVPA)
0.0026
Neuroticism
0.0014
Obsessive compulsive disorder (OCD)
0.28
Schizophrenia
0.031
Tobacco use
0.78
Worry/vulnerability
0.64
Polygenic scores
Samples only included in baseline analyses
Longitudinal samples
Included longitudinal samples compared to samples that have only been inlcuded in baseline analyses

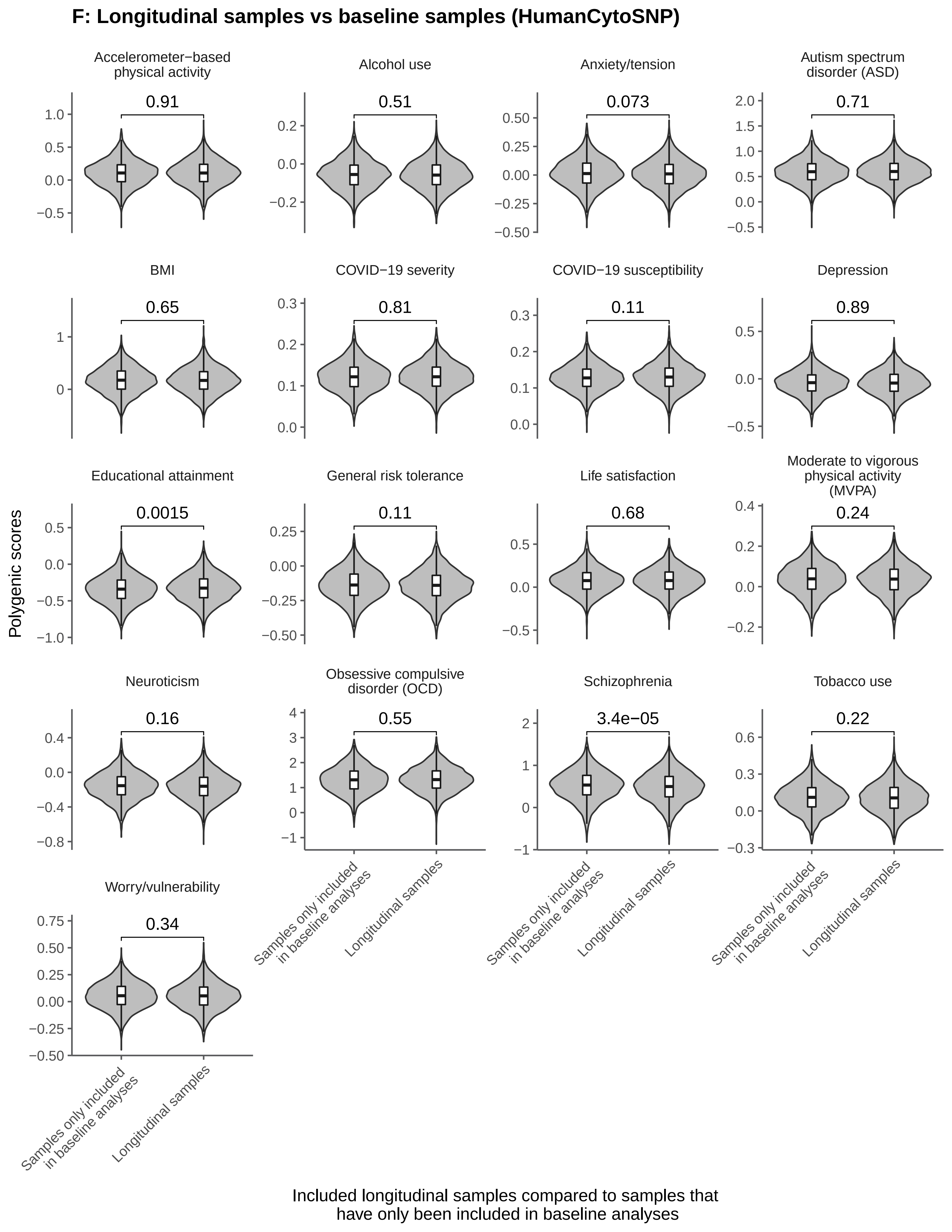

F: Longitudinal samples vs baseline samples (HumanCytoSNP)
Accelerometer−based physical activity
0.91
Alcohol use
0.51
Anxiety/tension
0.073
Autism spectrum disorder (ASD)
0.71
BMI
0.65
COVID−19 severity
0.81
COVID−19 susceptibility
0.11
Depression
0.89
Educational attainment
0.0015
General risk tolerance
0.11
Life satisfaction
0.68
Moderate to vigorous physical activity (MVPA)
0.24
Neuroticism
0.16
Obsessive compulsive disorder (OCD)
0.55
Schizophrenia
3.4e−05
Tobacco use
0.22
Worry/vulnerability
0.34
Polygenic scores
Samples only included in baseline analyses
Longitudinal samples
Included longitudinal samples compared to samples that have only been included in baseline analyses
