# Supplementary material for: Increased genetic contribution to wellbeing during the COVID-19 pandemic
Source: PLoS Genet. 2022 May 19;18(5):e1010135. doi: 10.1371/journal.pgen.1010135 (PMC9119461; doi:10.1371/journal.pgen.1010135)
Supplement: S1 Note — (DOCX) [file pgen.1010135.s025.docx]

1. Effect of the Schizophrenia-PGS on concern about the pandemic

At baseline, we observed that participants with a higher schizophrenia-PGS are more likely to be concerned about the pandemic (p-value: 5.0×10^-6^). Our longitudinal model shows that the magnitude of this effect diminishes over time (Fig. S4, p-value: 1.53×10^-3^). This effect is present in the sensitivity analysis using the PGS effect size per questionnaire but could not be confirmed using the 7,502 samples and 4 questionnaires.
