# Supplementary material for: Increased genetic contribution to wellbeing during the COVID-19 pandemic
Source: PLoS Genet. 2022 May 19;18(5):e1010135. doi: 10.1371/journal.pgen.1010135 (PMC9119461; doi:10.1371/journal.pgen.1010135)
Supplement: S2 Note — (DOCX) [file pgen.1010135.s026.docx]

1. Results of sensitivity analyses

To ensure the validity of our models we performed several analyses to validate our results. First, we selected 7502 samples that all completed questionnaires 4, 9, 14, and 19 to create a sample that is guaranteed not to suffer from attrition bias or non-random censoring. Using only these samples and the answers given at those four timepoints we reran the 11 significant longitudinal models (S9 Table). Four of these models (Life satisfaction-PGS on “Quality of life” and on “Felt good”; Neuroticism-PGS on “Felt good”; COVID-19 susceptibility-PGS on “Ever positive SARS-CoV-2 PCR test”) show significant replication of the PGS × time interaction effects (p-value ≤ 0.05), these all have the same direction. Out of the six models that do not show significant replication, five have the same direction of effect as the models on all the samples. One model did not converge. Clearly this analysis is underpowered, but since 9 out of the 10 validated models show an interaction effect in the same direction, we conclude that our results are not driven by attrition bias among the participants.

Secondly, we tested the validity of our longitudinal models by testing the association between each PGS and each question outcome separately for each questionnaire. This yielded up to 19 regression coefficients per PGS-question pair. We then correlated these regression coefficients per questionnaire to the date each questionnaire was sent out. This allowed use to ascertain in a very naïve model if the genetic contribution to an outcome was increasing or decreasing over time. We compared these directions to the 11 FDR significant PGS × time interactions we identified using the longitudinal models and found that all are nominally significant and show the same direction of effect.

Thirdly, we ascertained whether we could validate our results separately from PGSs using heritability analyses. Using the longitudinal mixed-effects models, we identified seven outcomes with one (or more) significant PGS × time interaction. Within this subset of seven, we calculated heritability estimates and variances explained by shared environment (S13 Table) based on household composition. The outcome “Ever positive SARS-CoV-2 PCR test”, did not yield reliable heritability estimates due to a too low number of cases at the start of the pandemic. We attempted to model changes over time in the remaining six outcomes. Herein, four outcomes did show a significant change over time (“Felt good”, “Felt physically exhausted”, “Felt tired”, and “Was easily tired”) (S10 Fig). Variances explained by shared family environment did not show significant changes over time (S11 Fig).
