# Supplementary material for: Increased genetic contribution to wellbeing during the COVID-19 pandemic
Source: PLoS Genet. 2022 May 19;18(5):e1010135. doi: 10.1371/journal.pgen.1010135 (PMC9119461; doi:10.1371/journal.pgen.1010135)
Supplement: S1 File — (PDF) [file pgen.1010135.s027.pdf]

Lifelines Corona Research Initiative

The authors wish to acknowledge the efforts of the Lifelines Corona Research Initiative and the following initiative participants:

H. Marike Boezen (1), Jochen O. Mierau (2,3), Lude H. Franke (4), Jackie Dekens (4,6), Patrick Deelen (4), Pauline Lanting (4), Judith M. Vonk (1), Ilja Nolte (1), Anil P.S. Ori (4,5), Annique Claringbould (4), Floranne Boulogne (4), Marjolein X.L. Dijkema (4), Henry H. Wiersma (4), C.A. Robert Warmerdam (4), Soesma A. Jankipersadsing (4), Irene V. van Blokland (4,7).

1) Department of Epidemiology, University of Groningen, University Medical Center Groningen, Groningen, The Netherlands

2) Faculty of Economics and Business, University of Groningen, Groningen, The Netherlands

3) Aletta Jacobs School of Public Health, Groningen, The Netherlands

4) Department of Genetics, University of Groningen, University Medical Center Groningen, Groningen, The Netherlands

5) Department of Psychiatry, University of Groningen, University Medical Center Groningen, Groningen, The Netherlands

6) Center of Development and Innovation, University of Groningen, University Medical Center Groningen, Groningen, The Netherlands

7) Department of Cardiology, University of Groningen, University Medical Center Groningen, Groningen, The Netherlands
