# Supplementary material for: Increased genetic contribution to wellbeing during the COVID-19 pandemic
Source: PLoS Genet. 2022 May 19;18(5):e1010135. doi: 10.1371/journal.pgen.1010135 (PMC9119461; doi:10.1371/journal.pgen.1010135)
Supplement: S2 File — (PDF) [file pgen.1010135.s028.pdf]

LifeLines Cohort Study

The authors wish to acknowledge the services of the Lifelines Cohort Study, the contributing research centers delivering data to Lifelines, and all the study participants.

Raul Aguirre-Gamboa (1), Patrick Deelen (1), Lude Franke (1), Jan A Kuivenhoven (2), Esteban A Lopera Maya (1), Ilja M Nolte (3), Serena Sanna (1), Harold Snieder (3), Morris A Swertz (1), Judith M Vonk (3), Cisca Wijmenga (1)

1) Department of Genetics, University of Groningen, University Medical Center Groningen, The Netherlands

2) Department of Pediatrics, University of Groningen, University Medical Center Groningen, The Netherlands

3) Department of Epidemiology, University of Groningen, University Medical Center Groningen, The Netherlands
