# Supplementary material for: Increased genetic contribution to wellbeing during the COVID-19 pandemic
Source: PLoS Genet. 2022 May 19;18(5):e1010135. doi: 10.1371/journal.pgen.1010135 (PMC9119461; doi:10.1371/journal.pgen.1010135)
Supplement: Attachment — Submitted filename: Response_to_reviewers.docx [file pgen.1010135.s029.docx]

Response to reviewers

We would like to the thank the reviewers for their critical review of our manuscript. Their suggestions and feedback have helped us to improve the manuscript. We have revised it accordingly and discuss these in detail below:

# Reviewer #1

- *The paper reports on PGS prediction for a number of traits that were assessed repeatedly during the COVID-19 pandemic. The sample derives from the Northern part of the Netherlands, which represents a part of the country that was not hit very hard by the virus, although lockdown was implemented in a similar manner as the rest of the country, which might have had differential effects: are there possibilities to collaborate with other biobanks in the Netherlands to test for replication?*

We fully agree that in the start of the pandemic the Northern part of the Netherlands was not hit very hard in terms of infections. However, after the initial first wave of infections the incidence in the Northern provinces rose substantially. Moreover, lockdown measures were the same across the country.

A replication in another Dutch biobank would indeed be of great value. Unfortunately, there is no biobank that we know of or that we have been in contact with that has the appropriate data to replicate our findings. Specifically, replication in the Dutch Twin Registry is unfortunately not possible, because there is no data for that cohort on the traits that we find to be significantly associated with PGSs. The Rotterdam Study constitutes of a confined set of individuals with a mean age of 70.2 at baseline. The average age of participants within our dataset is 54.2 (SD = 13.7). We are unable to replicate our results in our dataset when we confine ourselves to individuals aged 65 and above due to a lack of power. Since sample size of the Rotterdam study is also limited, replication in this cohort is also not realistic. Furthermore, prior to the initial submission we have reached out within the international COVID-19 host genetics initiative with a request for replication. We have not received any response from consortium members following this request.

- *The Lifelines study has 167,000 participants, this project included 27,537 participants: are these people with genotype data who returned at least one survey? (how many people were approached? Did the people with and without genotype data differ in the traits that were studied?).*

It is correct that 27,537 is the number of genotyped participants that responded at least once. We approached 139,713 lifelines participants to participate in the Lifelines Corona Research Initiative. We now included this number in the manuscript.

We have also performed a comparison of the studied traits between samples that had genotype data and samples that had no genotype data. This comparison is presented in Table S12, and we included these differences in the discussion. Here, we do see minor differences between people who responded and who did not.

- *Does the group of people that returned surveys include family members? Did their responses resemble each other? Did familial resemblance change during the pandemic?*

The group of people that returned surveys indeed include family members. To assess if their responses resembled each other, we have now done heritability analyses in which we calculated the variance explained by shared environment based on household composition as well as heritability estimates based on pedigree information (Table S13, Fig. S10 and Fig. S11). These analyses show a significant increase in the estimates for heritability over time for 4 out of 6 outcomes. An increase in the variance explained by shared environment is not significant. We included results and a discussion for these analyses in the manuscript.

- *There were 17831 Ss with genotype data and at least 2 surveys? (one survey in the beginning and one in the second half of the study). Do the PGS of the Ss without survey information differ from those who completed the surveys?*

The reviewer is correct. There are indeed 17,831 participants with genotype data and who returned at least two questionnaires, one in the first half and one in the second half of our study. We included a comparison of PGSs between samples that were invited but did not return questionnaires and samples that did return questionnaires in Fig. S9. We now also included a comparison of PGSs between samples that were included in the longitudinal analysis and samples that were only included in the baseline analysis.

- *I do not understand how figure S9 demonstrates “that sample selection based on quality control has been favorable for controlling false positives caused by loss to follow-up”.*

Regretfully the statement the reviewer refers to is unclear, and Fig. S9 that we submitted with our manuscript did not support this statement. We have now updated Fig. S9 to show differences in polygenic scores for samples that were only included at baseline compared to samples that were used in the longitudinal analyses (Fig. S9E and S9F). This comparison only shows minor differences, although the difference for educational attainment and schizophrenia is highly significant. Should all baseline samples be included in the analysis, such differential attrition would have biased our results. This suggests that selecting a confined set of samples for longitudinal analysis was appropriate.

- *Genotype data were used to obtain PGS for 17 traits: did the GWA discovery studies include Lifelines participants? (or their family members?). If yes, were LOO results obtained before PGS were constructed? Please note: figure 2 does not report on 17, but on 14 PGS. The choice for the discovery GWAS is not fully explained: why the focus on lifestyle and psychological traits?*

Lifelines was one of the 126 studies included in the BMI GWAS, and contributed approximately 1% of the total samples for the COVID-19 susceptibility GWAS.

Since the Lifelines samples represented, for each of these GWASs, only a very small part of the total number of samples, we expect that the effect of this on our PGSs is very limited. We therefore did not use LOO results before constructing the PGSs.

Fig. 2 indeed only shows 14 PGSs. We used two PGSs related to physical activity (Accelerometer-based physical activity & Moderate to vigorous physical activity) in our study. For clarity we used the maximum Z-score of these two PGSs for the physical activity column in this figure. Furthermore, we only show PGSs that have at least one significant association eliminating the PGSs for COVID-19 severity and OCD.

In principle, our study could have been performed using a GWAS on any other trait. However, for practical reasons we made a selection of traits we deemed interesting in this context.

- *Baseline / univariate analyses: on how many Ss, from which survey? (the first survey, any survey?).*

The reviewer asks which survey was used for the baseline analyses and how many samples were included in these analyses. We agree with the reviewer that we mistakenly have not included this information. For each of the variables in the baseline analysis we used the questionnaire wherein the question was asked first, with the exception of the ‘Ever positive SARS-CoV-2 PCR test’ variable. For this we included all self-reported positive SARS-CoV-2 PCR test results in any of the questionnaires. We have now included the necessary details on questionnaire number and sample size in Table S5.

- *The 2 arrays (N is given for the 27537 Ss by array but not for the 17831 Ss?) were imputed separately? Were SNPs for PGS harmonized across arrays? (e.g. same numbers of variants?).*

Indeed, genotyping has been executed using two different genotyping array platforms. We have now specified the number of samples per array for the different analyses in Fig. 1. We did not harmonize this data. Instead, we opted to use a meta-analysis to integrate these results. This way we could use all available variants to calculate the PGSs in the most optimal manner. Since a meta-analysis is used it does not matter that the performance of the PGSs might be different between cohorts. In theory, if attrition bias is correlated to the used array, this might result in a bias. But since we also run a validation analysis without attrition, we can safely conclude that array differences do not drive our findings.

- *Please explain: “We recoded questionnaire items to either an ordinal or dichotomous data type when this was not already the case”: continuous traits were not analyzed as continuous? (line 325 suggests they were?).*

We agree with the reviewer that the cited text is unclear and are sorry to have not written this more clearly. Original questions were not necessarily asked on a binary, categorical, ordinal or continuous scale. For each of the included questions we have attempted to recode answers so that we could analyze them as a binary, ordinal or continuous variable. Answer options that conflicted with an otherwise ordinal scale, such as ‘not applicable’, were excluded. We analyzed questions with an approximate continuous answer scale as continuous variables. Questions with a more limited number of answer options or for which the answers were not approximately normally distributed were analyzed as ordinal or binary variables. We have now adapted the manuscript to be clearer on how we recoded answers to make them suitable for our analyses.

- *Regression (Line 341): did confounders correct for familial relatedness? Or only sex and age?*

We did not correct for family relatedness in one of the confounders. In order to assess if our results hold when correcting for family relatedness, we have now performed the same analysis in a set of samples wherein we removed approximately all first- and second-degree relatives. We merged both genotyped sub cohorts and pruned the variants to maintain those that have been directly genotyped in either of the two genetic datasets, that are in approximate linkage equilibrium, and that did not contain missingness. From this dataset we applied PLINK to select 14,404 samples that were approximately unrelated (relationship coefficient ≤ 0.20). Within this limited set of samples, we found that 10 out of the 11 remain significant. Only the effect of life satisfaction on fatigue had a p-value of 0.054 but here the effect was in the correct direction.

Additionally, we have now also calculated heritability estimates on the different time points. Here we explicitly account for the pedigree structure and household composition. Using this analysis, we could also validate part of our models.

- *What is u(s) in regression equation?*

We inadvertently did not describe what the *u_s_* symbol depicts. We have now stated in our manuscript that the *u_s_* symbol denotes the sample random effect intercept.

- *How were missing data handled? (e.g. there were Ss with 11 vs 16 returned surveys?*

Missing data were handled with two different approaches. Our primary longitudinal analysis makes use of a generalized linear mixed-effects models. An advantage of such models is that it can handle data points from samples wherein multiple measurements are missing. The case highlighted by the reviewer wherein one sample returns 11 questionnaires and another returns 16 questionnaires is therefore perfectly aligned with the assumptions of these models. However, special attention is required to deal with attrition bias. For instance, one may consider a hypothetical group of samples that is more likely to stop returning questionnaires than another group. When the two groups are different from each other, the non-random dropouts can introduce false positives. In order to ascertain that our analyses did not suffer from attrition bias we performed additional analyses in which we confined ourselves to questionnaires 4, 9, 14, and 19 and all 7,502 samples that responded to each of these four timepoints. This is further described in our manuscript under ‘sensitivity analyses’ and additional results are provided in Note S2.

- *How many surveys were required to be included in the longitudinal analyses, or were all data analyzed?).*

In our quality control procedure, we initially excluded questions that were not answered by at least 95% of the respondents in the questionnaire. Thereafter, we only filtered data points on a per sample level. Confining ourselves to those questions that were answered by at least 95% of respondents, we ascertained how many answers were missing per sample-questionnaire combination. Questionnaires were excluded only on a per sample level when more than 5% of answers were missing for a particular sample. In our longitudinal analysis we only included samples that have returned a questionnaire in both the first half (returned before 1 September 2020; questionnaire 1-12) and the second half of the studied time period (returned after 31 August 2020; questionnaire 13-19). We have made sure that our quality control procedure is more clearly described in the methods section.

# Reviewer #2

*Immediate thoughts re. this paper are that this is a potentially interesting areas as it challenges the dynamic relationship between environmental and heritable contributions to trait variability using a universal (though variable) exposure. in COVID-19. From a basic point of view, one might anticipate a constriction of heritable variance for a complex outcome like well-being in the presence of an environmental challenge. However, in the presence of genetic contributions to COVID-19 experience itself (infection/outcome/the demographics of lockdown and exposure), there may be theoretical reasons as to why an inflation in heritable contributions to wellbeing. Whilst the authors present an examination of a series of PRS and their performance during the pandemic and longitudinal data available, there is no systematic assessment of the heritability of the outcomes in question here through time. This could be achieved using multiple methods and would give an important umbrella assessment of heritable contributions through time (and potentially by inceptive events during the pandemic).*

*The work presented looks to examine genetic contributions to wellbeing in a collection of ~25000 (17k with at least one questionnaire) participants with data collected through the course of the pandemic (lifelines). In brief, PRS were derived for a series of complex traits (including BMI, COVID risk and severity, EA, and psycho-behavioural traits) and these were associated with 288 questionnaire items running across the sampling period. This includes interesting association patterns such as that between PRS neuroticism/schiz’/depression and questionnaire outcomes re. personality traits and exhaustion. Further, the authors observe associations between other complex factors (EA, alcohol consumption, risky behaviour) and a series of variables recorded through the pandemic by questionnaire. This is interesting - though difficult to interpret clearly, however it does demonstrate the existence of analytical power across the data set available.*

*Followup analyses used the longitudinal nature of data collection to examine the possibility of varying genetic contributions to recorded outcomes through time (in those cases where there is association in baseline data). In this analysis, 11 associations showed some time dependence with 2 beating chance. Mean perceived quality of life varied and there was an apparent increase in the association between PRS for life satisfaction with some (who had high PRSs) showing resilience. This effect was reassessed in a sub analysis of 7500 participants with compete data at 4 time points.*

*The authors also show interesting relationships between COVID-19 PRS (susceptibility) and both incidence of infection (with increased incidence by score) and susceptibility - but with contributions to overall susceptibility (magnitude of effect) going down with time.*

*These are extremely interesting observations and the authors are conservative with both their interpretation of effect sizes and also the inferences drawn about these variable observations - this is great. However, there are a series of specific queries which need to be addressed:*

- *Given the nature of the analyses and the PRS being deployed here, the structure of the lifelines sampling frame will have an important effect on the presence and interpretation of estimates. One assumes that the ~25k respondents are not completely representative of the overall lifelines study and it would be good to have more analytical description of the actual sample and discussion on how this might influence outcomes. For example, the analysis of volunteers who are self-selected (a status likely correlated with many of the outcomes being assessed) may have an influence on the association seen through time in this data set.*

Here the reviewer raises concerns over both the representativeness of the respondents included in our analysis, and over the influence of participant characteristics on outcomes. The Lifelines population cohort has been shown to be representative of the Northern Dutch population^[1]^. In our cohort description we have shown that the Lifelines COVID-19 cohort differs from the full Lifelines population cohort and is therefore not fully representative of the Northern Dutch population. Moreover, we also see that the subjects in our longitudinal selection are on average older, slightly more often male, more often have a chronic illness, less often live alone and less often have children living at home, compared to the subjects in our baseline selection (Table S2).

Although we cannot conclude that the observed interaction effect is also present in other populations, we can conclude that this effect is present within the population that we studied. A potential bias could be that some phenotypes are correlated to loss of follow-up. To make sure that our findings are not confounded by this attrition bias, we also performed our analyses in a subset of individuals with measurements throughout the study period.

The latter concern the reviewer raises is arguably more important. We think that this concern has been properly addressed by the analysis that is described in our manuscript under sensitivity analyses and in Note S2. In this analysis we confined ourselves to questionnaires 4, 9, 14, and 19 and all 7,502 samples that responded to each of the four timepoints. This indicated that our results are not merely the result of self-selection of volunteers over time.

- *How are PRS chosen and constructed?*

In our manuscript we calculated polygenic scores (PGSs) for a number of traits. Here, the reviewer asks how these traits are chosen, and how PGSs were calculated. In our manuscript we chose traits that focus on lifestyle and psychology. This selection was made simply because we thought these were the most interesting to report on.

The construction of PGSs was done by executing a combination of PRS-CS and PLINK2 after a number of preprocessing steps. Within the genotype data we removed ambiguous, rare, and possibly erroneous variants based on minor allele frequency, call rate and imputation score. Variant identifiers were updated within both genotype data and GWAS summary statistics when necessary. We have adapted the methods section to include all steps in detail.

- *Related to the question above, what is the underlying correlation structure for heritable contributions to the risk factors (PRS) and outcomes being assessed? This is difficult for the questionnaire outcomes, but for the PRS, it is possible to generate estimates of shared heritability across the traits taken into PRS exposures and to ask about the relative independence of these. Ie. what is the rg across the GWAS that formed the PRS?*

In line with the request from the reviewer we have now calculated genetic correlations using LDSC. The genetic correlations are presented in Table S4.

- *The analysis of COVID-19 risk and or severity genetics is tricky. As to the former, whilst case control analysis may be possible, the nature of the cases (in particular) will be important here re. the changing nature of COVID-19 case status through time in this data set. It is likely the case that the cases in the later stages of he longitudinal data here are substantively different to those at the start (when the HGI was run) and that these differences lend to a lessening of overlap between the heritable contributions to case at later stages of the pandemic (notwithstanding the complications of vaccination). Further, the analysis of severity is necessarily a case-only assessment. Again, features which are associated with case status are likely also to be associated with the other questionnaire derived variables here. As a consequence, when stratifying GWAS on case status to undertake severity analyses in the HGI, biases can be introduced which alter the interpretation of the PRS severity and therefore the downstream associations with lifelines variables. These types of complexity do need to be considered in this paper as they will not only influence baseline associations, but also are likely to influence the longitudinal associations see in this work.*

Regarding the COVID-19 susceptibility risk analysis we fully concur with the considerations made by the reviewer, although the COVID-19 HGI release that we used is from January 2021 instead of the start of the pandemic. Still, we would like to remark that a large number of cases included in the COVID-19 susceptibility risk GWAS are contributed by clinical cohorts, making the GWAS even less representative for a population cohort like Lifelines. We have adapted the manuscript to more comprehensively discuss these complexities.

The reviewer suggests that the COVID-19 severity GWAS is necessarily a case-only assessment. However, we would like to remark that this is not the case for the B2 analysis that we used to construct our COVID-19 severity PGS. This instead compares hospitalized COVID-19 cases with the general population. We therefore argue that we did not introduce biases through this GWAS and the accompanying PGSs. However, we do concur with the reviewer that for this trait the interpretation is also complex. For instance, the Lifelines Corona Research data relies on participants themselves to report if they are hospitalized or not. Since this is not always feasible, our data is expected to be deflated for hospitalized cases.

- *One technical point - whilst the trends in PRS association seen are compelling, they are not so strong as to overcome the limited power in these analyses. Correlations with time are fascinating here, but there should be emphasis put on the scale of these differences (which is there I concede) and also the power of the analyses. Replication would of course be excellent - especially as there are a series of large studies with comparable - cross-pandemic - longitudinal data and genetics.*

The prediction accuracy of PGSs is still limited, which will have affected the observed correlations. In reality the association could be stronger than we’ve observed. We now also added a remark on the small effect sizes in the discussion of our paper. In order to get additional confidence in our findings we have now also performed heritability analysis where we account for the pedigree structure and shared households. Herein we could verify most trends that we also observe for PGSs.

With regards to a replication in another (Dutch) biobank, a replication in another Dutch biobank would indeed be of great value. Unfortunately, there is no biobank that we know of or that we have been in contact with that has the appropriate data to replicate our findings. Specifically, replication in the Dutch Twin Registry is unfortunately not possible, because there is no data for that cohort on the traits that we find to be significantly associated with PGSs. The Rotterdam Study constitutes of a confined set of individuals with a mean age of 70.2 at baseline. The average age of participants within our dataset is 54.2 (SD = 13.7) and 57.1 (SD = 12.8) for the baseline and longitudinal samples respectively. We are unable to replicate our results in our dataset when we confine ourselves to individuals aged 65 and above due to a lack of power. Since sample size of the Rotterdam study is also limited replication in this cohort is also not realistic. Furthermore, prior to the initial submission we have reached out within the international COVID-19 host genetics initiative with a request for replication. We have not received any response from consortium members following this request.

- *Lastly - referring to my point above - if it is possible to assess variation in heritable contributions to questionnaire responses and or exposures in this sample through time, then a coincident assessment of the variation in PRS effects but he same time points (i.e. superimposed onto heritability changes) would be an excellent addition.*

We agree that heritability analyses are a good addition to our manuscript and have now done these analyses in which we calculated heritability estimates based on pedigree information. We also took shared environment into account by simultaneously calculating the variance explained by shared environment based on household composition (Table S13, Fig. S10 and Fig. S11). These analyses show a significant increase in the estimates for heritability over time for 4 out of 6 outcomes. An increase in the variance explained by shared environment is not significant. We included results and a discussion for these analyses in the manuscript.

*Taken together, this is an interesting field and one which could take advantage of unique conditions.*

# Reviewer #3

*The main objective of the manuscript is to investigate the effect of genetic predisposition to certain traits and conditions on the physical and mental health of individuals affected by the COVID-19 pandemic. They conducted a study on 17,831 participants from Lifeline’s biobank with genetic data (total N=167,000). Ninet	een repeated questionnaires were sent out to the participants between March 30, 2020 and January 5 2021. This included total of 288 questions related to sociodemographic parameters, chronic diseases, COVID-19 infection, general health and symptoms, medication use, mental health, well-being, social life, and lifestyle. They computed polygenic scores on 17 traits and conditions by applying PRS-CS methods on GWAS summary statistics of these traits. Then, they conducted series of analyses to test for association between 1) PGSs and baseline questionnaire responses. 2) Longitudinal models on the significant PGS-Question from baseline. The analysis with the baseline questionnaire identified 30 significant associations with the most significant association between Neuroticism-PGS and “depression, schizophrenia, felt nervous, felt tired quickly”, Life satisfaction-PGS, and “excessive worrying, quality of life”. Other interesting finding includes a positive association between educational attainment and trust in the government response to COVID-19. Longitudinal models identified 11 FDR significant associations, and authors mainly focused on Life satisfaction PGS and perceived quality of life in the paper. It is an exciting finding showing that individuals with higher PGS for Life satisfaction have increased perceived quality of life compared to individuals with low PGS for life satisfaction even though mean the perceived quality of life decreased at the end of their follow-up period.*

*Main comments:*

- *Authors have presented an interesting study that gives insight on the impact of the COVID-19 pandemic on the perceived quality of life. Overall results and data are well presented, and the manuscript is written well.*

Thank you.

- *Although Figure 2 shows the association results, it would be great if Zscore could be made available in the supplementary files.*

We apologize for the absence of this supplementary information. We have added additional output files with Z-scores (Table S6).

- *Models are adjusted for chronic conditions. However, there was no mention of what and how the chronic disease was selected? Is BMI included in the chronic condition? As obesity has a high correlation with depression. I would assume adjusting the model with BMI would be necessary.*

The origin of the chronic conditions was indeed not described. We would like to apologize to the reviewer for this oversight and have added this to the manuscript. Whether or not a participant suffered from a chronic condition was determined from the question ‘Do you have a chronic health condition?’. BMI was therefore only included as a chronic condition if this was reported as such by the participant themself. GWASs like depression, neuroticism and life satisfaction all did not correct for BMI. Therefore, any genetic effect of BMI that also propagates to those traits will have been already captured by the GWASs. The corresponding PGSs would thus also include this effect. Therefore, including BMI as a covariate would merely act as a mediator variable reducing our statistical power.

- *Since the genotype data was generated on two different platforms, the authors said they performed the analysis on the two sets separately and then performed a meta-analysis. If the genetic data was imputed already, why didn’t author consider merging the data and run as a single set rather than a meta-analysis? This will increase the power. Of course, they need to adjust for the genotyping panel – which is standard practice in the field.*

Thank you for this suggestion to increase the power of our analysis. Indeed, a merged dataset could result in slightly more power. However, adding the genotyping platform as a confounder also cost a degree of freedom. Therefore, we expect very similar results for both a meta-analysis and the method proposed by the reviewer. Furthermore, a meta-analysis also appropriately deals with other aspects, for example differences in PGSs due to differences in coverage of the used genotyping platform and subsequent imputation accuracies. To be on the save side we opted to do a meta-analysis to make sure that the PGS performances do not influence our results.

- *Figure 3b and 4b shows the PGS vs Quality of life for 12,522 samples. However, earlier in the paper, they state ~17 samples were selected for the study. Can they clarify the discrepancy? Is the rest of the data in these two figures are from 12k samples or 17k?*

The reviewer is correct that there is a discrepancy in sample size. Fig. 3b, 3c, 4b and 4c show data for the 12,522 samples that were genotyped on the Global Screening Array (GSA) platform. The figures for the remaining samples, genotyped on the HumanCytoSNP array, are shown in the supplements (Fig. S4). Since we fitted the longitudinal model separately on these two cohorts and then performed a meta-analysis, we decided to visualize the results per array. For clarity, we chose to include only the visualization for the GSA samples in the main figure and to show that of the HumanCytoSNP samples in the supplements.

- *The paper does not follow the PLoS gen format. There is no introduction section in the paper. Authors should revise the paper and adhere to formatting guidelines.*

We acknowledge that our initial submission did not follow the appropriate format. We have revised our manuscript according to the PLOS Genetics formatting guidelines and as a result the manuscript now also includes an introduction section.

- *The discussion section is surprisingly short. My suggestion would be to restructure the paper and move several discussion points from the results section to the discussion.*

We have chosen to keep the results and discussion combined in one section and have critically assessed each subsection to include appropriate discussion.

- *Also, highlighting limitations and future directions for the paper would be great. There are several typos across the manuscript and figure. For example, Typo in figure 4b*

We have included discussion on the limitations and future directions for the paper in the final subsection of the results and discussion section. We thank the reviewer for pointing out this particular typo and have critically assessed our manuscript to eliminate further ones.

**Have all data underlying the figures and results presented in the manuscript been provided?**

Large-scale datasets should be made available via a public repository as described in the *PLOS Genetics* [data availability policy](https://eur03.safelinks.protection.outlook.com/?url=http%3A%2F%2Fjournals.plos.org%2Fplosgenetics%2Fs%2Fdata-availability&data=04%7C01%7Cc.a.warmerdam%40umcg.nl%7C4d9d4e7f4ae6472b79be08d97e21e6e8%7C335122f9d4f44d67a2fccd6dc20dde70%7C0%7C0%7C637679506346129763%7CUnknown%7CTWFpbGZsb3d8eyJWIjoiMC4wLjAwMDAiLCJQIjoiV2luMzIiLCJBTiI6Ik1haWwiLCJXVCI6Mn0%3D%7C1000&sdata=fAEPlY5niio4jStvlJh%2BY1TMHGpbMbGea%2F7KLg%2By0hA%3D&reserved=0), and numerical data that underlies graphs or summary statistics should be provided in spreadsheet form as supporting information.

Reviewer #1: **No:** Not entirely clear what the procedures are to access the data (through Life Lines in the Netherlands?)

We have revised the data availability section (shown below) to further clarify the procedures to access the data used in our project.

The individual-level data that support the findings in this publication were obtained from the Lifelines biobank under project application number ov20_0554. Due to privacy reasons the individual-level data can’t be made publicly available but can be made available upon reasonable request. This request should be directed to the Lifelines Research Office through email (research@lifelines.nl) or by using the application form on their website (<https://www.lifelines.nl/researcher/how-to-apply/apply-here>).

1. Klijs B, Scholtens S, Mandemakers JJ, Snieder H, Stolk RP, Smidt N (2015) Representativeness of the LifeLines Cohort Study. PLoS ONE 10(9): e0137203. https://doi-org.proxy-ub.rug.nl/10.1371/journal.pone.0137203
