# Supplementary material for: Increased genetic contribution to wellbeing during the COVID-19 pandemic
Source: PLoS Genet. 2022 May 19;18(5):e1010135. doi: 10.1371/journal.pgen.1010135 (PMC9119461; doi:10.1371/journal.pgen.1010135)
Supplement: Attachment — Submitted filename: ResponseToReviewers.docx [file pgen.1010135.s030.docx]

Response to reviewers

We would like to thank the reviewers for their feedback on the rebuttal of our manuscript. Below we provide a detailed response:

# Reviewer #1

- *The authors have responded well to some of the questions and concerns that were raised. However, their reply to the construction of PRS raises concerns about the validity of their approach and hence the results in the paper.

  The question was: "Genotype data were used to obtain PGS for 17 traits: did the GWA discovery studies include Lifelines participants? (or their family members?). If yes, were LOO results obtained before PGS were constructed?"

  Reply: "Lifelines was one of the 126 studies included in the BMI GWAS, and contributed approximately 1% of the total samples for the COVID-19 susceptibility GWAS. Since the Lifelines samples represented, for each of these GWASs, only a very small part of the total number of samples, we expect that the effect of this on our PGSs is very limited. We therefore did not use LOO results before constructing the PGSs."

  The expectation of the authors is unfounded. Please consult: Tutorial: a guide to performing polygenic risk score analyses. Choi SW, Mak TS, O'Reilly PF. Nat Protoc. 2020 Sep;15(9):2759-2772. doi: 10.1038/s41596-020-0353-1 and the references therein.*

*I cannot asses from the authors' reply: were BMI and covid-19 susceptibility the only two discovery GWAMA's in which Lifelines participated? (this seems unlikely).*

We have extended Table S3 to clarify to which GWASs Lifelines has contributed. Two columns were added: ‘contribution of Lifelines HumanCytoSNP samples’ and ‘contribution of Lifelines GSA samples’. It is important to note that the genotype data from the GSA samples has become available to very recently (early 2020) and was therefore only included in the COVID-19 susceptibility GWAS.

The samples for which we have HumanCytoSNP genotype data available have been used in three GWASs: the BMI GWAS as part of the GIANT consortium, the COVID-19 susceptibility GWAS as part of the COVID-19 Host Genetics Initiative and after a thorough check also the educational attainment GWAS. We previously missed the latter since the publication that we refer to only describes the newly added cohorts and Lifelines was part of the previous publication of which the data was also used in the meta-analysis from which we used the summary statistics. We thank the reviewer for bringing this to our attention. The Lifelines cohort was additionally used in the paper describing the Life satisfaction GWAS as part of the BIOS consortium, from which only methylation data was used for follow-up analyses.

In order to assess the impact of contributed Lifelines samples to the polygenic scores and the subsequent association results we aimed to ascertain whether our results for the BMI trait were driven by the HumanCytoSNP-12 subcohort. The HumanCytoSNP-12 subcohort has been included in the BMI GWAS. Therefore, the results obtained for this trait are possibly overestimated. Since the GSA subcohort is not included in the BMI GWAS, we are able to assess whether we can replicate our findings in the GSA subcohort. We were able to validate all 13 significant baseline results for BMI. In table 1, shown below, we see that all significant associations found are also significant and in the same direction when only using the GSA subcohort. This subcohort was not included in the BMI GWAS.

We performed the same analysis for the educational attainment trait. For this trait we found 76 Bonferroni significant associations. When confining ourselves to just the GSA subcohort we can replicate all associations with nominal significance and with the same direction of effect. All but 6 can also be replicated at a Bonferroni adjusted significance level (Table 2, shown below).

To address the concern of possible PGS association overestimation in the manuscript we have added the following paragraph to the discussion of our manuscript:

“It should be noted that the samples used in this study are also part of the GWASs for BMI, educational attainment, and COVID-19 susceptibility (Table S3). For the BMI and educational attainment GWASs this only holds for the samples genotyped using the HumanCytoSNP-12 array. Here, we found that all significant associations are also nominally significant (p-value ≤ 0.05) and have the same direction when only using the samples not included in the BMI and educational attainment GWASs. Most effects, 83 out of the 89, are even Bonferroni significant using only the GSA data. For the COVID-19 susceptibility we cannot rule out that the baseline association between the PGS and infection status is biased by the overlapping samples. However, this does not explain the change in PGS performance over time. ”

# Reviewer #3

*The revised version of the manuscript is much improved and authors have addressed all the comments aptly.

Minor comment: Figure S11 is not mentioned in the main text.*

We apologize for this oversight. We have added the following reference to this figure to the manuscript:

“For four outcome items […] we observed a significant increase of heritability over time (Fig. S10) without significant changes to the contribution of shared environment over time (Fig. S11).”

# Other changes

We referred to the preprint of the COVID-19 severity and COVID-19 susceptibility GWASs in Table S3 and in our manuscript. Since our first submission this preprint has been published. Therefore, we have removed the reference to the preprint and added a reference to the published paper.

Table 1 Baseline association results for 13 Bonferroni significant outcomes for the BMI trait broken down by subcohort. The table indicates that for all outcomes, the results for the GSA subcohort show the same direction of effect and can be replicated with a Bonferroni corrected alpha of 0.05 (3.85e-03).

| **Outcome** | **GSA subcohort correlation estimate** | **GSA subcohort p-value** | **HumanCytoSNP-12 subcohort correlation estimate** | **HumanCytoSNP-12 subcohort p-value** | **Meta-analysed correlation estimate** | **Meta-analysed p-value** |
| --- | --- | --- | --- | --- | --- | --- |
| Pre-pandemic (moderate) intensity physical activity | -2.2E-01 | 8.8E-04 | -3.5E-01 | 8.9E-04 | -2.6E-01 | 4.8E-06 |
| Noticeable appetite change or unintended weight change | 6.5E-01 | 5.0E-06 | 6.8E-01 | 5.0E-03 | 6.6E-01 | 8.4E-08 |
| Smoked | 7.6E-01 | 3.2E-08 | 7.5E-01 | 4.1E-04 | 7.6E-01 | 5.2E-11 |
| Washed hands for over 20 seconds more than six times per day | 3.8E-01 | 2.7E-06 | 3.8E-01 | 2.5E-03 | 3.8E-01 | 2.4E-08 |
| Being afraid of the important ground rights more easily swept aside in the future | 3.4E-01 | 1.2E-05 | 2.7E-01 | 2.6E-02 | 3.2E-01 | 1.1E-06 |
| Worried about there being a shortage of medication | 2.7E-01 | 7.4E-04 | 3.6E-01 | 2.7E-03 | 2.9E-01 | 7.9E-06 |
| Not feeling appreciated in society | 3.8E-01 | 3.7E-06 | 1.8E-01 | 1.6E-01 | 3.2E-01 | 3.3E-06 |
| Minutes of moderate-intensity or intensity physical activity | -2.5E-01 | 1.4E-04 | -3.4E-01 | 1.4E-03 | -2.7E-01 | 9.1E-07 |
| Had shortness of breath | 3.9E-01 | 1.3E-04 | 4.9E-01 | 2.6E-03 | 4.1E-01 | 1.3E-06 |
| Finding that the government must act tougher to reduce the number of infections | 3.5E-01 | 1.1E-05 | 5.6E-01 | 4.4E-06 | 4.1E-01 | 6.0E-10 |
| Being able to comply with the measure to wash hands for 20 minutes | 3.2E-01 | 6.3E-05 | 4.9E-01 | 7.4E-05 | 3.7E-01 | 3.4E-08 |
| Worry when feeling some sensation in the body | -3.7E-01 | 1.6E-05 | -2.3E-01 | 8.0E-02 | -3.3E-01 | 5.0E-06 |
| Having difficulty handling physical sensations in the body | -3.5E-01 | 2.4E-05 | -2.3E-01 | 6.4E-02 | -3.2E-01 | 5.5E-06 |

Table 2 Baseline association results for 76 Bonferroni significant outcomes for the educational attainment trait broken down by subcohort. The table indicates that for all outcomes, the results for the GSA subcohort show the same direction of effect and can be replicated at a nominal significance level of 0.05. All but 6 can also be replicated at a Bonferroni corrected alpha of 0.05 (6.57e-04).

| **Outcome** | **GSA subcohort correlation estimate** | **GSA subcohort p-value** | **HumanCytoSNP-12 subcohort correlation estimate** | **HumanCytoSNP-12 subcohort p-value** | **Meta-analysed correlation estimate** | **Meta-analysed p-value** |
| --- | --- | --- | --- | --- | --- | --- |
| Felt connected to all Dutch people | -2.41E-01 | 1.27E-02 | -7.69E-01 | 1.05E-07 | -4.05E-01 | 4.91E-07 |
| Appreciating social distancing in general | -4.20E-01 | 2.54E-03 | -9.23E-01 | 8.13E-06 | -5.77E-01 | 5.90E-07 |
| Complying with the measure of not visiting anyone in a high risk group | -3.70E-01 | 4.91E-03 | -9.53E-01 | 1.70E-06 | -5.47E-01 | 6.16E-07 |
| the freedom to go where i want / since the start of the intelligent lockdown, how badly did you miss the following? | -3.15E-01 | 5.58E-03 | -6.43E-01 | 1.45E-04 | -4.17E-01 | 9.87E-06 |
| Finding the pre-defined roadmap for the corona measures gives peace of mind | -3.75E-01 | 8.86E-04 | -6.84E-01 | 5.25E-05 | -4.70E-01 | 5.42E-07 |
| Having been able able to keep distance during a friend and/or family visit | -3.39E-01 | 3.03E-03 | -7.01E-01 | 3.61E-05 | -4.52E-01 | 1.86E-06 |
| Felt active | 4.88E-01 | 4.45E-06 | 4.48E-01 | 4.15E-03 | 4.75E-01 | 6.41E-08 |
| Not visiting vulnerable people for the next 10 days after a high-risk event | -4.53E-01 | 3.74E-05 | -7.51E-01 | 4.01E-06 | -5.46E-01 | 2.01E-09 |
| Finding companies should be protected from going bankrupt at all cost | -9.84E-01 | 5.15E-13 | -1.41E+00 | 1.84E-11 | -1.11E+00 | 2.06E-22 |
| Felt attentive | 7.09E-01 | 2.68E-11 | 9.13E-01 | 4.87E-09 | 7.74E-01 | 1.35E-18 |
| Pre-pandemic (moderate) intensity physical activity | 3.32E-01 | 3.74E-04 | 6.48E-01 | 3.88E-06 | 4.29E-01 | 3.40E-08 |
| Perceived threat of COVID-19 to Dutch citizen | -4.78E-01 | 7.94E-06 | -7.81E-01 | 2.40E-06 | -5.67E-01 | 2.76E-10 |
| Celebrating christmas in church before the pandemic | 1.37E+00 | 1.23E-24 | 1.17E+00 | 3.79E-09 | 1.31E+00 | 4.54E-32 |
| Having done volunteer work | 1.27E+00 | 2.93E-21 | 1.19E+00 | 1.58E-09 | 1.25E+00 | 3.24E-29 |
| Had difficulty breathing | -8.15E-01 | 1.40E-06 | -3.09E-01 | 2.37E-01 | -6.66E-01 | 2.67E-06 |
| Having (Foster)child(ren) in higher education living away from home | 1.13E+00 | 5.17E-07 | 1.61E+00 | 9.45E-08 | 1.30E+00 | 5.32E-13 |
| Has household member working outside the house | -7.81E-01 | 2.78E-10 | -6.96E-01 | 2.45E-04 | -7.56E-01 | 3.12E-13 |
| Has someone close with COVID-19 | 1.04E+00 | 4.15E-07 | 3.39E-01 | 2.70E-01 | 8.23E-01 | 1.41E-06 |
| Having quarantined or gone into (self-)isolation during second wave | 7.10E-01 | 2.33E-04 | 1.12E+00 | 2.24E-04 | 8.27E-01 | 3.63E-07 |
| Smoked | -1.50E+00 | 3.43E-14 | -1.29E+00 | 4.98E-06 | -1.43E+00 | 1.05E-18 |
| Taken medication | -4.99E-01 | 2.19E-05 | -4.62E-01 | 8.61E-03 | -4.87E-01 | 6.08E-07 |
| Number of visited or received guests older than 12 years and outside the own household during the christmas break | 8.68E-01 | 1.70E-13 | 1.16E+00 | 2.99E-11 | 9.59E-01 | 8.36E-23 |
| Use of public transport before coro | 1.26E+00 | 2.80E-26 | 1.14E+00 | 9.24E-11 | 1.22E+00 | 2.15E-35 |
| Current use of public transport | 8.41E-01 | 4.89E-07 | 1.34E+00 | 7.50E-08 | 9.95E-01 | 7.16E-13 |
| Washed hands for over 20 seconds more than six times per day | -8.52E-01 | 8.04E-14 | -1.20E+00 | 1.23E-12 | -9.60E-01 | 2.83E-24 |
| Washed hands more than six times per day | -4.68E-01 | 5.37E-05 | -8.01E-01 | 2.22E-06 | -5.75E-01 | 1.90E-09 |
| Self-assessment of personal health | 5.36E-01 | 7.51E-08 | 6.54E-01 | 1.66E-05 | 5.71E-01 | 6.93E-12 |
| Being afraid of short-term side effects of vaccition | -9.13E-01 | 2.51E-16 | -9.05E-01 | 2.57E-08 | -9.10E-01 | 3.87E-23 |
| Having fear of long-term adverse drug reactions upon vaccition | -8.98E-01 | 3.63E-16 | -8.85E-01 | 4.13E-08 | -8.94E-01 | 8.88E-23 |
| Being afraid of the important ground rights more easily swept aside in the future | -1.00E+00 | 3.01E-20 | -1.31E+00 | 7.00E-16 | -1.10E+00 | 5.81E-34 |
| Fear things will go wrong in society | -3.26E-01 | 6.07E-05 | -3.31E-01 | 7.43E-03 | -3.27E-01 | 1.41E-06 |
| Frustrated with how things go in society | -3.91E-01 | 2.09E-06 | -2.79E-01 | 2.55E-02 | -3.57E-01 | 2.07E-07 |
| Being relieved about there being a vaccine | 5.39E-01 | 3.01E-06 | 3.13E-01 | 6.34E-02 | 4.67E-01 | 9.44E-07 |
| Willingness to get vaccinated if I may adhare less strict to the measures | 4.30E-01 | 1.23E-04 | 4.40E-01 | 7.61E-03 | 4.33E-01 | 2.93E-06 |
| Worried about there being a shortage of medication | -3.92E-01 | 3.14E-04 | -7.61E-01 | 2.64E-06 | -5.07E-01 | 2.03E-08 |
| Avoiding parks and beaches | -5.22E-01 | 1.54E-04 | -1.12E+00 | 5.82E-08 | -7.07E-01 | 7.46E-10 |
| Doing every possible thing to help those who are infected | -3.62E-01 | 1.16E-04 | -7.79E-01 | 3.13E-08 | -4.91E-01 | 3.42E-10 |
| Not feeling obligated to comply with measures | -5.67E-01 | 2.63E-07 | -3.26E-01 | 4.71E-02 | -4.93E-01 | 7.37E-08 |
| Not kissing anyone on the cheek | 6.79E-01 | 8.60E-06 | 4.55E-01 | 4.58E-02 | 6.10E-01 | 1.52E-06 |
| Estimated chance to become infected | -4.84E-01 | 2.23E-05 | -8.15E-01 | 1.49E-06 | -5.87E-01 | 5.46E-10 |
| Estimated chance to infect someone else | -4.77E-01 | 2.63E-05 | -6.97E-01 | 3.19E-05 | -5.46E-01 | 6.15E-09 |
| Felt excluded by society | -4.66E-01 | 1.57E-05 | -5.25E-01 | 1.05E-03 | -4.84E-01 | 6.25E-08 |
| Finding that the government listening to scientists is important for the corona policy | 6.34E-01 | 1.24E-07 | 4.50E-01 | 1.15E-02 | 5.77E-01 | 6.74E-09 |
| Finding that reduced regular healthcare in favour of COVID-19 patients is justified | 7.30E-01 | 2.00E-11 | 7.22E-01 | 6.99E-06 | 7.27E-01 | 6.90E-16 |
| Finding facemasks lying around in the street and in ture problematic | -4.68E-01 | 7.90E-05 | -6.76E-01 | 1.38E-04 | -5.32E-01 | 6.63E-08 |
| Finding the corona vaccine scary | -8.53E-01 | 4.14E-14 | -6.11E-01 | 2.24E-04 | -7.76E-01 | 8.59E-17 |
| Recovering quickly after difficult times | 8.07E-01 | 1.48E-12 | 6.35E-01 | 1.36E-04 | 7.52E-01 | 1.31E-15 |
| Having faith in the response of the government to COVID-19 | 5.70E-01 | 3.94E-07 | 5.71E-01 | 6.90E-04 | 5.70E-01 | 1.05E-09 |
| Keep thinking about bad event that happened | -7.98E-01 | 3.79E-12 | -1.17E+00 | 3.12E-12 | -9.17E-01 | 4.13E-22 |
| Expecting a resurgence of the coronavirus because of christmas holidays | 4.31E-01 | 1.32E-04 | 4.19E-01 | 1.11E-02 | 4.27E-01 | 4.45E-06 |
| Finding that the money for financial support should be spend to improve healthcare | -7.97E-01 | 3.73E-13 | -9.88E-01 | 1.75E-09 | -8.56E-01 | 6.28E-21 |
| Belief that acceptance is necessary when something bad happens | 6.16E-01 | 2.52E-06 | 6.34E-01 | 8.89E-04 | 6.21E-01 | 8.34E-09 |
| Belief that is not that bad when something bad happens | 6.96E-01 | 1.20E-09 | 7.94E-01 | 1.93E-06 | 7.27E-01 | 1.29E-14 |
| Worried about getting sick | -4.74E-01 | 8.92E-07 | -4.88E-01 | 6.98E-04 | -4.79E-01 | 2.38E-09 |
| Being bothered about getting infected with COVID-19 | -5.56E-01 | 1.80E-06 | -1.20E+00 | 2.30E-12 | -7.60E-01 | 2.86E-15 |
| Felt low or depressed often | -1.39E+00 | 4.17E-06 | -9.18E-01 | 3.10E-02 | -1.23E+00 | 5.64E-07 |
| Having lost interest | -1.03E+00 | 7.69E-06 | -8.01E-01 | 1.90E-02 | -9.61E-01 | 5.18E-07 |
| Minutes of moderate-intensity or intensity physical activity | 4.62E-01 | 6.15E-07 | 7.19E-01 | 2.42E-07 | 5.41E-01 | 2.35E-12 |
| Average time spend sitting per working day | 5.07E-01 | 2.24E-07 | 4.51E-01 | 2.22E-03 | 4.90E-01 | 1.90E-09 |
| Felt inspired | 4.86E-01 | 3.57E-06 | 4.28E-01 | 5.50E-03 | 4.68E-01 | 6.89E-08 |
| Finding it is justified that young people are addressed as a group to comply the corona measures | -7.21E-01 | 6.98E-11 | -1.10E+00 | 2.27E-11 | -8.39E-01 | 6.30E-20 |
| Finding that more people die indirectly following the corona crisis than directly by COVID-19 | -7.84E-01 | 1.55E-12 | -8.89E-01 | 6.86E-08 | -8.17E-01 | 6.84E-19 |
| Finding that measures must be taken to reduce the number of infections among young people | -8.03E-01 | 5.92E-13 | -9.20E-01 | 2.90E-08 | -8.39E-01 | 1.17E-19 |
| Had muscle pain | -4.22E-01 | 4.00E-05 | -3.54E-01 | 2.13E-02 | -4.01E-01 | 2.67E-06 |
| Being able to comply with the measure of not giving handshakes | 6.39E-01 | 8.40E-05 | 5.60E-01 | 1.81E-02 | 6.14E-01 | 4.64E-06 |
| Not giving handshakes | 1.18E+00 | 1.95E-05 | 1.05E+00 | 1.01E-02 | 1.14E+00 | 6.39E-07 |
| Had shortness of breath | -6.84E-01 | 1.99E-06 | -8.10E-01 | 1.51E-04 | -7.23E-01 | 1.37E-09 |
| Perceiving others as a threat to personal well-being | -5.52E-01 | 3.62E-09 | -6.41E-01 | 4.92E-06 | -5.80E-01 | 9.79E-14 |
| Finding the government does not take economic interests sufficiently into account during the corona crisis | -7.27E-01 | 7.34E-11 | -6.59E-01 | 7.20E-05 | -7.06E-01 | 2.53E-14 |
| Finding that the government must act tougher to reduce the number of infections | -9.05E-01 | 1.57E-16 | -1.33E+00 | 6.16E-16 | -1.04E+00 | 7.33E-30 |
| Finding vaccines contribute to the return to the normal living situation | 6.88E-01 | 1.96E-09 | 3.12E-01 | 6.10E-02 | 5.67E-01 | 1.92E-09 |
| Thinking that a corona vaccine will have serious side effects | -8.70E-01 | 4.38E-15 | -6.04E-01 | 1.83E-04 | -7.84E-01 | 9.24E-18 |
| Thinking that vaccines in general have serious side effects | -1.07E+00 | 4.44E-22 | -7.72E-01 | 1.89E-06 | -9.76E-01 | 1.51E-26 |
| Being able to comply with the measure to wash hands for 20 minutes | -5.51E-01 | 1.17E-06 | -9.88E-01 | 3.83E-09 | -6.88E-01 | 2.36E-13 |
| Being able to comply with the 1.5 metre rule when visited at home | -4.03E-01 | 5.69E-04 | -5.16E-01 | 2.94E-03 | -4.38E-01 | 6.20E-06 |
| Finding that young people can do what they want if they keep enough distance | 3.89E-01 | 2.35E-04 | 4.28E-01 | 6.24E-03 | 4.01E-01 | 4.68E-06 |
